# A global empirical study on how street networks facilitate driving longer distances

**DOI:** 10.1038/s41598-023-45236-7

**Published:** 2023-10-24

**Authors:** Gabriel Maia, Caio Ponte, Carlos Caminha, Lara S. Furtado, Hygor P. M. Melo, Vasco Furtado

**Affiliations:** 1 Applied Informatics Department, University of Fortaleza, Fortaleza, 60811-905 Brazil; 2 Faculdade de Economia, Administração, Atuária e Contabilidade (FEAAC), Federal University of Ceará (UFC), Fortaleza, Brazil; 3 Graduate Department of Transportation Engineering, Federal University of Ceará, Fortaleza, 60020-181 Brazil; 4 Sony Computer Science Laboratories Rome, Joint Initiative CREF-SONY, Centro Ricerche Enrico Fermi, Via Panisperna 89/A, 00184 Rome, Italy; 5 Centro Ricerche Enrico Fermi, Via Panisperna 89/A, 00184 Rome, Italy; 6 Center for Theoretical and Computational Physics, University of Lisboa, 1649-004 Lisboa, Portugal

**Keywords:** Information theory and computation, Engineering

## Abstract

We simulated over 200 cities worldwide to investigate how the street network affects vehicle routes. We demonstrate that there is a ubiquitous super-linear relationship between time and distance when optimal route are chosen. More precisely, the average speed will be higher for longer trips when compared to shorter trips, showing that the street network makes driving further faster. We attribute this phenomenon to the spatial arrangement of extensive street segments that eliminate deceleration points. These results underscore the importance for cities to consider the distribution of deceleration-free streets while mitigating any negative impact on sustainability. To ensure efficient transportation planning and engineering, innovative approaches are necessary to facilitate the flow of goods and services while adhering to sustainable mobility principles.

## Introduction

Recent urbanization has facilitated the growth of human mobility, leading to the emergence of thriving economic and social urban centers^1,2^. However, alongside its benefits, urbanization presents challenges and potential negative impacts on mobility, traffic, and sustainability^3,4^. One notable concern is urban sprawl, which arises when rapid and unplanned urbanization encroaches upon rural and green areas, often driven by speculative practices^5^. In response to this issue, contemporary planning, guided by New Urbanism principles, advocates for livable centers, walkable neighborhoods, smaller building blocks, and denser concentric cities to foster sustainable and traffic-free environments^6,7^. To support these objectives, sustainable mobility plays a crucial role in designing urban networks that optimize transportation structures^8–13^ while enhancing well-being^14–17^, thereby influencing economic, social, and environmental indicators tied to sustainable development goals^18^.

Creating dense, vibrant, and functional urban centers while integrating sustainable mobility into planning processes remains a significant challenge. However, the emergence of Smart City technologies, including 5G, Artificial Intelligence, and the Internet of Things, presents new opportunities for understanding transit patterns and calculating novel mobility indicators^19^.

Moreover, Smart City solutions play a vital role in promoting sustainable development by ensuring efficient management of natural resources and equitable access to essential services for all citizens^20^. Data-driven policies on mobility can greatly benefit from simulation tools and advanced algorithms, thus advancing the cause of sustainable mobility^21^. When considering sustainable mobility indicators, an effective road network should minimize surface area usage, reduce time wasted in congestion, and shorten commuting travel times^22^. Additionally, it is important to encourage shorter average travel distances and discourage dispersed land use patterns, as they lead to longer trips and increased demands for transportation infrastructure space^23^.

Sustainable mobility, in essence, assesses street efficiency not solely based on speed, but also aims to achieve “reasonable and reliable travel times, even if it means slowing down movement”^22^. It is crucial to weigh the quantifiable advantages such as speed, flexibility, and accessibility to remote areas against the economic costs, environmental impact, and social equity concerns^18^. Interestingly, travelers often prioritize travel time over physical distance^24,25^, and this perception of distance can vary depending on street elements like intersections and stop signs^26^. As a result, the design and layout of the street network can influence drivers to opt for longer distances if they can reach their destination faster.

To achieve sustainable development goals, planning principles must consider how travel speed, time, and street infrastructure contribute to the creation of dense, multifunctional urban centers while curbing urban sprawl^23^. Physical characteristics of the street network, including connectivity, promote smooth traffic flow, reducing travel times and delays^27^. Moreover, expanding the street network in accordance with sustainable mobility factors involves accommodating vehicles from low-density areas and understanding how the existing infrastructure can either facilitate or deter sprawl.

Despite these considerations, there is a dearth of studies exploring how urban street expansion, street signage, and capacity affect the speed of motorized vehicle trips within cities. This research aims to bridge that gap by conducting an empirical experiment using computational advancements to simulate vehicle trips. The study measures the time and distance of routes, taking into account the existing street layout, to shed light on phenomena associated with urban morphology, an increasingly critical aspect in the context of Smart Cities.

To investigate this, we analyze street network data to examine how the spatial distribution of Deceleration Points (*DP*) and the street typology, which is correlated with specific permitted speeds, influence the duration and distance of trips. Simulations of motorized trips were conducted for 237 cities worldwide, where drivers follow the shortest route without traffic but considering variables related to the built properties of streets, such as the presence of traffic lights, stops, intersections, and permitted driving speeds for different road types (e.g., highways, motorways, residential areas, etc).

These simulations allowed us to calculate a $\beta$ exponent, from a power-law relationship between time and distance. For all cities studied, the $\beta$ accounted for a super-linear relationship, meaning that, given a specific time, the average distance traveled in each simulated trip increases at a proportionally faster rate than its duration. For instance, a trip with double the time typically covers more than double the distance. To account for potential limitations in simulating drivers’ paths using shortest routes, we incorporated traffic data in our simulations of nine cities. This approach underscored an even stronger super-linear relationship between time and distance. Additionally, we explore certain cities based on $\beta$, average street speed, and size (built footprint or square footage) to ground results in known geographic contexts.

We find that this super-linearity depends on the amount of Segment Without Deceleration Points (*SWDP*). We coined that term to refer to a segment of one or more streets that contain no points that force stopping or deceleration. The size, distribution, and number of *SWDP* impact the super-linear exponent $\beta$, meaning that longer *SWDP* with good spatial coverage are easier to access and increase the gain of scale between the relation of time and distance.

The paper begins by explaining the data acquisition and the relation between time and distance used to estimating a city’s $\beta$ exponent. This is followed by detailing of the methodology such as explaining Deceleration Points (*DP*) and optimal routes simulations, providing a foundation for understanding the subsequent findings. The results section delves into the significance of *SWDPs* and *DPs* in achieving super-linear $\beta$ exponent, analyzing this relationship across cities worldwide. Lastly, the conclusion section reflects on how these findings align with sustainable development principles.

## Methods

This empirical research that analyzed cities and simulated optimal travel routes based on the street footprint, permissible speeds, and traffic flow patterns. Initially, the routes were extracted from the collaborative mapping tool *OpenStreetMap*. Information pertaining to street lights, street typology (motorway, primary, secondary, etc.), maximum street speed, and the length of street segments was obtained for 237 cities across different continents. The *osmnx* library was utilized to generate a graph from the *OpenStreetMap* data^28^. To represent cities, a directed graph *G*(*V*, *E*) was employed, with nodes *v* $(\in V)$ denoting street intersections and edges *e* $(\in E)$ representing directed street segments connecting them.

Subsequently, routes were simulated from every city nodes and exploring all possible directions until a specified time threshold was reached. The path taken was influenced by factors such as street typology, street maximum speed (obtained from *OpenStreetMap*), and the presence of *DPs* along the way. The calculation and adjustment of *DPs* were elaborated upon in detail. The analysis resulted in the determination of a $\beta$ exponent, representing the correlation between trip time and distance traveled along each optimal route from different origin points. Simulations were conducted for all 237 cities, taking into account the characteristics of the street network, including signage, speed limits, and intersections. Additionally, a subset of 10 cities was used to simulate travel routes considering traffic conditions.

### Calculating $\beta$ based on time and distance

**Figure 1:**
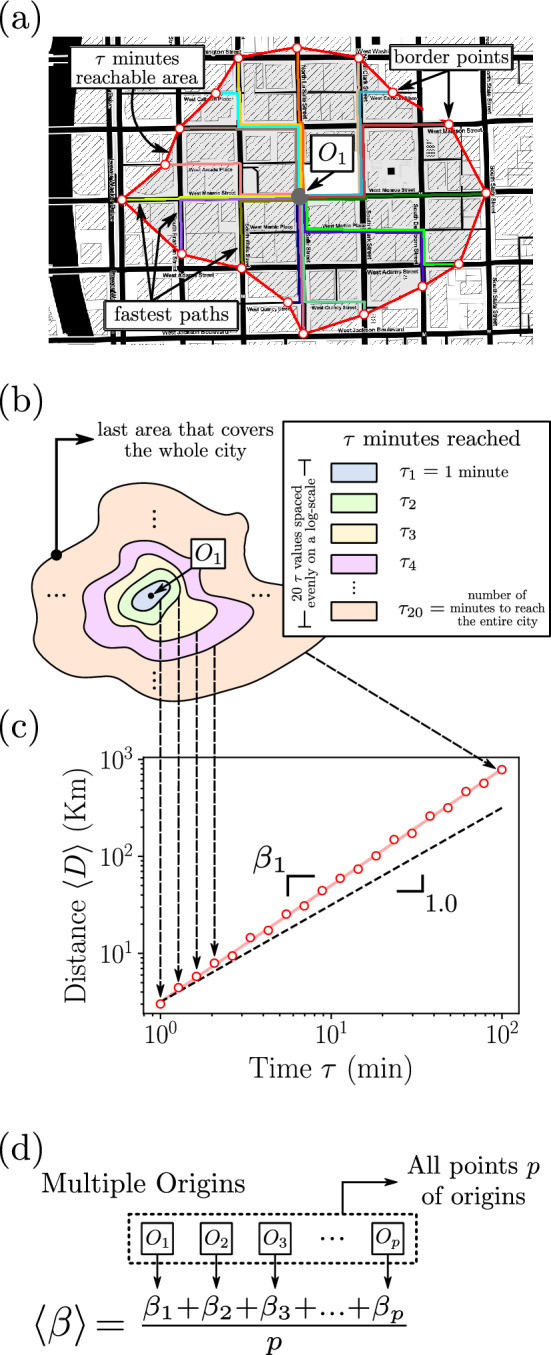
Method to estimate a city’s $\beta$ In (**a**) we show a city region where we simulated multiple optimal routes from all directions from a point of origin $O_1$. The routes are represented by different colors and take a shorter time than $\tau$. From those points we define an area that can be reached in $\tau$ minutes, represented by the area outlined in red. In (**b**), for a same origin $O_1$ we use multiple values of $\tau$. The legend in (**b**) indicates how we chose $\tau$ values. (**c**) shows how we establish a correlation between $\tau$ in the x-axis and the average distance reached $\langle D \rangle$ for the optimal fastest routes in the y-axis, both in logarithmic scale. Each point of this x-y relation is associated to an area of (**b**), where the value from the y-axis is defined as the average distance of the optimal routes within an area and the value from the x-axis is the $\tau$ used to reach that area. The solid red line shows the regression with a better fit between those points, with an inclination of $\beta _1$. The dashed line in black is a guideline, with exponent equal to 1.0. In (**d**) we define the calculus used to obtain a city’s $\beta$, which is the average of all $\beta _i$ values. Each $\beta _i$ is associated to a point of origin $O_i$ where the number of points *p* represents the total number of nodes in the city (cf. this figure(**d**)). This figure was generated using the open source drawing software *Inkscape* (v1.2) and the *Python* open source library *Matplotlib* (v3.7.1).

Figure 1a shows a point of origin $O_1$ chosen to simulate routes for a specific city region. We simulate all optimal (fastest) routes that can be traveled starting from $O_1$ during a specified time threshold, $\tau$, that represents the maximum allowable duration for a vehicle to reach its destination. The colored lines exemplifies routes that start from an origin point and reach several destination points. The border points show the furthest destinations reached to shape an area that represent the region which can be accessed starting from $O_1$ considering the time limit $\tau$. When connected, the border points form an isochrone area^12^. Such final points are not necessarily placed in city intersections and can also be points in the middle of a block since they must equate to places where an optimal route has weight equal to $\tau$. When an border point coordinate is not an intersection, we can conduct a linear interpolation in the node to find the edge coordinate.

The time needed to reach one node from a city’s network ($t_d$, where *d* is the node id) is calculated and stored from a single execution of the Dijkstra algorithm. Once we obtain the values of $t_d$ for a given origin and a value for $\tau$ we can find the border points. We generate 20 $\tau$ values for each city ($\tau _1, \tau _2, \tau _3,..., \tau _{20}$), where $\tau _1=1$ minute and $\tau _{20}=max(t_d)$ represent the amount of time it takes to get to a city’s boundary from an initial point. The other values for $\tau$’s ($\tau _2, \tau _3,..., \tau _{19}$) are generated to occupy a logarithmic scale. Each value for $\tau _j$ represents an area that can be covered in the city, starting from a shorter time until getting to an area that covers the entire city. Figure 1b presents the various isochrones generated for an origin point $O_1$ and with each of the 20 values for $\tau$.

We calculated the correlation between trip time and distance traveled for each optimal route originating from all nodes in the graph. Figure 1c shows the relation between time and distance for point $O_1$. Each point in the figure illustrates an isochrone area obtained from the varying $\tau$ values. The *x* axis represents the time for optimal routes, in minutes, which is also equal to the $\tau$ for that area, and the *y* axis represents the average distance of the optimal routes within the area, $\langle D \rangle$, in km, given a single unique distance value for each time limit. The axis are plotted in log-log scale. The correlation between $\tau$ and $\langle D \rangle$ helps to understand how routes with different lengths take place within a city.

The relation between time and distance is formally described by a Power Law^1^,

1 $$\begin{aligned} \langle D \rangle =a\tau ^\beta \end{aligned}$$

where $\tau$ is time, $\langle D \rangle$ quantifies the distance run, *a* is a pre-factor and $\beta$ is the exponent we want to measure.

The linear regression exponent between $log(\langle D \rangle )$ and $log(\tau )$ shows the efficiency of longer paths simulated within a city. If a correlation exponent is larger than 1, then a trip two times longer, for example, will reach an average distance more than twice as long, indicating a gain of scale.

In the example from Fig. 1c the $\beta _1$ exponent for the correlation between time and distance is associated to origin $O_1$, showing how $\beta$ gets calculated from a single origin. Figure 1d shows the the calculation of the exponent value $\beta$ for the entire city with multiple *p* origin points, is the average of all $\beta _i$, where $i=1,2,3,..,p$. In the next subsections, we will detail the processes presented in Fig. 1, including the explanation of the concepts of *DPs* and *SWDPs*, which are essential for the execution of the algorithm that simulates optimal routes.

### Defining deceleration points

A crucial factor that influences routes is the distribution of *DPs*, which are street nodes that can potentially cause stops or significant reductions in vehicle speed. We highlight that *DPs* are not static entities since their presence depends on the vehicle’s direction when passing a specific node. Consequently, identifying *DPs* in a city requires selecting a segment and direction. A particular intersection may serve as a *DP* for one route but not for another if the vehicle approaches from a different direction. These variations occur in each simulation of trips within a city, depending on street hierarchy. So due to the dynamic nature of *DPs*, all nodes are considered *DPs*, depending on the direction of travel of the vehicle.

**Figure 2:**
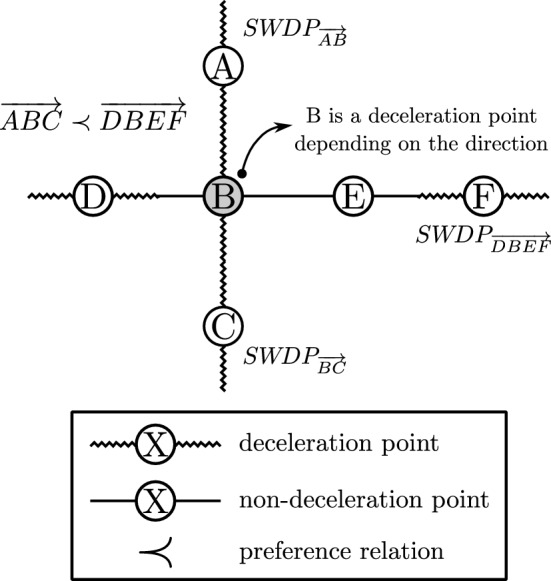
Identification of Deceleration Points and Segments Without Deceleration Points. The figure presents an example of how to identify a *DP*. In the cross-shaped diagram, the circles represent crossroads - street intersections, while the lines represent segments connecting these nodes. The relation of preference given to each street, shown by ($\prec$), indicates which nodes to classify as *DP*. This classification is dynamic and depends on the route the vehicle takes. For instance, the node B highlighted is classified as a *DP* if the vehicle takes a route moving in direction $\overrightarrow{ABC}$ while for the direction $\overrightarrow{DBEF}$ the node B is not a *DP*. This takes place because $\overrightarrow{ABC} \prec \overrightarrow{DBEF}$. The drawing style of the lines help illustrate when node is a *DP* (dashed lines) and not a *DP* (filled lines). Once the *DP* are established, the *SWDP* can be defined by joining the segments between points. The example presents three different *SWDP*: $SWDP_{\overrightarrow{AB}}$, $SWDP_{\overrightarrow{BC}}$ and $SWDP_{\overrightarrow{DBEF}}$. The first and last nodes of the *SWDP* will always be a *DP* and the intermediate nodes, if existent, will not be *DP*. This figure was generated using the open source drawing software *Inkscape* (v1.2).

The preferential relationship between intersecting streets is determined by the street typology obtained from *OpenStreetMap*: service $\prec$ residential $\prec$ tertiary $\prec$ secondary $\prec$ primary $\prec$ trunk $\prec$ motorway. Here, B $\prec$ A denotes that street segment A has priority over lane B, and if C $\prec$ B, then C $\prec$ A. Lanes of the same typology do not exhibit preferential differences; in such cases, A $=$ B. This phenomena is shown in Fig. 2.

If the route begins from points *A* or *C*, node *B* takes on a function of *DP*, whereas if the vehicle begins a trip from point *D* to *F*, it does not need to stop at *B* because the segment $\overrightarrow{AB}$ has lower preference than segment $\overrightarrow{DBEF}$, meaning that *B* does not characterize as a *DP*. It is also possible to define *SWDPs*, which are when the intermediate segments of one or more streets are not *DP*. Figure 2 shows three *SWDP*: the $SWDP_{\overrightarrow{DBEF}}$, which starts at *D* and ends at *F*, the nodes *B* and *E* which are not *DP*, and the segments $SWDP_{\overrightarrow{AB}}$ and $SWDP_{\overrightarrow{BC}}$ which present only a start and finish node.

**Figure 3:**
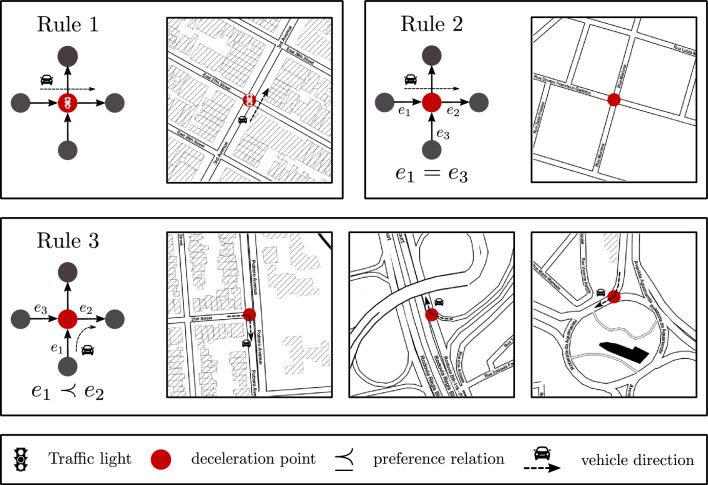
Rules to define Deceleration Points. Rule 1 states that any node with a traffic light is a *DP*. The other rules look at preferential streets, to define which streets have priority to allow for vehicle passage and are represented by “$\prec$” and “$=$” symbols. Rule 2 establishes that if a vehicle hits a node with a segment $e_1$ with equal priority among the incident segments (equal to $e_3$), then this node gets classified as *DP*, *i*.*e*. $e_1 = e_3$. Rule 3 states that a node will be *DP*, *i*.*e*. $e_1 \prec e_2$ if the car reaches a node coming from a segment with lower preference $e_1$ than the node used to continue $e_2$.The mapping figures present examples for how rules define *DP* when the vehicle approaches an avenue, roundabout or street junctions. This figure was generated using the open source drawing software *Inkscape* (v1.2) and the *Python* open source library *Folium* (v0.14.0).

In addition to the preference relation determined by street hierarchy, the distribution of *DPs* is also influenced by street signage, specifically traffic lights, and street topology, which can result in changes in vehicle direction. Figure 3 presents a simplified schematic depicting the rules associated with these additional elements.

### Simulating routes

With the defined rules and conditions for determining *DPs*, we proceed to simulate routes. The process begins by selecting random origin points and generating routes in every feasible direction, adhering to the street typology, permitted direction flows, and the presence of *DPs* along the way. The identification of *DPs* occurs simultaneously with the computation of time and distance for multiple optimal routes simulated from a graph. The trip continues until a specified time threshold is reached, and we estimate speeds based on the maximum speed data obtained from *OpenStreetMap*. Since *OpenStreetMap* does not inform the speed for every single segment and street, we employ a method to input missing speeds which is detailed in Supplementary Materials.

In the graph G, we conducted simulations of multiple routes originating from various points. The segments *e* within the graph are assigned a weight *w*(*e*), representing the average time required to traverse the segment. This time is calculated using the formula $w(e) = l(e) / V(e)$, where *l*(*e*) denotes the segment length and *V*(*e*) represents the maximum permitted speed on the street. Additionally, each node *v* is assigned a weight $w_v(v)$, reflecting the time a vehicle spends stopped at an intersection. Nodes classified as Deceleration Points (*DPs*) have $w_v(v) = 10$ seconds, while *non-DP* nodes have $w_v(v) = 0$. The Supplementary Material elaborates on the impact of varying $w_v$ on the results.

The sizes of the segments depend on a time threshold ($\tau$), which specifies the maximum allowable time for a vehicle to reach its destination. In other words, the sum of the weights of nodes and edges must not exceed $\tau$. To determine the optimal routes, we adapt the algorithm described in^29^.

The methodology employed to obtain the $\beta$ exponents for each city involves two algorithms, as described in the following pseudocodes. Algorithm 1 takes the graph of a specific city as input and calculates the corresponding $\beta$. Algorithm 1 is invoked by Algorithm 2 to identify border nodes, utilizing a list of routes and a time limit of $\tau$ as input. The calculation of border nodes is necessary for determining the precise location where a path should terminate, based on a specific value of $\tau$. So the border nodes provide a more accurate delimitation of isochronous areas.

**Algorithm 1:**
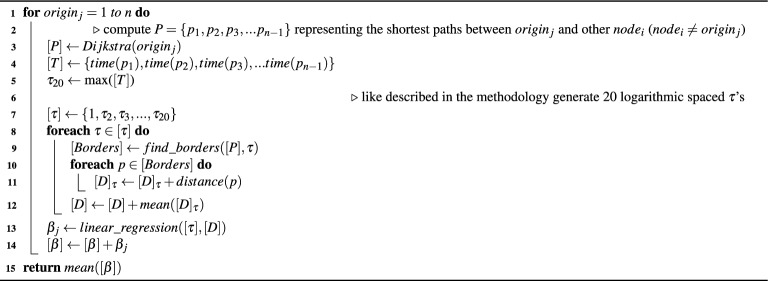
$$\beta$$ calculation(Graph $$G =[node_1, node_2, node_3, ...,node_n]$$).

Algorithm 1 has its main loop between lines 1 and 14 where we iterate over each city node and calculate their $\beta$. On line 3 it computes the shortest path from an origin to each other node on the graph (described by comment on line 2). Between lines 5 and 7 it selects the value for $\tau$ used as the time limit. It calculates the distance associated for each $\tau$ in lines 8 to 12. This main loop executes the Dijkstra algorithm for each origin of the graph *G*, in order to obtain an exponent $\beta _j$ for each origin. This procedure is performed to obtain a better estimate of the value of $\beta$ in the city as a whole and not just in a specific region. Algorithm 2 is called to compute what routes will be used as border. On line 13 it conducts a linear regression using $\tau$s and the average distances associated to them and store the exponent of that regression as a result. On line 15, the algorithm returns an average for the exponents calculated for each point.

**Algorithm 2:**
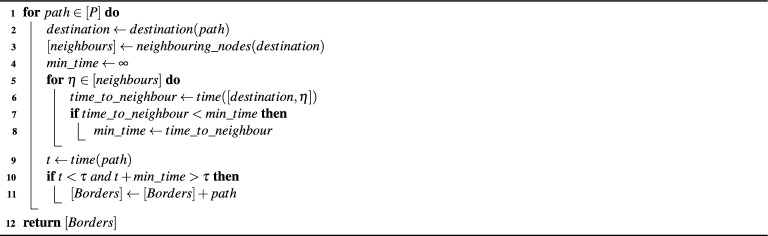
Find borders (Paths $[P] = [path_1, path_2, path_3,..., path_{n-1}]$, Time limit $\tau$).

The algorithm 2 finds the routes associated to a determined $\tau$, called border routes. It defines border routes as those where it is possible to reach a destination node within a time limit $\tau$, but where it is not possible to reach another further node considering the time. From lines 1 to 11 it computes the iteration on each route. From lines 2 to 8 it selects the shortest time obtained from a destination node, it iterates on every neighbor of the destination node (5 to 8) and select that one with shortest time. On line 10 we use $\tau$, the total route time *t* and the shortest time found previously to select whether a path is a border route. In case it is, line 11 adds that border route to a list of routes returned on line 12 at the end of the algorithm.

In Dijkstra^29^, the algorithm stops running when it finds the optimal path between two pre-defined points. In our adaptation, the algorithm runs a shortest path from a all origin point during a time limit $\tau$. Thus, there is no established final point and time is the limiting factor for the extent of the route as well as the nodes and segments weights.

### Calculations considering traffic

Subsequent experiments were conducted to explore a different scenario that incorporated traffic data to calculate the $\beta$ exponent. The objective was to demonstrate the possibility of obtaining similar super-linear results even when considering real-world traffic conditions. Nine cities were carefully for these simulations, aiming to encompass a wide range of $\beta$ values, equally distributed across continents.

The selected API, the *HERE Isoline Routing API v8*, was well-suited for our purposes, as it factors in traffic history when performing calculations. This made it an appropriate tool for investigating the influence of traffic on $\beta$ exponents. The API requires inputting the geographic coordinates of an origin point, a departure time, and a duration, and then generates polygons representing isochronous areas. Each edge of the polygon represents a destination reachable within the specified time parameters. For example, using *Here*, one can determine the maximum distance that can be traveled within twenty minutes when departing from a downtown location. We utilized the *Here* service to conduct simulations using a sample of origin points around 6 pm, a time period assumed to experience heavy traffic flow.

## Results

This section verifies the correlation between the time threshold $\tau$, and the average distances that are reachable $\langle D \rangle$ for the cities selected. As previously stated, this paper encompasses 237 cities from different continents and calculates a $\beta$ value for each. Table 1 details the non-linear relation between time and distance, measured by $\beta$ for all 237 cities, largely superior to 1.0. More statistical descriptions about the cities, including the number of nodes, edges, *SWDP*, the beta distribution, and the size distribution of *SWDP* segments, can be found in the Supplementary Material.

### How the spatial distribution of DPs and SWDPs impact $\langle \beta _s \rangle$ values

To comprehend the impact of spatial placement of *DPs* and *SWDPs* on the $\beta$ exponent, we conducted an experiment to investigate spatial autocorrelation. As conventional measures like Moran’s i index were unsuitable due to the dynamic nature of *DP* placement, we devised an alternative strategy. This involved randomly shuffling the values of $w_v(v)$ and *w*(*e*) and re-running the simulation for optimal routes in each city. Six mega-cities from diverse continents were chosen for this detailed analysis.

The shuffling process was performed for every node triplex, which consists of a route between three nodes, $A \rightarrow B \rightarrow C$. Node $A \rightarrow B$ represents the current street being traversed by the vehicle, while $B \rightarrow C$ indicates the desired street. Depending on the priority of streets $A \rightarrow B$ and $B \rightarrow C$, node B can be classified as a *DP* or not, following the rules outlined in Fig. 3. Once the triplexes are categorized as *DP* or *non-DP*, these classifications are randomized to eliminate the spatial correlation among *DPs* in the city. The time threshold $\tau$ and average distances $\langle D \rangle$ are then recalculated to determine a new exponent, denoted as $\langle \beta _s \rangle$, for the randomly configured *DPs*. A similar process is applied to randomly distribute street speeds, which consequently alters the node weights *w*(*e*). This process will not conserve the number of *SWDP*, since we want to study the natural emergence of these large-scale structures from the spatial correlations.

**Figure 4:**
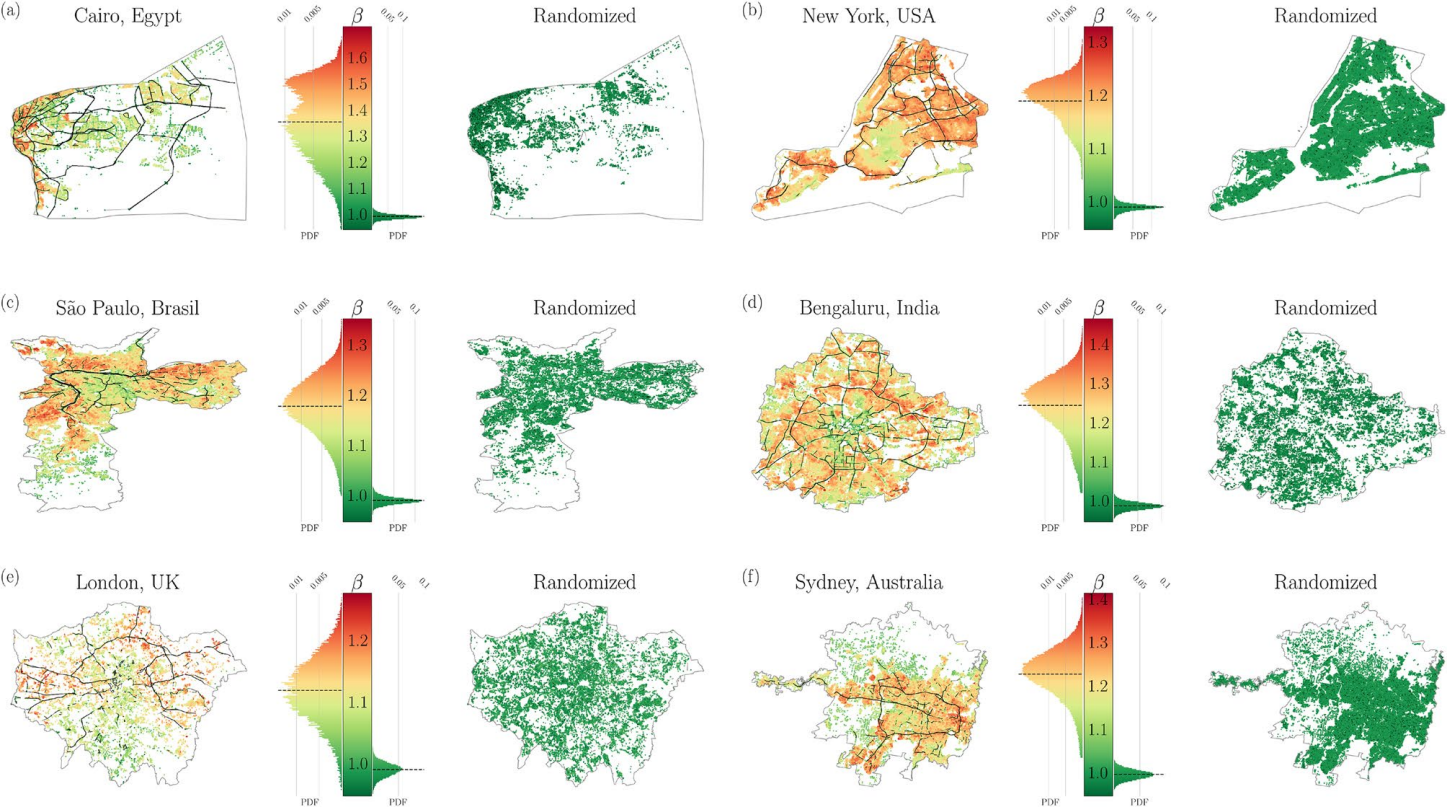
Spatial distribution of $\beta 's$ for different cities. In (**a**–**e**), we calculate the values for $\beta$ (on the left) and $\langle \beta _s \rangle$ (on the right) for each of those six cities. We take all the nodes from the street network as origin points. The points are colored according to their exponent value and their color is painted by the color scheme at the center. The black lines represent the larger *SWDP*. The function for probability density for each experiment’s $\langle \beta _s \rangle$ is shown in both sides of the color scale to represent each result from the execution, and a dashed line also shows its average distribution. This figure also shows the linearity of the $\langle \beta _s \rangle$ exponent for a random experiment, which stays around 1 for all cities. It also shows how *SWDPs* are important to ensure the non-linear characteristic of $\langle \beta _s \rangle$’s, indicating there is a spatial correlation between values with high exponents and *SWDP*. This figure was generated using the *Python* open source library *Matplotlib* (v3.7.1).

Figure 4 illustrates the spatial distribution of $\beta$ values for the six cities examined. To generate this visualization, we utilized all nodes within each city as origin points and implemented the proposed methodology to assign a specific $\beta$ value to each node, represented by its corresponding color. The experiment was conducted twice for each city. In the first run, the simulated routes took into account the city’s signaling system (displayed on the left side of the figure). In the second run, we employed the previously described shuffling process (shown on the right side of the figure).

The observations reveal two effects resulting from the shuffling: i) the disruption of free flow along continuous street segments, and ii) a more uniform distribution of $\beta$ exponents, with values closer to 1 in all cities. These effects are clearly discernible in the maps presented on the right side of the figure.

The results of the regression analysis for these cities demonstrate that the relationships between the shuffled *DPs* and street speeds vanish. This experiment highlights that the nonlinear association between $\tau$ and $\langle D \rangle$ arises as a consequence of the spatial correlation between the street network and *DPs*.

**Table 1:** Exponent for all cities simulated.

City	$\beta$	City	$\beta$	City	$\beta$
Jakarta, Indonesia	1.4 $\pm 0.07$	Cairo, Egypt	1.36 $\pm 0.02$	Seoul, South Korea	1.29 $\pm 0.03$
Amsterdam, Netherlands	1.29 $\pm 0.02$	Dubai, United Arab Emirates	1.29 $\pm 0.02$	Rio de Janeiro, Brasil	1.28 $\pm 0.03$
Casablanca, Morocco	1.28 $\pm 0.02$	Mumbai, India	1.28 $\pm 0.03$	Reykjavik, Iceland	1.27 $\pm 0.03$
Salvador, Brasil	1.26 $\pm 0.03$	Antwerp, Belgium	1.26 $\pm 0.03$	Ho Chi Minh City, Vietnam	1.25 $\pm 0.04$
Bengaluru, India	1.25 $\pm 0.02$	Guayaquil, Ecuador	1.24 $\pm 0.03$	Calgary, Canadá	1.24 $\pm 0.02$
Tripoli, Libya	1.24 $\pm 0.03$	Cologne, Germany	1.24 $\pm 0.02$	Kuala Lumpur, Malaysia	1.24 $\pm 0.03$
Singapore	1.24 $\pm 0.02$	Las Vegas, USA	1.24 $\pm 0.02$	Toronto, Canadá	1.24 $\pm 0.03$
Wollongong, Australia	1.24 $\pm 0.03$	San Diego, USA	1.23 $\pm 0.02$	São Bernardo do Campo, Brasil	1.23 $\pm 0.02$
Fes, Morocco	1.23 $\pm 0.02$	Sydney, Australia	1.23 $\pm 0.02$	Quebec City, Canadá	1.23 $\pm 0.03$
Adelaide, Australia	1.23 $\pm 0.02$	Ribeirão Preto, Brazil	1.23 $\pm 0.02$	Shenzhen, China	1.23 $\pm 0.02$
Ljubljana, Slovenia	1.22 $\pm 0.03$	Cork, Ireland	1.22 $\pm 0.03$	Playa del Carmen, Mexico	1.22 $\pm 0.02$
Lisboa, Portugal	1.22 $\pm 0.02$	Bergen, Vestland, Norway	1.22 $\pm 0.02$	Bern, Switzerland	1.21 $\pm 0.03$
Madrid, Spain	1.21 $\pm 0.02$	Conakry, Guinea	1.21 $\pm 0.02$	Tulsa, USA	1.21 $\pm 0.02$
Valparaíso, Chile	1.21 $\pm 0.03$	Mexico City, Mexico	1.21 $\pm 0.03$	Trondheim, Trøndelag, Norway	1.21 $\pm 0.02$
Prague, Czechia	1.2 $\pm 0.02$	Houston, USA	1.2 $\pm 0.02$	Edmonton, Canadá	1.2 $\pm 0.02$
Mérida, Yucatán, Mexico	1.2 $\pm 0.02$	Dallas, USA	1.2 $\pm 0.02$	Marrakesh. Morocco	1.2 $\pm 0.02$
Oakland, California, USA	1.2 $\pm 0.03$	Marrakesh, Morocco	1.2 $\pm 0.02$	Oklahoma City, USA	1.2 $\pm 0.02$
Chandigarh, India	1.2 $\pm 0.02$	Austin, USA	1.2 $\pm 0.02$	Campinas, Brasil	1.2 $\pm 0.03$
Utrecht, Netherlands	1.2 $\pm 0.02$	Porto, Portugal	1.19 $\pm 0.03$	Winchester, UK	1.19 $\pm 0.02$
Albuquerque, USA	1.19 $\pm 0.02$	Hanoi, Vietnam	1.19 $\pm 0.02$	Florianópolis, Brasil	1.19 $\pm 0.03$
Natal, Brasil	1.19 $\pm 0.03$	Cartagena, Colombia	1.19 $\pm 0.02$	São Luís, Brasil	1.19 $\pm 0.03$
Jacksonville, Florida, USA	1.19 $\pm 0.02$	The Hague, Netherlands	1.19 $\pm 0.02$	San Antonio, USA	1.19 $\pm 0.02$
Porto Velho, Brasil	1.19 $\pm 0.02$	Maputo, Mozambique	1.19 $\pm 0.03$	New York, USA	1.19 $\pm 0.02$
Valencia, Spain	1.18 $\pm 0.02$	Brisbane, Australia	1.18 $\pm 0.02$	Portland, USA	1.18 $\pm 0.02$
Vila Velha, Brasil	1.18 $\pm 0.03$	New Delhi, India	1.18 $\pm 0.02$	Columbus, USA	1.18 $\pm 0.02$
Los Angeles, USA	1.18 $\pm 0.02$	Caracas, Venezuela	1.18 $\pm 0.03$	São Paulo, Brasil	1.18 $\pm 0.02$
Taipei, Taiwan	1.18 $\pm 0.02$	Detroit, USA	1.18 $\pm 0.03$	Phoenix, USA	1.18 $\pm 0.02$
Bogotá, Colombia	1.17 $\pm 0.02$	Saint Louis, Illinois, USA	1.17 $\pm 0.02$	Louisville, USA	1.17 $\pm 0.02$
Brussels, Belgium	1.17 $\pm 0.02$	Vienna, Austria	1.17 $\pm 0.02$	Memphis, USA	1.17 $\pm 0.02$
Londrina, Brazil	1.17 $\pm 0.02$	Saskatoon, Canadá	1.17 $\pm 0.02$	Joinville, Brasil	1.17 $\pm 0.02$
Nuremberg, Germany	1.17 $\pm 0.02$	Nashville, USA	1.17 $\pm 0.02$	Hong Kong	1.17 $\pm 0.02$
Fortaleza, Brasil	1.17 $\pm 0.02$	Goiânia, Brasil	1.17 $\pm 0.02$	Campo Grande, Brasil	1.17 $\pm 0.01$
Genoa, Italy	1.16 $\pm 0.02$	Marseille, France	1.16 $\pm 0.03$	Curitiba, Brasil	1.16 $\pm 0.02$
Bamako, Mali	1.16 $\pm 0.02$	Boa Vista, Brasil	1.16 $\pm 0.02$	Niamey, Niger	1.16 $\pm 0.02$
Recife, Brasil	1.16 $\pm 0.02$	New Orleans, USA	1.16 $\pm 0.02$	Orlando, USA	1.16 $\pm 0.02$
Kyoto, Kyoto Prefecture, Japan	1.16 $\pm 0.01$	Buenos Aires, Argentina	1.16 $\pm 0.02$	Aracaju, Brasil	1.16 $\pm 0.02$
Kansas City,USA	1.16 $\pm 0.02$	Rabat, Morocco	1.15 $\pm 0.02$	Miami, USA	1.15 $\pm 0.02$
Paris, France	1.15 $\pm 0.02$	Nagpur, India	1.15 $\pm 0.02$	Baton Rouge, USA	1.15 $\pm 0.02$
Munich, Germany	1.15 $\pm 0.02$	Venice, Italy	1.15 $\pm 0.02$	Chicago, USA	1.15 $\pm 0.02$
Frankfurt, Germany	1.15 $\pm 0.02$	Rio Branco, Brasil	1.15 $\pm 0.01$	Ciudad del Este, Paraguay	1.15 $\pm 0.02$
Sapporo, Japan	1.15 $\pm 0.02$	Liverpool, UK	1.15 $\pm 0.02$	Manila, Philippines	1.14 $\pm 0.02$
Umea, Sweden	1.14 $\pm 0.02$	Belgrade, Serbia	1.14 $\pm 0.02$	Teresina, Brasil	1.14 $\pm 0.02$
Philadelphia, USA	1.14 $\pm 0.02$	João Pessoa, Brasil	1.14 $\pm 0.02$	Bucharest, Romania	1.14 $\pm 0.02$
Ulaanbaatar, Mongolia	1.14 $\pm 0.02$	Viña del Mar, Chile	1.14 $\pm 0.02$	Tirana, Albania	1.14 $\pm 0.03$
Manaus, Brasil	1.14 $\pm 0.02$	Barcelona, Spain	1.14 $\pm 0.02$	Guadalajara, Mexico	1.14 $\pm 0.02$
Nouakchott, Mauritania	1.13 $\pm 0.02$	Oslo,Norway	1.13 $\pm 0.03$	Nice, France	1.13 $\pm 0.02$
Charlotte, USA	1.13 $\pm 0.02$	Seattle, USA	1.13 $\pm 0.02$	Cali, Colombia	1.13 $\pm 0.02$

Honolulu, USA	1.13 $\pm 0.02$	Nairobi, Kenya	1.13 $\pm 0.02$	Budapest, Hungary	1.13 $\pm 0.02$
Joensuu, Finland	1.13 $\pm 0.02$	Montevideo, Uruguay	1.13 $\pm 0.02$	Maceió, Brasil	1.13 $\pm 0.02$
Accra, Ghana	1.13 $\pm 0.02$	Santo André, Brasil	1.12 $\pm 0.02$	Ostrava, Czechia	1.12 $\pm 0.02$
Raleigh, USA	1.12 $\pm 0.02$	Wuhan, China	1.12 $\pm 0.02$	Medellín, Colombia	1.12 $\pm 0.02$
Nur-Sultan, Kazakhstan	1.12 $\pm 0.02$	Asunción, Paraguay	1.12 $\pm 0.02$	Atlanta, USA	1.12 $\pm 0.02$
Beira, Mozambique	1.12 $\pm 0.02$	Denver,USA	1.12 $\pm 0.02$	Edinburgh, UK	1.12 $\pm 0.02$
Kinshasa, Democratic Republic of the Congo	1.12 $\pm 0.01$	London, UK	1.12 $\pm 0.02$	Montreal, Canadá	1.12 $\pm 0.02$
Manchester, UK	1.11 $\pm 0.02$	Cancun, Mexico	1.11 $\pm 0.04$	Dar es Salaam, Tanzania	1.11 $\pm 0.02$
Setúbal, Portugal	1.11 $\pm 0.02$	Hamilton, New Zealand	1.11 $\pm 0.02$	Santos, Brasil	1.11 $\pm 0.01$
Santa Fe, USA	1.11 $\pm 0.02$	Zagreb, Croatia	1.11 $\pm 0.02$	Pori, Finland	1.11 $\pm 0.02$
Porto Alegre, Brasil	1.11 $\pm 0.02$	Mombasa, Kenya	1.11 $\pm 0.02$	Palmas, Brasil	1.11 $\pm 0.01$
Sundsvall, Sweden	1.1 $\pm 0.03$	Oxford, UK	1.1 $\pm 0.02$	Turin, Italy	1.1 $\pm 0.02$
Nottingham, UK	1.1 $\pm 0.02$	Sofia, Bulgaria	1.1 $\pm 0.02$	Milwaukee, USA	1.1 $\pm 0.02$
Richmond, USA	1.1 $\pm 0.02$	Boulder, USA	1.09 $\pm 0.02$	Leipzig, Germany	1.09 $\pm 0.01$
Christchurch, New Zealand	1.09 $\pm 0.02$	Turku, Finland	1.09 $\pm 0.02$	Minneapolis, USA	1.09 $\pm 0.02$
Mar del Plata, Argentina	1.09 $\pm 0.02$	San Miguel de Allende, Mexico	1.09 $\pm 0.03$	Birmingham, UK	1.09 $\pm 0.01$
Timisoara, Romania	1.09 $\pm 0.02$	Dresden, Germany	1.09 $\pm 0.02$	Buffalo, New York, USA	1.09 $\pm 0.02$
Sarajevo, Bosnia and Herzegovina	1.08 $\pm 0.01$	Eugene, Oregon, USA	1.08 $\pm 0.02$	Rijeka, Croatia	1.08 $\pm 0.02$
Bristol, UK	1.08 $\pm 0.02$	Brazzaville, Congo-Brazzaville	1.08 $\pm 0.01$	Pittsburgh, Pennsylvania, USA	1.08 $\pm 0.02$
Kolkata, India	1.08 $\pm 0.02$	Nouméa, New Caledonia	1.07 $\pm 0.02$	Luanda, Angola	1.07 $\pm 0.02$
Baltimore, USA	1.07 $\pm 0.01$	Niteroi, Brasil	1.07 $\pm 0.03$	Boston, USA	1.06 $\pm 0.02$
Graz, Austria	1.06 $\pm 0.02$	Innsbruck, Austria	1.06 $\pm 0.02$	N’Djamena, Chad	1.06 $\pm 0.02$
Lyon, France	1.06 $\pm 0.01$	Milan, Italy	1.06 $\pm 0.01$	Copenhagen, Denmark	1.06 $\pm 0.01$
Akureyri, Iceland	1.05 $\pm 0.02$	Pyongyang, North Korea	1.05 $\pm 0.02$	Dublin, Ireland	1.05 $\pm 0.01$
Reading, USA	1.05 $\pm 0.01$	Salt Lake City, USA	1.05 $\pm 0.03$	Santiago, Chile	1.05 $\pm 0.01$
Yaoundé, Cameroon	1.04 $\pm 0.01$	Yantai, China	1.04 $\pm 0.01$	La Plata, Argentina	1.04 $\pm 0.01$
Port Harcourt, Nigeria	1.04 $\pm 0.01$	Kampala, Uganda	1.04 $\pm 0.02$	Nantes, France	1.04 $\pm 0.02$
Cuiabá, Brasil	1.03 $\pm 0.02$	Salzburg, Austria	1.03 $\pm 0.01$	Newark, USA	1.03 $\pm 0.02$
Cambridge, UK	1.03 $\pm 0.01$	Norwich, UK	1.03 $\pm 0.01$	Vancouver, Canadá	1.02 $\pm 0.01$
Papeete, France	1.02 $\pm 0.02$	Scranton, Pennsylvania, USA	1.02 $\pm 0.01$	Georgetown, Guyana	1.02 $\pm 0.02$
Jersey City, USA	1.01 $\pm 0.01$	Cayenne, France	1.01 $\pm 0.02$	Washington, USA	1.0 $\pm 0.01$
Geneva, Switzerland	0.99 $\pm 0.01$	Iquitos, Peru	0.99 $\pm 0.02$	Wilmington, USA	0.97 $\pm 0.01$

### The role of the large SWDPs

Figure 4 shows also the more extensive *SWDPs*, highlighted in black. In this paper we selected the 20% longest *SWDP* of each city. Next to the color bars we illustrate the density probability functions of the $\beta$ to the left and $\beta _s$ to the right. The results presented on the images to the left reveal the heterogeneity of the $\beta$ values, depending on the initial point where routes are simulated. Such heterogeneity is explained by the fact that our method simulates routes from every city node to estimate the $\beta$ for each city.

We notice there are origin points which produce routes with high $\beta$ around large *SWDP*. In New York, for instance, *Brooklyn* concentrates most of the smaller exponent values and practically no large *SWDP*, while that scenario is completely opposite for *Queens* and *Bronx*. Cairo is the city where such correlation can be the hardest to visualize since it is the only city where the distribution of $\beta$ values is multimodal. Still, it is possible to notice that hot spots concentrate to the left along with most of the large *SWDP*. In general, Fig. 4 shows that the large *SWDP* explain the non-linearity since they boost the increase in speed and allow for a gain of scale by reducing time for longer trips within cities.

**Figure 5:**
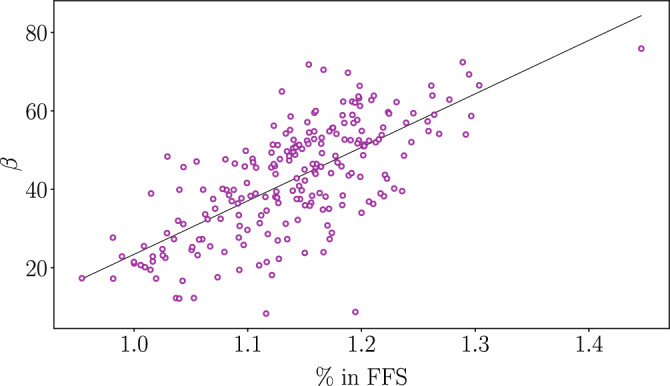
Correlation between $\delta ^{\%}_{SWDP}$ and $\beta$. Each point represents a city where $\delta ^{\%}_{SWDP}$, representing the percentage of the trips spent on free flow segments, is in the *x* axis and the exponent $\beta$ is in the *y* axis. The black line shows a linear regression that establishes a relation between both axis. This figure was generated using the *Python* open source library *Matplotlib* (v3.7.1).

Figure 5 shows another evidence of how *SWDP* are determining to the phenomena of non-linearity presented in this paper. We correlate the $\beta$ exponent with the percentage of trips completed in *SWDP* for each city ($\delta ^{\%}_{SWDP}$). This percentage illustrates how much gain of scale one can achieve with a route based on two factors. First, the greater the *SWDP*, the greater the gain of scale for a route in a trip. Second, the frequency with which trips use *SWDP* also increases the possibility of a gain of scale, and, in turn, an increase in $\beta$. Thus, we observe from Fig. 5 that the higher the density of $\delta ^{\%}_{SWDP}$, the more $\beta$ is exponentially higher for a specific city.

**Figure 6:**
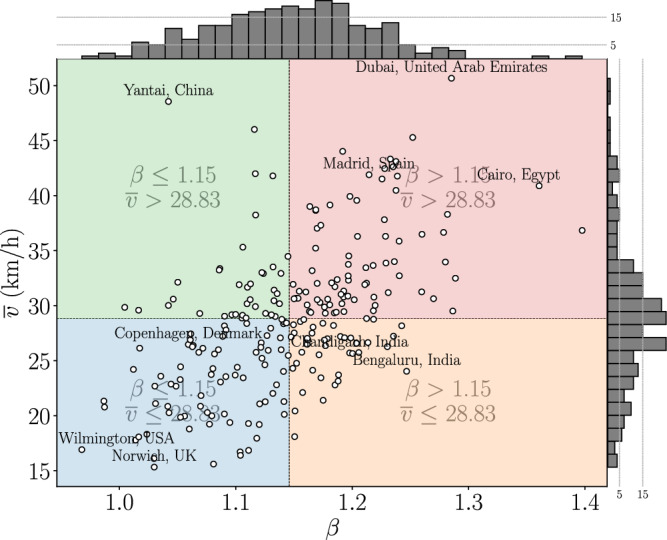
$\beta$ exponent versus average speed. Each point represents a city where, the *x* axis presents the average exponent ($\beta$) and the *y* axis presents the average speed $\overline{v}$ achieved for each trip simulated. The histograms for $\beta$ and $\overline{v}$ values are shown in the upper and right-hand corner, respectively. The guidelines separating the four colored quadrants are calculated based on their average $\langle \beta \rangle$ and $\overline{v}$ values. This figure was generated using the *Python* open source library *Matplotlib* (v3.7.1).

### How traffic affects the $\langle \beta _s \rangle$ exponent

Table 2 compares $\beta$ values obtained with and without traffic data from simulations run in the nine selected cities. In both cases, the simulations considered the same points of origin to allow for a more reliable comparison. Although these results are based on a small city subset, they show that the super-linear result remains constant in all cases. In fact, all cities turned out to have even greater $\beta$ in the scenarios that included traffic data. This indicates that traffic exacerbates the super-linear relation between time and distance since traffic tends to be more intense in areas without *SWDP*, which further lowers travel speed. Both scenarios are complementary since the street network sets the base for transit flow and traffic inherently operates within that fixed spatial structure.

**Table 2:** Comparing the $\beta$ exponent with and without traffic.

City	$\beta$ without traffic	$\beta$ with traffic
Amsterdam, Netherlands	1.29 ± 0.02	1.46 ± 0.02
Baltimore, USA	1.05 ± 0.01	1.36 ± 0.01
Barcelona, Spain	1.11 ± 0.02	1.46 ± 0.03
Christchurch, New Zealand	1.08 ± 0.01	1.31 ± 0.01
Dubai, United Arab Emirates	1.24 ± 0.02	1.34 ± 0.02
Fortaleza, Brazil	1.17 ± 0.01	1.23 ± 0.01
New Delhi, India	1.16 ± 0.01	1.17 ± 0.01
Oslo, Norway	1.13 ± 0.02	1.33 ± 0.01
Portland, USA	1.17 ± 0.02	1.40 ± 0.01

The traffic analysis is not exhaustive since it focuses on a non-representative sample, but it sets interesting and consistent results with findings in previous sections. Such analysis strengthens the notion that the street network and the distribution of *SWDP*, which remain constant, explain the super-linear exponent.

## Characterizing cities by average speed

Figure 6 depicts the analyzed cities as white dots distributed across four quadrants based on their $\overline{v}$ and $\beta$ values. We provide examples to illustrate the exponents, but our aim is not to establish causal relationships for cities’ placement in specific quadrants.

The first quadrant in blue is situated in the lower-left portion and encompasses cities with low average $\overline{v}$ and smaller gains of scale (lower $\beta$) for simulated trips. These cities are typically smaller in size, featuring a limited number of major arterial roadways and streets with lower maximum permitted speeds. Examples include Copenhagen, renowned as a model city in Yan Gehl’s “Cities for people”^30^, Boulder, recognized for its successful Urban Growth Boundary implementation, and London. Generally, these cities have street layouts and urban planning characterized by smaller segments, which hinder free flow and diminish the prominence of speed gains. Other cities within this quadrant include Miami and Detroit, both possessing relatively high street connectivity and density^31^, as well as Buffalo, celebrated for Frederick Law Olmsted’s planned green-way system and accessible streets^32^.

The upper-left quadrant in green features cities with lower scale efficiency but higher speeds for both long and short trips. These cities tend to have smaller built footprints, such as Frankfurt, La Plata, Vancouver, and Budapest. While these cities possess well-connected streets that facilitate higher speeds, they also exhibit larger city blocks and smaller total areas. For instance, Vancouver covers a modest 115 $km^{2}$ in comparison to Toronto (630 $km^{2}$) and Ottawa (2,778 $km^{2}$). La Plata, in Argentina, consists of a grid of six by six blocks intersected by diagonal streets, occupying only 27 $km^{2}$. Due to their limited extent, these cities do not allow for long *SWDP*, which accounts for their lower $\beta$ values.

The other two quadrants encompass cities with larger $\beta$, indicating that longer trips are completed proportionately faster than shorter ones. The lower-right quadrant, colored orange, includes cities with lower average speeds, such as Paris, New York, and Bogota. These larger cities are known for their extensive highway systems that traverse urban areas, enabling higher average speeds for longer distances. However, these cities are also constrained within dense urban environments. This constraint stems from measures taken to limit urban sprawl, either organically (e.g., NYC’s island location) or through legislation (refer to^33^ for the urban growth boundary in Portland). Consequently, smaller building blocks and a dense street network result in more *DP* points and can reduce overall speeds.

The red quadrant comprises cities with larger $\beta$ values but lower average speeds. This quadrant includes major metropolises like Rio de Janeiro, São Paulo, Los Angeles, Mexico City, Mumbai, and Seoul. These world-class cities have populations in the millions and have experienced substantial urban expansion. It is noteworthy that several of these cities, which achieve higher $\beta$s, are also associated with low sustainability and high greenhouse gas emissions resulting from on-road transportation^34^. Additional examples of such cities include Houston, known for its lack of zoning laws; Dubai, constructed with a focus on large freeways over the past two decades; and Shenzhen, a new city founded in the 1980s and notorious for its severe traffic conditions.

These examples highlight that regardless of attempts to control urban growth, the advantage of high speed for longer distances is evident in all cases. This is made possible by the utilization of highway networks with higher speeds and fewer deceleration points, which are commonly used for longer trips^35^. In summary, the presence of large-scale arterial roads with fewer *DPs* is associated with increased $\beta$ exponents.

Additionally, the previous examples demonstrate that $\beta$ values are not exclusive to planned or unplanned cities. The concept of planning refers to an urban settlement that has been carefully designed and constructed based on a predetermined blueprint or set of principles, which can vary according to different theoretical approaches guiding the development of street networks. For instance, a modernist city like Chandigarh (1.2) exhibits a higher $\beta$ than a New Urban town like Copenhagen (1.06), despite both being fundamentally planned cities.

In brief, these exponents alone are insufficient to categorize a city as “good” or “bad.” Contemporary planning principles, emphasizing sustainability, emphasize the importance of considering other indicators to conduct a comprehensive evaluation of the exponent. A lower $\beta$ value can be desirable if a city provides accessibility with a balanced distribution of services and opportunities. In such cases, citizens can enjoy a comfortable lifestyle with lower average speeds while making shorter trips. Conversely, a city with a low $\beta$ value due to the absence of connecting transportation infrastructure through *SWDPs* may face limitations in the transportation of goods and reduced urban efficiency. Therefore, while *SWDPs* play a significant role, they should be balanced with other factors that influence sustainable development.

## Conclusion

The objective of this paper was to investigate the relationship between travel time and distance traveled by examining the characteristics of the street network in 237 cities worldwide. The results revealed a non-linear correlation between time and distance for the simulated trips, indicating that longer trips within a city setting take proportionally less time than shorter trips. The $\beta$ exponent, which measures the power-law relationship between time and distance, indicated that cities with higher exponents enable proportionally faster trips as the required distance for those trips increases. In other words, if the required distance for a trip doubles in a city, the time spent on that trip increases by less than double. These super-linear results were consistently obtained across cities of various sizes and urban topologies. While we acknowledge that the Dijkstra algorithm may not accurately depict all real-world conditions, we have covered this limitation by simulating realistic traffic in nine different cities. This approach allowed us to capture a more realistic dynamic, and it’s worth noting that all observed results remain consistent.

The analysis demonstrated that the urban morphology and the street network of *SWDP* segments directly influences the magnitude of the $\beta$ exponent, resulting in longer trips exhibiting higher average speeds and shorter travel times when compared to shorter trips. Specifically, the spatial distribution of *DP* and street speeds contribute to form large *SWDPs*, which play a crucial role in forming the non-linear relationship between time and distance. This phenomenon can be understood by considering the natural growth patterns of cities, where residential and commercial zones typically exhibit a high density of *DP* and lower average speeds, connected by avenues with fewer *DP* and higher speeds. As cities grow, this street layout design facilitates the development of longer *SWDPs* in the form of highways.

This global study consistently yielded similar results across hundreds of cities, offering valuable insights into the universal principles governing urban dynamics. Such findings provide a robust foundation for developing effective strategies and policies that can be implemented across diverse urban contexts to address common challenges and promote sustainable development. From a practical standpoint, these findings can assist urban planners in evaluating how the construction of street networks worldwide influences driving behavior in terms of longer distances. On one hand, these networks are vital for improving travel efficiency, underscoring the continued importance of investing in highway high-speed networks to foster efficient and seamless mobility. On the other hand, the tendency to travel further is a natural and ubiquitous process, raising the question of how urban sprawl can be curtailed when traveling longer distances offers advantages in terms of speed. While enhancing transportation speed is crucial for urban efficiency, it must be balanced with sustainability principles, such as containing urban sprawl^36^. Moving forward, planners must carefully examine how these road segments are integrated into the urban fabric to prevent the fragmentation of neighborhoods and mitigate adverse effects such as increased pollution and decreased walkability.

The results can lead us to speculate regarding a potential connection between the spatial distribution of $\beta$ and accessibility by car. Though our study does not delve deeply into this aspect, it opens a promising avenue for future research. Understanding this connection may require a comprehensive examination of multiple factors, such as the influence of the pre-factor *a* in Eq. (1). We hope that our findings may serve as a stepping stone for these future investigations.

### Supplementary Information

Supplementary Information.

## Data Availability

The data that support the findings of this study are available from the open and collaborative mapping tool *OpenStreetMap*. We used the Python’s library *osmnx* to generate the graph based on *OpenStreetMap* data^28^.
