# Supplementary material for: A global empirical study on how street networks facilitate driving longer distances
Source: Sci Rep. 2023 Oct 24;13:18154. doi: 10.1038/s41598-023-45236-7 (PMC10598139; doi:10.1038/s41598-023-45236-7)
Supplement: Supplementary file 1 — Supplementary Information. [file 41598_2023_45236_MOESM1_ESM.pdf]

Supplementary Material

A Global Empirical Study on How Street Networks Facilitate Driving Longer Distances

1. OBTAINING MISSING MAXIMUM SPEEDS

Since some cities did not have maximum street speeds $V(e)$ filled out by OSM, we developed a method to specifically input the missing values. We used an attribute from OSM that classifies the type of street as *highway*. For each value of *highway*, we calculate the average *maxspeed* of all edges with reported maximum speed values to obtain an average maximum speed associated to each street type. We then assign those average values to streets segments of the same type with missing values.

In order to evaluate how those filled out values influenced the estimated exponent, we calculated the percent of initial *maxspeed* filled out for every city analyzed in the paper, defined by $V(e)_{\%}$. For example, a city with $V(e)_{\%} = 50\%$ has initially half of its streets with assigned speed values. We selected every city with $V(e)_{\%} > 80\%$ and randomly lowered that percentage to analyze the effect of different $V(e)_{\%}$ in the original exponent. Figure S1 presents an example on the results obtained where,in (a) as the $V(e)_{\%}$ becomes smaller the exponent either varies within the margin of error or becomes a bit smaller, which indicates the method is conservative. Out of the 32 cities with $V(e)_{\%} > 80\%$, 8 stayed within the margin of error, like the case of Norwich, in (b). Other 21 cities followed a similar pattern as Dublin, in (a) where the exponent decreases with very small filled out speeds. The exponent varied greatly outside the margin of error in only 3 cities such as the case of Buenos Aires, in (c).

In general, when applied to cities with incomplete $V(e)_{\%}$, in 90% of the cases, the method applied to artificially fill out missing speeds does not significantly alter the exponent, which either remains within the average margin of error or becomes smaller. Thus, the super-linear effect we obtained in this paper was not caused by the process of filling out missing values for *maxspeed*.

2. CHOICE OF THE DECELERATION TIMES AT INTERSECTIONS (W_V)

This section details how the deceleration times at intersections w_v had an effect on the β exponent. The time w_v is equivalent to the weight, in seconds, associated to a node (v). When such node is a DP, we increase the value of w_v to represent the longer time it takes to cross a specific intersection.

We calculated the variable w_v for Cairo, New York, São Paulo, Bengaluru, London and Sydney. For each of these cities, we chose 100 random points and assigned them a w_v that varied between zero (no deceleration) and sixty seconds to record how various DPs would have an effect on the simulations.

Figure S2 shows the relation between w_v and β values for each city. For most cities, We observe a considerable increase in β values for the first 20 seconds, which is apparently followed by a plateau with no sudden change. In no moment at all were the β values linear, which was expected since the DPs originate both the exponent and the spatial distribution of a city's street speeds.

3. CITY METHODOLOGICAL FILE

We produced a methodological file for each city analyzed on the paper, which contains the most relevant and novel information generated during the research. In general, each file presents where each city falls within the quadrants that classify average speed by the exponent, a map of the exponents and the longest $SWDP$ shown by Figure 5 of the original paper, the relation between time τ and average distance reached $\langle D \rangle$, a histogram for the $SWDP$ lengths and other information such as $\langle \beta \rangle$ and number of nodes and edges.

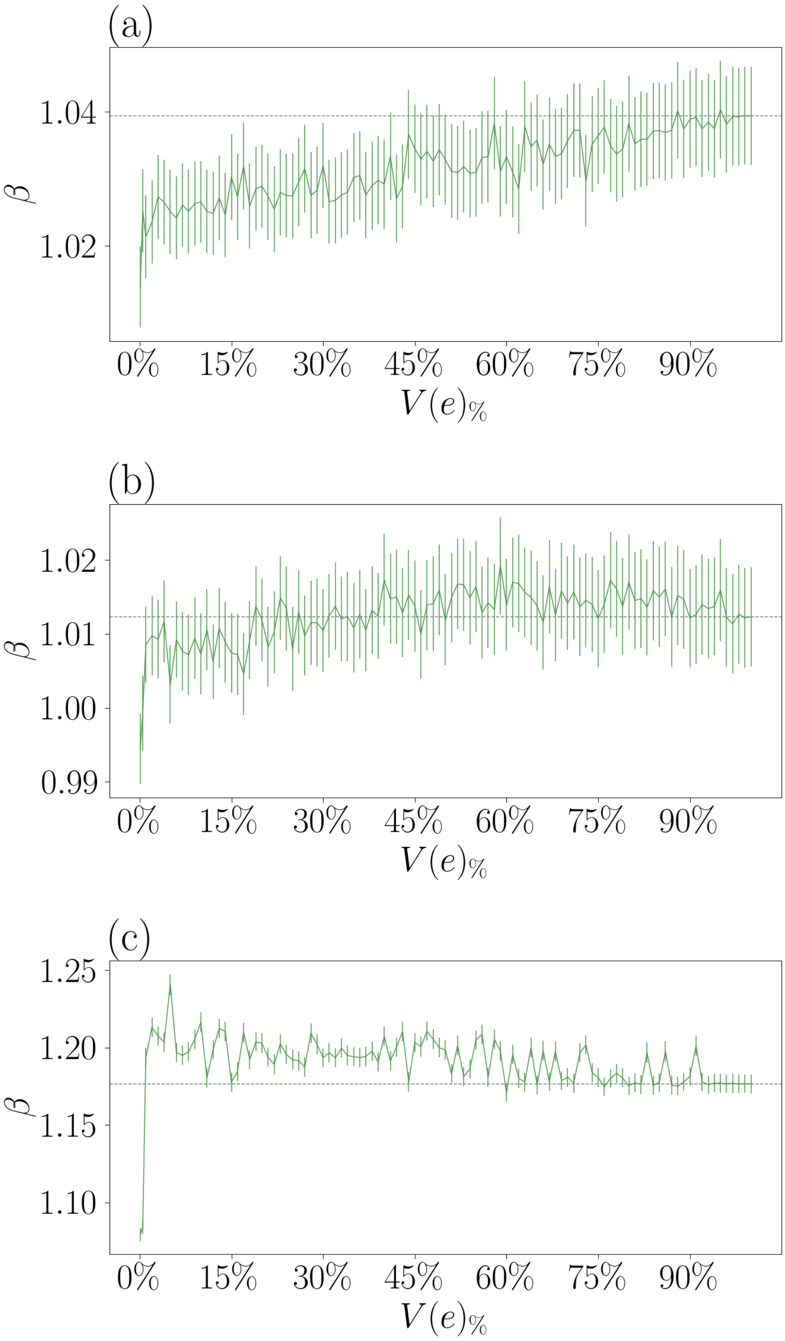

Fig. S1. Analysis of the variation of $V(e)_{\%}$. In all the plots, the x-axis presents the percentage of *maxspeed* initially filled out for all edges of a the city graph and the y-axis presents the exponent obtained when executing the methodology to fill out missing speeds for one hundred random points. The dashed horizontal line represents the β we obtained from the city's original speeds (with missing speeds). We also plotted the standard errors for the calculated average maximum speeds, represented by the vertical lines along the main plot line. In (a) the plot is for the city of Dublin, Ireland; (b) for the city of Norwich, UK; (c) for the city of Buenos Aires, Argentina.

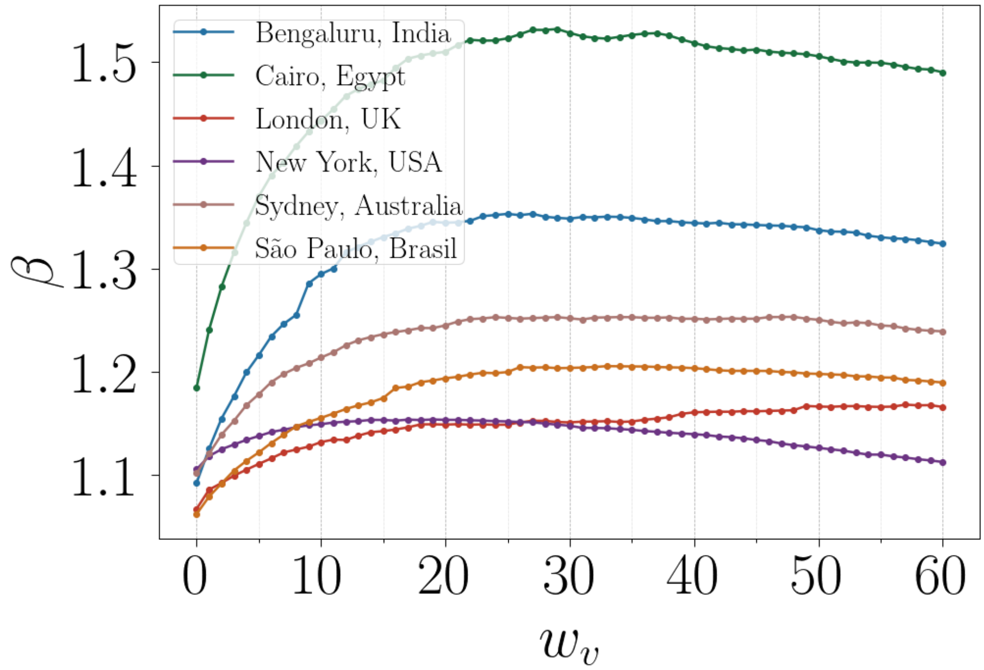

Fig. S2. β **exponent by** w_v. Each line represents a city where, the x axis shows the average (β) and the y axis shows the impact of the time w_v on a deceleration point. The cities selected are the same mentioned previously, also detailed in the figure caption.

Accra, Ghana

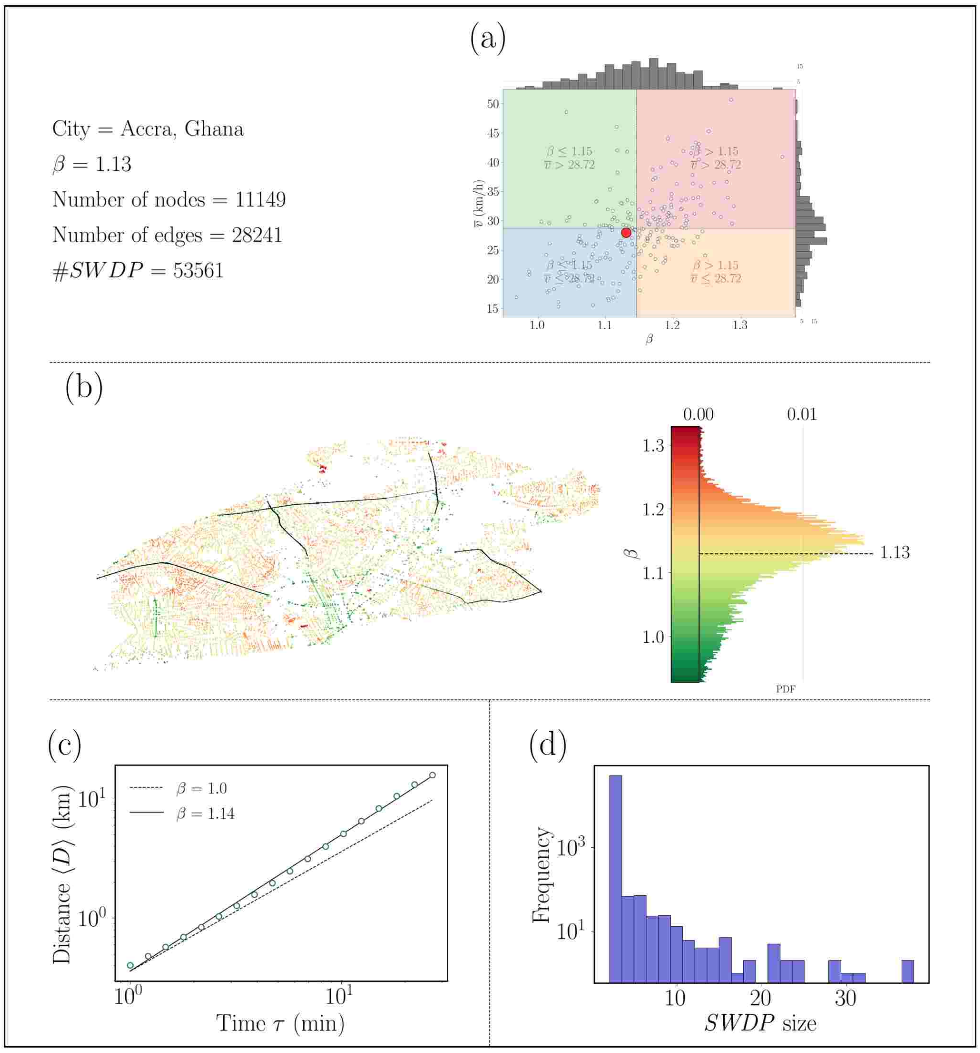

Fig. S3. Methodological sheet - Accra, Ghana. In (a) Each point represents a city, with mean exponent (β), on the x-axis, and mean speed $\overline{v}$ obtained in all trips made to calculate the exponent on the axis y. The histograms of the values of β and $\overline{v}$ are shown on the axes in the upper and right corners, respectively. The graph was segmented into four quadrants, in which the division is performed by the mean values of β and $\overline{v}$. The quadrants were colored and annotated according to the division criteria. The red dot represents the location of Accra, Ghana. In (b) taking all the nodes of Accra, Ghana as origin, the dots are colored as a function of their exponent value and their color is quantified by the color bar in the center. The longest segments without a deceleration point (SWDP) are plotted in black. The probability density function of the $\beta's$ for each experiment is shown on the left of the color scale Figure (c) shows the mean correlation curve between time τ and the distance $\langle D \rangle$. The black traced line represents the exponent equal to 1.0. Figure (d) shows the distribution of SWDP sizes in number of nodes per frequency of occurrence.

Adelaide, Australia

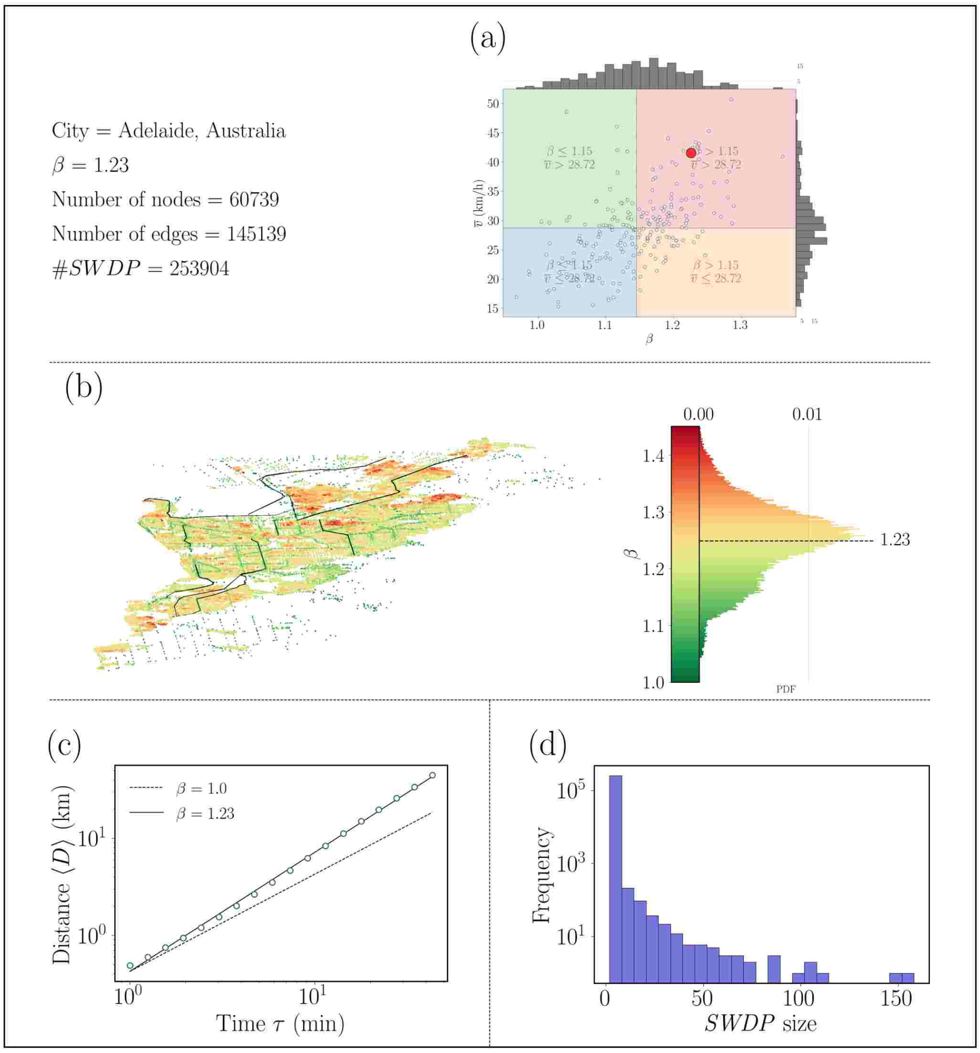

Fig. S4. Methodological sheet - Adelaide, Australia. In (a) Each point represents a city, with mean exponent (β), on the x-axis, and mean speed $\overline{v}$ obtained in all trips made to calculate the exponent on the axis y. The histograms of the values of β and $\overline{v}$ are shown on the axes in the upper and right corners, respectively. The graph was segmented into four quadrants, in which the division is performed by the mean values of β and $\overline{v}$. The quadrants were colored and annotated according to the division criteria. The red dot represents the location of Adelaide, Australia. In (b) taking all the nodes of Adelaide, Australia as origin, the dots are colored as a function of their exponent value and their color is quantified by the color bar in the center. The longest segments without a deceleration point (SWDP) are plotted in black. The probability density function of the $\beta's$ for each experiment is shown on the left of the color scale Figure (c) shows the mean correlation curve between time τ and the distance $\langle D \rangle$. The black traced line represents the exponent equal to 1.0. Figure (d) shows the distribution of SWDP sizes in number of nodes per frequency of occurrence.

Akureyri, Iceland

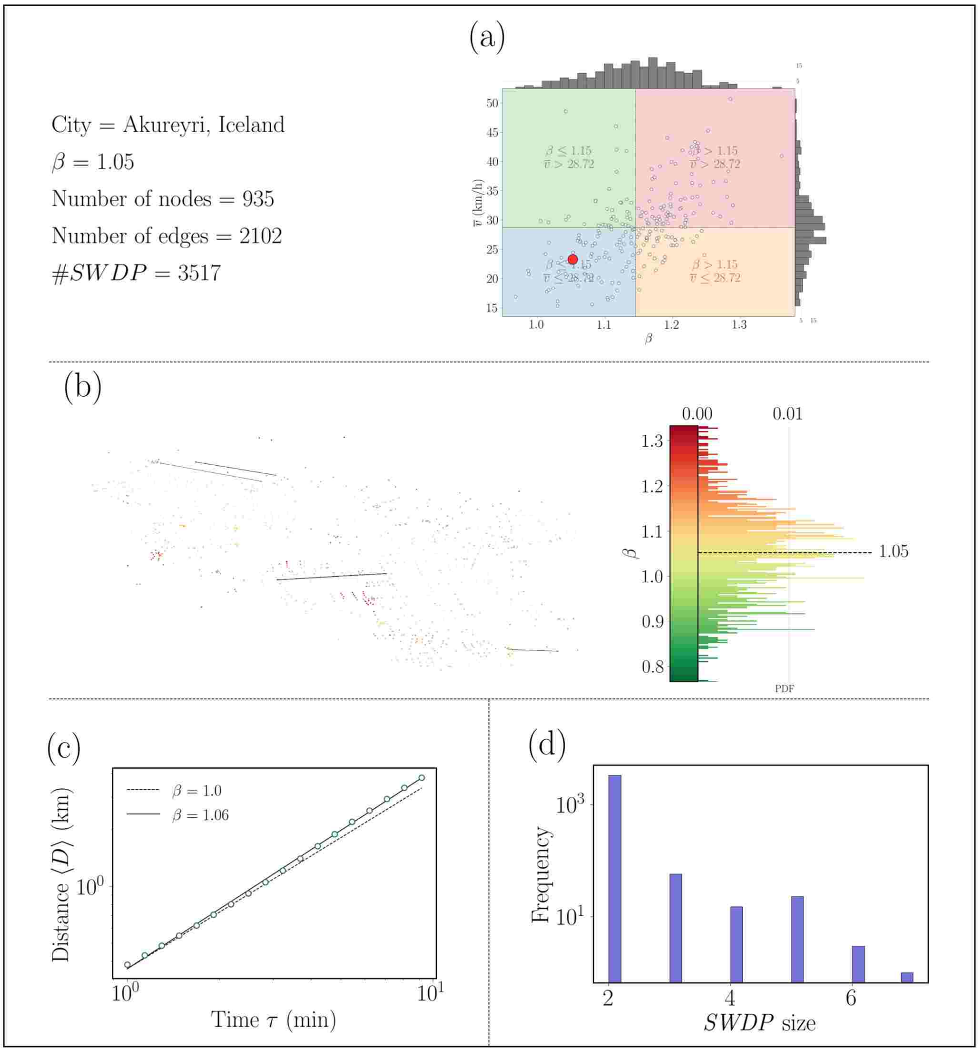

Fig. S5. Methodological sheet - Akureyri, Iceland. In (a) Each point represents a city, with mean exponent (β), on the x-axis, and mean speed $\overline{v}$ obtained in all trips made to calculate the exponent on the axis y. The histograms of the values of β and $\overline{v}$ are shown on the axes in the upper and right corners, respectively. The graph was segmented into four quadrants, in which the division is performed by the mean values of β and $\overline{v}$. The quadrants were colored and annotated according to the division criteria. The red dot represents the location of Akureyri, Iceland. In (b) taking all the nodes of Akureyri, Iceland as origin, the dots are colored as a function of their exponent value and their color is quantified by the color bar in the center. The longest segments without a deceleration point (SWDP) are plotted in black. The probability density function of the $\beta's$ for each experiment is shown on the left of the color scale Figure (c) shows the mean correlation curve between time τ and the distance $\langle D \rangle$. The black traced line represents the exponent equal to 1.0. Figure (d) shows the distribution of SWDP sizes in number of nodes per frequency of occurrence.

Albuquerque, USA

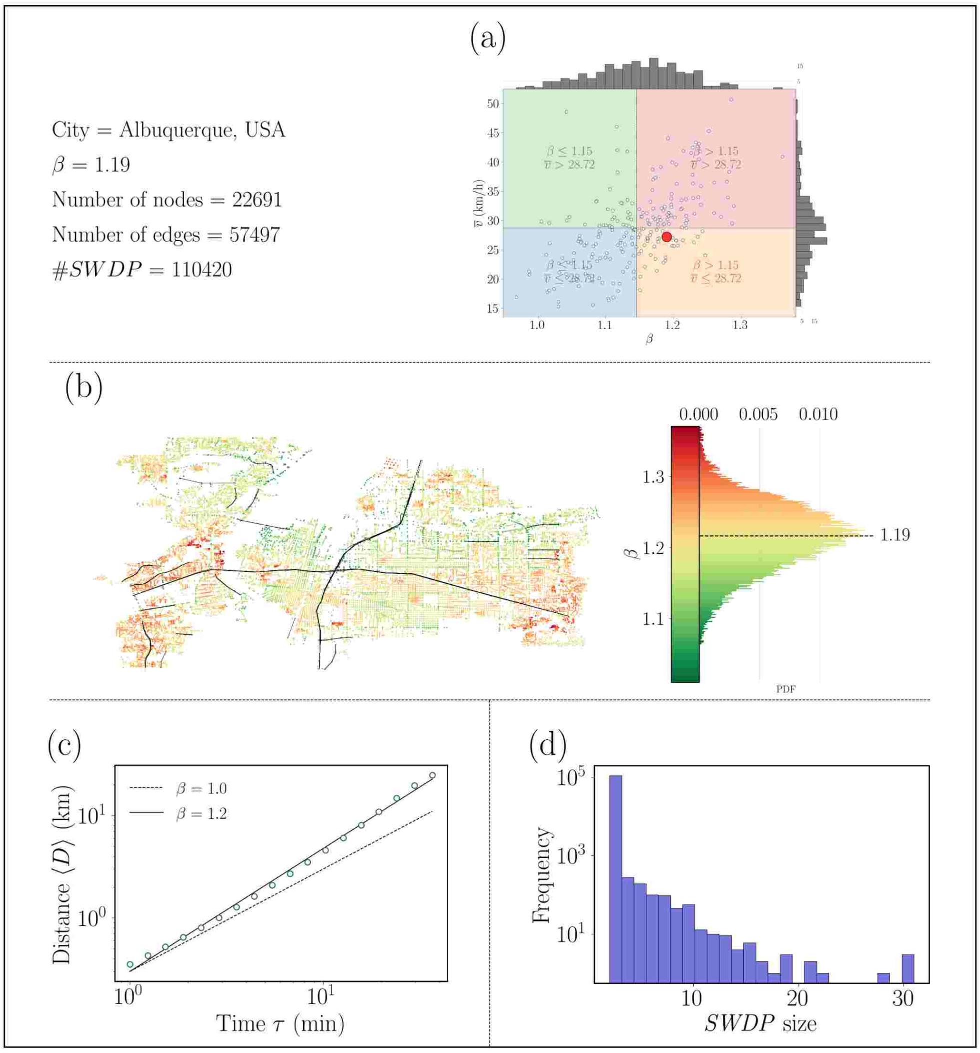

Fig. S6. Methodological sheet - Albuquerque, USA. In (a) Each point represents a city, with mean exponent (β), on the x-axis, and mean speed $\overline{v}$ obtained in all trips made to calculate the exponent on the axis y. The histograms of the values of β and $\overline{v}$ are shown on the axes in the upper and right corners, respectively. The graph was segmented into four quadrants, in which the division is performed by the mean values of β and $\overline{v}$. The quadrants were colored and annotated according to the division criteria. The red dot represents the location of Albuquerque, USA. In (b) taking all the nodes of Albuquerque, USA as origin, the dots are colored as a function of their exponent value and their color is quantified by the color bar in the center. The longest segments without a deceleration point (SWDP) are plotted in black. The probability density function of the $\beta's$ for each experiment is shown on the left of the color scale Figure (c) shows the mean correlation curve between time τ and the distance $\langle D\rangle$. The black traced line represents the exponent equal to 1.0. Figure (d) shows the distribution of SWDP sizes in number of nodes per frequency of occurrence.

Amsterdam, Netherlands

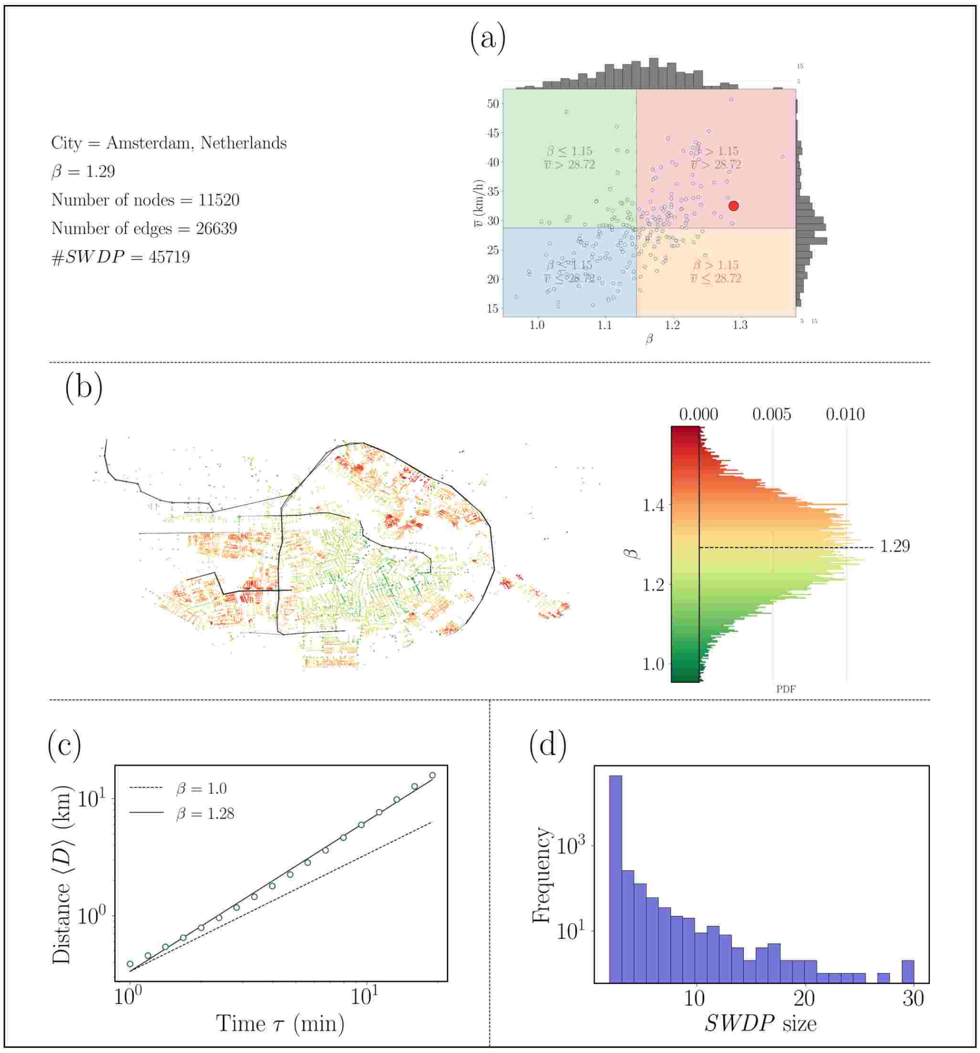

Fig. S7. Methodological sheet - Amsterdam, Netherlands. In (a) Each point represents a city, with mean exponent (β), on the x-axis, and mean speed $\overline{v}$ obtained in all trips made to calculate the exponent on the axis y. The histograms of the values of β and $\overline{v}$ are shown on the axes in the upper and right corners, respectively. The graph was segmented into four quadrants, in which the division is performed by the mean values of β and $\overline{v}$. The quadrants were colored and annotated according to the division criteria. The red dot represents the location of Amsterdam, Netherlands. In (b) taking all the nodes of Amsterdam, Netherlands as origin, the dots are colored as a function of their exponent value and their color is quantified by the color bar in the center. The longest segments without a deceleration point (SWDP) are plotted in black. The probability density function of the $\beta's$ for each experiment is shown on the left of the color scale Figure (c) shows the mean correlation curve between time τ and the distance $\langle D \rangle$. The black traced line represents the exponent equal to 1.0. Figure (d) shows the distribution of SWDP sizes in number of nodes per frequency of occurrence.

Antwerp, Belgium

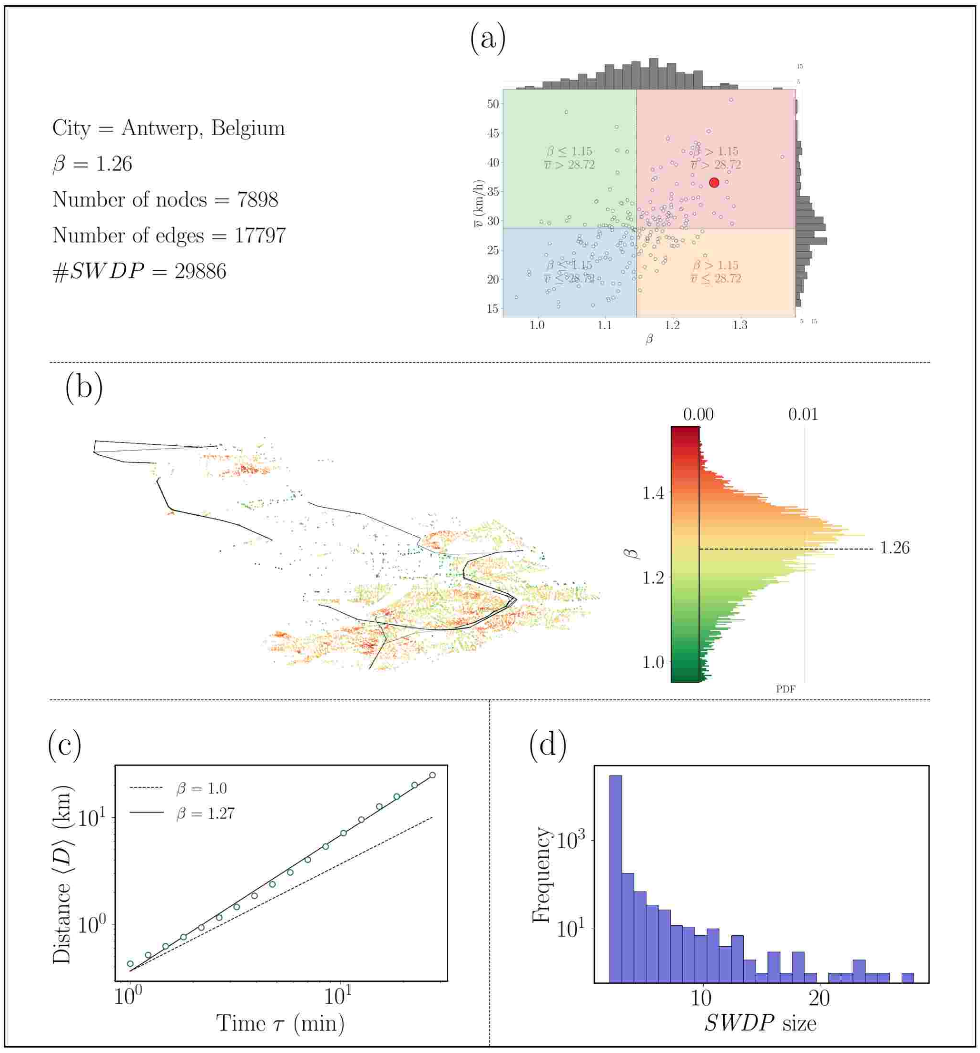

Fig. S8. Methodological sheet - Antwerp, Belgium. In (a) Each point represents a city, with mean exponent (β), on the x-axis, and mean speed $\overline{v}$ obtained in all trips made to calculate the exponent on the axis y. The histograms of the values of β and $\overline{v}$ are shown on the axes in the upper and right corners, respectively. The graph was segmented into four quadrants, in which the division is performed by the mean values of β and $\overline{v}$. The quadrants were colored and annotated according to the division criteria. The red dot represents the location of Antwerp, Belgium. In (b) taking all the nodes of Antwerp, Belgium as origin, the dots are colored as a function of their exponent value and their color is quantified by the color bar in the center. The longest segments without a deceleration point (SWDP) are plotted in black. The probability density function of the $\beta's$ for each experiment is shown on the left of the color scale Figure (c) shows the mean correlation curve between time τ and the distance $\langle D \rangle$. The black traced line represents the exponent equal to 1.0. Figure (d) shows the distribution of SWDP sizes in number of nodes per frequency of occurrence.

Asunción, Paraguay

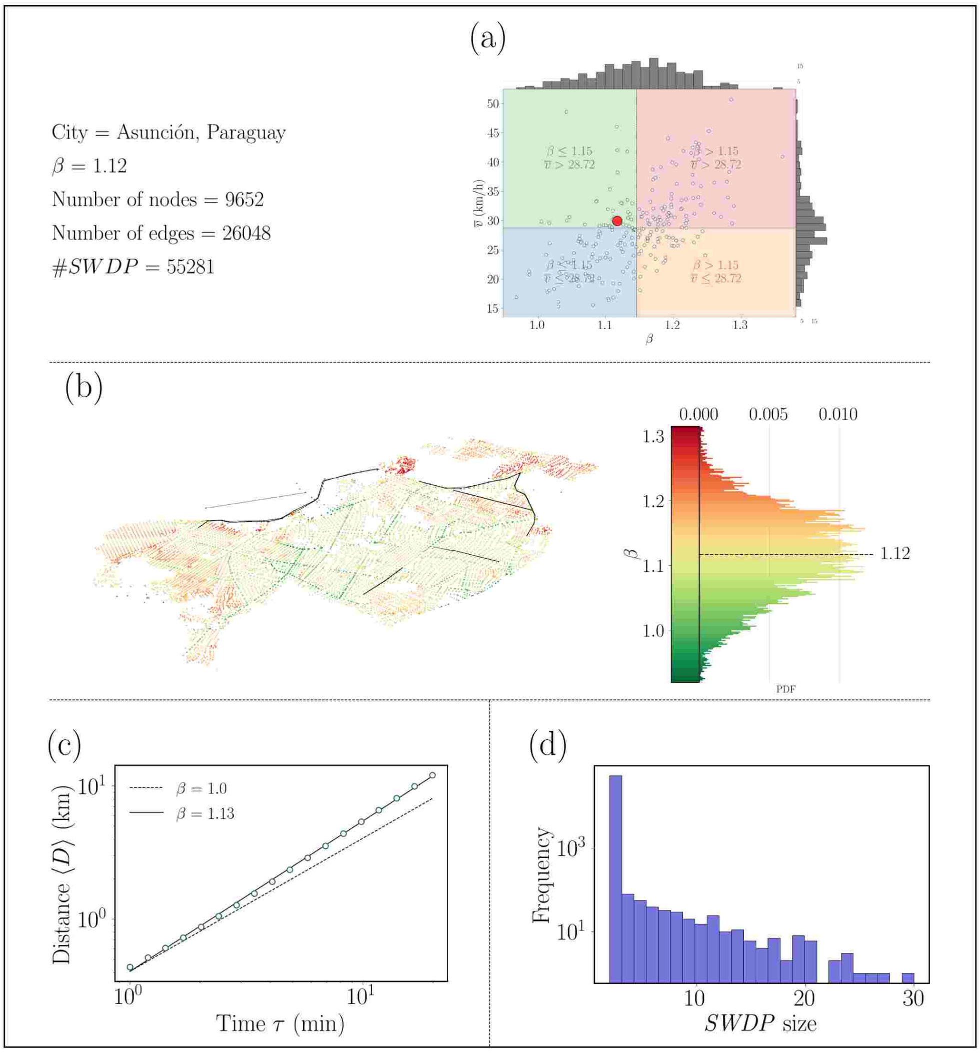

Fig. S9. Methodological sheet - Asunción, Paraguay. In (a) Each point represents a city, with mean exponent (β), on the x-axis, and mean speed $\overline{v}$ obtained in all trips made to calculate the exponent on the axis y. The histograms of the values of β and $\overline{v}$ are shown on the axes in the upper and right corners, respectively. The graph was segmented into four quadrants, in which the division is performed by the mean values of β and $\overline{v}$. The quadrants were colored and annotated according to the division criteria. The red dot represents the location of Asunción, Paraguay. In (b) taking all the nodes of Asunción, Paraguay as origin, the dots are colored as a function of their exponent value and their color is quantified by the color bar in the center. The longest segments without a deceleration point (SWDP) are plotted in black. The probability density function of the $\beta's$ for each experiment is shown on the left of the color scale Figure (c) shows the mean correlation curve between time τ and the distance $\langle D \rangle$. The black traced line represents the exponent equal to 1.0. Figure (d) shows the distribution of SWDP sizes in number of nodes per frequency of occurrence.

Atlanta, USA

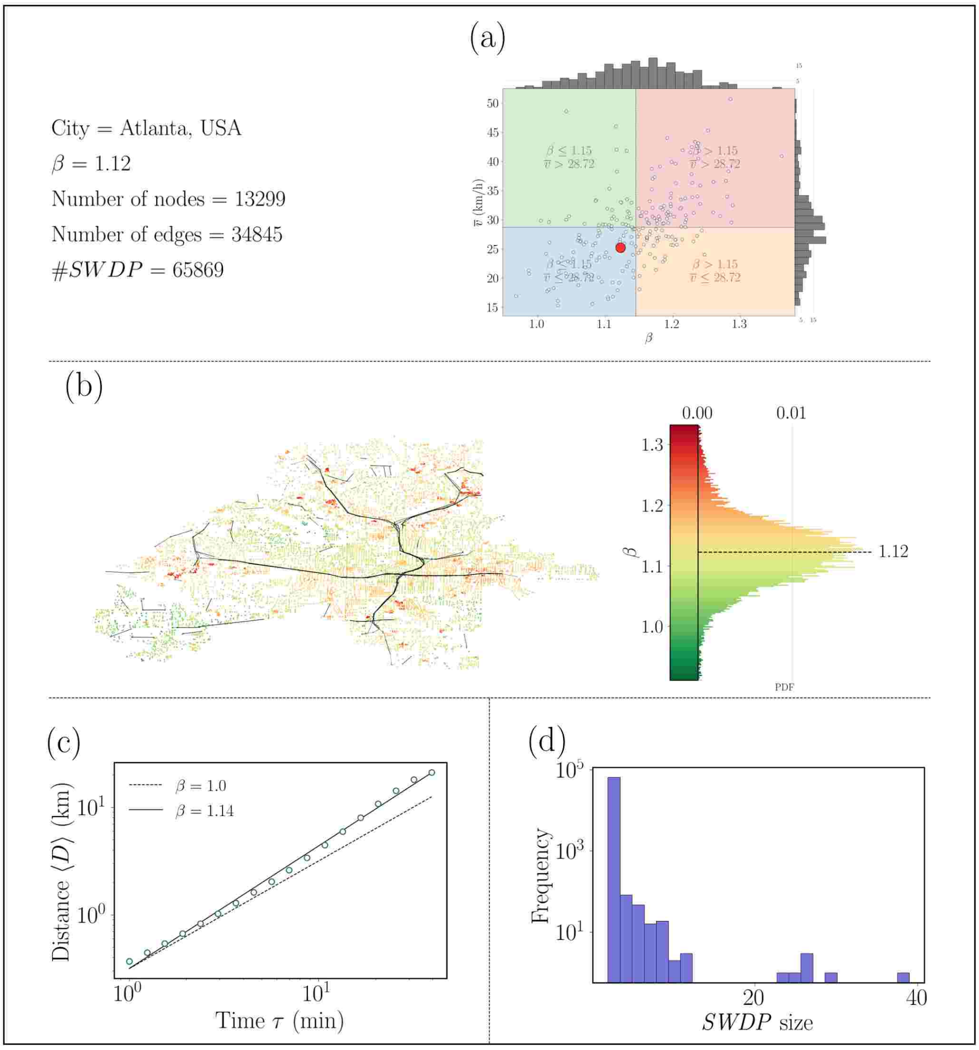

Fig. S10. Methodological sheet - Atlanta, USA. In (a) Each point represents a city, with mean exponent (β), on the x-axis, and mean speed $\overline{v}$ obtained in all trips made to calculate the exponent on the axis y. The histograms of the values of β and $\overline{v}$ are shown on the axes in the upper and right corners, respectively. The graph was segmented into four quadrants, in which the division is performed by the mean values of β and $\overline{v}$. The quadrants were colored and annotated according to the division criteria. The red dot represents the location of Atlanta, USA. In (b) taking all the nodes of Atlanta, USA as origin, the dots are colored as a function of their exponent value and their color is quantified by the color bar in the center. The longest segments without a deceleration point (SWDP) are plotted in black. The probability density function of the $\beta' s$ for each experiment is shown on the left of the color scale Figure (c) shows the mean correlation curve between time τ and the distance $\langle D \rangle$. The black traced line represents the exponent equal to 1.0. Figure (d) shows the distribution of SWDP sizes in number of nodes per frequency of occurrence.

Austin, USA

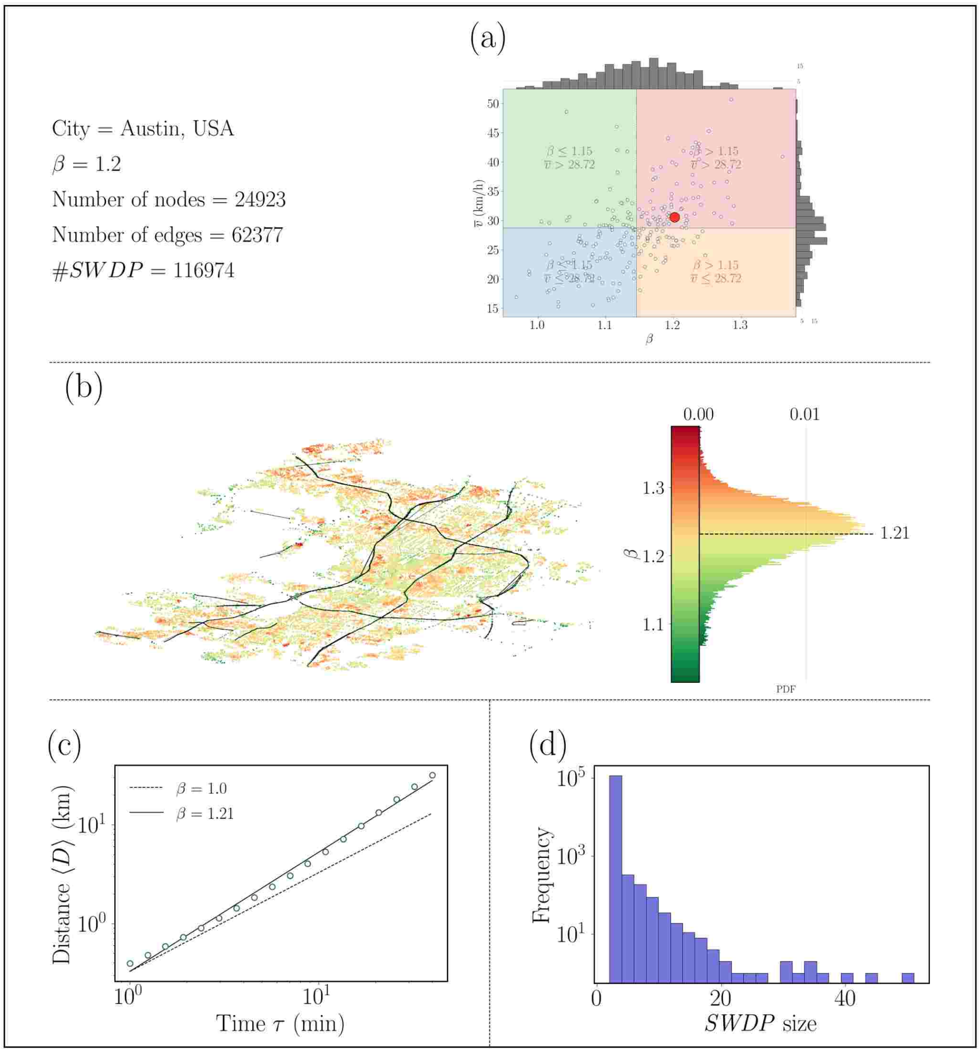

Fig. S11. Methodological sheet - Austin, USA. In (a) Each point represents a city, with mean exponent (β), on the x-axis, and mean speed $\overline{v}$ obtained in all trips made to calculate the exponent on the axis y. The histograms of the values of β and $\overline{v}$ are shown on the axes in the upper and right corners, respectively. The graph was segmented into four quadrants, in which the division is performed by the mean values of β and $\overline{v}$. The quadrants were colored and annotated according to the division criteria. The red dot represents the location of Austin, USA. In (b) taking all the nodes of Austin, USA as origin, the dots are colored as a function of their exponent value and their color is quantified by the color bar in the center. The longest segments without a deceleration point (SWDP) are plotted in black. The probability density function of the $\beta's$ for each experiment is shown on the left of the color scale Figure (c) shows the mean correlation curve between time τ and the distance $\langle D \rangle$. The black traced line represents the exponent equal to 1.0. Figure (d) shows the distribution of SWDP sizes in number of nodes per frequency of occurrence.

Bamako, Mali

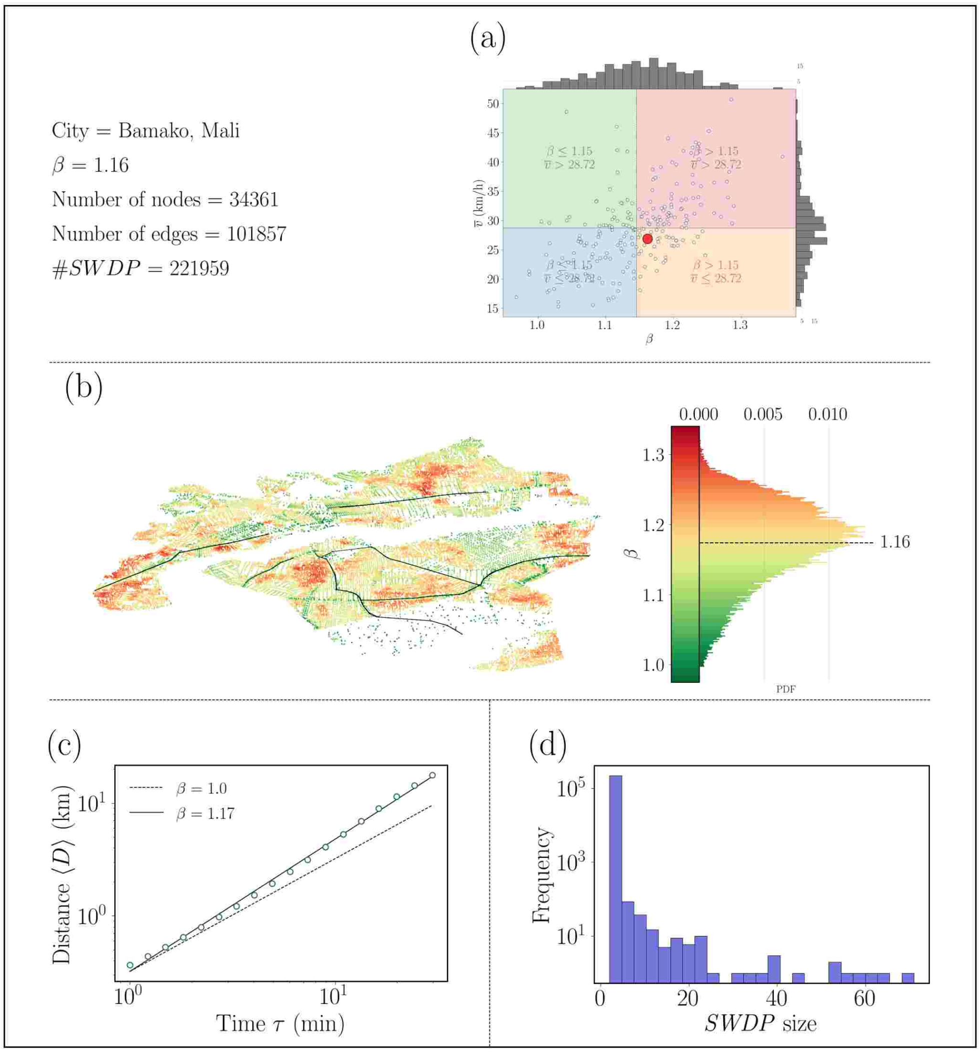

Fig. S12. Methodological sheet - Bamako, Mali. In (a) Each point represents a city, with mean exponent (β), on the x-axis, and mean speed $\overline{v}$ obtained in all trips made to calculate the exponent on the axis y. The histograms of the values of β and $\overline{v}$ are shown on the axes in the upper and right corners, respectively. The graph was segmented into four quadrants, in which the division is performed by the mean values of β and $\overline{v}$. The quadrants were colored and annotated according to the division criteria. The red dot represents the location of Bamako, Mali. In (b) taking all the nodes of Bamako, Mali as origin, the dots are colored as a function of their exponent value and their color is quantified by the color bar in the center. The longest segments without a deceleration point (SWDP) are plotted in black. The probability density function of the $\beta' s$ for each experiment is shown on the left of the color scale Figure (c) shows the mean correlation curve between time τ and the distance $\langle D \rangle$. The black traced line represents the exponent equal to 1.0. Figure (d) shows the distribution of SWDP sizes in number of nodes per frequency of occurrence.

Barcelona, Spain

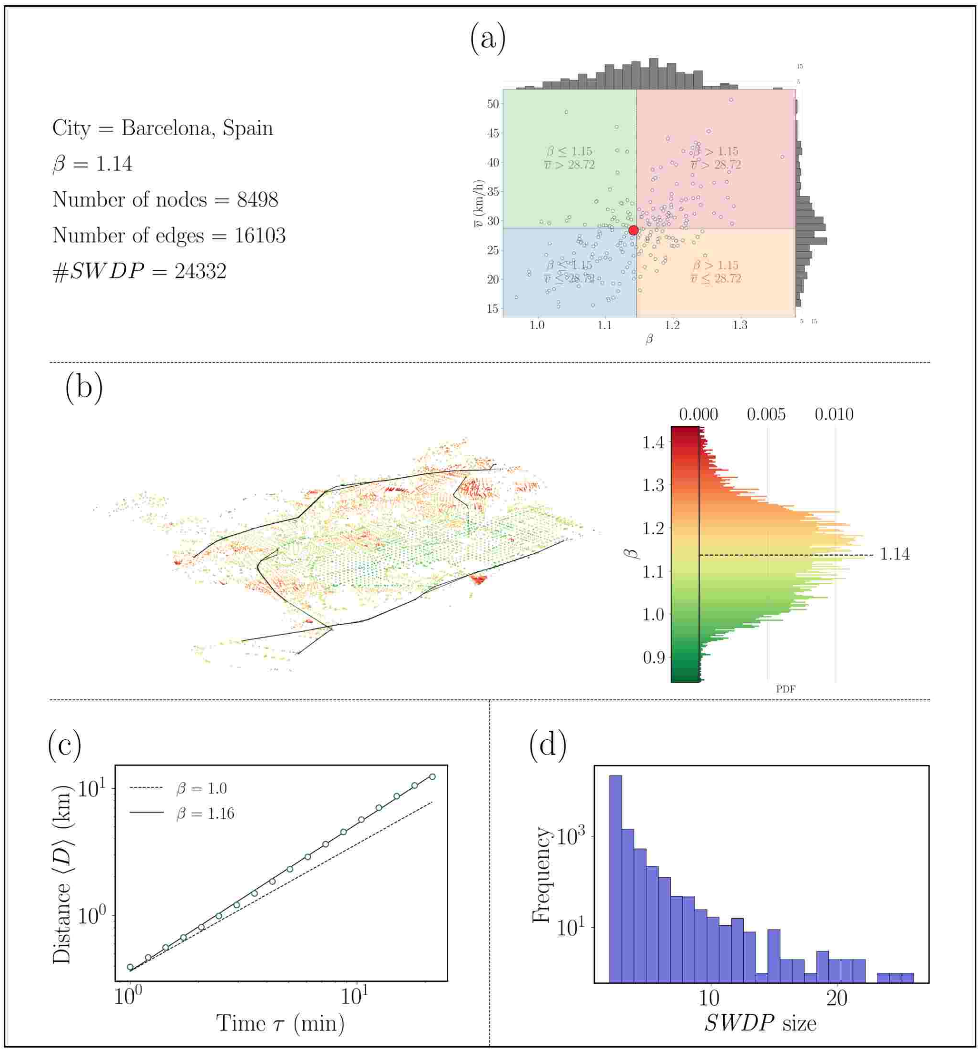

Fig. S13. Methodological sheet - Barcelona, Spain. In (a) Each point represents a city, with mean exponent (β), on the x-axis, and mean speed $\overline{v}$ obtained in all trips made to calculate the exponent on the axis y. The histograms of the values of β and $\overline{v}$ are shown on the axes in the upper and right corners, respectively. The graph was segmented into four quadrants, in which the division is performed by the mean values of β and $\overline{v}$. The quadrants were colored and annotated according to the division criteria. The red dot represents the location of Barcelona, Spain. In (b) taking all the nodes of Barcelona, Spain as origin, the dots are colored as a function of their exponent value and their color is quantified by the color bar in the center. The longest segments without a deceleration point (SWDP) are plotted in black. The probability density function of the $\beta' s$ for each experiment is shown on the left of the color scale Figure (c) shows the mean correlation curve between time τ and the distance $\langle D \rangle$. The black traced line represents the exponent equal to 1.0. Figure (d) shows the distribution of SWDP sizes in number of nodes per frequency of occurrence.

Baton Rouge, USA

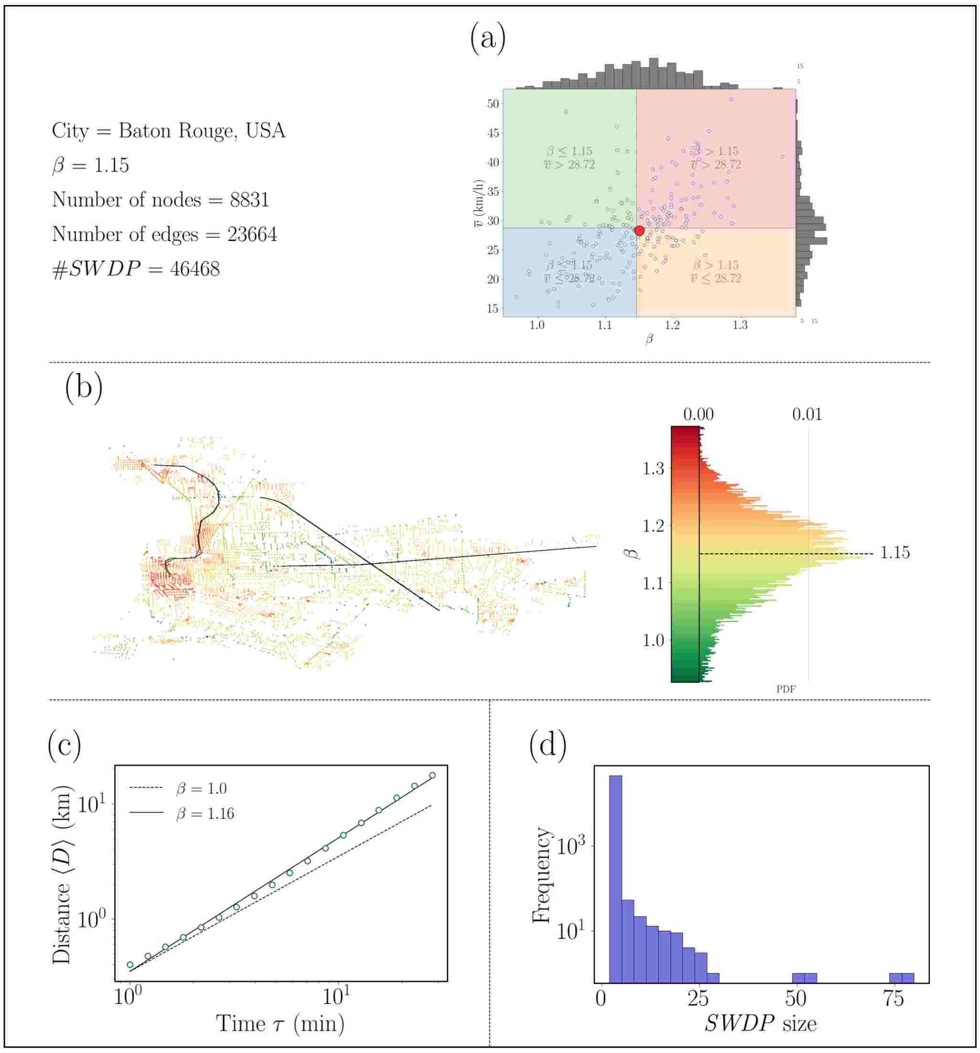

Fig. S14. Methodological sheet - Baton Rouge, USA. In (a) Each point represents a city, with mean exponent (β), on the x-axis, and mean speed $\overline{v}$ obtained in all trips made to calculate the exponent on the axis y. The histograms of the values of β and $\overline{v}$ are shown on the axes in the upper and right corners, respectively. The graph was segmented into four quadrants, in which the division is performed by the mean values of β and $\overline{v}$. The quadrants were colored and annotated according to the division criteria. The red dot represents the location of Baton Rouge, USA. In (b) taking all the nodes of Baton Rouge, USA as origin, the dots are colored as a function of their exponent value and their color is quantified by the color bar in the center. The longest segments without a deceleration point (SWDP) are plotted in black. The probability density function of the $\beta's$ for each experiment is shown on the left of the color scale Figure (c) shows the mean correlation curve between time τ and the distance $\langle D \rangle$. The black traced line represents the exponent equal to 1.0. Figure (d) shows the distribution of SWDP sizes in number of nodes per frequency of occurrence.

Beira, Mozambique

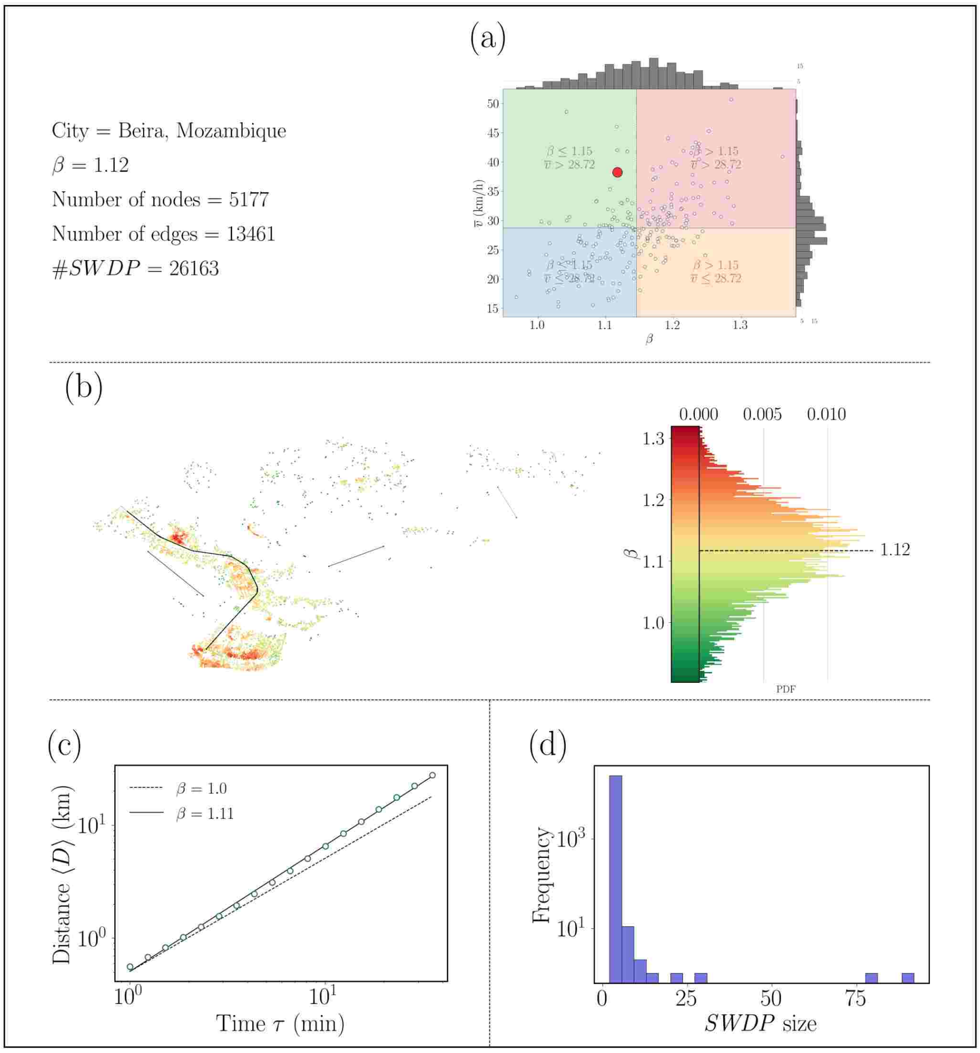

Fig. S15. Methodological sheet - Beira, Mozambique. In (a) Each point represents a city, with mean exponent (β), on the x-axis, and mean speed $\overline{v}$ obtained in all trips made to calculate the exponent on the axis y. The histograms of the values of β and $\overline{v}$ are shown on the axes in the upper and right corners, respectively. The graph was segmented into four quadrants, in which the division is performed by the mean values of β and $\overline{v}$. The quadrants were colored and annotated according to the division criteria. The red dot represents the location of Beira, Mozambique. In (b) taking all the nodes of Beira, Mozambique as origin, the dots are colored as a function of their exponent value and their color is quantified by the color bar in the center. The longest segments without a deceleration point (SWDP) are plotted in black. The probability density function of the $\beta's$ for each experiment is shown on the left of the color scale Figure (c) shows the mean correlation curve between time τ and the distance $\langle D \rangle$. The black traced line represents the exponent equal to 1.0. Figure (d) shows the distribution of SWDP sizes in number of nodes per frequency of occurrence.

Belgrade, Serbia

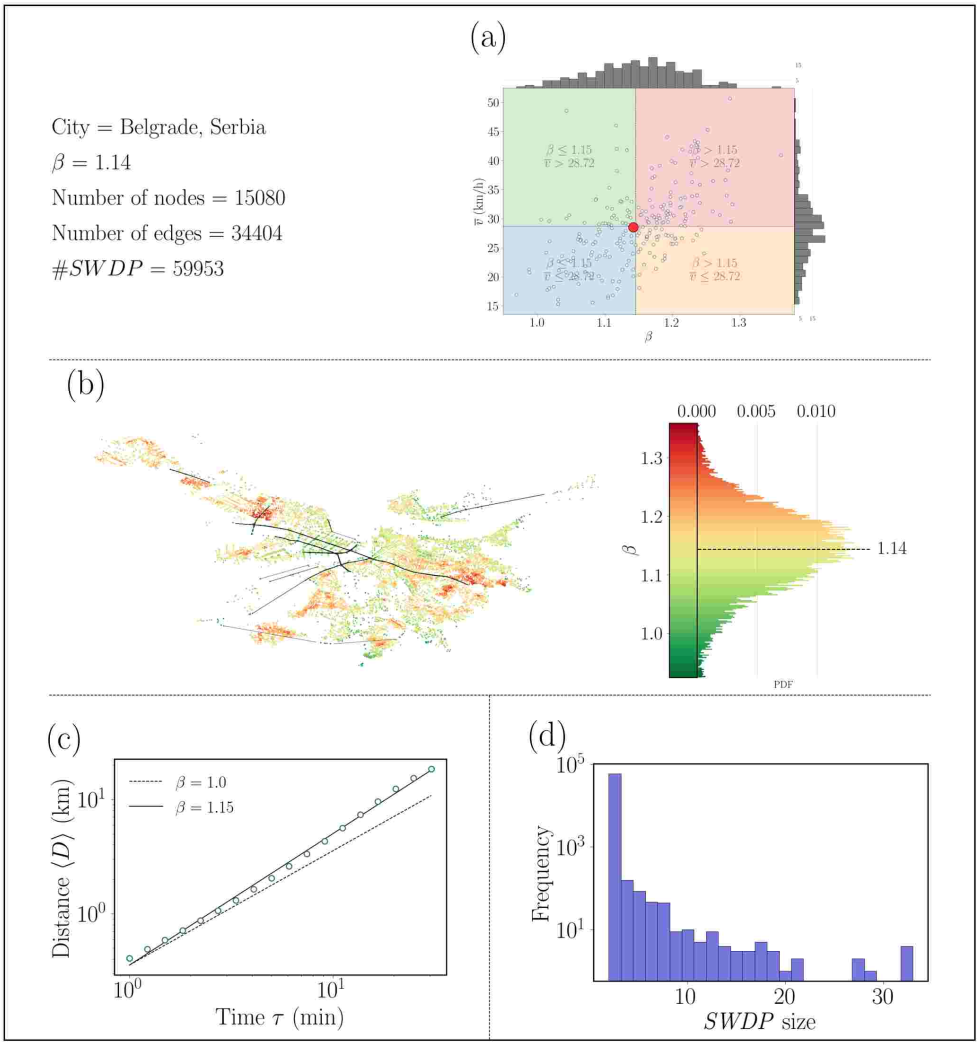

Fig. S16. Methodological sheet - Belgrade, Serbia. In (a) Each point represents a city, with mean exponent (β), on the x-axis, and mean speed $\overline{v}$ obtained in all trips made to calculate the exponent on the axis y. The histograms of the values of β and $\overline{v}$ are shown on the axes in the upper and right corners, respectively. The graph was segmented into four quadrants, in which the division is performed by the mean values of β and $\overline{v}$. The quadrants were colored and annotated according to the division criteria. The red dot represents the location of Belgrade, Serbia. In (b) taking all the nodes of Belgrade, Serbia as origin, the dots are colored as a function of their exponent value and their color is quantified by the color bar in the center. The longest segments without a deceleration point (SWDP) are plotted in black. The probability density function of the $\beta's$ for each experiment is shown on the left of the color scale Figure (c) shows the mean correlation curve between time τ and the distance $\langle D \rangle$. The black traced line represents the exponent equal to 1.0. Figure (d) shows the distribution of SWDP sizes in number of nodes per frequency of occurrence.

Bengaluru, India

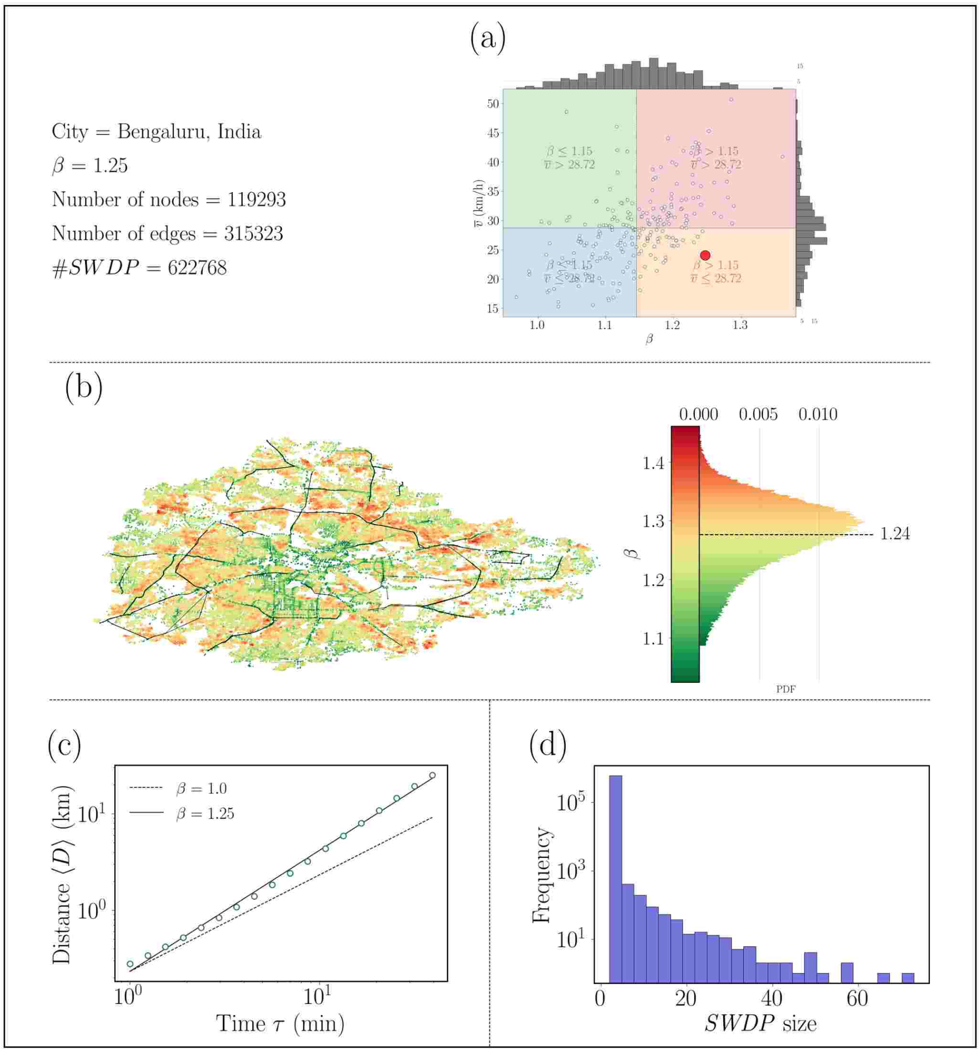

Fig. S17. Methodological sheet - Bengaluru, India. In (a) Each point represents a city, with mean exponent (β), on the x-axis, and mean speed $\overline{v}$ obtained in all trips made to calculate the exponent on the axis y. The histograms of the values of β and $\overline{v}$ are shown on the axes in the upper and right corners, respectively. The graph was segmented into four quadrants, in which the division is performed by the mean values of β and $\overline{v}$. The quadrants were colored and annotated according to the division criteria. The red dot represents the location of Bengaluru, India. In (b) taking all the nodes of Bengaluru, India as origin, the dots are colored as a function of their exponent value and their color is quantified by the color bar in the center. The longest segments without a deceleration point (SWDP) are plotted in black. The probability density function of the $\beta's$ for each experiment is shown on the left of the color scale Figure (c) shows the mean correlation curve between time τ and the distance $\langle D \rangle$. The black traced line represents the exponent equal to 1.0. Figure (d) shows the distribution of SWDP sizes in number of nodes per frequency of occurrence.

Bern, Switzerland

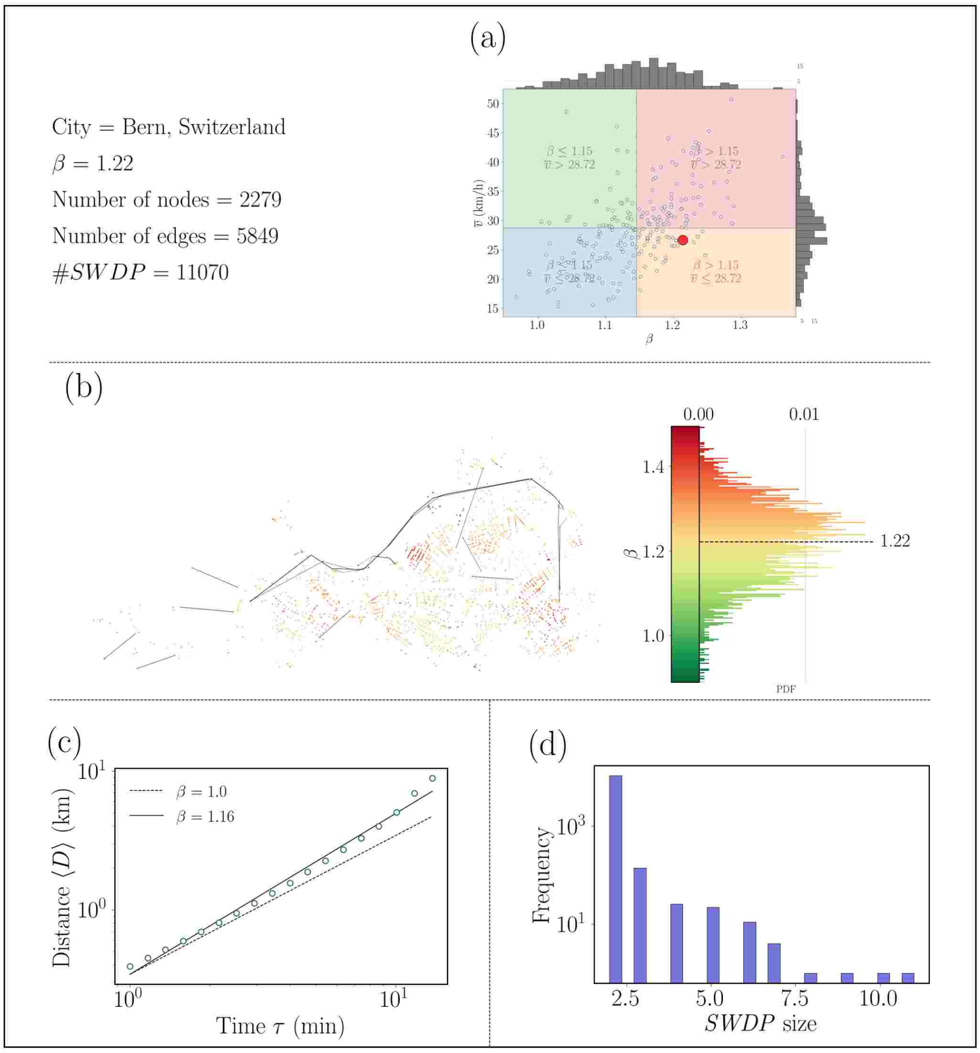

Fig. S18. Methodological sheet - Bern, Switzerland. In (a) Each point represents a city, with mean exponent (β), on the x-axis, and mean speed $\overline{v}$ obtained in all trips made to calculate the exponent on the axis y. The histograms of the values of β and $\overline{v}$ are shown on the axes in the upper and right corners, respectively. The graph was segmented into four quadrants, in which the division is performed by the mean values of β and $\overline{v}$. The quadrants were colored and annotated according to the division criteria. The red dot represents the location of Bern, Switzerland. In (b) taking all the nodes of Bern, Switzerland as origin, the dots are colored as a function of their exponent value and their color is quantified by the color bar in the center. The longest segments without a deceleration point (SWDP) are plotted in black. The probability density function of the $\beta's$ for each experiment is shown on the left of the color scale Figure (c) shows the mean correlation curve between time τ and the distance $\langle D \rangle$. The black traced line represents the exponent equal to 1.0. Figure (d) shows the distribution of SWDP sizes in number of nodes per frequency of occurrence.

Birmingham, UK

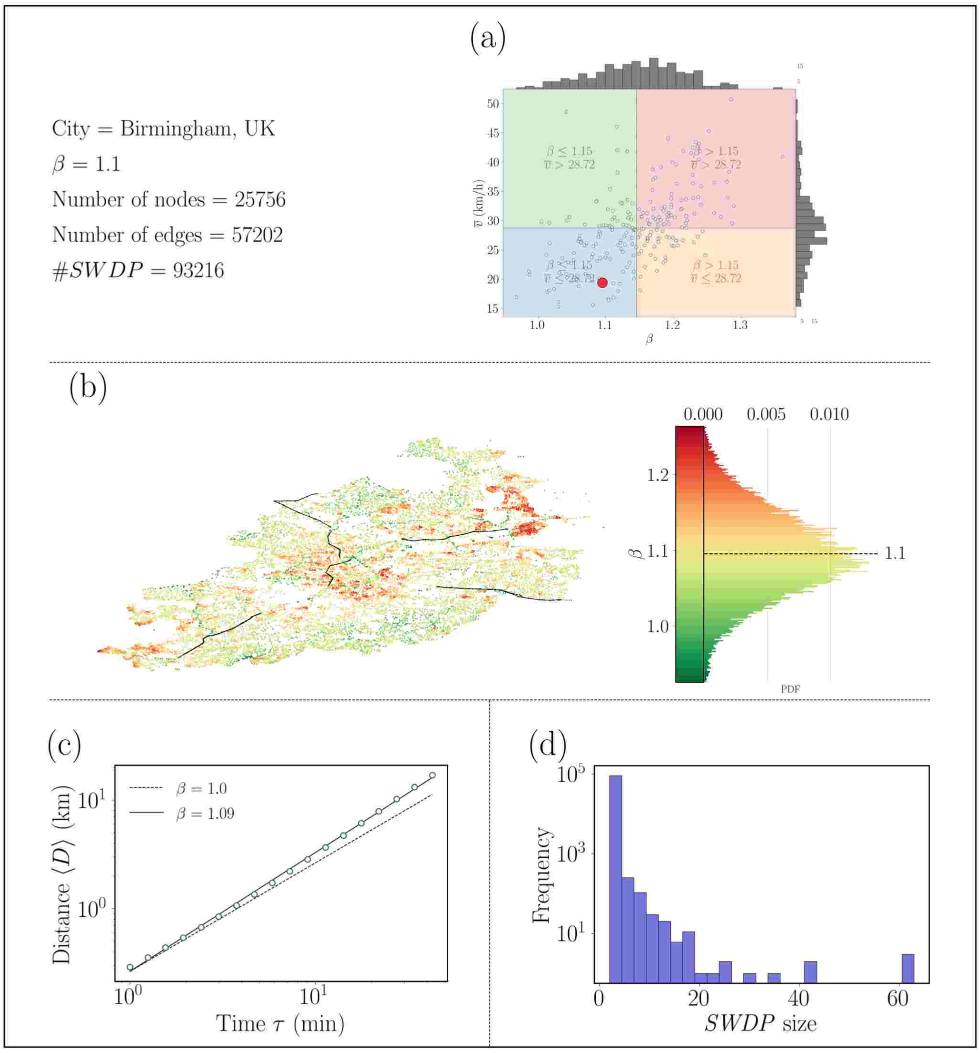

Fig. S19. Methodological sheet - Birmingham, UK. In (a) Each point represents a city, with mean exponent (β), on the x-axis, and mean speed $\overline{v}$ obtained in all trips made to calculate the exponent on the axis y. The histograms of the values of β and $\overline{v}$ are shown on the axes in the upper and right corners, respectively. The graph was segmented into four quadrants, in which the division is performed by the mean values of β and $\overline{v}$. The quadrants were colored and annotated according to the division criteria. The red dot represents the location of Birmingham, UK. In (b) taking all the nodes of Birmingham, UK as origin, the dots are colored as a function of their exponent value and their color is quantified by the color bar in the center. The longest segments without a deceleration point (SWDP) are plotted in black. The probability density function of the $\beta's$ for each experiment is shown on the left of the color scale Figure (c) shows the mean correlation curve between time τ and the distance $\langle D \rangle$. The black traced line represents the exponent equal to 1.0. Figure (d) shows the distribution of SWDP sizes in number of nodes per frequency of occurrence.

Bogotá, Colombia

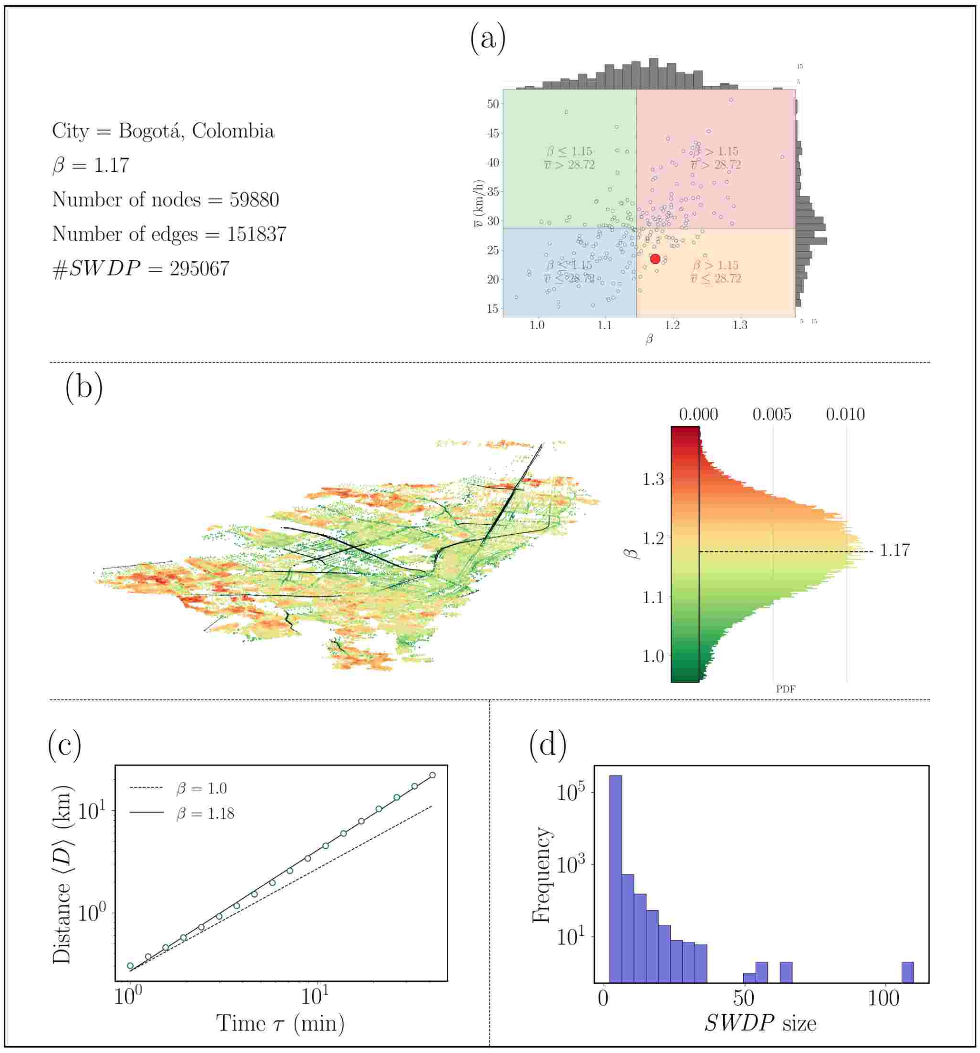

Fig. S20. Methodological sheet - Bogotá, Colombia. In (a) Each point represents a city, with mean exponent (β), on the x-axis, and mean speed $\overline{v}$ obtained in all trips made to calculate the exponent on the axis y. The histograms of the values of β and $\overline{v}$ are shown on the axes in the upper and right corners, respectively. The graph was segmented into four quadrants, in which the division is performed by the mean values of β and $\overline{v}$. The quadrants were colored and annotated according to the division criteria. The red dot represents the location of Bogotá, Colombia. In (b) taking all the nodes of Bogotá, Colombia as origin, the dots are colored as a function of their exponent value and their color is quantified by the color bar in the center. The longest segments without a deceleration point (SWDP) are plotted in black. The probability density function of the $\beta's$ for each experiment is shown on the left of the color scale Figure (c) shows the mean correlation curve between time τ and the distance $\langle D \rangle$. The black traced line represents the exponent equal to 1.0. Figure (d) shows the distribution of SWDP sizes in number of nodes per frequency of occurrence.

Boston, USA

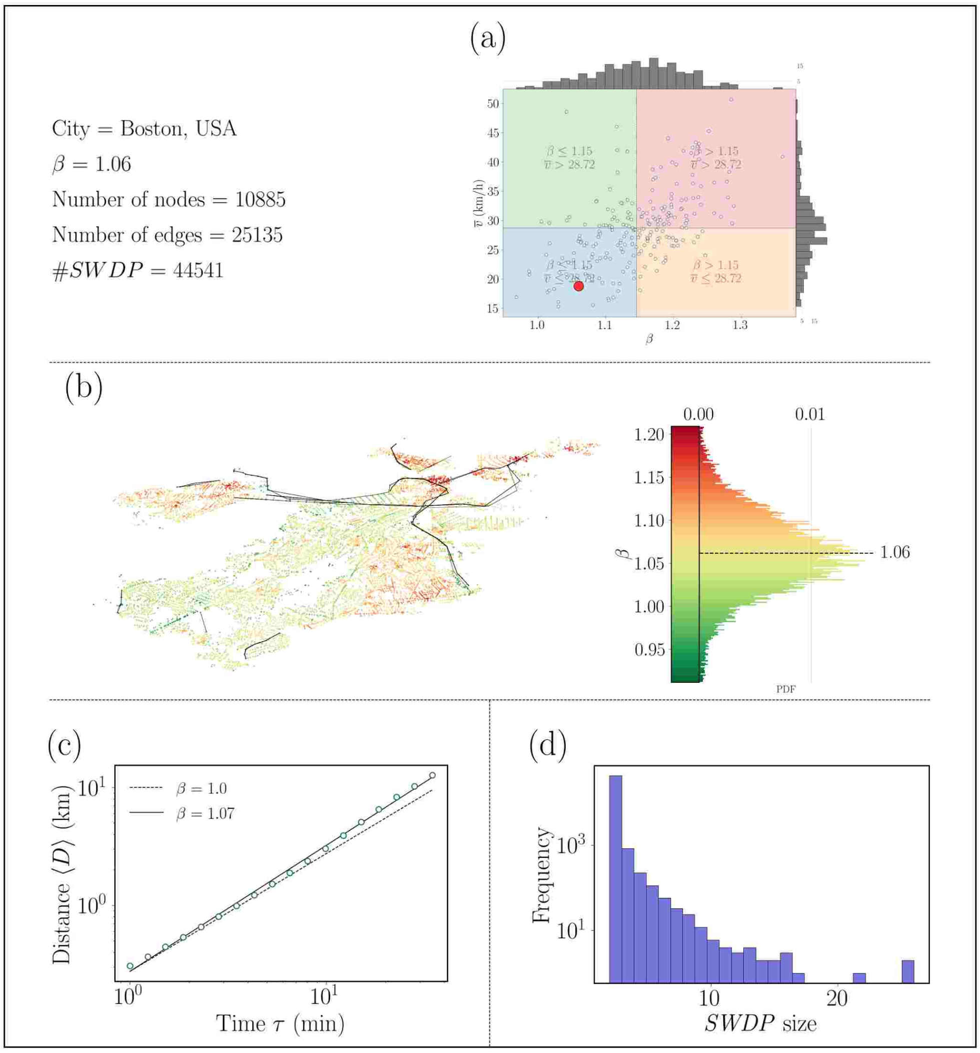

Fig. S21. Methodological sheet - Boston, USA. In (a) Each point represents a city, with mean exponent (β), on the x-axis, and mean speed $\overline{v}$ obtained in all trips made to calculate the exponent on the axis y. The histograms of the values of β and $\overline{v}$ are shown on the axes in the upper and right corners, respectively. The graph was segmented into four quadrants, in which the division is performed by the mean values of β and $\overline{v}$. The quadrants were colored and annotated according to the division criteria. The red dot represents the location of Boston, USA. In (b) taking all the nodes of Boston, USA as origin, the dots are colored as a function of their exponent value and their color is quantified by the color bar in the center. The longest segments without a deceleration point (SWDP) are plotted in black. The probability density function of the $\beta's$ for each experiment is shown on the left of the color scale Figure (c) shows the mean correlation curve between time τ and the distance $\langle D \rangle$. The black traced line represents the exponent equal to 1.0. Figure (d) shows the distribution of SWDP sizes in number of nodes per frequency of occurrence.

Boulder, USA

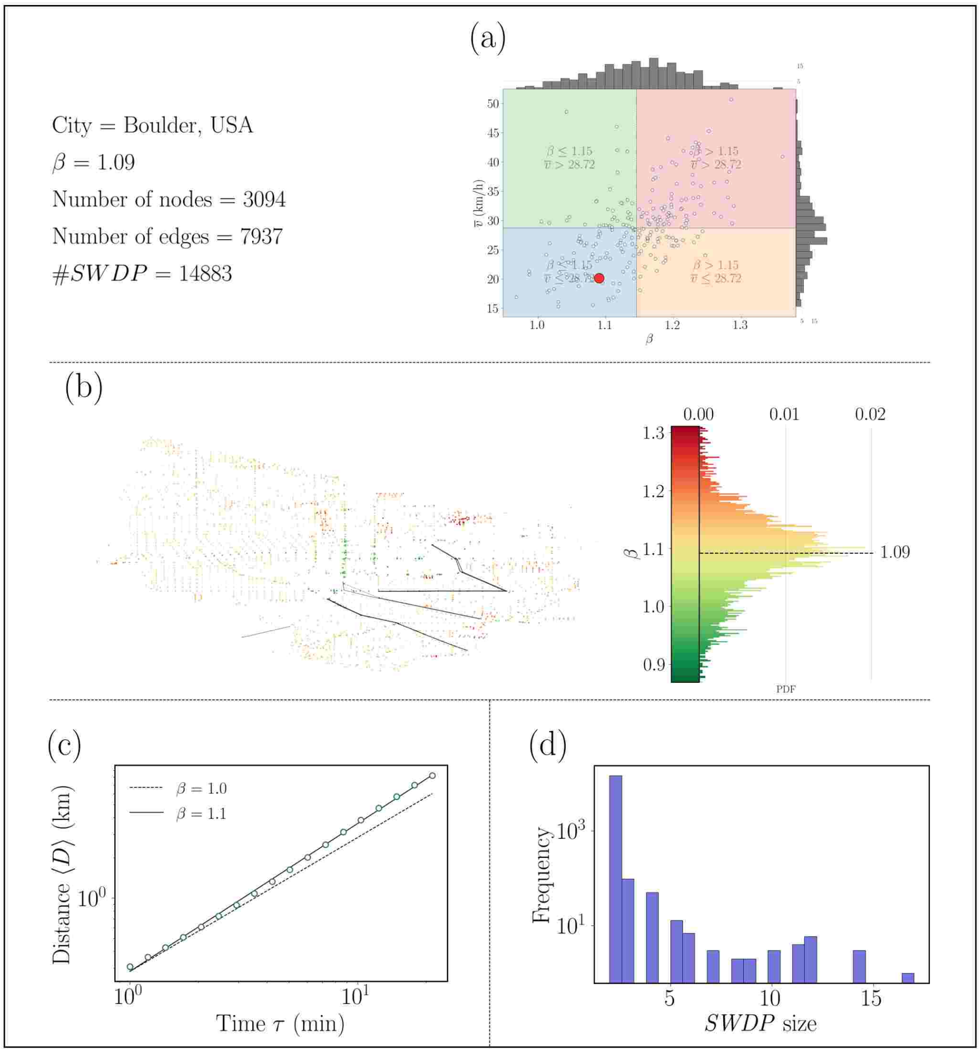

Fig. S22. Methodological sheet - Boulder, USA. In (a) Each point represents a city, with mean exponent (β), on the x-axis, and mean speed $\overline{v}$ obtained in all trips made to calculate the exponent on the axis y. The histograms of the values of β and $\overline{v}$ are shown on the axes in the upper and right corners, respectively. The graph was segmented into four quadrants, in which the division is performed by the mean values of β and $\overline{v}$. The quadrants were colored and annotated according to the division criteria. The red dot represents the location of Boulder, USA. In (b) taking all the nodes of Boulder, USA as origin, the dots are colored as a function of their exponent value and their color is quantified by the color bar in the center. The longest segments without a deceleration point (SWDP) are plotted in black. The probability density function of the $\beta's$ for each experiment is shown on the left of the color scale Figure (c) shows the mean correlation curve between time τ and the distance $\langle D\rangle$. The black traced line represents the exponent equal to 1.0. Figure (d) shows the distribution of SWDP sizes in number of nodes per frequency of occurrence.

Brisbane, Australia

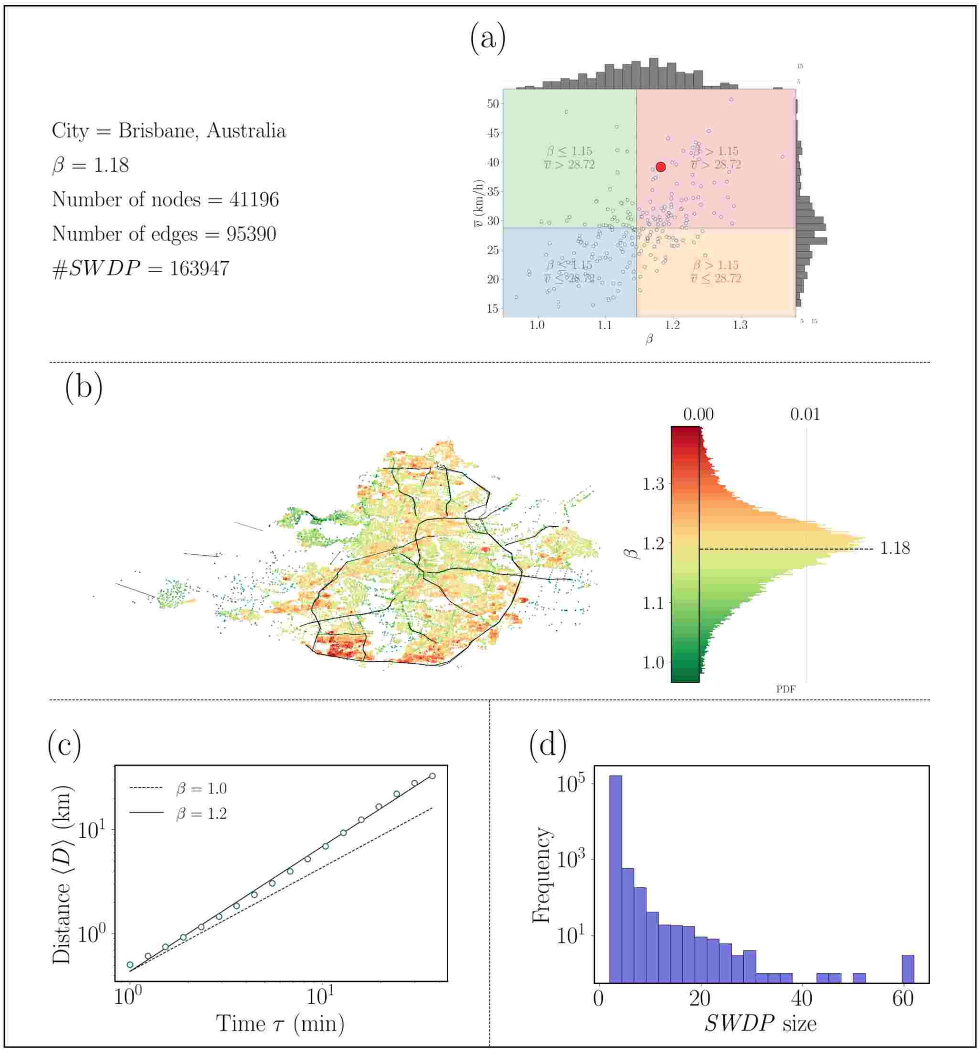

Fig. S23. Methodological sheet - Brisbane, Australia. In (a) Each point represents a city, with mean exponent (β), on the x-axis, and mean speed $\overline{v}$ obtained in all trips made to calculate the exponent on the axis y. The histograms of the values of β and $\overline{v}$ are shown on the axes in the upper and right corners, respectively. The graph was segmented into four quadrants, in which the division is performed by the mean values of β and $\overline{v}$. The quadrants were colored and annotated according to the division criteria. The red dot represents the location of Brisbane, Australia. In (b) taking all the nodes of Brisbane, Australia as origin, the dots are colored as a function of their exponent value and their color is quantified by the color bar in the center. The longest segments without a deceleration point (SWDP) are plotted in black. The probability density function of the $\beta's$ for each experiment is shown on the left of the color scale Figure (c) shows the mean correlation curve between time τ and the distance $\langle D \rangle$. The black traced line represents the exponent equal to 1.0. Figure (d) shows the distribution of SWDP sizes in number of nodes per frequency of occurrence.

Bristol, UK

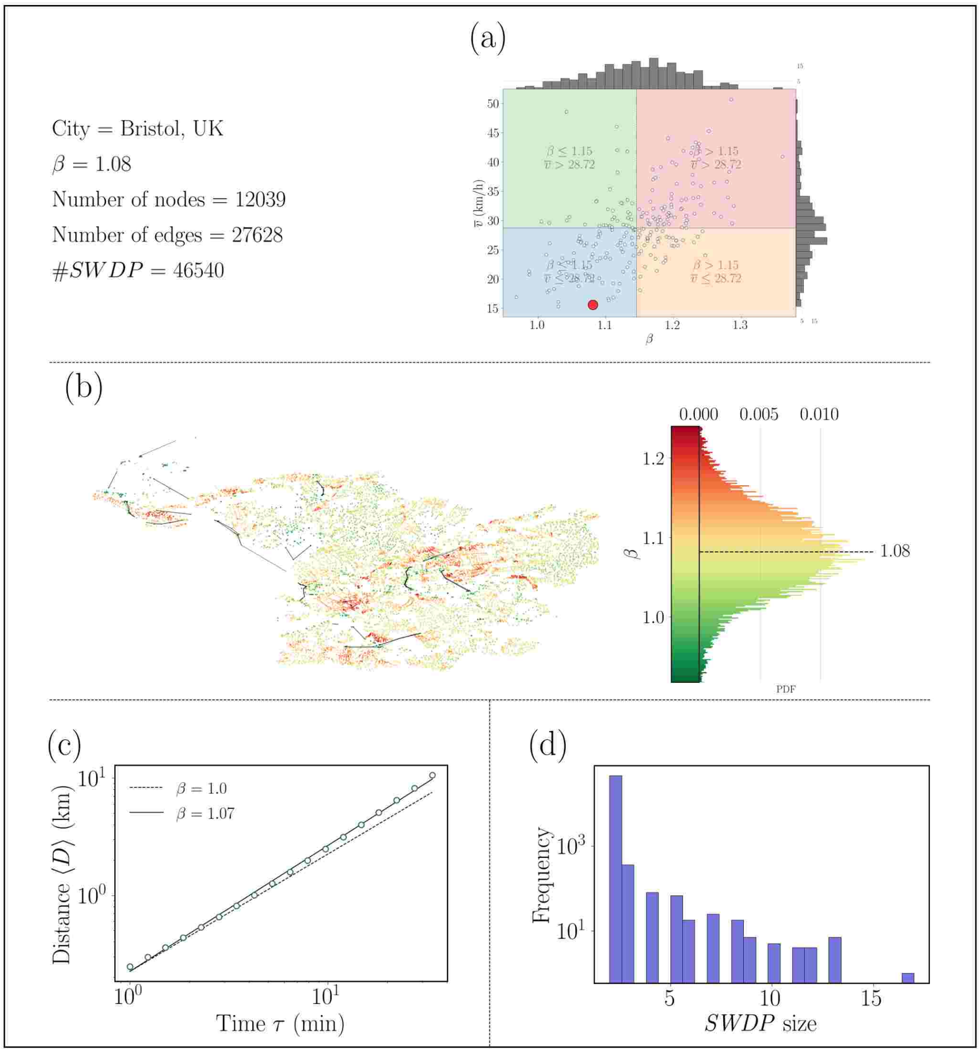

Fig. S24. Methodological sheet - Bristol, UK. In (a) Each point represents a city, with mean exponent (β), on the x-axis, and mean speed $\overline{v}$ obtained in all trips made to calculate the exponent on the axis y. The histograms of the values of β and $\overline{v}$ are shown on the axes in the upper and right corners, respectively. The graph was segmented into four quadrants, in which the division is performed by the mean values of β and $\overline{v}$. The quadrants were colored and annotated according to the division criteria. The red dot represents the location of Bristol, UK. In (b) taking all the nodes of Bristol, UK as origin, the dots are colored as a function of their exponent value and their color is quantified by the color bar in the center. The longest segments without a deceleration point (SWDP) are plotted in black. The probability density function of the $\beta's$ for each experiment is shown on the left of the color scale Figure (c) shows the mean correlation curve between time τ and the distance $\langle D \rangle$. The black traced line represents the exponent equal to 1.0. Figure (d) shows the distribution of SWDP sizes in number of nodes per frequency of occurrence.

Brussels, Belgium

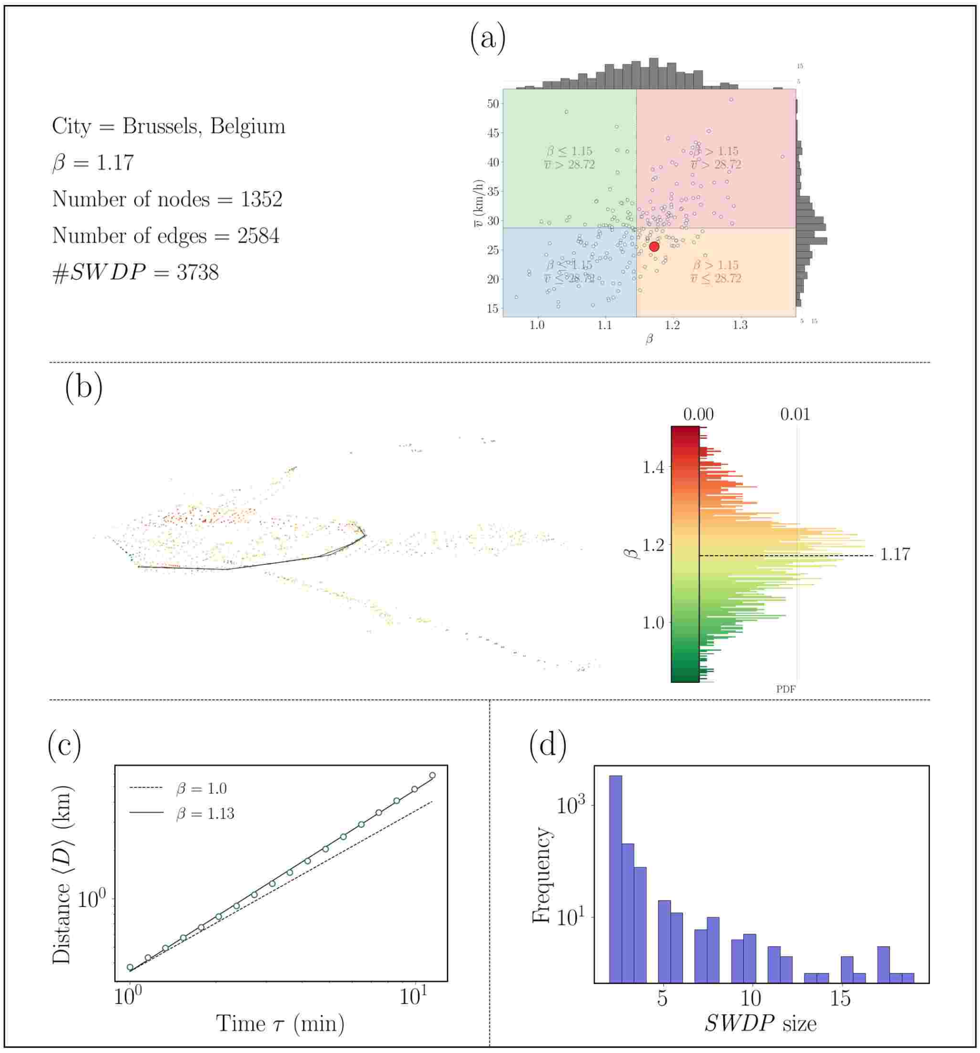

Fig. S25. Methodological sheet - Brussels, Belgium. In (a) Each point represents a city, with mean exponent (β), on the x-axis, and mean speed $\overline{v}$ obtained in all trips made to calculate the exponent on the axis y. The histograms of the values of β and $\overline{v}$ are shown on the axes in the upper and right corners, respectively. The graph was segmented into four quadrants, in which the division is performed by the mean values of β and $\overline{v}$. The quadrants were colored and annotated according to the division criteria. The red dot represents the location of Brussels, Belgium. In (b) taking all the nodes of Brussels, Belgium as origin, the dots are colored as a function of their exponent value and their color is quantified by the color bar in the center. The longest segments without a deceleration point (SWDP) are plotted in black. The probability density function of the $\beta's$ for each experiment is shown on the left of the color scale Figure (c) shows the mean correlation curve between time τ and the distance $\langle D \rangle$. The black traced line represents the exponent equal to 1.0. Figure (d) shows the distribution of SWDP sizes in number of nodes per frequency of occurrence.

Bucharest, Romania

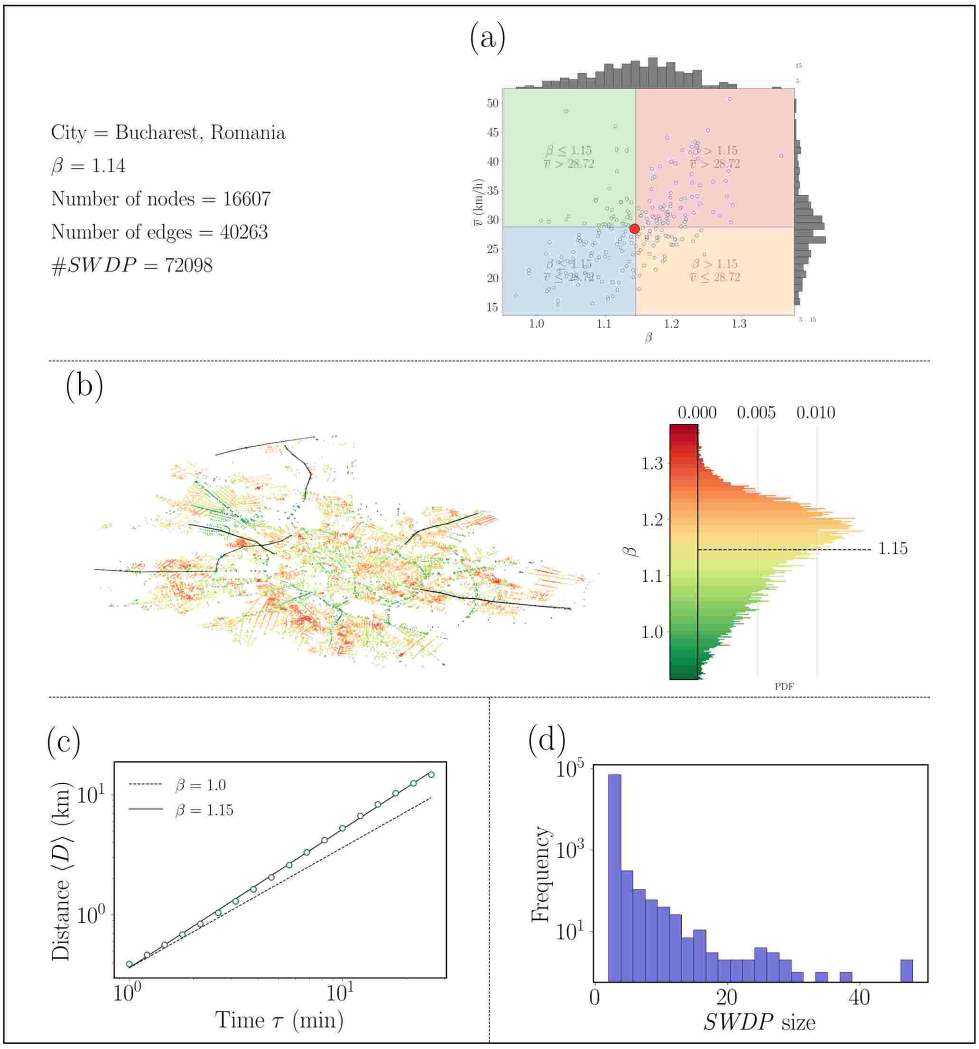

Fig. S26. Methodological sheet - Bucharest, Romania. In (a) Each point represents a city, with mean exponent (β), on the x-axis, and mean speed $\overline{v}$ obtained in all trips made to calculate the exponent on the axis y. The histograms of the values of β and $\overline{v}$ are shown on the axes in the upper and right corners, respectively. The graph was segmented into four quadrants, in which the division is performed by the mean values of β and $\overline{v}$. The quadrants were colored and annotated according to the division criteria. The red dot represents the location of Bucharest, Romania. In (b) taking all the nodes of Bucharest, Romania as origin, the dots are colored as a function of their exponent value and their color is quantified by the color bar in the center. The longest segments without a deceleration point (SWDP) are plotted in black. The probability density function of the $\beta's$ for each experiment is shown on the left of the color scale Figure (c) shows the mean correlation curve between time τ and the distance $\langle D \rangle$. The black traced line represents the exponent equal to 1.0. Figure (d) shows the distribution of SWDP sizes in number of nodes per frequency of occurrence.

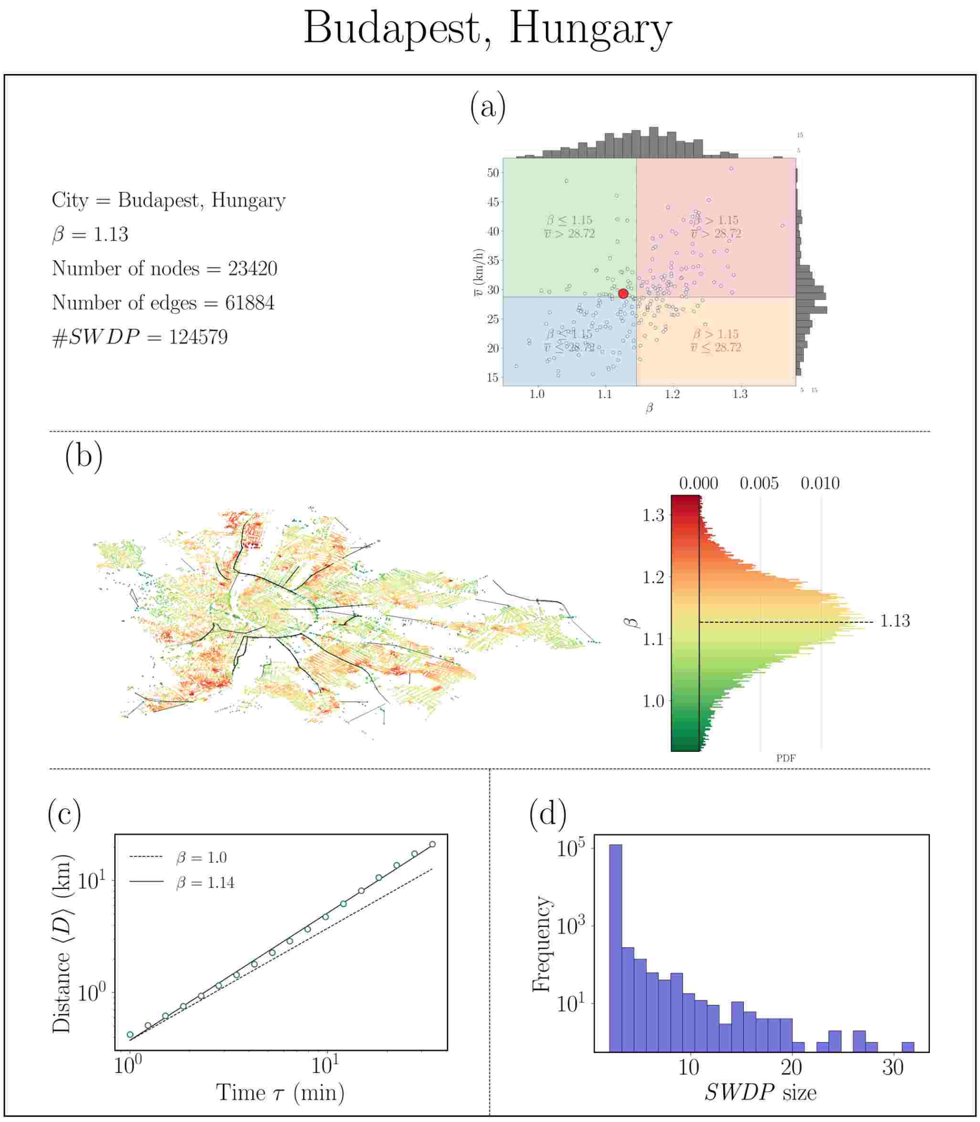

Fig. S27. Methodological sheet - Budapest, Hungary. In (a) Each point represents a city, with mean exponent (β), on the x-axis, and mean speed $\overline{v}$ obtained in all trips made to calculate the exponent on the axis y. The histograms of the values of β and $\overline{v}$ are shown on the axes in the upper and right corners, respectively. The graph was segmented into four quadrants, in which the division is performed by the mean values of β and $\overline{v}$. The quadrants were colored and annotated according to the division criteria. The red dot represents the location of Budapest, Hungary. In (b) taking all the nodes of Budapest, Hungary as origin, the dots are colored as a function of their exponent value and their color is quantified by the color bar in the center. The longest segments without a deceleration point (SWDP) are plotted in black. The probability density function of the $\beta's$ for each experiment is shown on the left of the color scale Figure (c) shows the mean correlation curve between time τ and the distance $\langle D \rangle$. The black traced line represents the exponent equal to 1.0. Figure (d) shows the distribution of SWDP sizes in number of nodes per frequency of occurrence.

Buenos Aires, Argentina

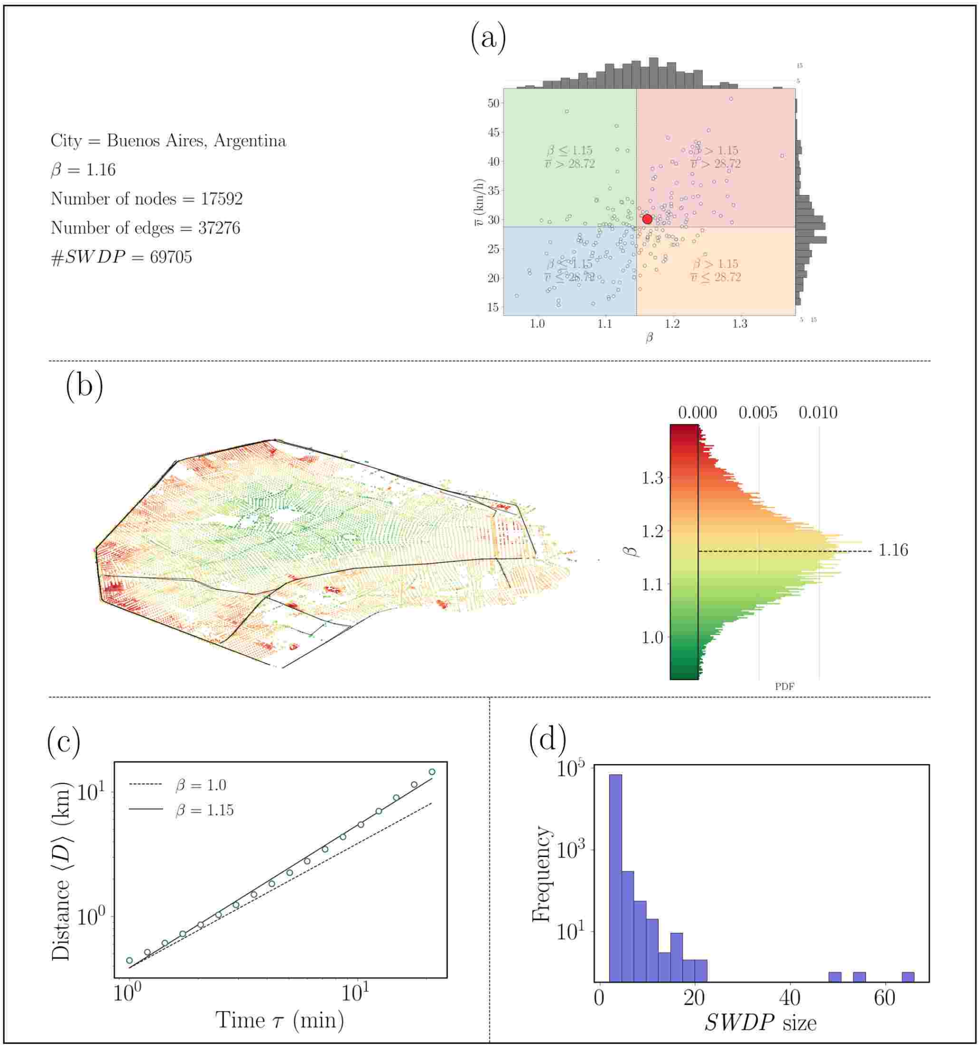

Fig. S28. Methodological sheet - Buenos Aires, Argentina. In (a) Each point represents a city, with mean exponent (β), on the x-axis, and mean speed $\overline{v}$ obtained in all trips made to calculate the exponent on the axis y. The histograms of the values of β and $\overline{v}$ are shown on the axes in the upper and right corners, respectively. The graph was segmented into four quadrants, in which the division is performed by the mean values of β and $\overline{v}$. The quadrants were colored and annotated according to the division criteria. The red dot represents the location of Buenos Aires, Argentina. In (b) taking all the nodes of Buenos Aires, Argentina as origin, the dots are colored as a function of their exponent value and their color is quantified by the color bar in the center. The longest segments without a deceleration point (SWDP) are plotted in black. The probability density function of the $\beta's$ for each experiment is shown on the left of the color scale Figure (c) shows the mean correlation curve between time τ and the distance $\langle D \rangle$. The black traced line represents the exponent equal to 1.0. Figure (d) shows the distribution of SWDP sizes in number of nodes per frequency of occurrence.

Buffalo, New York, USA

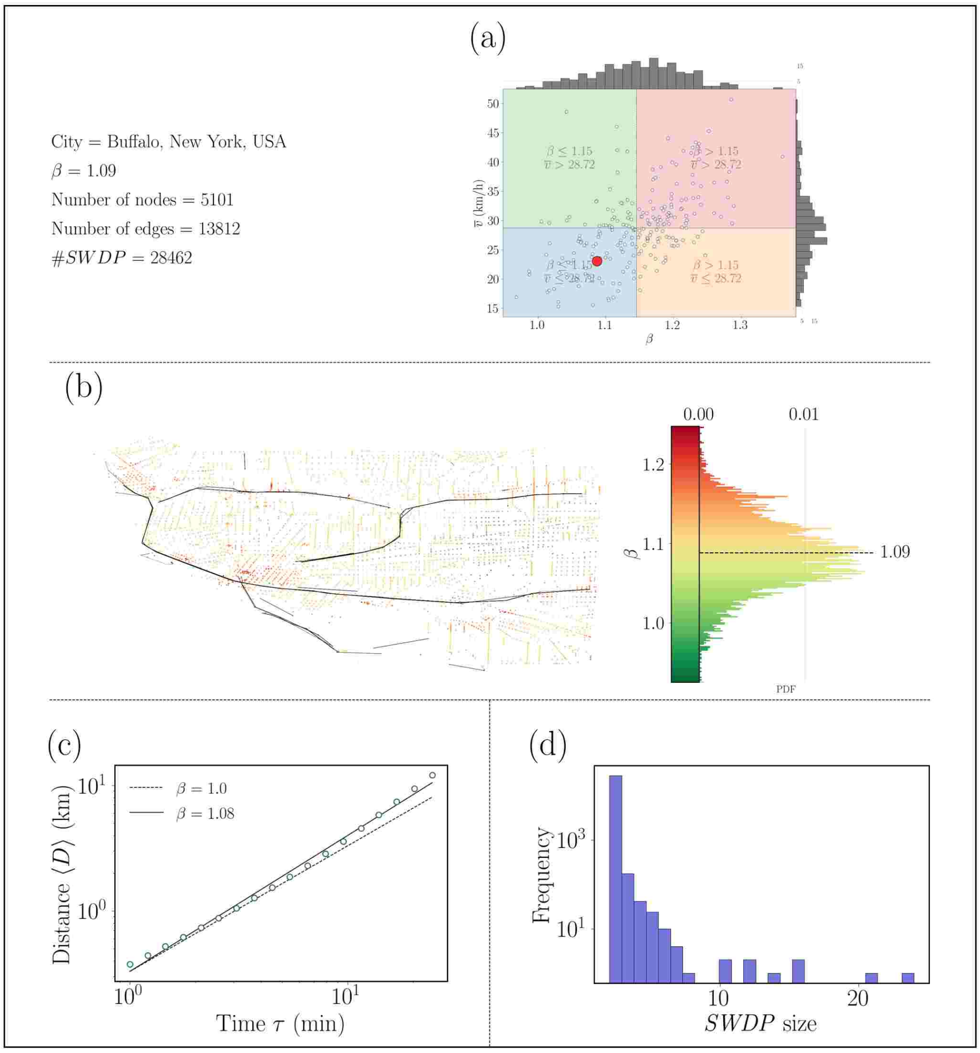

Fig. S29. Methodological sheet - Buffalo, New York, USA. In (a) Each point represents a city, with mean exponent (β), on the x-axis, and mean speed $\overline{v}$ obtained in all trips made to calculate the exponent on the axis y. The histograms of the values of β and $\overline{v}$ are shown on the axes in the upper and right corners, respectively. The graph was segmented into four quadrants, in which the division is performed by the mean values of β and $\overline{v}$. The quadrants were colored and annotated according to the division criteria. The red dot represents the location of Buffalo, New York, USA. In (b) taking all the nodes of Buffalo, New York, USA as origin, the dots are colored as a function of their exponent value and their color is quantified by the color bar in the center. The longest segments without a deceleration point (SWDP) are plotted in black. The probability density function of the $\beta's$ for each experiment is shown on the left of the color scale Figure (c) shows the mean correlation curve between time τ and the distance $\langle D \rangle$. The black traced line represents the exponent equal to 1.0. Figure (d) shows the distribution of SWDP sizes in number of nodes per frequency of occurrence.

Cairo, Egypt

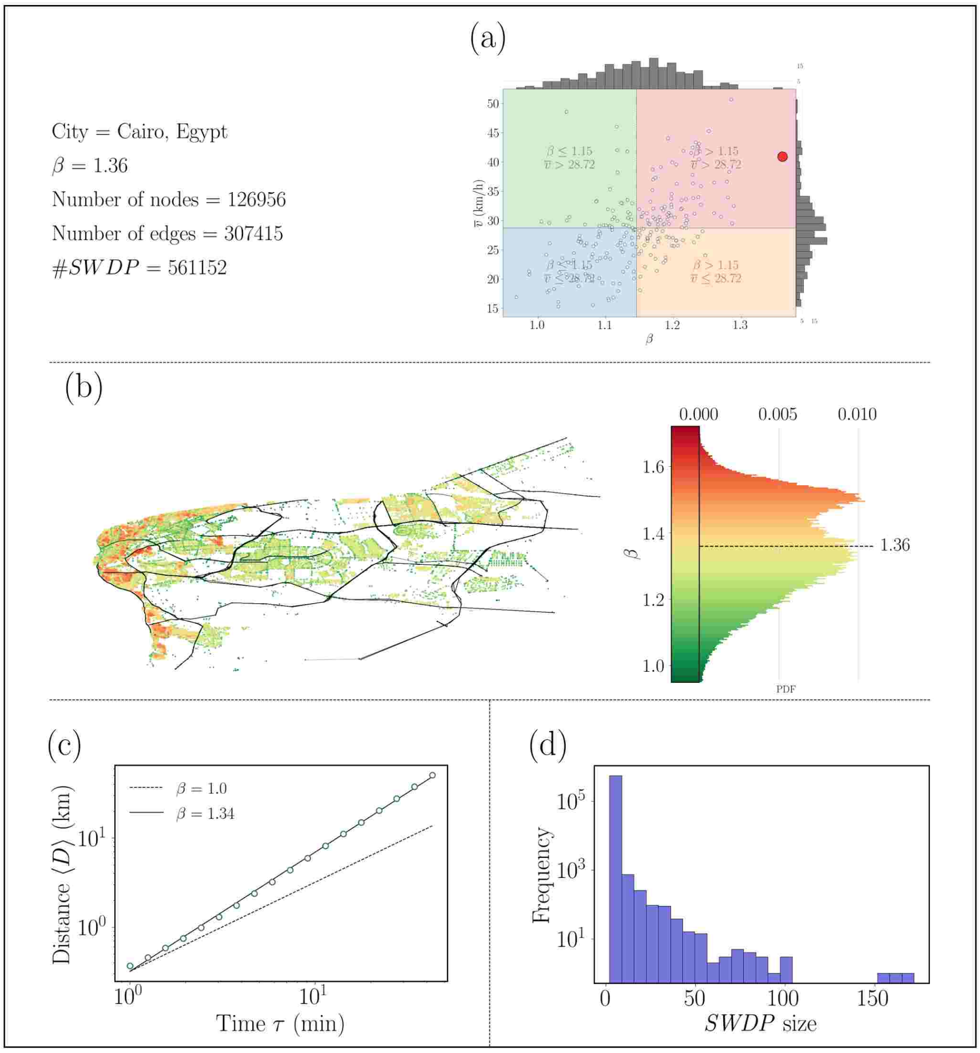

Fig. S30. Methodological sheet - Cairo, Egypt. In (a) Each point represents a city, with mean exponent (β), on the x-axis, and mean speed $\overline{v}$ obtained in all trips made to calculate the exponent on the axis y. The histograms of the values of β and $\overline{v}$ are shown on the axes in the upper and right corners, respectively. The graph was segmented into four quadrants, in which the division is performed by the mean values of β and $\overline{v}$. The quadrants were colored and annotated according to the division criteria. The red dot represents the location of Cairo, Egypt. In (b) taking all the nodes of Cairo, Egypt as origin, the dots are colored as a function of their exponent value and their color is quantified by the color bar in the center. The longest segments without a deceleration point (SWDP) are plotted in black. The probability density function of the $\beta's$ for each experiment is shown on the left of the color scale Figure (c) shows the mean correlation curve between time τ and the distance $\langle D \rangle$. The black traced line represents the exponent equal to 1.0. Figure (d) shows the distribution of SWDP sizes in number of nodes per frequency of occurrence.

Calgary, Canadá

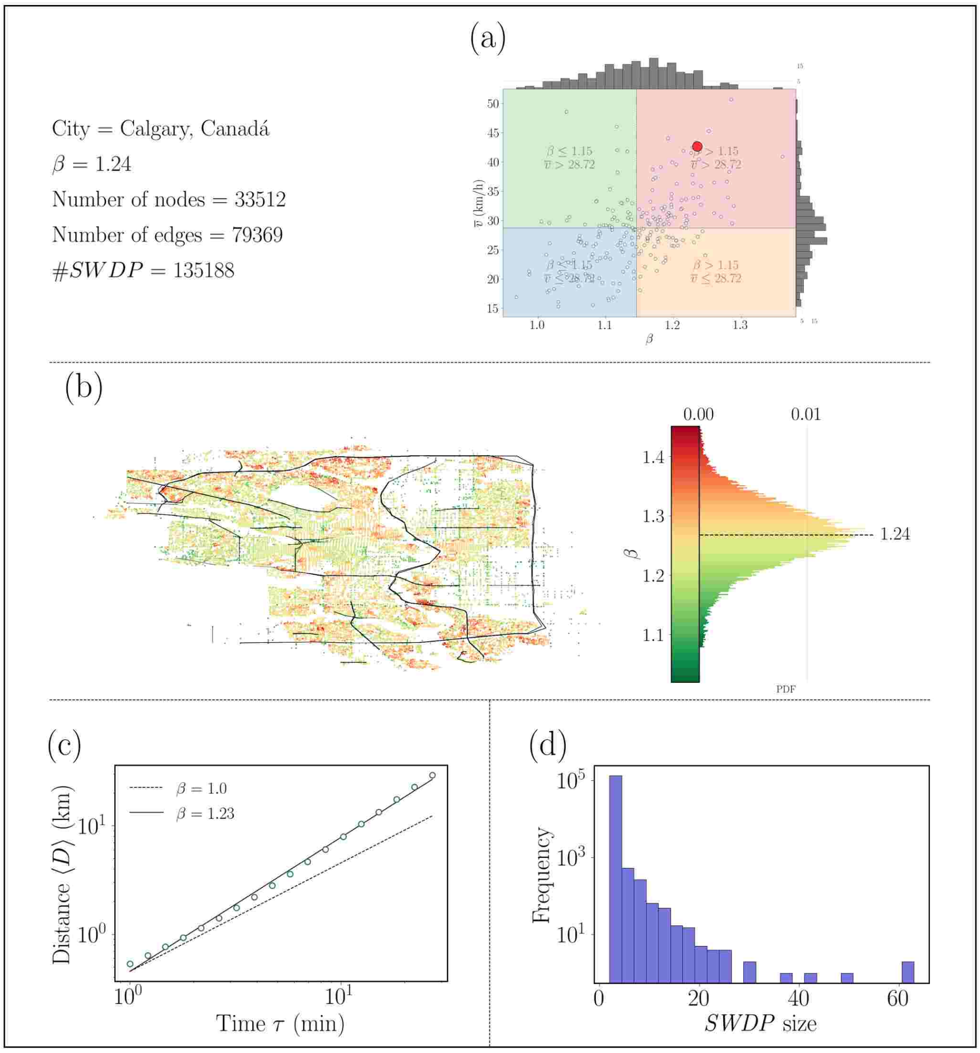

Fig. S31. Methodological sheet - Calgary, Canadá. In (a) Each point represents a city, with mean exponent (β), on the x-axis, and mean speed $\overline{v}$ obtained in all trips made to calculate the exponent on the axis y. The histograms of the values of β and $\overline{v}$ are shown on the axes in the upper and right corners, respectively. The graph was segmented into four quadrants, in which the division is performed by the mean values of β and $\overline{v}$. The quadrants were colored and annotated according to the division criteria. The red dot represents the location of Calgary, Canadá. In (b) taking all the nodes of Calgary, Canadá as origin, the dots are colored as a function of their exponent value and their color is quantified by the color bar in the center. The longest segments without a deceleration point (SWDP) are plotted in black. The probability density function of the $\beta's$ for each experiment is shown on the left of the color scale Figure (c) shows the mean correlation curve between time τ and the distance $\langle D \rangle$. The black traced line represents the exponent equal to 1.0. Figure (d) shows the distribution of SWDP sizes in number of nodes per frequency of occurrence.

Cali, Colombia

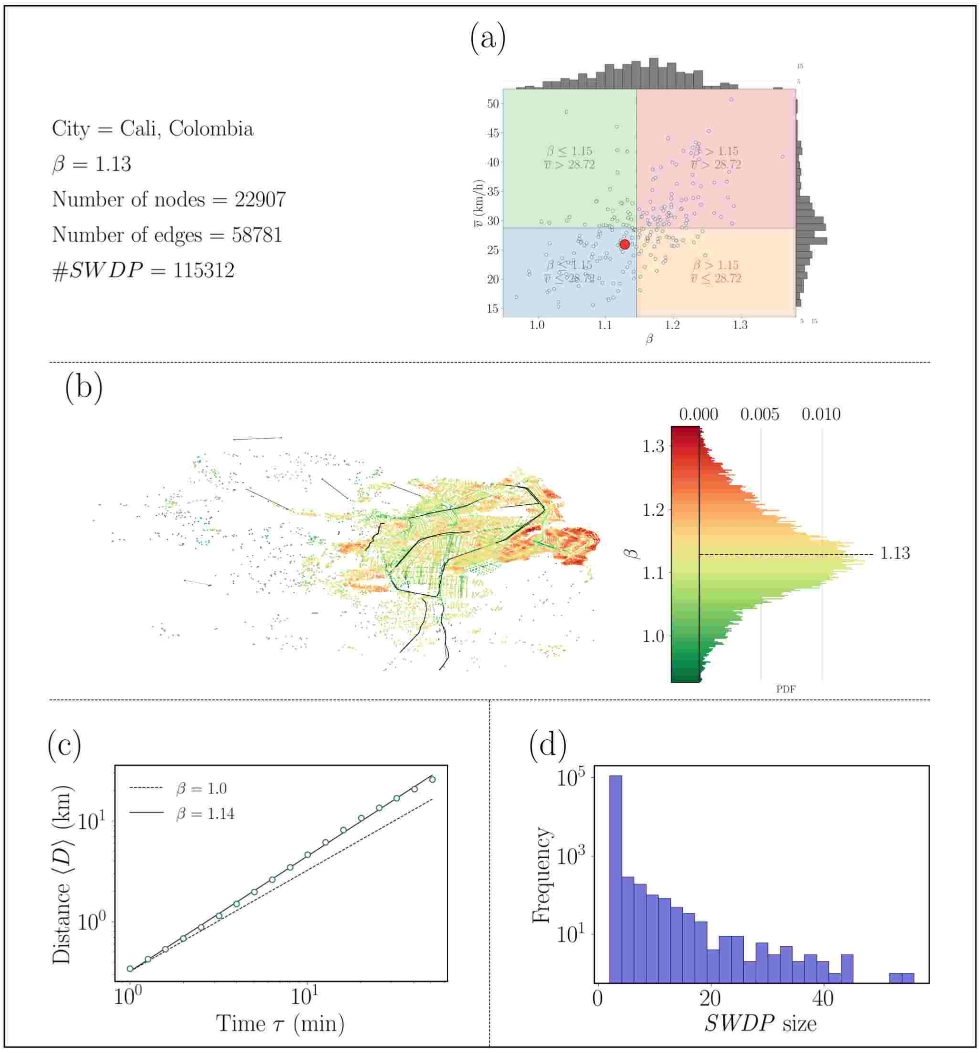

Fig. S32. Methodological sheet - Cali, Colombia. In (a) Each point represents a city, with mean exponent (β), on the x-axis, and mean speed $\overline{v}$ obtained in all trips made to calculate the exponent on the axis y. The histograms of the values of β and $\overline{v}$ are shown on the axes in the upper and right corners, respectively. The graph was segmented into four quadrants, in which the division is performed by the mean values of β and $\overline{v}$. The quadrants were colored and annotated according to the division criteria. The red dot represents the location of Cali, Colombia. In (b) taking all the nodes of Cali, Colombia as origin, the dots are colored as a function of their exponent value and their color is quantified by the color bar in the center. The longest segments without a deceleration point (SWDP) are plotted in black. The probability density function of the $\beta's$ for each experiment is shown on the left of the color scale Figure (c) shows the mean correlation curve between time τ and the distance $\langle D \rangle$. The black traced line represents the exponent equal to 1.0. Figure (d) shows the distribution of SWDP sizes in number of nodes per frequency of occurrence.

Cambridge, UK

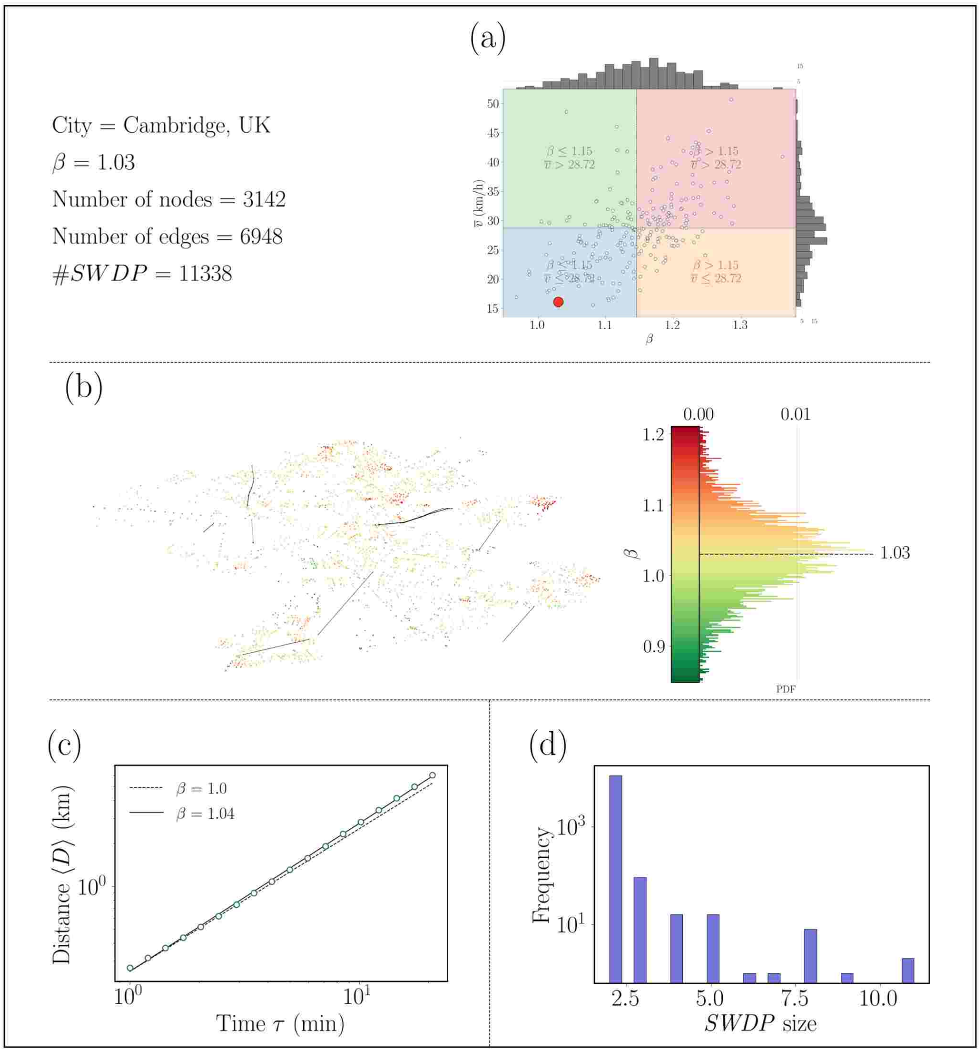

Fig. S33. Methodological sheet - Cambridge, UK. In (a) Each point represents a city, with mean exponent (β), on the x-axis, and mean speed $\overline{v}$ obtained in all trips made to calculate the exponent on the axis y. The histograms of the values of β and $\overline{v}$ are shown on the axes in the upper and right corners, respectively. The graph was segmented into four quadrants, in which the division is performed by the mean values of β and $\overline{v}$. The quadrants were colored and annotated according to the division criteria. The red dot represents the location of Cambridge, UK. In (b) taking all the nodes of Cambridge, UK as origin, the dots are colored as a function of their exponent value and their color is quantified by the color bar in the center. The longest segments without a deceleration point (SWDP) are plotted in black. The probability density function of the $\beta's$ for each experiment is shown on the left of the color scale Figure (c) shows the mean correlation curve between time τ and the distance $\langle D \rangle$. The black traced line represents the exponent equal to 1.0. Figure (d) shows the distribution of SWDP sizes in number of nodes per frequency of occurrence.

Campinas, Brasil

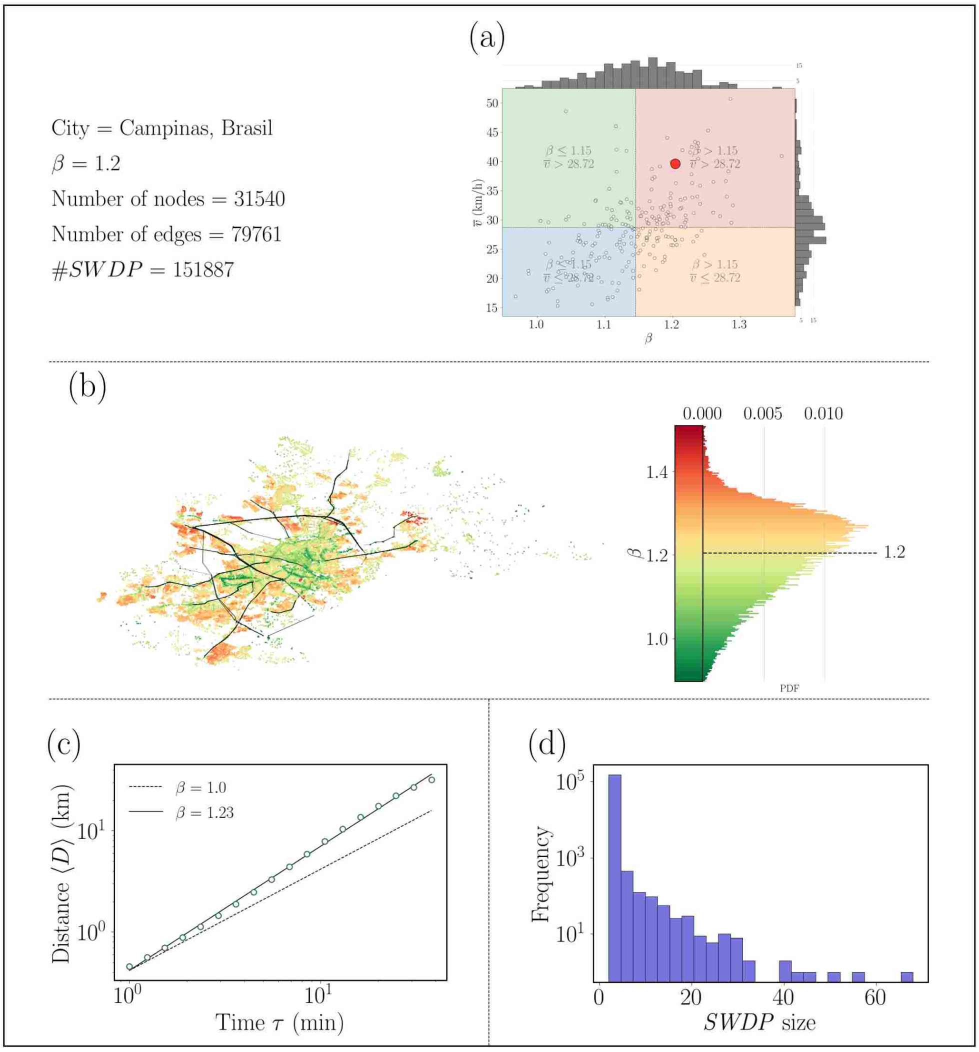

Fig. S34. Methodological sheet - Campinas, Brasil. In (a) Each point represents a city, with mean exponent (β), on the x-axis, and mean speed $\overline{v}$ obtained in all trips made to calculate the exponent on the axis y. The histograms of the values of β and $\overline{v}$ are shown on the axes in the upper and right corners, respectively. The graph was segmented into four quadrants, in which the division is performed by the mean values of β and $\overline{v}$. The quadrants were colored and annotated according to the division criteria. The red dot represents the location of Campinas, Brasil. In (b) taking all the nodes of Campinas, Brasil as origin, the dots are colored as a function of their exponent value and their color is quantified by the color bar in the center. The longest segments without a deceleration point (SWDP) are plotted in black. The probability density function of the $\beta's$ for each experiment is shown on the left of the color scale Figure (c) shows the mean correlation curve between time τ and the distance $\langle D \rangle$. The black traced line represents the exponent equal to 1.0. Figure (d) shows the distribution of SWDP sizes in number of nodes per frequency of occurrence.

Cancun, Mexico

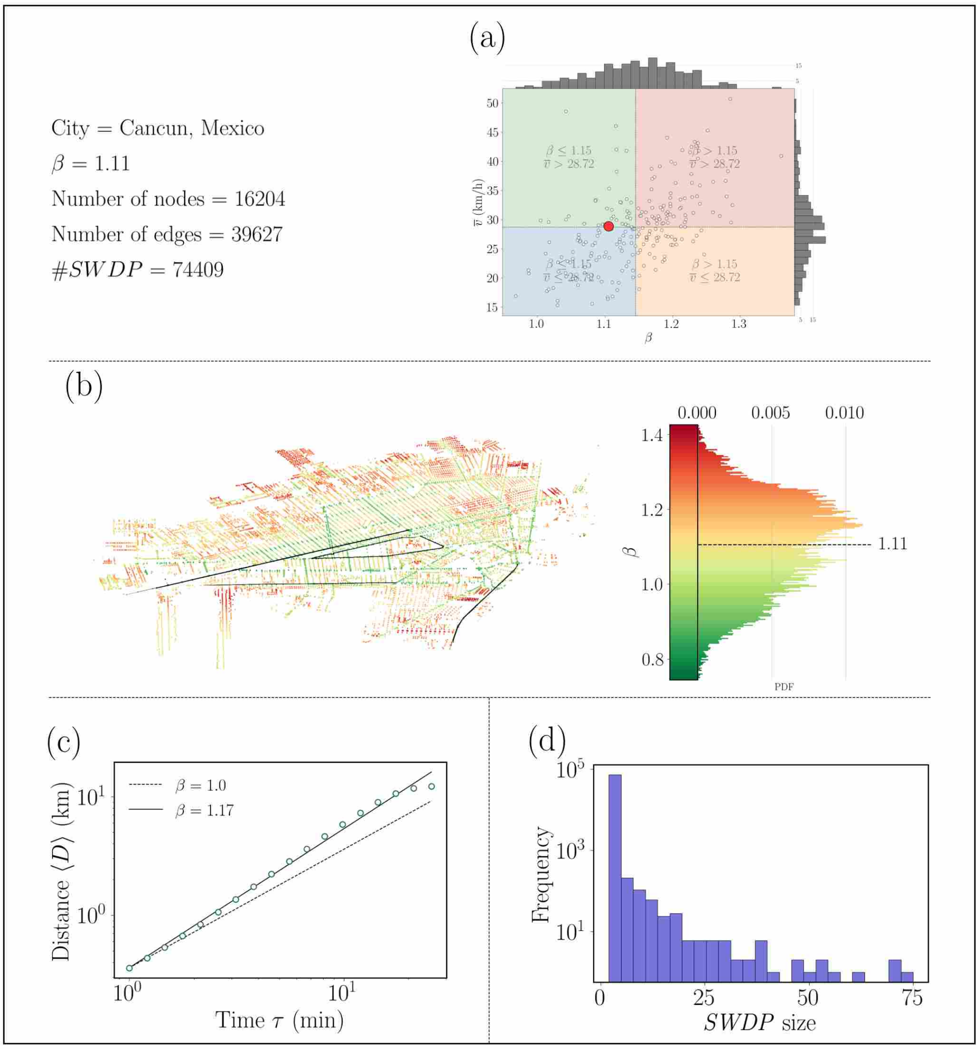

Fig. S35. Methodological sheet - Cancun, Mexico. In (a) Each point represents a city, with mean exponent (β), on the x-axis, and mean speed $\overline{v}$ obtained in all trips made to calculate the exponent on the axis y. The histograms of the values of β and $\overline{v}$ are shown on the axes in the upper and right corners, respectively. The graph was segmented into four quadrants, in which the division is performed by the mean values of β and $\overline{v}$. The quadrants were colored and annotated according to the division criteria. The red dot represents the location of Cancun, Mexico. In (b) taking all the nodes of Cancun, Mexico as origin, the dots are colored as a function of their exponent value and their color is quantified by the color bar in the center. The longest segments without a deceleration point (SWDP) are plotted in black. The probability density function of the $\beta's$ for each experiment is shown on the left of the color scale Figure (c) shows the mean correlation curve between time τ and the distance $\langle D \rangle$. The black traced line represents the exponent equal to 1.0. Figure (d) shows the distribution of SWDP sizes in number of nodes per frequency of occurrence.

Caracas, Venezuela

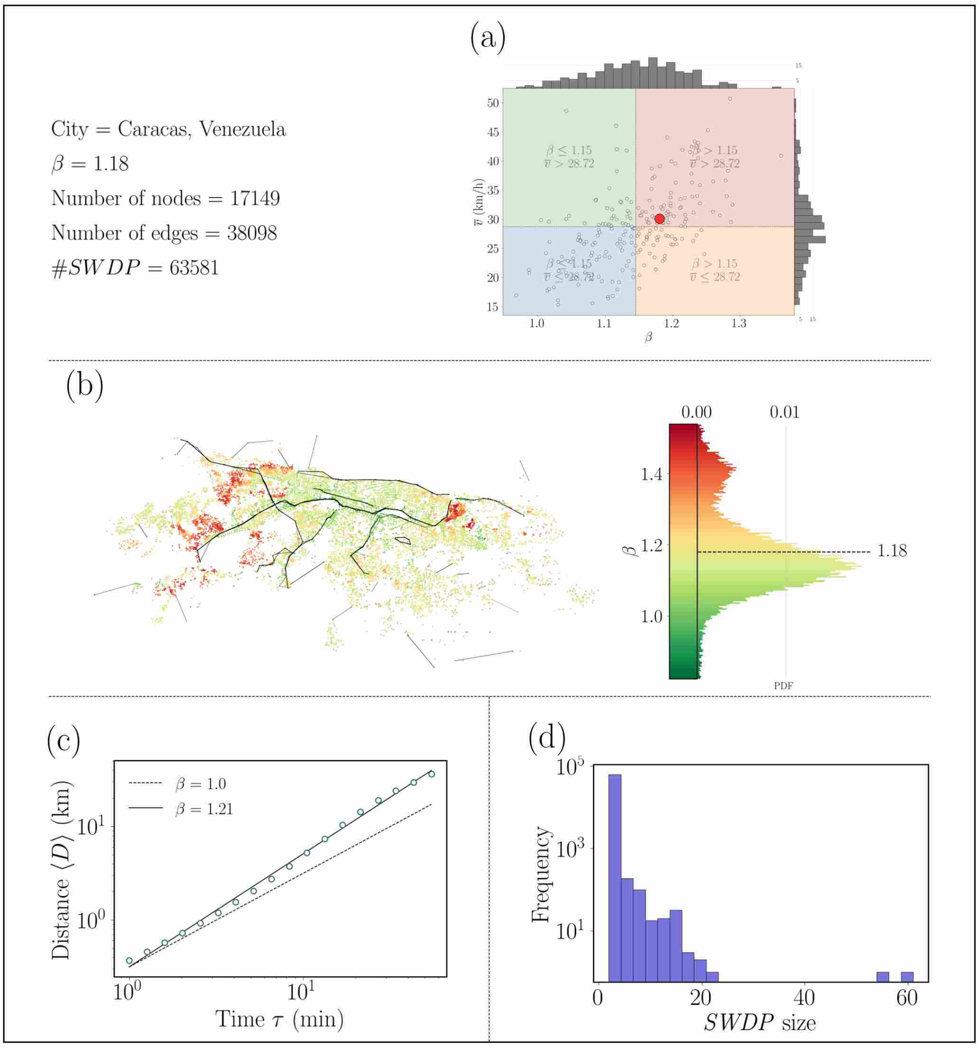

Fig. S36. Methodological sheet - Caracas, Venezuela. In (a) Each point represents a city, with mean exponent (β), on the x-axis, and mean speed $\overline{v}$ obtained in all trips made to calculate the exponent on the axis y. The histograms of the values of β and $\overline{v}$ are shown on the axes in the upper and right corners, respectively. The graph was segmented into four quadrants, in which the division is performed by the mean values of β and $\overline{v}$. The quadrants were colored and annotated according to the division criteria. The red dot represents the location of Caracas, Venezuela. In (b) taking all the nodes of Caracas, Venezuela as origin, the dots are colored as a function of their exponent value and their color is quantified by the color bar in the center. The longest segments without a deceleration point (SWDP) are plotted in black. The probability density function of the $\beta's$ for each experiment is shown on the left of the color scale Figure (c) shows the mean correlation curve between time τ and the distance $\langle D \rangle$. The black traced line represents the exponent equal to 1.0. Figure (d) shows the distribution of SWDP sizes in number of nodes per frequency of occurrence.

Cartagena, Colombia

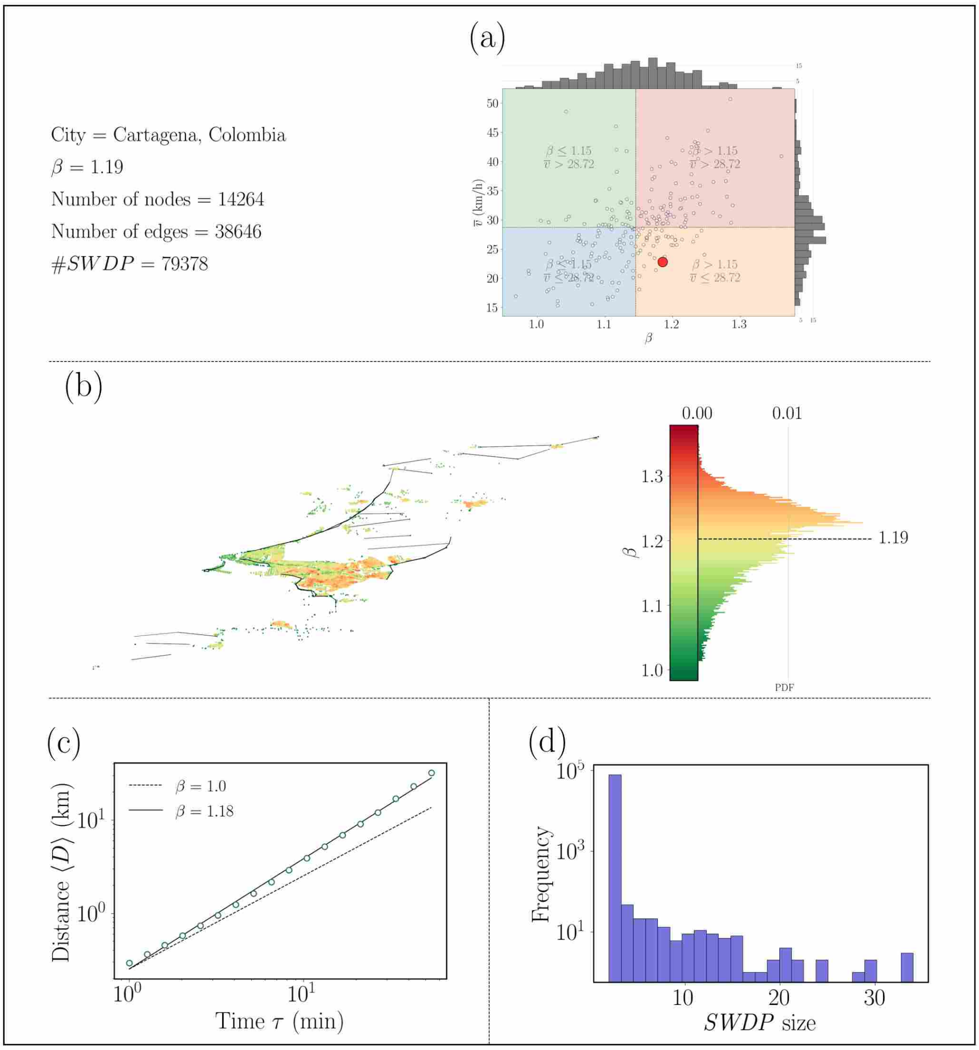

Fig. S37. Methodological sheet - Cartagena, Colombia. In (a) Each point represents a city, with mean exponent (β), on the x-axis, and mean speed $\overline{v}$ obtained in all trips made to calculate the exponent on the axis y. The histograms of the values of β and $\overline{v}$ are shown on the axes in the upper and right corners, respectively. The graph was segmented into four quadrants, in which the division is performed by the mean values of β and $\overline{v}$. The quadrants were colored and annotated according to the division criteria. The red dot represents the location of Cartagena, Colombia. In (b) taking all the nodes of Cartagena, Colombia as origin, the dots are colored as a function of their exponent value and their color is quantified by the color bar in the center. The longest segments without a deceleration point (SWDP) are plotted in black. The probability density function of the $\beta's$ for each experiment is shown on the left of the color scale Figure (c) shows the mean correlation curve between time τ and the distance $\langle D \rangle$. The black traced line represents the exponent equal to 1.0. Figure (d) shows the distribution of SWDP sizes in number of nodes per frequency of occurrence.

Casablanca, Morocco

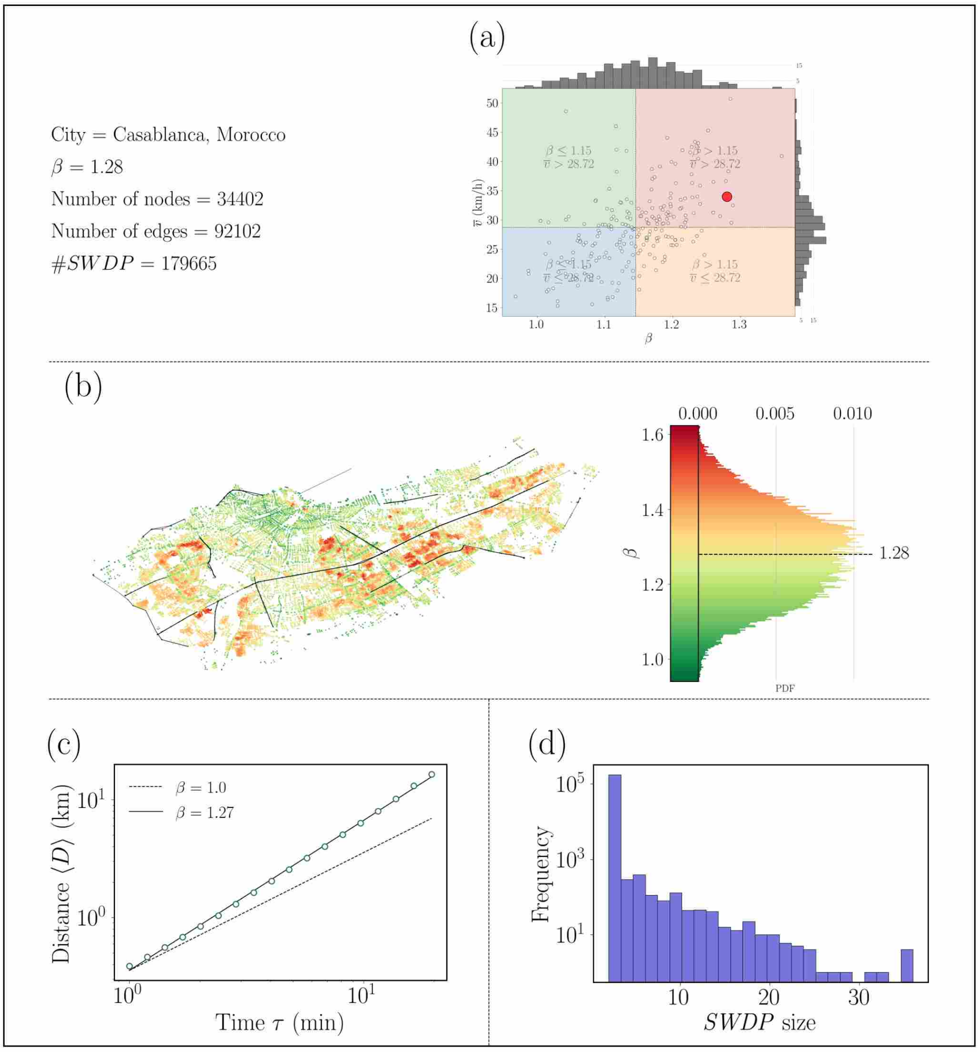

Fig. S38. Methodological sheet - Casablanca, Morocco. In (a) Each point represents a city, with mean exponent (β), on the x-axis, and mean speed $\overline{v}$ obtained in all trips made to calculate the exponent on the axis y. The histograms of the values of β and $\overline{v}$ are shown on the axes in the upper and right corners, respectively. The graph was segmented into four quadrants, in which the division is performed by the mean values of β and $\overline{v}$. The quadrants were colored and annotated according to the division criteria. The red dot represents the location of Casablanca, Morocco. In (b) taking all the nodes of Casablanca, Morocco as origin, the dots are colored as a function of their exponent value and their color is quantified by the color bar in the center. The longest segments without a deceleration point (SWDP) are plotted in black. The probability density function of the $\beta's$ for each experiment is shown on the left of the color scale Figure (c) shows the mean correlation curve between time τ and the distance $\langle D \rangle$. The black traced line represents the exponent equal to 1.0. Figure (d) shows the distribution of SWDP sizes in number of nodes per frequency of occurrence.

Cayenne, France

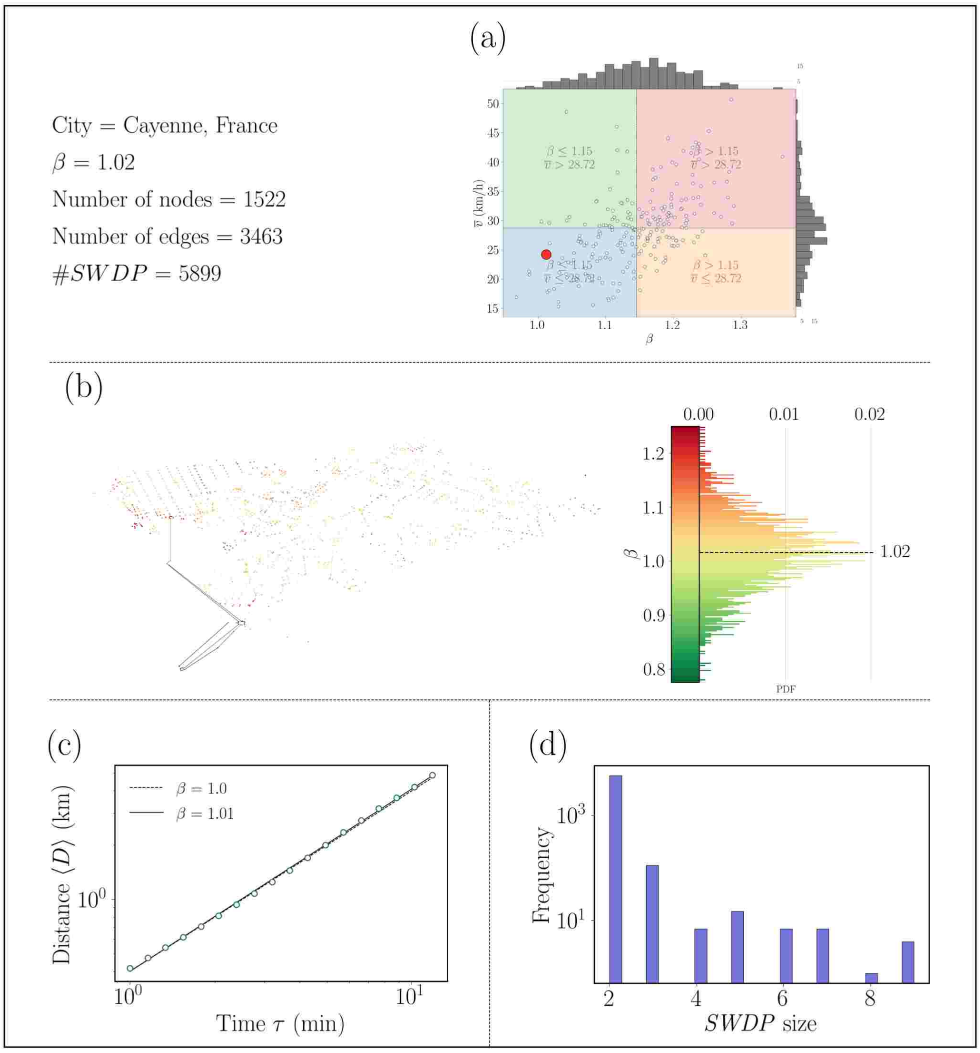

Fig. S39. Methodological sheet - Cayenne, France. In (a) Each point represents a city, with mean exponent (β), on the x-axis, and mean speed $\overline{v}$ obtained in all trips made to calculate the exponent on the axis y. The histograms of the values of β and $\overline{v}$ are shown on the axes in the upper and right corners, respectively. The graph was segmented into four quadrants, in which the division is performed by the mean values of β and $\overline{v}$. The quadrants were colored and annotated according to the division criteria. The red dot represents the location of Cayenne, France. In (b) taking all the nodes of Cayenne, France as origin, the dots are colored as a function of their exponent value and their color is quantified by the color bar in the center. The longest segments without a deceleration point (SWDP) are plotted in black. The probability density function of the $\beta's$ for each experiment is shown on the left of the color scale Figure (c) shows the mean correlation curve between time τ and the distance $\langle D \rangle$. The black traced line represents the exponent equal to 1.0. Figure (d) shows the distribution of SWDP sizes in number of nodes per frequency of occurrence.

Chandigarh, India

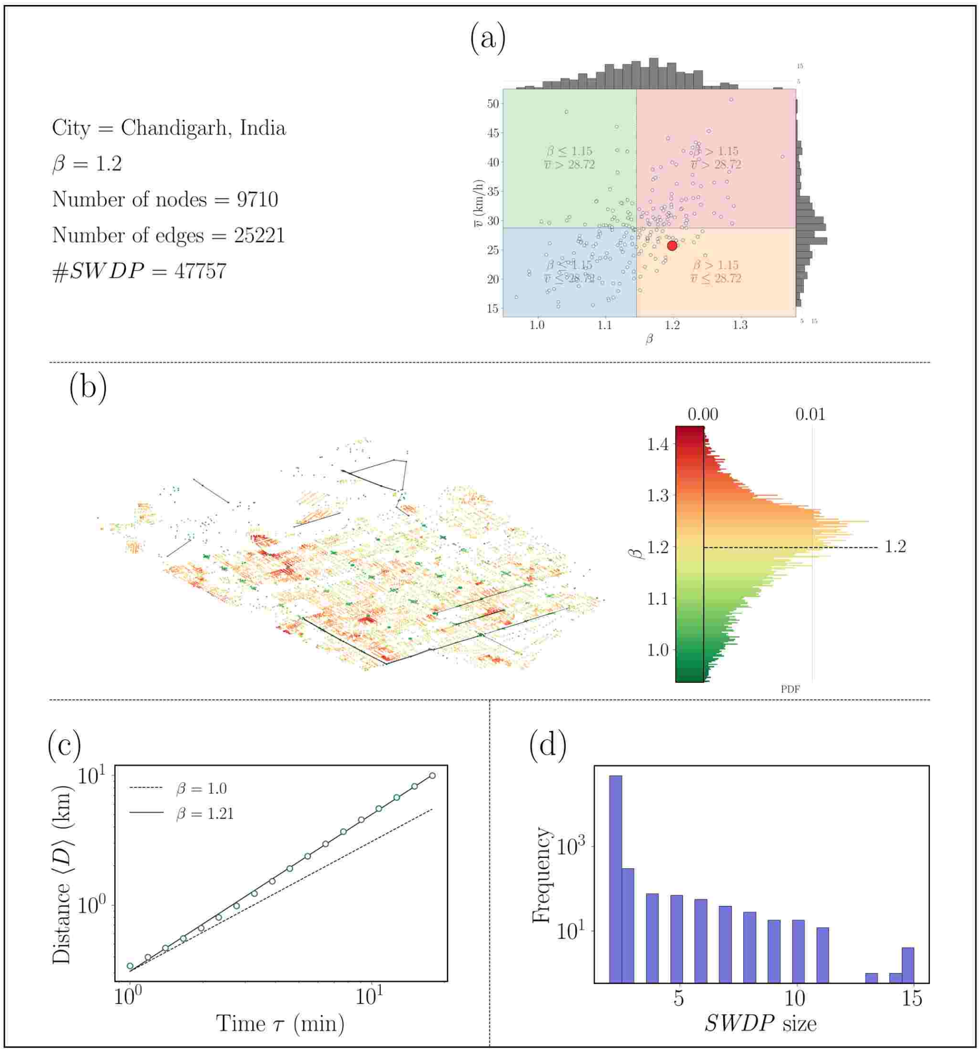

Fig. S40. Methodological sheet - Chandigarh, India. In (a) Each point represents a city, with mean exponent (β), on the x-axis, and mean speed $\overline{v}$ obtained in all trips made to calculate the exponent on the axis y. The histograms of the values of β and $\overline{v}$ are shown on the axes in the upper and right corners, respectively. The graph was segmented into four quadrants, in which the division is performed by the mean values of β and $\overline{v}$. The quadrants were colored and annotated according to the division criteria. The red dot represents the location of Chandigarh, India. In (b) taking all the nodes of Chandigarh, India as origin, the dots are colored as a function of their exponent value and their color is quantified by the color bar in the center. The longest segments without a deceleration point (SWDP) are plotted in black. The probability density function of the $\beta's$ for each experiment is shown on the left of the color scale Figure (c) shows the mean correlation curve between time τ and the distance $\langle D \rangle$. The black traced line represents the exponent equal to 1.0. Figure (d) shows the distribution of SWDP sizes in number of nodes per frequency of occurrence.

Charlotte, USA

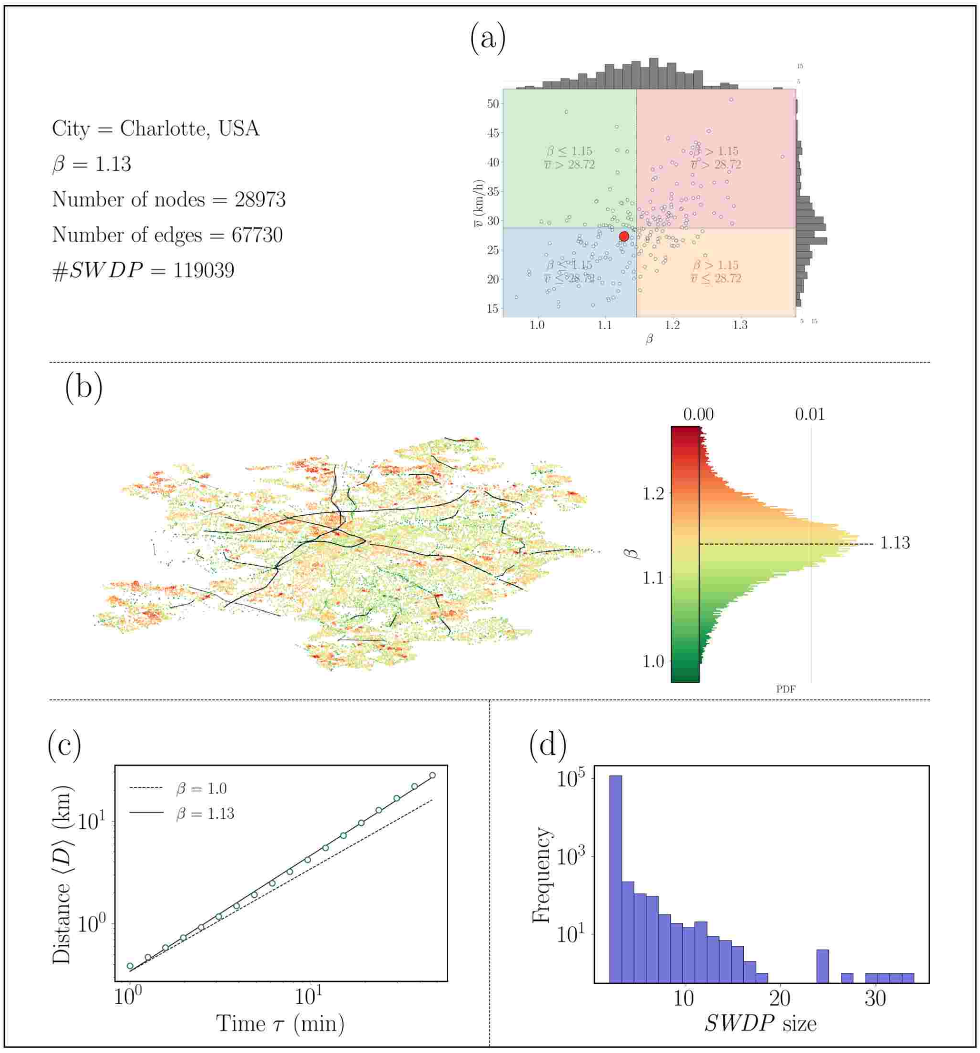

Fig. S41. Methodological sheet - Charlotte, USA. In (a) Each point represents a city, with mean exponent (β), on the x-axis, and mean speed $\overline{v}$ obtained in all trips made to calculate the exponent on the axis y. The histograms of the values of β and $\overline{v}$ are shown on the axes in the upper and right corners, respectively. The graph was segmented into four quadrants, in which the division is performed by the mean values of β and $\overline{v}$. The quadrants were colored and annotated according to the division criteria. The red dot represents the location of Charlotte, USA. In (b) taking all the nodes of Charlotte, USA as origin, the dots are colored as a function of their exponent value and their color is quantified by the color bar in the center. The longest segments without a deceleration point (SWDP) are plotted in black. The probability density function of the $\beta's$ for each experiment is shown on the left of the color scale Figure (c) shows the mean correlation curve between time τ and the distance $\langle D \rangle$. The black traced line represents the exponent equal to 1.0. Figure (d) shows the distribution of SWDP sizes in number of nodes per frequency of occurrence.

Chicago, USA

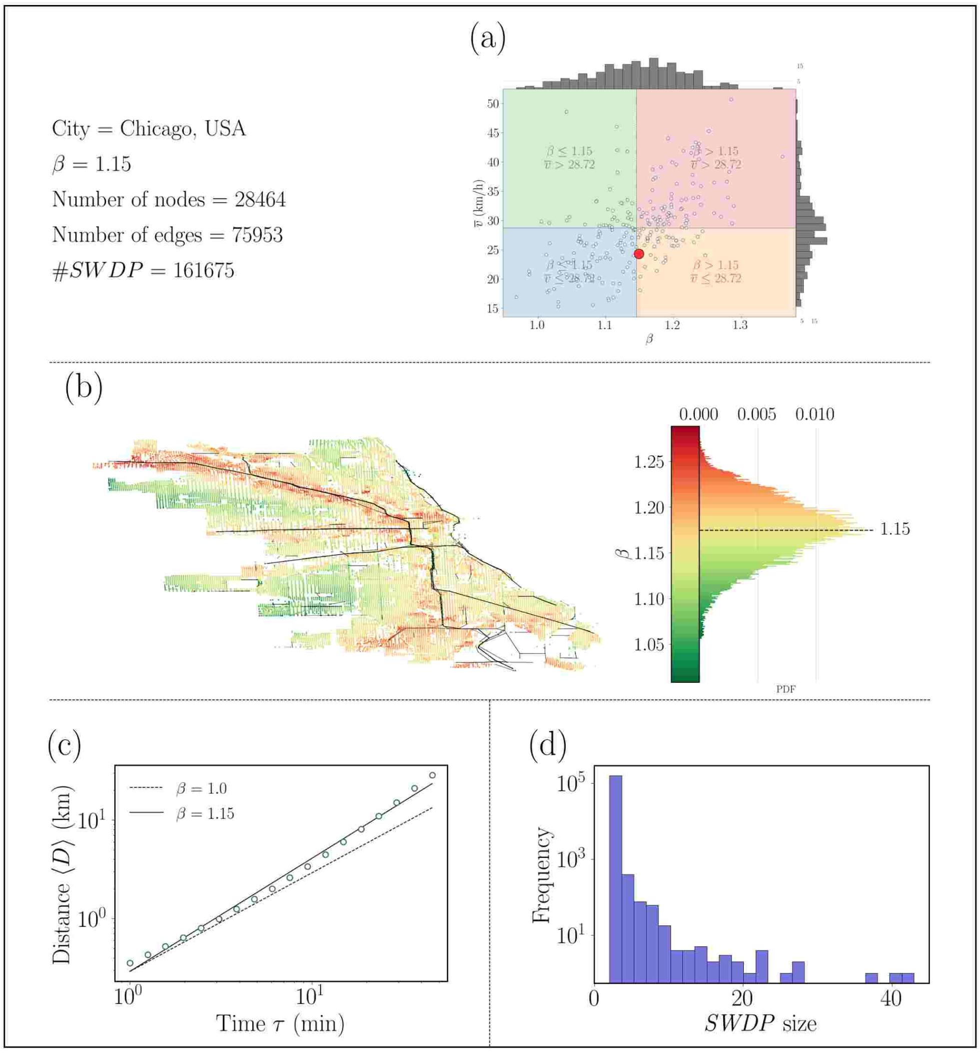

Fig. S42. Methodological sheet - Chicago, USA. In (a) Each point represents a city, with mean exponent (β), on the x-axis, and mean speed $\overline{v}$ obtained in all trips made to calculate the exponent on the axis y. The histograms of the values of β and $\overline{v}$ are shown on the axes in the upper and right corners, respectively. The graph was segmented into four quadrants, in which the division is performed by the mean values of β and $\overline{v}$. The quadrants were colored and annotated according to the division criteria. The red dot represents the location of Chicago, USA. In (b) taking all the nodes of Chicago, USA as origin, the dots are colored as a function of their exponent value and their color is quantified by the color bar in the center. The longest segments without a deceleration point (SWDP) are plotted in black. The probability density function of the $\beta' s$ for each experiment is shown on the left of the color scale Figure (c) shows the mean correlation curve between time τ and the distance $\langle D \rangle$. The black traced line represents the exponent equal to 1.0. Figure (d) shows the distribution of SWDP sizes in number of nodes per frequency of occurrence.

Christchurch, New Zealand

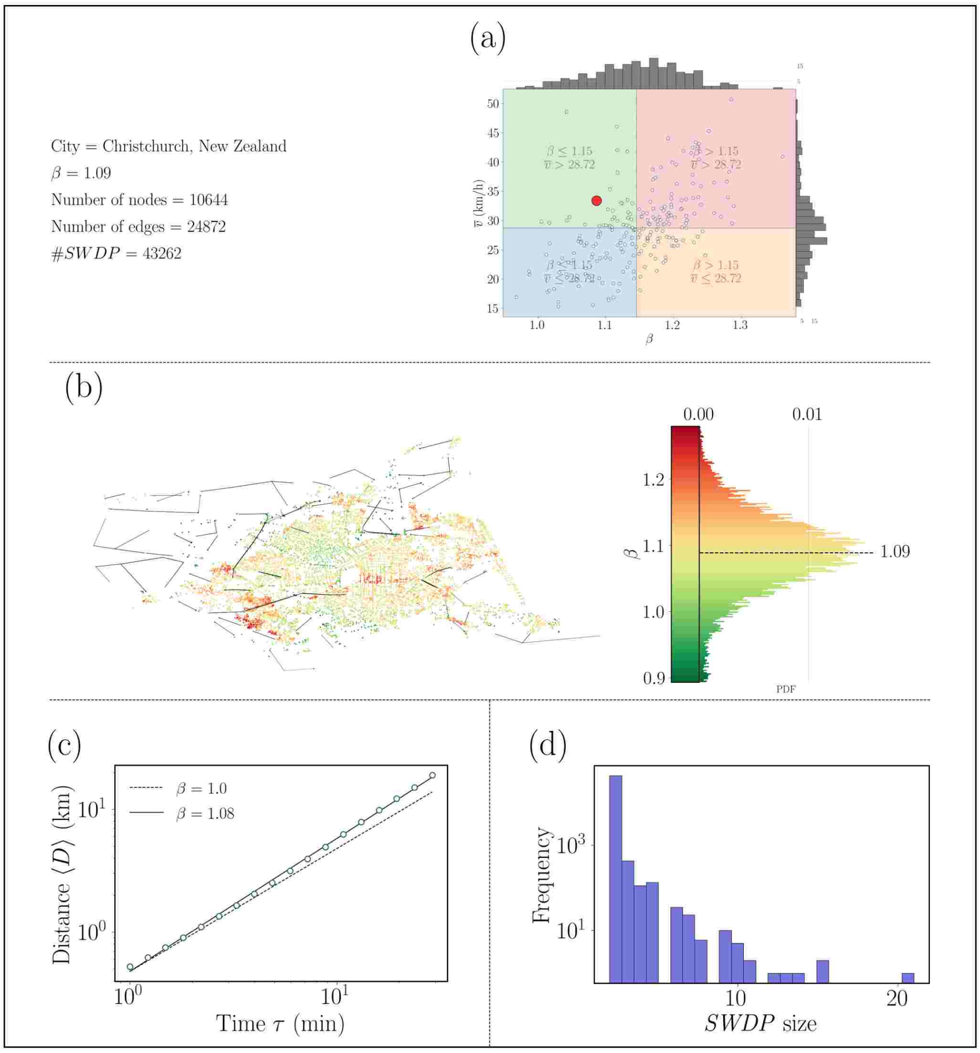

Fig. S43. Methodological sheet - Christchurch, New Zealand. In (a) Each point represents a city, with mean exponent (β), on the x-axis, and mean speed $\overline{v}$ obtained in all trips made to calculate the exponent on the axis y. The histograms of the values of β and $\overline{v}$ are shown on the axes in the upper and right corners, respectively. The graph was segmented into four quadrants, in which the division is performed by the mean values of β and $\overline{v}$. The quadrants were colored and annotated according to the division criteria. The red dot represents the location of Christchurch, New Zealand. In (b) taking all the nodes of Christchurch, New Zealand as origin, the dots are colored as a function of their exponent value and their color is quantified by the color bar in the center. The longest segments without a deceleration point (SWDP) are plotted in black. The probability density function of the $\beta's$ for each experiment is shown on the left of the color scale Figure (c) shows the mean correlation curve between time τ and the distance $\langle D \rangle$. The black traced line represents the exponent equal to 1.0. Figure (d) shows the distribution of SWDP sizes in number of nodes per frequency of occurrence.

Ciudad del Este, Paraguay

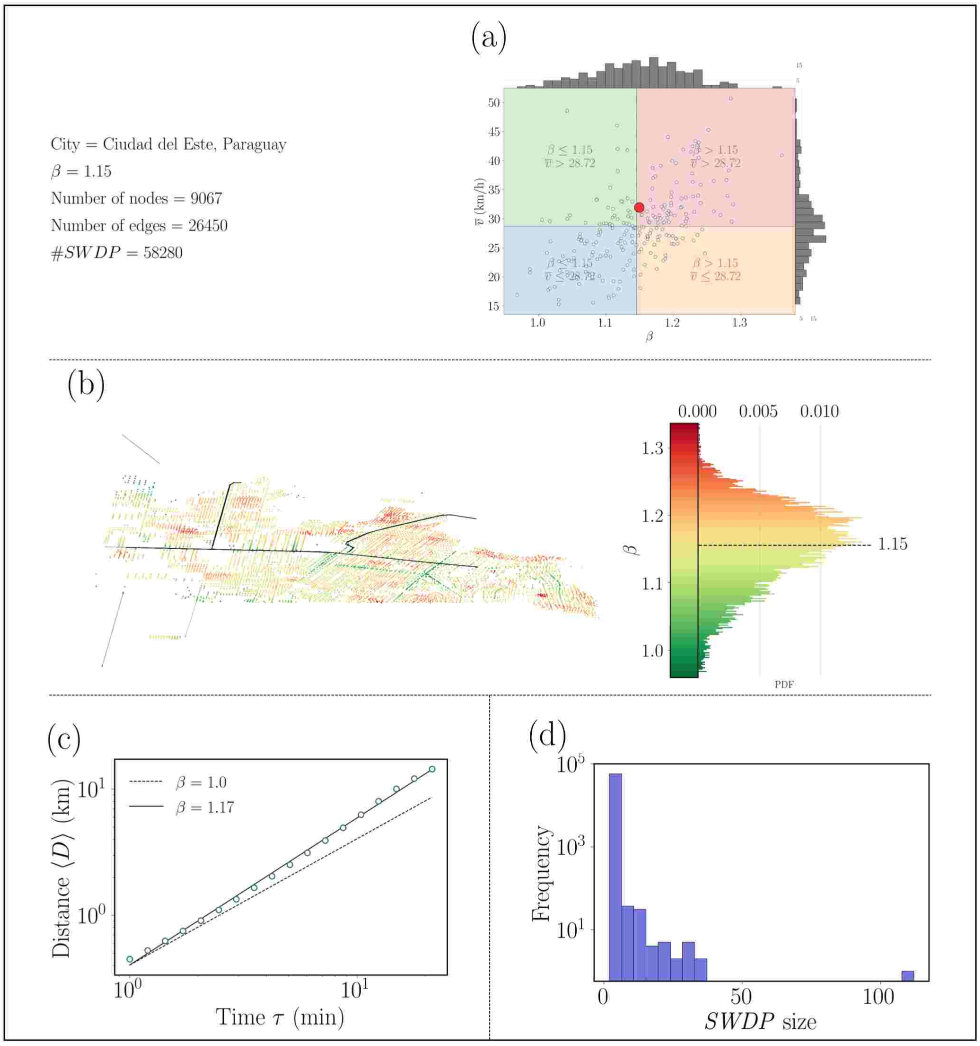

Fig. S44. Methodological sheet - Ciudad del Este, Paraguay. In (a) Each point represents a city, with mean exponent (β), on the x-axis, and mean speed $\overline{v}$ obtained in all trips made to calculate the exponent on the axis y. The histograms of the values of β and $\overline{v}$ are shown on the axes in the upper and right corners, respectively. The graph was segmented into four quadrants, in which the division is performed by the mean values of β and $\overline{v}$. The quadrants were colored and annotated according to the division criteria. The red dot represents the location of Ciudad del Este, Paraguay. In (b) taking all the nodes of Ciudad del Este, Paraguay as origin, the dots are colored as a function of their exponent value and their color is quantified by the color bar in the center. The longest segments without a deceleration point (SWDP) are plotted in black. The probability density function of the $\beta's$ for each experiment is shown on the left of the color scale Figure (c) shows the mean correlation curve between time τ and the distance $\langle D \rangle$. The black traced line represents the exponent equal to 1.0. Figure (d) shows the distribution of SWDP sizes in number of nodes per frequency of occurrence.

Cologne, Germany

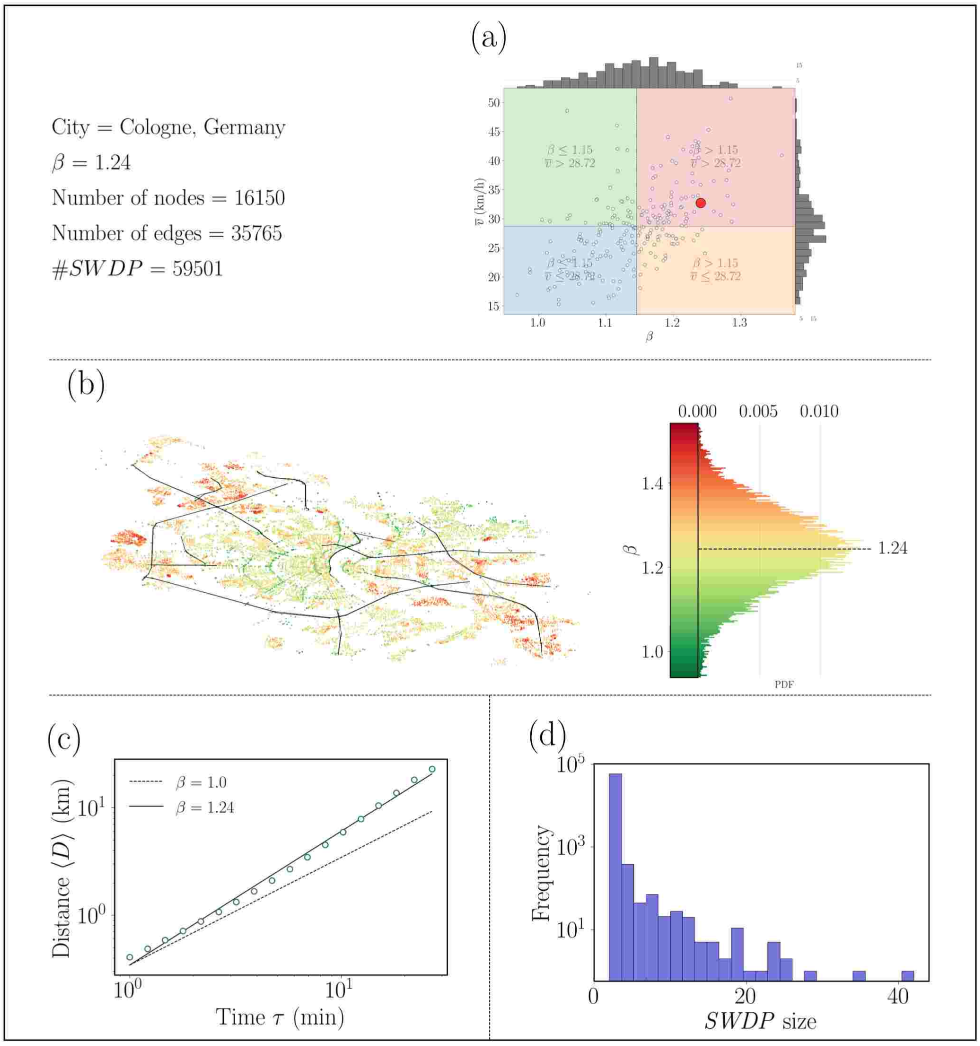

Fig. S45. Methodological sheet - Cologne, Germany. In (a) Each point represents a city, with mean exponent (β), on the x-axis, and mean speed $\overline{v}$ obtained in all trips made to calculate the exponent on the axis y. The histograms of the values of β and $\overline{v}$ are shown on the axes in the upper and right corners, respectively. The graph was segmented into four quadrants, in which the division is performed by the mean values of β and $\overline{v}$. The quadrants were colored and annotated according to the division criteria. The red dot represents the location of Cologne, Germany. In (b) taking all the nodes of Cologne, Germany as origin, the dots are colored as a function of their exponent value and their color is quantified by the color bar in the center. The longest segments without a deceleration point (SWDP) are plotted in black. The probability density function of the $\beta's$ for each experiment is shown on the left of the color scale Figure (c) shows the mean correlation curve between time τ and the distance $\langle D \rangle$. The black traced line represents the exponent equal to 1.0. Figure (d) shows the distribution of SWDP sizes in number of nodes per frequency of occurrence.

Columbus, USA

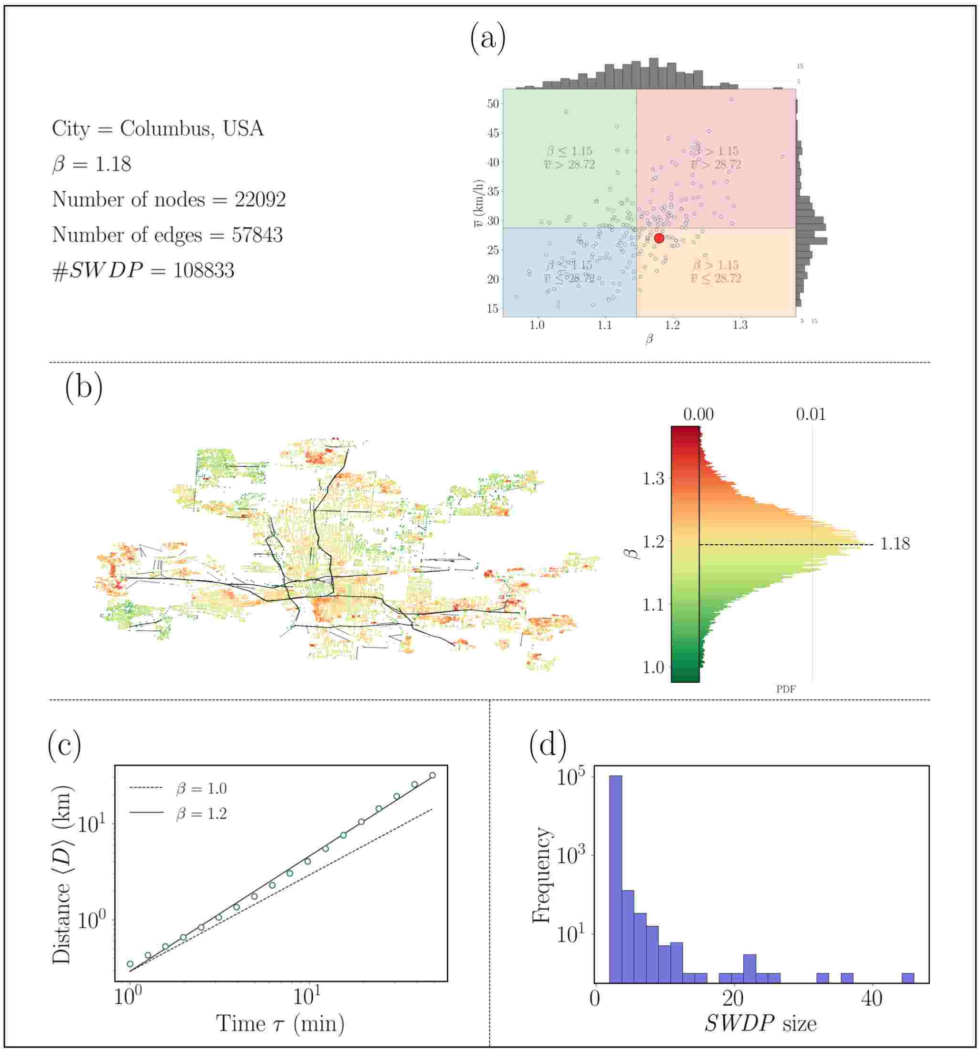

Fig. S46. Methodological sheet - Columbus, USA. In (a) Each point represents a city, with mean exponent (β), on the x-axis, and mean speed $\overline{v}$ obtained in all trips made to calculate the exponent on the axis y. The histograms of the values of β and $\overline{v}$ are shown on the axes in the upper and right corners, respectively. The graph was segmented into four quadrants, in which the division is performed by the mean values of β and $\overline{v}$. The quadrants were colored and annotated according to the division criteria. The red dot represents the location of Columbus, USA. In (b) taking all the nodes of Columbus, USA as origin, the dots are colored as a function of their exponent value and their color is quantified by the color bar in the center. The longest segments without a deceleration point (SWDP) are plotted in black. The probability density function of the $\beta's$ for each experiment is shown on the left of the color scale Figure (c) shows the mean correlation curve between time τ and the distance $\langle D\rangle$. The black traced line represents the exponent equal to 1.0. Figure (d) shows the distribution of SWDP sizes in number of nodes per frequency of occurrence.

Conakry, Guinea

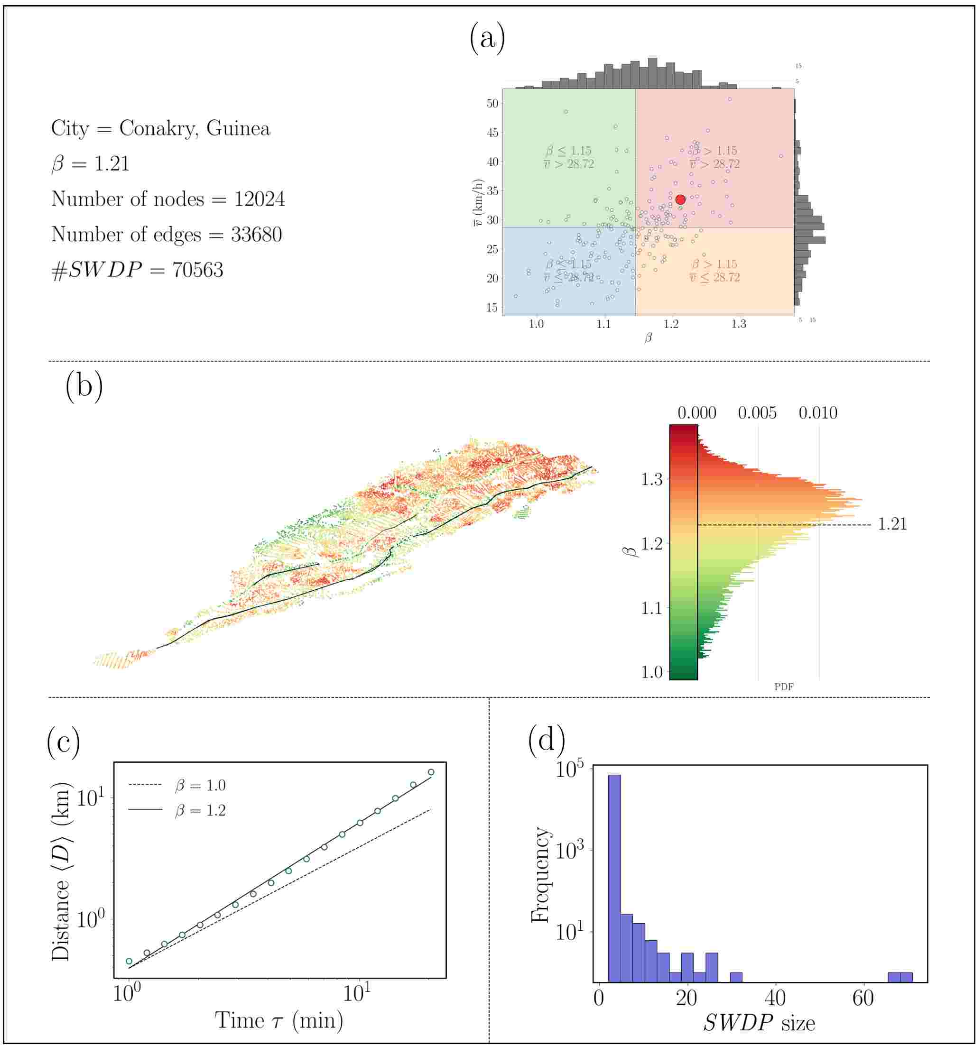

Fig. S47. Methodological sheet - Conakry, Guinea. In (a) Each point represents a city, with mean exponent (β), on the x-axis, and mean speed $\overline{v}$ obtained in all trips made to calculate the exponent on the axis y. The histograms of the values of β and $\overline{v}$ are shown on the axes in the upper and right corners, respectively. The graph was segmented into four quadrants, in which the division is performed by the mean values of β and $\overline{v}$. The quadrants were colored and annotated according to the division criteria. The red dot represents the location of Conakry, Guinea. In (b) taking all the nodes of Conakry, Guinea as origin, the dots are colored as a function of their exponent value and their color is quantified by the color bar in the center. The longest segments without a deceleration point (SWDP) are plotted in black. The probability density function of the $\beta's$ for each experiment is shown on the left of the color scale Figure (c) shows the mean correlation curve between time τ and the distance $\langle D \rangle$. The black traced line represents the exponent equal to 1.0. Figure (d) shows the distribution of SWDP sizes in number of nodes per frequency of occurrence.

Copenhagen, Denmark

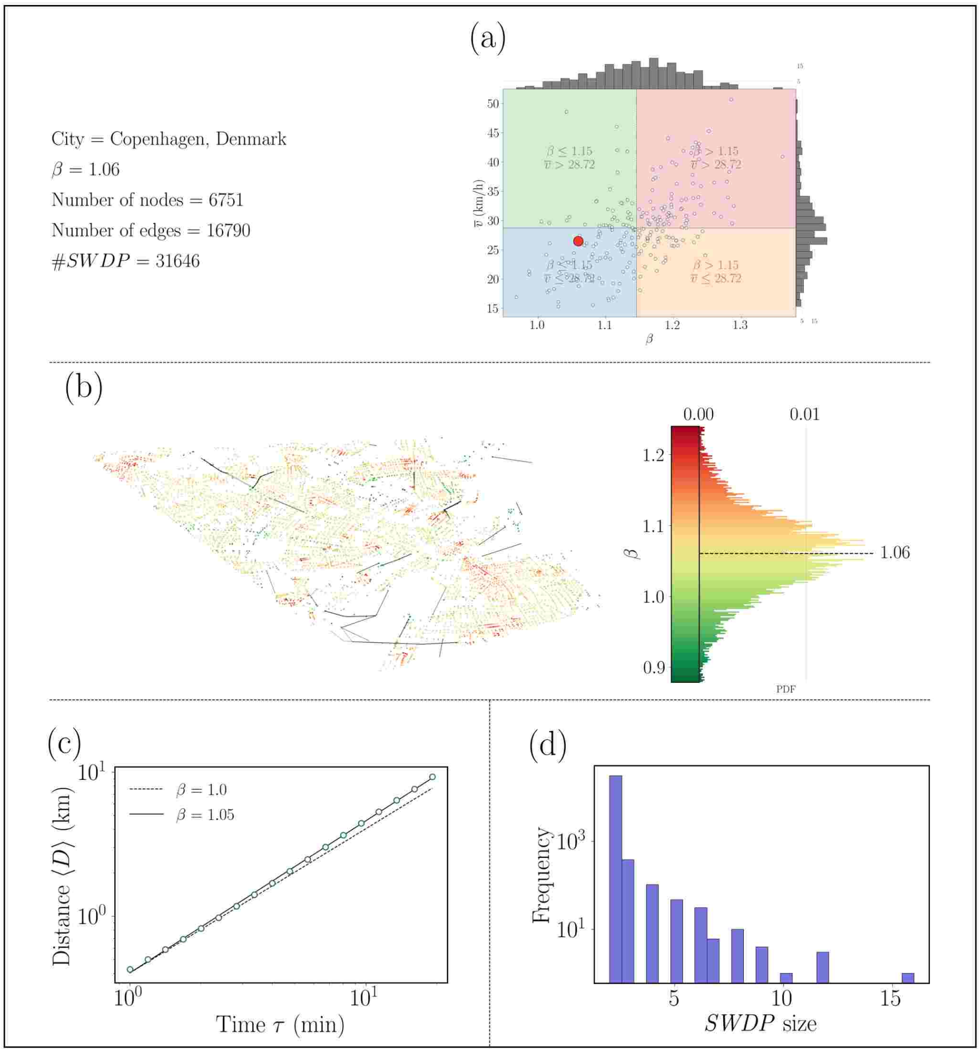

Fig. S48. Methodological sheet - Copenhagen, Denmark. In (a) Each point represents a city, with mean exponent (β), on the x-axis, and mean speed $\overline{v}$ obtained in all trips made to calculate the exponent on the axis y. The histograms of the values of β and $\overline{v}$ are shown on the axes in the upper and right corners, respectively. The graph was segmented into four quadrants, in which the division is performed by the mean values of β and $\overline{v}$. The quadrants were colored and annotated according to the division criteria. The red dot represents the location of Copenhagen, Denmark. In (b) taking all the nodes of Copenhagen, Denmark as origin, the dots are colored as a function of their exponent value and their color is quantified by the color bar in the center. The longest segments without a deceleration point (SWDP) are plotted in black. The probability density function of the $\beta's$ for each experiment is shown on the left of the color scale Figure (c) shows the mean correlation curve between time τ and the distance $\langle D \rangle$. The black traced line represents the exponent equal to 1.0. Figure (d) shows the distribution of SWDP sizes in number of nodes per frequency of occurrence.

Cork, Ireland

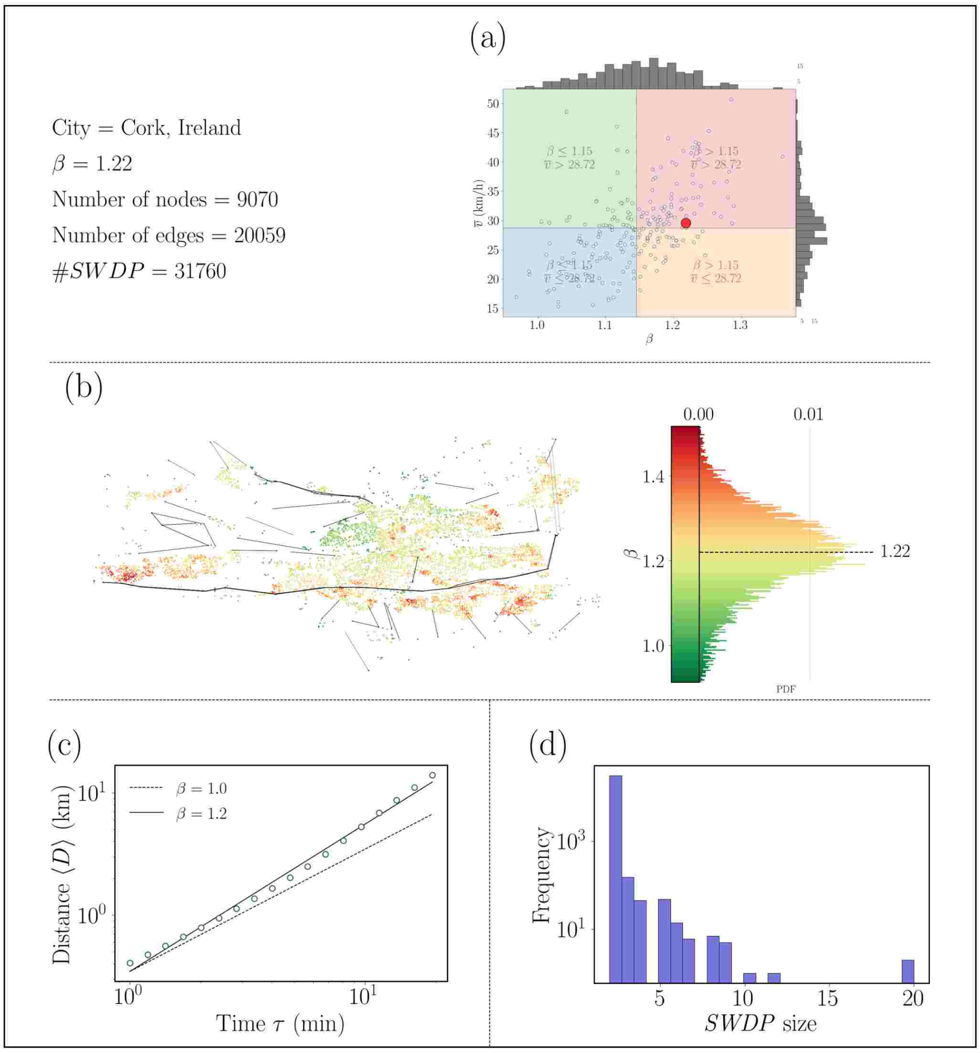

Fig. S49. Methodological sheet - Cork, Ireland. In (a) Each point represents a city, with mean exponent (β), on the x-axis, and mean speed $\overline{v}$ obtained in all trips made to calculate the exponent on the axis y. The histograms of the values of β and $\overline{v}$ are shown on the axes in the upper and right corners, respectively. The graph was segmented into four quadrants, in which the division is performed by the mean values of β and $\overline{v}$. The quadrants were colored and annotated according to the division criteria. The red dot represents the location of Cork, Ireland. In (b) taking all the nodes of Cork, Ireland as origin, the dots are colored as a function of their exponent value and their color is quantified by the color bar in the center. The longest segments without a deceleration point (SWDP) are plotted in black. The probability density function of the $\beta's$ for each experiment is shown on the left of the color scale Figure (c) shows the mean correlation curve between time τ and the distance $\langle D\rangle$. The black traced line represents the exponent equal to 1.0. Figure (d) shows the distribution of SWDP sizes in number of nodes per frequency of occurrence.

Curitiba, Brasil

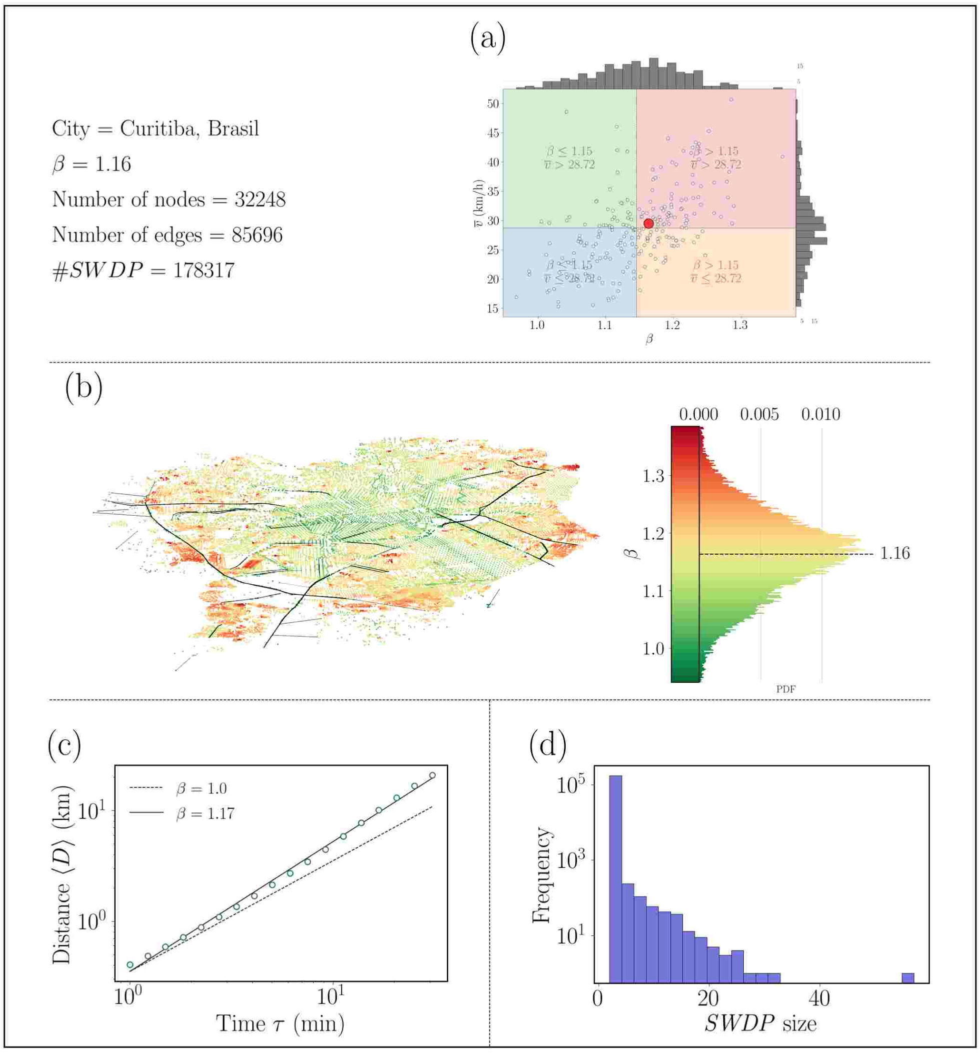

Fig. S50. Methodological sheet - Curitiba, Brasil. In (a) Each point represents a city, with mean exponent (β), on the x-axis, and mean speed $\overline{v}$ obtained in all trips made to calculate the exponent on the axis y. The histograms of the values of β and $\overline{v}$ are shown on the axes in the upper and right corners, respectively. The graph was segmented into four quadrants, in which the division is performed by the mean values of β and $\overline{v}$. The quadrants were colored and annotated according to the division criteria. The red dot represents the location of Curitiba, Brasil. In (b) taking all the nodes of Curitiba, Brasil as origin, the dots are colored as a function of their exponent value and their color is quantified by the color bar in the center. The longest segments without a deceleration point (SWDP) are plotted in black. The probability density function of the $\beta's$ for each experiment is shown on the left of the color scale Figure (c) shows the mean correlation curve between time τ and the distance $\langle D \rangle$. The black traced line represents the exponent equal to 1.0. Figure (d) shows the distribution of SWDP sizes in number of nodes per frequency of occurrence.

Dallas, USA

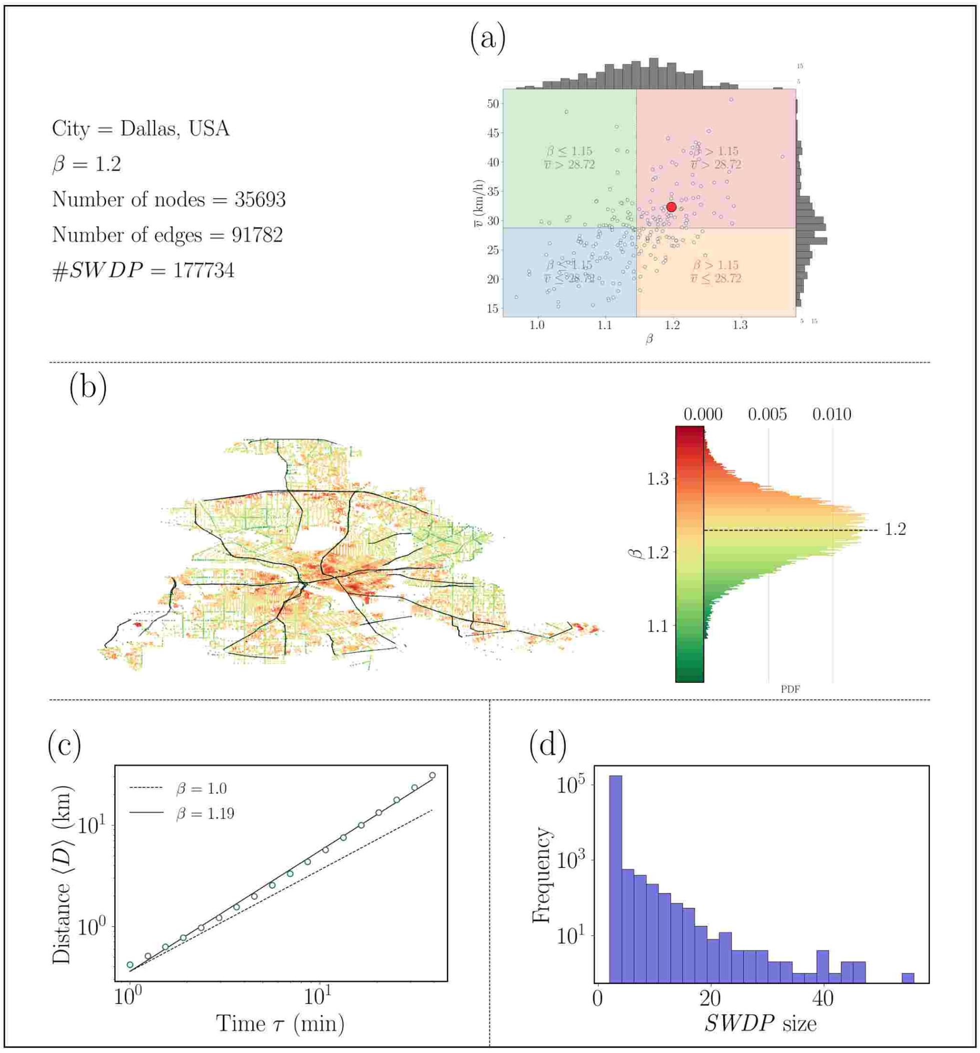

Fig. S51. Methodological sheet - Dallas, USA. In (a) Each point represents a city, with mean exponent (β), on the x-axis, and mean speed $\overline{v}$ obtained in all trips made to calculate the exponent on the axis y. The histograms of the values of β and $\overline{v}$ are shown on the axes in the upper and right corners, respectively. The graph was segmented into four quadrants, in which the division is performed by the mean values of β and $\overline{v}$. The quadrants were colored and annotated according to the division criteria. The red dot represents the location of Dallas, USA. In (b) taking all the nodes of Dallas, USA as origin, the dots are colored as a function of their exponent value and their color is quantified by the color bar in the center. The longest segments without a deceleration point (SWDP) are plotted in black. The probability density function of the $\beta's$ for each experiment is shown on the left of the color scale Figure (c) shows the mean correlation curve between time τ and the distance $\langle D \rangle$. The black traced line represents the exponent equal to 1.0. Figure (d) shows the distribution of SWDP sizes in number of nodes per frequency of occurrence.

Dar es Salaam, Tanzania

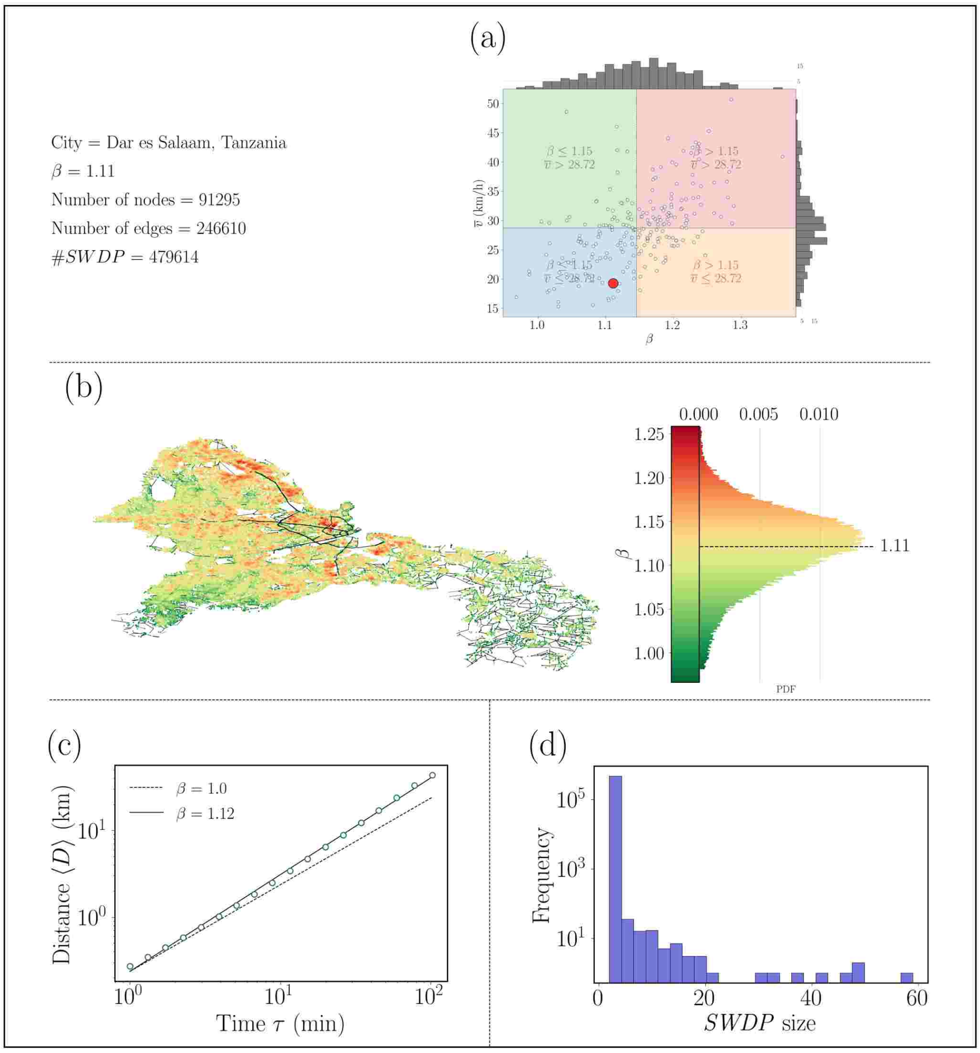

Fig. S52. Methodological sheet - Dar es Salaam, Tanzania. In (a) Each point represents a city, with mean exponent (β), on the x-axis, and mean speed $\overline{v}$ obtained in all trips made to calculate the exponent on the axis y. The histograms of the values of β and $\overline{v}$ are shown on the axes in the upper and right corners, respectively. The graph was segmented into four quadrants, in which the division is performed by the mean values of β and $\overline{v}$. The quadrants were colored and annotated according to the division criteria. The red dot represents the location of Dar es Salaam, Tanzania. In (b) taking all the nodes of Dar es Salaam, Tanzania as origin, the dots are colored as a function of their exponent value and their color is quantified by the color bar in the center. The longest segments without a deceleration point (SWDP) are plotted in black. The probability density function of the $\beta's$ for each experiment is shown on the left of the color scale Figure (c) shows the mean correlation curve between time τ and the distance $\langle D \rangle$. The black traced line represents the exponent equal to 1.0. Figure (d) shows the distribution of SWDP sizes in number of nodes per frequency of occurrence.

Denver,USA

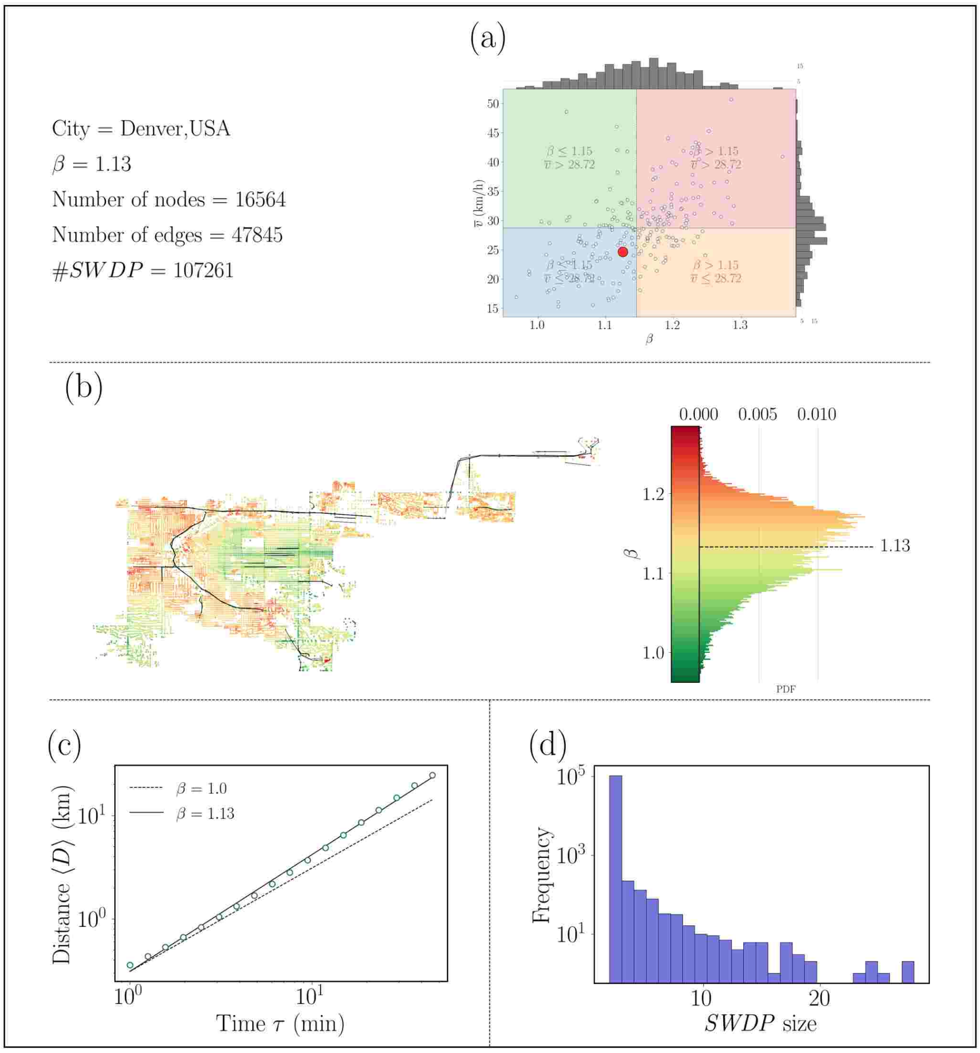

Fig. S53. Methodological sheet - Denver,USA. In (a) Each point represents a city, with mean exponent (β), on the x-axis, and mean speed $\overline{v}$ obtained in all trips made to calculate the exponent on the axis y. The histograms of the values of β and $\overline{v}$ are shown on the axes in the upper and right corners, respectively. The graph was segmented into four quadrants, in which the division is performed by the mean values of β and $\overline{v}$. The quadrants were colored and annotated according to the division criteria. The red dot represents the location of Denver,USA. In (b) taking all the nodes of Denver,USA as origin, the dots are colored as a function of their exponent value and their color is quantified by the color bar in the center. The longest segments without a deceleration point (SWDP) are plotted in black. The probability density function of the $\beta's$ for each experiment is shown on the left of the color scale Figure (c) shows the mean correlation curve between time τ and the distance $\langle D \rangle$. The black traced line represents the exponent equal to 1.0. Figure (d) shows the distribution of SWDP sizes in number of nodes per frequency of occurrence.

Detroit, USA

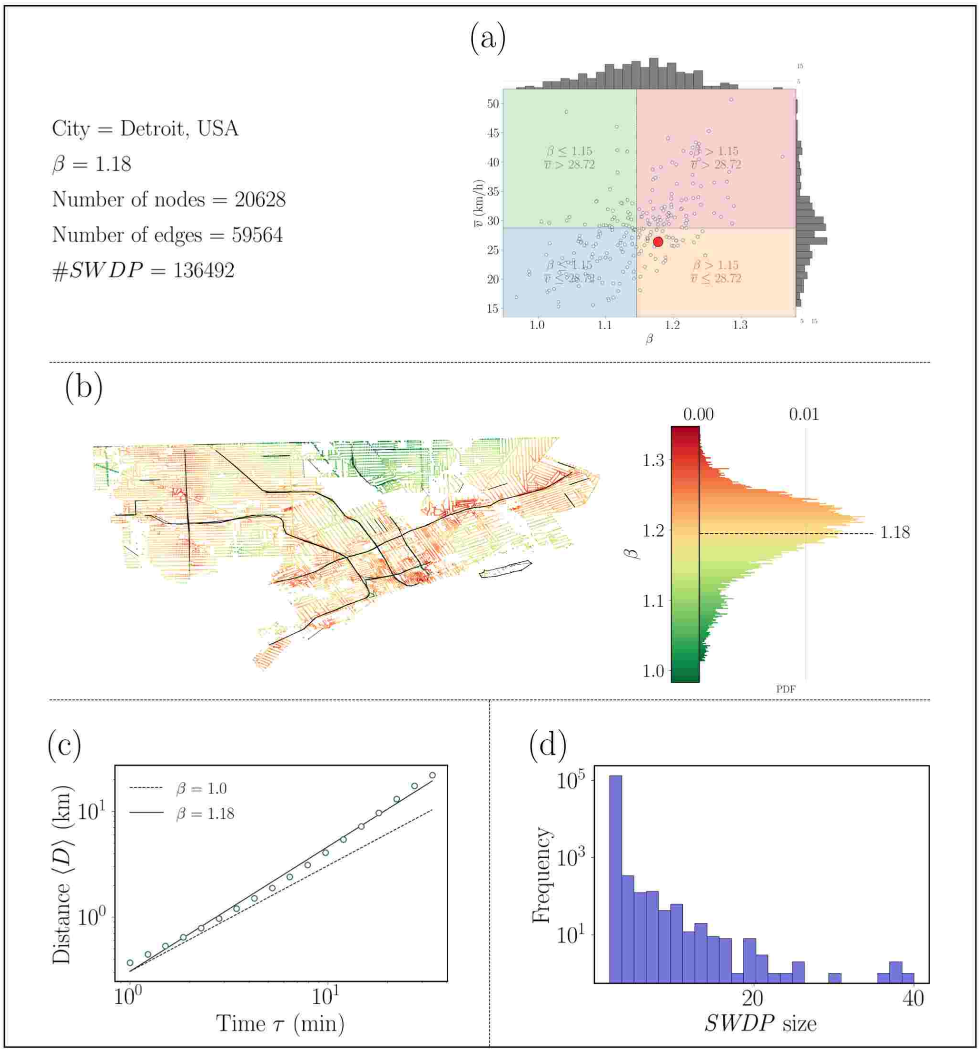

Fig. S54. Methodological sheet - Detroit, USA. In (a) Each point represents a city, with mean exponent (β), on the x-axis, and mean speed $\overline{v}$ obtained in all trips made to calculate the exponent on the axis y. The histograms of the values of β and $\overline{v}$ are shown on the axes in the upper and right corners, respectively. The graph was segmented into four quadrants, in which the division is performed by the mean values of β and $\overline{v}$. The quadrants were colored and annotated according to the division criteria. The red dot represents the location of Detroit, USA. In (b) taking all the nodes of Detroit, USA as origin, the dots are colored as a function of their exponent value and their color is quantified by the color bar in the center. The longest segments without a deceleration point (SWDP) are plotted in black. The probability density function of the $\beta's$ for each experiment is shown on the left of the color scale Figure (c) shows the mean correlation curve between time τ and the distance $\langle D \rangle$. The black traced line represents the exponent equal to 1.0. Figure (d) shows the distribution of SWDP sizes in number of nodes per frequency of occurrence.

Dresden, Germany

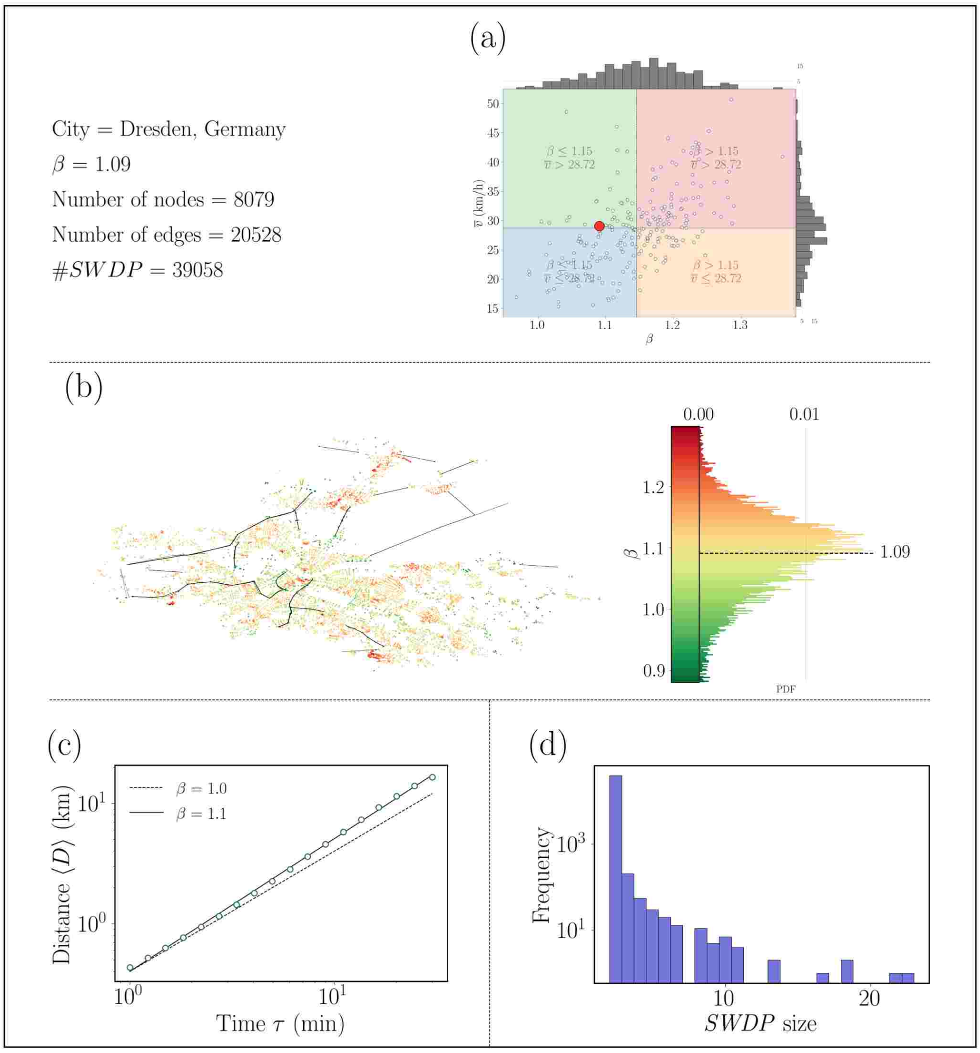

Fig. S55. Methodological sheet - Dresden, Germany. In (a) Each point represents a city, with mean exponent (β), on the x-axis, and mean speed $\overline{v}$ obtained in all trips made to calculate the exponent on the axis y. The histograms of the values of β and $\overline{v}$ are shown on the axes in the upper and right corners, respectively. The graph was segmented into four quadrants, in which the division is performed by the mean values of β and $\overline{v}$. The quadrants were colored and annotated according to the division criteria. The red dot represents the location of Dresden, Germany. In (b) taking all the nodes of Dresden, Germany as origin, the dots are colored as a function of their exponent value and their color is quantified by the color bar in the center. The longest segments without a deceleration point (SWDP) are plotted in black. The probability density function of the $\beta's$ for each experiment is shown on the left of the color scale Figure (c) shows the mean correlation curve between time τ and the distance $\langle D \rangle$. The black traced line represents the exponent equal to 1.0. Figure (d) shows the distribution of SWDP sizes in number of nodes per frequency of occurrence.

Dubai, United Arab Emirates

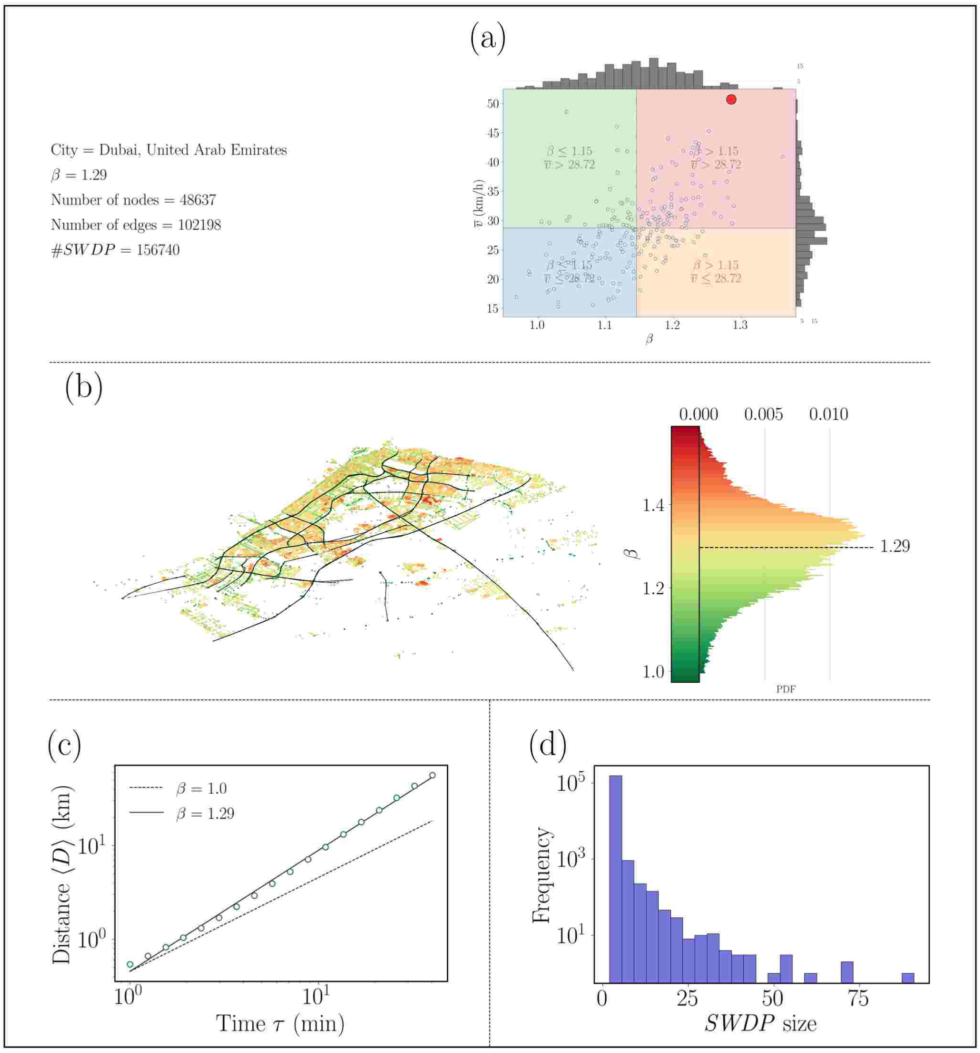

Fig. S56. Methodological sheet - Dubai, United Arab Emirates. In (a) Each point represents a city, with mean exponent (β), on the x-axis, and mean speed $\overline{v}$ obtained in all trips made to calculate the exponent on the axis y. The histograms of the values of β and $\overline{v}$ are shown on the axes in the upper and right corners, respectively. The graph was segmented into four quadrants, in which the division is performed by the mean values of β and $\overline{v}$. The quadrants were colored and annotated according to the division criteria. The red dot represents the location of Dubai, United Arab Emirates. In (b) taking all the nodes of Dubai, United Arab Emirates as origin, the dots are colored as a function of their exponent value and their color is quantified by the color bar in the center. The longest segments without a deceleration point (SWDP) are plotted in black. The probability density function of the $\beta's$ for each experiment is shown on the left of the color scale Figure (c) shows the mean correlation curve between time τ and the distance $\langle D \rangle$. The black traced line represents the exponent equal to 1.0. Figure (d) shows the distribution of SWDP sizes in number of nodes per frequency of occurrence.

Dublin, Ireland

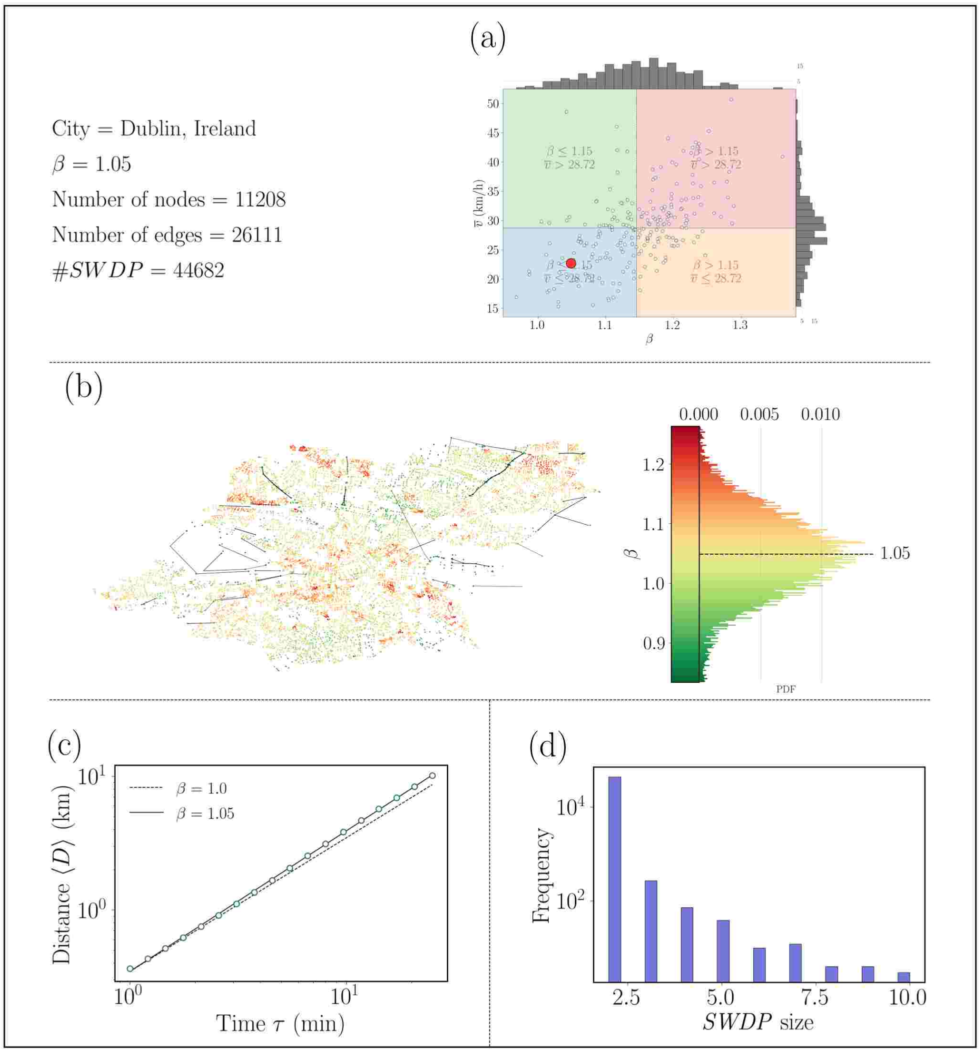

Fig. S57. Methodological sheet - Dublin, Ireland. In (a) Each point represents a city, with mean exponent (β), on the x-axis, and mean speed $\overline{v}$ obtained in all trips made to calculate the exponent on the axis y. The histograms of the values of β and $\overline{v}$ are shown on the axes in the upper and right corners, respectively. The graph was segmented into four quadrants, in which the division is performed by the mean values of β and $\overline{v}$. The quadrants were colored and annotated according to the division criteria. The red dot represents the location of Dublin, Ireland. In (b) taking all the nodes of Dublin, Ireland as origin, the dots are colored as a function of their exponent value and their color is quantified by the color bar in the center. The longest segments without a deceleration point (SWDP) are plotted in black. The probability density function of the $\beta' s$ for each experiment is shown on the left of the color scale Figure (c) shows the mean correlation curve between time τ and the distance $\langle D \rangle$. The black traced line represents the exponent equal to 1.0. Figure (d) shows the distribution of SWDP sizes in number of nodes per frequency of occurrence.

Edinburgh, UK

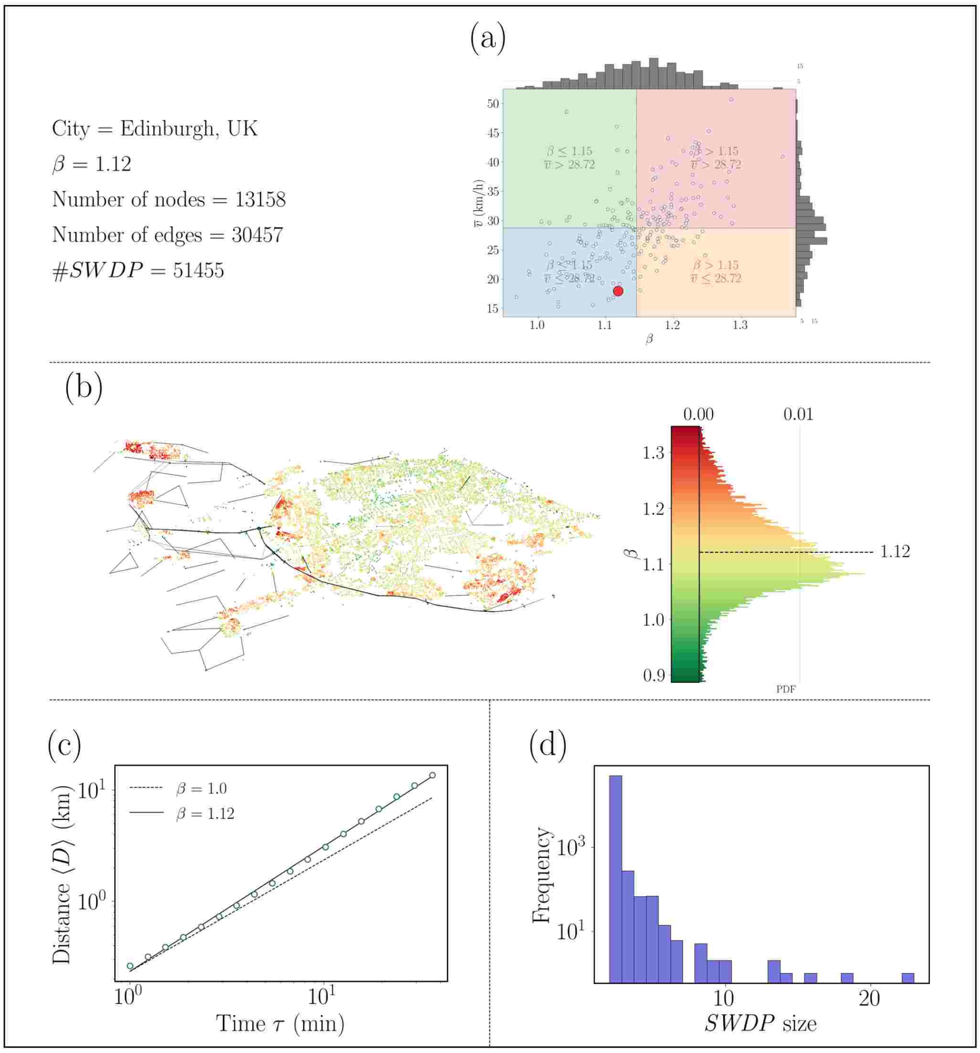

Fig. S58. Methodological sheet - Edinburgh, UK. In (a) Each point represents a city, with mean exponent (β), on the x-axis, and mean speed $\overline{v}$ obtained in all trips made to calculate the exponent on the axis y. The histograms of the values of β and $\overline{v}$ are shown on the axes in the upper and right corners, respectively. The graph was segmented into four quadrants, in which the division is performed by the mean values of β and $\overline{v}$. The quadrants were colored and annotated according to the division criteria. The red dot represents the location of Edinburgh, UK. In (b) taking all the nodes of Edinburgh, UK as origin, the dots are colored as a function of their exponent value and their color is quantified by the color bar in the center. The longest segments without a deceleration point (SWDP) are plotted in black. The probability density function of the $\beta's$ for each experiment is shown on the left of the color scale Figure (c) shows the mean correlation curve between time τ and the distance $\langle D \rangle$. The black traced line represents the exponent equal to 1.0. Figure (d) shows the distribution of SWDP sizes in number of nodes per frequency of occurrence.

Edmonton, Canadá

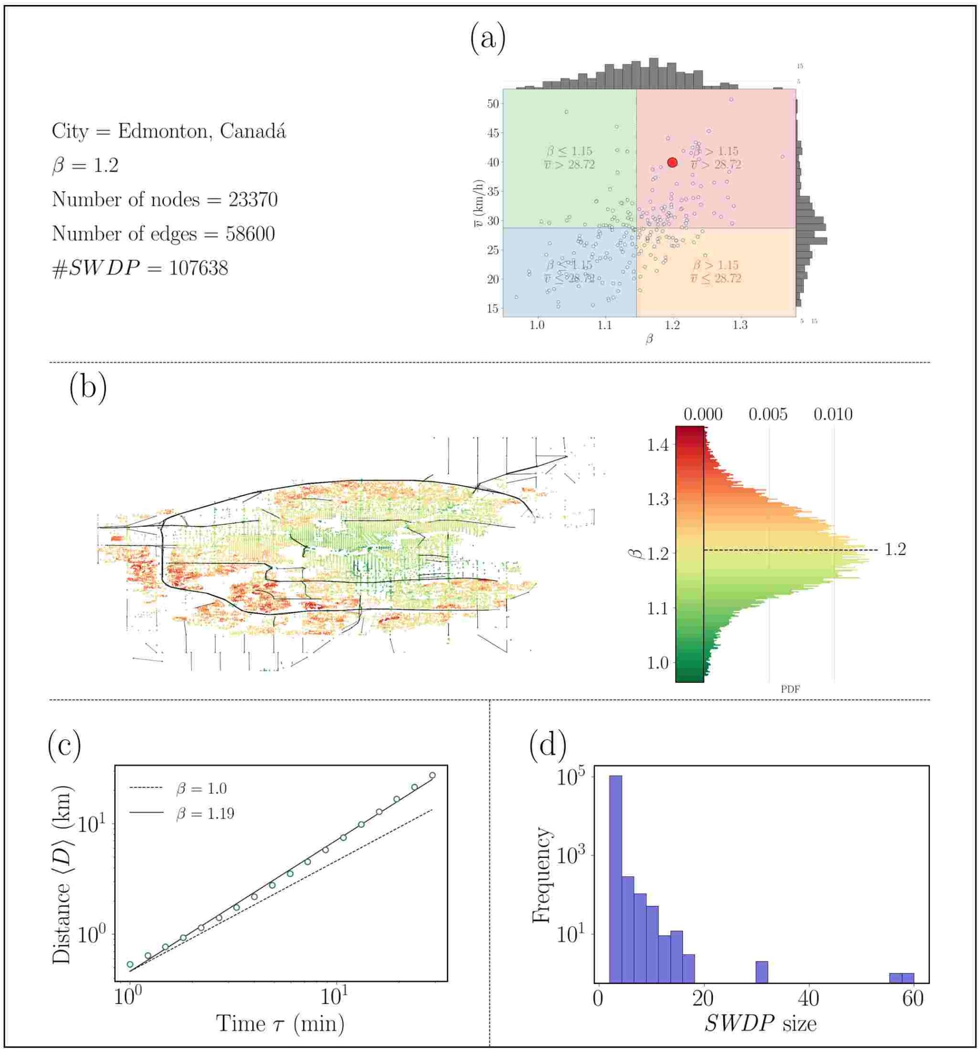

Fig. S59. Methodological sheet - Edmonton, Canadá. In (a) Each point represents a city, with mean exponent (β), on the x-axis, and mean speed $\overline{v}$ obtained in all trips made to calculate the exponent on the axis y. The histograms of the values of β and $\overline{v}$ are shown on the axes in the upper and right corners, respectively. The graph was segmented into four quadrants, in which the division is performed by the mean values of β and $\overline{v}$. The quadrants were colored and annotated according to the division criteria. The red dot represents the location of Edmonton, Canadá. In (b) taking all the nodes of Edmonton, Canadá as origin, the dots are colored as a function of their exponent value and their color is quantified by the color bar in the center. The longest segments without a deceleration point (SWDP) are plotted in black. The probability density function of the $\beta's$ for each experiment is shown on the left of the color scale Figure (c) shows the mean correlation curve between time τ and the distance $\langle D \rangle$. The black traced line represents the exponent equal to 1.0. Figure (d) shows the distribution of SWDP sizes in number of nodes per frequency of occurrence.

Eugene, Oregon, USA

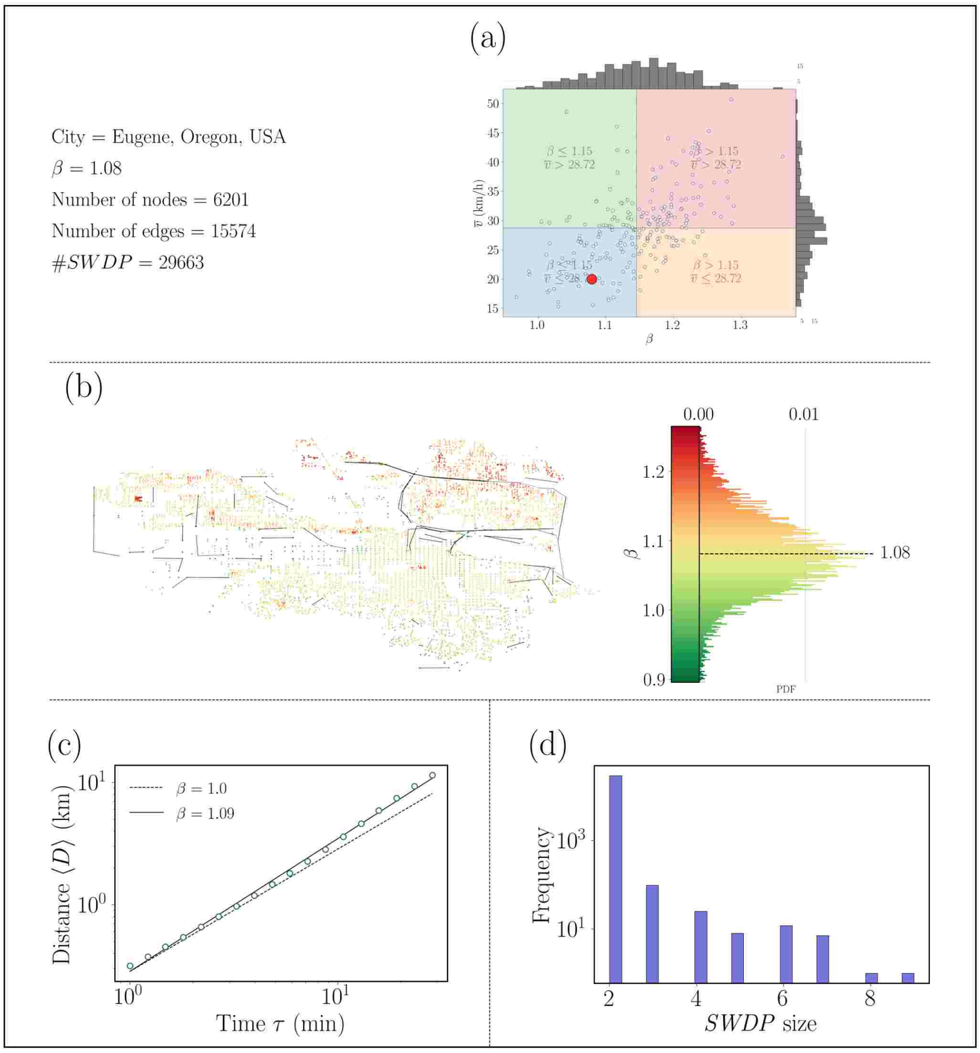

Fig. S60. Methodological sheet - Eugene, Oregon, USA. In (a) Each point represents a city, with mean exponent (β), on the x-axis, and mean speed $\overline{v}$ obtained in all trips made to calculate the exponent on the axis y. The histograms of the values of β and $\overline{v}$ are shown on the axes in the upper and right corners, respectively. The graph was segmented into four quadrants, in which the division is performed by the mean values of β and $\overline{v}$. The quadrants were colored and annotated according to the division criteria. The red dot represents the location of Eugene, Oregon, USA. In (b) taking all the nodes of Eugene, Oregon, USA as origin, the dots are colored as a function of their exponent value and their color is quantified by the color bar in the center. The longest segments without a deceleration point (SWDP) are plotted in black. The probability density function of the $\beta's$ for each experiment is shown on the left of the color scale Figure (c) shows the mean correlation curve between time τ and the distance $\langle D \rangle$. The black traced line represents the exponent equal to 1.0. Figure (d) shows the distribution of SWDP sizes in number of nodes per frequency of occurrence.

Fes, Morocco

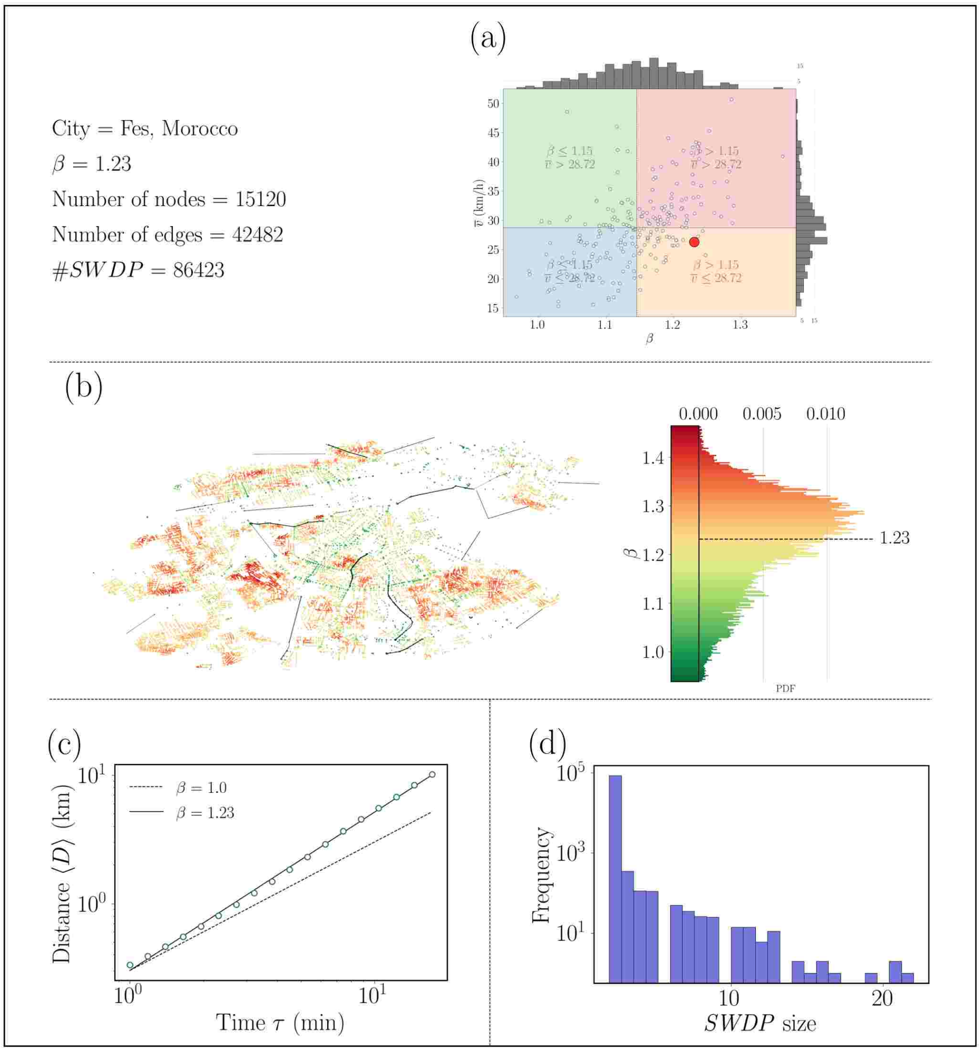

Fig. S61. Methodological sheet - Fes, Morocco. In (a) Each point represents a city, with mean exponent (β), on the x-axis, and mean speed $\overline{v}$ obtained in all trips made to calculate the exponent on the axis y. The histograms of the values of β and $\overline{v}$ are shown on the axes in the upper and right corners, respectively. The graph was segmented into four quadrants, in which the division is performed by the mean values of β and $\overline{v}$. The quadrants were colored and annotated according to the division criteria. The red dot represents the location of Fes, Morocco. In (b) taking all the nodes of Fes, Morocco as origin, the dots are colored as a function of their exponent value and their color is quantified by the color bar in the center. The longest segments without a deceleration point (SWDP) are plotted in black. The probability density function of the $\beta's$ for each experiment is shown on the left of the color scale Figure (c) shows the mean correlation curve between time τ and the distance $\langle D \rangle$. The black traced line represents the exponent equal to 1.0. Figure (d) shows the distribution of SWDP sizes in number of nodes per frequency of occurrence.

Florianópolis, Brasil

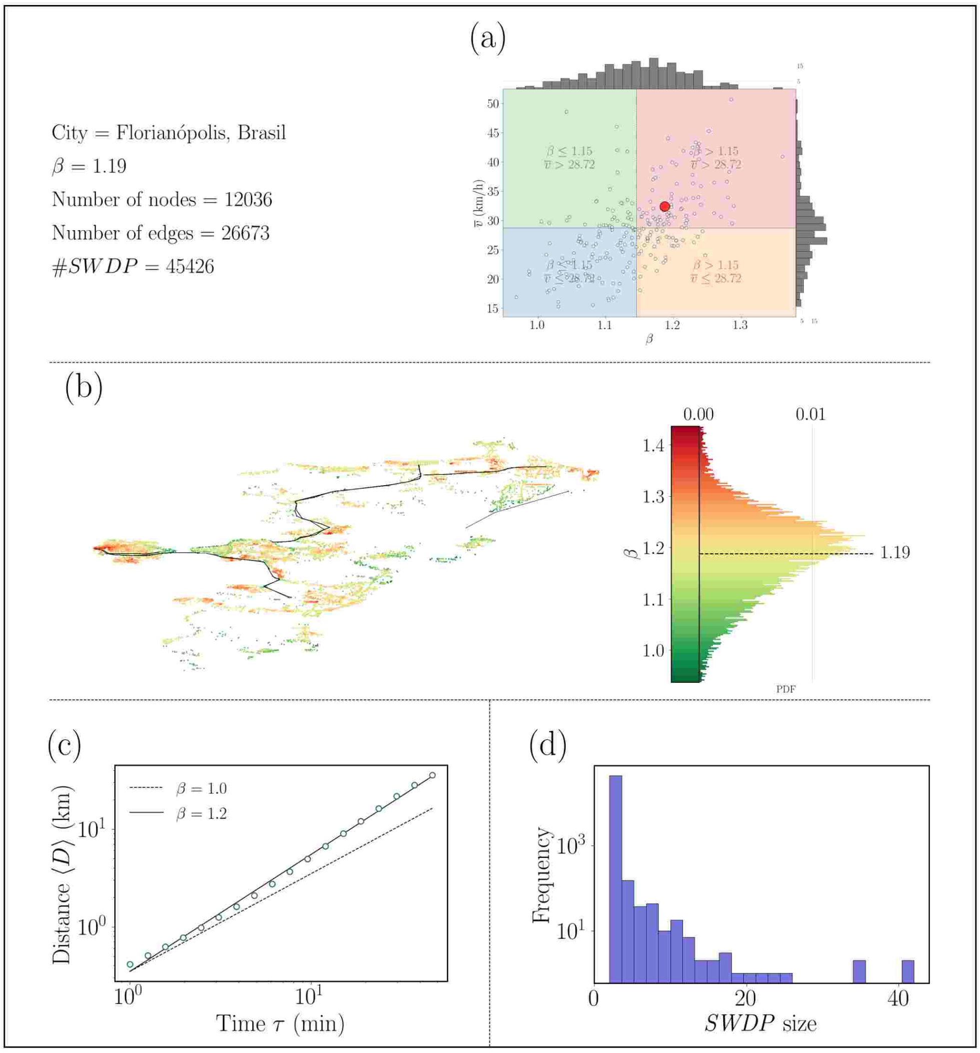

Fig. S62. Methodological sheet - Florianópolis, Brasil. In (a) Each point represents a city, with mean exponent (β), on the x-axis, and mean speed $\overline{v}$ obtained in all trips made to calculate the exponent on the axis y. The histograms of the values of β and $\overline{v}$ are shown on the axes in the upper and right corners, respectively. The graph was segmented into four quadrants, in which the division is performed by the mean values of β and $\overline{v}$. The quadrants were colored and annotated according to the division criteria. The red dot represents the location of Florianópolis, Brasil. In (b) taking all the nodes of Florianópolis, Brasil as origin, the dots are colored as a function of their exponent value and their color is quantified by the color bar in the center. The longest segments without a deceleration point (SWDP) are plotted in black. The probability density function of the $\beta' s$ for each experiment is shown on the left of the color scale Figure (c) shows the mean correlation curve between time τ and the distance $\langle D \rangle$. The black traced line represents the exponent equal to 1.0. Figure (d) shows the distribution of SWDP sizes in number of nodes per frequency of occurrence.

Fortaleza, Brasil

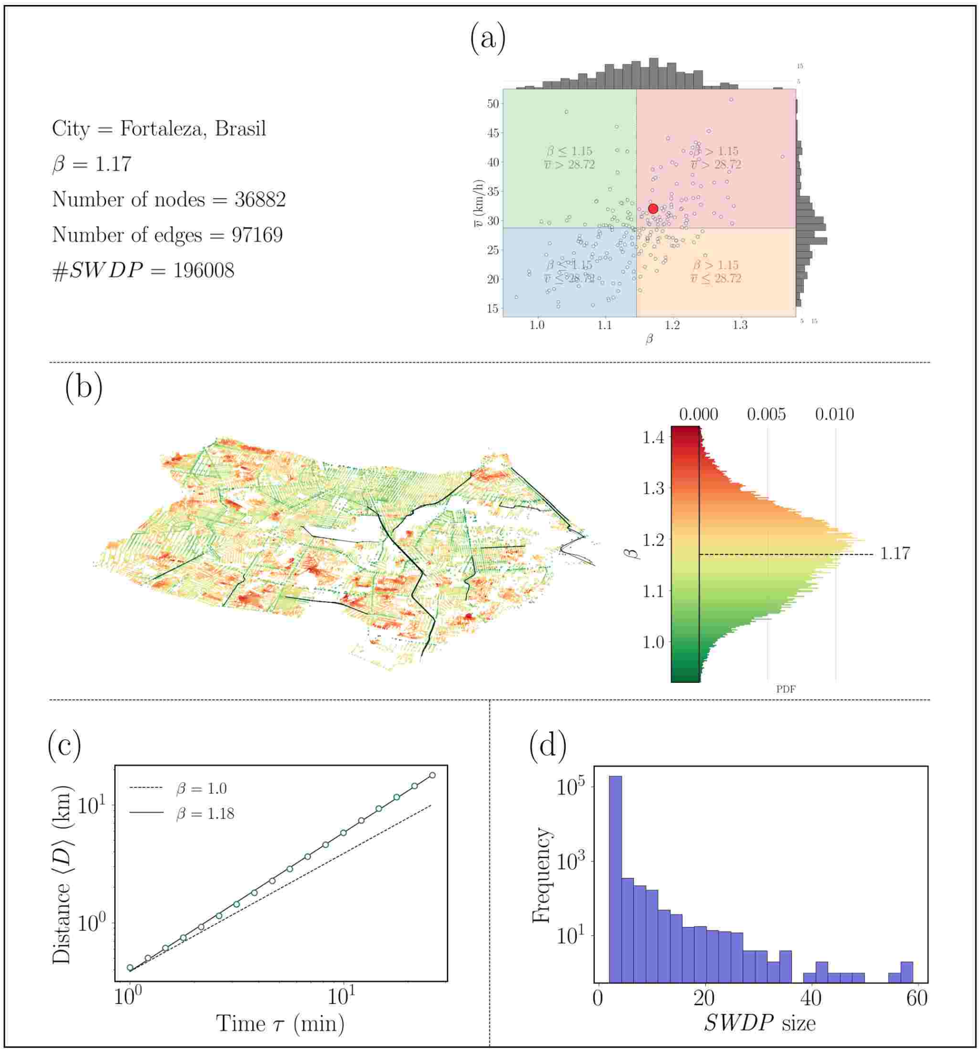

Fig. S63. Methodological sheet - Fortaleza, Brasil. In (a) Each point represents a city, with mean exponent (β), on the x-axis, and mean speed $\overline{v}$ obtained in all trips made to calculate the exponent on the axis y. The histograms of the values of β and $\overline{v}$ are shown on the axes in the upper and right corners, respectively. The graph was segmented into four quadrants, in which the division is performed by the mean values of β and $\overline{v}$. The quadrants were colored and annotated according to the division criteria. The red dot represents the location of Fortaleza, Brasil. In (b) taking all the nodes of Fortaleza, Brasil as origin, the dots are colored as a function of their exponent value and their color is quantified by the color bar in the center. The longest segments without a deceleration point (SWDP) are plotted in black. The probability density function of the $\beta's$ for each experiment is shown on the left of the color scale Figure (c) shows the mean correlation curve between time τ and the distance $\langle D \rangle$. The black traced line represents the exponent equal to 1.0. Figure (d) shows the distribution of SWDP sizes in number of nodes per frequency of occurrence.

Frankfurt, Germany

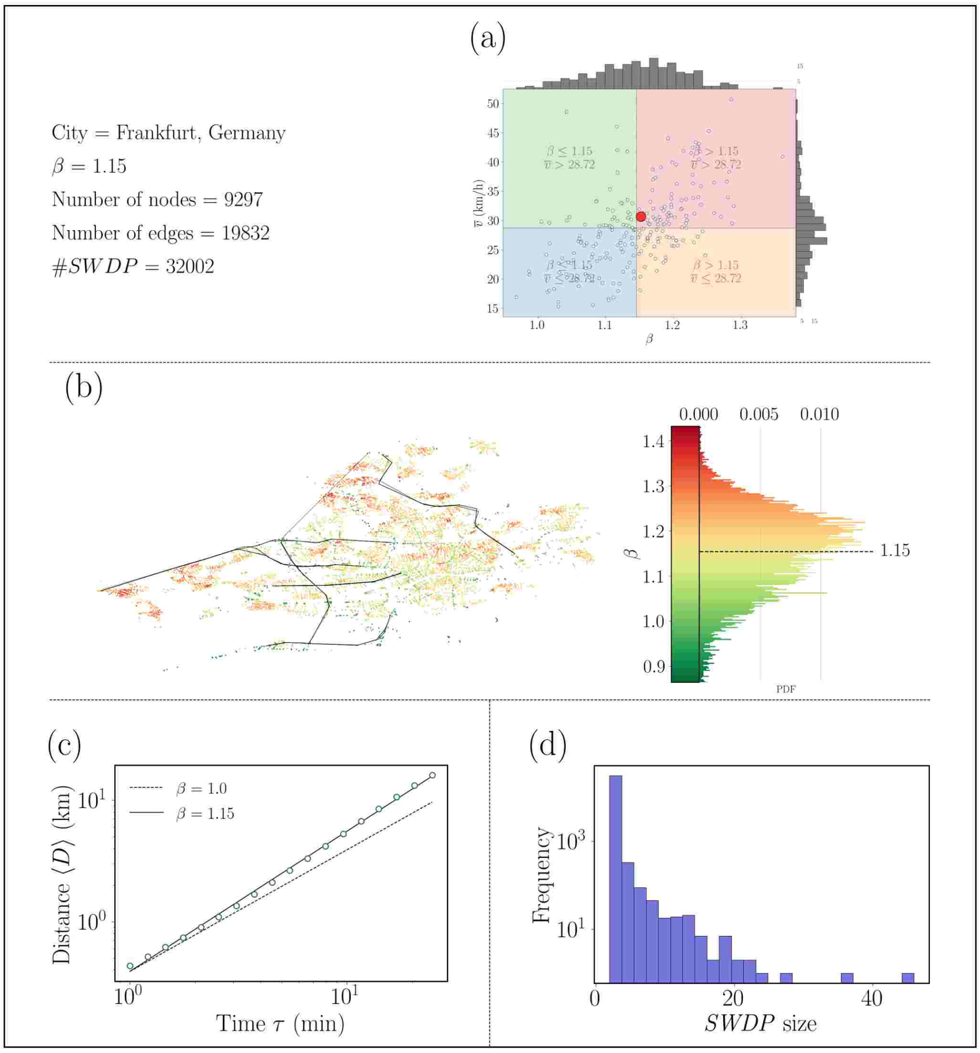

Fig. S64. Methodological sheet - Frankfurt, Germany. In (a) Each point represents a city, with mean exponent (β), on the x-axis, and mean speed $\overline{v}$ obtained in all trips made to calculate the exponent on the axis y. The histograms of the values of β and $\overline{v}$ are shown on the axes in the upper and right corners, respectively. The graph was segmented into four quadrants, in which the division is performed by the mean values of β and $\overline{v}$. The quadrants were colored and annotated according to the division criteria. The red dot represents the location of Frankfurt, Germany. In (b) taking all the nodes of Frankfurt, Germany as origin, the dots are colored as a function of their exponent value and their color is quantified by the color bar in the center. The longest segments without a deceleration point (SWDP) are plotted in black. The probability density function of the $\beta's$ for each experiment is shown on the left of the color scale Figure (c) shows the mean correlation curve between time τ and the distance $\langle D \rangle$. The black traced line represents the exponent equal to 1.0. Figure (d) shows the distribution of SWDP sizes in number of nodes per frequency of occurrence.

Geneva, Switzerland

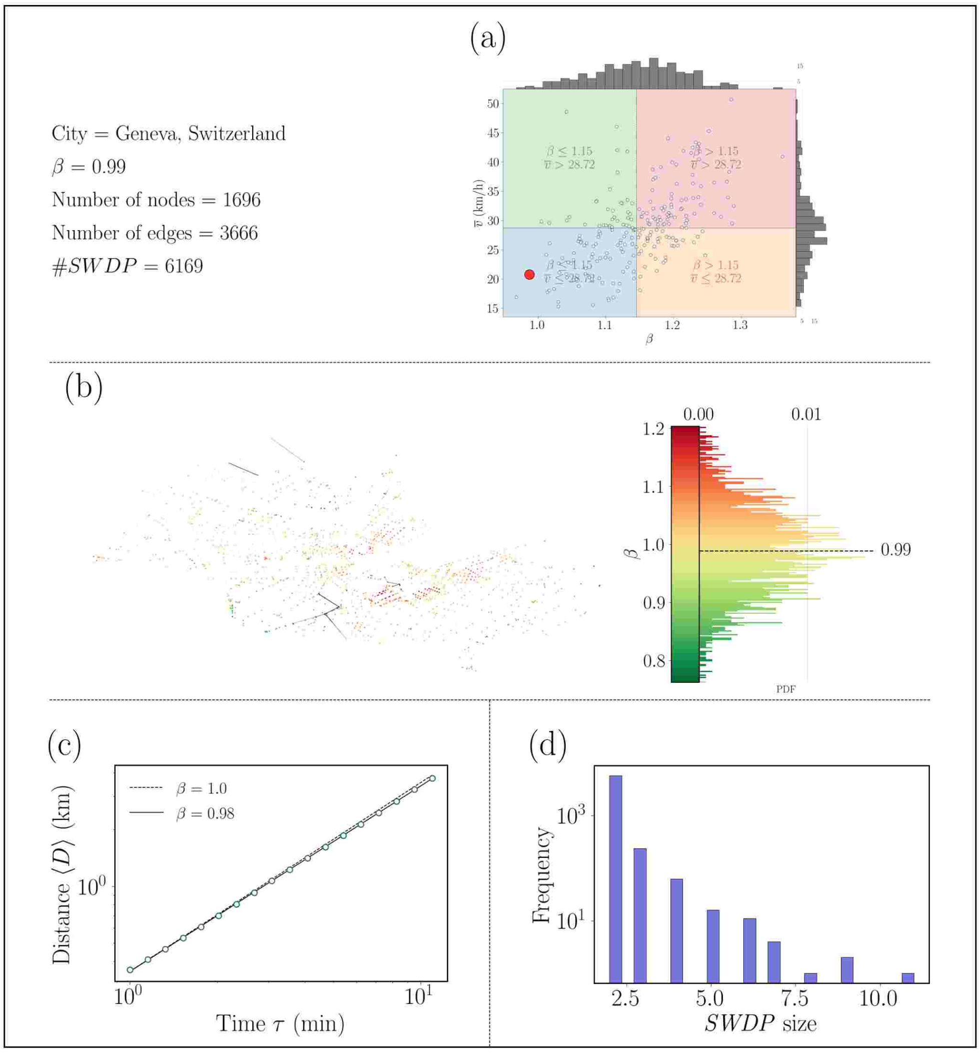

Fig. S65. Methodological sheet - Geneva, Switzerland. In (a) Each point represents a city, with mean exponent (β), on the x-axis, and mean speed $\overline{v}$ obtained in all trips made to calculate the exponent on the axis y. The histograms of the values of β and $\overline{v}$ are shown on the axes in the upper and right corners, respectively. The graph was segmented into four quadrants, in which the division is performed by the mean values of β and $\overline{v}$. The quadrants were colored and annotated according to the division criteria. The red dot represents the location of Geneva, Switzerland. In (b) taking all the nodes of Geneva, Switzerland as origin, the dots are colored as a function of their exponent value and their color is quantified by the color bar in the center. The longest segments without a deceleration point (SWDP) are plotted in black. The probability density function of the $\beta's$ for each experiment is shown on the left of the color scale Figure (c) shows the mean correlation curve between time τ and the distance $\langle D \rangle$. The black traced line represents the exponent equal to 1.0. Figure (d) shows the distribution of SWDP sizes in number of nodes per frequency of occurrence.

Genoa, Italy

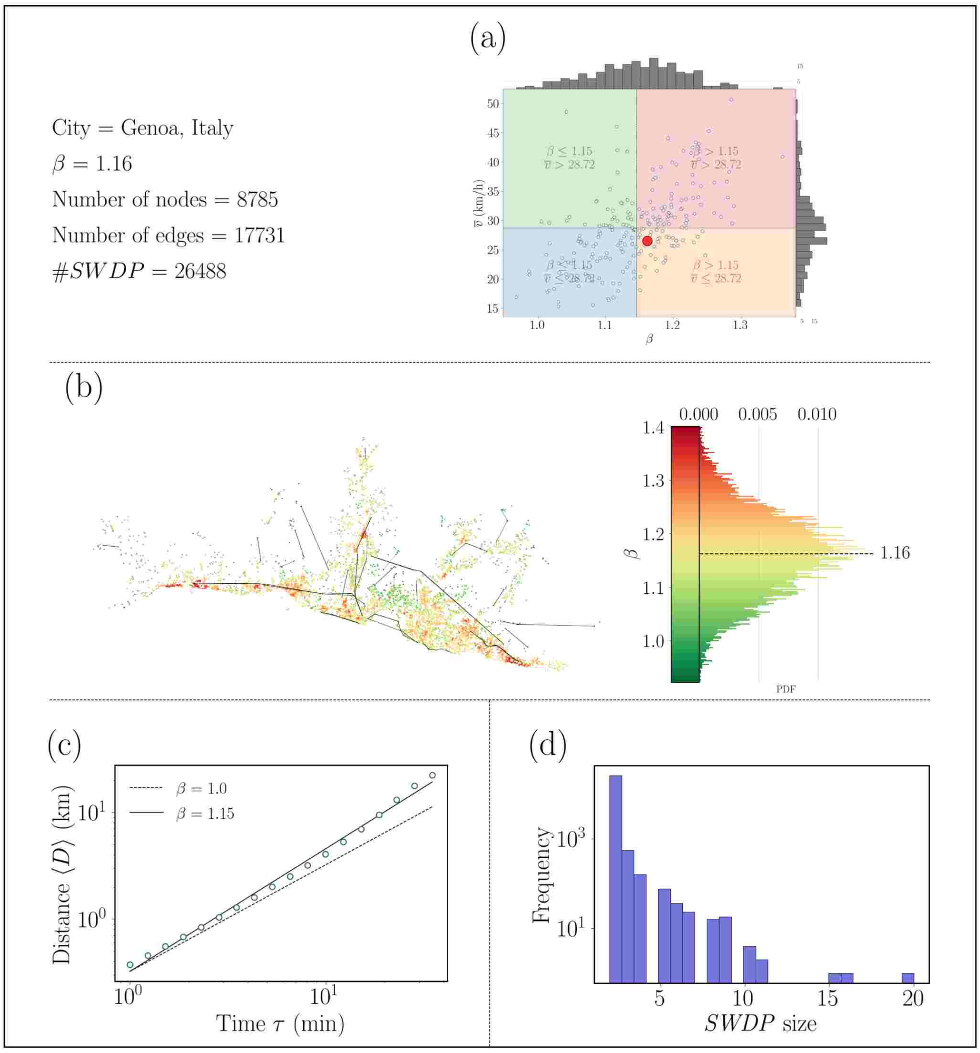

Fig. S66. Methodological sheet - Genoa, Italy. In (a) Each point represents a city, with mean exponent (β), on the x-axis, and mean speed $\overline{v}$ obtained in all trips made to calculate the exponent on the axis y. The histograms of the values of β and $\overline{v}$ are shown on the axes in the upper and right corners, respectively. The graph was segmented into four quadrants, in which the division is performed by the mean values of β and $\overline{v}$. The quadrants were colored and annotated according to the division criteria. The red dot represents the location of Genoa, Italy. In (b) taking all the nodes of Genoa, Italy as origin, the dots are colored as a function of their exponent value and their color is quantified by the color bar in the center. The longest segments without a deceleration point (SWDP) are plotted in black. The probability density function of the $\beta's$ for each experiment is shown on the left of the color scale Figure (c) shows the mean correlation curve between time τ and the distance $\langle D \rangle$. The black traced line represents the exponent equal to 1.0. Figure (d) shows the distribution of SWDP sizes in number of nodes per frequency of occurrence.

Goiânia, Brasil

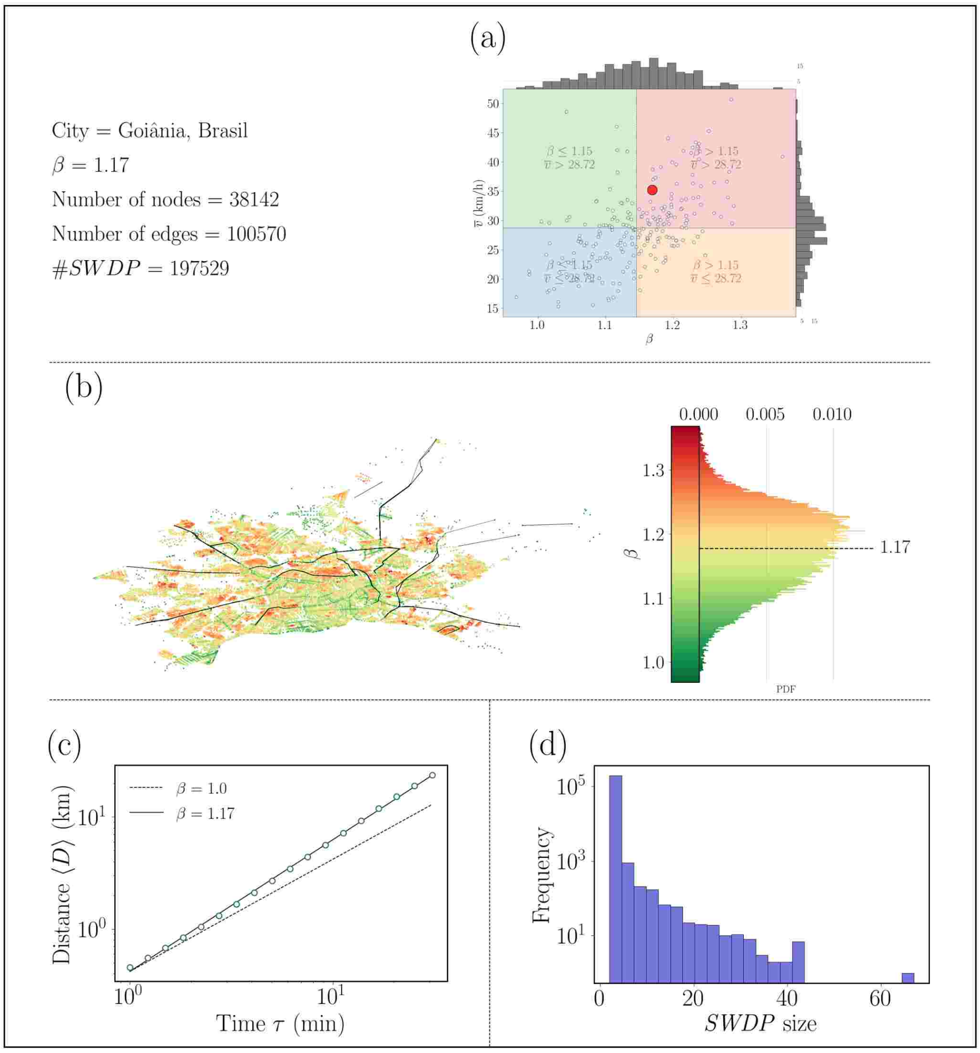

Fig. S67. Methodological sheet - Goiânia, Brasil. In (a) Each point represents a city, with mean exponent (β), on the x-axis, and mean speed $\overline{v}$ obtained in all trips made to calculate the exponent on the axis y. The histograms of the values of β and $\overline{v}$ are shown on the axes in the upper and right corners, respectively. The graph was segmented into four quadrants, in which the division is performed by the mean values of β and $\overline{v}$. The quadrants were colored and annotated according to the division criteria. The red dot represents the location of Goiânia, Brasil. In (b) taking all the nodes of Goiânia, Brasil as origin, the dots are colored as a function of their exponent value and their color is quantified by the color bar in the center. The longest segments without a deceleration point (SWDP) are plotted in black. The probability density function of the $\beta's$ for each experiment is shown on the left of the color scale Figure (c) shows the mean correlation curve between time τ and the distance $\langle D \rangle$. The black traced line represents the exponent equal to 1.0. Figure (d) shows the distribution of SWDP sizes in number of nodes per frequency of occurrence.

Graz, Austria

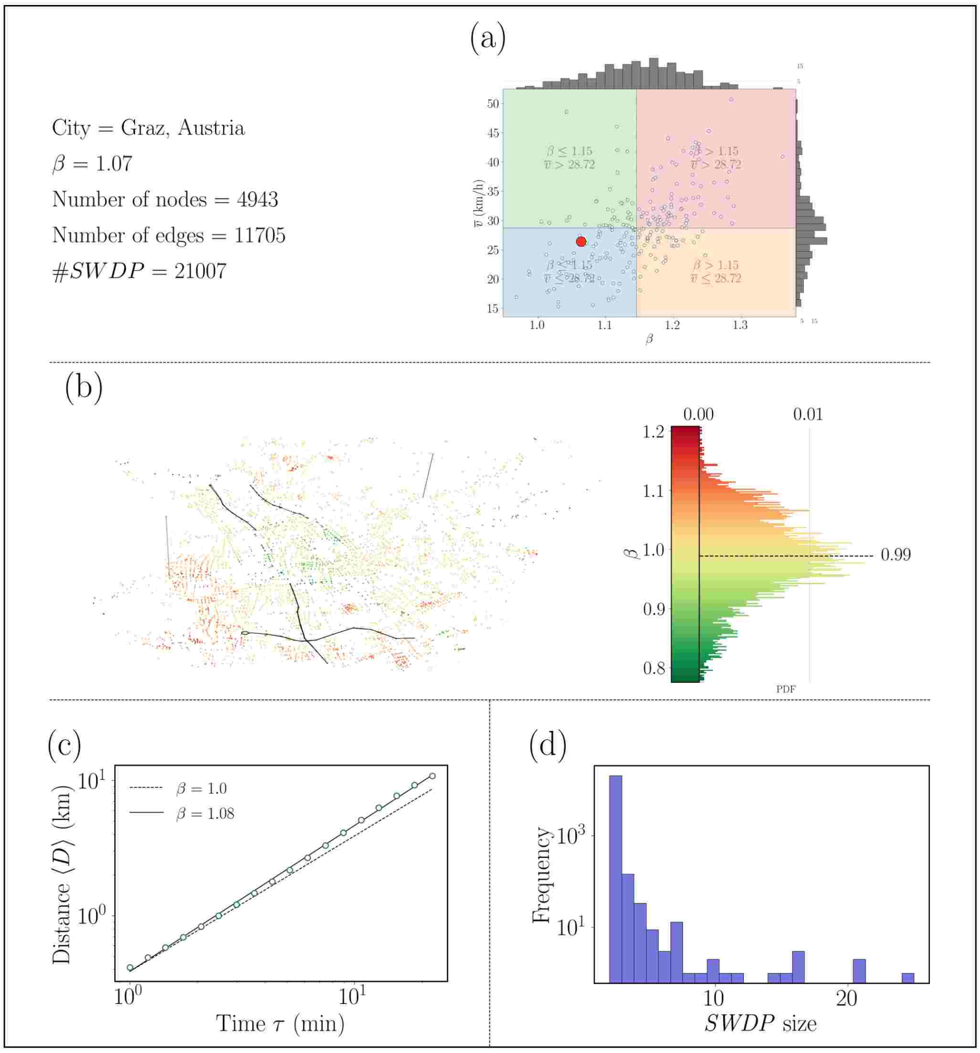

Fig. S68. Methodological sheet - Graz, Austria. In (a) Each point represents a city, with mean exponent (β), on the x-axis, and mean speed $\overline{v}$ obtained in all trips made to calculate the exponent on the axis y. The histograms of the values of β and $\overline{v}$ are shown on the axes in the upper and right corners, respectively. The graph was segmented into four quadrants, in which the division is performed by the mean values of β and $\overline{v}$. The quadrants were colored and annotated according to the division criteria. The red dot represents the location of Graz, Austria. In (b) taking all the nodes of Graz, Austria as origin, the dots are colored as a function of their exponent value and their color is quantified by the color bar in the center. The longest segments without a deceleration point (SWDP) are plotted in black. The probability density function of the $\beta's$ for each experiment is shown on the left of the color scale Figure (c) shows the mean correlation curve between time τ and the distance $\langle D \rangle$. The black traced line represents the exponent equal to 1.0. Figure (d) shows the distribution of SWDP sizes in number of nodes per frequency of occurrence.

Guadalajara, Mexico

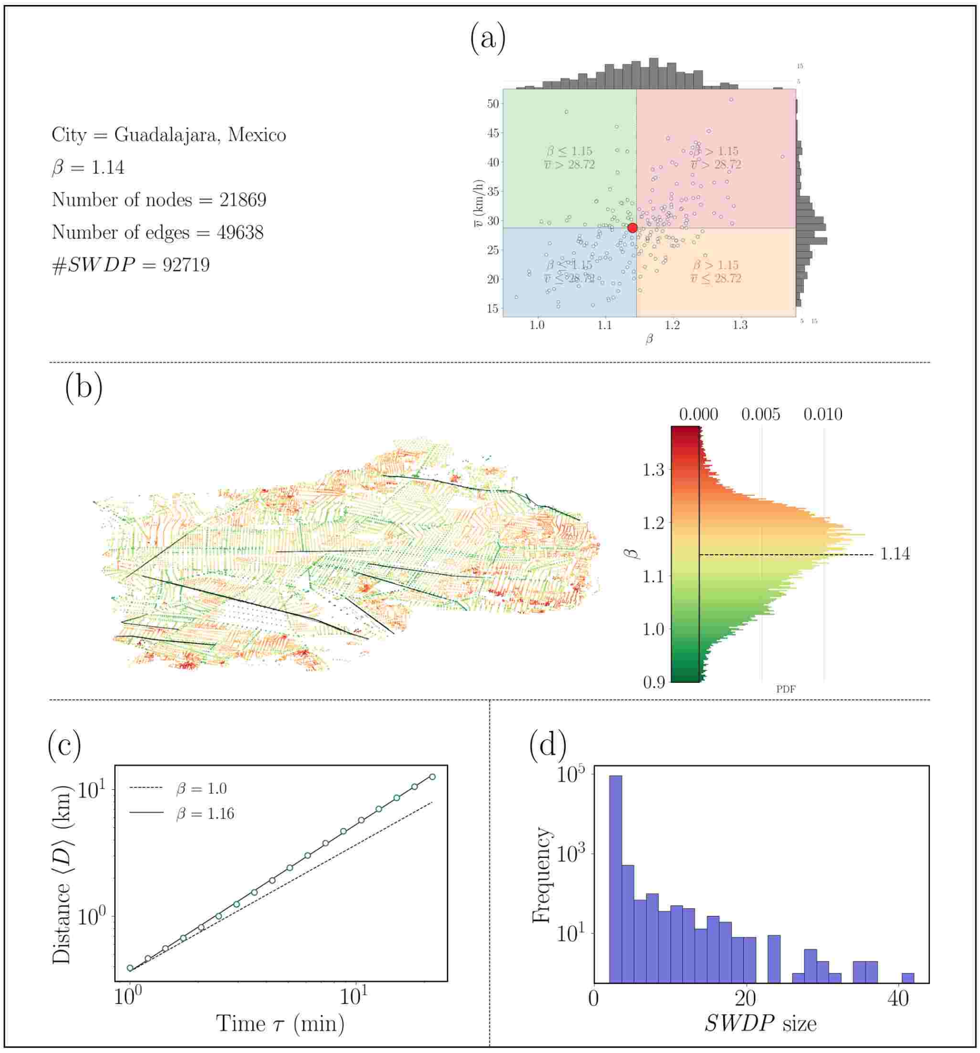

Fig. S69. Methodological sheet - Guadalajara, Mexico. In (a) Each point represents a city, with mean exponent (β), on the x-axis, and mean speed $\overline{v}$ obtained in all trips made to calculate the exponent on the axis y. The histograms of the values of β and $\overline{v}$ are shown on the axes in the upper and right corners, respectively. The graph was segmented into four quadrants, in which the division is performed by the mean values of β and $\overline{v}$. The quadrants were colored and annotated according to the division criteria. The red dot represents the location of Guadalajara, Mexico. In (b) taking all the nodes of Guadalajara, Mexico as origin, the dots are colored as a function of their exponent value and their color is quantified by the color bar in the center. The longest segments without a deceleration point (SWDP) are plotted in black. The probability density function of the $\beta's$ for each experiment is shown on the left of the color scale Figure (c) shows the mean correlation curve between time τ and the distance $\langle D \rangle$. The black traced line represents the exponent equal to 1.0. Figure (d) shows the distribution of SWDP sizes in number of nodes per frequency of occurrence.

Guayaquil, Ecuador

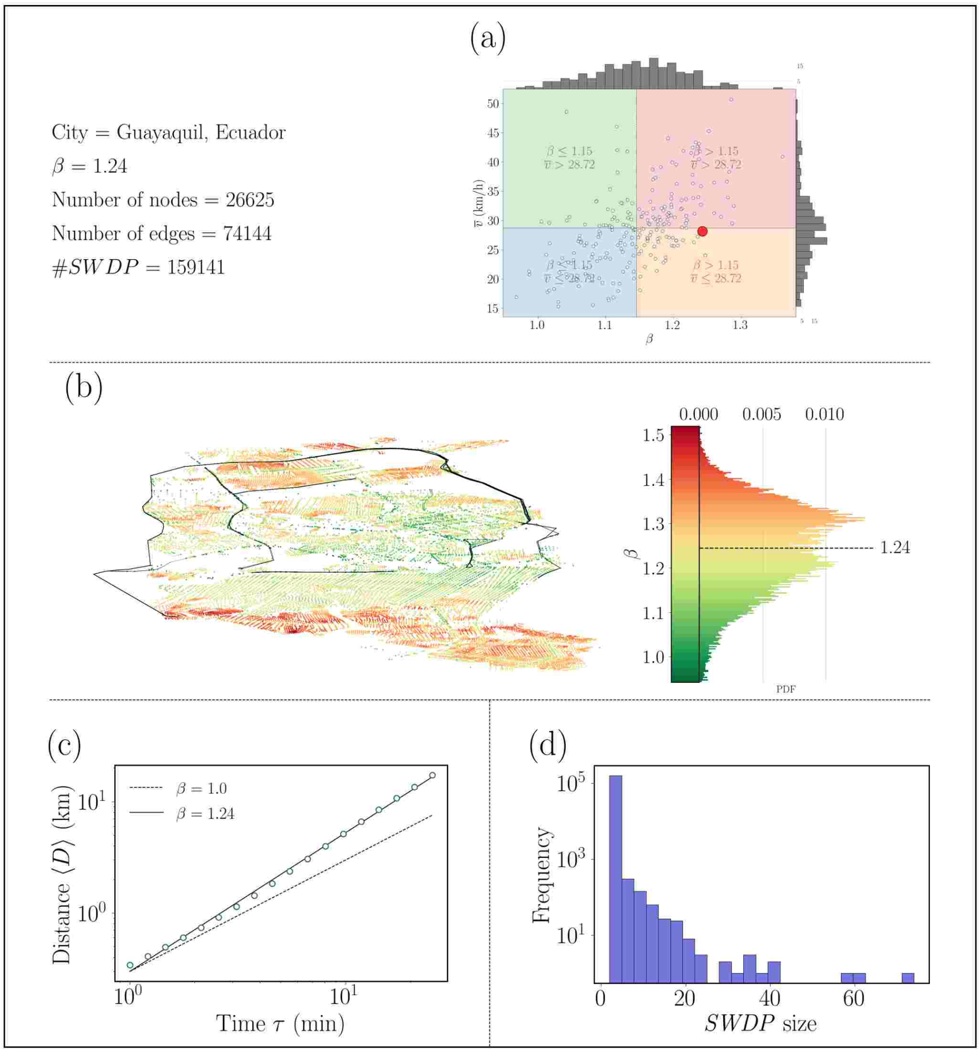

Fig. S70. Methodological sheet - Guayaquil, Ecuador. In (a) Each point represents a city, with mean exponent (β), on the x-axis, and mean speed $\overline{v}$ obtained in all trips made to calculate the exponent on the axis y. The histograms of the values of β and $\overline{v}$ are shown on the axes in the upper and right corners, respectively. The graph was segmented into four quadrants, in which the division is performed by the mean values of β and $\overline{v}$. The quadrants were colored and annotated according to the division criteria. The red dot represents the location of Guayaquil, Ecuador. In (b) taking all the nodes of Guayaquil, Ecuador as origin, the dots are colored as a function of their exponent value and their color is quantified by the color bar in the center. The longest segments without a deceleration point (SWDP) are plotted in black. The probability density function of the $\beta's$ for each experiment is shown on the left of the color scale Figure (c) shows the mean correlation curve between time τ and the distance $\langle D \rangle$. The black traced line represents the exponent equal to 1.0. Figure (d) shows the distribution of SWDP sizes in number of nodes per frequency of occurrence.

Hamilton, New Zealand

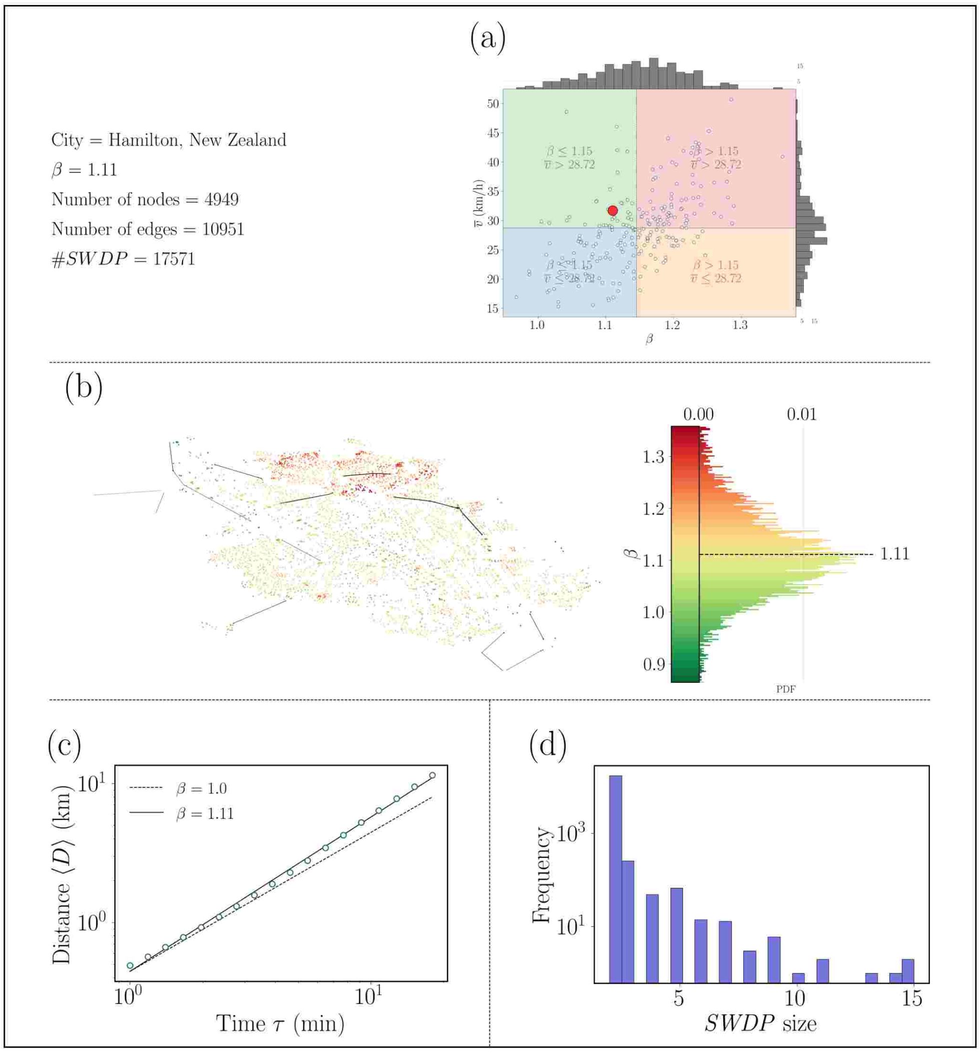

Fig. S71. Methodological sheet - Hamilton, New Zealand. In (a) Each point represents a city, with mean exponent (β), on the x-axis, and mean speed $\overline{v}$ obtained in all trips made to calculate the exponent on the axis y. The histograms of the values of β and $\overline{v}$ are shown on the axes in the upper and right corners, respectively. The graph was segmented into four quadrants, in which the division is performed by the mean values of β and $\overline{v}$. The quadrants were colored and annotated according to the division criteria. The red dot represents the location of Hamilton, New Zealand. In (b) taking all the nodes of Hamilton, New Zealand as origin, the dots are colored as a function of their exponent value and their color is quantified by the color bar in the center. The longest segments without a deceleration point (SWDP) are plotted in black. The probability density function of the $\beta's$ for each experiment is shown on the left of the color scale Figure (c) shows the mean correlation curve between time τ and the distance $\langle D \rangle$. The black traced line represents the exponent equal to 1.0. Figure (d) shows the distribution of SWDP sizes in number of nodes per frequency of occurrence.

Hanoi, Vietnam

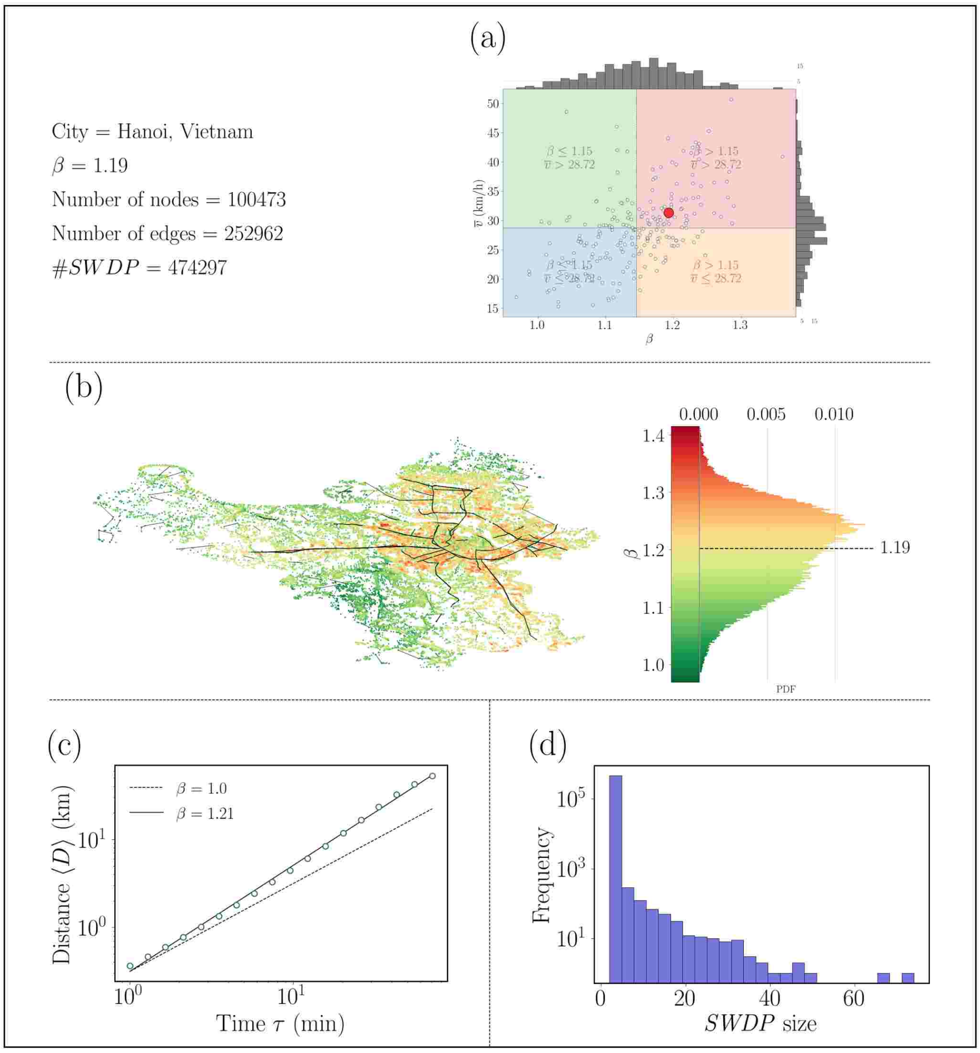

Fig. S72. Methodological sheet - Hanoi, Vietnam. In (a) Each point represents a city, with mean exponent (β), on the x-axis, and mean speed $\overline{v}$ obtained in all trips made to calculate the exponent on the axis y. The histograms of the values of β and $\overline{v}$ are shown on the axes in the upper and right corners, respectively. The graph was segmented into four quadrants, in which the division is performed by the mean values of β and $\overline{v}$. The quadrants were colored and annotated according to the division criteria. The red dot represents the location of Hanoi, Vietnam. In (b) taking all the nodes of Hanoi, Vietnam as origin, the dots are colored as a function of their exponent value and their color is quantified by the color bar in the center. The longest segments without a deceleration point (SWDP) are plotted in black. The probability density function of the $\beta's$ for each experiment is shown on the left of the color scale Figure (c) shows the mean correlation curve between time τ and the distance $\langle D \rangle$. The black traced line represents the exponent equal to 1.0. Figure (d) shows the distribution of SWDP sizes in number of nodes per frequency of occurrence.

Ho Chi Minh City, Vietnam

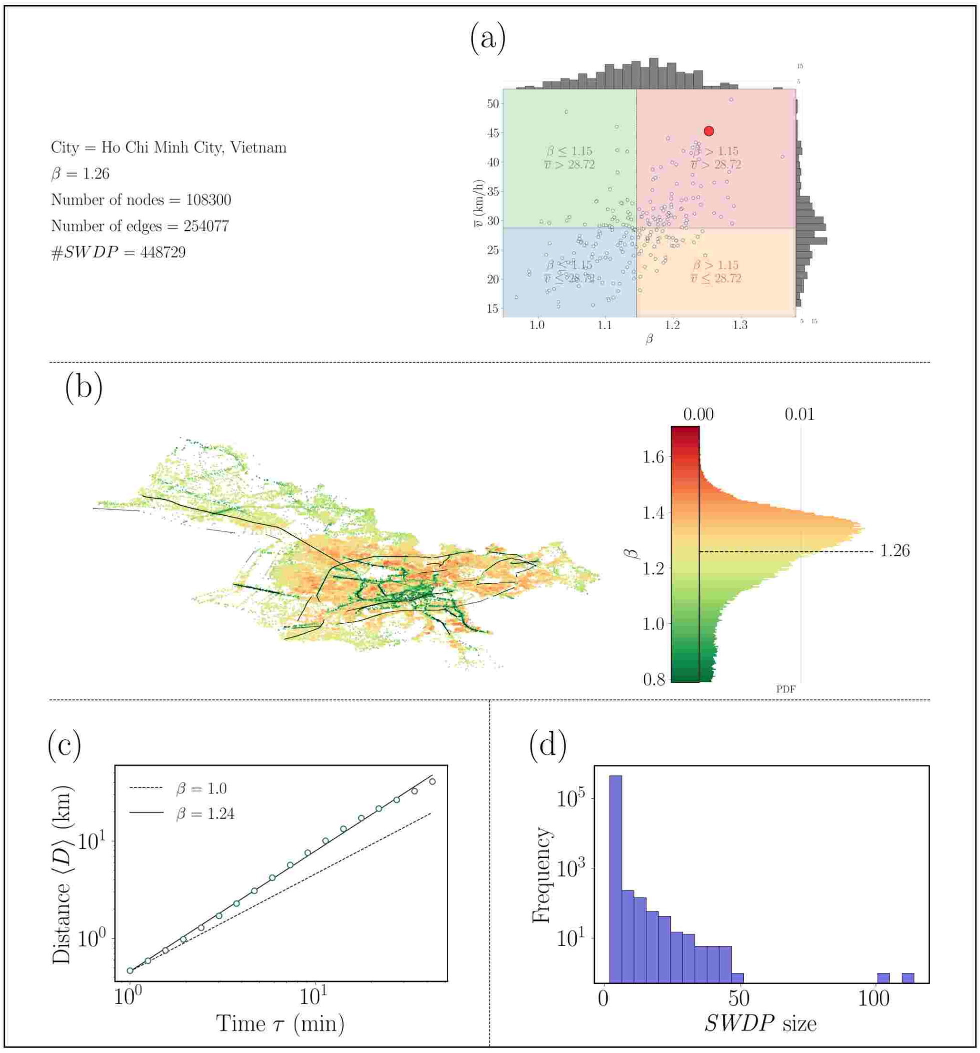

Fig. S73. Methodological sheet - Ho Chi Minh City, Vietnam. In (a) Each point represents a city, with mean exponent (β), on the x-axis, and mean speed $\overline{v}$ obtained in all trips made to calculate the exponent on the axis y. The histograms of the values of β and $\overline{v}$ are shown on the axes in the upper and right corners, respectively. The graph was segmented into four quadrants, in which the division is performed by the mean values of β and $\overline{v}$. The quadrants were colored and annotated according to the division criteria. The red dot represents the location of Ho Chi Minh City, Vietnam. In (b) taking all the nodes of Ho Chi Minh City, Vietnam as origin, the dots are colored as a function of their exponent value and their color is quantified by the color bar in the center. The longest segments without a deceleration point (SWDP) are plotted in black. The probability density function of the $\beta's$ for each experiment is shown on the left of the color scale Figure (c) shows the mean correlation curve between time τ and the distance $\langle D \rangle$. The black traced line represents the exponent equal to 1.0. Figure (d) shows the distribution of SWDP sizes in number of nodes per frequency of occurrence.

Hong Kong

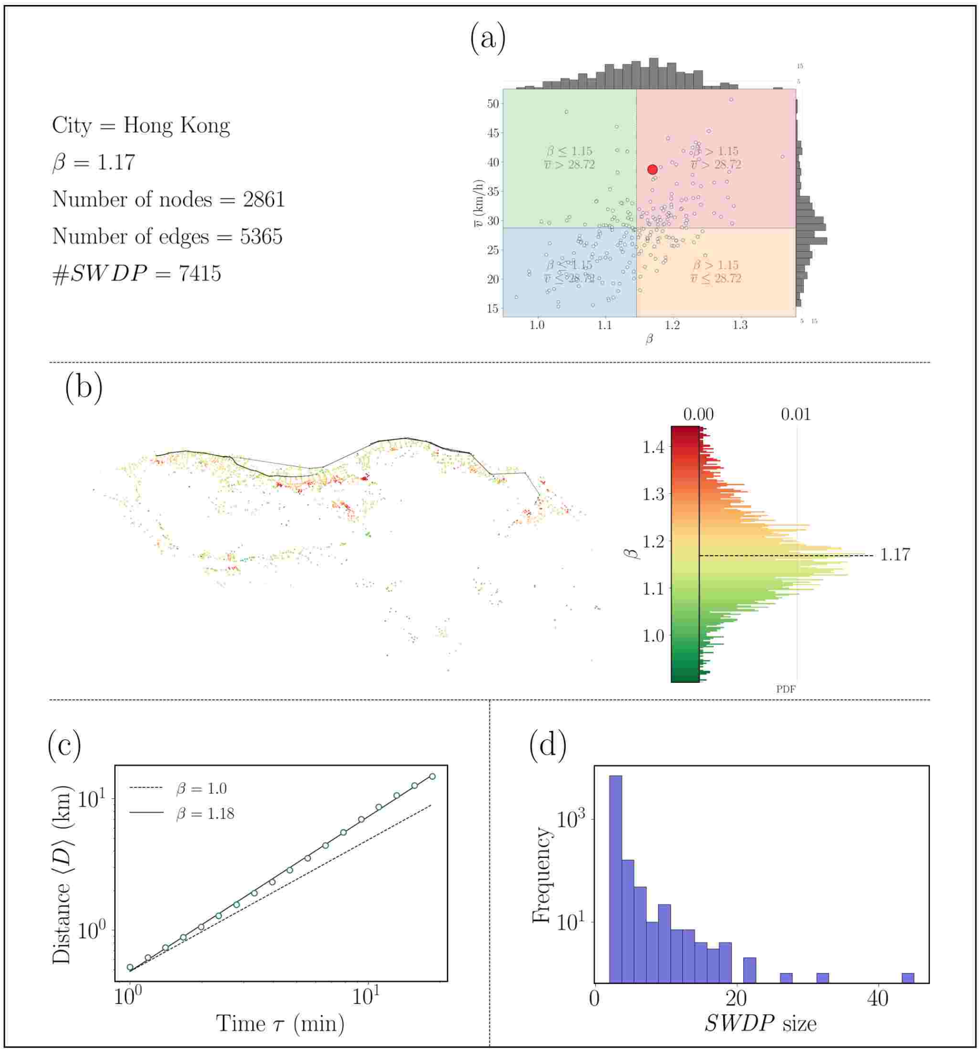

Fig. S74. Methodological sheet - Hong Kong. In (a) Each point represents a city, with mean exponent (β), on the x-axis, and mean speed $\overline{v}$ obtained in all trips made to calculate the exponent on the axis y. The histograms of the values of β and $\overline{v}$ are shown on the axes in the upper and right corners, respectively. The graph was segmented into four quadrants, in which the division is performed by the mean values of β and $\overline{v}$. The quadrants were colored and annotated according to the division criteria. The red dot represents the location of Hong Kong. In (b) taking all the nodes of Hong Kong as origin, the dots are colored as a function of their exponent value and their color is quantified by the color bar in the center. The longest segments without a deceleration point (SWDP) are plotted in black. The probability density function of the $\beta's$ for each experiment is shown on the left of the color scale Figure (c) shows the mean correlation curve between time τ and the distance $\langle D \rangle$. The black traced line represents the exponent equal to 1.0. Figure (d) shows the distribution of SWDP sizes in number of nodes per frequency of occurrence.

Honolulu, USA

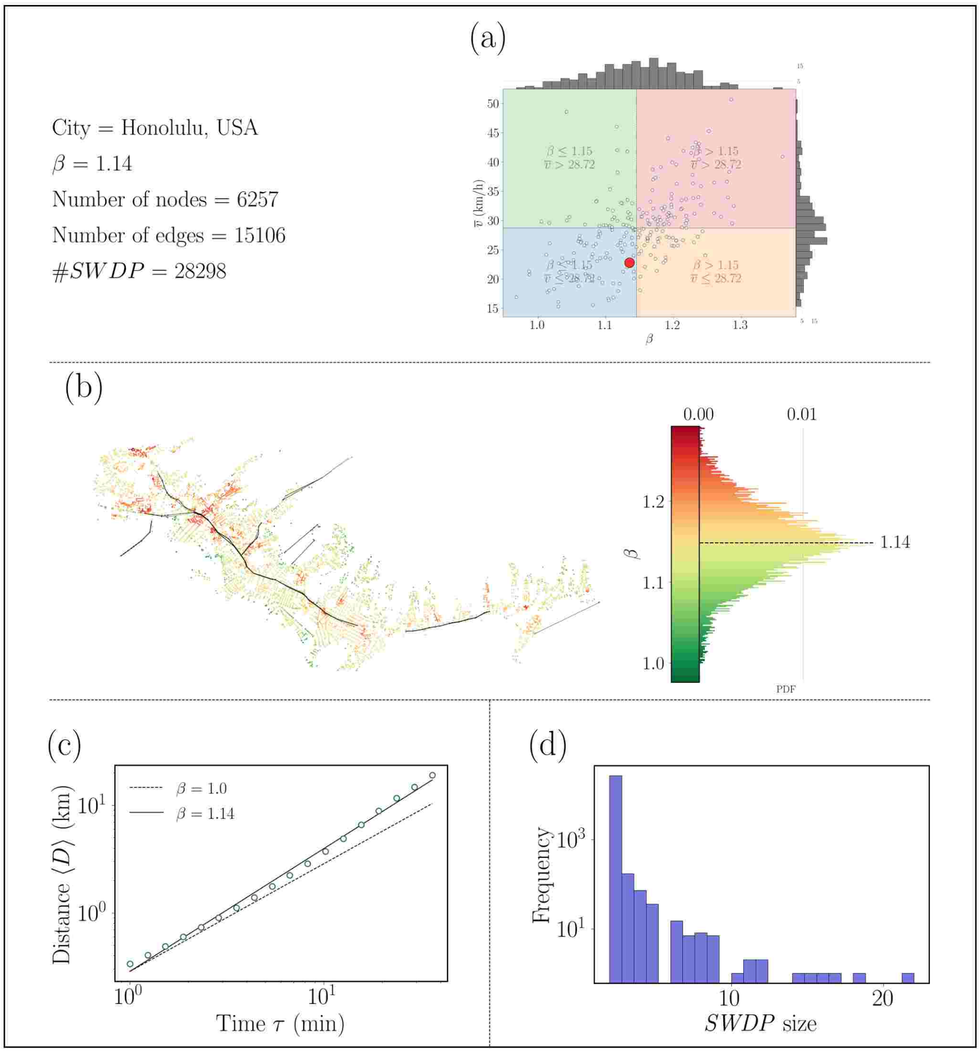

Fig. S75. Methodological sheet - Honolulu, USA. In (a) Each point represents a city, with mean exponent (β), on the x-axis, and mean speed $\overline{v}$ obtained in all trips made to calculate the exponent on the axis y. The histograms of the values of β and $\overline{v}$ are shown on the axes in the upper and right corners, respectively. The graph was segmented into four quadrants, in which the division is performed by the mean values of β and $\overline{v}$. The quadrants were colored and annotated according to the division criteria. The red dot represents the location of Honolulu, USA. In (b) taking all the nodes of Honolulu, USA as origin, the dots are colored as a function of their exponent value and their color is quantified by the color bar in the center. The longest segments without a deceleration point (SWDP) are plotted in black. The probability density function of the $\beta's$ for each experiment is shown on the left of the color scale Figure (c) shows the mean correlation curve between time τ and the distance $\langle D \rangle$. The black traced line represents the exponent equal to 1.0. Figure (d) shows the distribution of SWDP sizes in number of nodes per frequency of occurrence.

Houston, USA

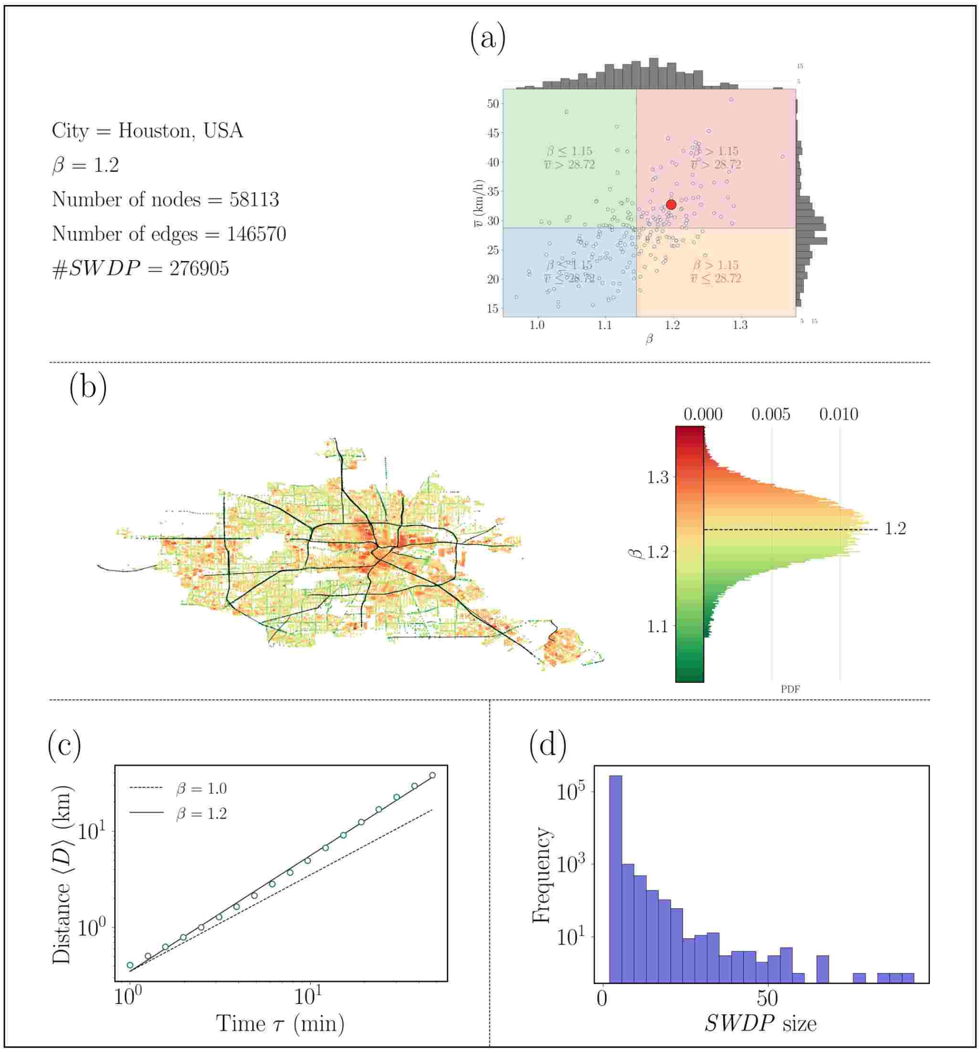

Fig. S76. Methodological sheet - Houston, USA. In (a) Each point represents a city, with mean exponent (β), on the x-axis, and mean speed $\overline{v}$ obtained in all trips made to calculate the exponent on the axis y. The histograms of the values of β and $\overline{v}$ are shown on the axes in the upper and right corners, respectively. The graph was segmented into four quadrants, in which the division is performed by the mean values of β and $\overline{v}$. The quadrants were colored and annotated according to the division criteria. The red dot represents the location of Houston, USA. In (b) taking all the nodes of Houston, USA as origin, the dots are colored as a function of their exponent value and their color is quantified by the color bar in the center. The longest segments without a deceleration point (SWDP) are plotted in black. The probability density function of the $\beta' s$ for each experiment is shown on the left of the color scale Figure (c) shows the mean correlation curve between time τ and the distance $\langle D \rangle$. The black traced line represents the exponent equal to 1.0. Figure (d) shows the distribution of SWDP sizes in number of nodes per frequency of occurrence.

Innsbruck, Austria

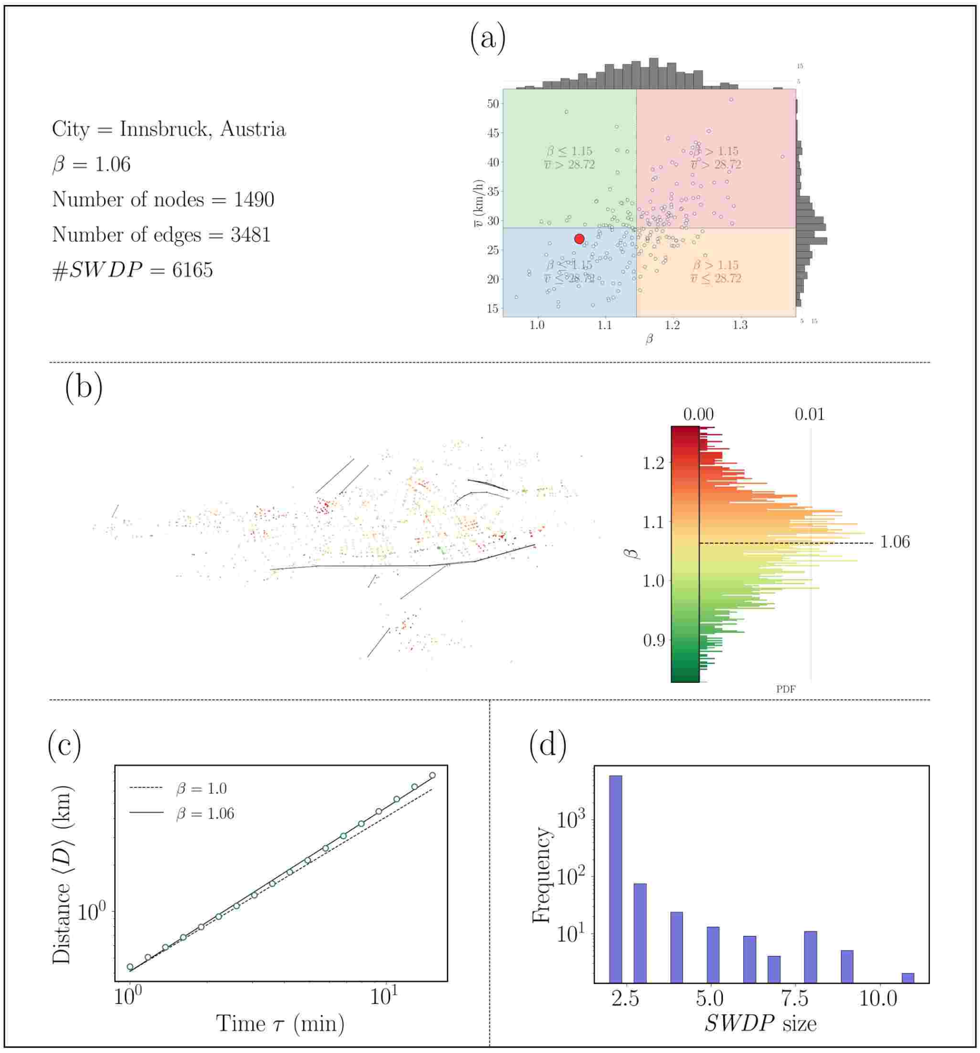

Fig. S77. Methodological sheet - Innsbruck, Austria. In (a) Each point represents a city, with mean exponent (β), on the x-axis, and mean speed $\overline{v}$ obtained in all trips made to calculate the exponent on the axis y. The histograms of the values of β and $\overline{v}$ are shown on the axes in the upper and right corners, respectively. The graph was segmented into four quadrants, in which the division is performed by the mean values of β and $\overline{v}$. The quadrants were colored and annotated according to the division criteria. The red dot represents the location of Innsbruck, Austria. In (b) taking all the nodes of Innsbruck, Austria as origin, the dots are colored as a function of their exponent value and their color is quantified by the color bar in the center. The longest segments without a deceleration point (SWDP) are plotted in black. The probability density function of the $\beta's$ for each experiment is shown on the left of the color scale Figure (c) shows the mean correlation curve between time τ and the distance $\langle D \rangle$. The black traced line represents the exponent equal to 1.0. Figure (d) shows the distribution of SWDP sizes in number of nodes per frequency of occurrence.

Joensuu, Finland

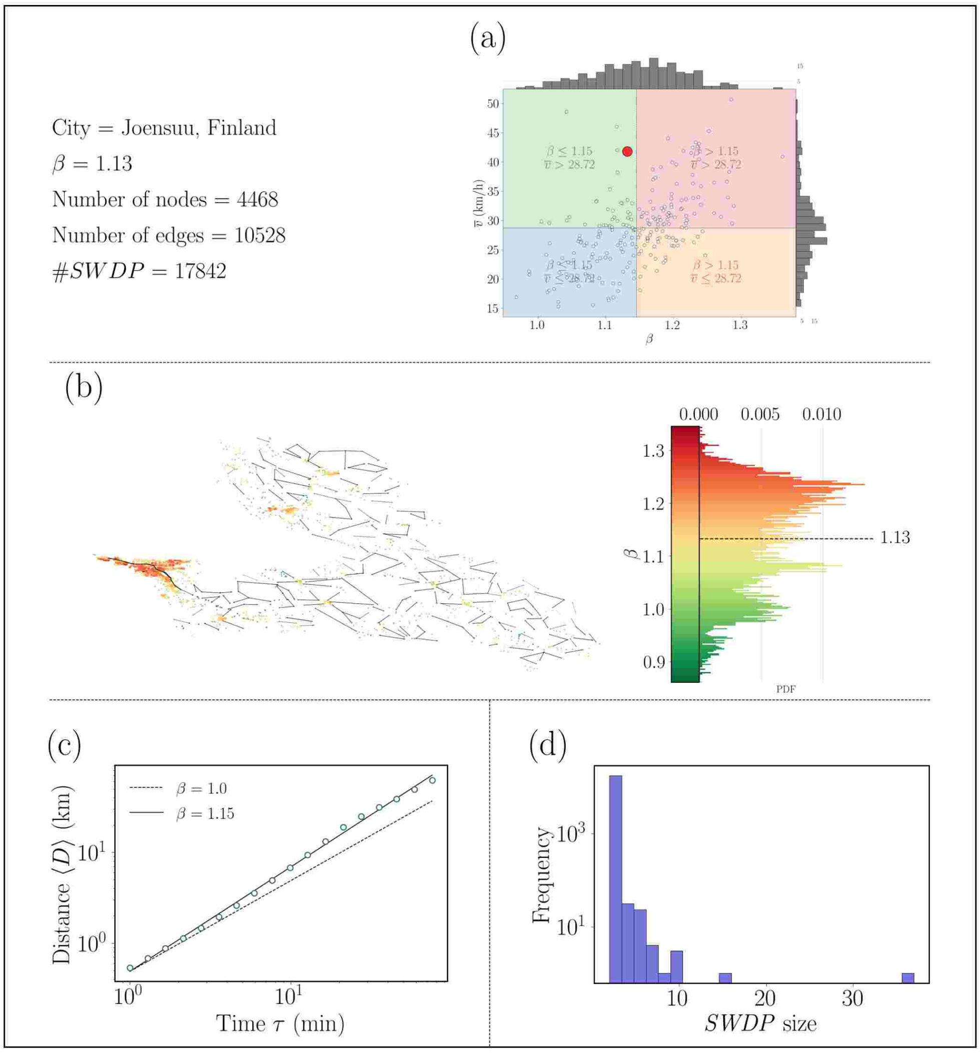

Fig. S78. Methodological sheet - Joensuu, Finland. In (a) Each point represents a city, with mean exponent (β), on the x-axis, and mean speed $\overline{v}$ obtained in all trips made to calculate the exponent on the axis y. The histograms of the values of β and $\overline{v}$ are shown on the axes in the upper and right corners, respectively. The graph was segmented into four quadrants, in which the division is performed by the mean values of β and $\overline{v}$. The quadrants were colored and annotated according to the division criteria. The red dot represents the location of Joensuu, Finland. In (b) taking all the nodes of Joensuu, Finland as origin, the dots are colored as a function of their exponent value and their color is quantified by the color bar in the center. The longest segments without a deceleration point (SWDP) are plotted in black. The probability density function of the $\beta's$ for each experiment is shown on the left of the color scale Figure (c) shows the mean correlation curve between time τ and the distance $\langle D \rangle$. The black traced line represents the exponent equal to 1.0. Figure (d) shows the distribution of SWDP sizes in number of nodes per frequency of occurrence.

Joinville, Brasil

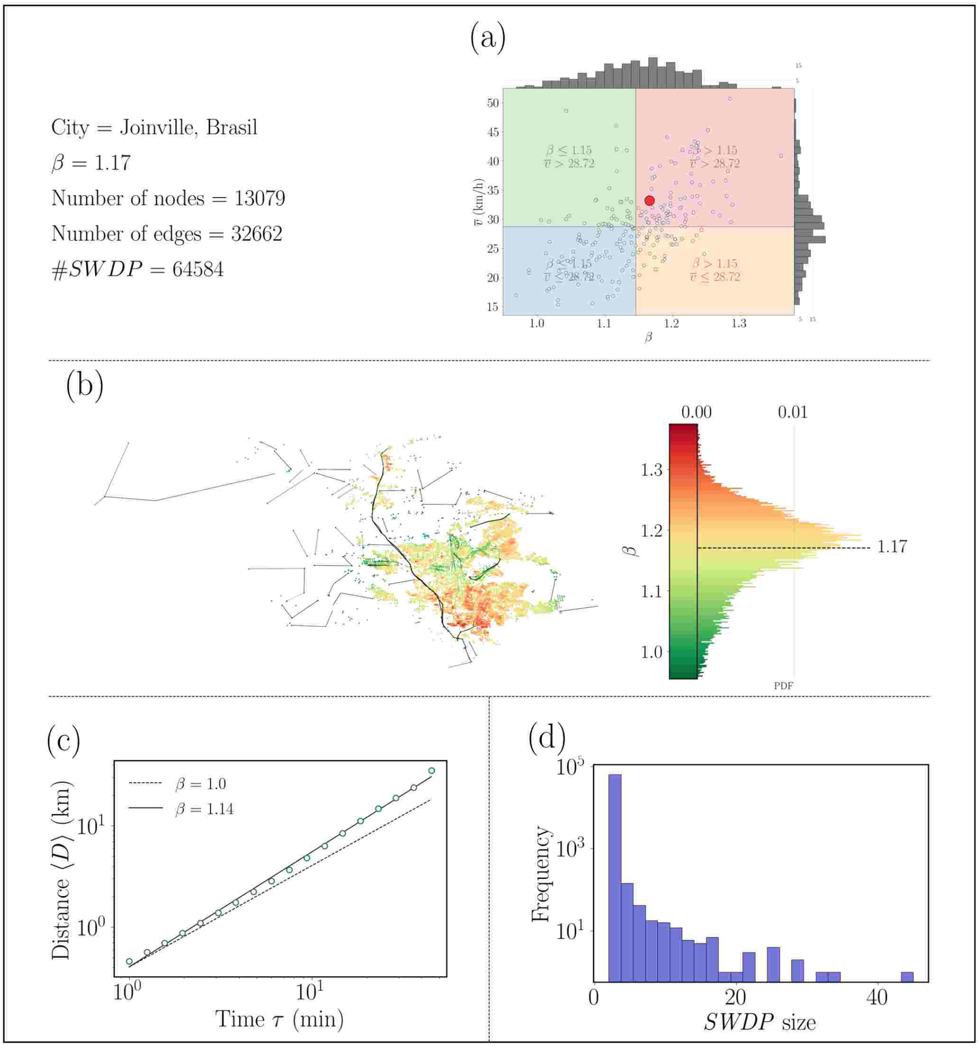

Fig. S79. Methodological sheet - Joinville, Brasil. In (a) Each point represents a city, with mean exponent (β), on the x-axis, and mean speed $\overline{v}$ obtained in all trips made to calculate the exponent on the axis y. The histograms of the values of β and $\overline{v}$ are shown on the axes in the upper and right corners, respectively. The graph was segmented into four quadrants, in which the division is performed by the mean values of β and $\overline{v}$. The quadrants were colored and annotated according to the division criteria. The red dot represents the location of Joinville, Brasil. In (b) taking all the nodes of Joinville, Brasil as origin, the dots are colored as a function of their exponent value and their color is quantified by the color bar in the center. The longest segments without a deceleration point (SWDP) are plotted in black. The probability density function of the $\beta's$ for each experiment is shown on the left of the color scale Figure (c) shows the mean correlation curve between time τ and the distance $\langle D \rangle$. The black traced line represents the exponent equal to 1.0. Figure (d) shows the distribution of SWDP sizes in number of nodes per frequency of occurrence.

João Pessoa, Brasil

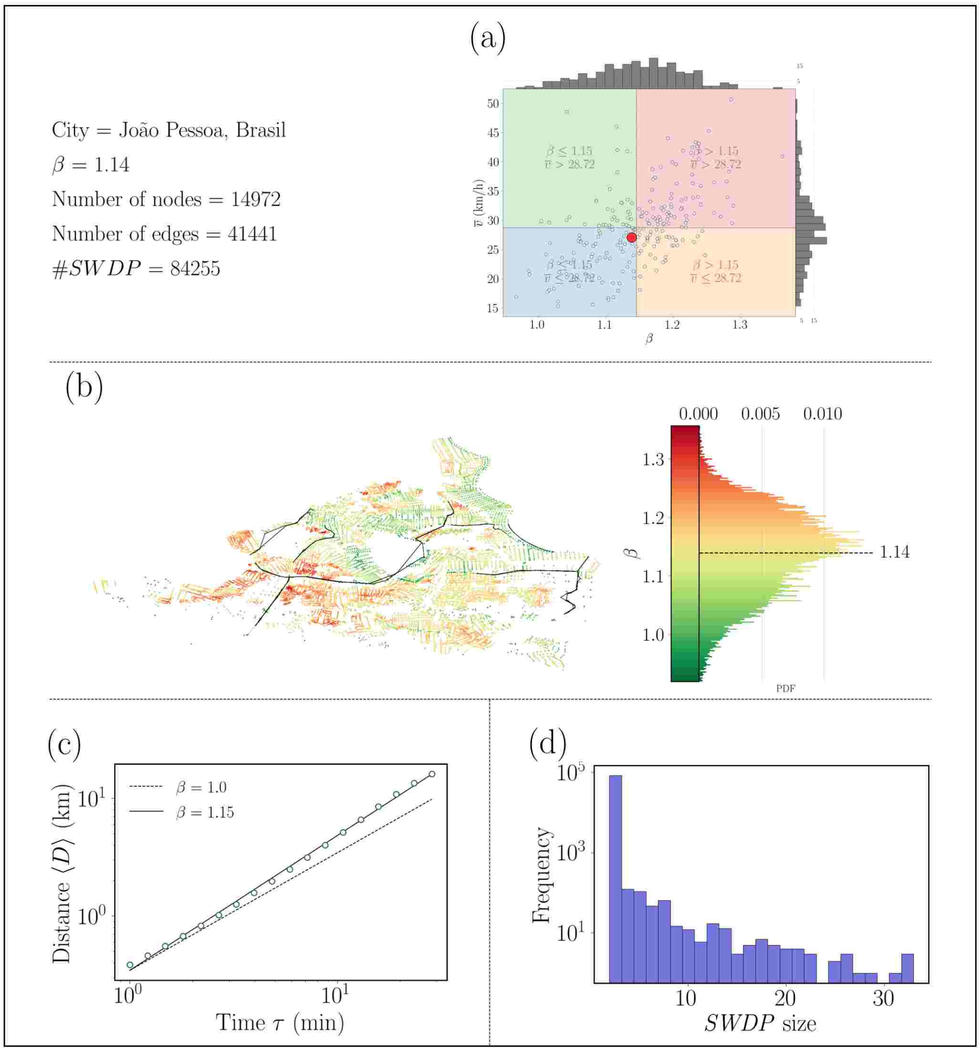

Fig. S80. Methodological sheet - João Pessoa, Brasil. In (a) Each point represents a city, with mean exponent (β), on the x-axis, and mean speed $\overline{v}$ obtained in all trips made to calculate the exponent on the axis y. The histograms of the values of β and $\overline{v}$ are shown on the axes in the upper and right corners, respectively. The graph was segmented into four quadrants, in which the division is performed by the mean values of β and $\overline{v}$. The quadrants were colored and annotated according to the division criteria. The red dot represents the location of João Pessoa, Brasil. In (b) taking all the nodes of João Pessoa, Brasil as origin, the dots are colored as a function of their exponent value and their color is quantified by the color bar in the center. The longest segments without a deceleration point (SWDP) are plotted in black. The probability density function of the $\beta's$ for each experiment is shown on the left of the color scale Figure (c) shows the mean correlation curve between time τ and the distance $\langle D \rangle$. The black traced line represents the exponent equal to 1.0. Figure (d) shows the distribution of SWDP sizes in number of nodes per frequency of occurrence.

Kampala, Uganda

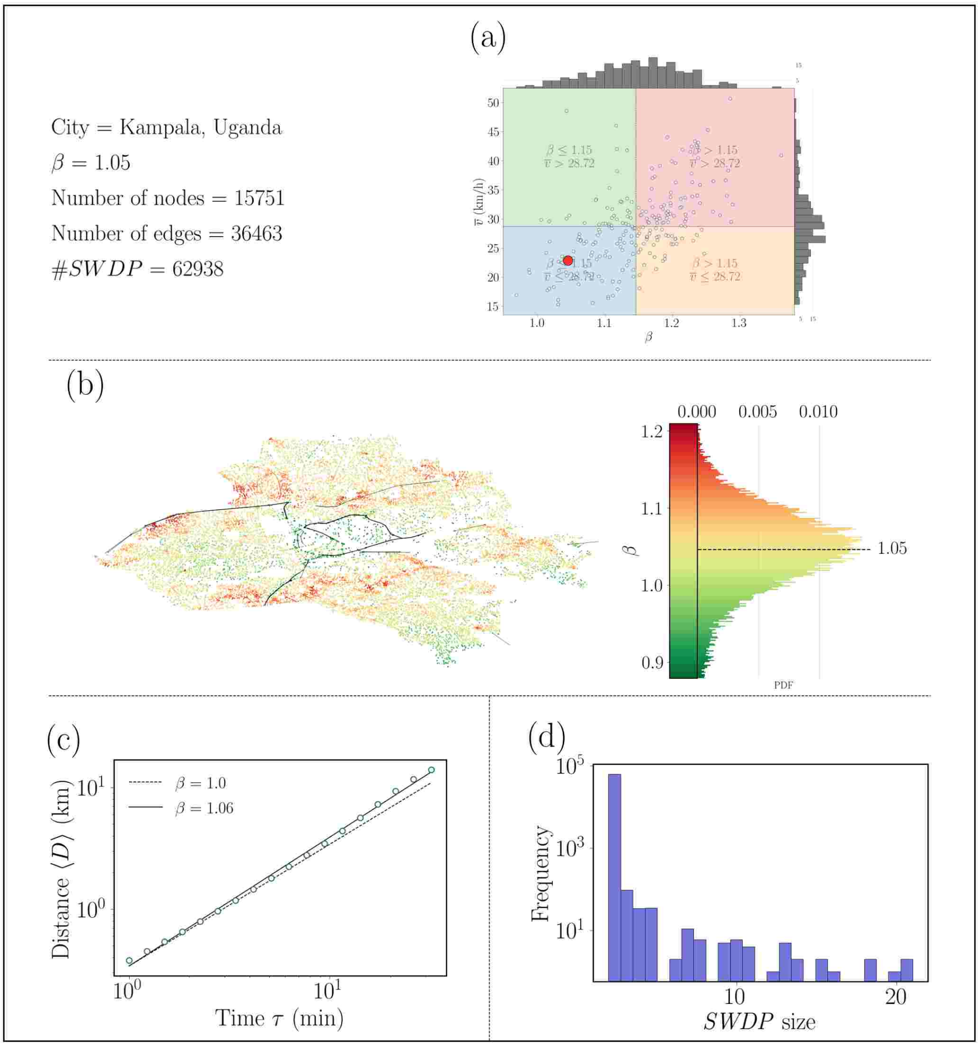

Fig. S81. Methodological sheet - Kampala, Uganda. In (a) Each point represents a city, with mean exponent (β), on the x-axis, and mean speed $\overline{v}$ obtained in all trips made to calculate the exponent on the axis y. The histograms of the values of β and $\overline{v}$ are shown on the axes in the upper and right corners, respectively. The graph was segmented into four quadrants, in which the division is performed by the mean values of β and $\overline{v}$. The quadrants were colored and annotated according to the division criteria. The red dot represents the location of Kampala, Uganda. In (b) taking all the nodes of Kampala, Uganda as origin, the dots are colored as a function of their exponent value and their color is quantified by the color bar in the center. The longest segments without a deceleration point (SWDP) are plotted in black. The probability density function of the $\beta's$ for each experiment is shown on the left of the color scale Figure (c) shows the mean correlation curve between time τ and the distance $\langle D \rangle$. The black traced line represents the exponent equal to 1.0. Figure (d) shows the distribution of SWDP sizes in number of nodes per frequency of occurrence.

Kansas City,USA

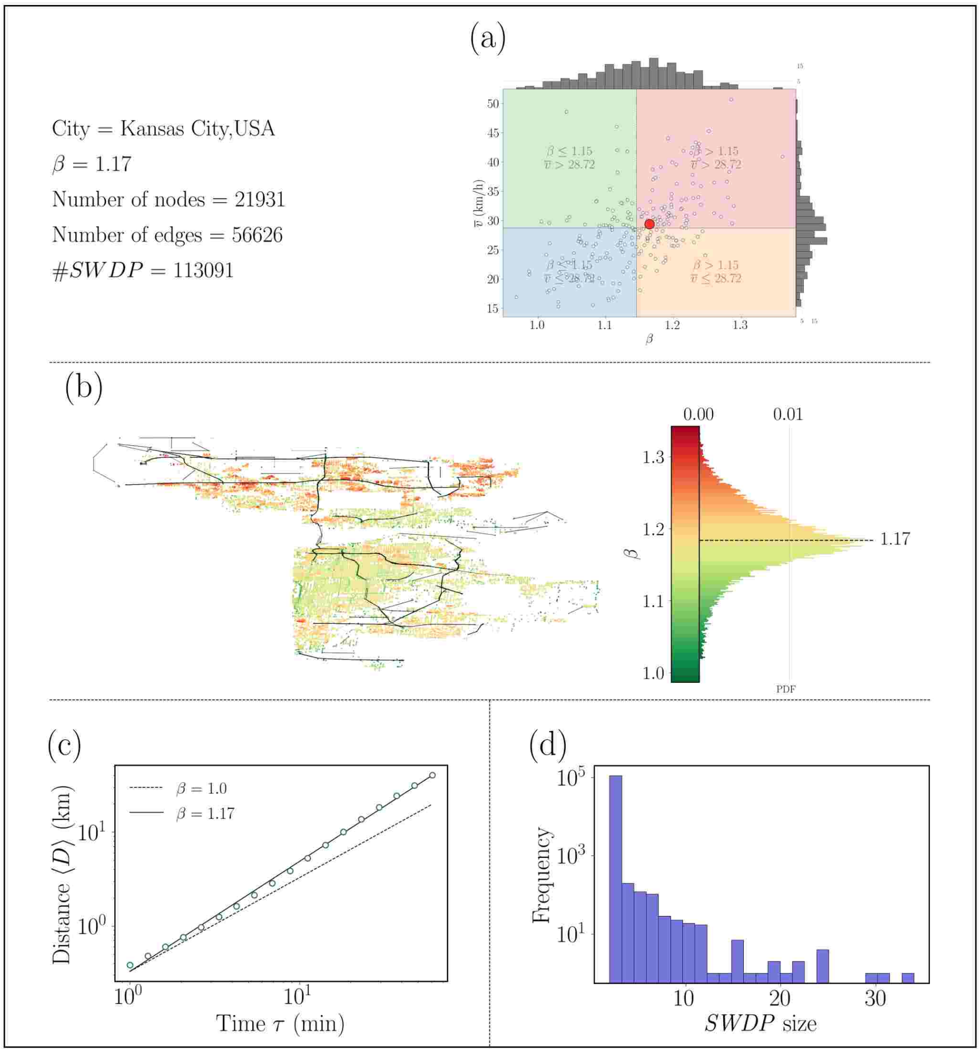

Fig. S82. Methodological sheet - Kansas City,USA. In (a) Each point represents a city, with mean exponent (β), on the x-axis, and mean speed $\overline{v}$ obtained in all trips made to calculate the exponent on the axis y. The histograms of the values of β and $\overline{v}$ are shown on the axes in the upper and right corners, respectively. The graph was segmented into four quadrants, in which the division is performed by the mean values of β and $\overline{v}$. The quadrants were colored and annotated according to the division criteria. The red dot represents the location of Kansas City,USA. In (b) taking all the nodes of Kansas City,USA as origin, the dots are colored as a function of their exponent value and their color is quantified by the color bar in the center. The longest segments without a deceleration point (SWDP) are plotted in black. The probability density function of the $\beta's$ for each experiment is shown on the left of the color scale Figure (c) shows the mean correlation curve between time τ and the distance $\langle D \rangle$. The black traced line represents the exponent equal to 1.0. Figure (d) shows the distribution of SWDP sizes in number of nodes per frequency of occurrence.

Kuala Lumpur, Malaysia

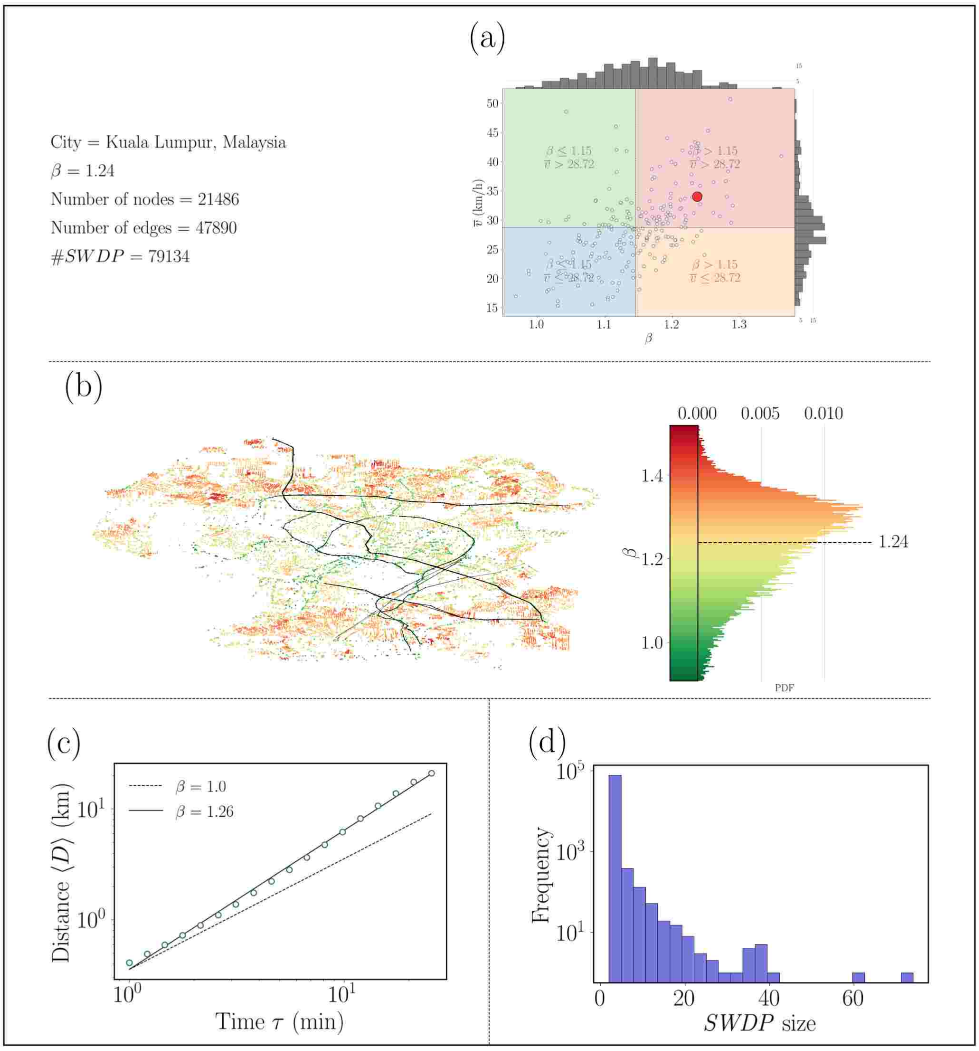

Fig. S83. Methodological sheet - Kuala Lumpur, Malaysia. In (a) Each point represents a city, with mean exponent (β), on the x-axis, and mean speed $\overline{v}$ obtained in all trips made to calculate the exponent on the axis y. The histograms of the values of β and $\overline{v}$ are shown on the axes in the upper and right corners, respectively. The graph was segmented into four quadrants, in which the division is performed by the mean values of β and $\overline{v}$. The quadrants were colored and annotated according to the division criteria. The red dot represents the location of Kuala Lumpur, Malaysia. In (b) taking all the nodes of Kuala Lumpur, Malaysia as origin, the dots are colored as a function of their exponent value and their color is quantified by the color bar in the center. The longest segments without a deceleration point (SWDP) are plotted in black. The probability density function of the $\beta's$ for each experiment is shown on the left of the color scale Figure (c) shows the mean correlation curve between time τ and the distance $\langle D \rangle$. The black traced line represents the exponent equal to 1.0. Figure (d) shows the distribution of SWDP sizes in number of nodes per frequency of occurrence.

Kyoto, Kyoto Prefecture, Japan

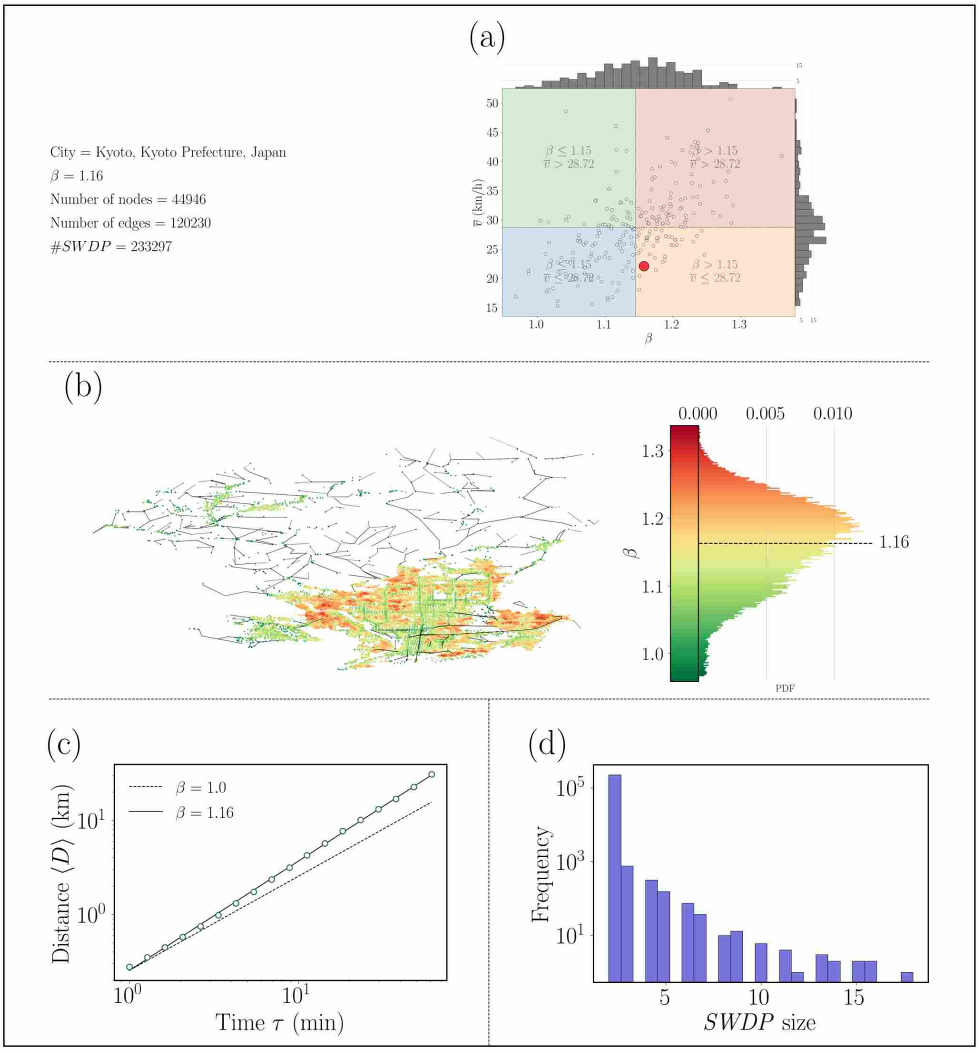

Fig. S84. Methodological sheet - Kyoto, Kyoto Prefecture, Japan. In (a) Each point represents a city, with mean exponent (β), on the x-axis, and mean speed $\overline{v}$ obtained in all trips made to calculate the exponent on the axis y. The histograms of the values of β and $\overline{v}$ are shown on the axes in the upper and right corners, respectively. The graph was segmented into four quadrants, in which the division is performed by the mean values of β and $\overline{v}$. The quadrants were colored and annotated according to the division criteria. The red dot represents the location of Kyoto, Kyoto Prefecture, Japan. In (b) taking all the nodes of Kyoto, Kyoto Prefecture, Japan as origin, the dots are colored as a function of their exponent value and their color is quantified by the color bar in the center. The longest segments without a deceleration point (SWDP) are plotted in black. The probability density function of the $\beta's$ for each experiment is shown on the left of the color scale Figure (c) shows the mean correlation curve between time τ and the distance $\langle D \rangle$. The black traced line represents the exponent equal to 1.0. Figure (d) shows the distribution of SWDP sizes in number of nodes per frequency of occurrence.

La Plata, Argentina

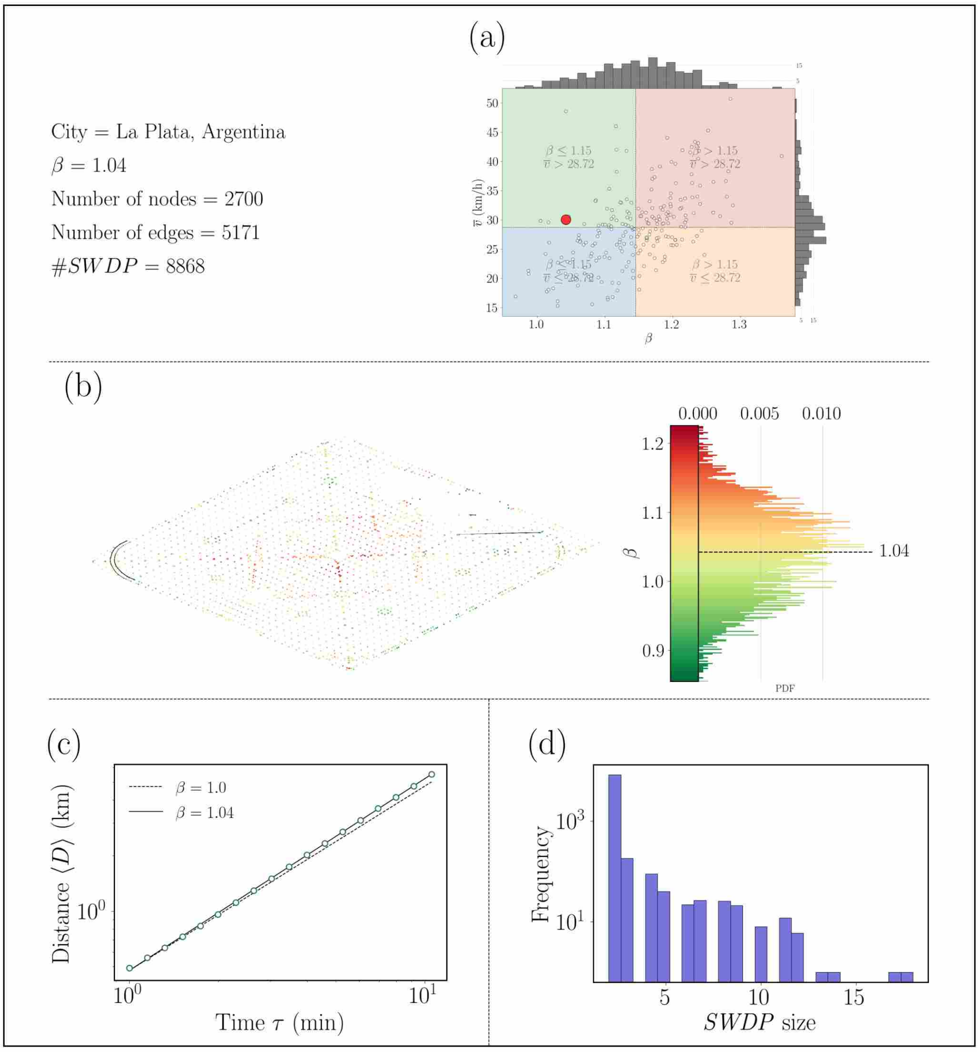

Fig. S85. Methodological sheet - La Plata, Argentina. In (a) Each point represents a city, with mean exponent (β), on the x-axis, and mean speed $\overline{v}$ obtained in all trips made to calculate the exponent on the axis y. The histograms of the values of β and $\overline{v}$ are shown on the axes in the upper and right corners, respectively. The graph was segmented into four quadrants, in which the division is performed by the mean values of β and $\overline{v}$. The quadrants were colored and annotated according to the division criteria. The red dot represents the location of La Plata, Argentina. In (b) taking all the nodes of La Plata, Argentina as origin, the dots are colored as a function of their exponent value and their color is quantified by the color bar in the center. The longest segments without a deceleration point (SWDP) are plotted in black. The probability density function of the $\beta's$ for each experiment is shown on the left of the color scale Figure (c) shows the mean correlation curve between time τ and the distance $\langle D \rangle$. The black traced line represents the exponent equal to 1.0. Figure (d) shows the distribution of SWDP sizes in number of nodes per frequency of occurrence.

Las Vegas, USA

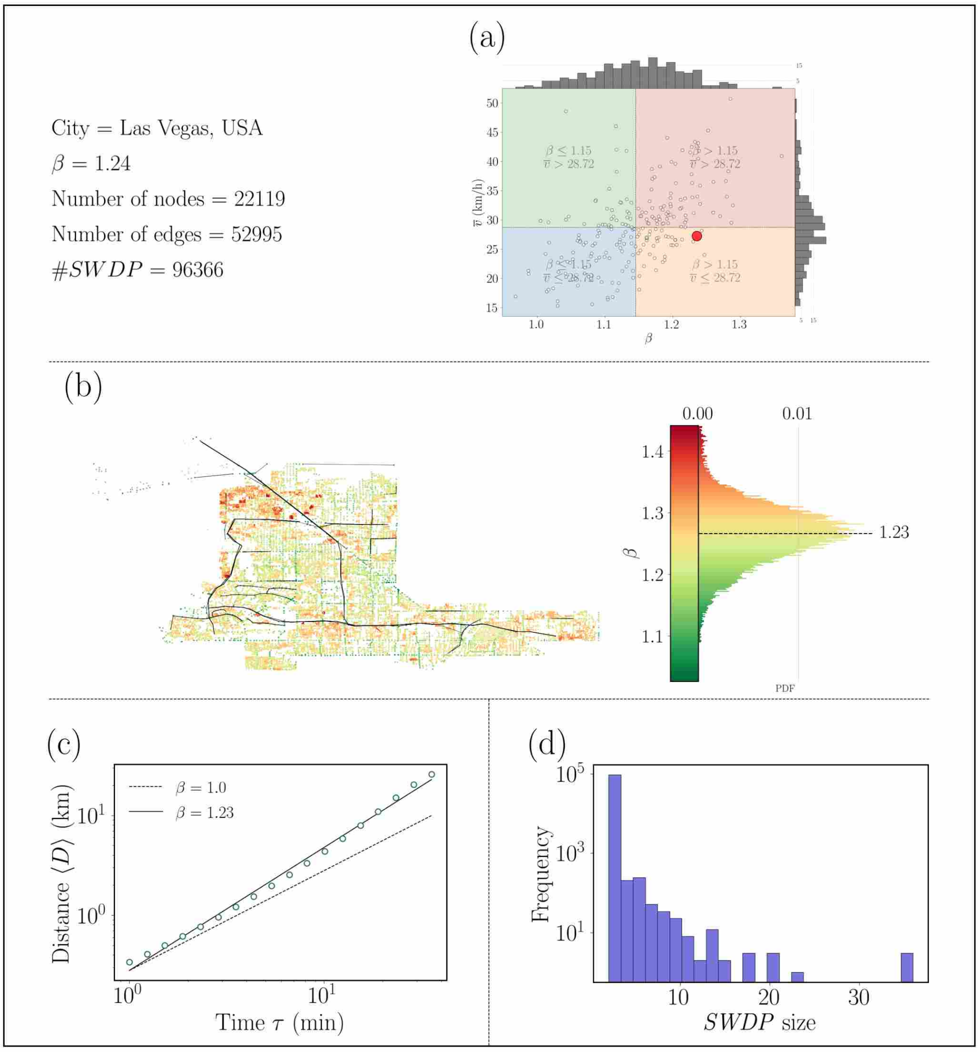

Fig. S86. Methodological sheet - Las Vegas, USA. In (a) Each point represents a city, with mean exponent (β), on the x-axis, and mean speed $\overline{v}$ obtained in all trips made to calculate the exponent on the axis y. The histograms of the values of β and $\overline{v}$ are shown on the axes in the upper and right corners, respectively. The graph was segmented into four quadrants, in which the division is performed by the mean values of β and $\overline{v}$. The quadrants were colored and annotated according to the division criteria. The red dot represents the location of Las Vegas, USA. In (b) taking all the nodes of Las Vegas, USA as origin, the dots are colored as a function of their exponent value and their color is quantified by the color bar in the center. The longest segments without a deceleration point (SWDP) are plotted in black. The probability density function of the $\beta's$ for each experiment is shown on the left of the color scale Figure (c) shows the mean correlation curve between time τ and the distance $\langle D \rangle$. The black traced line represents the exponent equal to 1.0. Figure (d) shows the distribution of SWDP sizes in number of nodes per frequency of occurrence.

Lisboa, Portugal

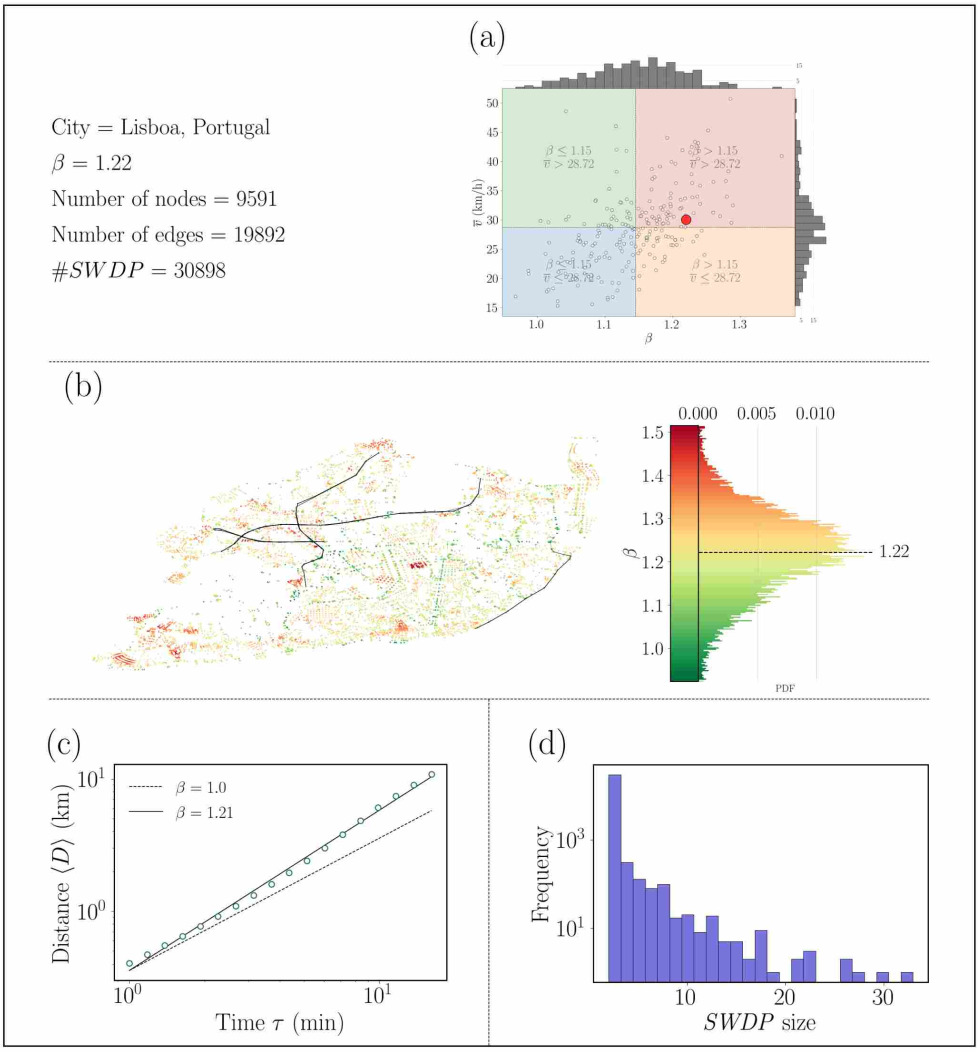

Fig. S87. Methodological sheet - Lisboa, Portugal. In (a) Each point represents a city, with mean exponent (β), on the x-axis, and mean speed $\overline{v}$ obtained in all trips made to calculate the exponent on the axis y. The histograms of the values of β and $\overline{v}$ are shown on the axes in the upper and right corners, respectively. The graph was segmented into four quadrants, in which the division is performed by the mean values of β and $\overline{v}$. The quadrants were colored and annotated according to the division criteria. The red dot represents the location of Lisboa, Portugal. In (b) taking all the nodes of Lisboa, Portugal as origin, the dots are colored as a function of their exponent value and their color is quantified by the color bar in the center. The longest segments without a deceleration point (SWDP) are plotted in black. The probability density function of the $\beta's$ for each experiment is shown on the left of the color scale Figure (c) shows the mean correlation curve between time τ and the distance $\langle D\rangle$. The black traced line represents the exponent equal to 1.0. Figure (d) shows the distribution of SWDP sizes in number of nodes per frequency of occurrence.

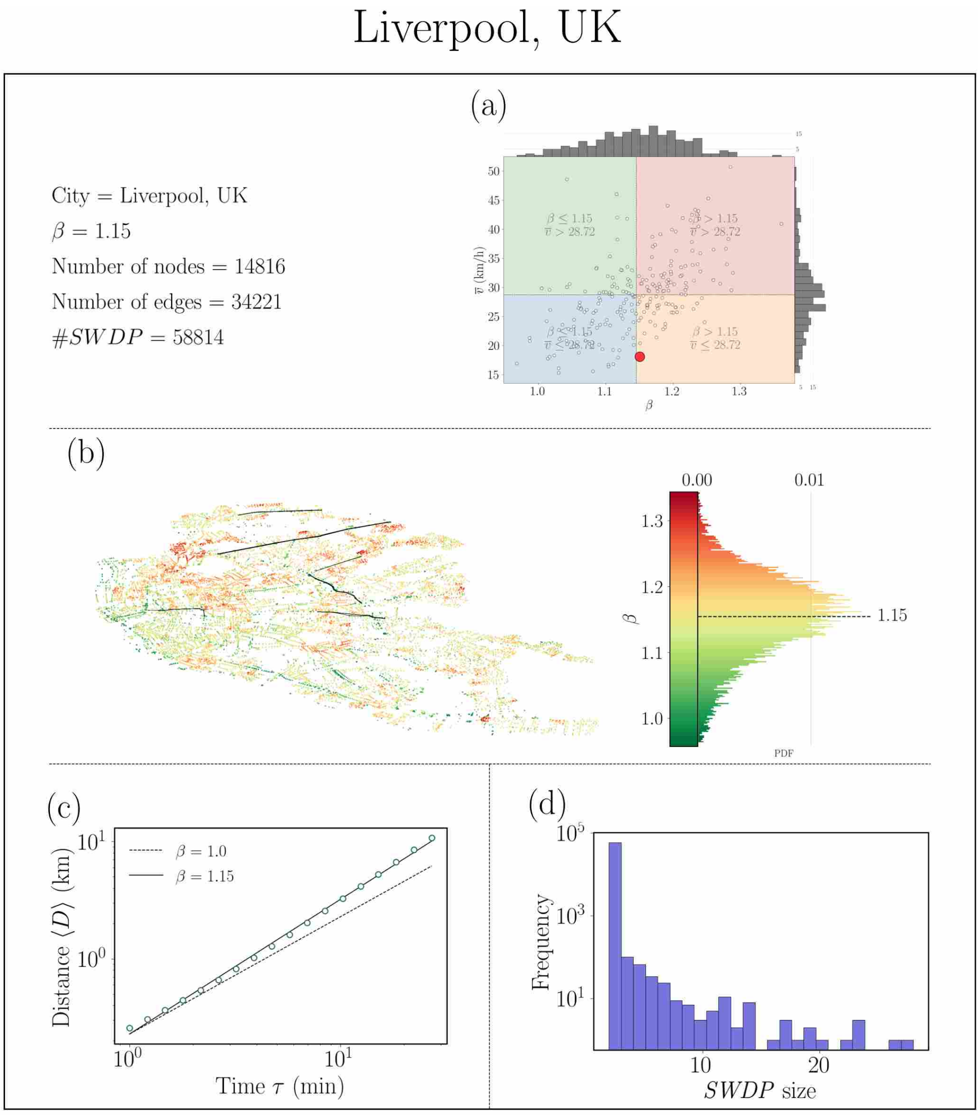

Fig. S88. Methodological sheet - Liverpool, UK. In (a) Each point represents a city, with mean exponent (β), on the x-axis, and mean speed $\overline{v}$ obtained in all trips made to calculate the exponent on the axis y. The histograms of the values of β and $\overline{v}$ are shown on the axes in the upper and right corners, respectively. The graph was segmented into four quadrants, in which the division is performed by the mean values of β and $\overline{v}$. The quadrants were colored and annotated according to the division criteria. The red dot represents the location of Liverpool, UK. In (b) taking all the nodes of Liverpool, UK as origin, the dots are colored as a function of their exponent value and their color is quantified by the color bar in the center. The longest segments without a deceleration point (SWDP) are plotted in black. The probability density function of the $\beta's$ for each experiment is shown on the left of the color scale Figure (c) shows the mean correlation curve between time τ and the distance $\langle D \rangle$. The black traced line represents the exponent equal to 1.0. Figure (d) shows the distribution of SWDP sizes in number of nodes per frequency of occurrence.

Ljubljana, Slovenia

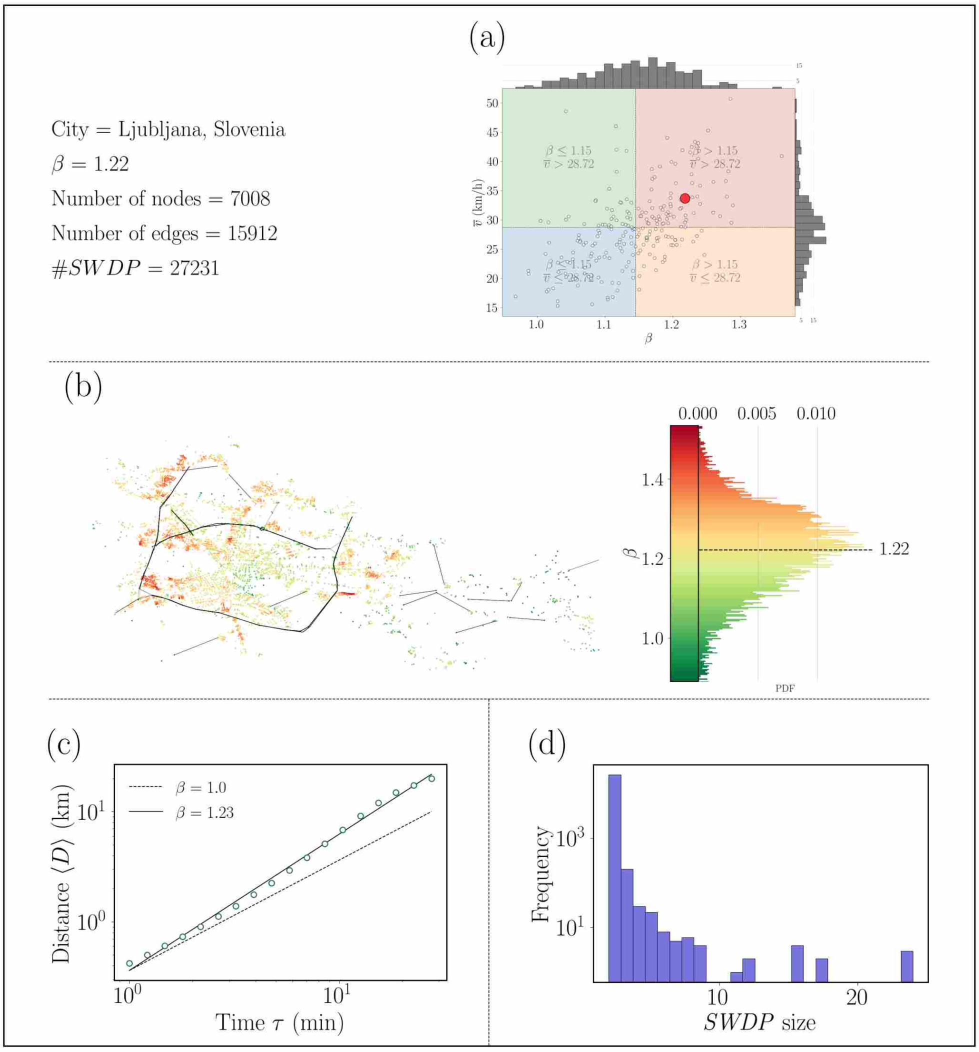

Fig. S89. Methodological sheet - Ljubljana, Slovenia. In (a) Each point represents a city, with mean exponent (β), on the x-axis, and mean speed $\overline{v}$ obtained in all trips made to calculate the exponent on the axis y. The histograms of the values of β and $\overline{v}$ are shown on the axes in the upper and right corners, respectively. The graph was segmented into four quadrants, in which the division is performed by the mean values of β and $\overline{v}$. The quadrants were colored and annotated according to the division criteria. The red dot represents the location of Ljubljana, Slovenia. In (b) taking all the nodes of Ljubljana, Slovenia as origin, the dots are colored as a function of their exponent value and their color is quantified by the color bar in the center. The longest segments without a deceleration point (SWDP) are plotted in black. The probability density function of the $\beta's$ for each experiment is shown on the left of the color scale Figure (c) shows the mean correlation curve between time τ and the distance $\langle D \rangle$. The black traced line represents the exponent equal to 1.0. Figure (d) shows the distribution of SWDP sizes in number of nodes per frequency of occurrence.

London, UK

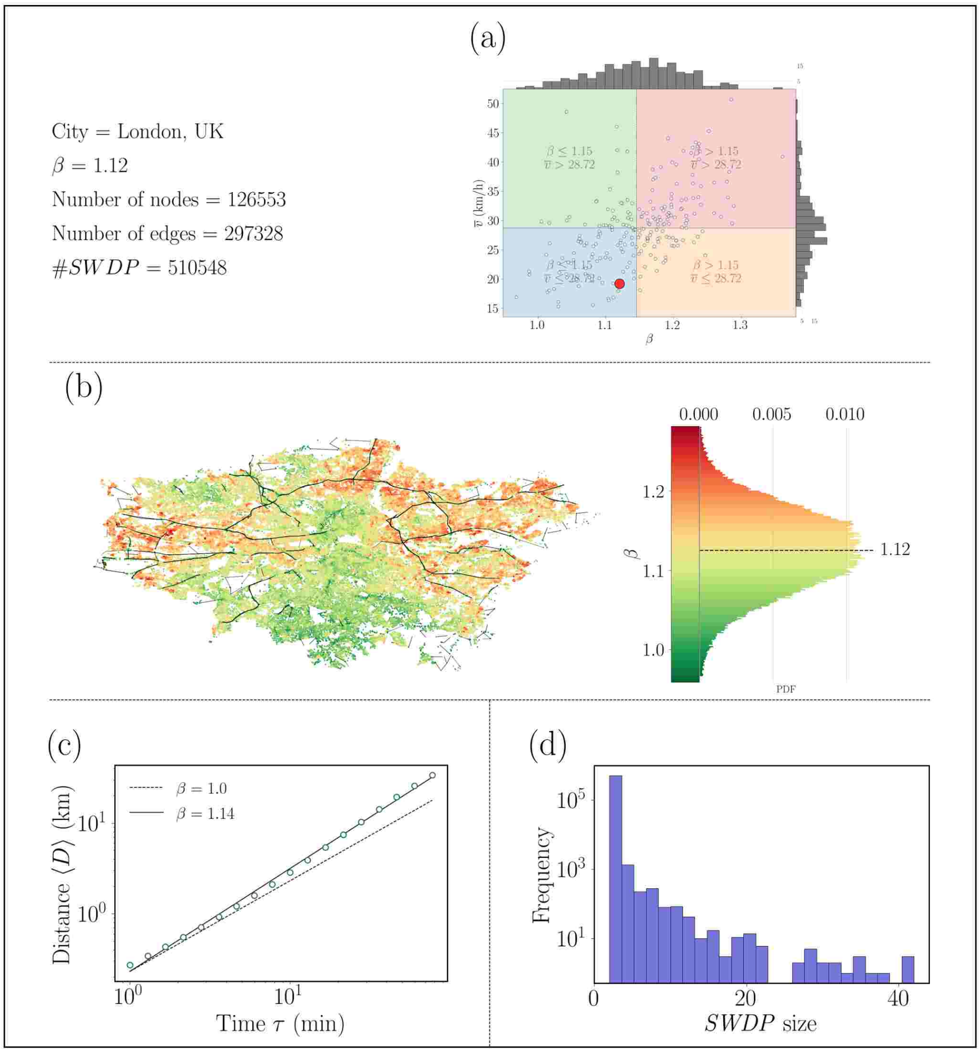

Fig. S90. Methodological sheet - London, UK. In (a) Each point represents a city, with mean exponent (β), on the x-axis, and mean speed $\overline{v}$ obtained in all trips made to calculate the exponent on the axis y. The histograms of the values of β and $\overline{v}$ are shown on the axes in the upper and right corners, respectively. The graph was segmented into four quadrants, in which the division is performed by the mean values of β and $\overline{v}$. The quadrants were colored and annotated according to the division criteria. The red dot represents the location of London, UK. In (b) taking all the nodes of London, UK as origin, the dots are colored as a function of their exponent value and their color is quantified by the color bar in the center. The longest segments without a deceleration point (SWDP) are plotted in black. The probability density function of the $\beta' s$ for each experiment is shown on the left of the color scale Figure (c) shows the mean correlation curve between time τ and the distance $\langle D \rangle$. The black traced line represents the exponent equal to 1.0. Figure (d) shows the distribution of SWDP sizes in number of nodes per frequency of occurrence.

Los Angeles, USA

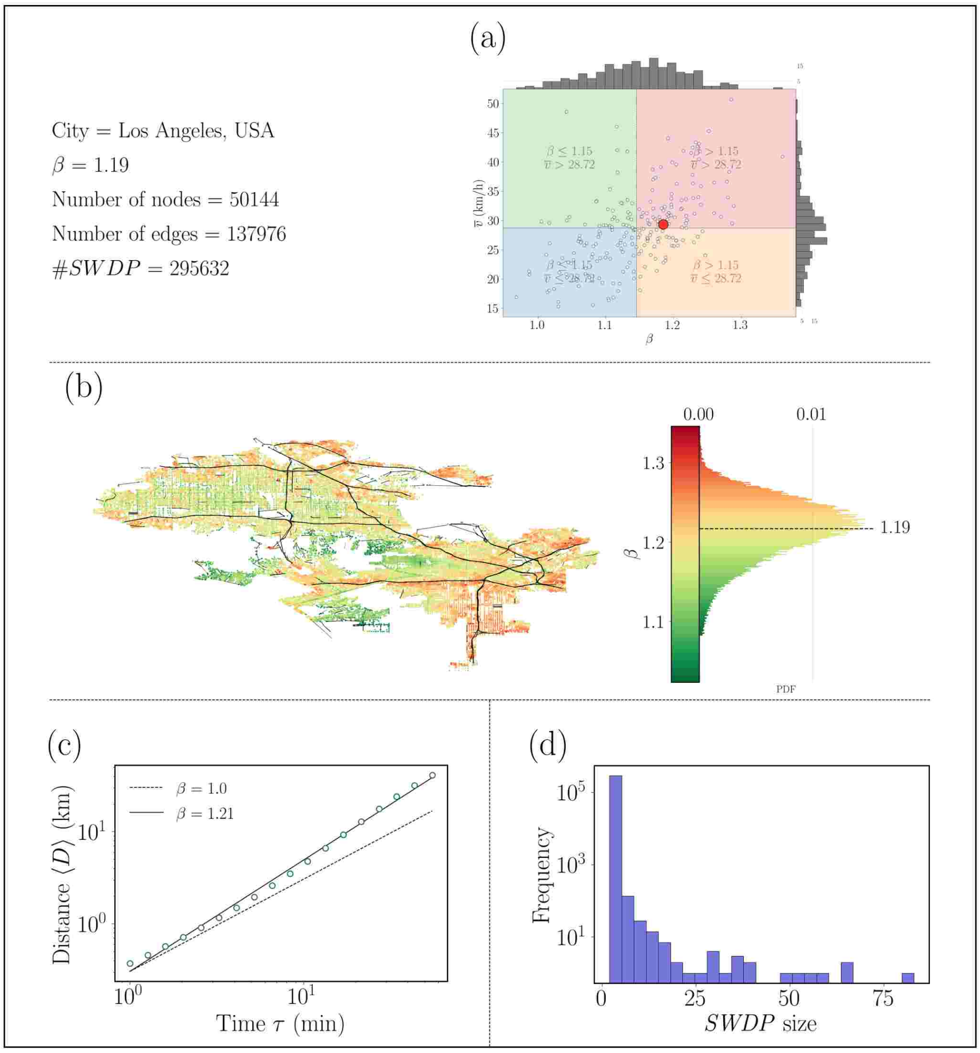

Fig. S91. Methodological sheet - Los Angeles, USA. In (a) Each point represents a city, with mean exponent (β), on the x-axis, and mean speed $\overline{v}$ obtained in all trips made to calculate the exponent on the axis y. The histograms of the values of β and $\overline{v}$ are shown on the axes in the upper and right corners, respectively. The graph was segmented into four quadrants, in which the division is performed by the mean values of β and $\overline{v}$. The quadrants were colored and annotated according to the division criteria. The red dot represents the location of Los Angeles, USA. In (b) taking all the nodes of Los Angeles, USA as origin, the dots are colored as a function of their exponent value and their color is quantified by the color bar in the center. The longest segments without a deceleration point (SWDP) are plotted in black. The probability density function of the $\beta's$ for each experiment is shown on the left of the color scale Figure (c) shows the mean correlation curve between time τ and the distance $\langle D \rangle$. The black traced line represents the exponent equal to 1.0. Figure (d) shows the distribution of SWDP sizes in number of nodes per frequency of occurrence.

Louisville, USA

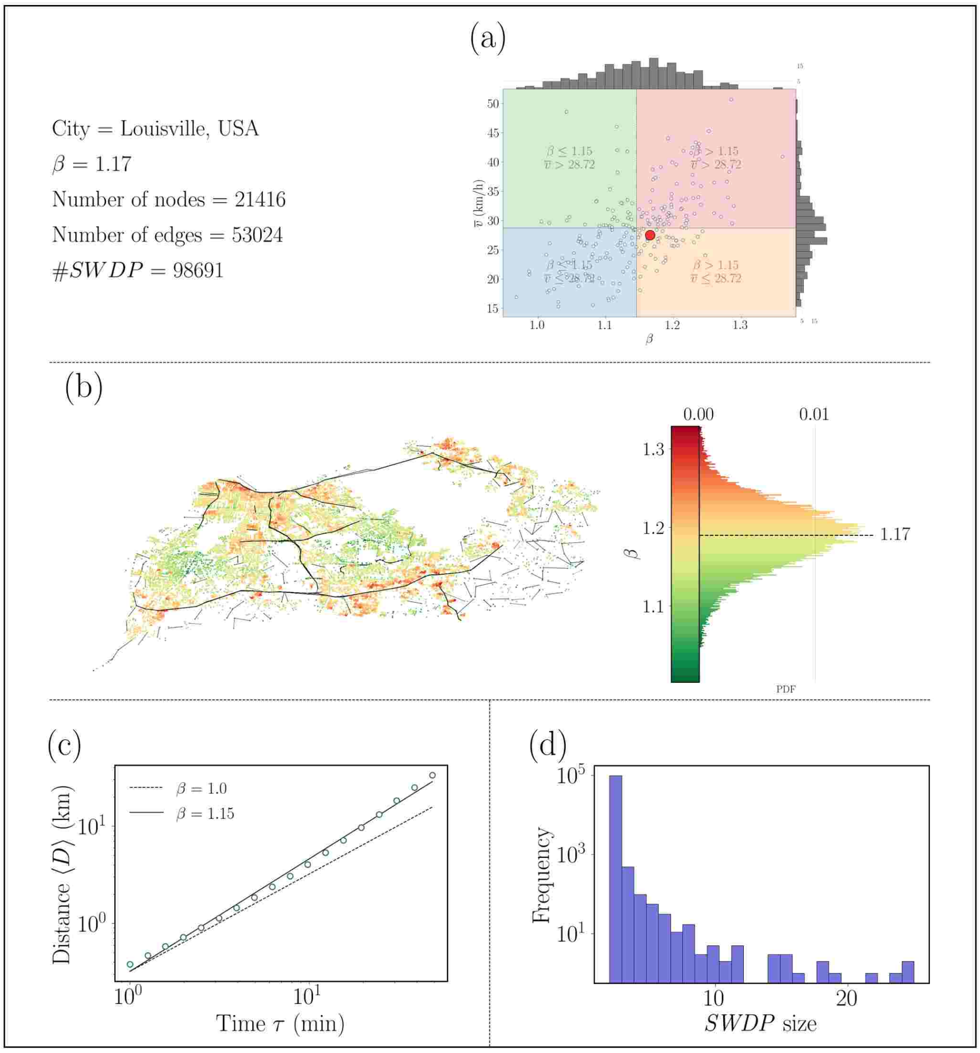

Fig. S92. Methodological sheet - Louisville, USA. In (a) Each point represents a city, with mean exponent (β), on the x-axis, and mean speed $\overline{v}$ obtained in all trips made to calculate the exponent on the axis y. The histograms of the values of β and $\overline{v}$ are shown on the axes in the upper and right corners, respectively. The graph was segmented into four quadrants, in which the division is performed by the mean values of β and $\overline{v}$. The quadrants were colored and annotated according to the division criteria. The red dot represents the location of Louisville, USA. In (b) taking all the nodes of Louisville, USA as origin, the dots are colored as a function of their exponent value and their color is quantified by the color bar in the center. The longest segments without a deceleration point (SWDP) are plotted in black. The probability density function of the $\beta's$ for each experiment is shown on the left of the color scale Figure (c) shows the mean correlation curve between time τ and the distance $\langle D \rangle$. The black traced line represents the exponent equal to 1.0. Figure (d) shows the distribution of SWDP sizes in number of nodes per frequency of occurrence.

Luanda, Angola

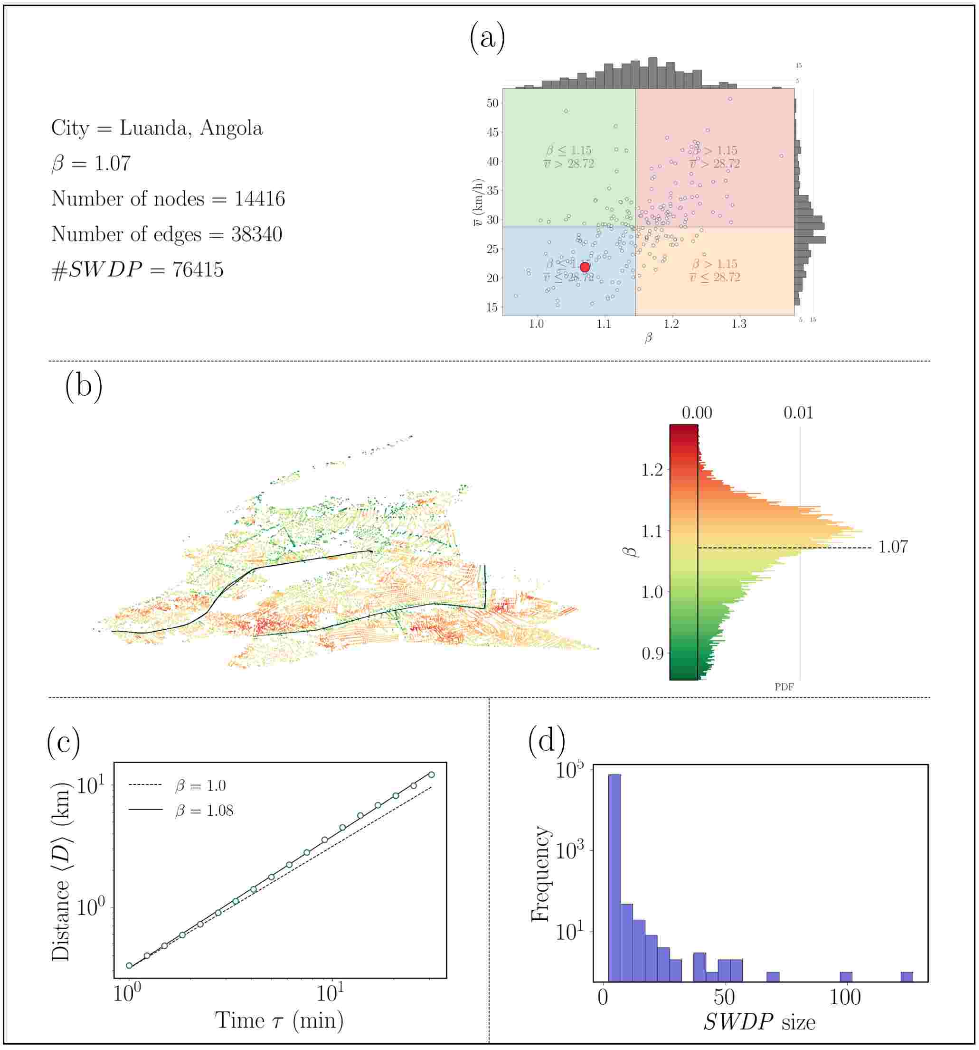

Fig. S93. Methodological sheet - Luanda, Angola. In (a) Each point represents a city, with mean exponent (β), on the x-axis, and mean speed $\overline{v}$ obtained in all trips made to calculate the exponent on the axis y. The histograms of the values of β and $\overline{v}$ are shown on the axes in the upper and right corners, respectively. The graph was segmented into four quadrants, in which the division is performed by the mean values of β and $\overline{v}$. The quadrants were colored and annotated according to the division criteria. The red dot represents the location of Luanda, Angola. In (b) taking all the nodes of Luanda, Angola as origin, the dots are colored as a function of their exponent value and their color is quantified by the color bar in the center. The longest segments without a deceleration point (SWDP) are plotted in black. The probability density function of the $\beta's$ for each experiment is shown on the left of the color scale Figure (c) shows the mean correlation curve between time τ and the distance $\langle D \rangle$. The black traced line represents the exponent equal to 1.0. Figure (d) shows the distribution of SWDP sizes in number of nodes per frequency of occurrence.

Lyon, France

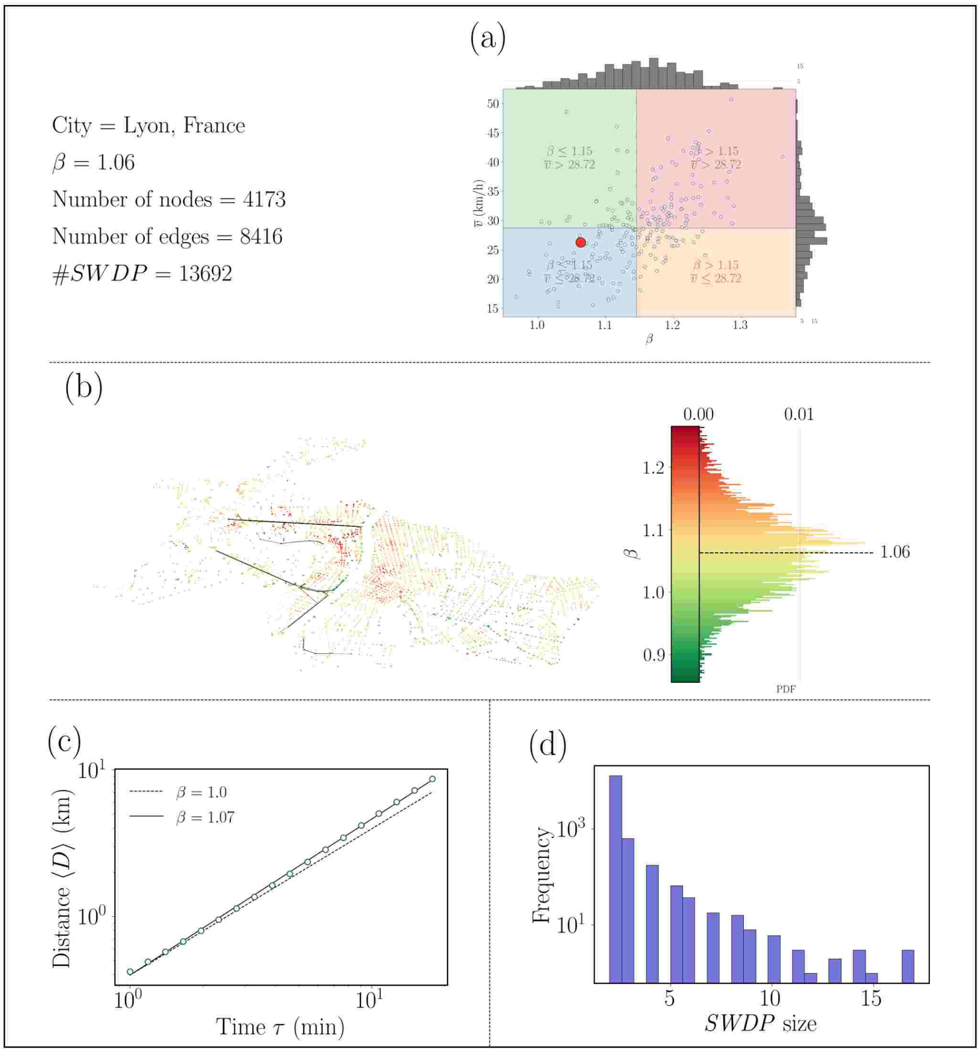

Fig. S94. Methodological sheet - Lyon, France. In (a) Each point represents a city, with mean exponent (β), on the x-axis, and mean speed $\overline{v}$ obtained in all trips made to calculate the exponent on the axis y. The histograms of the values of β and $\overline{v}$ are shown on the axes in the upper and right corners, respectively. The graph was segmented into four quadrants, in which the division is performed by the mean values of β and $\overline{v}$. The quadrants were colored and annotated according to the division criteria. The red dot represents the location of Lyon, France. In (b) taking all the nodes of Lyon, France as origin, the dots are colored as a function of their exponent value and their color is quantified by the color bar in the center. The longest segments without a deceleration point (SWDP) are plotted in black. The probability density function of the $\beta's$ for each experiment is shown on the left of the color scale Figure (c) shows the mean correlation curve between time τ and the distance $\langle D \rangle$. The black traced line represents the exponent equal to 1.0. Figure (d) shows the distribution of SWDP sizes in number of nodes per frequency of occurrence.

Maceió, Brasil

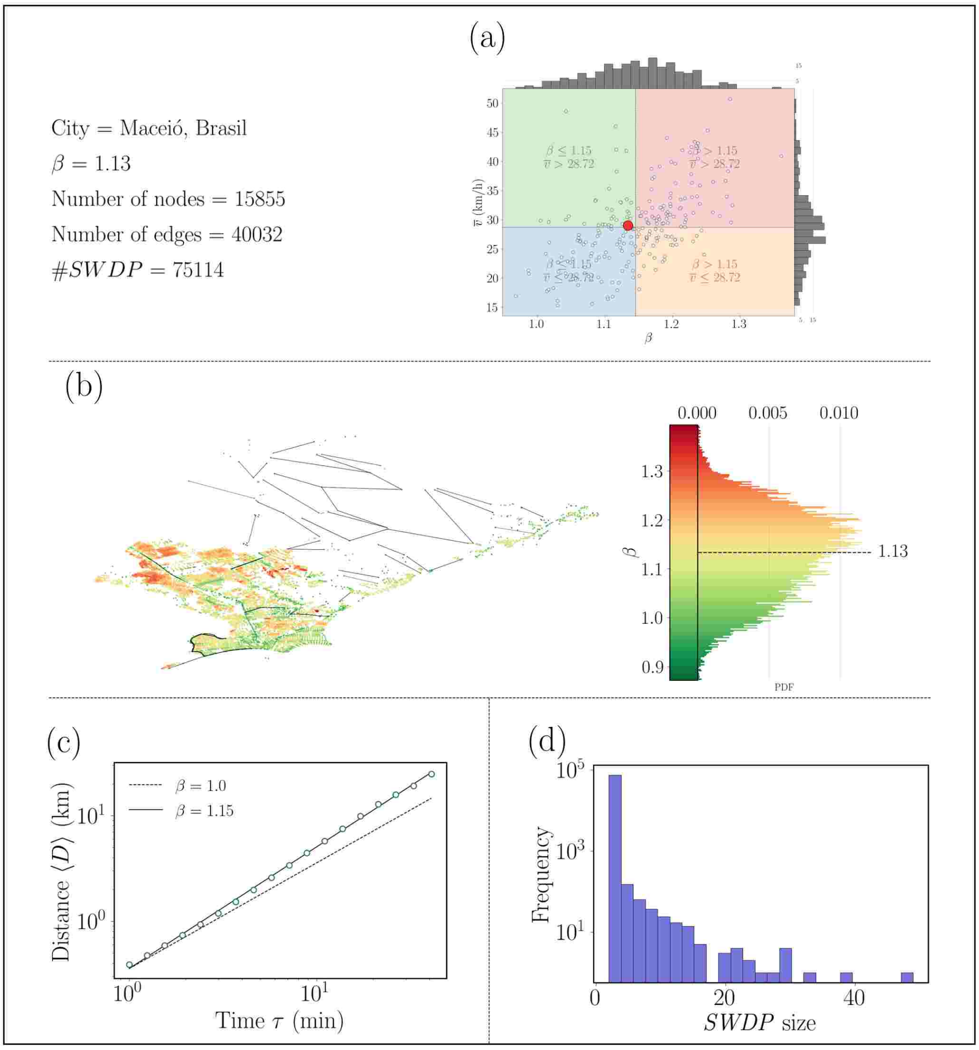

Fig. S95. Methodological sheet - Maceió, Brasil. In (a) Each point represents a city, with mean exponent (β), on the x-axis, and mean speed $\overline{v}$ obtained in all trips made to calculate the exponent on the axis y. The histograms of the values of β and $\overline{v}$ are shown on the axes in the upper and right corners, respectively. The graph was segmented into four quadrants, in which the division is performed by the mean values of β and $\overline{v}$. The quadrants were colored and annotated according to the division criteria. The red dot represents the location of Maceió, Brasil. In (b) taking all the nodes of Maceió, Brasil as origin, the dots are colored as a function of their exponent value and their color is quantified by the color bar in the center. The longest segments without a deceleration point (SWDP) are plotted in black. The probability density function of the $\beta's$ for each experiment is shown on the left of the color scale Figure (c) shows the mean correlation curve between time τ and the distance $\langle D \rangle$. The black traced line represents the exponent equal to 1.0. Figure (d) shows the distribution of SWDP sizes in number of nodes per frequency of occurrence.

Madrid, Spain

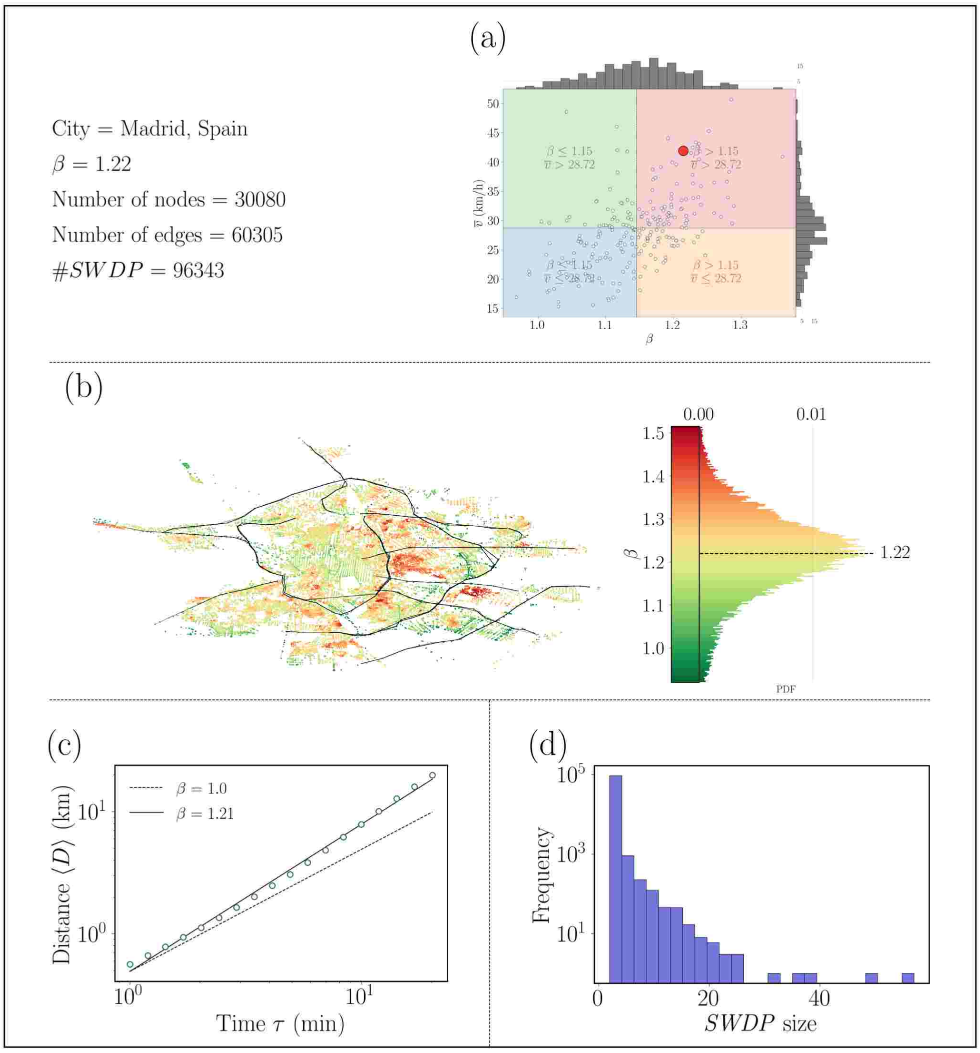

Fig. S96. Methodological sheet - Madrid, Spain. In (a) Each point represents a city, with mean exponent (β), on the x-axis, and mean speed $\overline{v}$ obtained in all trips made to calculate the exponent on the axis y. The histograms of the values of β and $\overline{v}$ are shown on the axes in the upper and right corners, respectively. The graph was segmented into four quadrants, in which the division is performed by the mean values of β and $\overline{v}$. The quadrants were colored and annotated according to the division criteria. The red dot represents the location of Madrid, Spain. In (b) taking all the nodes of Madrid, Spain as origin, the dots are colored as a function of their exponent value and their color is quantified by the color bar in the center. The longest segments without a deceleration point (SWDP) are plotted in black. The probability density function of the $\beta' s$ for each experiment is shown on the left of the color scale Figure (c) shows the mean correlation curve between time τ and the distance $\langle D \rangle$. The black traced line represents the exponent equal to 1.0. Figure (d) shows the distribution of SWDP sizes in number of nodes per frequency of occurrence.

Manchester, UK

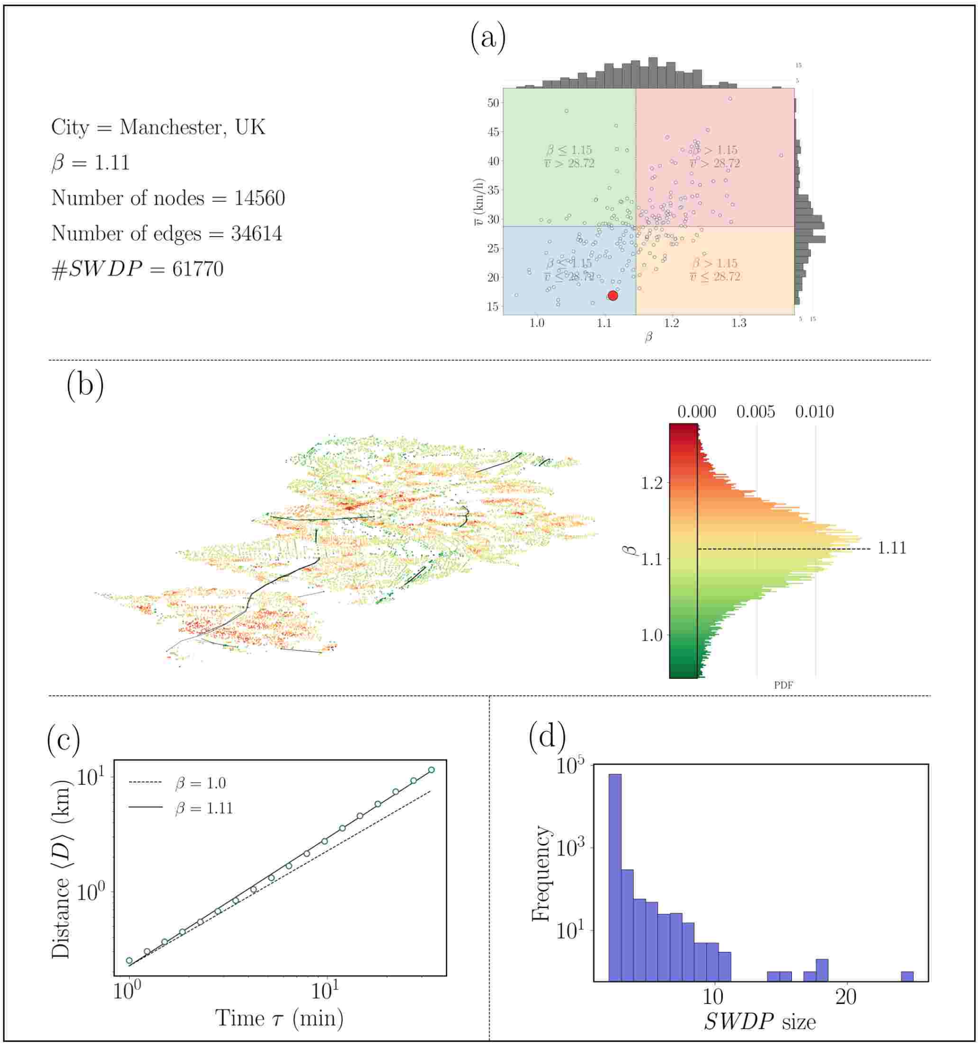

Fig. S97. Methodological sheet - Manchester, UK. In (a) Each point represents a city, with mean exponent (β), on the x-axis, and mean speed $\overline{v}$ obtained in all trips made to calculate the exponent on the axis y. The histograms of the values of β and $\overline{v}$ are shown on the axes in the upper and right corners, respectively. The graph was segmented into four quadrants, in which the division is performed by the mean values of β and $\overline{v}$. The quadrants were colored and annotated according to the division criteria. The red dot represents the location of Manchester, UK. In (b) taking all the nodes of Manchester, UK as origin, the dots are colored as a function of their exponent value and their color is quantified by the color bar in the center. The longest segments without a deceleration point (SWDP) are plotted in black. The probability density function of the $\beta's$ for each experiment is shown on the left of the color scale Figure (c) shows the mean correlation curve between time τ and the distance $\langle D \rangle$. The black traced line represents the exponent equal to 1.0. Figure (d) shows the distribution of SWDP sizes in number of nodes per frequency of occurrence.

Manila, Philippines

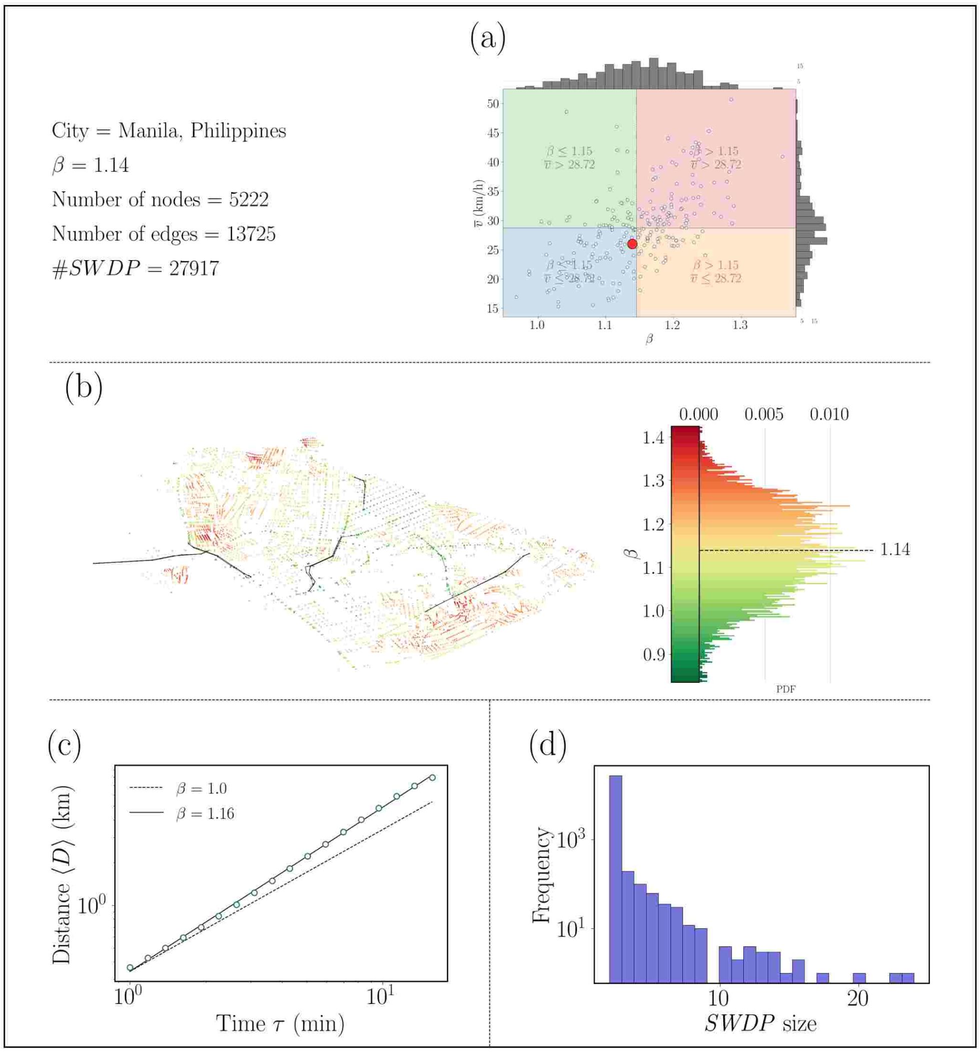

Fig. S98. Methodological sheet - Manila, Philippines. In (a) Each point represents a city, with mean exponent (β), on the x-axis, and mean speed $\overline{v}$ obtained in all trips made to calculate the exponent on the axis y. The histograms of the values of β and $\overline{v}$ are shown on the axes in the upper and right corners, respectively. The graph was segmented into four quadrants, in which the division is performed by the mean values of β and $\overline{v}$. The quadrants were colored and annotated according to the division criteria. The red dot represents the location of Manila, Philippines. In (b) taking all the nodes of Manila, Philippines as origin, the dots are colored as a function of their exponent value and their color is quantified by the color bar in the center. The longest segments without a deceleration point (SWDP) are plotted in black. The probability density function of the $\beta' s$ for each experiment is shown on the left of the color scale Figure (c) shows the mean correlation curve between time τ and the distance $\langle D \rangle$. The black traced line represents the exponent equal to 1.0. Figure (d) shows the distribution of SWDP sizes in number of nodes per frequency of occurrence.

Maputo, Mozambique

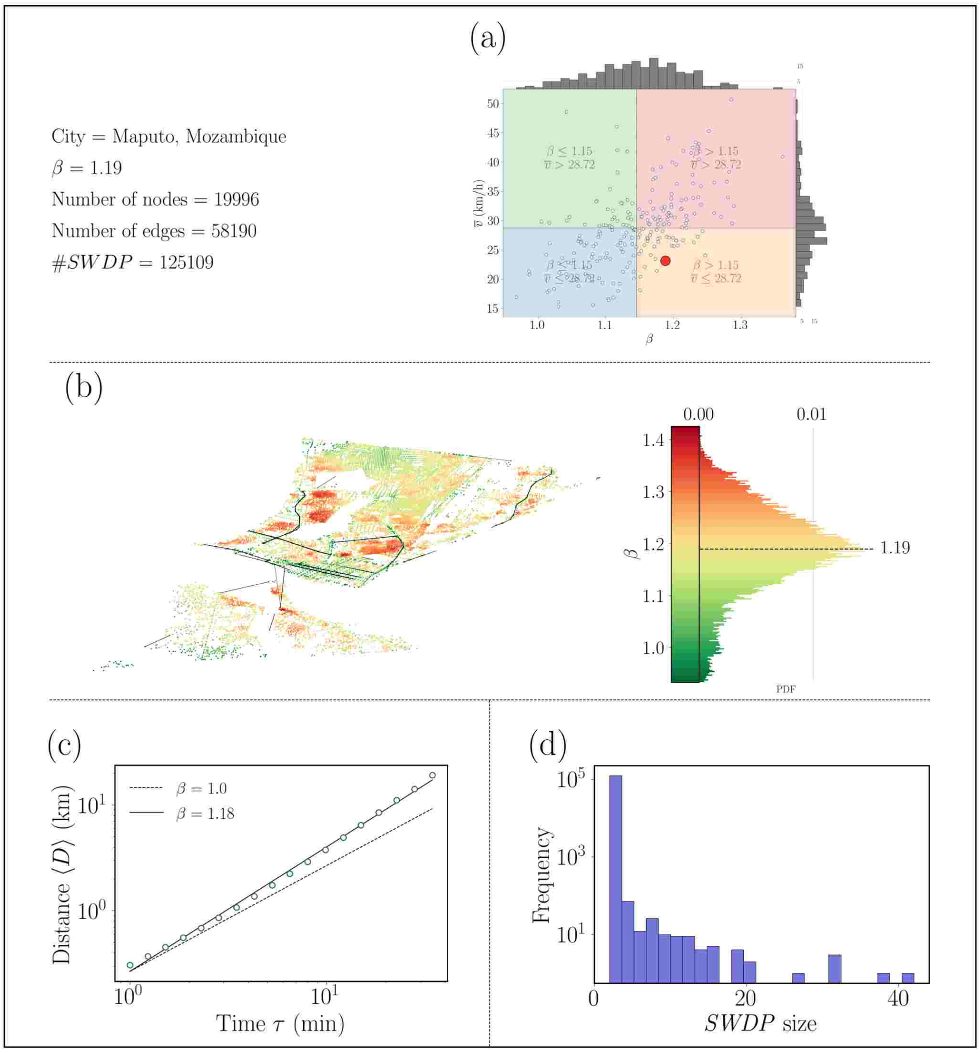

Fig. S99. Methodological sheet - Maputo, Mozambique. In (a) Each point represents a city, with mean exponent (β), on the x-axis, and mean speed $\overline{v}$ obtained in all trips made to calculate the exponent on the axis y. The histograms of the values of β and $\overline{v}$ are shown on the axes in the upper and right corners, respectively. The graph was segmented into four quadrants, in which the division is performed by the mean values of β and $\overline{v}$. The quadrants were colored and annotated according to the division criteria. The red dot represents the location of Maputo, Mozambique. In (b) taking all the nodes of Maputo, Mozambique as origin, the dots are colored as a function of their exponent value and their color is quantified by the color bar in the center. The longest segments without a deceleration point (SWDP) are plotted in black. The probability density function of the $\beta's$ for each experiment is shown on the left of the color scale Figure (c) shows the mean correlation curve between time τ and the distance $\langle D \rangle$. The black traced line represents the exponent equal to 1.0. Figure (d) shows the distribution of SWDP sizes in number of nodes per frequency of occurrence.

Mar del Plata, Argentina

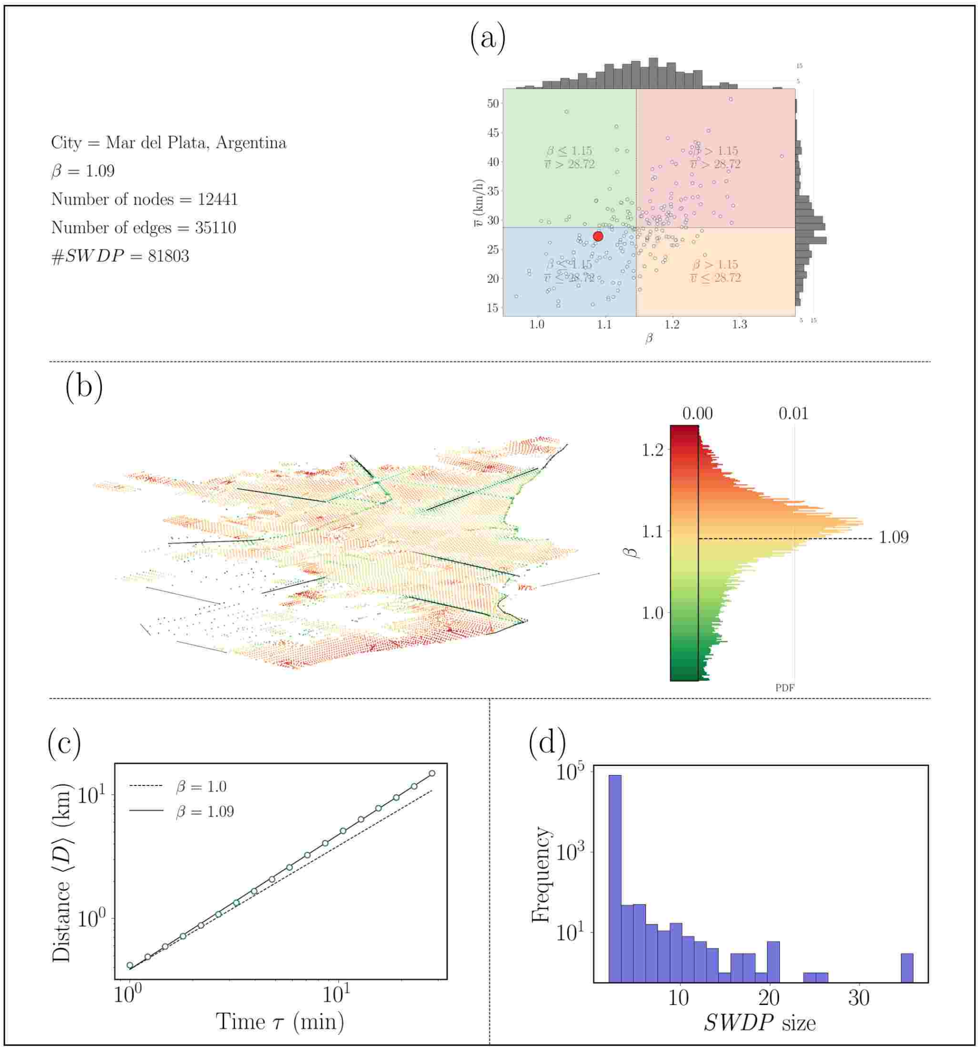

Fig. S100. Methodological sheet - Mar del Plata, Argentina. In (a) Each point represents a city, with mean exponent (β), on the x-axis, and mean speed $\overline{v}$ obtained in all trips made to calculate the exponent on the axis y. The histograms of the values of β and $\overline{v}$ are shown on the axes in the upper and right corners, respectively. The graph was segmented into four quadrants, in which the division is performed by the mean values of β and $\overline{v}$. The quadrants were colored and annotated according to the division criteria. The red dot represents the location of Mar del Plata, Argentina. In (b) taking all the nodes of Mar del Plata, Argentina as origin, the dots are colored as a function of their exponent value and their color is quantified by the color bar in the center. The longest segments without a deceleration point (SWDP) are plotted in black. The probability density function of the $\beta's$ for each experiment is shown on the left of the color scale Figure (c) shows the mean correlation curve between time τ and the distance $\langle D \rangle$. The black traced line represents the exponent equal to 1.0. Figure (d) shows the distribution of SWDP sizes in number of nodes per frequency of occurrence.

Marrakesh, Morocco

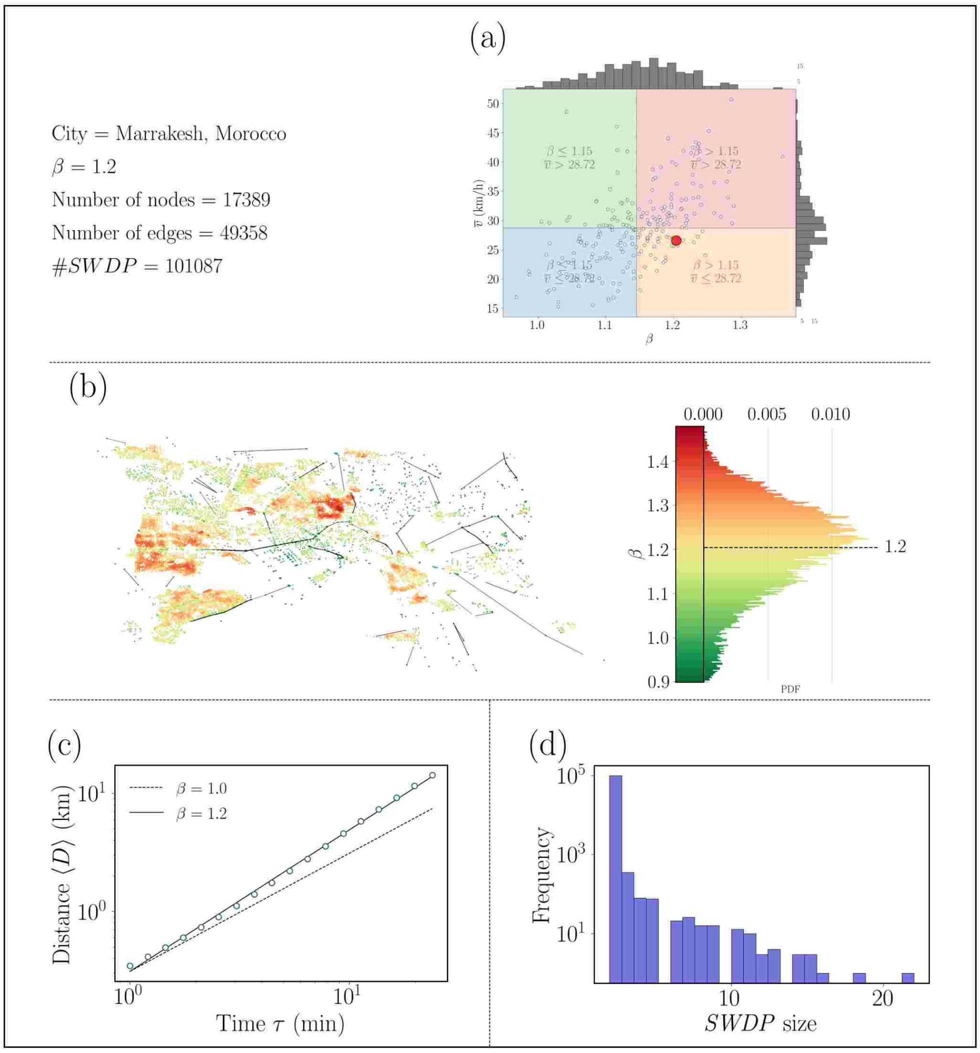

Fig. S101. Methodological sheet - Marrakesh, Morocco. In (a) Each point represents a city, with mean exponent (β), on the x-axis, and mean speed $\overline{v}$ obtained in all trips made to calculate the exponent on the axis y. The histograms of the values of β and $\overline{v}$ are shown on the axes in the upper and right corners, respectively. The graph was segmented into four quadrants, in which the division is performed by the mean values of β and $\overline{v}$. The quadrants were colored and annotated according to the division criteria. The red dot represents the location of Marrakesh, Morocco. In (b) taking all the nodes of Marrakesh, Morocco as origin, the dots are colored as a function of their exponent value and their color is quantified by the color bar in the center. The longest segments without a deceleration point (SWDP) are plotted in black. The probability density function of the $\beta's$ for each experiment is shown on the left of the color scale Figure (c) shows the mean correlation curve between time τ and the distance $\langle D \rangle$. The black traced line represents the exponent equal to 1.0. Figure (d) shows the distribution of SWDP sizes in number of nodes per frequency of occurrence.

Marrakesh. Morocco

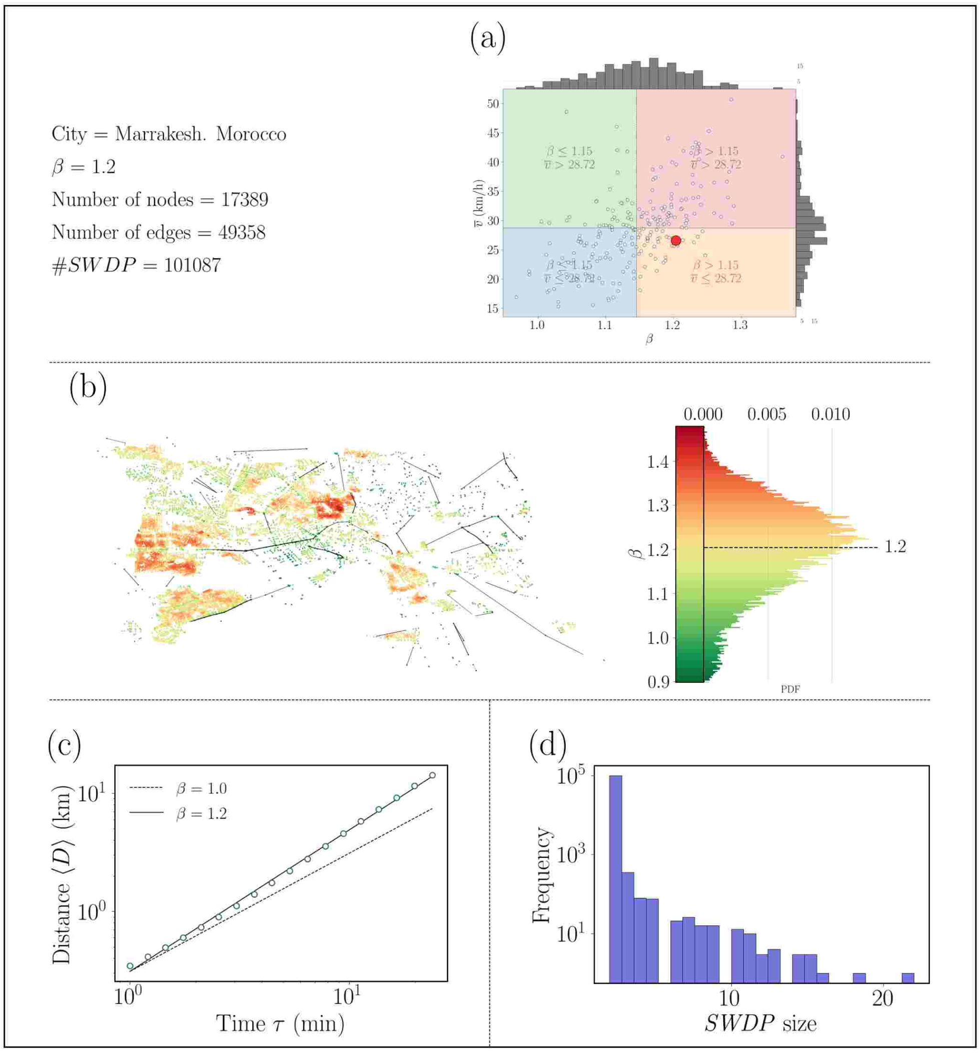

Fig. S102. Methodological sheet - Marrakesh. Morocco. In (a) Each point represents a city, with mean exponent (β), on the x-axis, and mean speed $\overline{v}$ obtained in all trips made to calculate the exponent on the axis y. The histograms of the values of β and $\overline{v}$ are shown on the axes in the upper and right corners, respectively. The graph was segmented into four quadrants, in which the division is performed by the mean values of β and $\overline{v}$. The quadrants were colored and annotated according to the division criteria. The red dot represents the location of Marrakesh. Morocco. In (b) taking all the nodes of Marrakesh. Morocco as origin, the dots are colored as a function of their exponent value and their color is quantified by the color bar in the center. The longest segments without a deceleration point (SWDP) are plotted in black. The probability density function of the $\beta's$ for each experiment is shown on the left of the color scale Figure (c) shows the mean correlation curve between time τ and the distance $\langle D \rangle$. The black traced line represents the exponent equal to 1.0. Figure (d) shows the distribution of SWDP sizes in number of nodes per frequency of occurrence.

Marseille, France

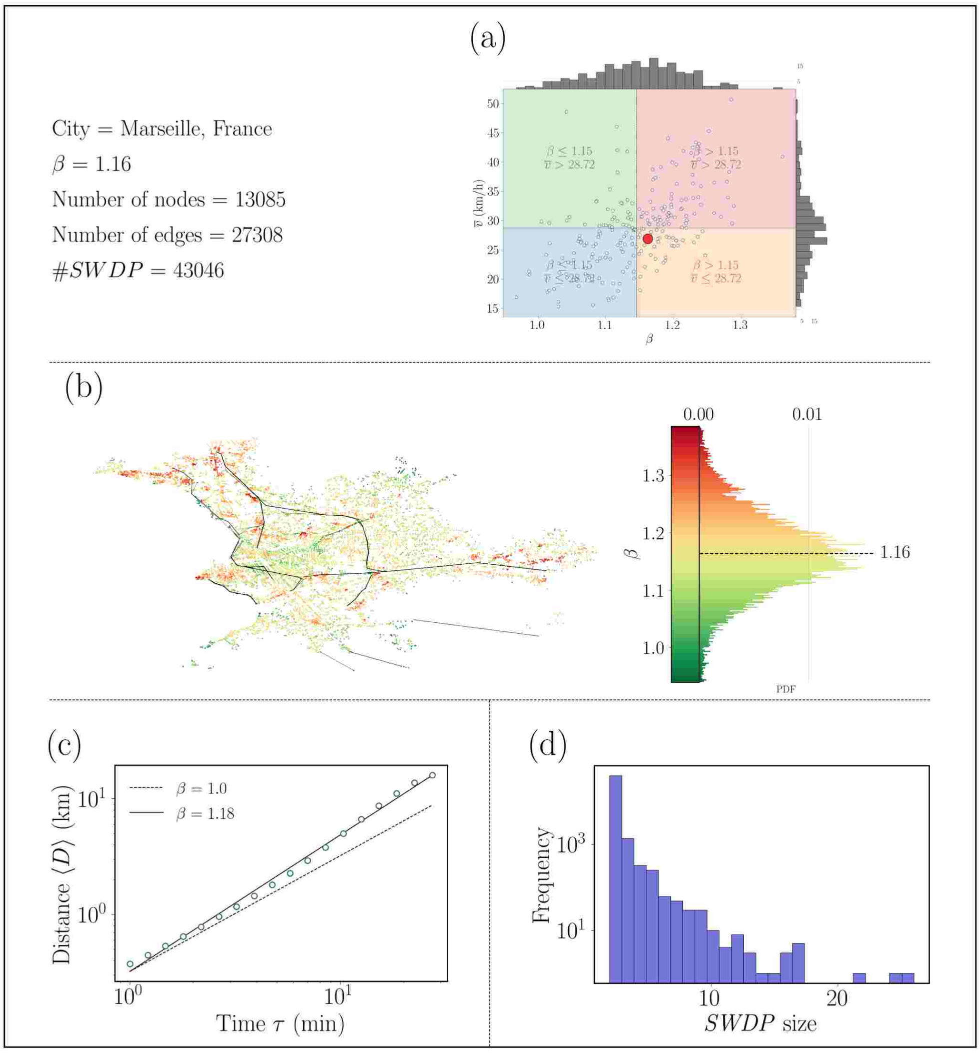

Fig. S103. Methodological sheet - Marseille, France. In (a) Each point represents a city, with mean exponent (β), on the x-axis, and mean speed $\overline{v}$ obtained in all trips made to calculate the exponent on the axis y. The histograms of the values of β and $\overline{v}$ are shown on the axes in the upper and right corners, respectively. The graph was segmented into four quadrants, in which the division is performed by the mean values of β and $\overline{v}$. The quadrants were colored and annotated according to the division criteria. The red dot represents the location of Marseille, France. In (b) taking all the nodes of Marseille, France as origin, the dots are colored as a function of their exponent value and their color is quantified by the color bar in the center. The longest segments without a deceleration point (SWDP) are plotted in black. The probability density function of the $\beta's$ for each experiment is shown on the left of the color scale Figure (c) shows the mean correlation curve between time τ and the distance $\langle D \rangle$. The black traced line represents the exponent equal to 1.0. Figure (d) shows the distribution of SWDP sizes in number of nodes per frequency of occurrence.

Medellín, Colombia

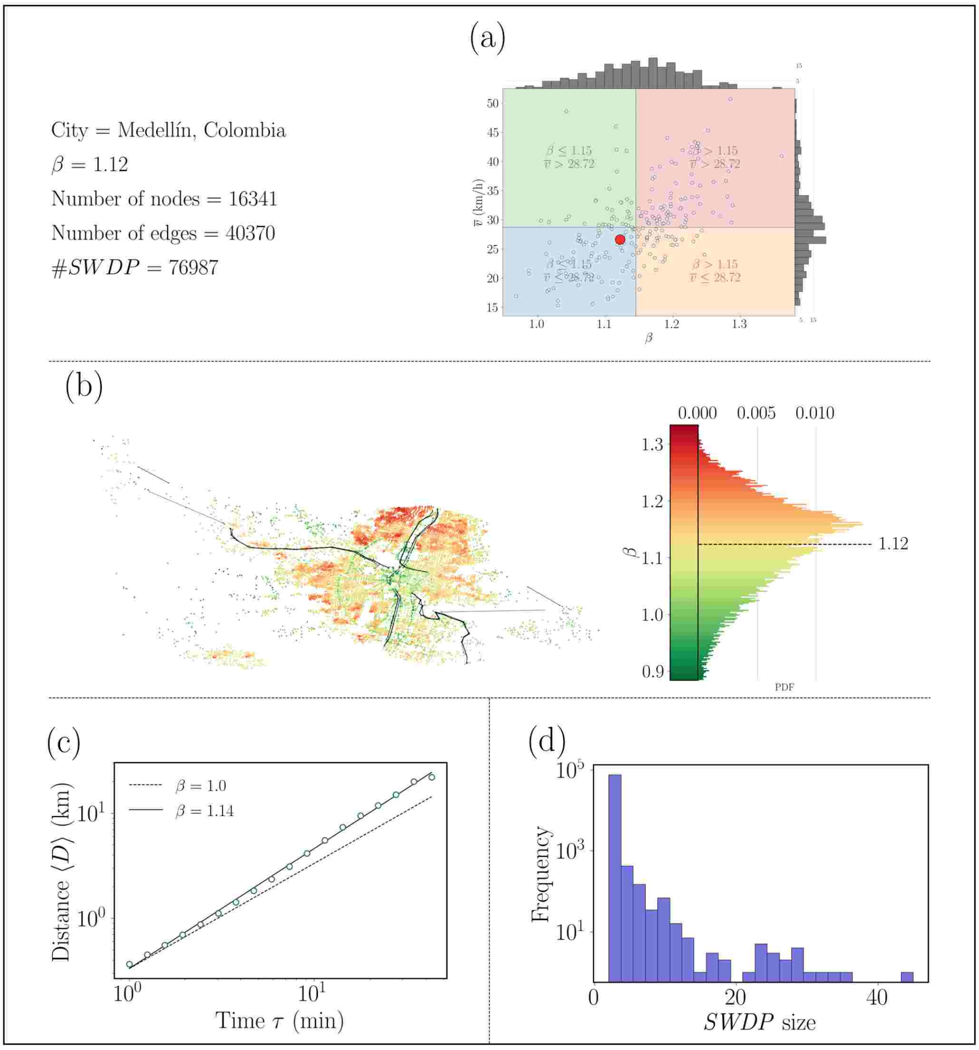

Fig. S104. Methodological sheet - Medellín, Colombia. In (a) Each point represents a city, with mean exponent (β), on the x-axis, and mean speed $\overline{v}$ obtained in all trips made to calculate the exponent on the axis y. The histograms of the values of β and $\overline{v}$ are shown on the axes in the upper and right corners, respectively. The graph was segmented into four quadrants, in which the division is performed by the mean values of β and $\overline{v}$. The quadrants were colored and annotated according to the division criteria. The red dot represents the location of Medellín, Colombia. In (b) taking all the nodes of Medellín, Colombia as origin, the dots are colored as a function of their exponent value and their color is quantified by the color bar in the center. The longest segments without a deceleration point (SWDP) are plotted in black. The probability density function of the $\beta's$ for each experiment is shown on the left of the color scale Figure (c) shows the mean correlation curve between time τ and the distance $\langle D \rangle$. The black traced line represents the exponent equal to 1.0. Figure (d) shows the distribution of SWDP sizes in number of nodes per frequency of occurrence.

Memphis, USA

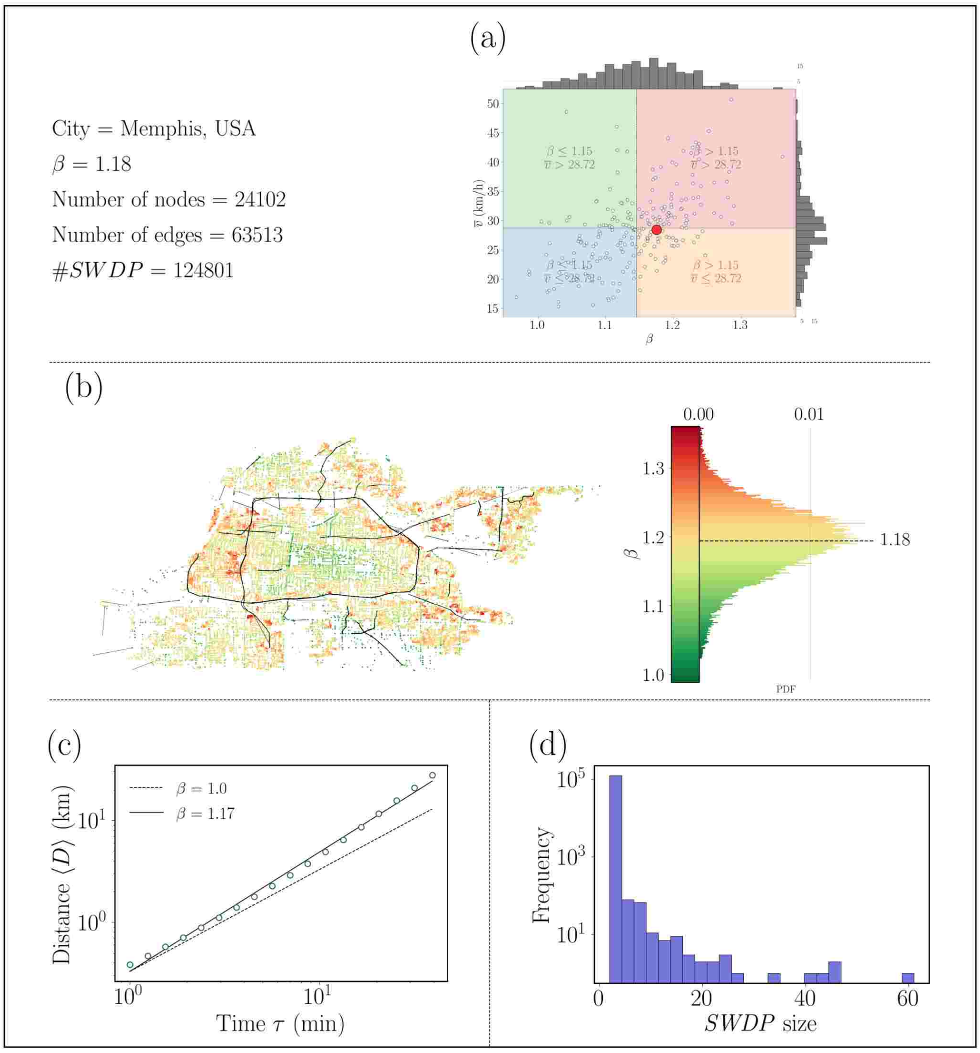

Fig. S105. Methodological sheet - Memphis, USA. In (a) Each point represents a city, with mean exponent (β), on the x-axis, and mean speed $\overline{v}$ obtained in all trips made to calculate the exponent on the axis y. The histograms of the values of β and $\overline{v}$ are shown on the axes in the upper and right corners, respectively. The graph was segmented into four quadrants, in which the division is performed by the mean values of β and $\overline{v}$. The quadrants were colored and annotated according to the division criteria. The red dot represents the location of Memphis, USA. In (b) taking all the nodes of Memphis, USA as origin, the dots are colored as a function of their exponent value and their color is quantified by the color bar in the center. The longest segments without a deceleration point (SWDP) are plotted in black. The probability density function of the $\beta's$ for each experiment is shown on the left of the color scale Figure (c) shows the mean correlation curve between time τ and the distance $\langle D \rangle$. The black traced line represents the exponent equal to 1.0. Figure (d) shows the distribution of SWDP sizes in number of nodes per frequency of occurrence.

Mexico City, Mexico

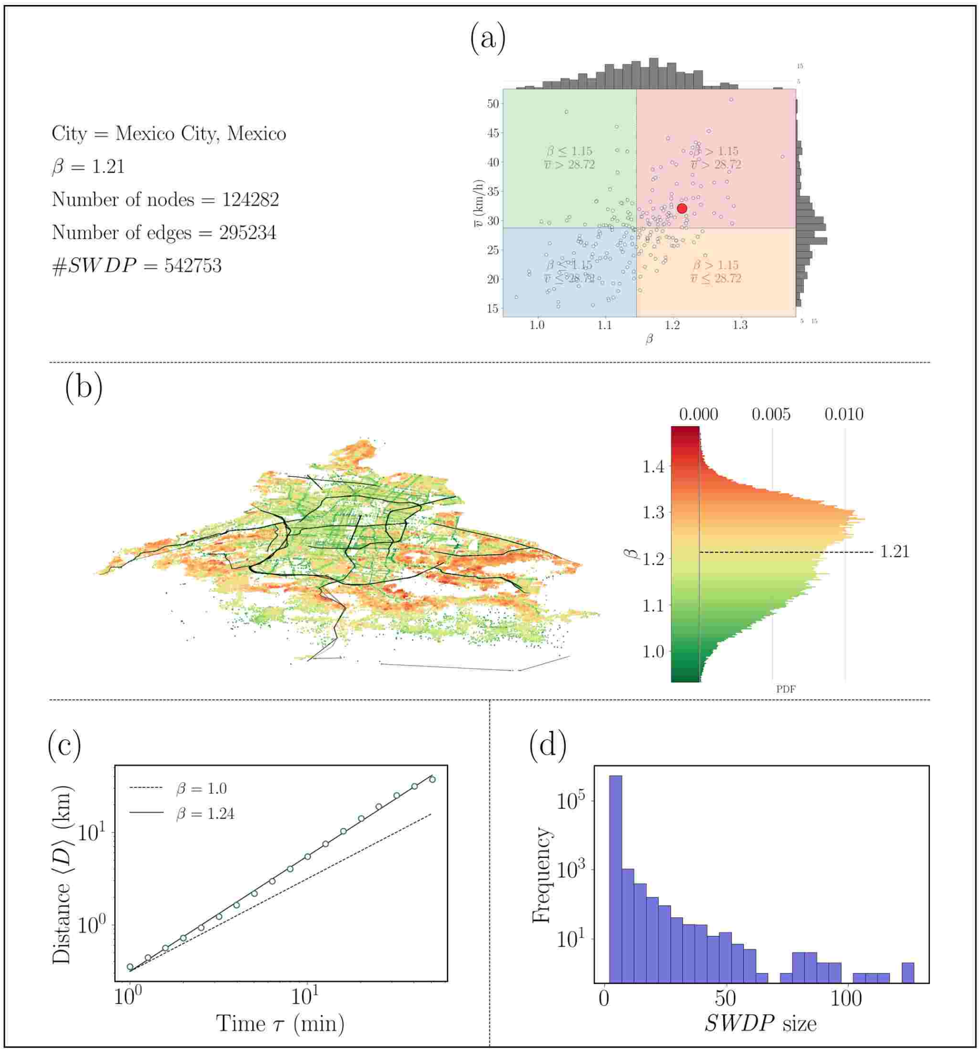

Fig. S106. Methodological sheet - Mexico City, Mexico. In (a) Each point represents a city, with mean exponent (β), on the x-axis, and mean speed $\overline{v}$ obtained in all trips made to calculate the exponent on the axis y. The histograms of the values of β and $\overline{v}$ are shown on the axes in the upper and right corners, respectively. The graph was segmented into four quadrants, in which the division is performed by the mean values of β and $\overline{v}$. The quadrants were colored and annotated according to the division criteria. The red dot represents the location of Mexico City, Mexico. In (b) taking all the nodes of Mexico City, Mexico as origin, the dots are colored as a function of their exponent value and their color is quantified by the color bar in the center. The longest segments without a deceleration point (SWDP) are plotted in black. The probability density function of the $\beta's$ for each experiment is shown on the left of the color scale Figure (c) shows the mean correlation curve between time τ and the distance $\langle D \rangle$. The black traced line represents the exponent equal to 1.0. Figure (d) shows the distribution of SWDP sizes in number of nodes per frequency of occurrence.

Miami, USA

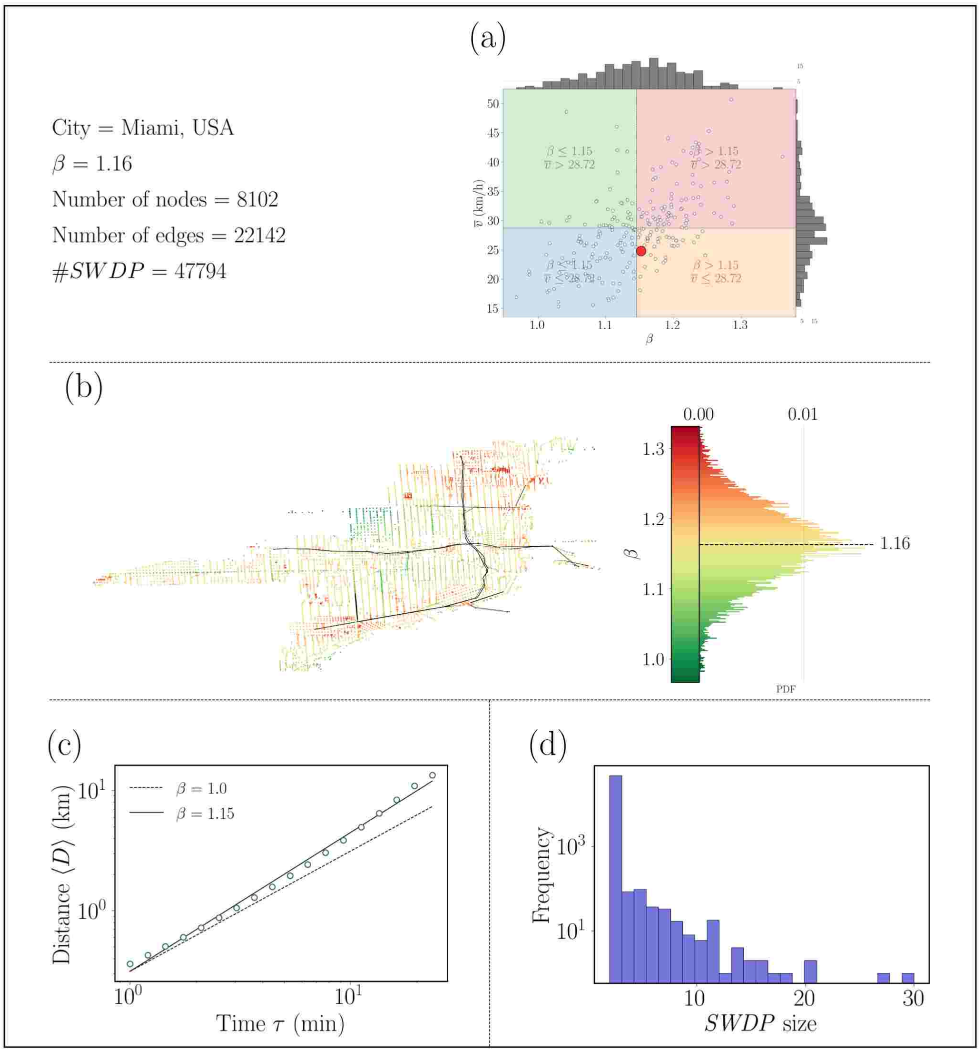

Fig. S107. Methodological sheet - Miami, USA. In (a) Each point represents a city, with mean exponent (β), on the x-axis, and mean speed $\overline{v}$ obtained in all trips made to calculate the exponent on the axis y. The histograms of the values of β and $\overline{v}$ are shown on the axes in the upper and right corners, respectively. The graph was segmented into four quadrants, in which the division is performed by the mean values of β and $\overline{v}$. The quadrants were colored and annotated according to the division criteria. The red dot represents the location of Miami, USA. In (b) taking all the nodes of Miami, USA as origin, the dots are colored as a function of their exponent value and their color is quantified by the color bar in the center. The longest segments without a deceleration point (SWDP) are plotted in black. The probability density function of the $\beta's$ for each experiment is shown on the left of the color scale Figure (c) shows the mean correlation curve between time τ and the distance $\langle D \rangle$. The black traced line represents the exponent equal to 1.0. Figure (d) shows the distribution of SWDP sizes in number of nodes per frequency of occurrence.

Milan, Italy

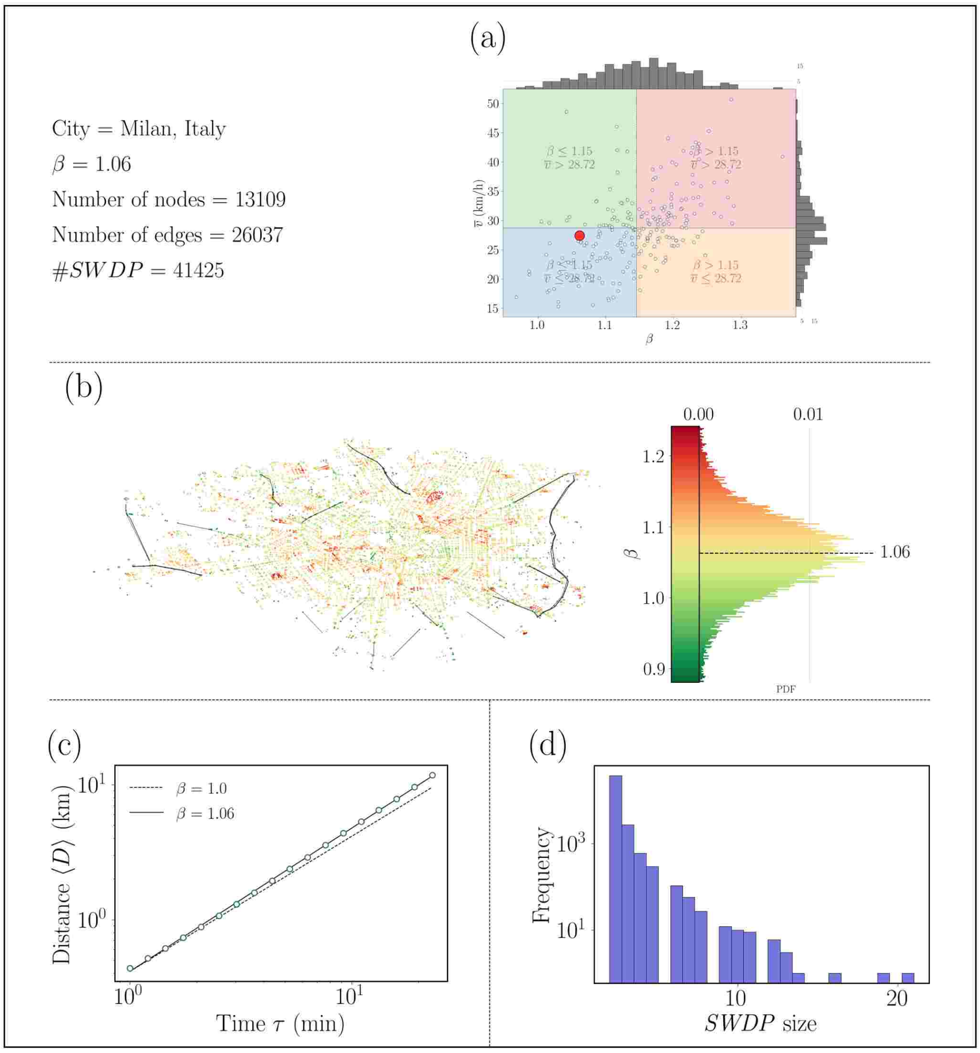

Fig. S108. Methodological sheet - Milan, Italy. In (a) Each point represents a city, with mean exponent (β), on the x-axis, and mean speed $\overline{v}$ obtained in all trips made to calculate the exponent on the axis y. The histograms of the values of β and $\overline{v}$ are shown on the axes in the upper and right corners, respectively. The graph was segmented into four quadrants, in which the division is performed by the mean values of β and $\overline{v}$. The quadrants were colored and annotated according to the division criteria. The red dot represents the location of Milan, Italy. In (b) taking all the nodes of Milan, Italy as origin, the dots are colored as a function of their exponent value and their color is quantified by the color bar in the center. The longest segments without a deceleration point (SWDP) are plotted in black. The probability density function of the $\beta's$ for each experiment is shown on the left of the color scale Figure (c) shows the mean correlation curve between time τ and the distance $\langle D \rangle$. The black traced line represents the exponent equal to 1.0. Figure (d) shows the distribution of SWDP sizes in number of nodes per frequency of occurrence.

Milwaukee, USA

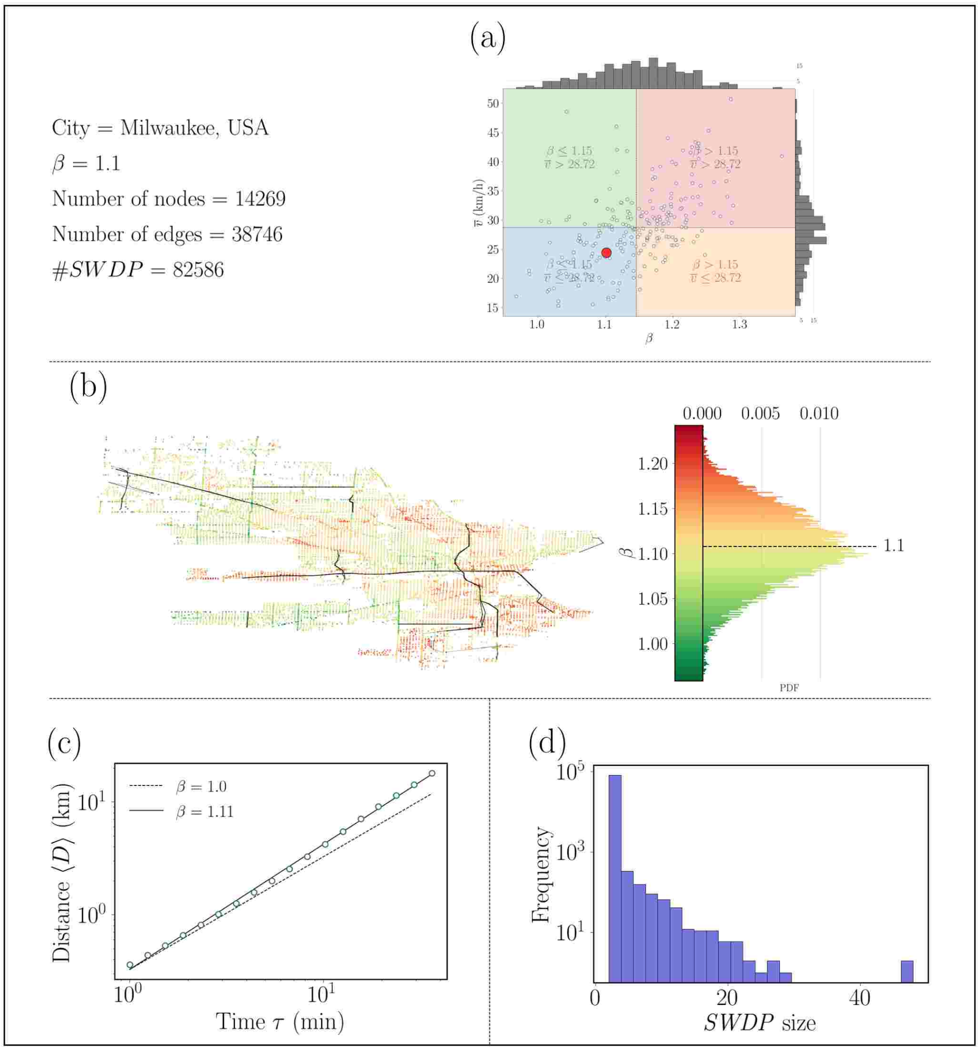

Fig. S109. Methodological sheet - Milwaukee, USA. In (a) Each point represents a city, with mean exponent (β), on the x-axis, and mean speed $\overline{v}$ obtained in all trips made to calculate the exponent on the axis y. The histograms of the values of β and $\overline{v}$ are shown on the axes in the upper and right corners, respectively. The graph was segmented into four quadrants, in which the division is performed by the mean values of β and $\overline{v}$. The quadrants were colored and annotated according to the division criteria. The red dot represents the location of Milwaukee, USA. In (b) taking all the nodes of Milwaukee, USA as origin, the dots are colored as a function of their exponent value and their color is quantified by the color bar in the center. The longest segments without a deceleration point (SWDP) are plotted in black. The probability density function of the $\beta's$ for each experiment is shown on the left of the color scale Figure (c) shows the mean correlation curve between time τ and the distance $\langle D \rangle$. The black traced line represents the exponent equal to 1.0. Figure (d) shows the distribution of SWDP sizes in number of nodes per frequency of occurrence.

Minneapolis, USA

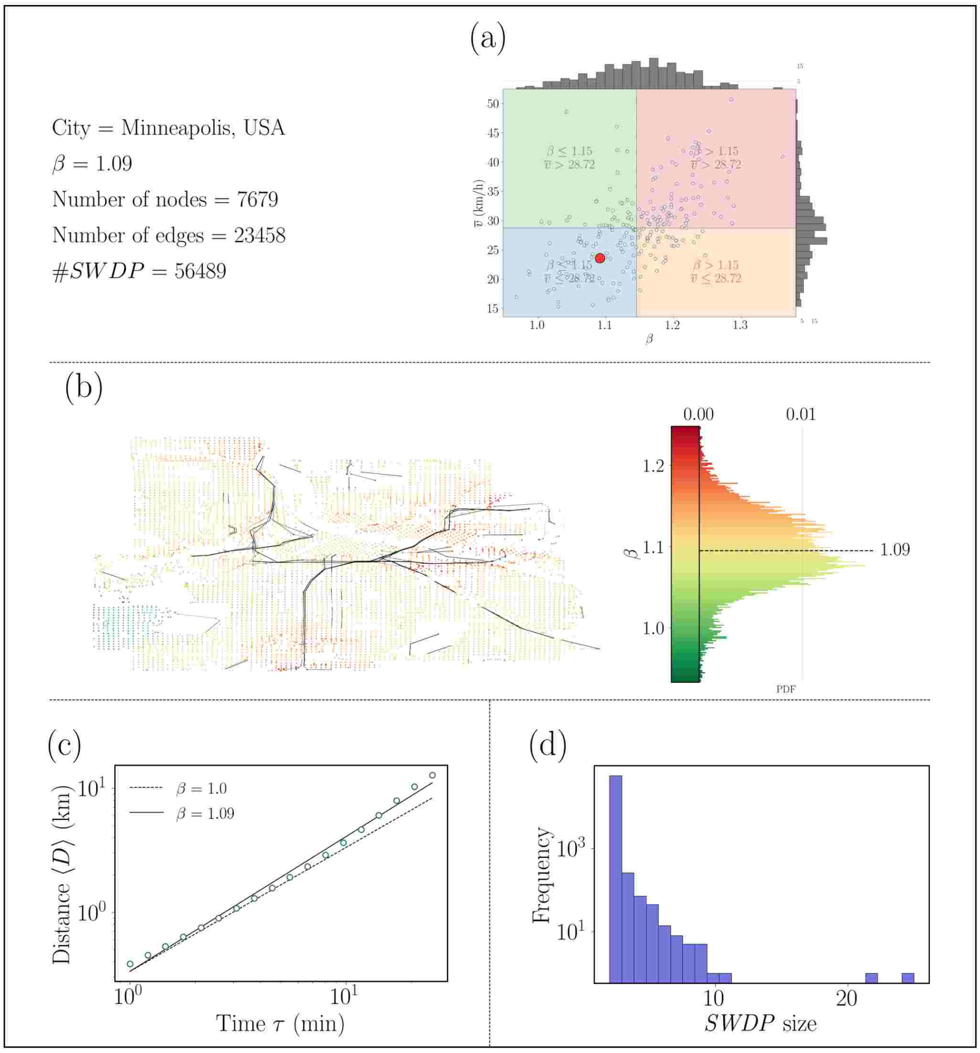

Fig. S110. Methodological sheet - Minneapolis, USA. In (a) Each point represents a city, with mean exponent (β), on the x-axis, and mean speed $\overline{v}$ obtained in all trips made to calculate the exponent on the axis y. The histograms of the values of β and $\overline{v}$ are shown on the axes in the upper and right corners, respectively. The graph was segmented into four quadrants, in which the division is performed by the mean values of β and $\overline{v}$. The quadrants were colored and annotated according to the division criteria. The red dot represents the location of Minneapolis, USA. In (b) taking all the nodes of Minneapolis, USA as origin, the dots are colored as a function of their exponent value and their color is quantified by the color bar in the center. The longest segments without a deceleration point (SWDP) are plotted in black. The probability density function of the $\beta's$ for each experiment is shown on the left of the color scale Figure (c) shows the mean correlation curve between time τ and the distance $\langle D \rangle$. The black traced line represents the exponent equal to 1.0. Figure (d) shows the distribution of SWDP sizes in number of nodes per frequency of occurrence.

Mombasa, Kenya

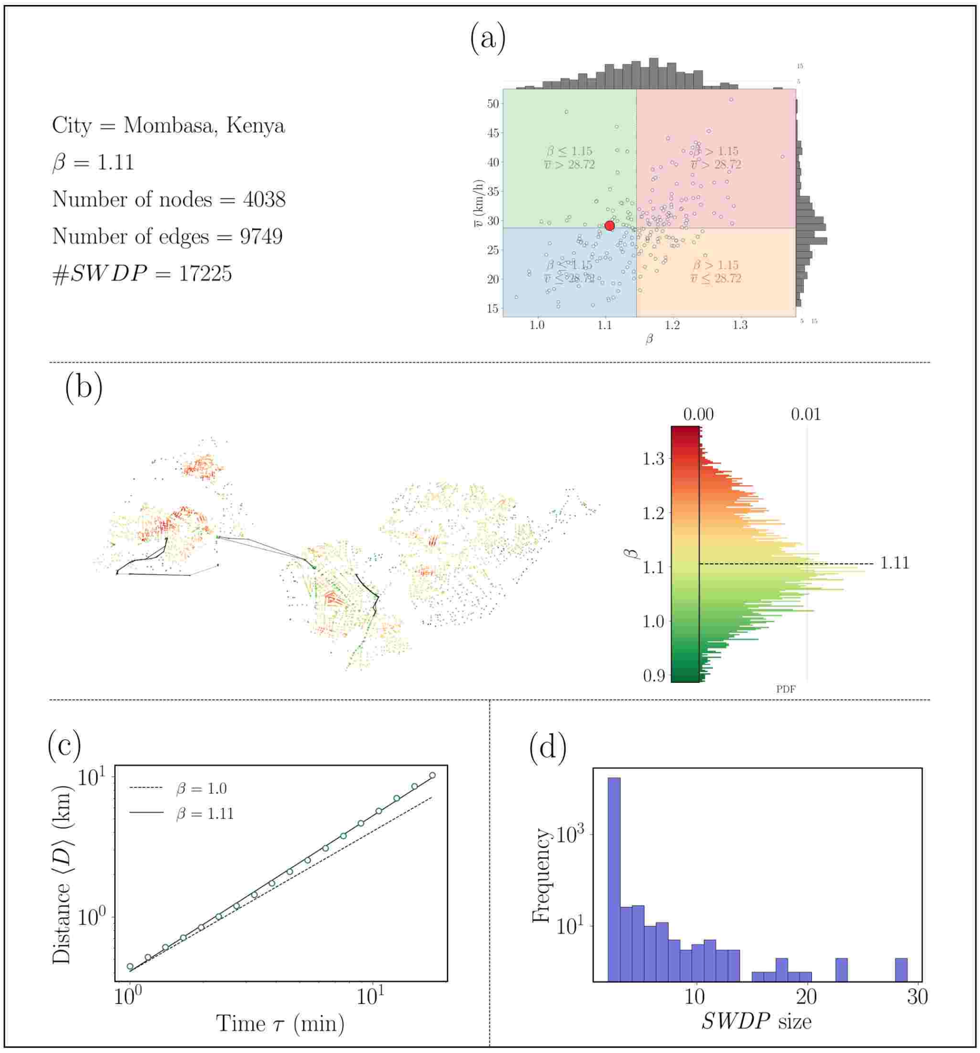

Fig. S111. Methodological sheet - Mombasa, Kenya. In (a) Each point represents a city, with mean exponent (β), on the x-axis, and mean speed $\overline{v}$ obtained in all trips made to calculate the exponent on the axis y. The histograms of the values of β and $\overline{v}$ are shown on the axes in the upper and right corners, respectively. The graph was segmented into four quadrants, in which the division is performed by the mean values of β and $\overline{v}$. The quadrants were colored and annotated according to the division criteria. The red dot represents the location of Mombasa, Kenya. In (b) taking all the nodes of Mombasa, Kenya as origin, the dots are colored as a function of their exponent value and their color is quantified by the color bar in the center. The longest segments without a deceleration point (SWDP) are plotted in black. The probability density function of the $\beta's$ for each experiment is shown on the left of the color scale Figure (c) shows the mean correlation curve between time τ and the distance $\langle D \rangle$. The black traced line represents the exponent equal to 1.0. Figure (d) shows the distribution of SWDP sizes in number of nodes per frequency of occurrence.

Montevideo, Uruguay

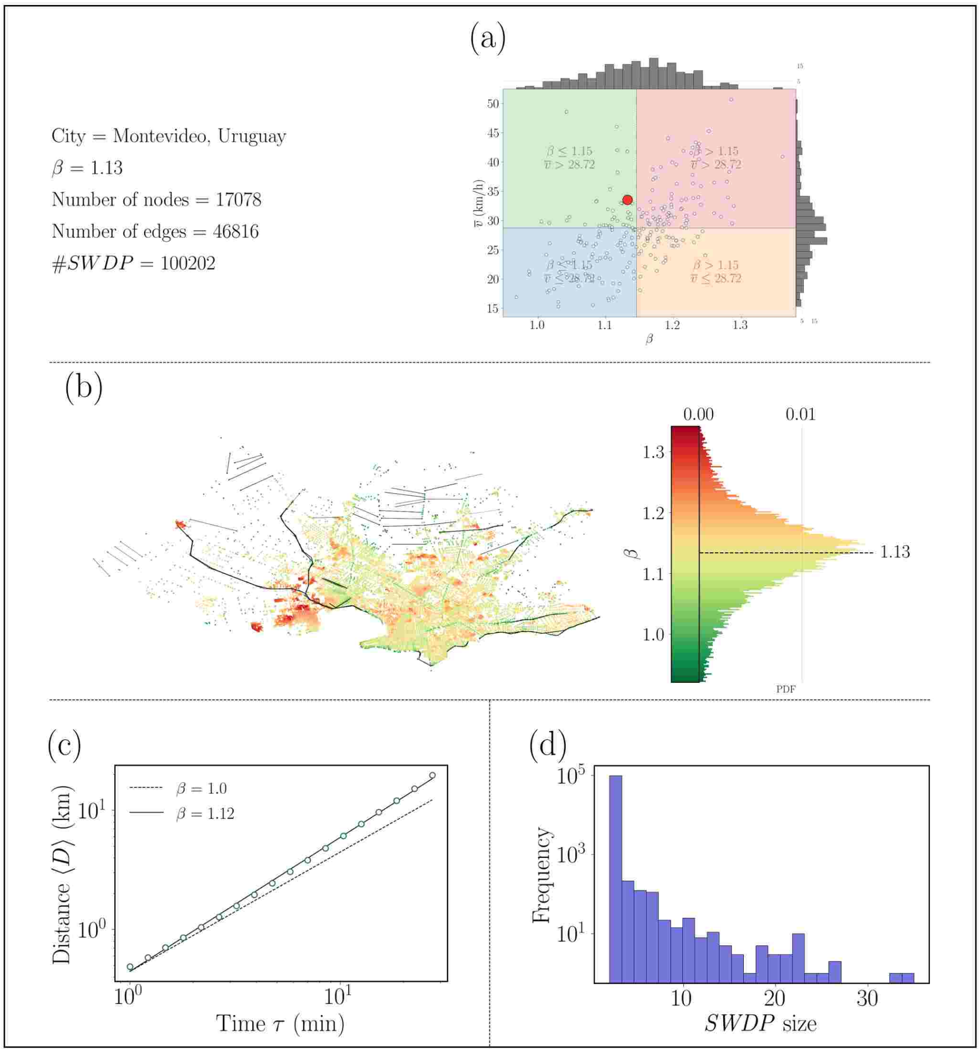

Fig. S112. Methodological sheet - Montevideo, Uruguay. In (a) Each point represents a city, with mean exponent (β), on the x-axis, and mean speed $\overline{v}$ obtained in all trips made to calculate the exponent on the axis y. The histograms of the values of β and $\overline{v}$ are shown on the axes in the upper and right corners, respectively. The graph was segmented into four quadrants, in which the division is performed by the mean values of β and $\overline{v}$. The quadrants were colored and annotated according to the division criteria. The red dot represents the location of Montevideo, Uruguay. In (b) taking all the nodes of Montevideo, Uruguay as origin, the dots are colored as a function of their exponent value and their color is quantified by the color bar in the center. The longest segments without a deceleration point (SWDP) are plotted in black. The probability density function of the $\beta's$ for each experiment is shown on the left of the color scale Figure (c) shows the mean correlation curve between time τ and the distance $\langle D \rangle$. The black traced line represents the exponent equal to 1.0. Figure (d) shows the distribution of SWDP sizes in number of nodes per frequency of occurrence.

Montreal, Canadá

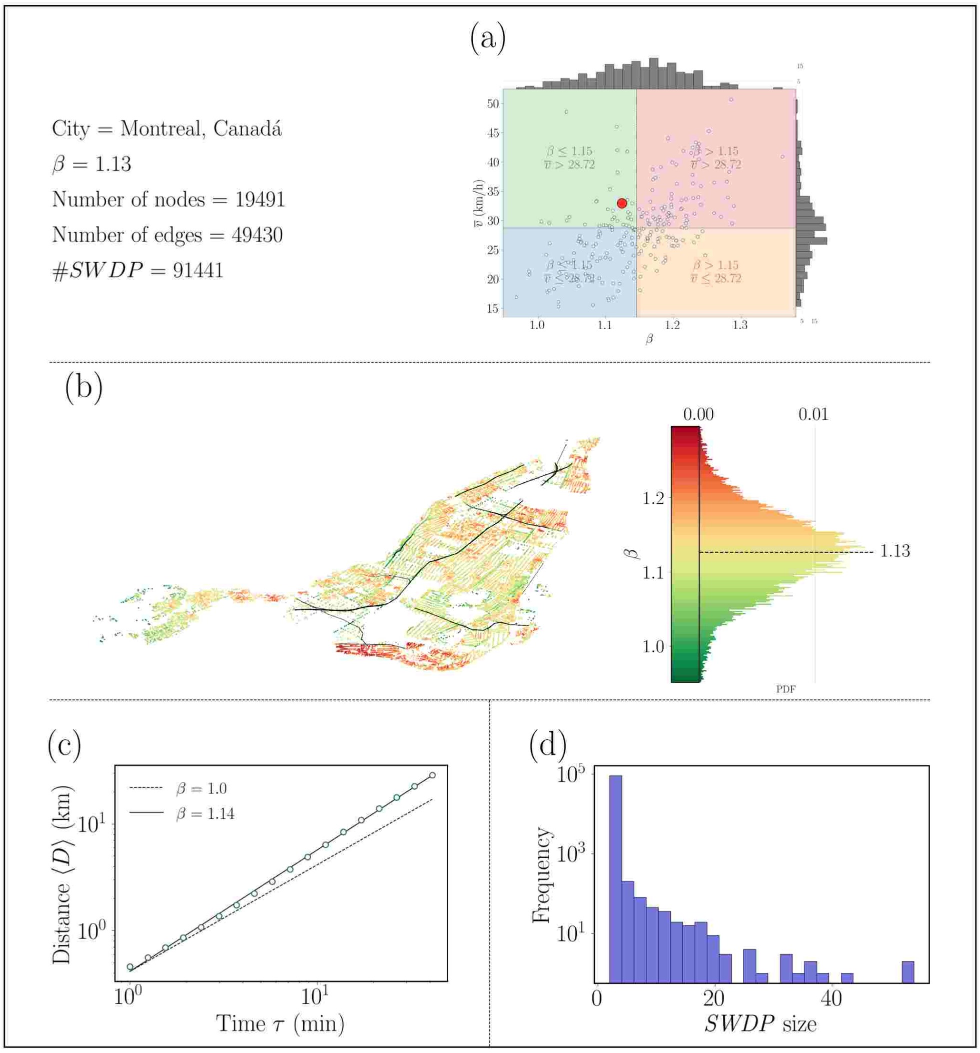

Fig. S113. Methodological sheet - Montreal, Canadá. In (a) Each point represents a city, with mean exponent (β), on the x-axis, and mean speed $\overline{v}$ obtained in all trips made to calculate the exponent on the axis y. The histograms of the values of β and $\overline{v}$ are shown on the axes in the upper and right corners, respectively. The graph was segmented into four quadrants, in which the division is performed by the mean values of β and $\overline{v}$. The quadrants were colored and annotated according to the division criteria. The red dot represents the location of Montreal, Canadá. In (b) taking all the nodes of Montreal, Canadá as origin, the dots are colored as a function of their exponent value and their color is quantified by the color bar in the center. The longest segments without a deceleration point (SWDP) are plotted in black. The probability density function of the $\beta's$ for each experiment is shown on the left of the color scale Figure (c) shows the mean correlation curve between time τ and the distance $\langle D \rangle$. The black traced line represents the exponent equal to 1.0. Figure (d) shows the distribution of SWDP sizes in number of nodes per frequency of occurrence.

Mumbai, India

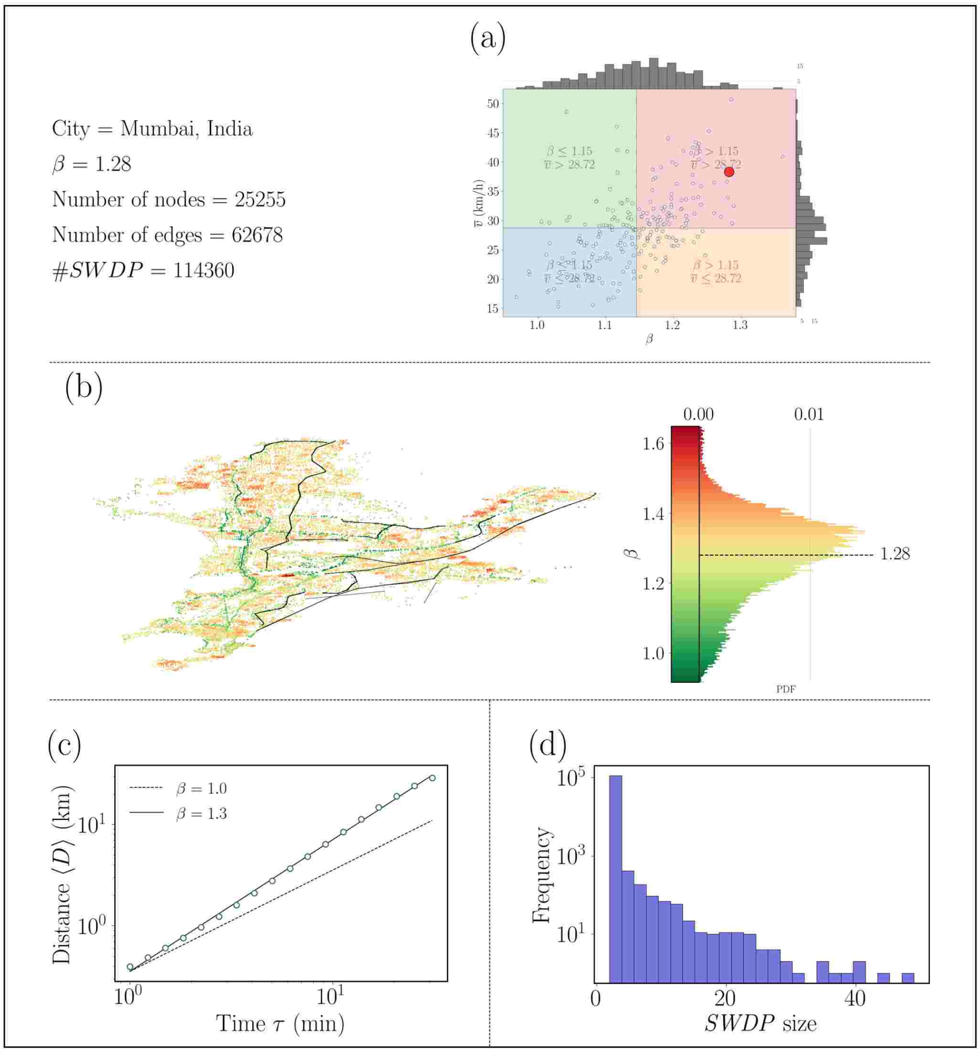

Fig. S114. Methodological sheet - Mumbai, India. In (a) Each point represents a city, with mean exponent (β), on the x-axis, and mean speed $\overline{v}$ obtained in all trips made to calculate the exponent on the axis y. The histograms of the values of β and $\overline{v}$ are shown on the axes in the upper and right corners, respectively. The graph was segmented into four quadrants, in which the division is performed by the mean values of β and $\overline{v}$. The quadrants were colored and annotated according to the division criteria. The red dot represents the location of Mumbai, India. In (b) taking all the nodes of Mumbai, India as origin, the dots are colored as a function of their exponent value and their color is quantified by the color bar in the center. The longest segments without a deceleration point (SWDP) are plotted in black. The probability density function of the $\beta's$ for each experiment is shown on the left of the color scale Figure (c) shows the mean correlation curve between time τ and the distance $\langle D \rangle$. The black traced line represents the exponent equal to 1.0. Figure (d) shows the distribution of SWDP sizes in number of nodes per frequency of occurrence.

Munich, Germany

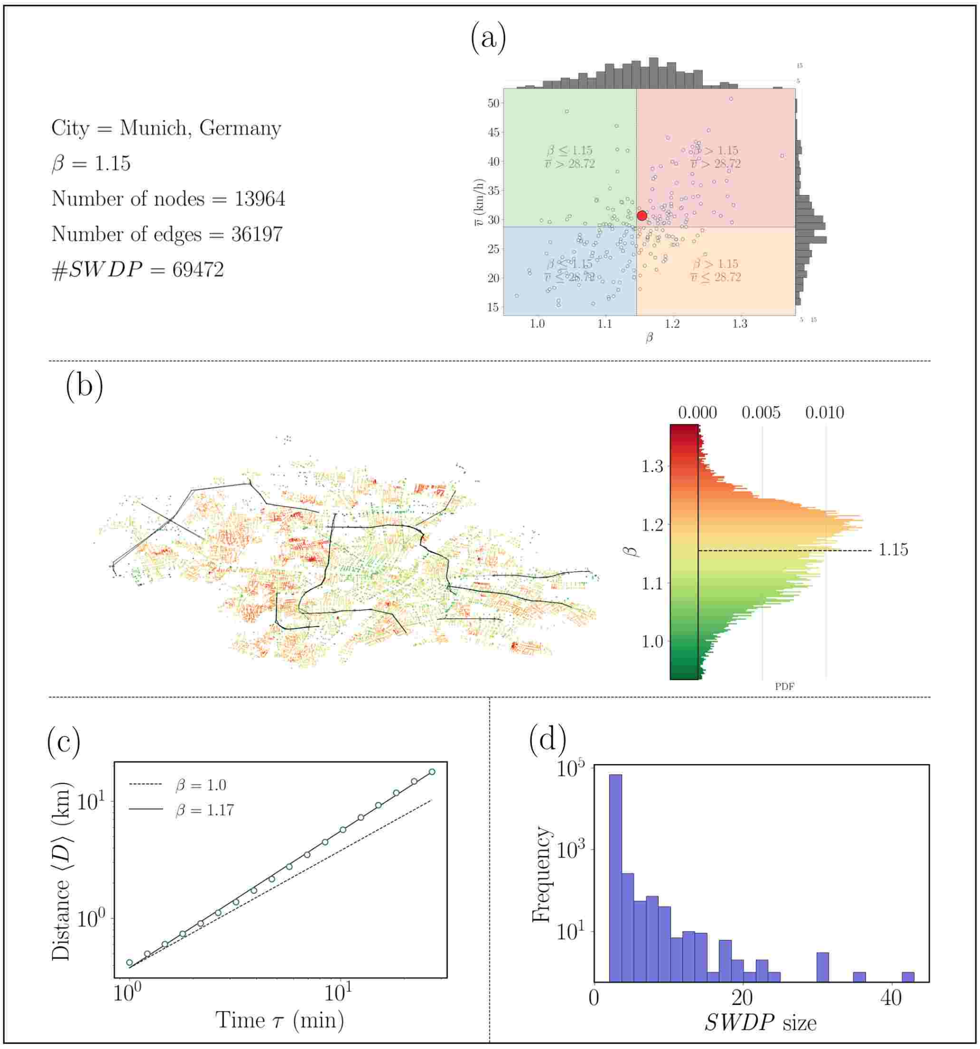

Fig. S115. Methodological sheet - Munich, Germany. In (a) Each point represents a city, with mean exponent (β), on the x-axis, and mean speed $\bar{v}$ obtained in all trips made to calculate the exponent on the axis y. The histograms of the values of β and $\bar{v}$ are shown on the axes in the upper and right corners, respectively. The graph was segmented into four quadrants, in which the division is performed by the mean values of β and $\bar{v}$. The quadrants were colored and annotated according to the division criteria. The red dot represents the location of Munich, Germany. In (b) taking all the nodes of Munich, Germany as origin, the dots are colored as a function of their exponent value and their color is quantified by the color bar in the center. The longest segments without a deceleration point (SWDP) are plotted in black. The probability density function of the $\beta's$ for each experiment is shown on the left of the color scale Figure (c) shows the mean correlation curve between time τ and the distance $\langle D \rangle$. The black traced line represents the exponent equal to 1.0. Figure (d) shows the distribution of SWDP sizes in number of nodes per frequency of occurrence.

Mérida, Yucatán, Mexico

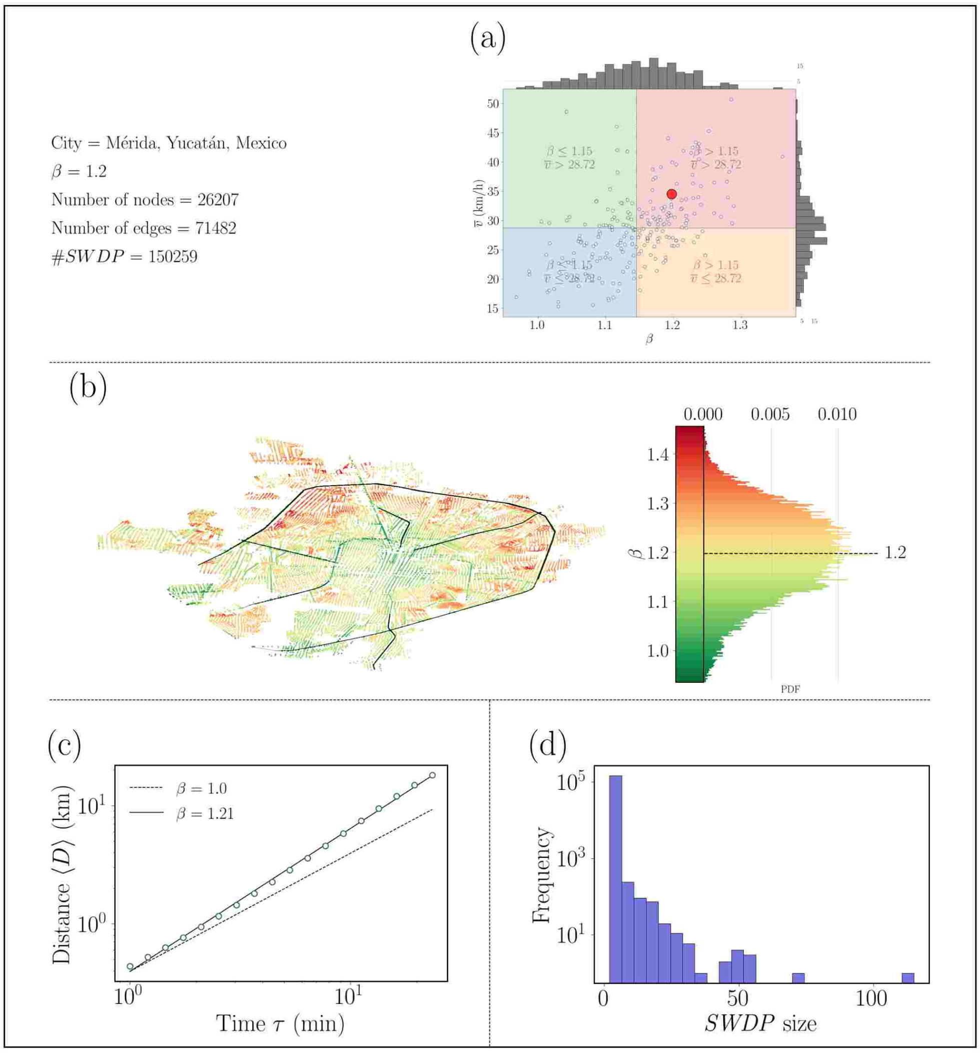

Fig. S116. Methodological sheet - Mérida, Yucatán, Mexico. In (a) Each point represents a city, with mean exponent (β), on the x-axis, and mean speed $\overline{v}$ obtained in all trips made to calculate the exponent on the axis y. The histograms of the values of β and $\overline{v}$ are shown on the axes in the upper and right corners, respectively. The graph was segmented into four quadrants, in which the division is performed by the mean values of β and $\overline{v}$. The quadrants were colored and annotated according to the division criteria. The red dot represents the location of Mérida, Yucatán, Mexico. In (b) taking all the nodes of Mérida, Yucatán, Mexico as origin, the dots are colored as a function of their exponent value and their color is quantified by the color bar in the center. The longest segments without a deceleration point (SWDP) are plotted in black. The probability density function of the $\beta's$ for each experiment is shown on the left of the color scale Figure (c) shows the mean correlation curve between time τ and the distance $\langle D \rangle$. The black traced line represents the exponent equal to 1.0. Figure (d) shows the distribution of SWDP sizes in number of nodes per frequency of occurrence.

N'Djamena, Chad

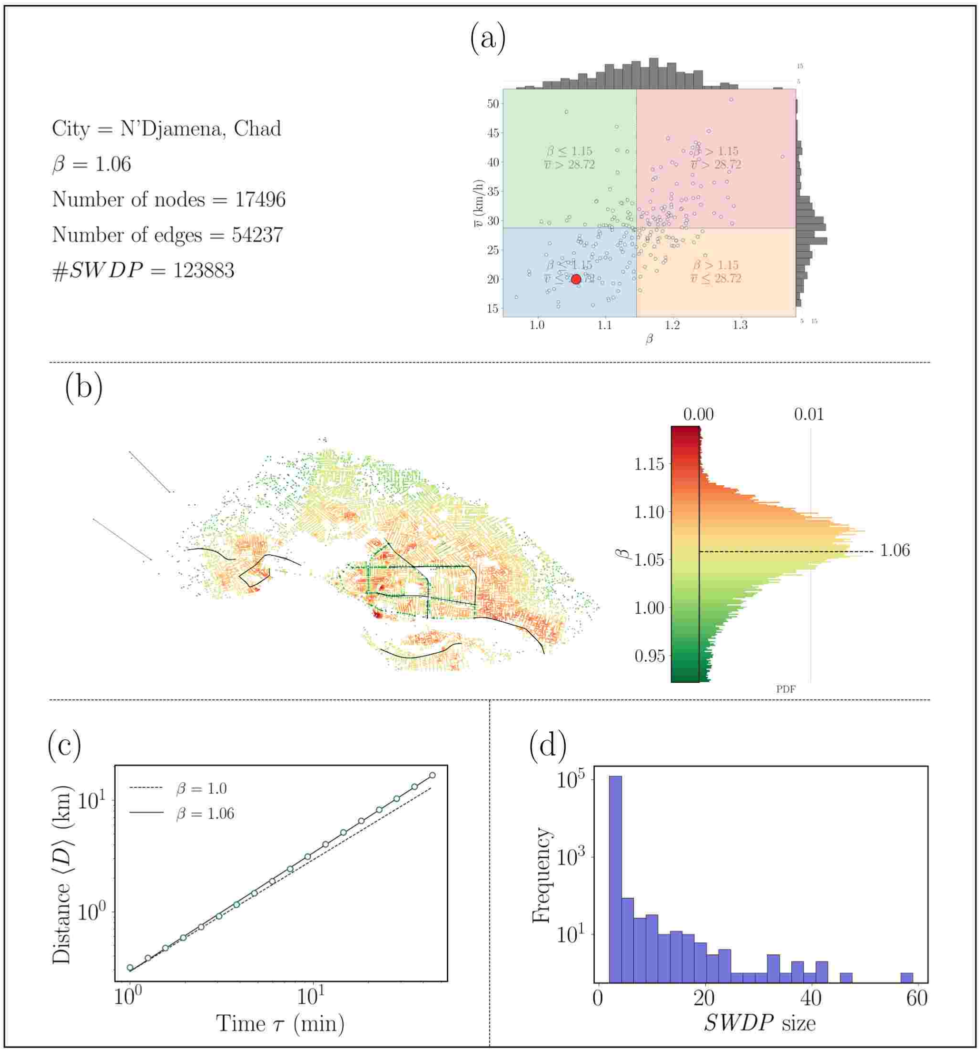

Fig. S117. Methodological sheet - N'Djamena, Chad. In (a) Each point represents a city, with mean exponent (β), on the x-axis, and mean speed $\overline{v}$ obtained in all trips made to calculate the exponent on the axis y. The histograms of the values of β and $\overline{v}$ are shown on the axes in the upper and right corners, respectively. The graph was segmented into four quadrants, in which the division is performed by the mean values of β and $\overline{v}$. The quadrants were colored and annotated according to the division criteria. The red dot represents the location of N'Djamena, Chad. In (b) taking all the nodes of N'Djamena, Chad as origin, the dots are colored as a function of their exponent value and their color is quantified by the color bar in the center. The longest segments without a deceleration point (SWDP) are plotted in black. The probability density function of the $\beta's$ for each experiment is shown on the left of the color scale Figure (c) shows the mean correlation curve between time τ and the distance $\langle D \rangle$. The black traced line represents the exponent equal to 1.0. Figure (d) shows the distribution of SWDP sizes in number of nodes per frequency of occurrence.

Nagpur, India

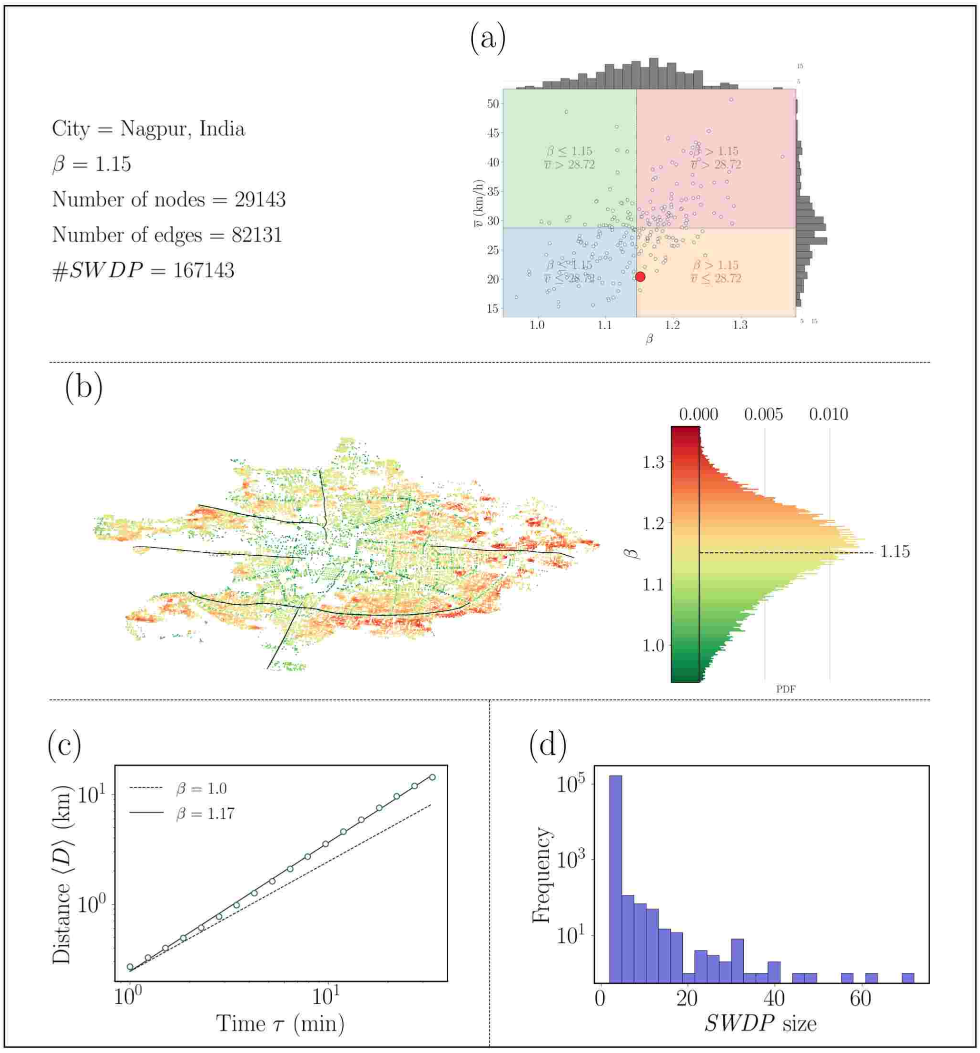

Fig. S118. Methodological sheet - Nagpur, India. In (a) Each point represents a city, with mean exponent (β), on the x-axis, and mean speed $\overline{v}$ obtained in all trips made to calculate the exponent on the axis y. The histograms of the values of β and $\overline{v}$ are shown on the axes in the upper and right corners, respectively. The graph was segmented into four quadrants, in which the division is performed by the mean values of β and $\overline{v}$. The quadrants were colored and annotated according to the division criteria. The red dot represents the location of Nagpur, India. In (b) taking all the nodes of Nagpur, India as origin, the dots are colored as a function of their exponent value and their color is quantified by the color bar in the center. The longest segments without a deceleration point (SWDP) are plotted in black. The probability density function of the $\beta' s$ for each experiment is shown on the left of the color scale Figure (c) shows the mean correlation curve between time τ and the distance $\langle D \rangle$. The black traced line represents the exponent equal to 1.0. Figure (d) shows the distribution of SWDP sizes in number of nodes per frequency of occurrence.

Nairobi, Kenya

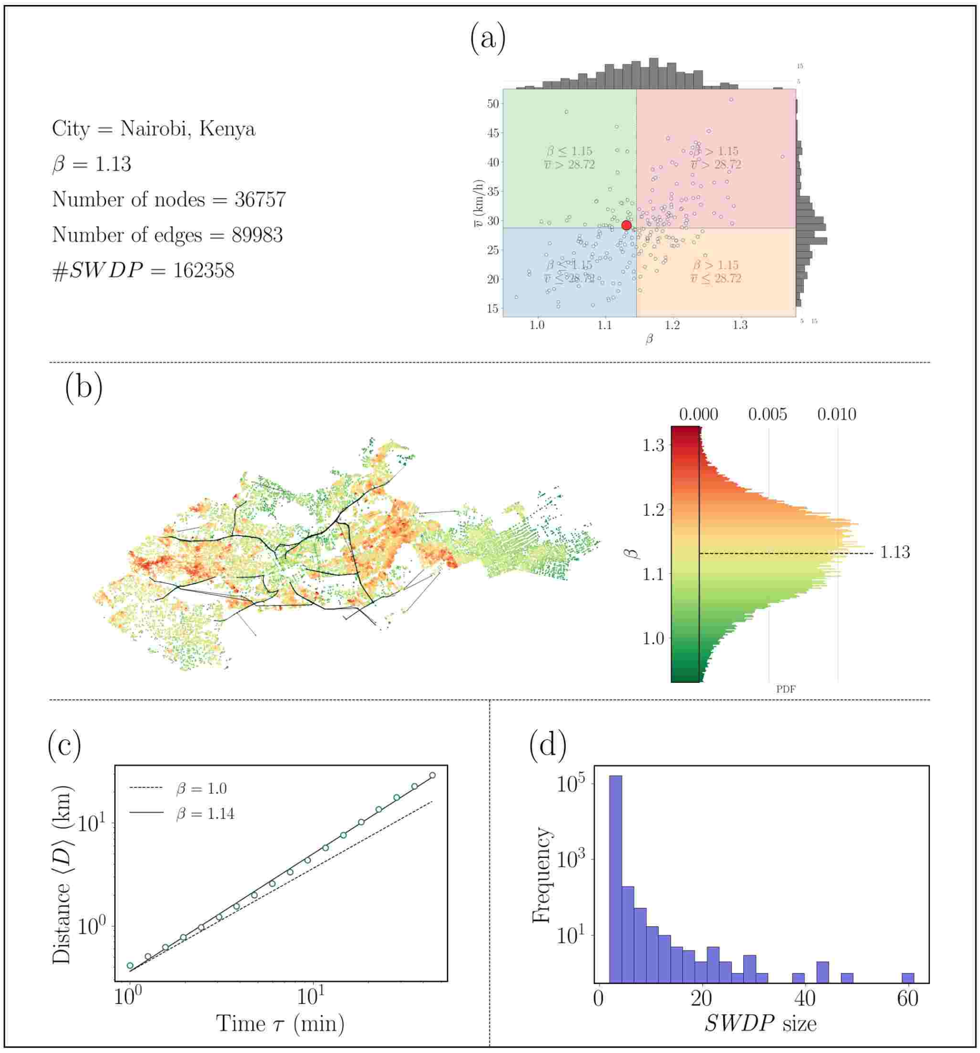

Fig. S119. Methodological sheet - Nairobi, Kenya. In (a) Each point represents a city, with mean exponent (β), on the x-axis, and mean speed $\overline{v}$ obtained in all trips made to calculate the exponent on the axis y. The histograms of the values of β and $\overline{v}$ are shown on the axes in the upper and right corners, respectively. The graph was segmented into four quadrants, in which the division is performed by the mean values of β and $\overline{v}$. The quadrants were colored and annotated according to the division criteria. The red dot represents the location of Nairobi, Kenya. In (b) taking all the nodes of Nairobi, Kenya as origin, the dots are colored as a function of their exponent value and their color is quantified by the color bar in the center. The longest segments without a deceleration point (SWDP) are plotted in black. The probability density function of the $\beta's$ for each experiment is shown on the left of the color scale Figure (c) shows the mean correlation curve between time τ and the distance $\langle D \rangle$. The black traced line represents the exponent equal to 1.0. Figure (d) shows the distribution of SWDP sizes in number of nodes per frequency of occurrence.

Nashville, USA

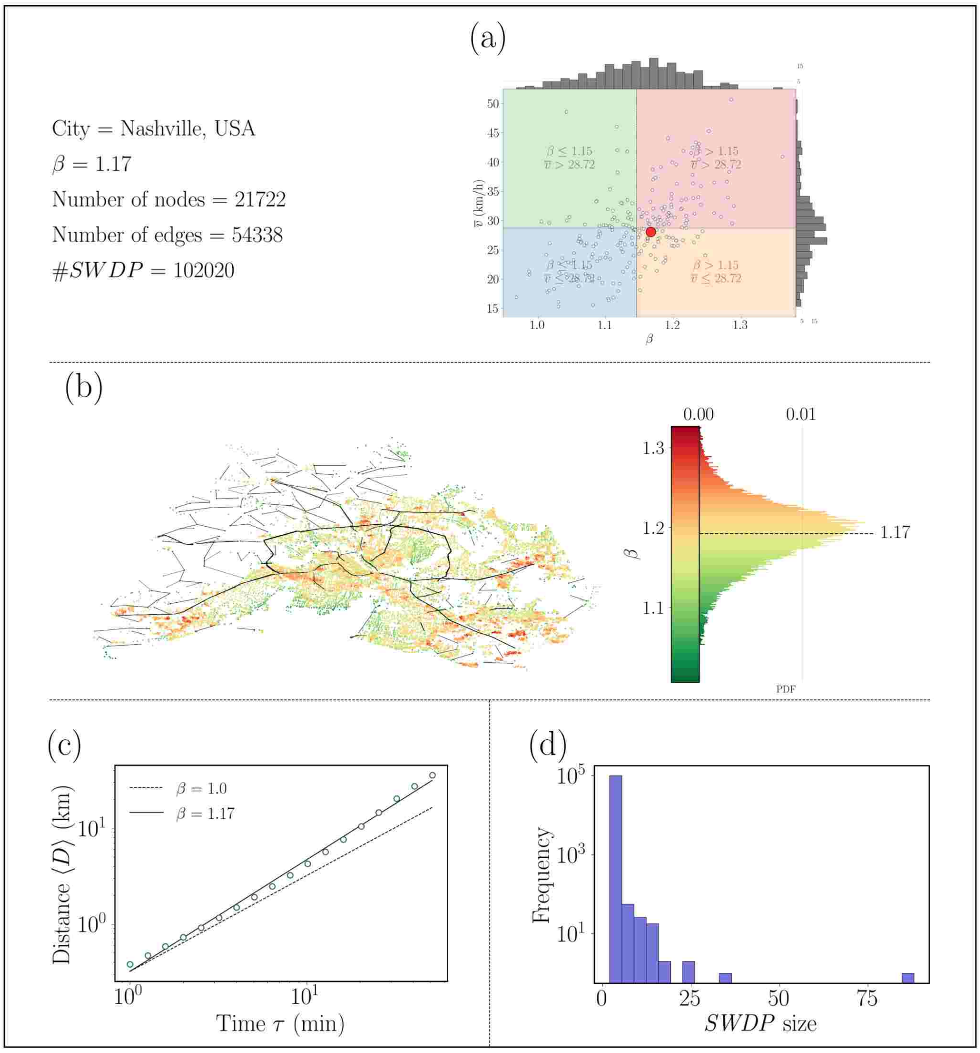

Fig. S120. Methodological sheet - Nashville, USA. In (a) Each point represents a city, with mean exponent (β), on the x-axis, and mean speed $\overline{v}$ obtained in all trips made to calculate the exponent on the axis y. The histograms of the values of β and $\overline{v}$ are shown on the axes in the upper and right corners, respectively. The graph was segmented into four quadrants, in which the division is performed by the mean values of β and $\overline{v}$. The quadrants were colored and annotated according to the division criteria. The red dot represents the location of Nashville, USA. In (b) taking all the nodes of Nashville, USA as origin, the dots are colored as a function of their exponent value and their color is quantified by the color bar in the center. The longest segments without a deceleration point (SWDP) are plotted in black. The probability density function of the $\beta's$ for each experiment is shown on the left of the color scale Figure (c) shows the mean correlation curve between time τ and the distance $\langle D \rangle$. The black traced line represents the exponent equal to 1.0. Figure (d) shows the distribution of SWDP sizes in number of nodes per frequency of occurrence.

Natal, Brasil

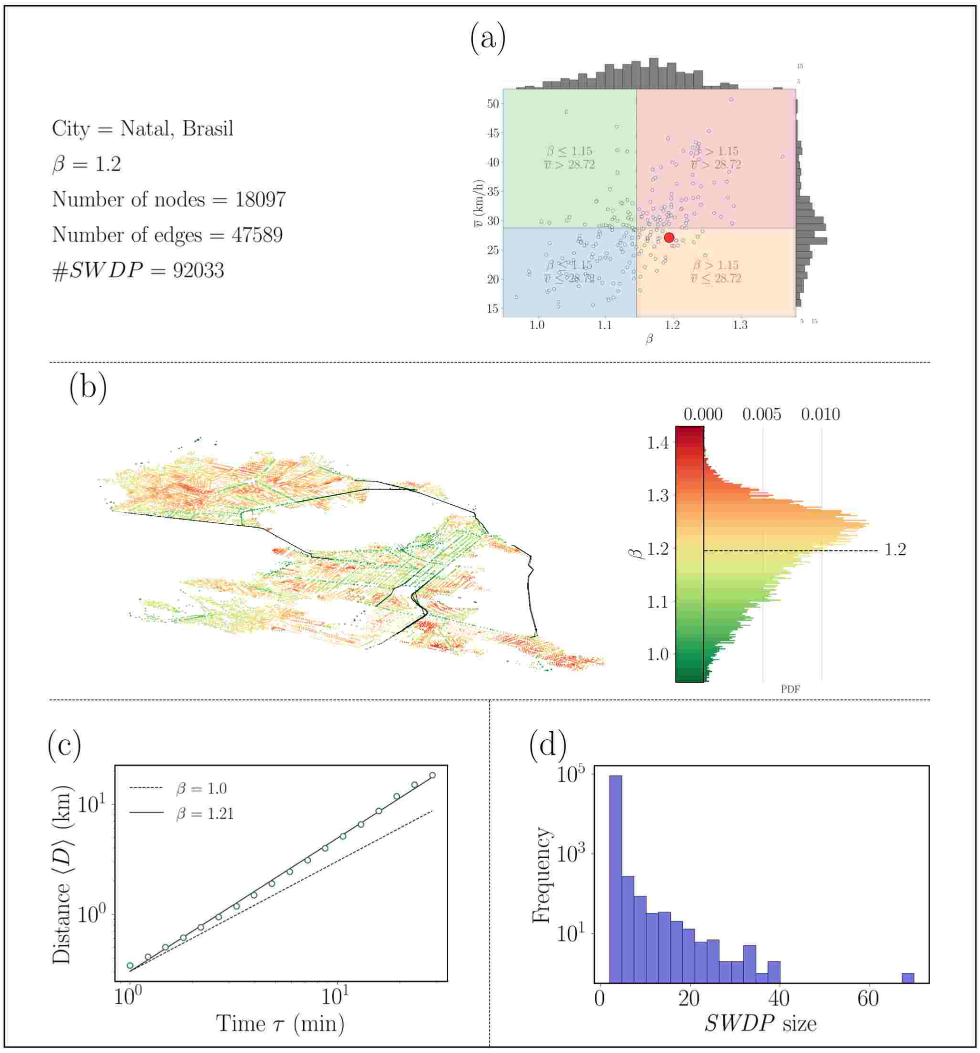

Fig. S121. Methodological sheet - Natal, Brasil. In (a) Each point represents a city, with mean exponent (β), on the x-axis, and mean speed $\overline{v}$ obtained in all trips made to calculate the exponent on the axis y. The histograms of the values of β and $\overline{v}$ are shown on the axes in the upper and right corners, respectively. The graph was segmented into four quadrants, in which the division is performed by the mean values of β and $\overline{v}$. The quadrants were colored and annotated according to the division criteria. The red dot represents the location of Natal, Brasil. In (b) taking all the nodes of Natal, Brasil as origin, the dots are colored as a function of their exponent value and their color is quantified by the color bar in the center. The longest segments without a deceleration point (SWDP) are plotted in black. The probability density function of the $\beta's$ for each experiment is shown on the left of the color scale Figure (c) shows the mean correlation curve between time τ and the distance $\langle D \rangle$. The black traced line represents the exponent equal to 1.0. Figure (d) shows the distribution of SWDP sizes in number of nodes per frequency of occurrence.

New Delhi, India

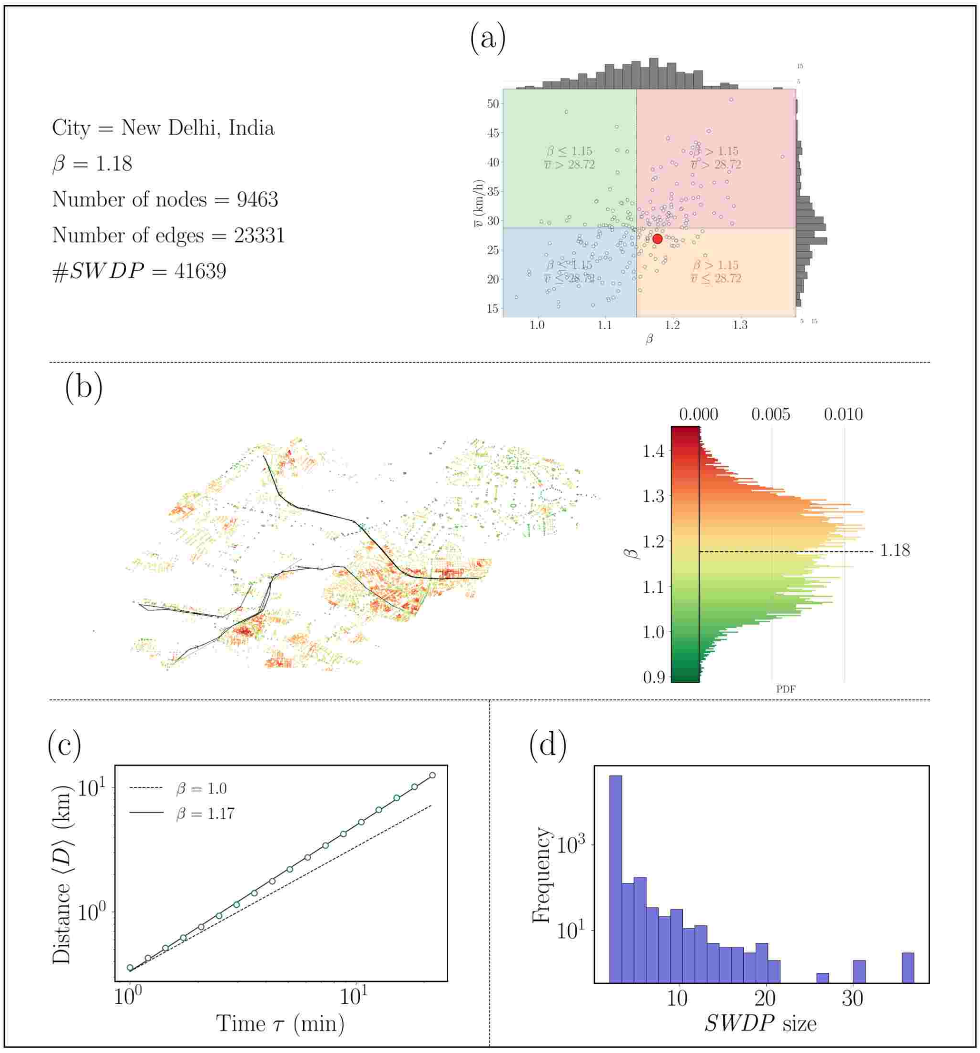

Fig. S122. Methodological sheet - New Delhi, India. In (a) Each point represents a city, with mean exponent (β), on the x-axis, and mean speed $\overline{v}$ obtained in all trips made to calculate the exponent on the axis y. The histograms of the values of β and $\overline{v}$ are shown on the axes in the upper and right corners, respectively. The graph was segmented into four quadrants, in which the division is performed by the mean values of β and $\overline{v}$. The quadrants were colored and annotated according to the division criteria. The red dot represents the location of New Delhi, India. In (b) taking all the nodes of New Delhi, India as origin, the dots are colored as a function of their exponent value and their color is quantified by the color bar in the center. The longest segments without a deceleration point (SWDP) are plotted in black. The probability density function of the $\beta' s$ for each experiment is shown on the left of the color scale Figure (c) shows the mean correlation curve between time τ and the distance $\langle D \rangle$. The black traced line represents the exponent equal to 1.0. Figure (d) shows the distribution of SWDP sizes in number of nodes per frequency of occurrence.

New Orleans, USA

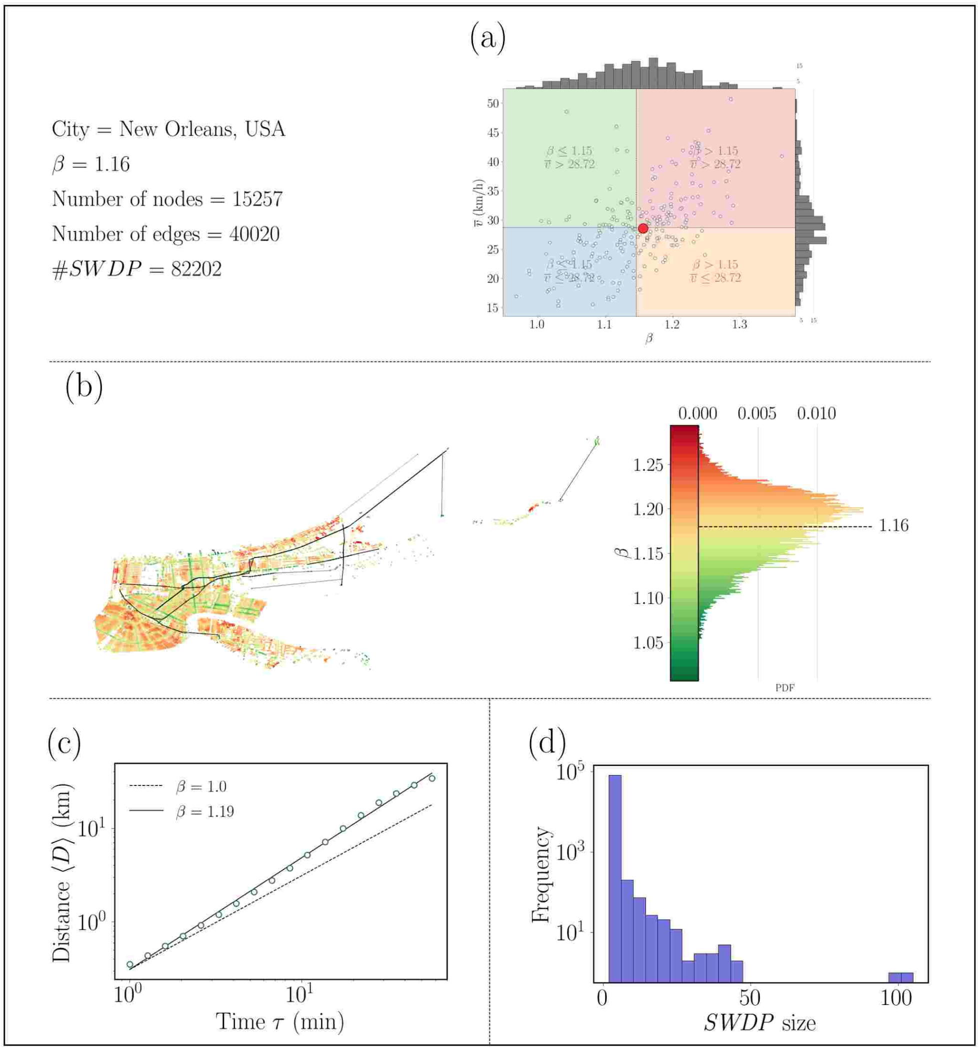

Fig. S123. Methodological sheet - New Orleans, USA. In (a) Each point represents a city, with mean exponent (β), on the x-axis, and mean speed $\overline{v}$ obtained in all trips made to calculate the exponent on the axis y. The histograms of the values of β and $\overline{v}$ are shown on the axes in the upper and right corners, respectively. The graph was segmented into four quadrants, in which the division is performed by the mean values of β and $\overline{v}$. The quadrants were colored and annotated according to the division criteria. The red dot represents the location of New Orleans, USA. In (b) taking all the nodes of New Orleans, USA as origin, the dots are colored as a function of their exponent value and their color is quantified by the color bar in the center. The longest segments without a deceleration point (SWDP) are plotted in black. The probability density function of the β'^s for each experiment is shown on the left of the color scale Figure (c) shows the mean correlation curve between time τ and the distance $\langle D \rangle$. The black traced line represents the exponent equal to 1.0. Figure (d) shows the distribution of SWDP sizes in number of nodes per frequency of occurrence.

New York, USA

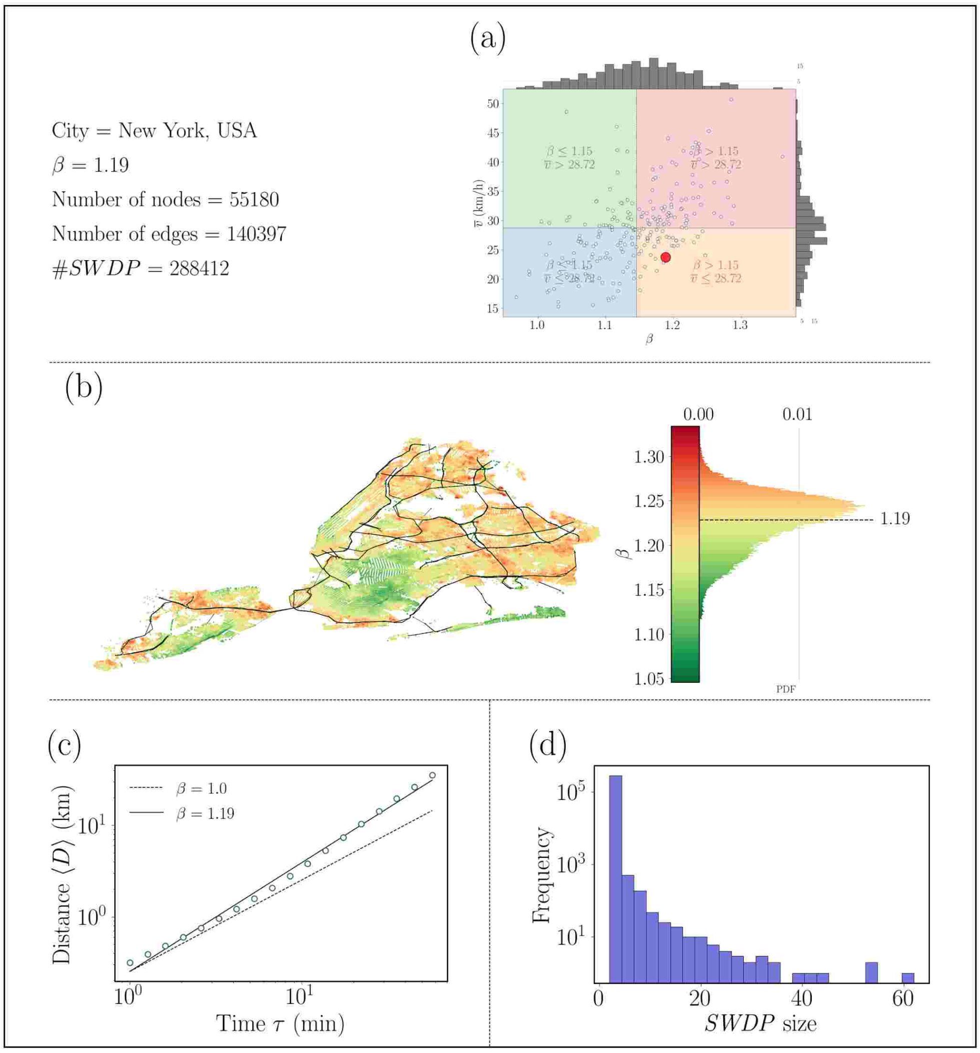

Fig. S124. Methodological sheet - New York, USA. In (a) Each point represents a city, with mean exponent (β), on the x-axis, and mean speed $\overline{v}$ obtained in all trips made to calculate the exponent on the axis y. The histograms of the values of β and $\overline{v}$ are shown on the axes in the upper and right corners, respectively. The graph was segmented into four quadrants, in which the division is performed by the mean values of β and $\overline{v}$. The quadrants were colored and annotated according to the division criteria. The red dot represents the location of New York, USA. In (b) taking all the nodes of New York, USA as origin, the dots are colored as a function of their exponent value and their color is quantified by the color bar in the center. The longest segments without a deceleration point (SWDP) are plotted in black. The probability density function of the $\beta's$ for each experiment is shown on the left of the color scale Figure (c) shows the mean correlation curve between time τ and the distance $\langle D \rangle$. The black traced line represents the exponent equal to 1.0. Figure (d) shows the distribution of SWDP sizes in number of nodes per frequency of occurrence.

Nice, France

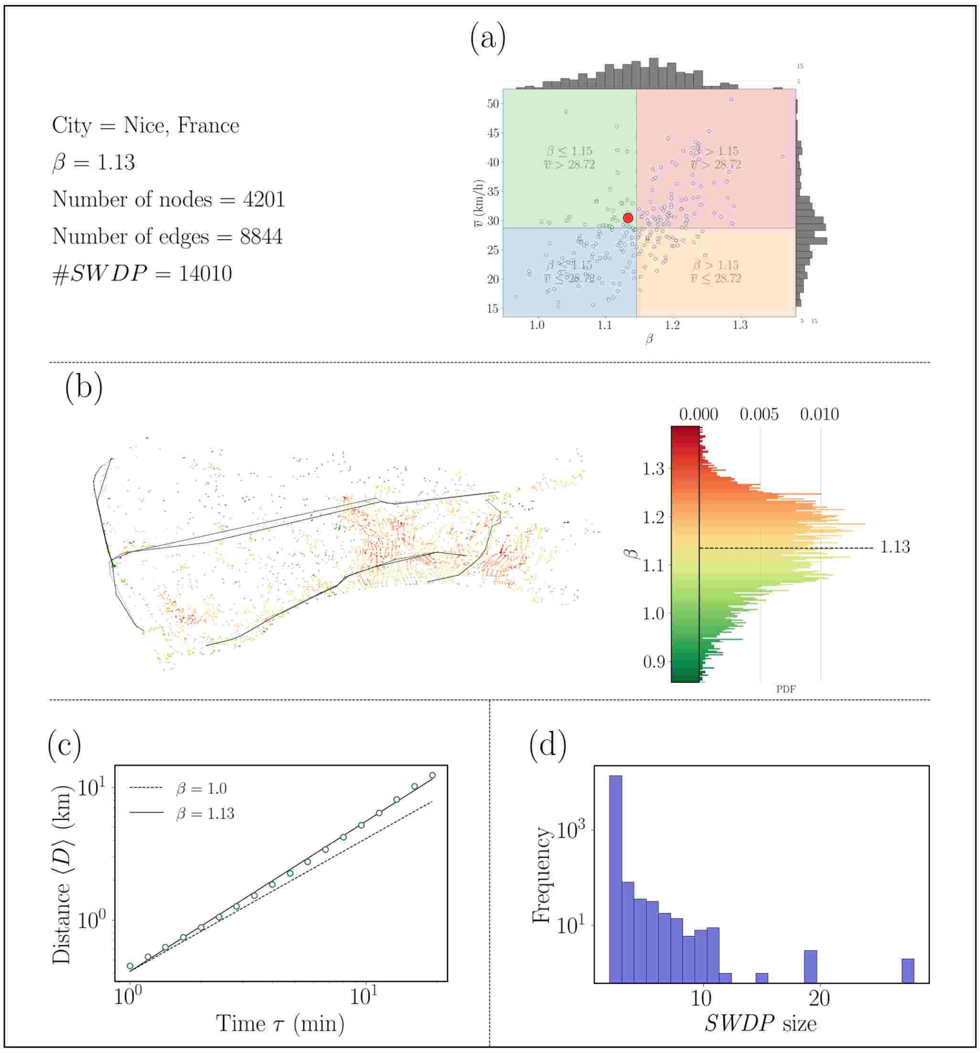

Fig. S125. Methodological sheet - Nice, France. In (a) Each point represents a city, with mean exponent (β), on the x-axis, and mean speed $\overline{v}$ obtained in all trips made to calculate the exponent on the axis y. The histograms of the values of β and $\overline{v}$ are shown on the axes in the upper and right corners, respectively. The graph was segmented into four quadrants, in which the division is performed by the mean values of β and $\overline{v}$. The quadrants were colored and annotated according to the division criteria. The red dot represents the location of Nice, France. In (b) taking all the nodes of Nice, France as origin, the dots are colored as a function of their exponent value and their color is quantified by the color bar in the center. The longest segments without a deceleration point (SWDP) are plotted in black. The probability density function of the $\beta's$ for each experiment is shown on the left of the color scale Figure (c) shows the mean correlation curve between time τ and the distance $\langle D \rangle$. The black traced line represents the exponent equal to 1.0. Figure (d) shows the distribution of SWDP sizes in number of nodes per frequency of occurrence.

Norwich, UK

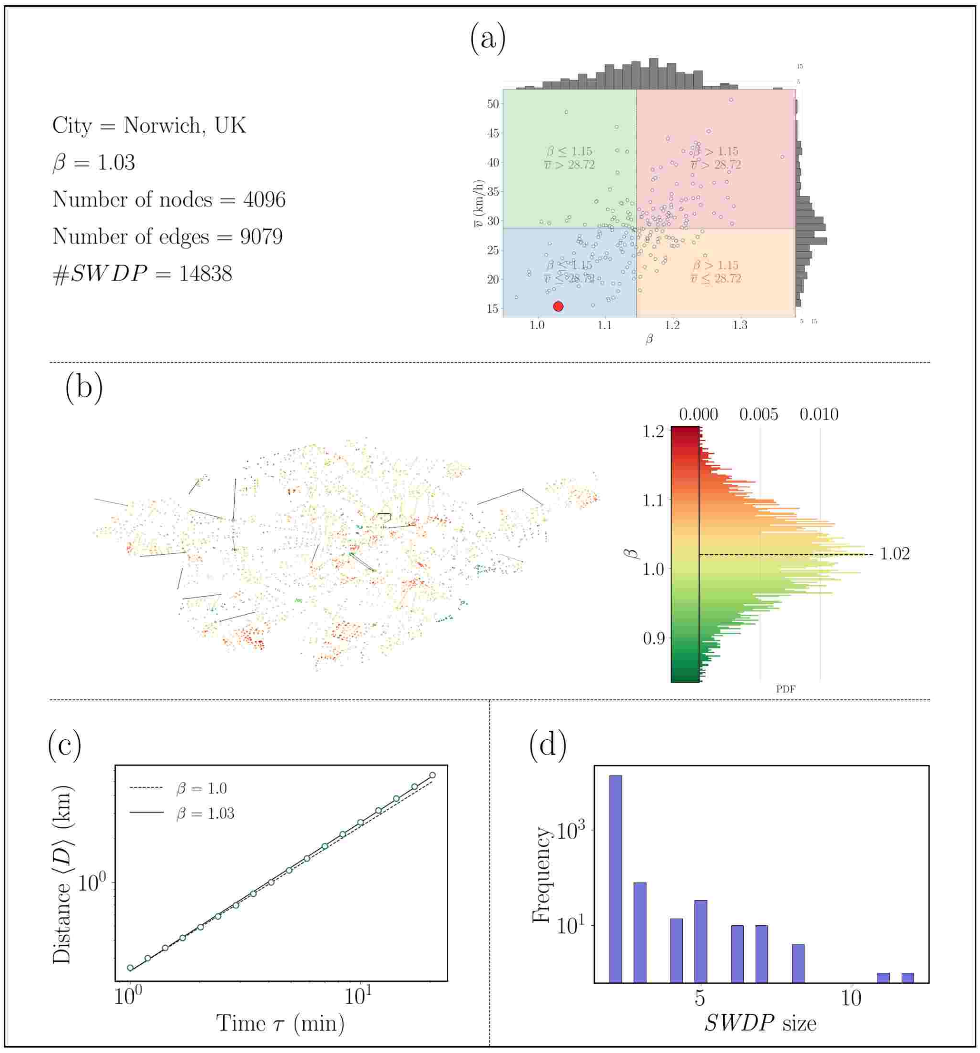

Fig. S126. Methodological sheet - Norwich, UK. In (a) Each point represents a city, with mean exponent (β), on the x-axis, and mean speed $\overline{v}$ obtained in all trips made to calculate the exponent on the axis y. The histograms of the values of β and $\overline{v}$ are shown on the axes in the upper and right corners, respectively. The graph was segmented into four quadrants, in which the division is performed by the mean values of β and $\overline{v}$. The quadrants were colored and annotated according to the division criteria. The red dot represents the location of Norwich, UK. In (b) taking all the nodes of Norwich, UK as origin, the dots are colored as a function of their exponent value and their color is quantified by the color bar in the center. The longest segments without a deceleration point (SWDP) are plotted in black. The probability density function of the $\beta's$ for each experiment is shown on the left of the color scale Figure (c) shows the mean correlation curve between time τ and the distance $\langle D \rangle$. The black traced line represents the exponent equal to 1.0. Figure (d) shows the distribution of SWDP sizes in number of nodes per frequency of occurrence.

Nottingham, UK

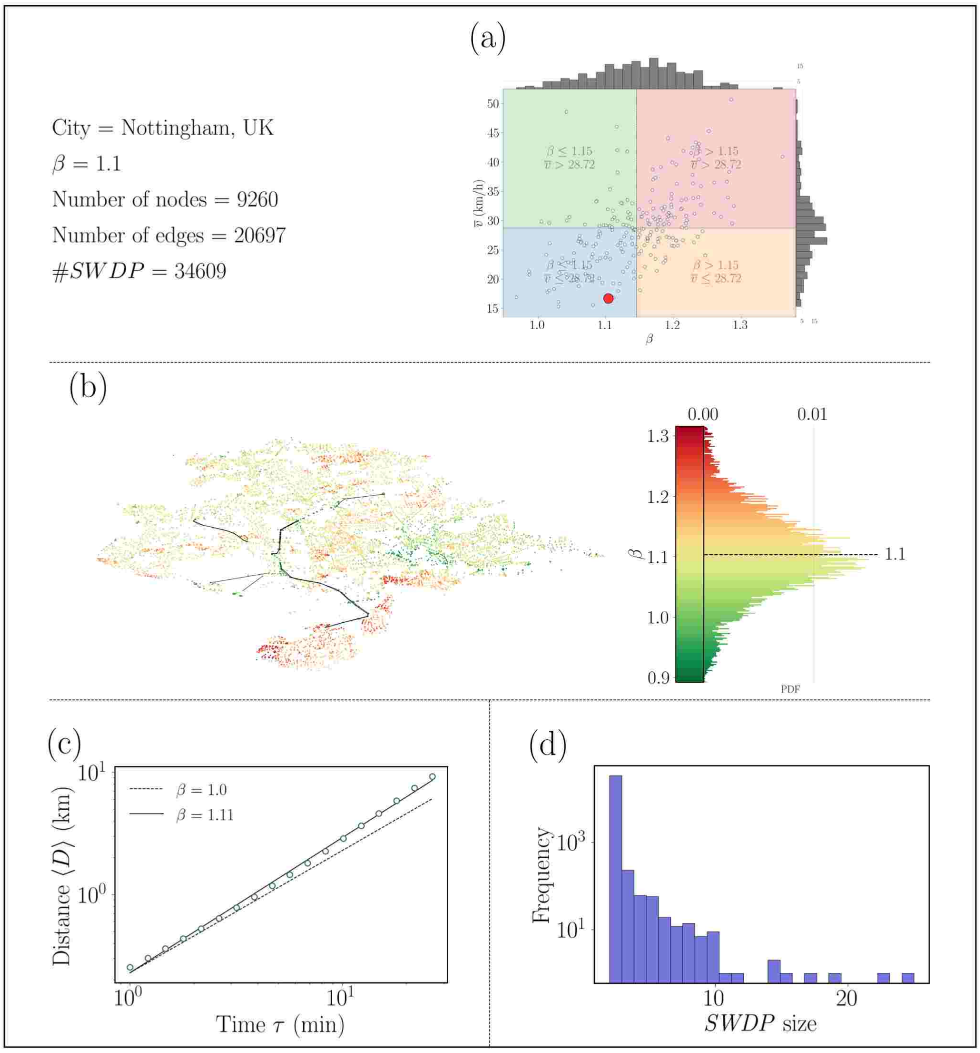

Fig. S127. Methodological sheet - Nottingham, UK. In (a) Each point represents a city, with mean exponent (β), on the x-axis, and mean speed $\overline{v}$ obtained in all trips made to calculate the exponent on the axis y. The histograms of the values of β and $\overline{v}$ are shown on the axes in the upper and right corners, respectively. The graph was segmented into four quadrants, in which the division is performed by the mean values of β and $\overline{v}$. The quadrants were colored and annotated according to the division criteria. The red dot represents the location of Nottingham, UK. In (b) taking all the nodes of Nottingham, UK as origin, the dots are colored as a function of their exponent value and their color is quantified by the color bar in the center. The longest segments without a deceleration point (SWDP) are plotted in black. The probability density function of the $\beta's$ for each experiment is shown on the left of the color scale Figure (c) shows the mean correlation curve between time τ and the distance $\langle D \rangle$. The black traced line represents the exponent equal to 1.0. Figure (d) shows the distribution of SWDP sizes in number of nodes per frequency of occurrence.

Nur-Sultan, Kazakhstan

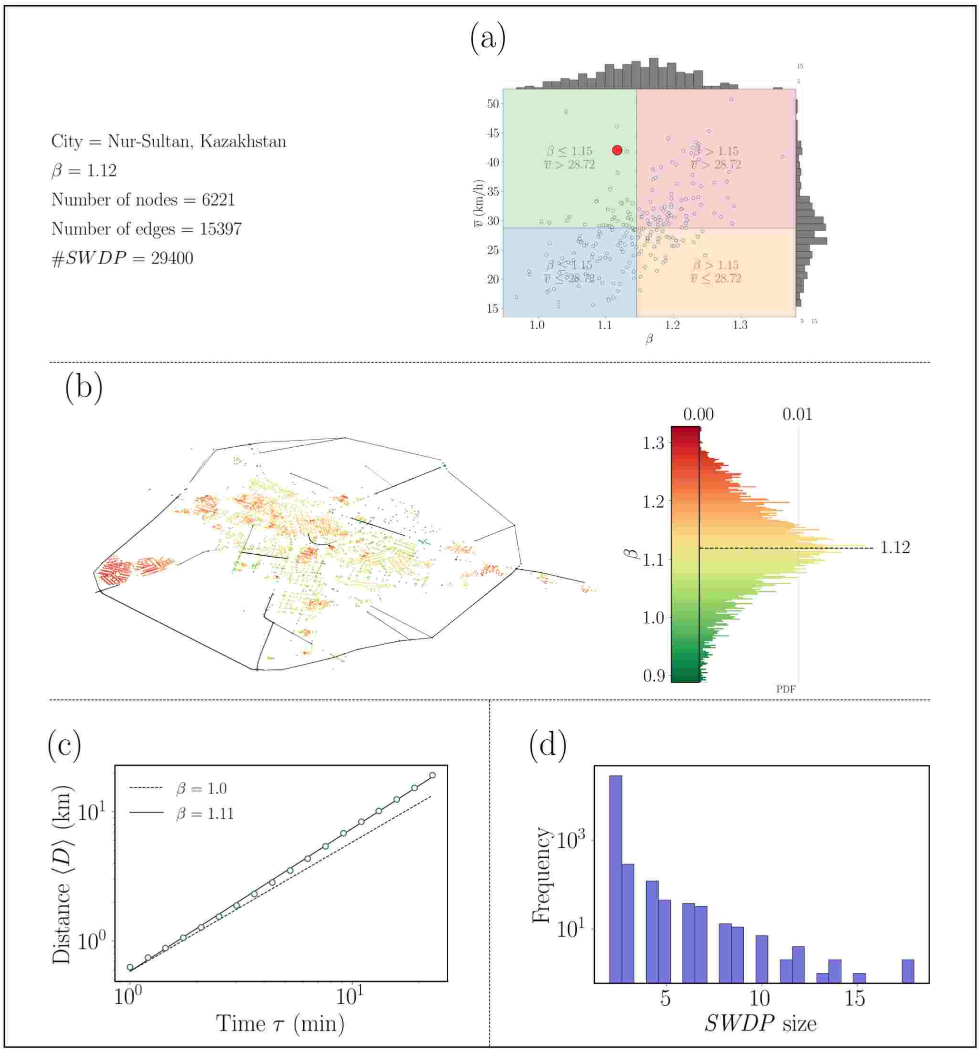

Fig. S128. Methodological sheet - Nur-Sultan, Kazakhstan. In (a) Each point represents a city, with mean exponent (β), on the x-axis, and mean speed $\overline{v}$ obtained in all trips made to calculate the exponent on the axis y. The histograms of the values of β and $\overline{v}$ are shown on the axes in the upper and right corners, respectively. The graph was segmented into four quadrants, in which the division is performed by the mean values of β and $\overline{v}$. The quadrants were colored and annotated according to the division criteria. The red dot represents the location of Nur-Sultan, Kazakhstan. In (b) taking all the nodes of Nur-Sultan, Kazakhstan as origin, the dots are colored as a function of their exponent value and their color is quantified by the color bar in the center. The longest segments without a deceleration point (SWDP) are plotted in black. The probability density function of the $\beta's$ for each experiment is shown on the left of the color scale Figure (c) shows the mean correlation curve between time τ and the distance $\langle D \rangle$. The black traced line represents the exponent equal to 1.0. Figure (d) shows the distribution of SWDP sizes in number of nodes per frequency of occurrence.

Nuremberg, Germany

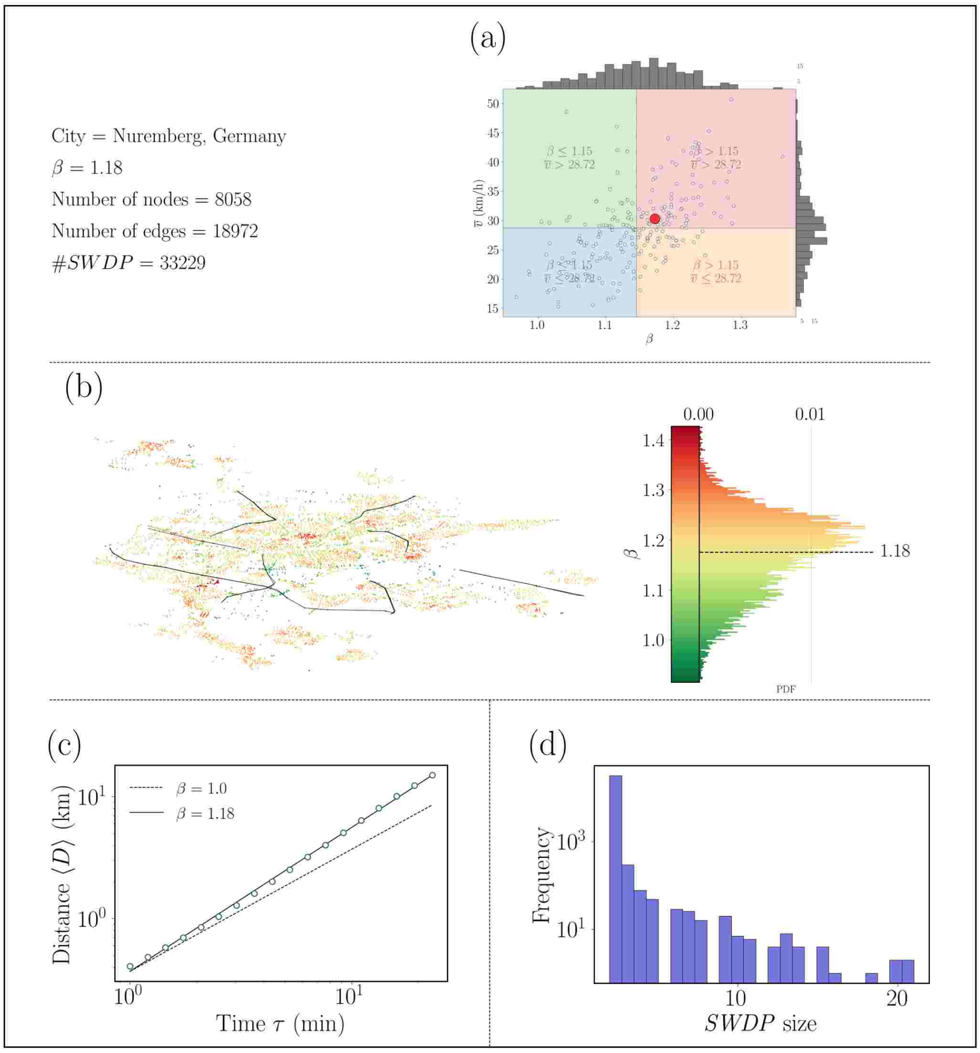

Fig. S129. Methodological sheet - Nuremberg, Germany. In (a) Each point represents a city, with mean exponent (β), on the x-axis, and mean speed $\overline{v}$ obtained in all trips made to calculate the exponent on the axis y. The histograms of the values of β and $\overline{v}$ are shown on the axes in the upper and right corners, respectively. The graph was segmented into four quadrants, in which the division is performed by the mean values of β and $\overline{v}$. The quadrants were colored and annotated according to the division criteria. The red dot represents the location of Nuremberg, Germany. In (b) taking all the nodes of Nuremberg, Germany as origin, the dots are colored as a function of their exponent value and their color is quantified by the color bar in the center. The longest segments without a deceleration point (SWDP) are plotted in black. The probability density function of the $\beta's$ for each experiment is shown on the left of the color scale Figure (c) shows the mean correlation curve between time τ and the distance $\langle D \rangle$. The black traced line represents the exponent equal to 1.0. Figure (d) shows the distribution of SWDP sizes in number of nodes per frequency of occurrence.

Oakland, California, USA

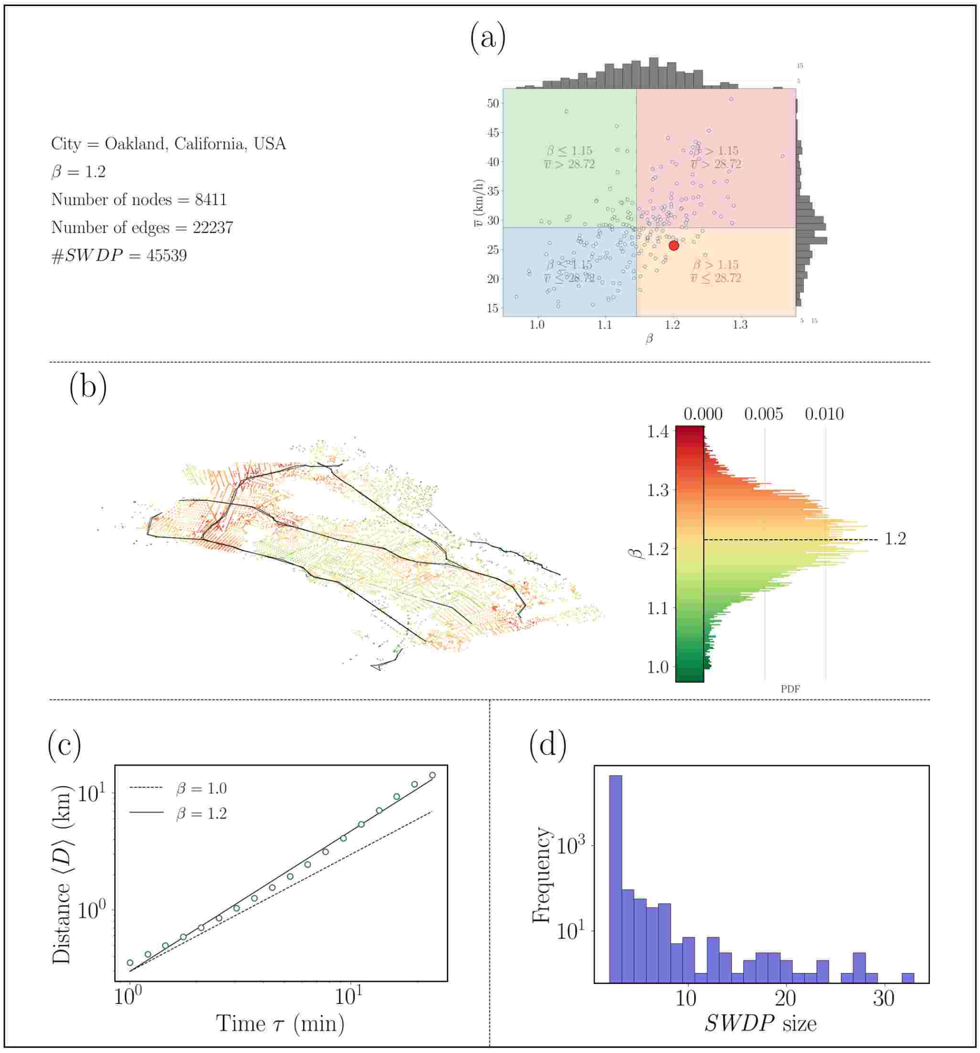

Fig. S130. Methodological sheet - Oakland, California, USA. In (a) Each point represents a city, with mean exponent (β), on the x-axis, and mean speed $\overline{v}$ obtained in all trips made to calculate the exponent on the axis y. The histograms of the values of β and $\overline{v}$ are shown on the axes in the upper and right corners, respectively. The graph was segmented into four quadrants, in which the division is performed by the mean values of β and $\overline{v}$. The quadrants were colored and annotated according to the division criteria. The red dot represents the location of Oakland, California, USA. In (b) taking all the nodes of Oakland, California, USA as origin, the dots are colored as a function of their exponent value and their color is quantified by the color bar in the center. The longest segments without a deceleration point (SWDP) are plotted in black. The probability density function of the $\beta's$ for each experiment is shown on the left of the color scale Figure (c) shows the mean correlation curve between time τ and the distance $\langle D \rangle$. The black traced line represents the exponent equal to 1.0. Figure (d) shows the distribution of SWDP sizes in number of nodes per frequency of occurrence.

Oklahoma City, USA

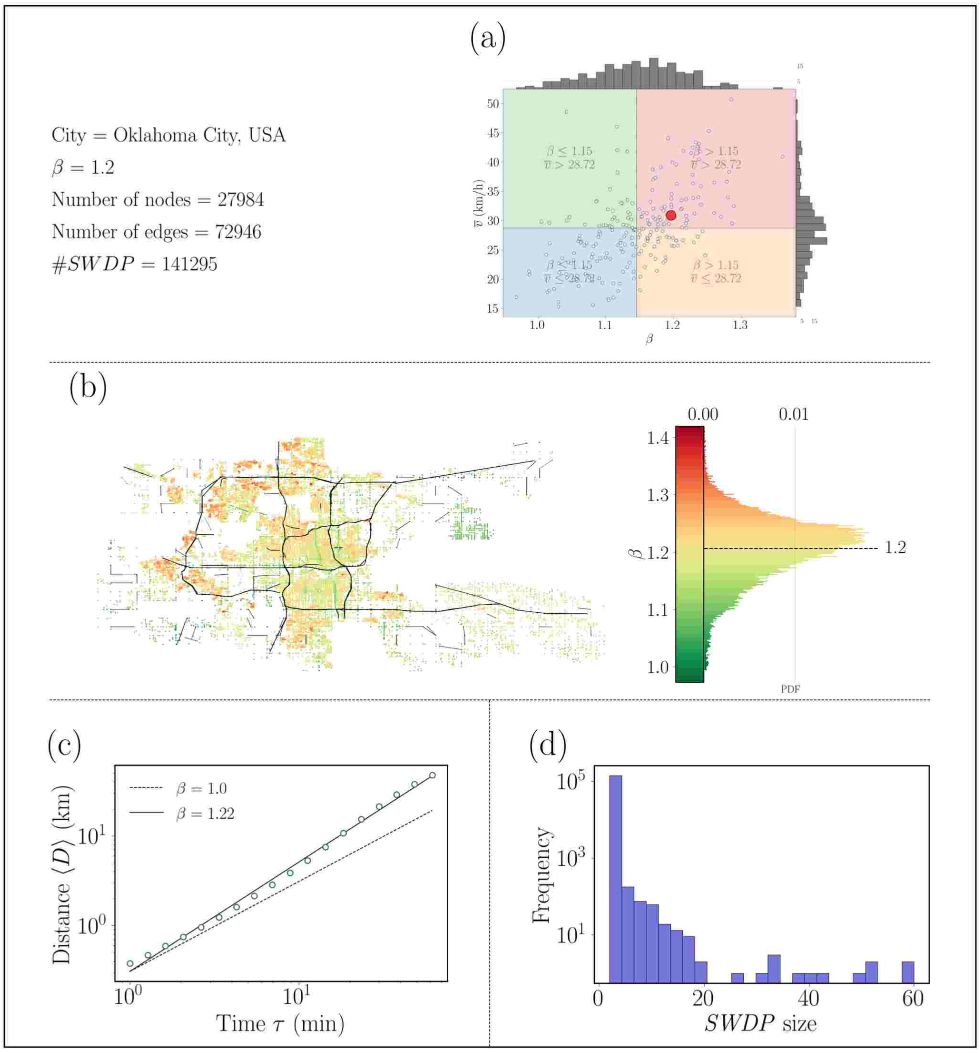

Fig. S131. Methodological sheet - Oklahoma City, USA. In (a) Each point represents a city, with mean exponent (β), on the x-axis, and mean speed $\overline{v}$ obtained in all trips made to calculate the exponent on the axis y. The histograms of the values of β and $\overline{v}$ are shown on the axes in the upper and right corners, respectively. The graph was segmented into four quadrants, in which the division is performed by the mean values of β and $\overline{v}$. The quadrants were colored and annotated according to the division criteria. The red dot represents the location of Oklahoma City, USA. In (b) taking all the nodes of Oklahoma City, USA as origin, the dots are colored as a function of their exponent value and their color is quantified by the color bar in the center. The longest segments without a deceleration point (SWDP) are plotted in black. The probability density function of the $\beta's$ for each experiment is shown on the left of the color scale Figure (c) shows the mean correlation curve between time τ and the distance $\langle D \rangle$. The black traced line represents the exponent equal to 1.0. Figure (d) shows the distribution of SWDP sizes in number of nodes per frequency of occurrence.

Orlando, USA

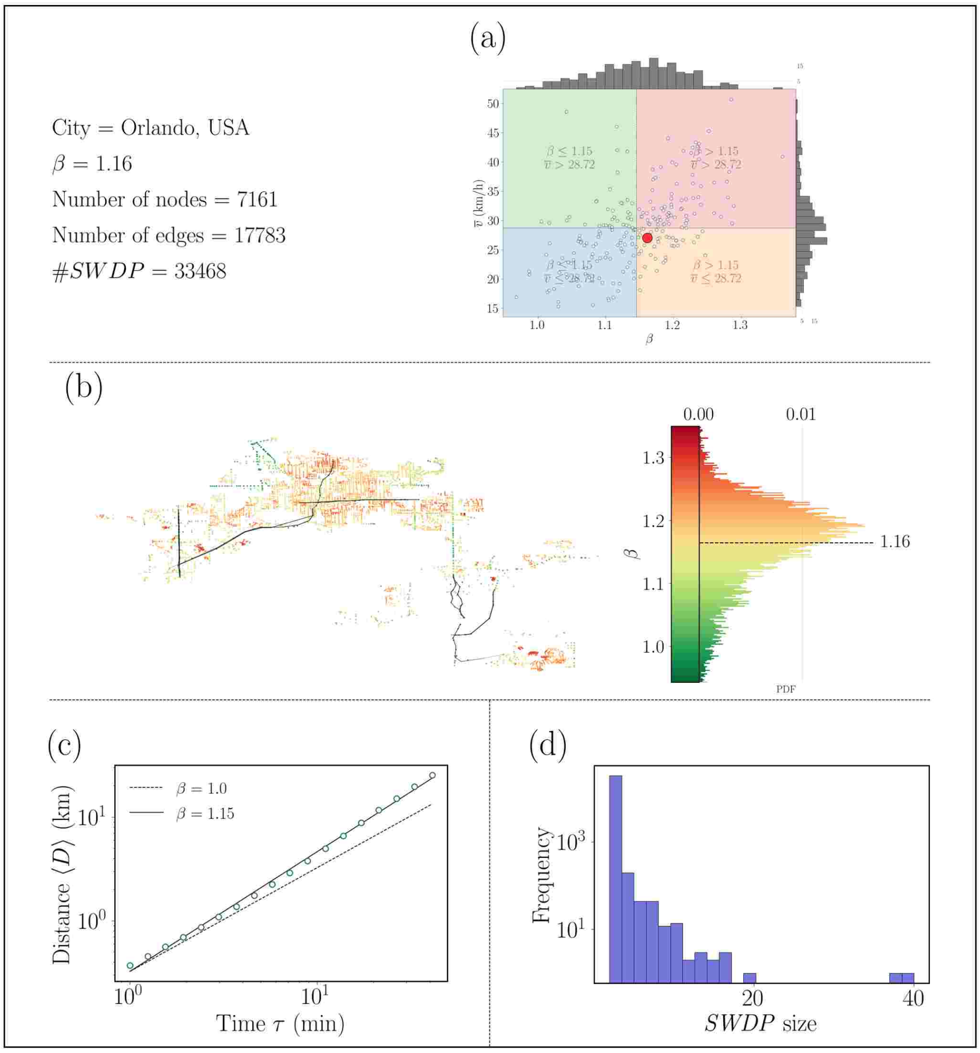

Fig. S132. Methodological sheet - Orlando, USA. In (a) Each point represents a city, with mean exponent (β), on the x-axis, and mean speed $\overline{v}$ obtained in all trips made to calculate the exponent on the axis y. The histograms of the values of β and $\overline{v}$ are shown on the axes in the upper and right corners, respectively. The graph was segmented into four quadrants, in which the division is performed by the mean values of β and $\overline{v}$. The quadrants were colored and annotated according to the division criteria. The red dot represents the location of Orlando, USA. In (b) taking all the nodes of Orlando, USA as origin, the dots are colored as a function of their exponent value and their color is quantified by the color bar in the center. The longest segments without a deceleration point (SWDP) are plotted in black. The probability density function of the $\beta's$ for each experiment is shown on the left of the color scale Figure (c) shows the mean correlation curve between time τ and the distance $\langle D \rangle$. The black traced line represents the exponent equal to 1.0. Figure (d) shows the distribution of SWDP sizes in number of nodes per frequency of occurrence.

Oslo,Norway

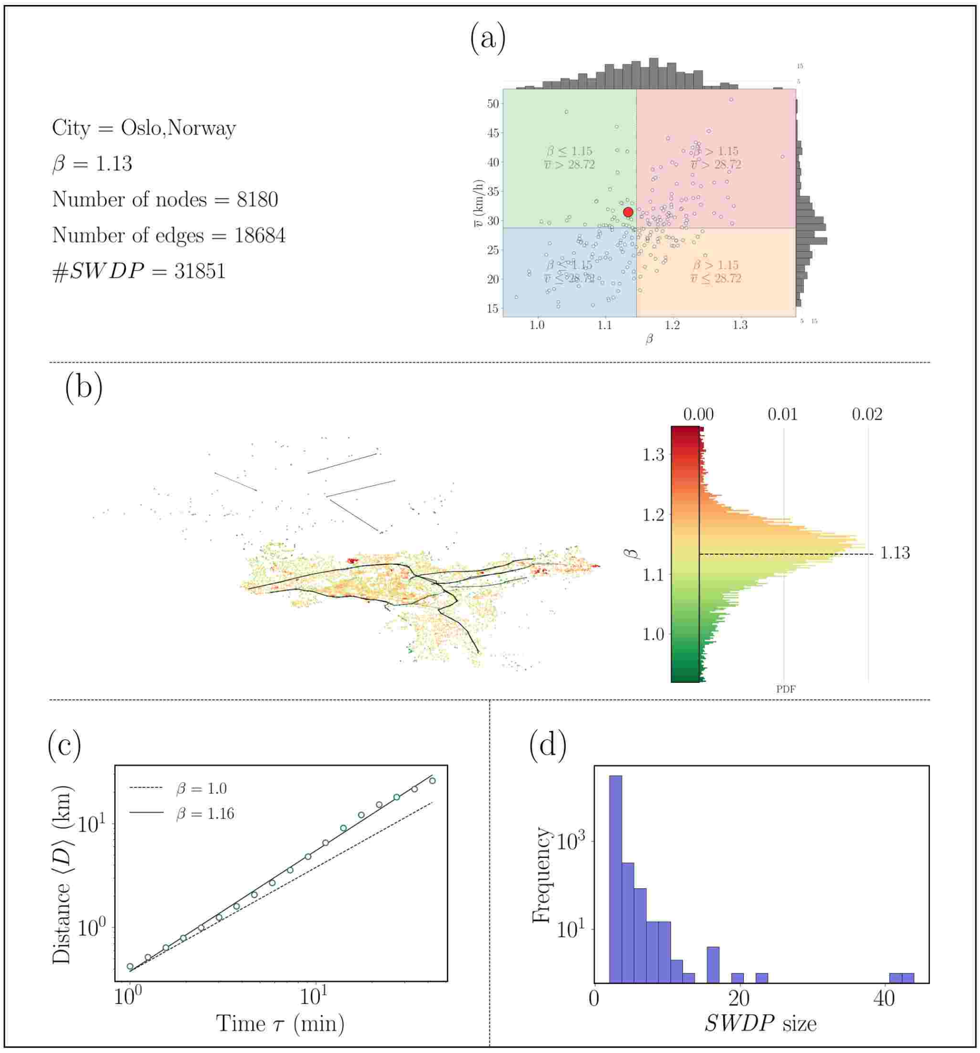

Fig. S133. Methodological sheet - Oslo,Norway. In (a) Each point represents a city, with mean exponent (β), on the x-axis, and mean speed $\overline{v}$ obtained in all trips made to calculate the exponent on the axis y. The histograms of the values of β and $\overline{v}$ are shown on the axes in the upper and right corners, respectively. The graph was segmented into four quadrants, in which the division is performed by the mean values of β and $\overline{v}$. The quadrants were colored and annotated according to the division criteria. The red dot represents the location of Oslo,Norway. In (b) taking all the nodes of Oslo,Norway as origin, the dots are colored as a function of their exponent value and their color is quantified by the color bar in the center. The longest segments without a deceleration point (SWDP) are plotted in black. The probability density function of the $\beta's$ for each experiment is shown on the left of the color scale Figure (c) shows the mean correlation curve between time τ and the distance $\langle D \rangle$. The black traced line represents the exponent equal to 1.0. Figure (d) shows the distribution of SWDP sizes in number of nodes per frequency of occurrence.

Ostrava, Czechia

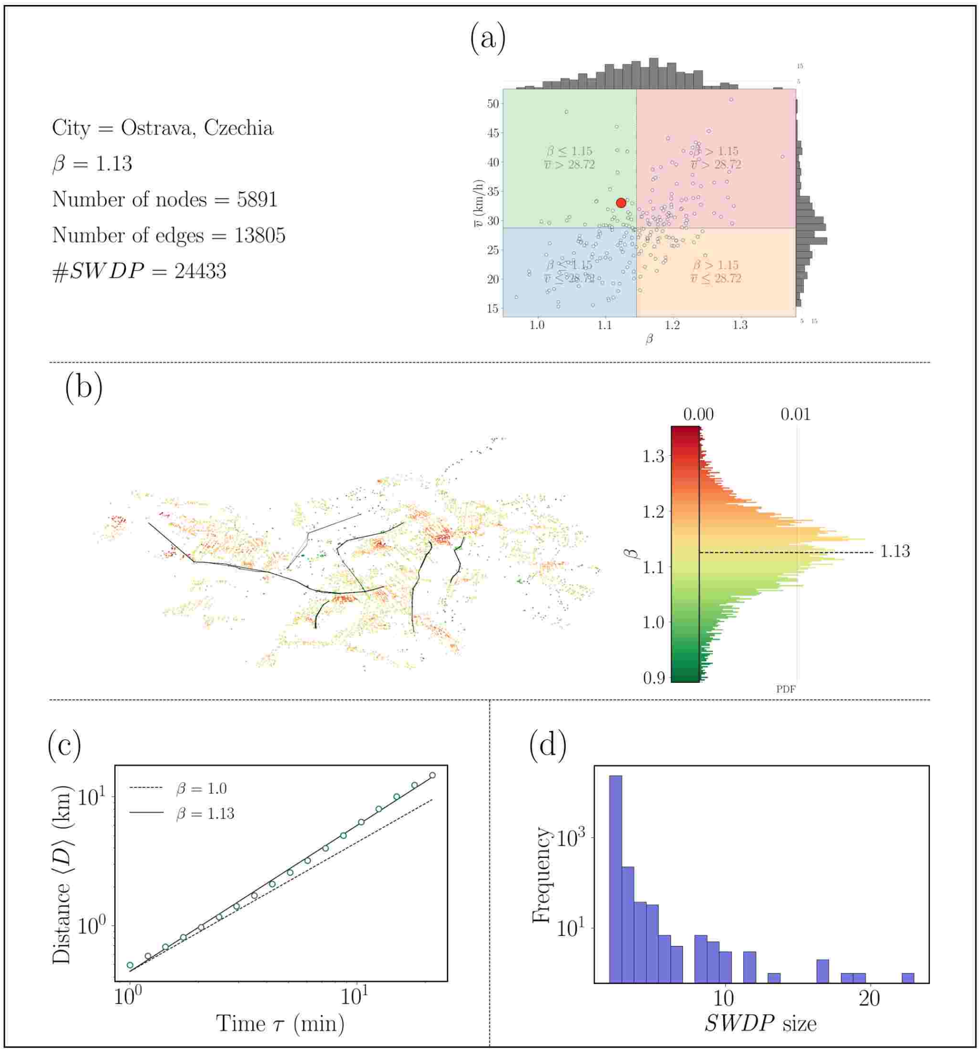

Fig. S134. Methodological sheet - Ostrava, Czechia. In (a) Each point represents a city, with mean exponent (β), on the x-axis, and mean speed $\overline{v}$ obtained in all trips made to calculate the exponent on the axis y. The histograms of the values of β and $\overline{v}$ are shown on the axes in the upper and right corners, respectively. The graph was segmented into four quadrants, in which the division is performed by the mean values of β and $\overline{v}$. The quadrants were colored and annotated according to the division criteria. The red dot represents the location of Ostrava, Czechia. In (b) taking all the nodes of Ostrava, Czechia as origin, the dots are colored as a function of their exponent value and their color is quantified by the color bar in the center. The longest segments without a deceleration point (SWDP) are plotted in black. The probability density function of the $\beta's$ for each experiment is shown on the left of the color scale Figure (c) shows the mean correlation curve between time τ and the distance $\langle D \rangle$. The black traced line represents the exponent equal to 1.0. Figure (d) shows the distribution of SWDP sizes in number of nodes per frequency of occurrence.

Oxford, UK

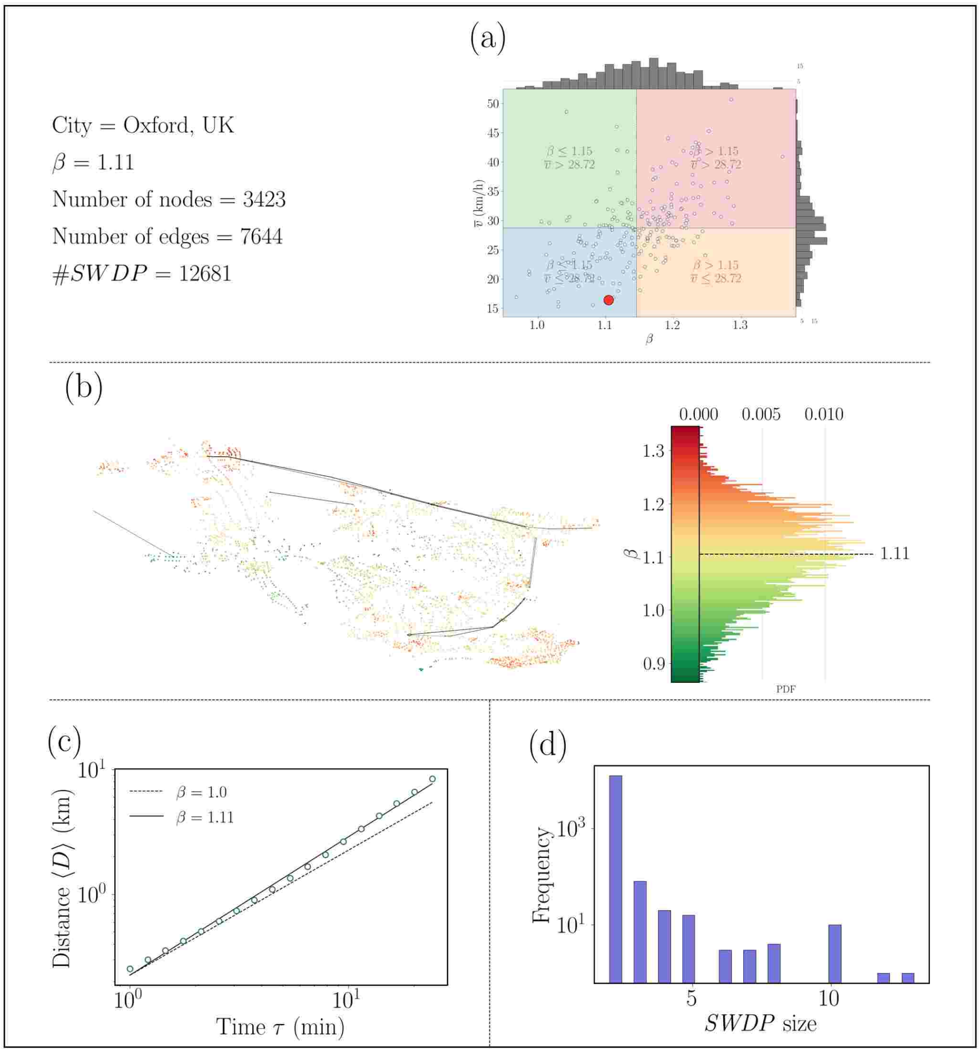

Fig. S135. Methodological sheet - Oxford, UK. In (a) Each point represents a city, with mean exponent (β), on the x-axis, and mean speed $\overline{v}$ obtained in all trips made to calculate the exponent on the axis y. The histograms of the values of β and $\overline{v}$ are shown on the axes in the upper and right corners, respectively. The graph was segmented into four quadrants, in which the division is performed by the mean values of β and $\overline{v}$. The quadrants were colored and annotated according to the division criteria. The red dot represents the location of Oxford, UK. In (b) taking all the nodes of Oxford, UK as origin, the dots are colored as a function of their exponent value and their color is quantified by the color bar in the center. The longest segments without a deceleration point (SWDP) are plotted in black. The probability density function of the $\beta's$ for each experiment is shown on the left of the color scale Figure (c) shows the mean correlation curve between time τ and the distance $\langle D \rangle$. The black traced line represents the exponent equal to 1.0. Figure (d) shows the distribution of SWDP sizes in number of nodes per frequency of occurrence.

Paris, France

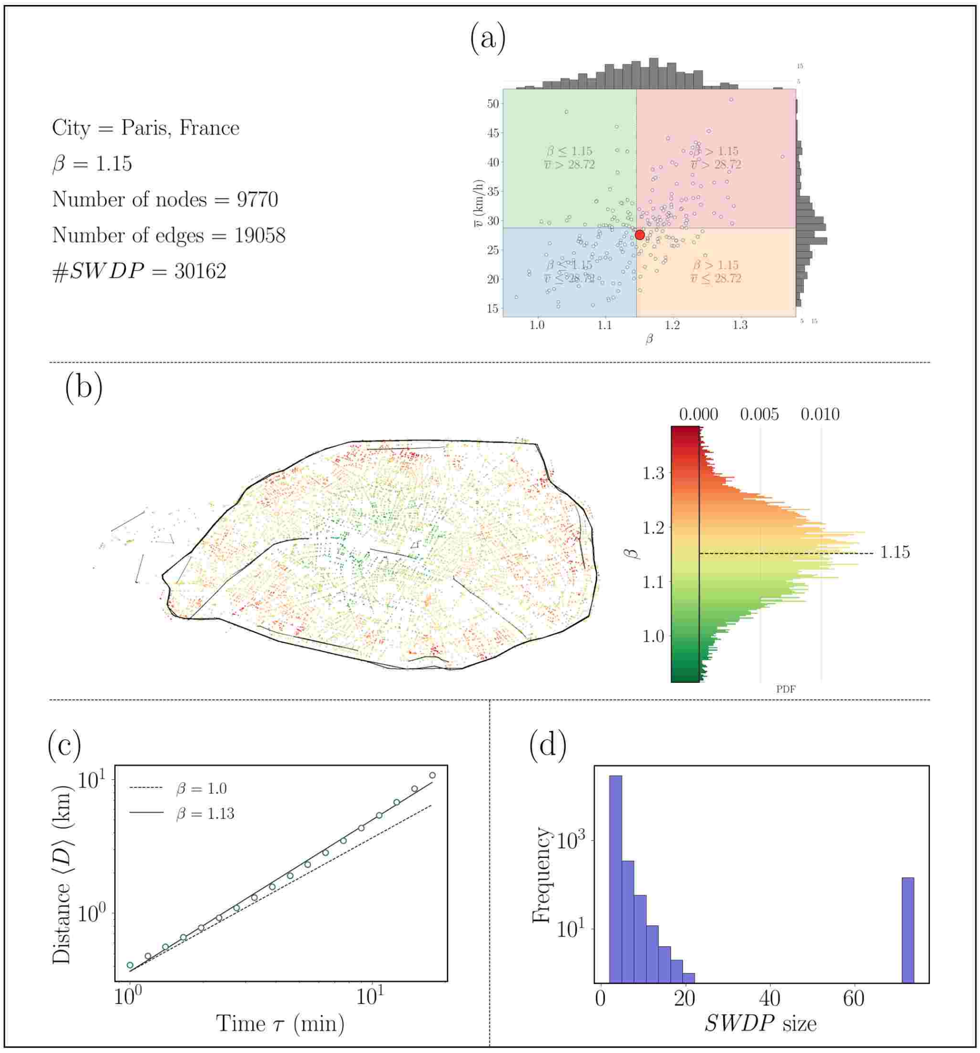

Fig. S136. Methodological sheet - Paris, France. In (a) Each point represents a city, with mean exponent (β), on the x-axis, and mean speed $\overline{v}$ obtained in all trips made to calculate the exponent on the axis y. The histograms of the values of β and $\overline{v}$ are shown on the axes in the upper and right corners, respectively. The graph was segmented into four quadrants, in which the division is performed by the mean values of β and $\overline{v}$. The quadrants were colored and annotated according to the division criteria. The red dot represents the location of Paris, France. In (b) taking all the nodes of Paris, France as origin, the dots are colored as a function of their exponent value and their color is quantified by the color bar in the center. The longest segments without a deceleration point (SWDP) are plotted in black. The probability density function of the $\beta's$ for each experiment is shown on the left of the color scale Figure (c) shows the mean correlation curve between time τ and the distance $\langle D \rangle$. The black traced line represents the exponent equal to 1.0. Figure (d) shows the distribution of SWDP sizes in number of nodes per frequency of occurrence.

Philadelphia, USA

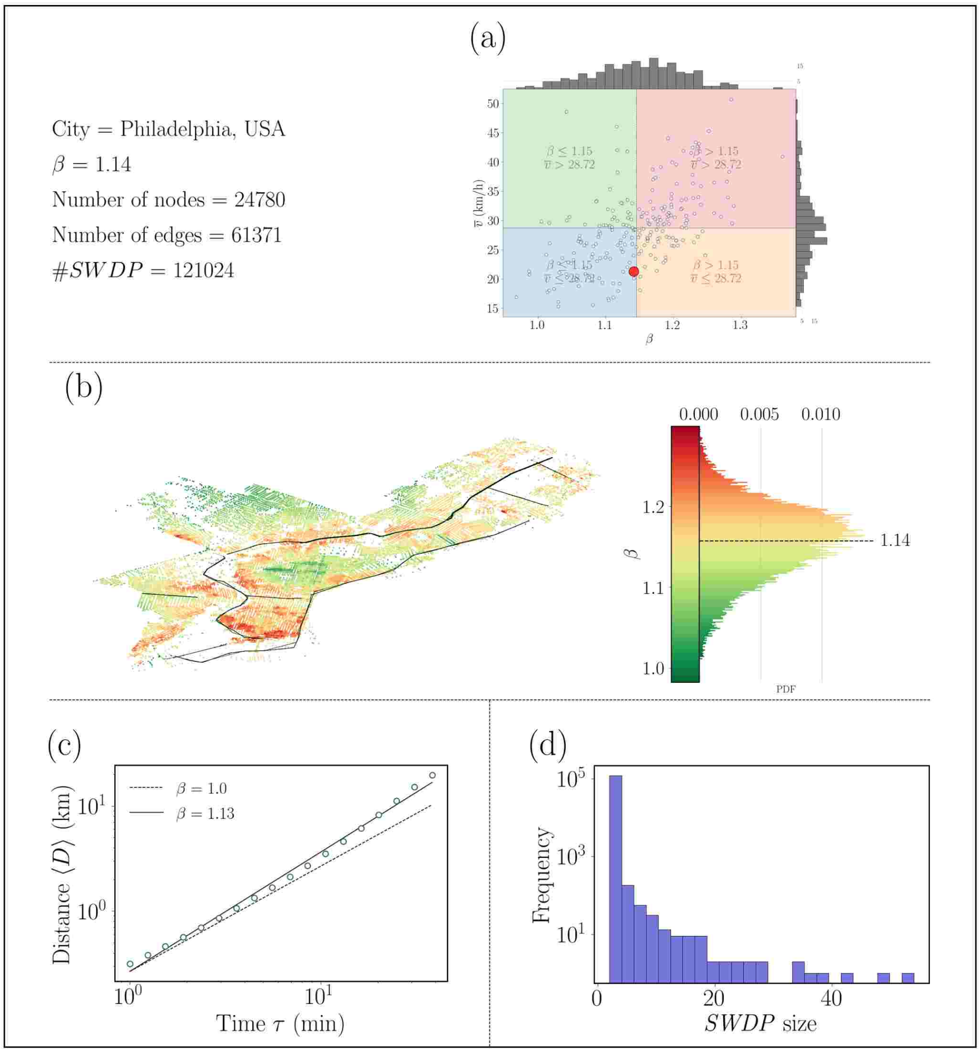

Fig. S137. Methodological sheet - Philadelphia, USA. In (a) Each point represents a city, with mean exponent (β), on the x-axis, and mean speed $\overline{v}$ obtained in all trips made to calculate the exponent on the axis y. The histograms of the values of β and $\overline{v}$ are shown on the axes in the upper and right corners, respectively. The graph was segmented into four quadrants, in which the division is performed by the mean values of β and $\overline{v}$. The quadrants were colored and annotated according to the division criteria. The red dot represents the location of Philadelphia, USA. In (b) taking all the nodes of Philadelphia, USA as origin, the dots are colored as a function of their exponent value and their color is quantified by the color bar in the center. The longest segments without a deceleration point (SWDP) are plotted in black. The probability density function of the $\beta's$ for each experiment is shown on the left of the color scale Figure (c) shows the mean correlation curve between time τ and the distance $\langle D \rangle$. The black traced line represents the exponent equal to 1.0. Figure (d) shows the distribution of SWDP sizes in number of nodes per frequency of occurrence.

Phoenix, USA

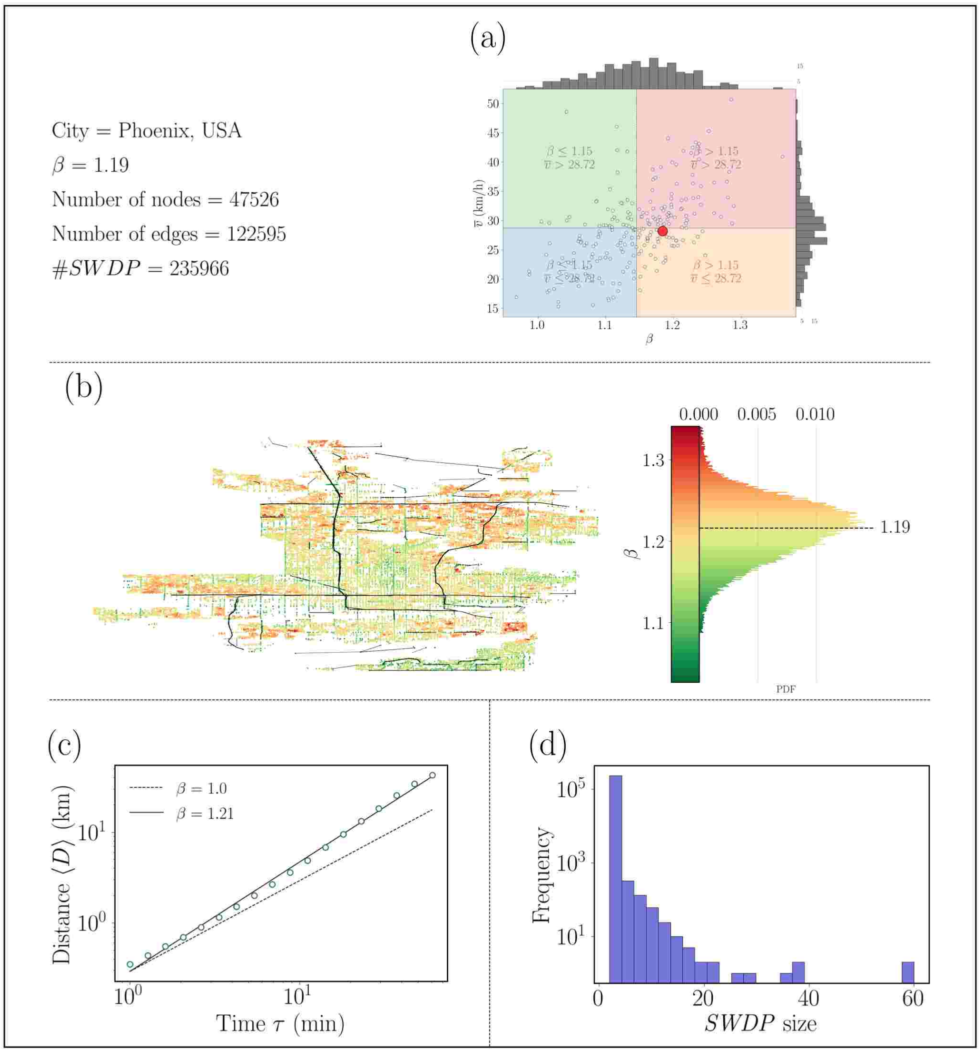

Fig. S138. Methodological sheet - Phoenix, USA. In (a) Each point represents a city, with mean exponent (β), on the x-axis, and mean speed $\overline{v}$ obtained in all trips made to calculate the exponent on the axis y. The histograms of the values of β and $\overline{v}$ are shown on the axes in the upper and right corners, respectively. The graph was segmented into four quadrants, in which the division is performed by the mean values of β and $\overline{v}$. The quadrants were colored and annotated according to the division criteria. The red dot represents the location of Phoenix, USA. In (b) taking all the nodes of Phoenix, USA as origin, the dots are colored as a function of their exponent value and their color is quantified by the color bar in the center. The longest segments without a deceleration point (SWDP) are plotted in black. The probability density function of the $\beta's$ for each experiment is shown on the left of the color scale Figure (c) shows the mean correlation curve between time τ and the distance $\langle D \rangle$. The black traced line represents the exponent equal to 1.0. Figure (d) shows the distribution of SWDP sizes in number of nodes per frequency of occurrence.

Pittsburgh, Pennsylvania, USA

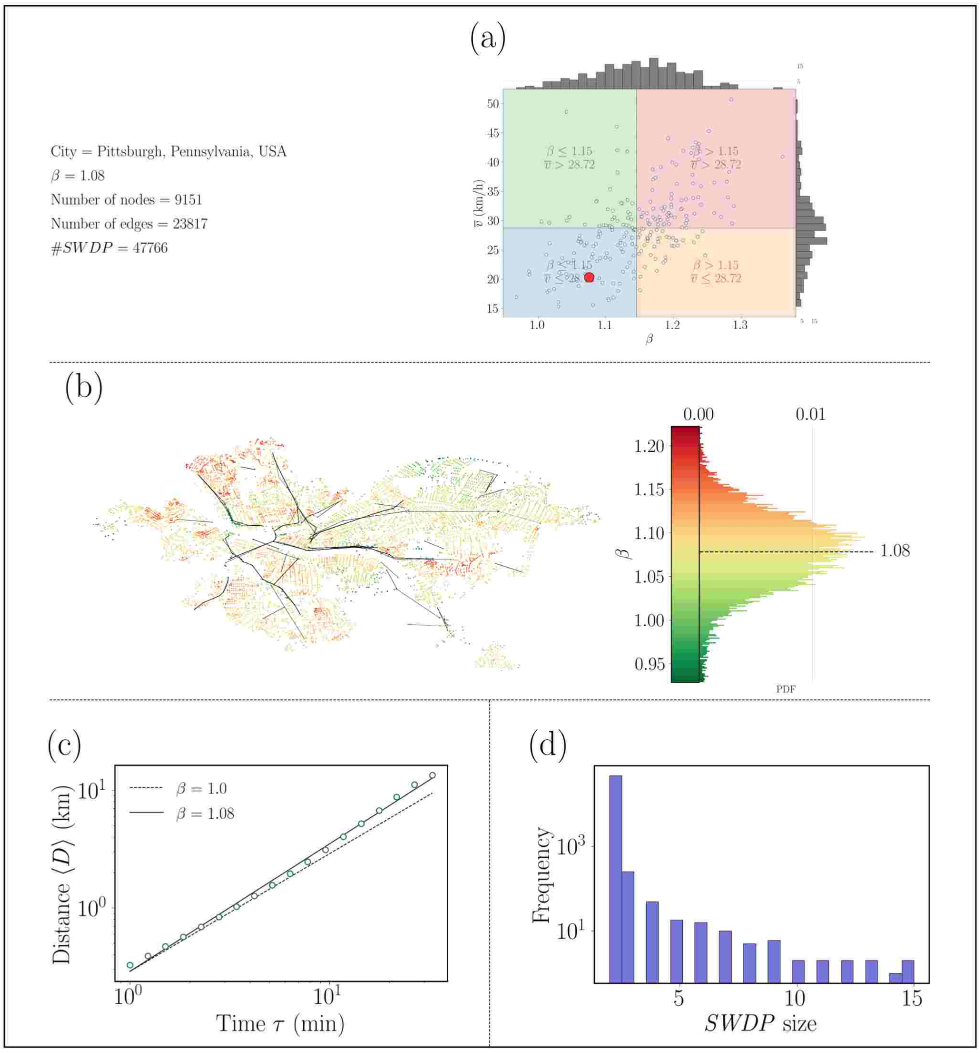

Fig. S139. Methodological sheet - Pittsburgh, Pennsylvania, USA. In (a) Each point represents a city, with mean exponent (β), on the x-axis, and mean speed $\overline{v}$ obtained in all trips made to calculate the exponent on the axis y. The histograms of the values of β and $\overline{v}$ are shown on the axes in the upper and right corners, respectively. The graph was segmented into four quadrants, in which the division is performed by the mean values of β and $\overline{v}$. The quadrants were colored and annotated according to the division criteria. The red dot represents the location of Pittsburgh, Pennsylvania, USA. In (b) taking all the nodes of Pittsburgh, Pennsylvania, USA as origin, the dots are colored as a function of their exponent value and their color is quantified by the color bar in the center. The longest segments without a deceleration point (SWDP) are plotted in black. The probability density function of the $\beta's$ for each experiment is shown on the left of the color scale Figure (c) shows the mean correlation curve between time τ and the distance $\langle D \rangle$. The black traced line represents the exponent equal to 1.0. Figure (d) shows the distribution of SWDP sizes in number of nodes per frequency of occurrence.

Playa del Carmen, Mexico

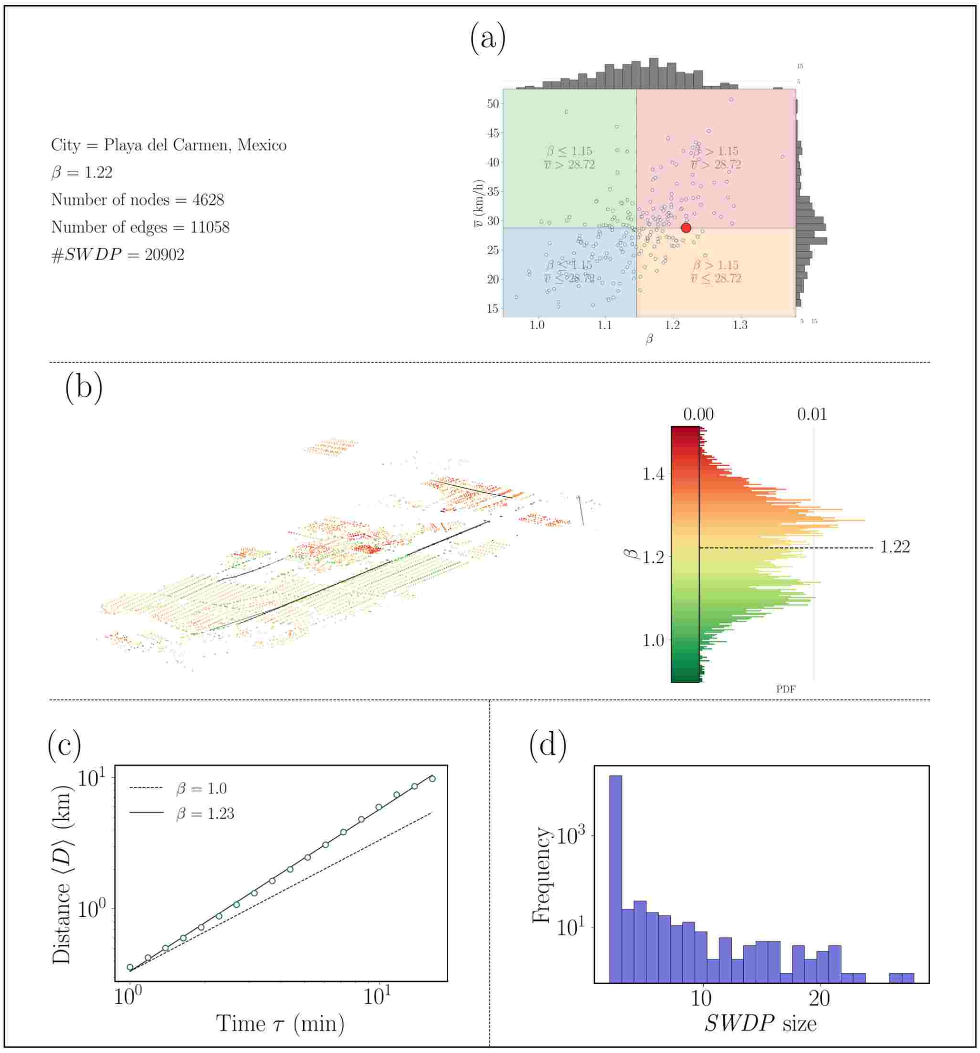

Fig. S140. Methodological sheet - Playa del Carmen, Mexico. In (a) Each point represents a city, with mean exponent (β), on the x-axis, and mean speed $\overline{v}$ obtained in all trips made to calculate the exponent on the axis y. The histograms of the values of β and $\overline{v}$ are shown on the axes in the upper and right corners, respectively. The graph was segmented into four quadrants, in which the division is performed by the mean values of β and $\overline{v}$. The quadrants were colored and annotated according to the division criteria. The red dot represents the location of Playa del Carmen, Mexico. In (b) taking all the nodes of Playa del Carmen, Mexico as origin, the dots are colored as a function of their exponent value and their color is quantified by the color bar in the center. The longest segments without a deceleration point (SWDP) are plotted in black. The probability density function of the $\beta's$ for each experiment is shown on the left of the color scale Figure (c) shows the mean correlation curve between time τ and the distance $\langle D \rangle$. The black traced line represents the exponent equal to 1.0. Figure (d) shows the distribution of SWDP sizes in number of nodes per frequency of occurrence.

Pori, Finland

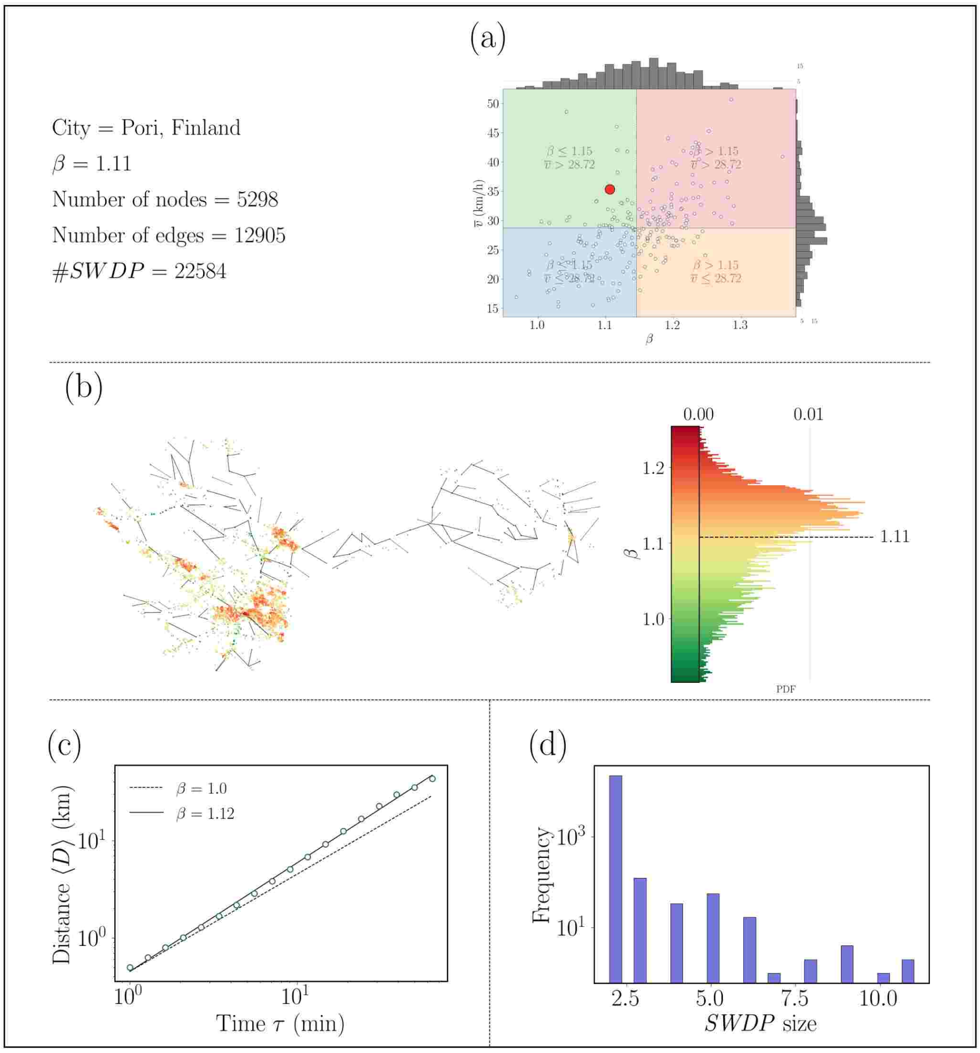

Fig. S141. Methodological sheet - Pori, Finland. In (a) Each point represents a city, with mean exponent (β), on the x-axis, and mean speed $\overline{v}$ obtained in all trips made to calculate the exponent on the axis y. The histograms of the values of β and $\overline{v}$ are shown on the axes in the upper and right corners, respectively. The graph was segmented into four quadrants, in which the division is performed by the mean values of β and $\overline{v}$. The quadrants were colored and annotated according to the division criteria. The red dot represents the location of Pori, Finland. In (b) taking all the nodes of Pori, Finland as origin, the dots are colored as a function of their exponent value and their color is quantified by the color bar in the center. The longest segments without a deceleration point (SWDP) are plotted in black. The probability density function of the $\beta's$ for each experiment is shown on the left of the color scale Figure (c) shows the mean correlation curve between time τ and the distance $\langle D \rangle$. The black traced line represents the exponent equal to 1.0. Figure (d) shows the distribution of SWDP sizes in number of nodes per frequency of occurrence.

Port Harcourt, Nigeria

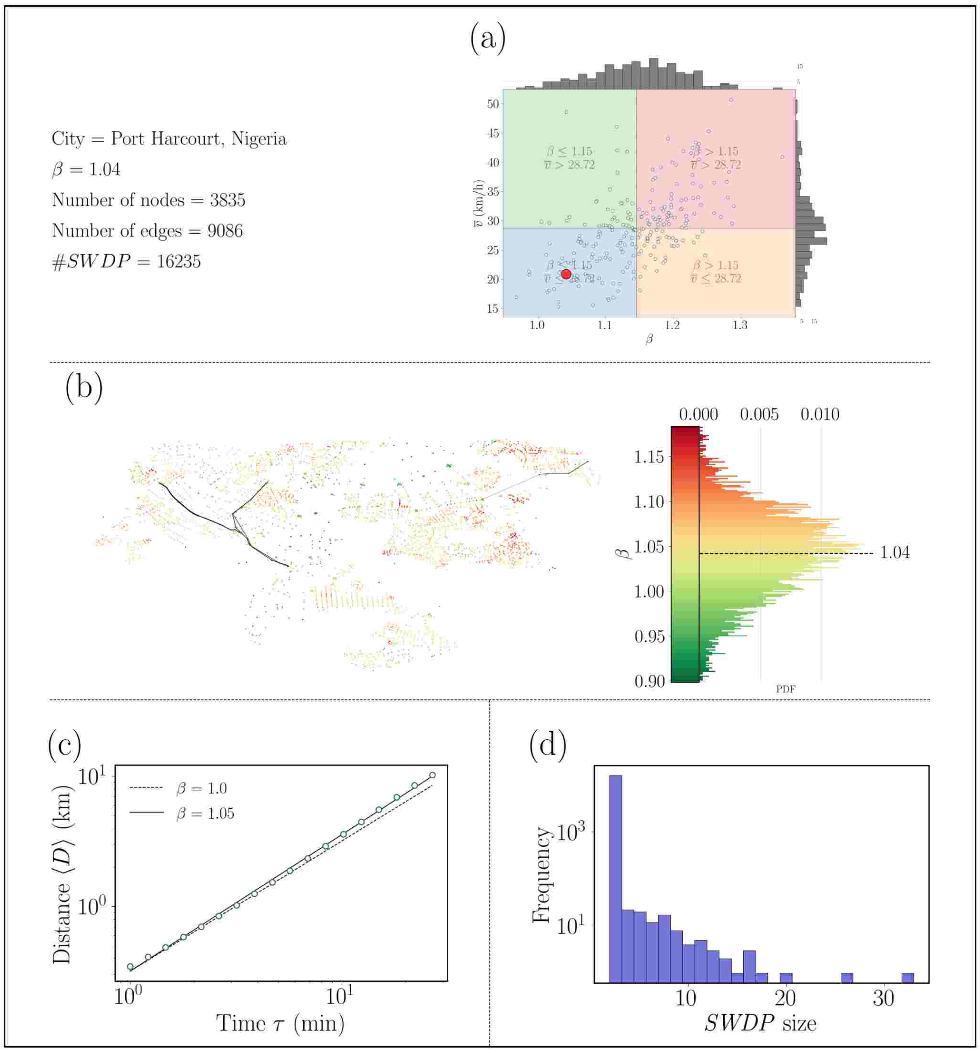

Fig. S142. Methodological sheet - Port Harcourt, Nigeria. In (a) Each point represents a city, with mean exponent (β), on the x-axis, and mean speed $\overline{v}$ obtained in all trips made to calculate the exponent on the axis y. The histograms of the values of β and $\overline{v}$ are shown on the axes in the upper and right corners, respectively. The graph was segmented into four quadrants, in which the division is performed by the mean values of β and $\overline{v}$. The quadrants were colored and annotated according to the division criteria. The red dot represents the location of Port Harcourt, Nigeria. In (b) taking all the nodes of Port Harcourt, Nigeria as origin, the dots are colored as a function of their exponent value and their color is quantified by the color bar in the center. The longest segments without a deceleration point (SWDP) are plotted in black. The probability density function of the $\beta's$ for each experiment is shown on the left of the color scale Figure (c) shows the mean correlation curve between time τ and the distance $\langle D \rangle$. The black traced line represents the exponent equal to 1.0. Figure (d) shows the distribution of SWDP sizes in number of nodes per frequency of occurrence.

Portland, USA

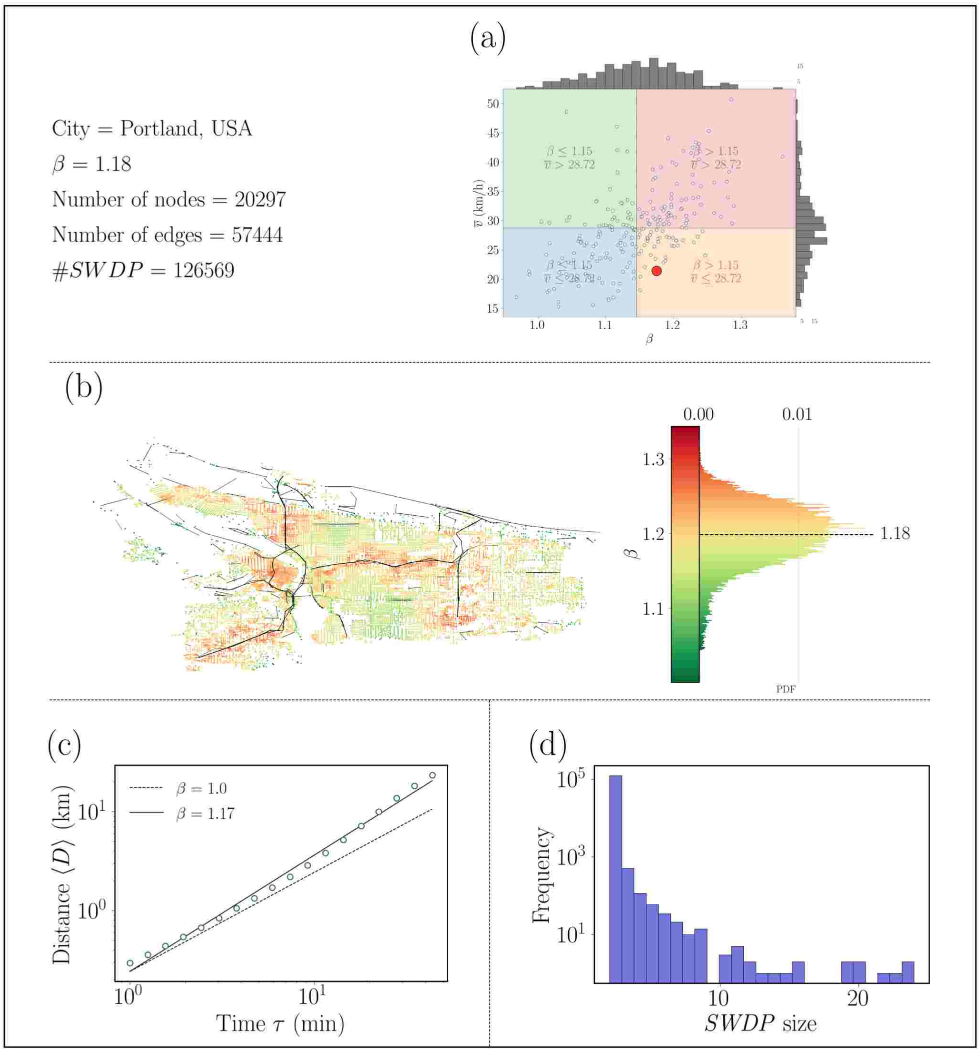

Fig. S143. Methodological sheet - Portland, USA. In (a) Each point represents a city, with mean exponent (β), on the x-axis, and mean speed $\overline{v}$ obtained in all trips made to calculate the exponent on the axis y. The histograms of the values of β and $\overline{v}$ are shown on the axes in the upper and right corners, respectively. The graph was segmented into four quadrants, in which the division is performed by the mean values of β and $\overline{v}$. The quadrants were colored and annotated according to the division criteria. The red dot represents the location of Portland, USA. In (b) taking all the nodes of Portland, USA as origin, the dots are colored as a function of their exponent value and their color is quantified by the color bar in the center. The longest segments without a deceleration point (SWDP) are plotted in black. The probability density function of the $\beta's$ for each experiment is shown on the left of the color scale Figure (c) shows the mean correlation curve between time τ and the distance $\langle D \rangle$. The black traced line represents the exponent equal to 1.0. Figure (d) shows the distribution of SWDP sizes in number of nodes per frequency of occurrence.

Porto Alegre, Brasil

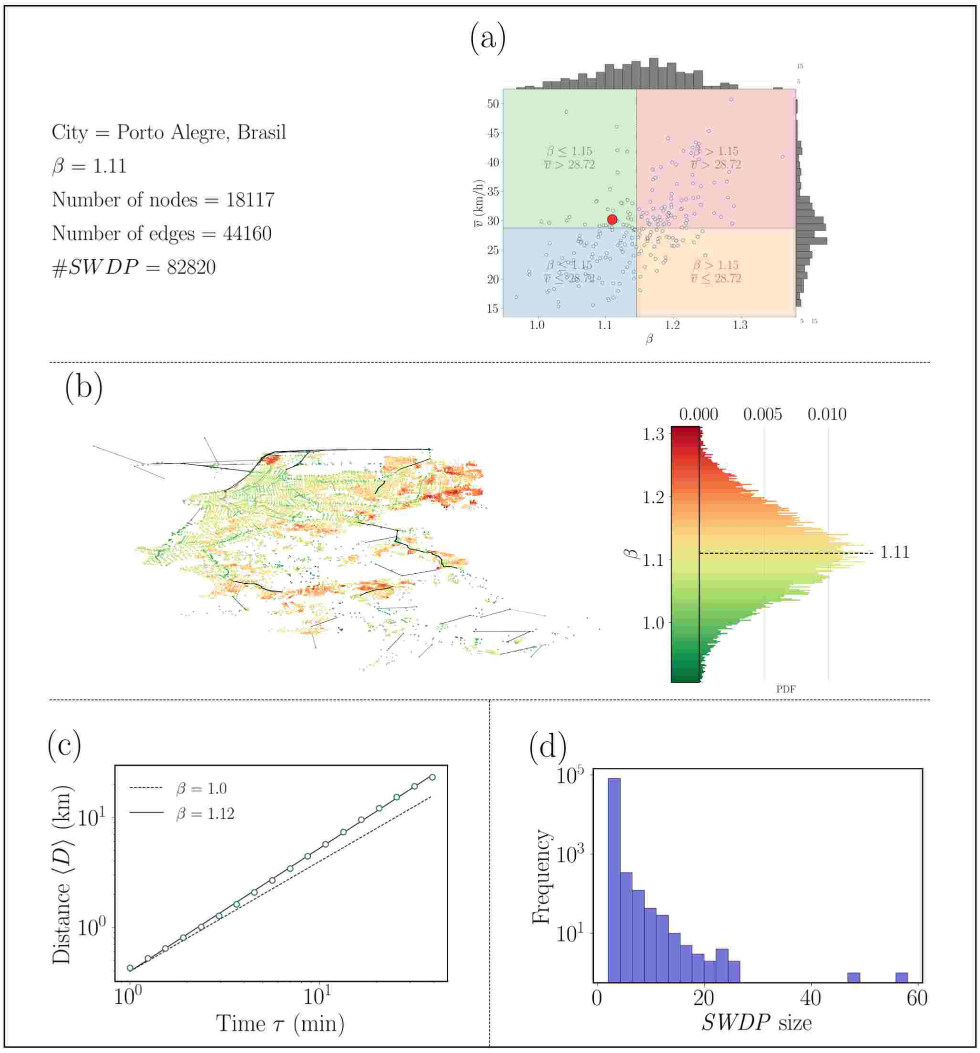

Fig. S144. Methodological sheet - Porto Alegre, Brasil. In (a) Each point represents a city, with mean exponent (β), on the x-axis, and mean speed $\overline{v}$ obtained in all trips made to calculate the exponent on the axis y. The histograms of the values of β and $\overline{v}$ are shown on the axes in the upper and right corners, respectively. The graph was segmented into four quadrants, in which the division is performed by the mean values of β and $\overline{v}$. The quadrants were colored and annotated according to the division criteria. The red dot represents the location of Porto Alegre, Brasil. In (b) taking all the nodes of Porto Alegre, Brasil as origin, the dots are colored as a function of their exponent value and their color is quantified by the color bar in the center. The longest segments without a deceleration point (SWDP) are plotted in black. The probability density function of the $\beta's$ for each experiment is shown on the left of the color scale Figure (c) shows the mean correlation curve between time τ and the distance $\langle D \rangle$. The black traced line represents the exponent equal to 1.0. Figure (d) shows the distribution of SWDP sizes in number of nodes per frequency of occurrence.

Porto Velho, Brasil

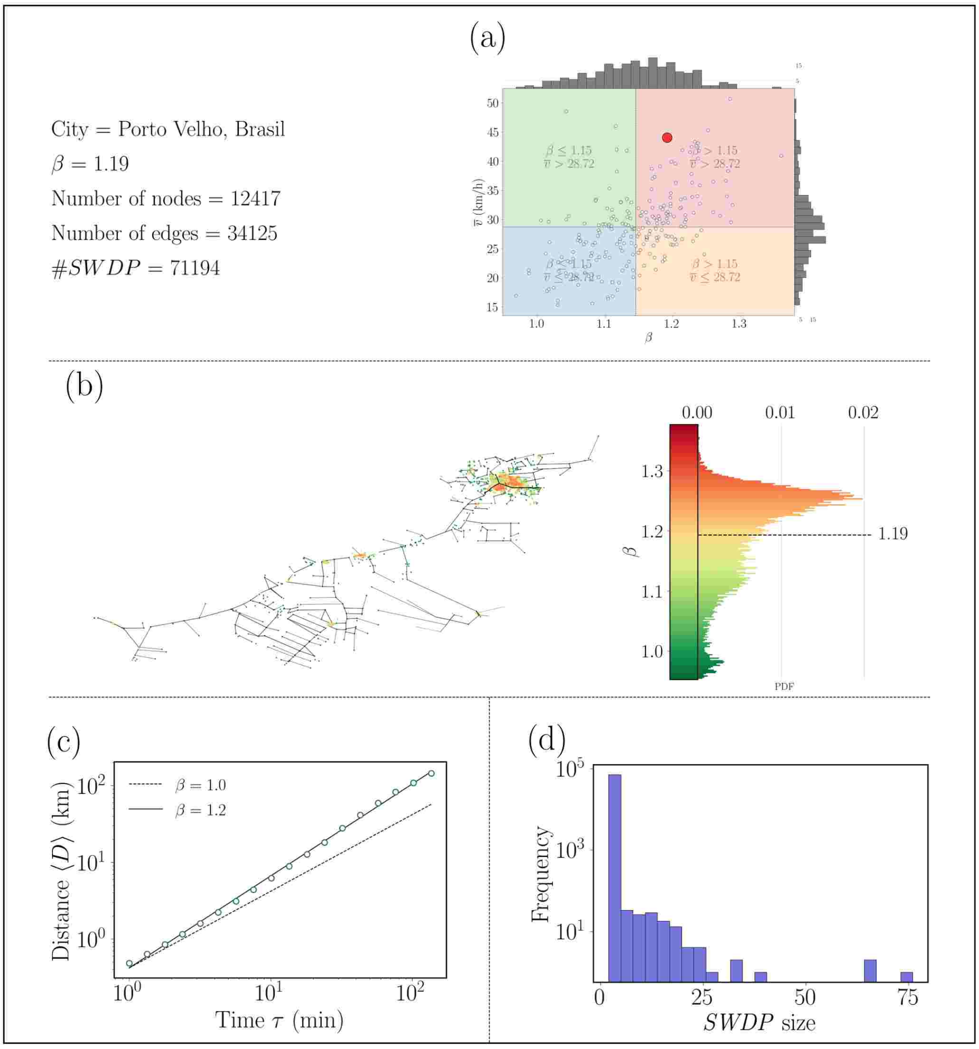

Fig. S145. Methodological sheet - Porto Velho, Brasil. In (a) Each point represents a city, with mean exponent (β), on the x-axis, and mean speed $\overline{v}$ obtained in all trips made to calculate the exponent on the axis y. The histograms of the values of β and $\overline{v}$ are shown on the axes in the upper and right corners, respectively. The graph was segmented into four quadrants, in which the division is performed by the mean values of β and $\overline{v}$. The quadrants were colored and annotated according to the division criteria. The red dot represents the location of Porto Velho, Brasil. In (b) taking all the nodes of Porto Velho, Brasil as origin, the dots are colored as a function of their exponent value and their color is quantified by the color bar in the center. The longest segments without a deceleration point (SWDP) are plotted in black. The probability density function of the $\beta's$ for each experiment is shown on the left of the color scale Figure (c) shows the mean correlation curve between time τ and the distance $\langle D \rangle$. The black traced line represents the exponent equal to 1.0. Figure (d) shows the distribution of SWDP sizes in number of nodes per frequency of occurrence.

Porto, Portugal

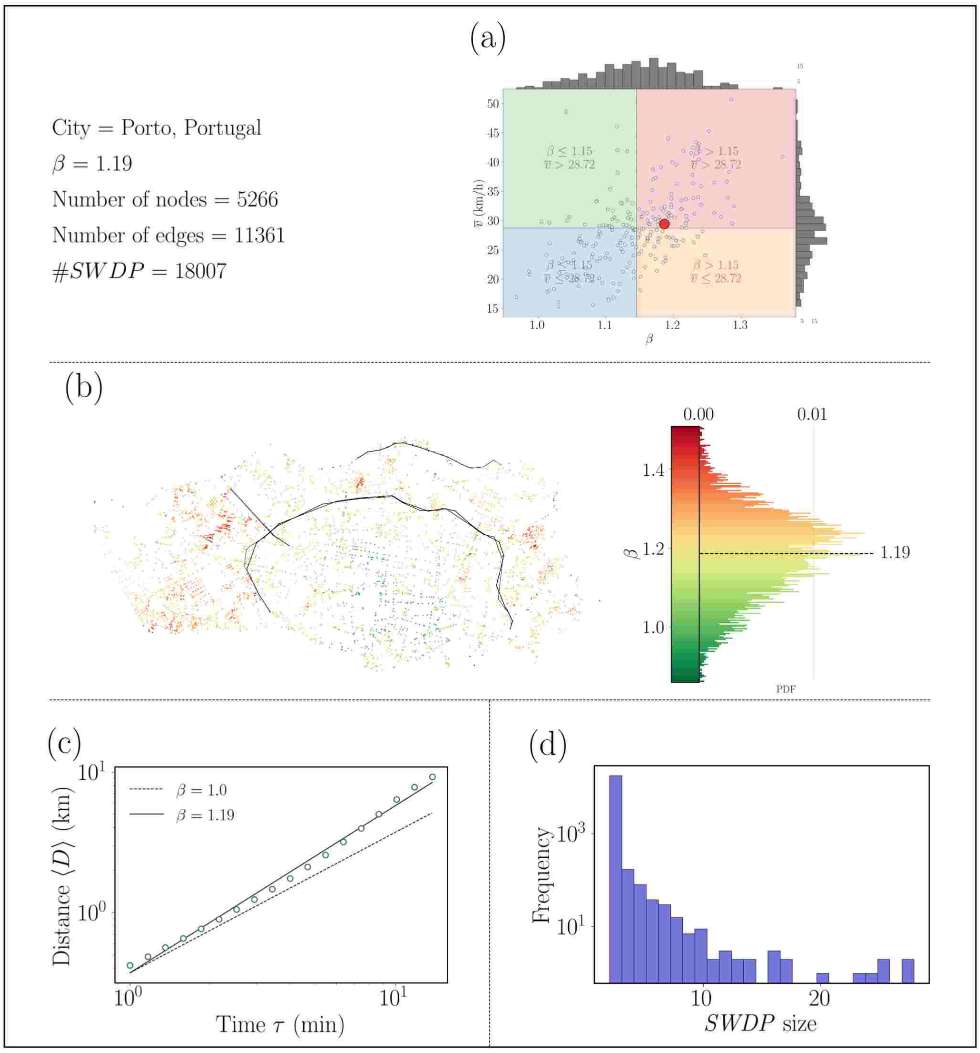

Fig. S146. Methodological sheet - Porto, Portugal. In (a) Each point represents a city, with mean exponent (β), on the x-axis, and mean speed $\overline{v}$ obtained in all trips made to calculate the exponent on the axis y. The histograms of the values of β and $\overline{v}$ are shown on the axes in the upper and right corners, respectively. The graph was segmented into four quadrants, in which the division is performed by the mean values of β and $\overline{v}$. The quadrants were colored and annotated according to the division criteria. The red dot represents the location of Porto, Portugal. In (b) taking all the nodes of Porto, Portugal as origin, the dots are colored as a function of their exponent value and their color is quantified by the color bar in the center. The longest segments without a deceleration point (SWDP) are plotted in black. The probability density function of the $\beta's$ for each experiment is shown on the left of the color scale Figure (c) shows the mean correlation curve between time τ and the distance $\langle D \rangle$. The black traced line represents the exponent equal to 1.0. Figure (d) shows the distribution of SWDP sizes in number of nodes per frequency of occurrence.

Prague, Czechia

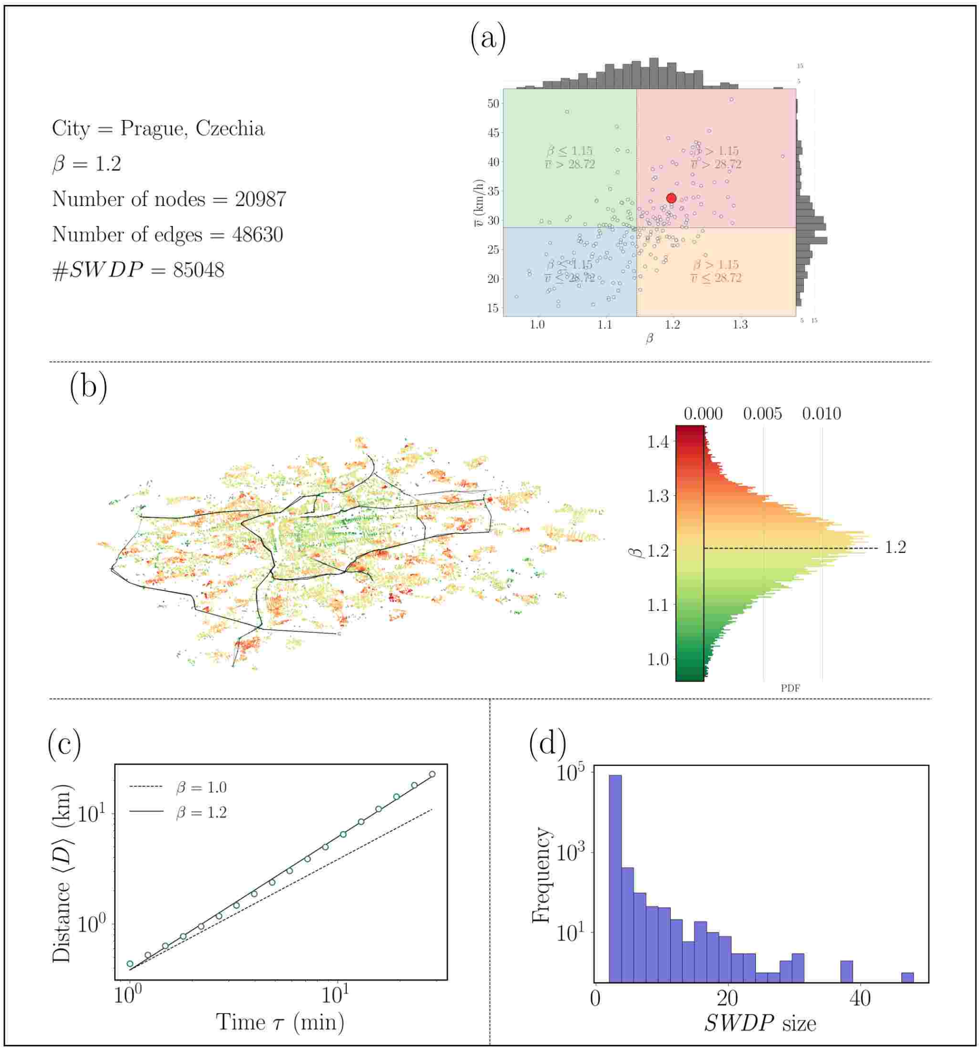

Fig. S147. Methodological sheet - Prague, Czechia. In (a) Each point represents a city, with mean exponent (β), on the x-axis, and mean speed $\overline{v}$ obtained in all trips made to calculate the exponent on the axis y. The histograms of the values of β and $\overline{v}$ are shown on the axes in the upper and right corners, respectively. The graph was segmented into four quadrants, in which the division is performed by the mean values of β and $\overline{v}$. The quadrants were colored and annotated according to the division criteria. The red dot represents the location of Prague, Czechia. In (b) taking all the nodes of Prague, Czechia as origin, the dots are colored as a function of their exponent value and their color is quantified by the color bar in the center. The longest segments without a deceleration point (SWDP) are plotted in black. The probability density function of the $\beta's$ for each experiment is shown on the left of the color scale Figure (c) shows the mean correlation curve between time τ and the distance $\langle D \rangle$. The black traced line represents the exponent equal to 1.0. Figure (d) shows the distribution of SWDP sizes in number of nodes per frequency of occurrence.

Pyongyang, North Korea

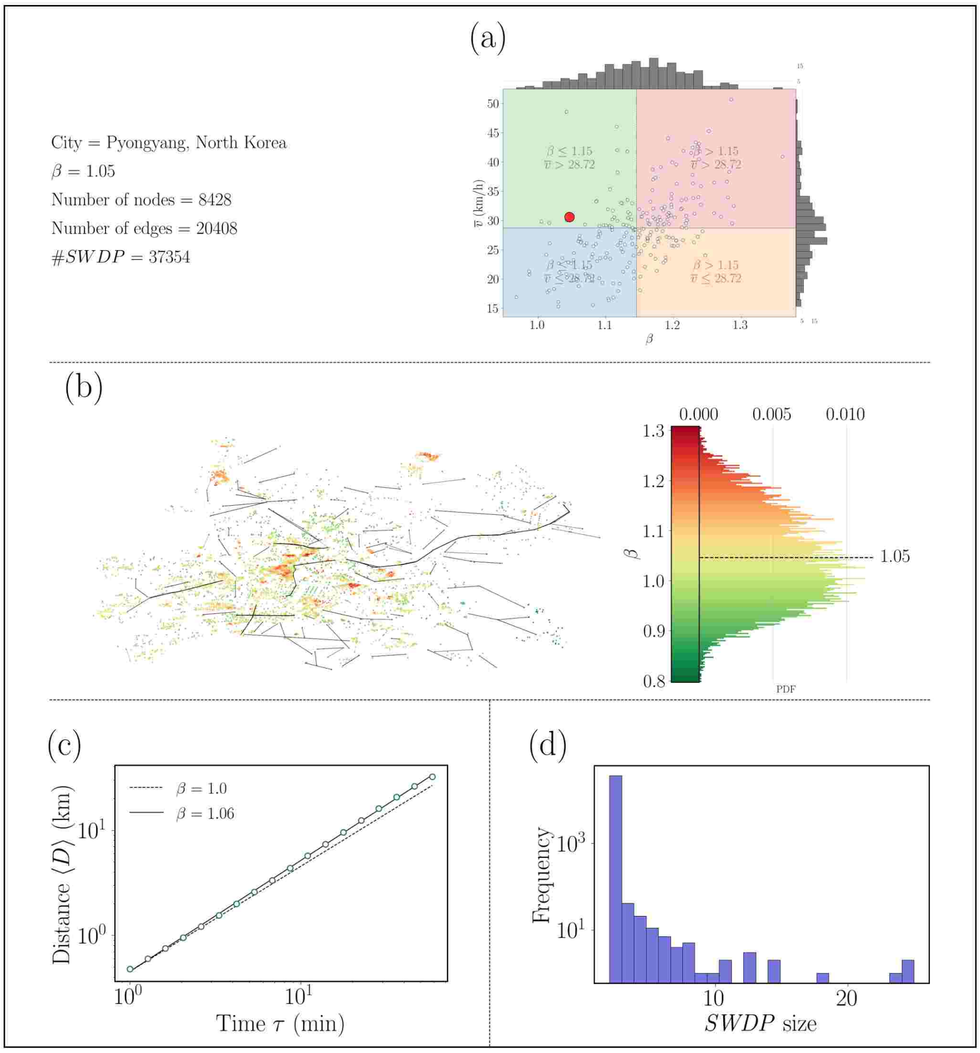

Fig. S148. Methodological sheet - Pyongyang, North Korea. In (a) Each point represents a city, with mean exponent (β), on the x-axis, and mean speed $\overline{v}$ obtained in all trips made to calculate the exponent on the axis y. The histograms of the values of β and $\overline{v}$ are shown on the axes in the upper and right corners, respectively. The graph was segmented into four quadrants, in which the division is performed by the mean values of β and $\overline{v}$. The quadrants were colored and annotated according to the division criteria. The red dot represents the location of Pyongyang, North Korea. In (b) taking all the nodes of Pyongyang, North Korea as origin, the dots are colored as a function of their exponent value and their color is quantified by the color bar in the center. The longest segments without a deceleration point (SWDP) are plotted in black. The probability density function of the $\beta's$ for each experiment is shown on the left of the color scale Figure (c) shows the mean correlation curve between time τ and the distance $\langle D \rangle$. The black traced line represents the exponent equal to 1.0. Figure (d) shows the distribution of SWDP sizes in number of nodes per frequency of occurrence.

Quebec City, Canadá

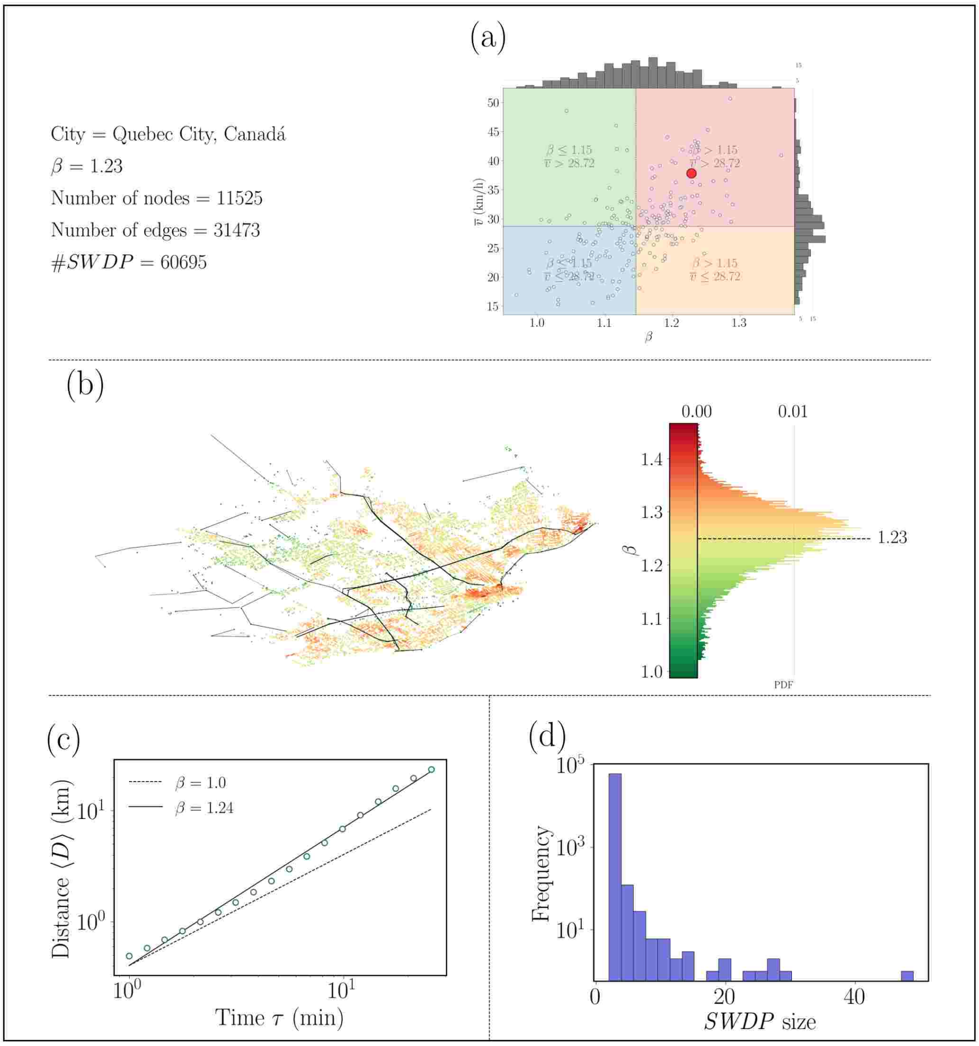

Fig. S149. Methodological sheet - Quebec City, Canadá. In (a) Each point represents a city, with mean exponent (β), on the x-axis, and mean speed $\overline{v}$ obtained in all trips made to calculate the exponent on the axis y. The histograms of the values of β and $\overline{v}$ are shown on the axes in the upper and right corners, respectively. The graph was segmented into four quadrants, in which the division is performed by the mean values of β and $\overline{v}$. The quadrants were colored and annotated according to the division criteria. The red dot represents the location of Quebec City, Canadá. In (b) taking all the nodes of Quebec City, Canadá as origin, the dots are colored as a function of their exponent value and their color is quantified by the color bar in the center. The longest segments without a deceleration point (SWDP) are plotted in black. The probability density function of the $\beta's$ for each experiment is shown on the left of the color scale Figure (c) shows the mean correlation curve between time τ and the distance $\langle D \rangle$. The black traced line represents the exponent equal to 1.0. Figure (d) shows the distribution of SWDP sizes in number of nodes per frequency of occurrence.

Rabat, Morocco

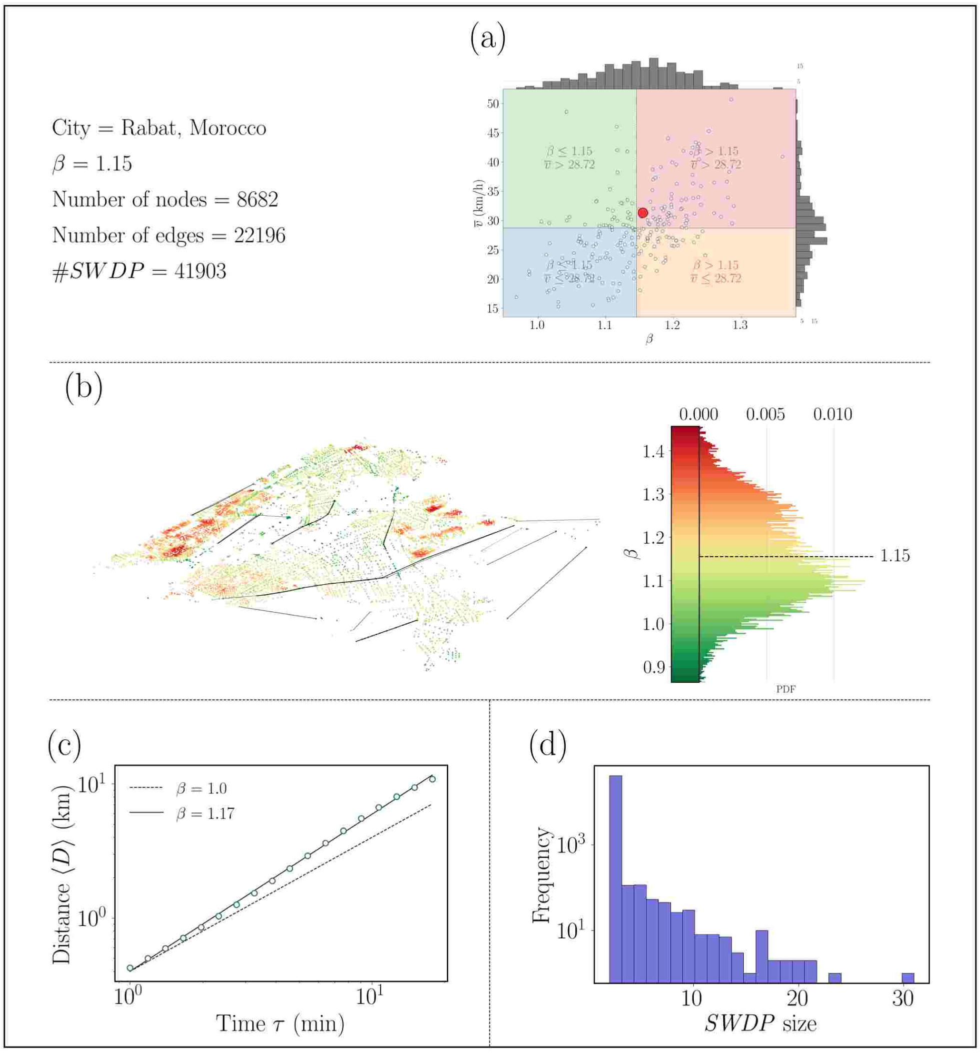

Fig. S150. Methodological sheet - Rabat, Morocco. In (a) Each point represents a city, with mean exponent (β), on the x-axis, and mean speed $\overline{v}$ obtained in all trips made to calculate the exponent on the axis y. The histograms of the values of β and $\overline{v}$ are shown on the axes in the upper and right corners, respectively. The graph was segmented into four quadrants, in which the division is performed by the mean values of β and $\overline{v}$. The quadrants were colored and annotated according to the division criteria. The red dot represents the location of Rabat, Morocco. In (b) taking all the nodes of Rabat, Morocco as origin, the dots are colored as a function of their exponent value and their color is quantified by the color bar in the center. The longest segments without a deceleration point (SWDP) are plotted in black. The probability density function of the $\beta's$ for each experiment is shown on the left of the color scale Figure (c) shows the mean correlation curve between time τ and the distance $\langle D \rangle$. The black traced line represents the exponent equal to 1.0. Figure (d) shows the distribution of SWDP sizes in number of nodes per frequency of occurrence.

Raleigh, USA

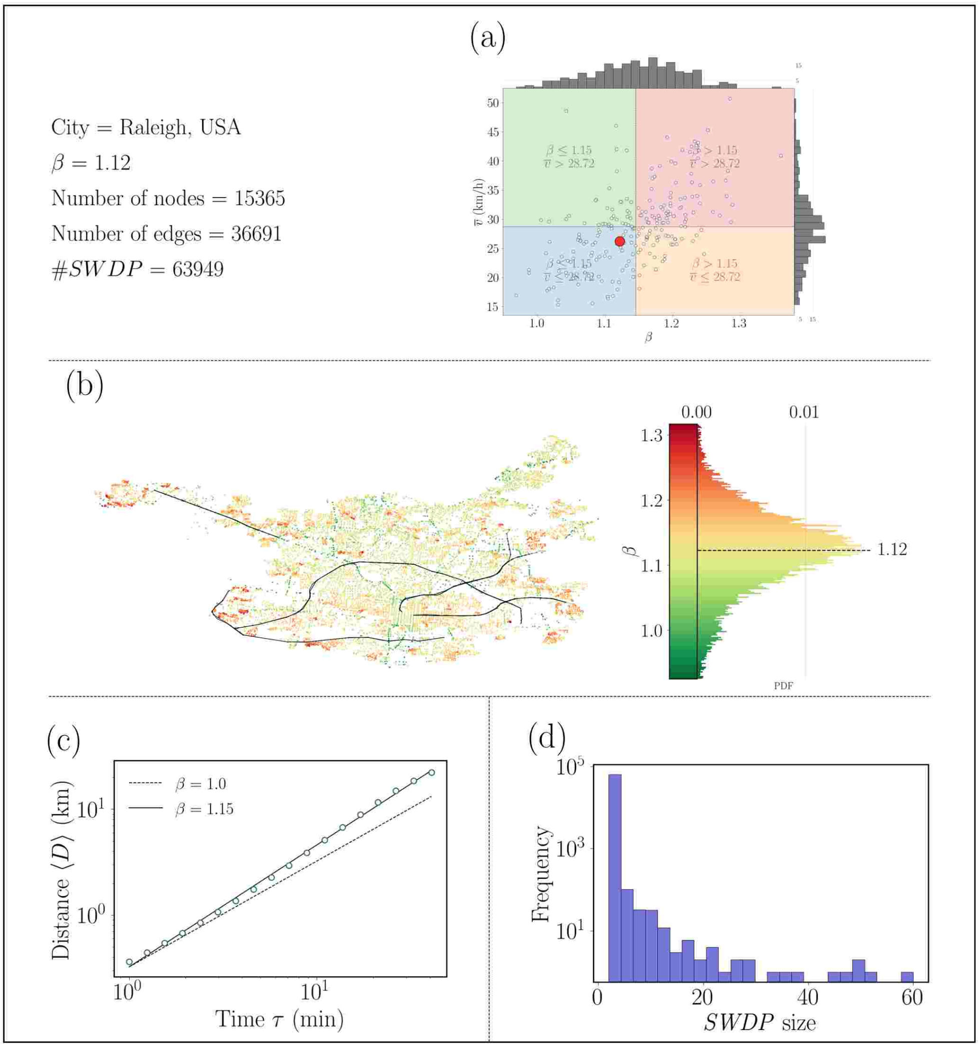

Fig. S151. Methodological sheet - Raleigh, USA. In (a) Each point represents a city, with mean exponent (β), on the x-axis, and mean speed $\overline{v}$ obtained in all trips made to calculate the exponent on the axis y. The histograms of the values of β and $\overline{v}$ are shown on the axes in the upper and right corners, respectively. The graph was segmented into four quadrants, in which the division is performed by the mean values of β and $\overline{v}$. The quadrants were colored and annotated according to the division criteria. The red dot represents the location of Raleigh, USA. In (b) taking all the nodes of Raleigh, USA as origin, the dots are colored as a function of their exponent value and their color is quantified by the color bar in the center. The longest segments without a deceleration point (SWDP) are plotted in black. The probability density function of the $\beta's$ for each experiment is shown on the left of the color scale Figure (c) shows the mean correlation curve between time τ and the distance $\langle D \rangle$. The black traced line represents the exponent equal to 1.0. Figure (d) shows the distribution of SWDP sizes in number of nodes per frequency of occurrence.

Reading, USA

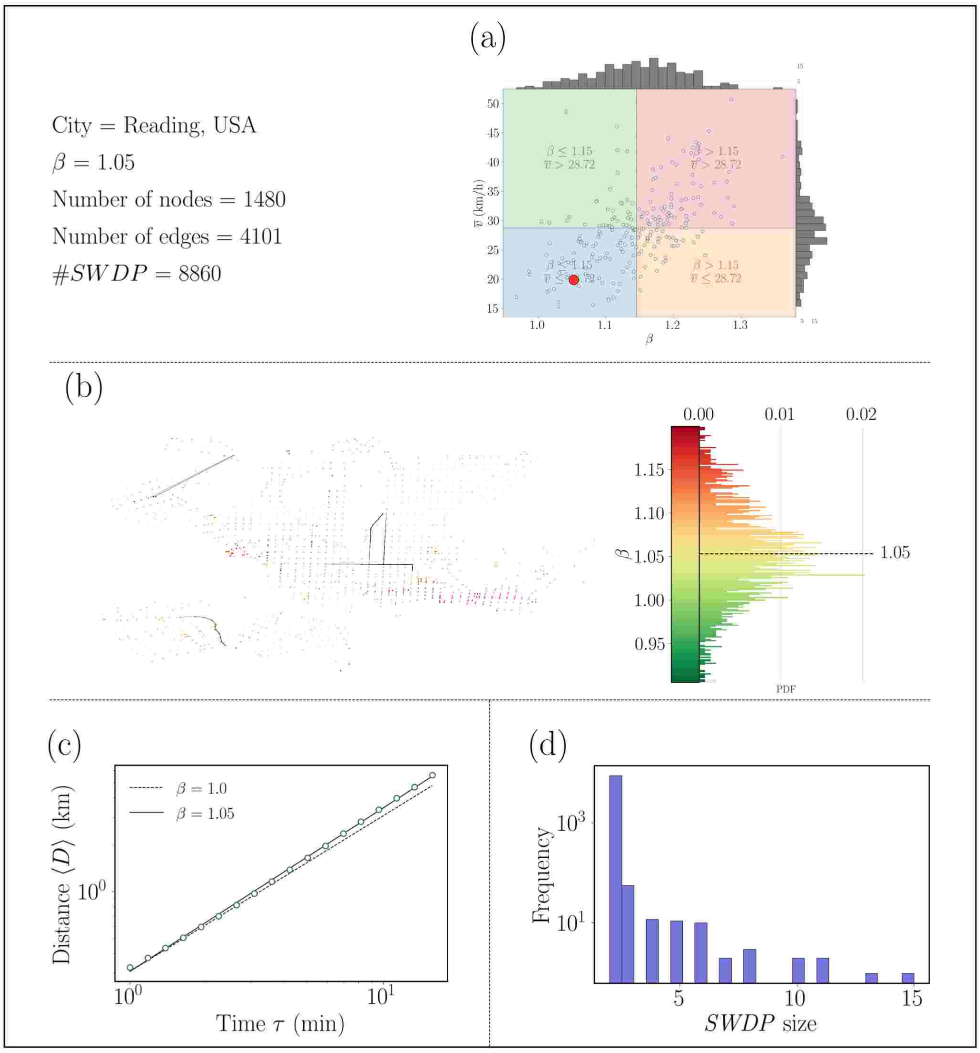

Fig. S152. Methodological sheet - Reading, USA. In (a) Each point represents a city, with mean exponent (β), on the x-axis, and mean speed $\overline{v}$ obtained in all trips made to calculate the exponent on the axis y. The histograms of the values of β and $\overline{v}$ are shown on the axes in the upper and right corners, respectively. The graph was segmented into four quadrants, in which the division is performed by the mean values of β and $\overline{v}$. The quadrants were colored and annotated according to the division criteria. The red dot represents the location of Reading, USA. In (b) taking all the nodes of Reading, USA as origin, the dots are colored as a function of their exponent value and their color is quantified by the color bar in the center. The longest segments without a deceleration point (SWDP) are plotted in black. The probability density function of the $\beta's$ for each experiment is shown on the left of the color scale Figure (c) shows the mean correlation curve between time τ and the distance $\langle D \rangle$. The black traced line represents the exponent equal to 1.0. Figure (d) shows the distribution of SWDP sizes in number of nodes per frequency of occurrence.

Recife, Brasil

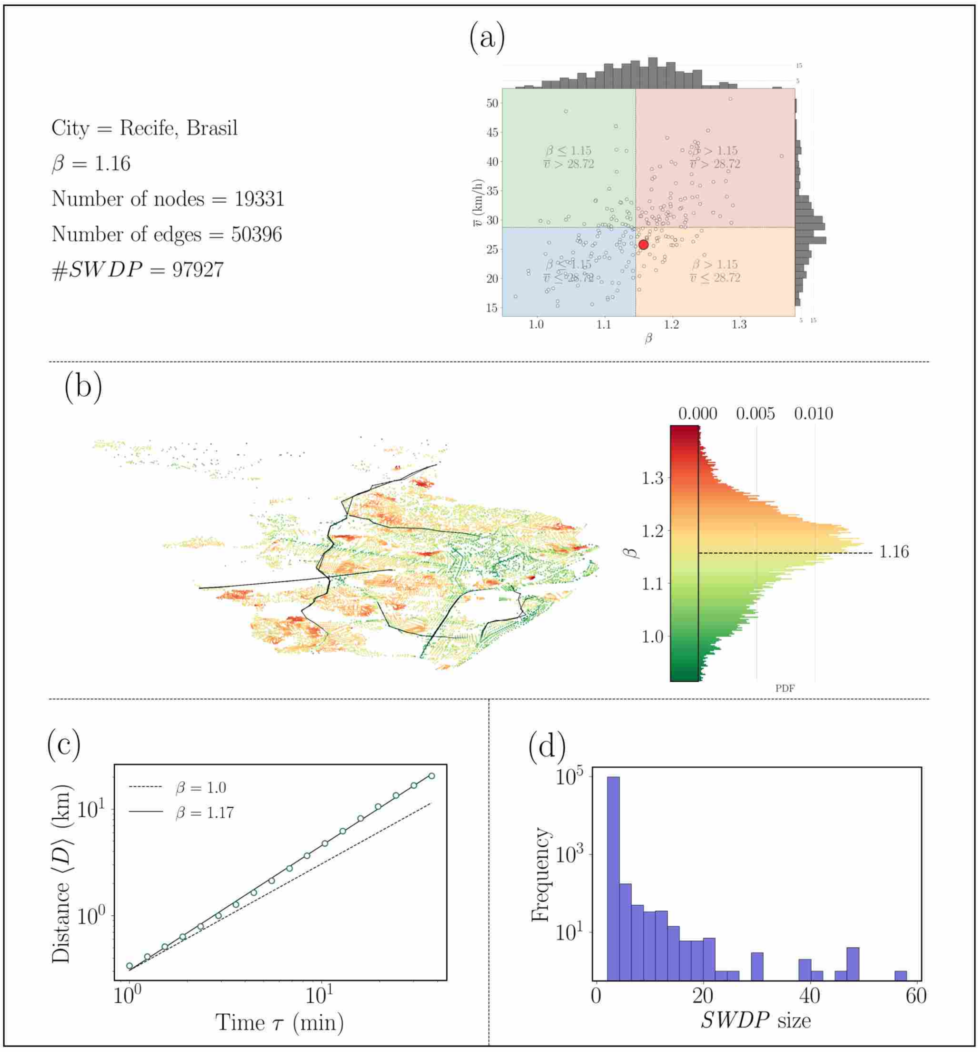

Fig. S153. Methodological sheet - Recife, Brasil. In (a) Each point represents a city, with mean exponent (β), on the x-axis, and mean speed $\overline{v}$ obtained in all trips made to calculate the exponent on the axis y. The histograms of the values of β and $\overline{v}$ are shown on the axes in the upper and right corners, respectively. The graph was segmented into four quadrants, in which the division is performed by the mean values of β and $\overline{v}$. The quadrants were colored and annotated according to the division criteria. The red dot represents the location of Recife, Brasil. In (b) taking all the nodes of Recife, Brasil as origin, the dots are colored as a function of their exponent value and their color is quantified by the color bar in the center. The longest segments without a deceleration point (SWDP) are plotted in black. The probability density function of the $\beta's$ for each experiment is shown on the left of the color scale Figure (c) shows the mean correlation curve between time τ and the distance $\langle D \rangle$. The black traced line represents the exponent equal to 1.0. Figure (d) shows the distribution of SWDP sizes in number of nodes per frequency of occurrence.

Reykjavik, Iceland

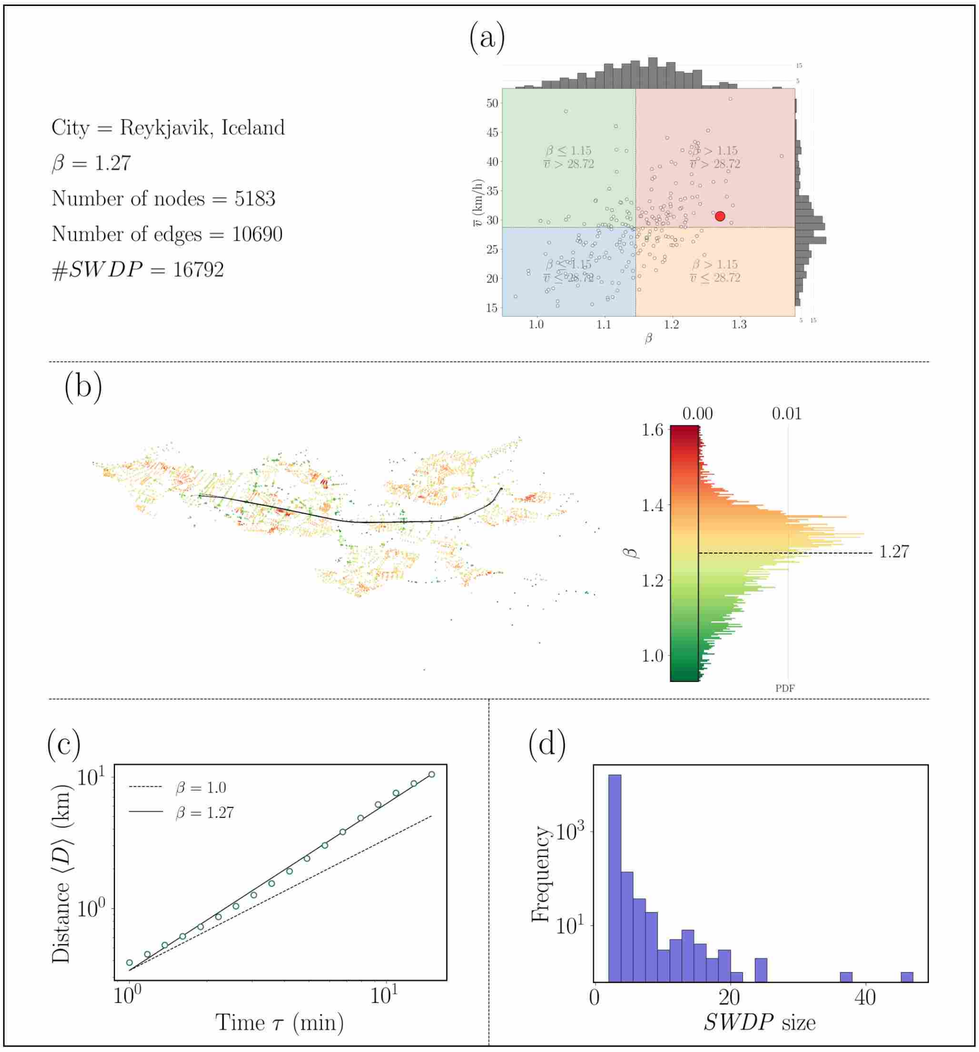

Fig. S154. Methodological sheet - Reykjavik, Iceland. In (a) Each point represents a city, with mean exponent (β), on the x-axis, and mean speed $\overline{v}$ obtained in all trips made to calculate the exponent on the axis y. The histograms of the values of β and $\overline{v}$ are shown on the axes in the upper and right corners, respectively. The graph was segmented into four quadrants, in which the division is performed by the mean values of β and $\overline{v}$. The quadrants were colored and annotated according to the division criteria. The red dot represents the location of Reykjavik, Iceland. In (b) taking all the nodes of Reykjavik, Iceland as origin, the dots are colored as a function of their exponent value and their color is quantified by the color bar in the center. The longest segments without a deceleration point (SWDP) are plotted in black. The probability density function of the $\beta's$ for each experiment is shown on the left of the color scale Figure (c) shows the mean correlation curve between time τ and the distance $\langle D \rangle$. The black traced line represents the exponent equal to 1.0. Figure (d) shows the distribution of SWDP sizes in number of nodes per frequency of occurrence.

Richmond, USA

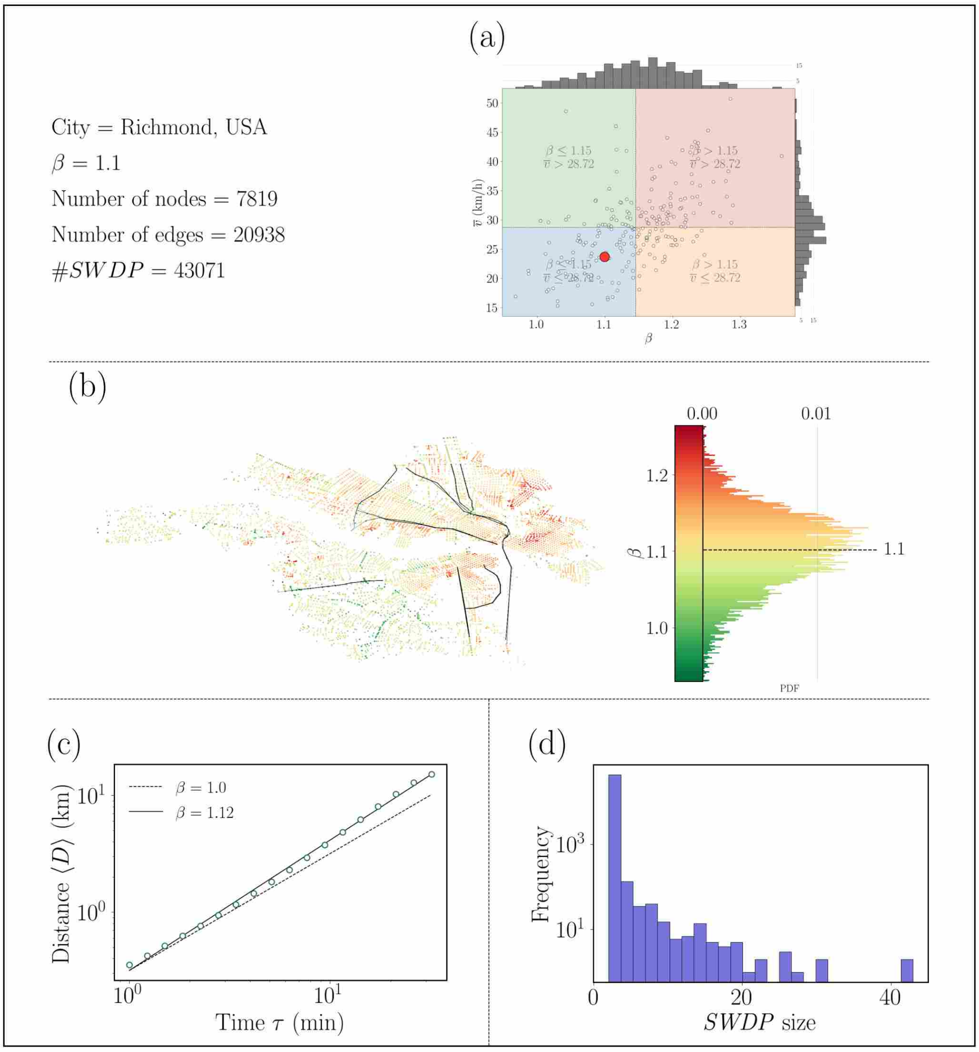

Fig. S155. Methodological sheet - Richmond, USA. In (a) Each point represents a city, with mean exponent (β), on the x-axis, and mean speed $\overline{v}$ obtained in all trips made to calculate the exponent on the axis y. The histograms of the values of β and $\overline{v}$ are shown on the axes in the upper and right corners, respectively. The graph was segmented into four quadrants, in which the division is performed by the mean values of β and $\overline{v}$. The quadrants were colored and annotated according to the division criteria. The red dot represents the location of Richmond, USA. In (b) taking all the nodes of Richmond, USA as origin, the dots are colored as a function of their exponent value and their color is quantified by the color bar in the center. The longest segments without a deceleration point (SWDP) are plotted in black. The probability density function of the $\beta's$ for each experiment is shown on the left of the color scale Figure (c) shows the mean correlation curve between time τ and the distance $\langle D \rangle$. The black traced line represents the exponent equal to 1.0. Figure (d) shows the distribution of SWDP sizes in number of nodes per frequency of occurrence.

Rijeka, Croatia

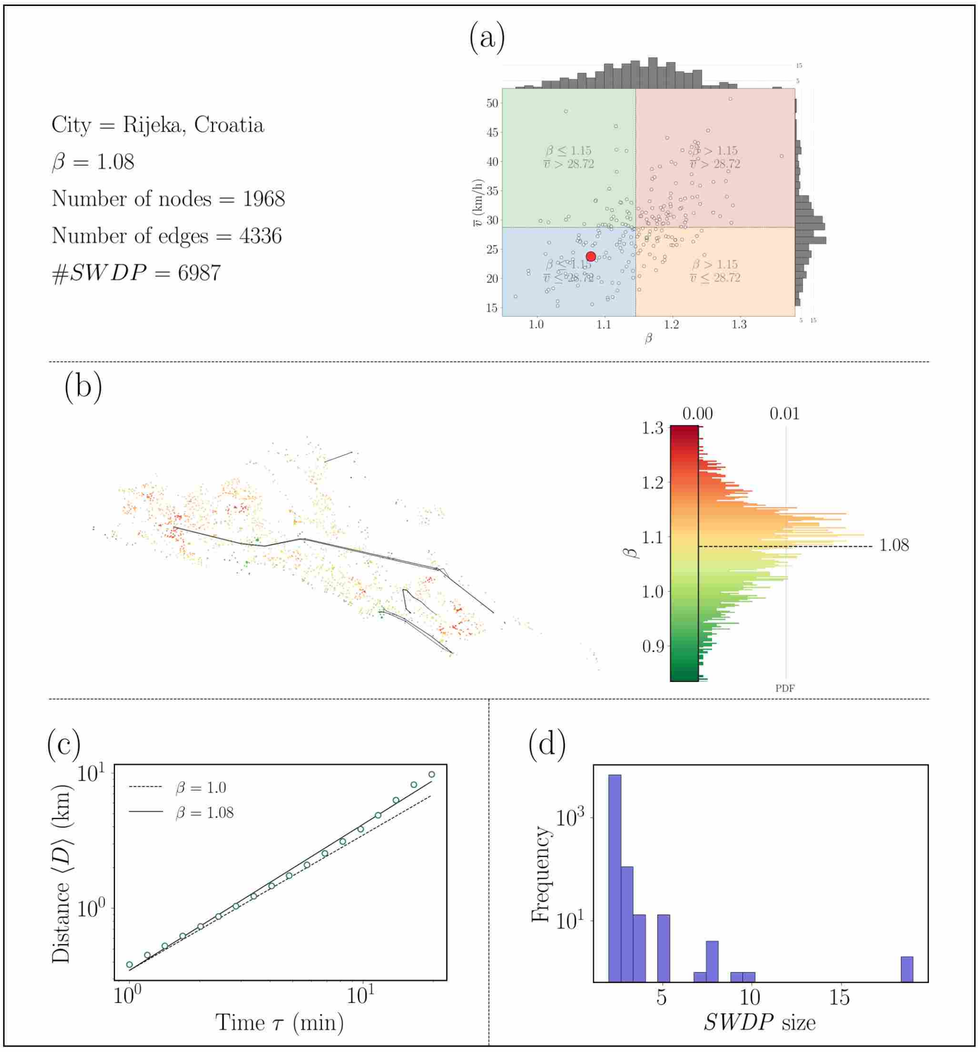

Fig. S156. Methodological sheet - Rijeka, Croatia. In (a) Each point represents a city, with mean exponent (β), on the x-axis, and mean speed $\overline{v}$ obtained in all trips made to calculate the exponent on the axis y. The histograms of the values of β and $\overline{v}$ are shown on the axes in the upper and right corners, respectively. The graph was segmented into four quadrants, in which the division is performed by the mean values of β and $\overline{v}$. The quadrants were colored and annotated according to the division criteria. The red dot represents the location of Rijeka, Croatia. In (b) taking all the nodes of Rijeka, Croatia as origin, the dots are colored as a function of their exponent value and their color is quantified by the color bar in the center. The longest segments without a deceleration point (SWDP) are plotted in black. The probability density function of the $\beta' s$ for each experiment is shown on the left of the color scale Figure (c) shows the mean correlation curve between time τ and the distance $\langle D \rangle$. The black traced line represents the exponent equal to 1.0. Figure (d) shows the distribution of SWDP sizes in number of nodes per frequency of occurrence.

Rio de Janeiro, Brasil

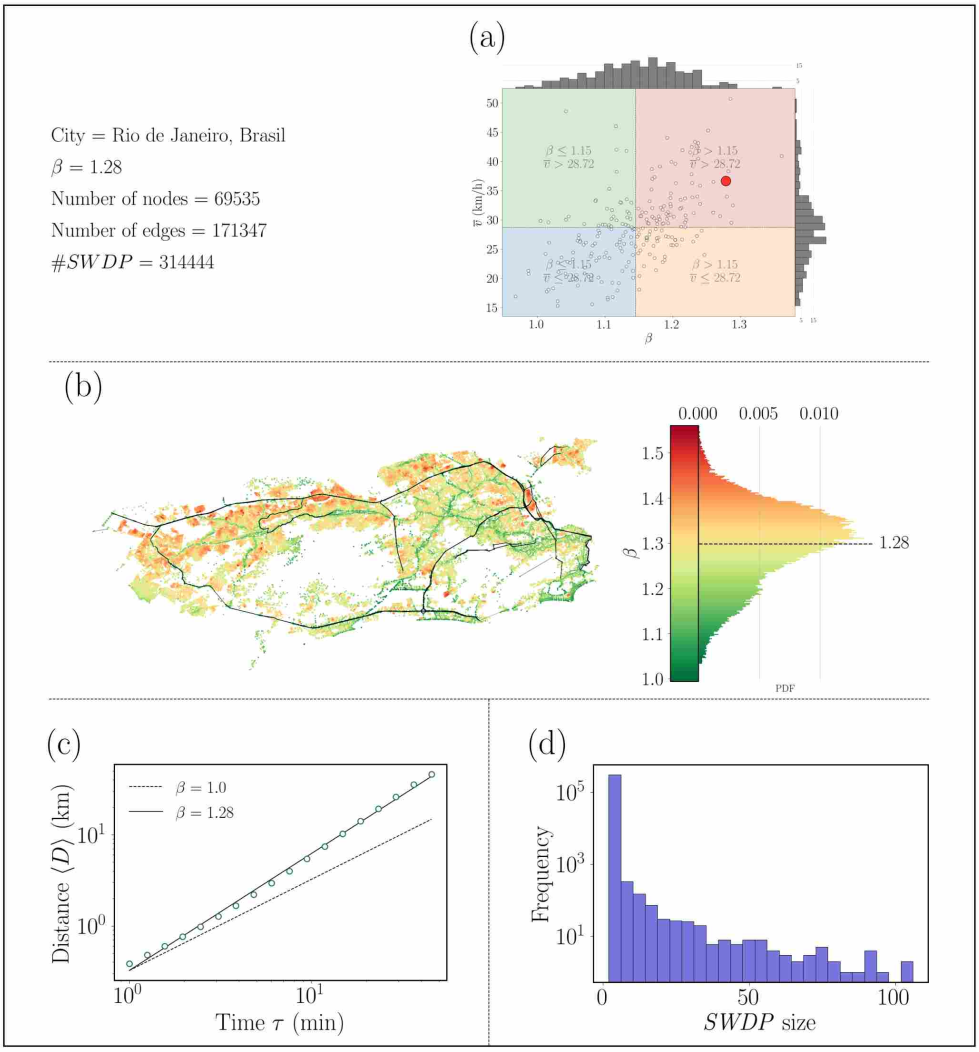

Fig. S157. Methodological sheet - Rio de Janeiro, Brasil. In (a) Each point represents a city, with mean exponent (β), on the x-axis, and mean speed $\overline{v}$ obtained in all trips made to calculate the exponent on the axis y. The histograms of the values of β and $\overline{v}$ are shown on the axes in the upper and right corners, respectively. The graph was segmented into four quadrants, in which the division is performed by the mean values of β and $\overline{v}$. The quadrants were colored and annotated according to the division criteria. The red dot represents the location of Rio de Janeiro, Brasil. In (b) taking all the nodes of Rio de Janeiro, Brasil as origin, the dots are colored as a function of their exponent value and their color is quantified by the color bar in the center. The longest segments without a deceleration point (SWDP) are plotted in black. The probability density function of the $\beta's$ for each experiment is shown on the left of the color scale Figure (c) shows the mean correlation curve between time τ and the distance $\langle D \rangle$. The black traced line represents the exponent equal to 1.0. Figure (d) shows the distribution of SWDP sizes in number of nodes per frequency of occurrence.

Saint Louis, Illinois, USA

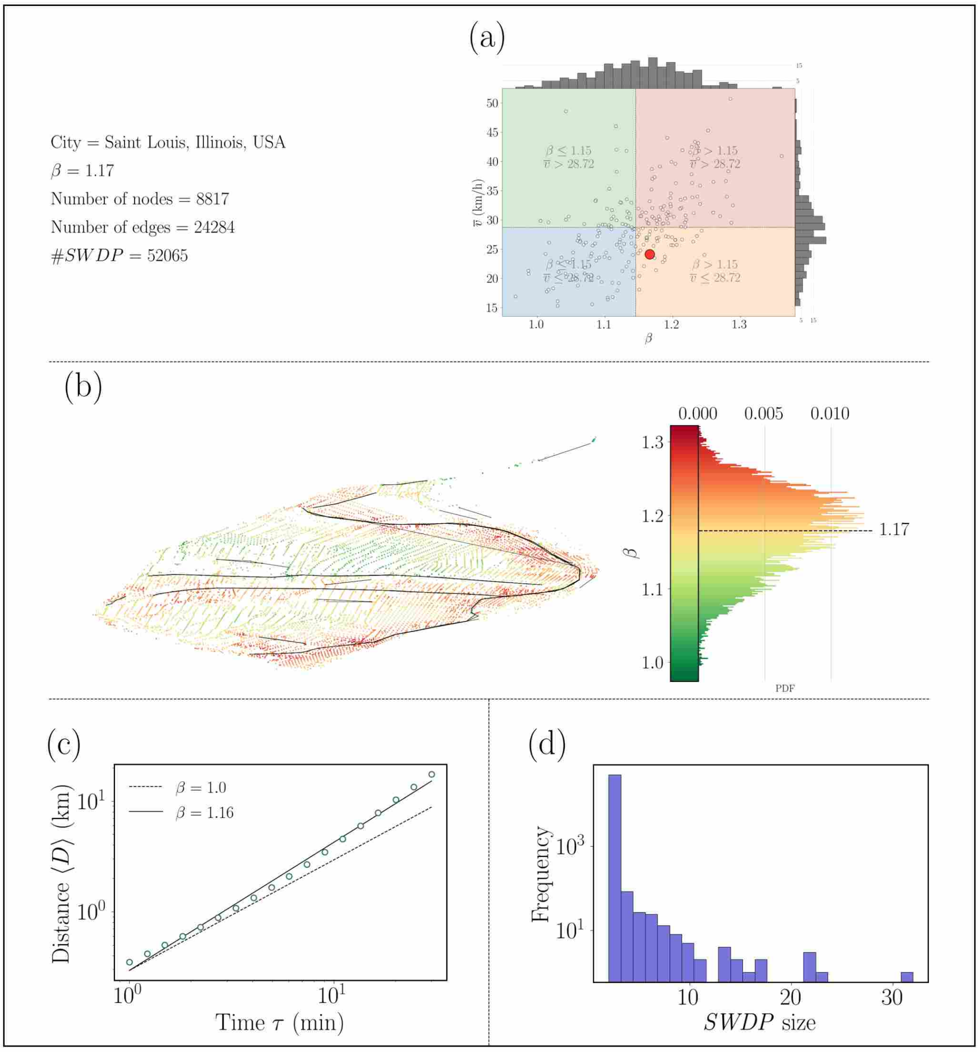

Fig. S158. Methodological sheet - Saint Louis, Illinois, USA. In (a) Each point represents a city, with mean exponent (β), on the x-axis, and mean speed $\overline{v}$ obtained in all trips made to calculate the exponent on the axis y. The histograms of the values of β and $\overline{v}$ are shown on the axes in the upper and right corners, respectively. The graph was segmented into four quadrants, in which the division is performed by the mean values of β and $\overline{v}$. The quadrants were colored and annotated according to the division criteria. The red dot represents the location of Saint Louis, Illinois, USA. In (b) taking all the nodes of Saint Louis, Illinois, USA as origin, the dots are colored as a function of their exponent value and their color is quantified by the color bar in the center. The longest segments without a deceleration point (SWDP) are plotted in black. The probability density function of the $\beta's$ for each experiment is shown on the left of the color scale Figure (c) shows the mean correlation curve between time τ and the distance $\langle D \rangle$. The black traced line represents the exponent equal to 1.0. Figure (d) shows the distribution of SWDP sizes in number of nodes per frequency of occurrence.

Salt Lake City, USA

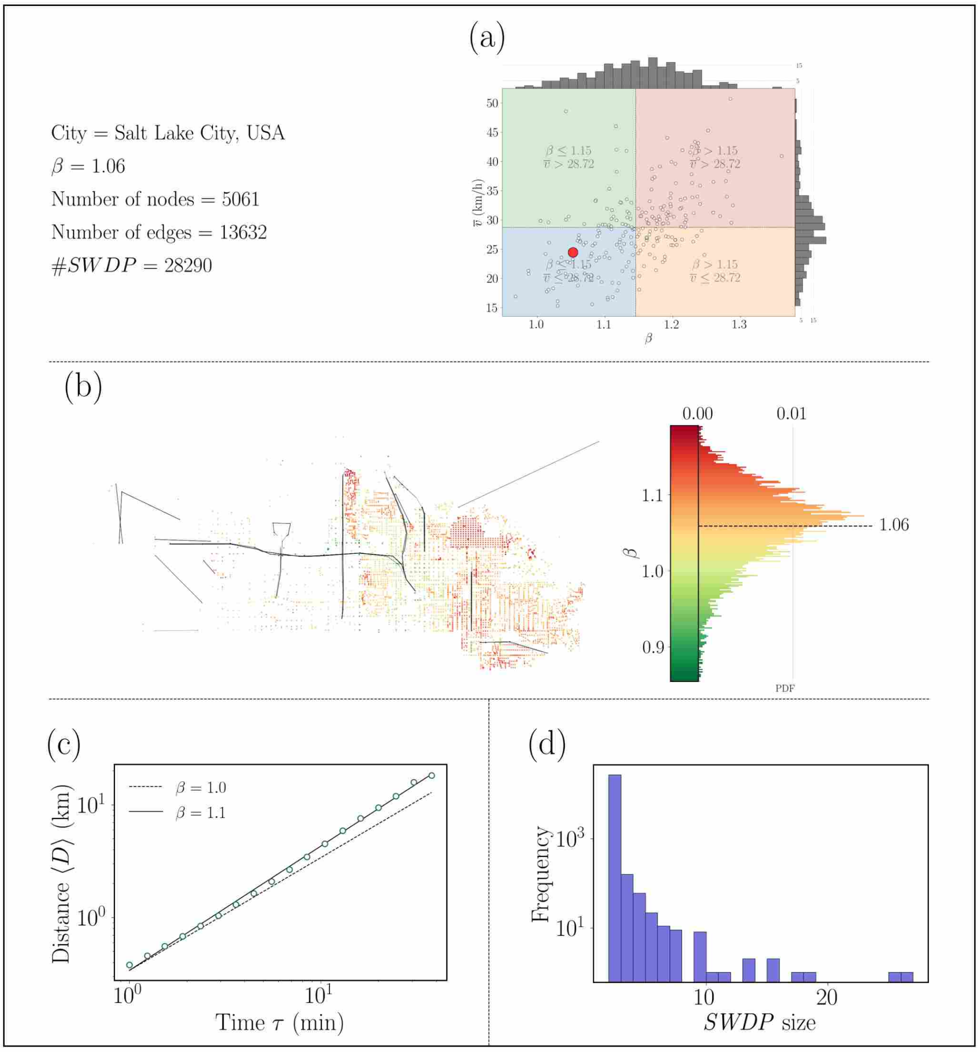

Fig. S159. Methodological sheet - Salt Lake City, USA. In (a) Each point represents a city, with mean exponent (β), on the x-axis, and mean speed $\overline{v}$ obtained in all trips made to calculate the exponent on the axis y. The histograms of the values of β and $\overline{v}$ are shown on the axes in the upper and right corners, respectively. The graph was segmented into four quadrants, in which the division is performed by the mean values of β and $\overline{v}$. The quadrants were colored and annotated according to the division criteria. The red dot represents the location of Salt Lake City, USA. In (b) taking all the nodes of Salt Lake City, USA as origin, the dots are colored as a function of their exponent value and their color is quantified by the color bar in the center. The longest segments without a deceleration point (SWDP) are plotted in black. The probability density function of the $\beta's$ for each experiment is shown on the left of the color scale Figure (c) shows the mean correlation curve between time τ and the distance $\langle D \rangle$. The black traced line represents the exponent equal to 1.0. Figure (d) shows the distribution of SWDP sizes in number of nodes per frequency of occurrence.

Salvador, Brasil

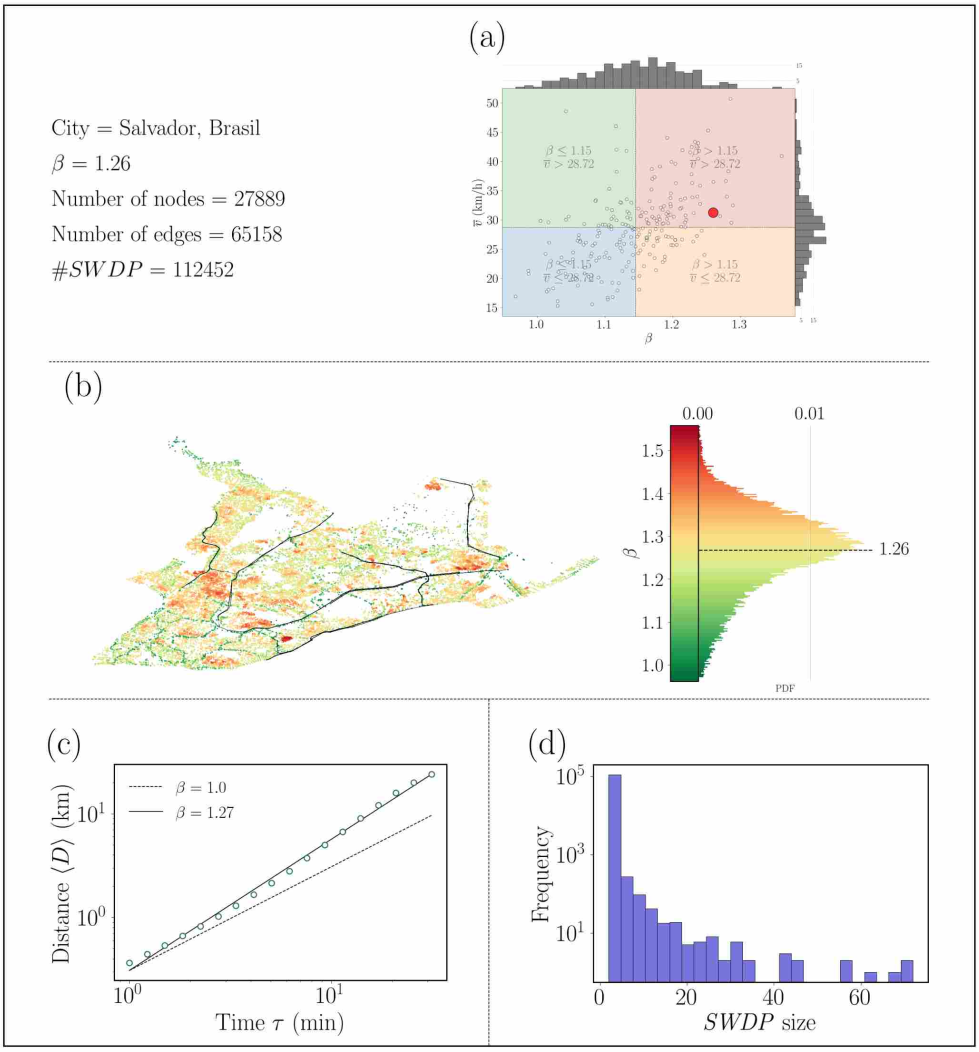

Fig. S160. Methodological sheet - Salvador, Brasil. In (a) Each point represents a city, with mean exponent (β), on the x-axis, and mean speed $\overline{v}$ obtained in all trips made to calculate the exponent on the axis y. The histograms of the values of β and $\overline{v}$ are shown on the axes in the upper and right corners, respectively. The graph was segmented into four quadrants, in which the division is performed by the mean values of β and $\overline{v}$. The quadrants were colored and annotated according to the division criteria. The red dot represents the location of Salvador, Brasil. In (b) taking all the nodes of Salvador, Brasil as origin, the dots are colored as a function of their exponent value and their color is quantified by the color bar in the center. The longest segments without a deceleration point (SWDP) are plotted in black. The probability density function of the $\beta's$ for each experiment is shown on the left of the color scale Figure (c) shows the mean correlation curve between time τ and the distance $\langle D \rangle$. The black traced line represents the exponent equal to 1.0. Figure (d) shows the distribution of SWDP sizes in number of nodes per frequency of occurrence.

Salzburg, Austria

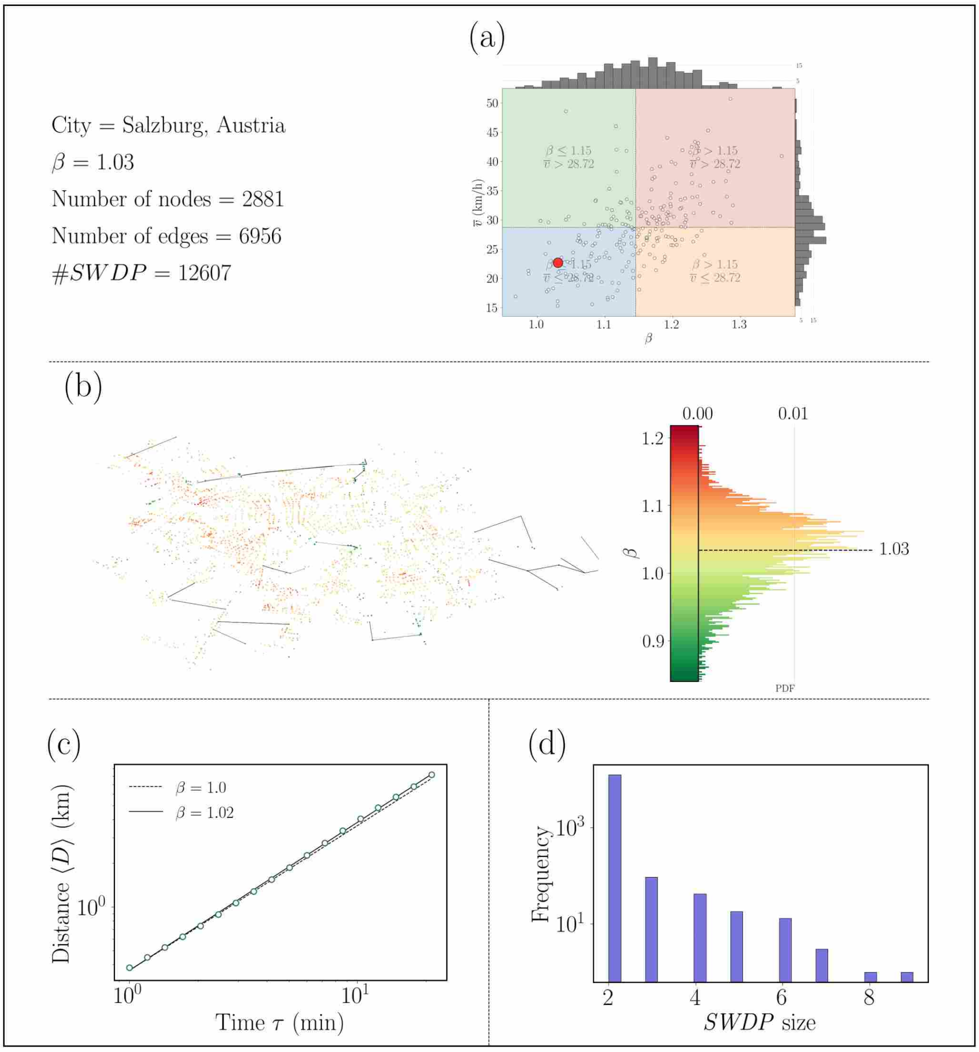

Fig. S161. Methodological sheet - Salzburg, Austria. In (a) Each point represents a city, with mean exponent (β), on the x-axis, and mean speed $\overline{v}$ obtained in all trips made to calculate the exponent on the axis y. The histograms of the values of β and $\overline{v}$ are shown on the axes in the upper and right corners, respectively. The graph was segmented into four quadrants, in which the division is performed by the mean values of β and $\overline{v}$. The quadrants were colored and annotated according to the division criteria. The red dot represents the location of Salzburg, Austria. In (b) taking all the nodes of Salzburg, Austria as origin, the dots are colored as a function of their exponent value and their color is quantified by the color bar in the center. The longest segments without a deceleration point (SWDP) are plotted in black. The probability density function of the $\beta's$ for each experiment is shown on the left of the color scale Figure (c) shows the mean correlation curve between time τ and the distance $\langle D \rangle$. The black traced line represents the exponent equal to 1.0. Figure (d) shows the distribution of SWDP sizes in number of nodes per frequency of occurrence.

San Antonio, USA

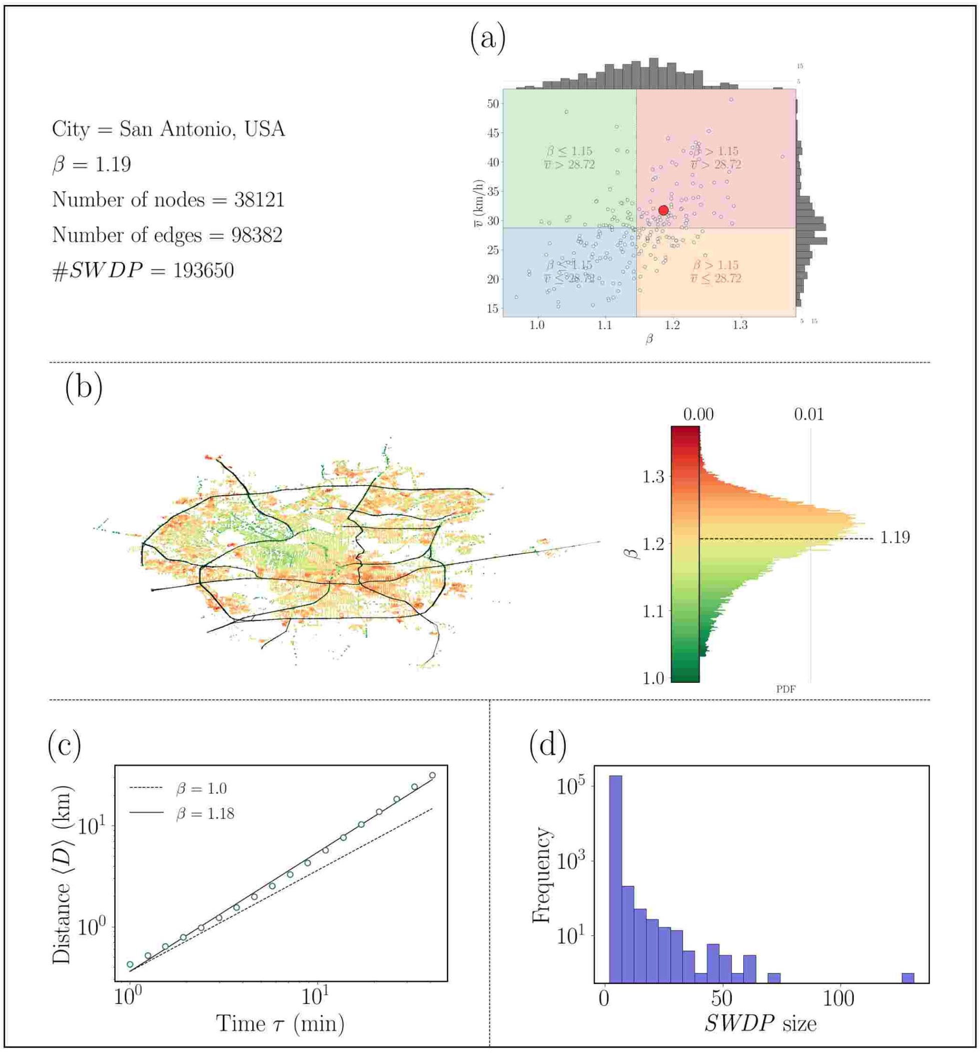

Fig. S162. Methodological sheet - San Antonio, USA. In (a) Each point represents a city, with mean exponent (β), on the x-axis, and mean speed $\overline{v}$ obtained in all trips made to calculate the exponent on the axis y. The histograms of the values of β and $\overline{v}$ are shown on the axes in the upper and right corners, respectively. The graph was segmented into four quadrants, in which the division is performed by the mean values of β and $\overline{v}$. The quadrants were colored and annotated according to the division criteria. The red dot represents the location of San Antonio, USA. In (b) taking all the nodes of San Antonio, USA as origin, the dots are colored as a function of their exponent value and their color is quantified by the color bar in the center. The longest segments without a deceleration point (SWDP) are plotted in black. The probability density function of the $\beta's$ for each experiment is shown on the left of the color scale Figure (c) shows the mean correlation curve between time τ and the distance $\langle D \rangle$. The black traced line represents the exponent equal to 1.0. Figure (d) shows the distribution of SWDP sizes in number of nodes per frequency of occurrence.

San Diego, USA

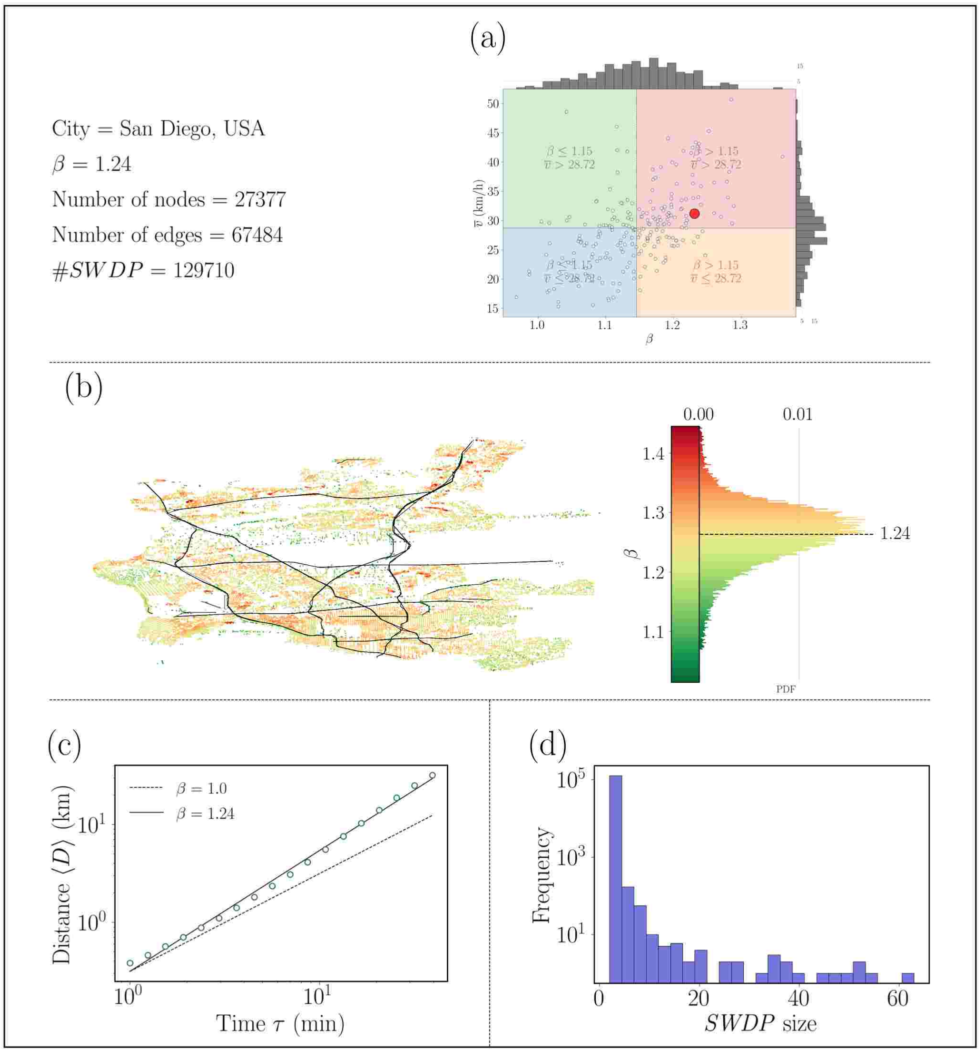

Fig. S163. Methodological sheet - San Diego, USA. In (a) Each point represents a city, with mean exponent (β), on the x-axis, and mean speed $\overline{v}$ obtained in all trips made to calculate the exponent on the axis y. The histograms of the values of β and $\overline{v}$ are shown on the axes in the upper and right corners, respectively. The graph was segmented into four quadrants, in which the division is performed by the mean values of β and $\overline{v}$. The quadrants were colored and annotated according to the division criteria. The red dot represents the location of San Diego, USA. In (b) taking all the nodes of San Diego, USA as origin, the dots are colored as a function of their exponent value and their color is quantified by the color bar in the center. The longest segments without a deceleration point (SWDP) are plotted in black. The probability density function of the $\beta's$ for each experiment is shown on the left of the color scale Figure (c) shows the mean correlation curve between time τ and the distance $\langle D \rangle$. The black traced line represents the exponent equal to 1.0. Figure (d) shows the distribution of SWDP sizes in number of nodes per frequency of occurrence.

Santa Fe, USA

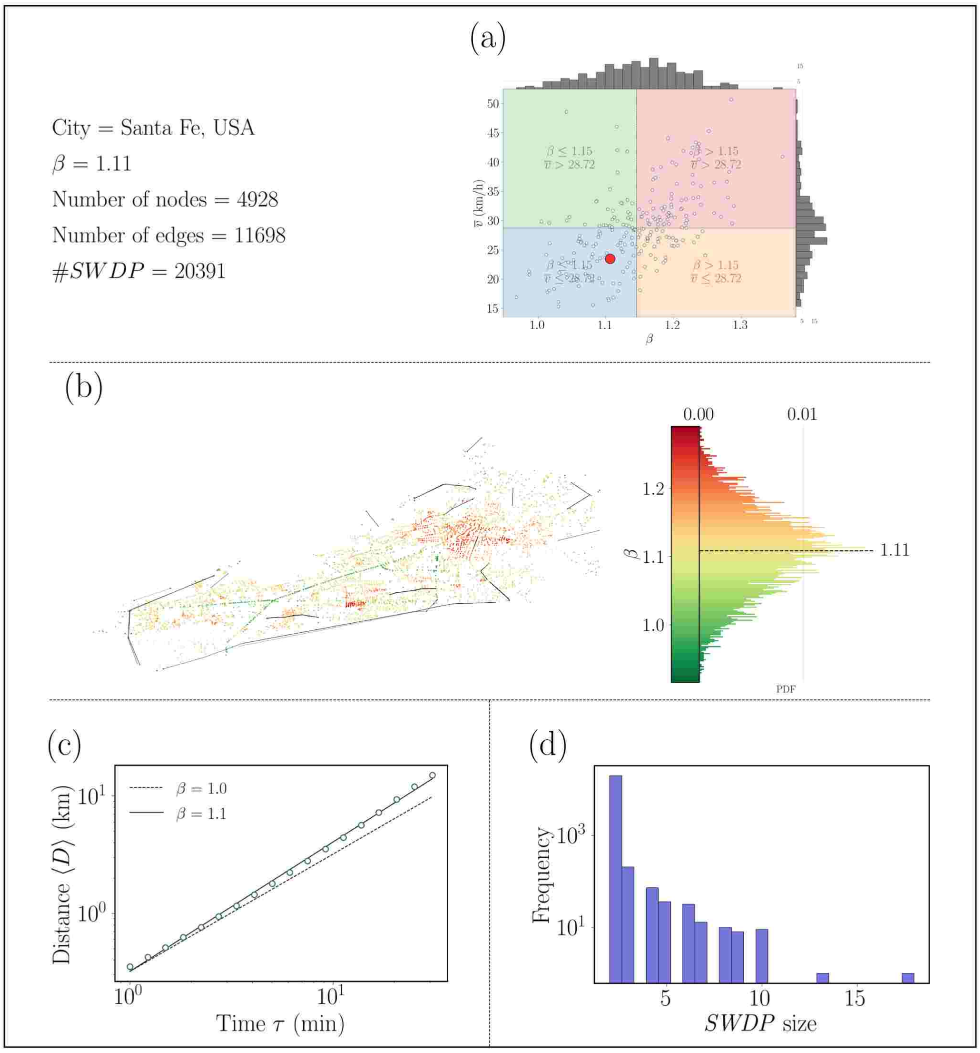

Fig. S164. Methodological sheet - Santa Fe, USA. In (a) Each point represents a city, with mean exponent (β), on the x-axis, and mean speed $\overline{v}$ obtained in all trips made to calculate the exponent on the axis y. The histograms of the values of β and $\overline{v}$ are shown on the axes in the upper and right corners, respectively. The graph was segmented into four quadrants, in which the division is performed by the mean values of β and $\overline{v}$. The quadrants were colored and annotated according to the division criteria. The red dot represents the location of Santa Fe, USA. In (b) taking all the nodes of Santa Fe, USA as origin, the dots are colored as a function of their exponent value and their color is quantified by the color bar in the center. The longest segments without a deceleration point (SWDP) are plotted in black. The probability density function of the $\beta's$ for each experiment is shown on the left of the color scale Figure (c) shows the mean correlation curve between time τ and the distance $\langle D \rangle$. The black traced line represents the exponent equal to 1.0. Figure (d) shows the distribution of SWDP sizes in number of nodes per frequency of occurrence.

Santos, Brasil

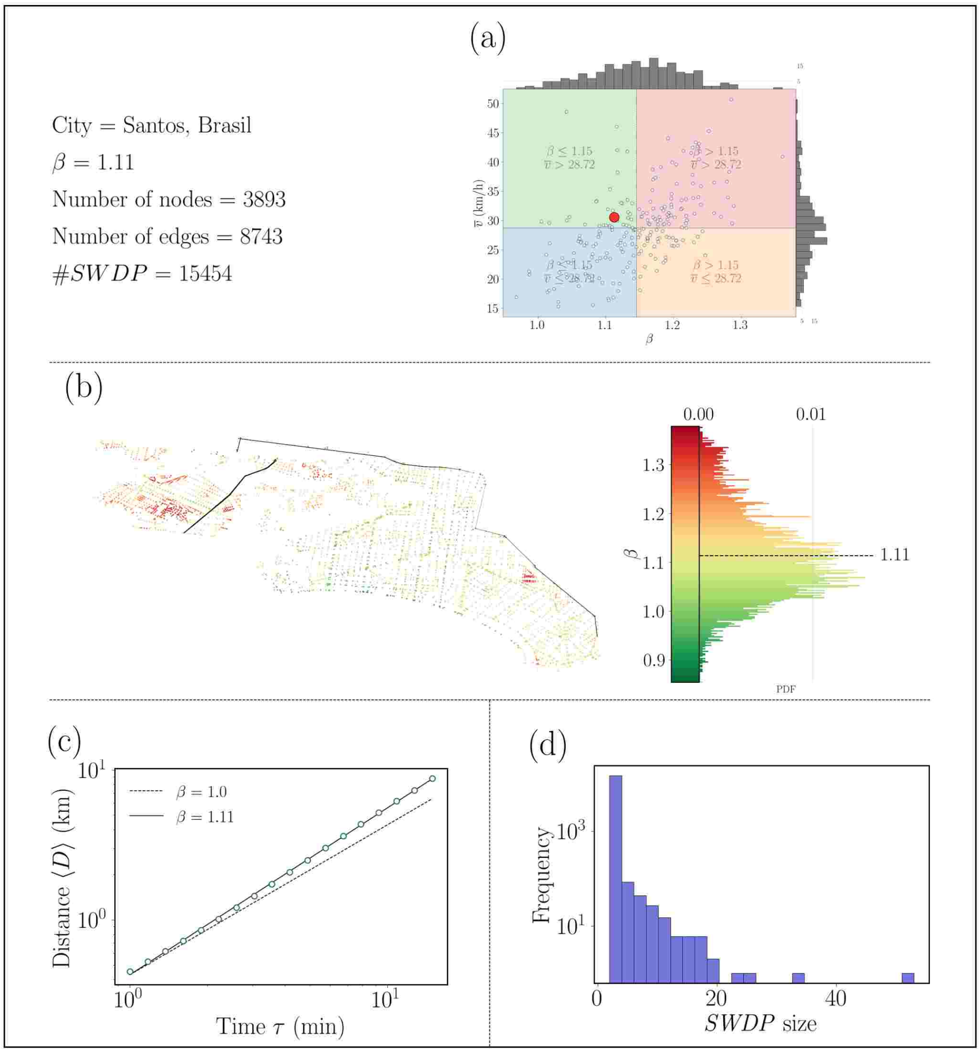

Fig. S165. Methodological sheet - Santos, Brasil. In (a) Each point represents a city, with mean exponent (β), on the x-axis, and mean speed $\overline{v}$ obtained in all trips made to calculate the exponent on the axis y. The histograms of the values of β and $\overline{v}$ are shown on the axes in the upper and right corners, respectively. The graph was segmented into four quadrants, in which the division is performed by the mean values of β and $\overline{v}$. The quadrants were colored and annotated according to the division criteria. The red dot represents the location of Santos, Brasil. In (b) taking all the nodes of Santos, Brasil as origin, the dots are colored as a function of their exponent value and their color is quantified by the color bar in the center. The longest segments without a deceleration point (SWDP) are plotted in black. The probability density function of the $\beta's$ for each experiment is shown on the left of the color scale Figure (c) shows the mean correlation curve between time τ and the distance $\langle D \rangle$. The black traced line represents the exponent equal to 1.0. Figure (d) shows the distribution of SWDP sizes in number of nodes per frequency of occurrence.

Sapporo, Japan

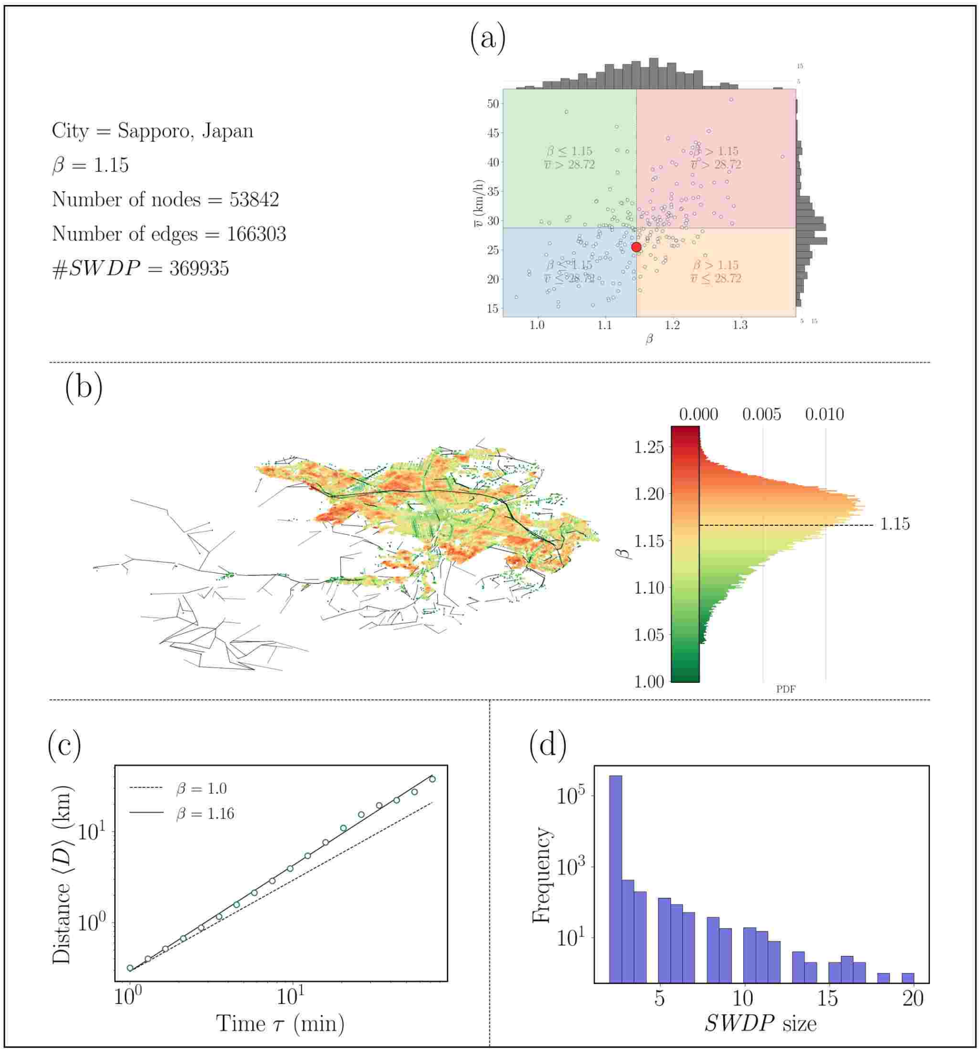

Fig. S166. Methodological sheet - Sapporo, Japan. In (a) Each point represents a city, with mean exponent (β), on the x-axis, and mean speed $\overline{v}$ obtained in all trips made to calculate the exponent on the axis y. The histograms of the values of β and $\overline{v}$ are shown on the axes in the upper and right corners, respectively. The graph was segmented into four quadrants, in which the division is performed by the mean values of β and $\overline{v}$. The quadrants were colored and annotated according to the division criteria. The red dot represents the location of Sapporo, Japan. In (b) taking all the nodes of Sapporo, Japan as origin, the dots are colored as a function of their exponent value and their color is quantified by the color bar in the center. The longest segments without a deceleration point (SWDP) are plotted in black. The probability density function of the $\beta's$ for each experiment is shown on the left of the color scale Figure (c) shows the mean correlation curve between time τ and the distance $\langle D \rangle$. The black traced line represents the exponent equal to 1.0. Figure (d) shows the distribution of SWDP sizes in number of nodes per frequency of occurrence.

Sarajevo, Bosnia and Herzegovina

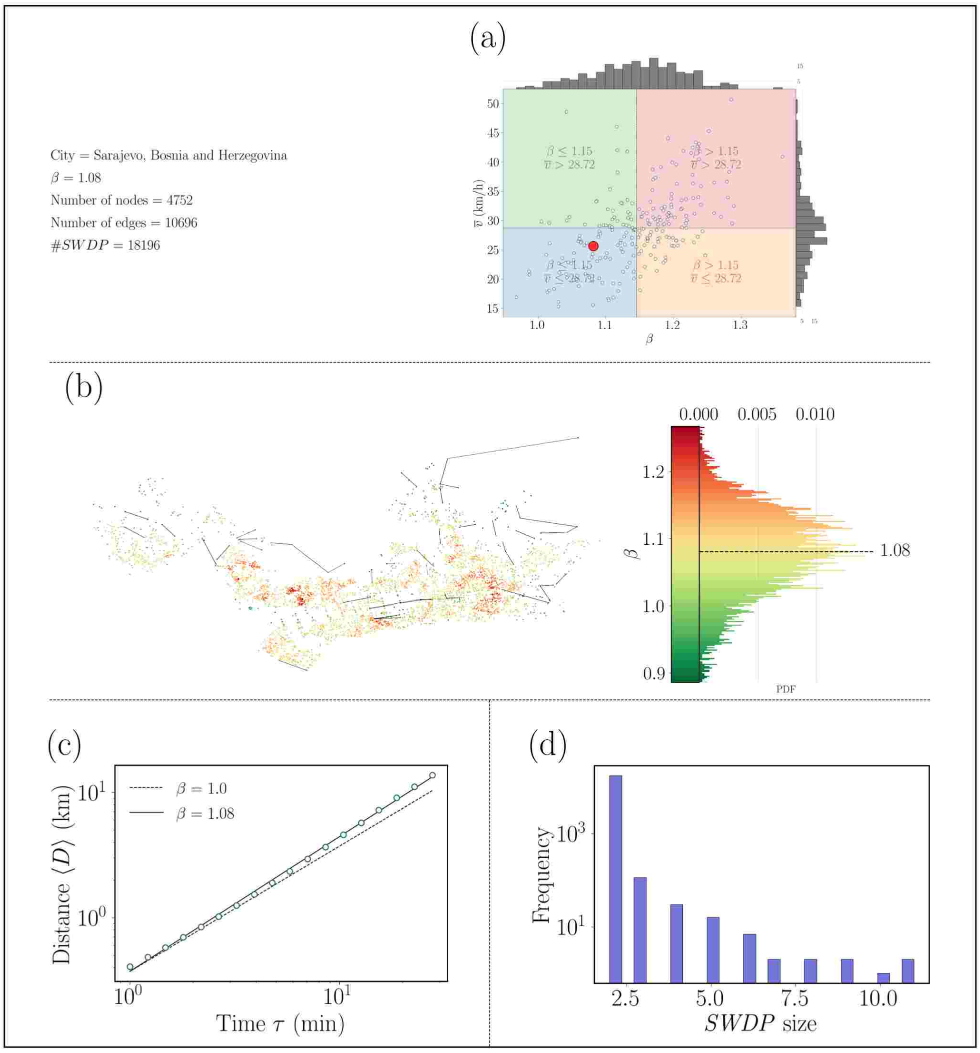

Fig. S167. Methodological sheet - Sarajevo, Bosnia and Herzegovina. In (a) Each point represents a city, with mean exponent (β), on the x-axis, and mean speed $\overline{v}$ obtained in all trips made to calculate the exponent on the axis y. The histograms of the values of β and $\overline{v}$ are shown on the axes in the upper and right corners, respectively. The graph was segmented into four quadrants, in which the division is performed by the mean values of β and $\overline{v}$. The quadrants were colored and annotated according to the division criteria. The red dot represents the location of Sarajevo, Bosnia and Herzegovina. In (b) taking all the nodes of Sarajevo, Bosnia and Herzegovina as origin, the dots are colored as a function of their exponent value and their color is quantified by the color bar in the center. The longest segments without a deceleration point (SWDP) are plotted in black. The probability density function of the $\beta's$ for each experiment is shown on the left of the color scale Figure (c) shows the mean correlation curve between time τ and the distance $\langle D \rangle$. The black traced line represents the exponent equal to 1.0. Figure (d) shows the distribution of SWDP sizes in number of nodes per frequency of occurrence.

Saskatoon, Canadá

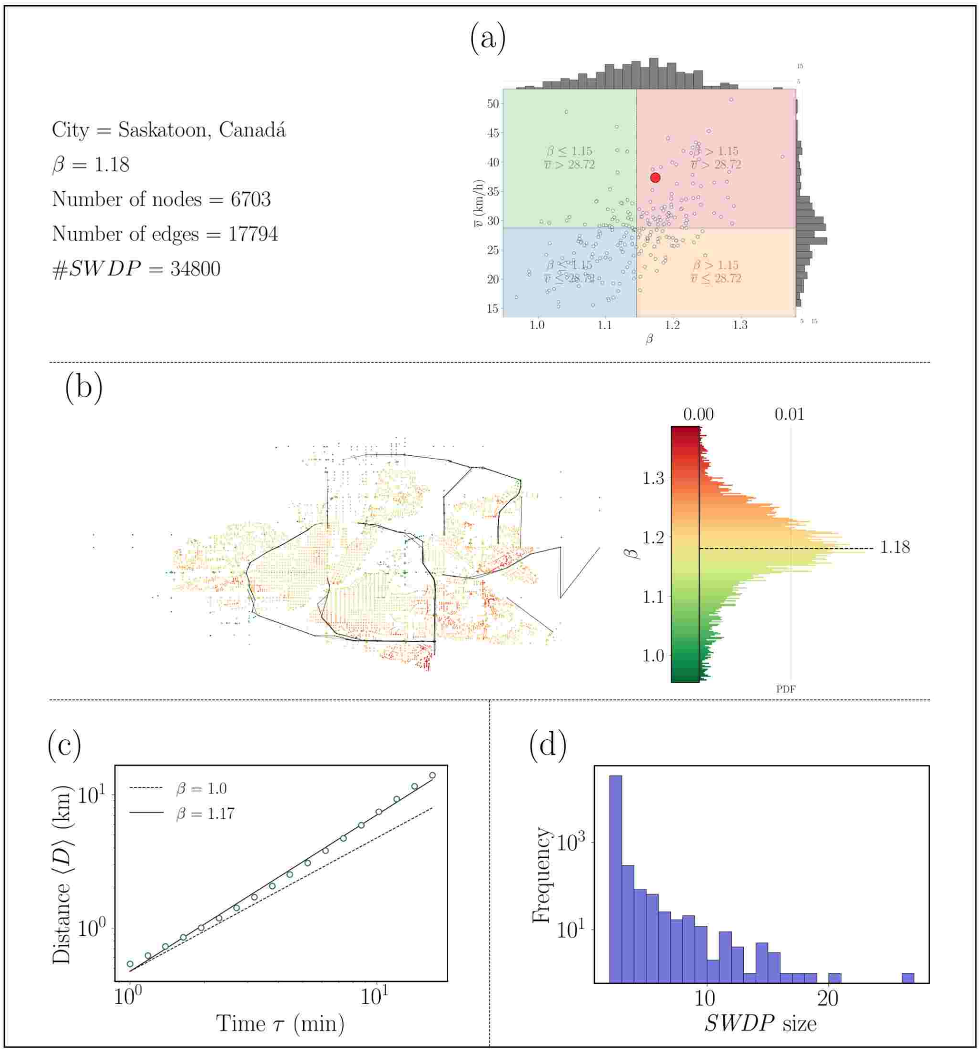

Fig. S168. Methodological sheet - Saskatoon, Canadá. In (a) Each point represents a city, with mean exponent (β), on the x-axis, and mean speed $\overline{v}$ obtained in all trips made to calculate the exponent on the axis y. The histograms of the values of β and $\overline{v}$ are shown on the axes in the upper and right corners, respectively. The graph was segmented into four quadrants, in which the division is performed by the mean values of β and $\overline{v}$. The quadrants were colored and annotated according to the division criteria. The red dot represents the location of Saskatoon, Canadá. In (b) taking all the nodes of Saskatoon, Canadá as origin, the dots are colored as a function of their exponent value and their color is quantified by the color bar in the center. The longest segments without a deceleration point (SWDP) are plotted in black. The probability density function of the $\beta's$ for each experiment is shown on the left of the color scale Figure (c) shows the mean correlation curve between time τ and the distance $\langle D \rangle$. The black traced line represents the exponent equal to 1.0. Figure (d) shows the distribution of SWDP sizes in number of nodes per frequency of occurrence.

Seattle, USA

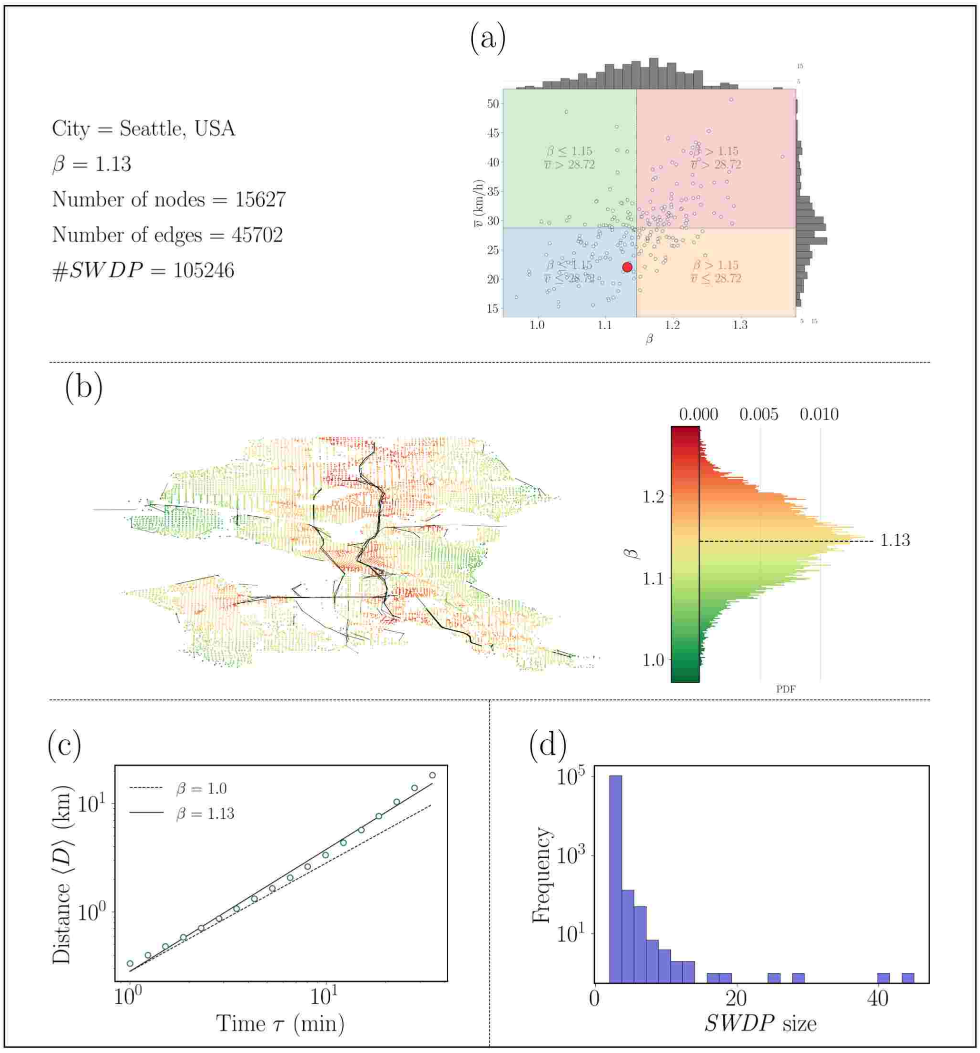

Fig. S169. Methodological sheet - Seattle, USA. In (a) Each point represents a city, with mean exponent (β), on the x-axis, and mean speed $\overline{v}$ obtained in all trips made to calculate the exponent on the axis y. The histograms of the values of β and $\overline{v}$ are shown on the axes in the upper and right corners, respectively. The graph was segmented into four quadrants, in which the division is performed by the mean values of β and $\overline{v}$. The quadrants were colored and annotated according to the division criteria. The red dot represents the location of Seattle, USA. In (b) taking all the nodes of Seattle, USA as origin, the dots are colored as a function of their exponent value and their color is quantified by the color bar in the center. The longest segments without a deceleration point (SWDP) are plotted in black. The probability density function of the $\beta's$ for each experiment is shown on the left of the color scale Figure (c) shows the mean correlation curve between time τ and the distance $\langle D \rangle$. The black traced line represents the exponent equal to 1.0. Figure (d) shows the distribution of SWDP sizes in number of nodes per frequency of occurrence.

Seoul, South Korea

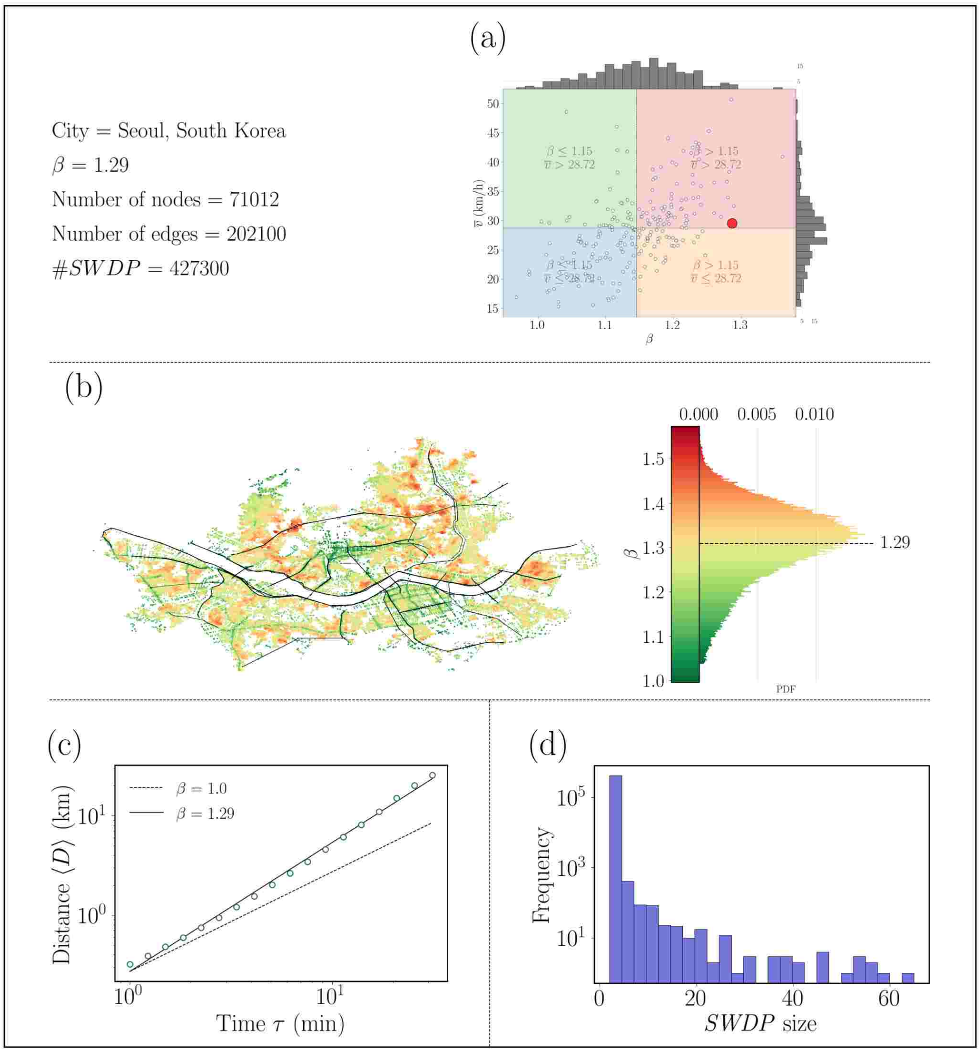

Fig. S170. Methodological sheet - Seoul, South Korea. In (a) Each point represents a city, with mean exponent (β), on the x-axis, and mean speed $\overline{v}$ obtained in all trips made to calculate the exponent on the axis y. The histograms of the values of β and $\overline{v}$ are shown on the axes in the upper and right corners, respectively. The graph was segmented into four quadrants, in which the division is performed by the mean values of β and $\overline{v}$. The quadrants were colored and annotated according to the division criteria. The red dot represents the location of Seoul, South Korea. In (b) taking all the nodes of Seoul, South Korea as origin, the dots are colored as a function of their exponent value and their color is quantified by the color bar in the center. The longest segments without a deceleration point (SWDP) are plotted in black. The probability density function of the $\beta's$ for each experiment is shown on the left of the color scale Figure (c) shows the mean correlation curve between time τ and the distance $\langle D \rangle$. The black traced line represents the exponent equal to 1.0. Figure (d) shows the distribution of SWDP sizes in number of nodes per frequency of occurrence.

Shenzhen, China

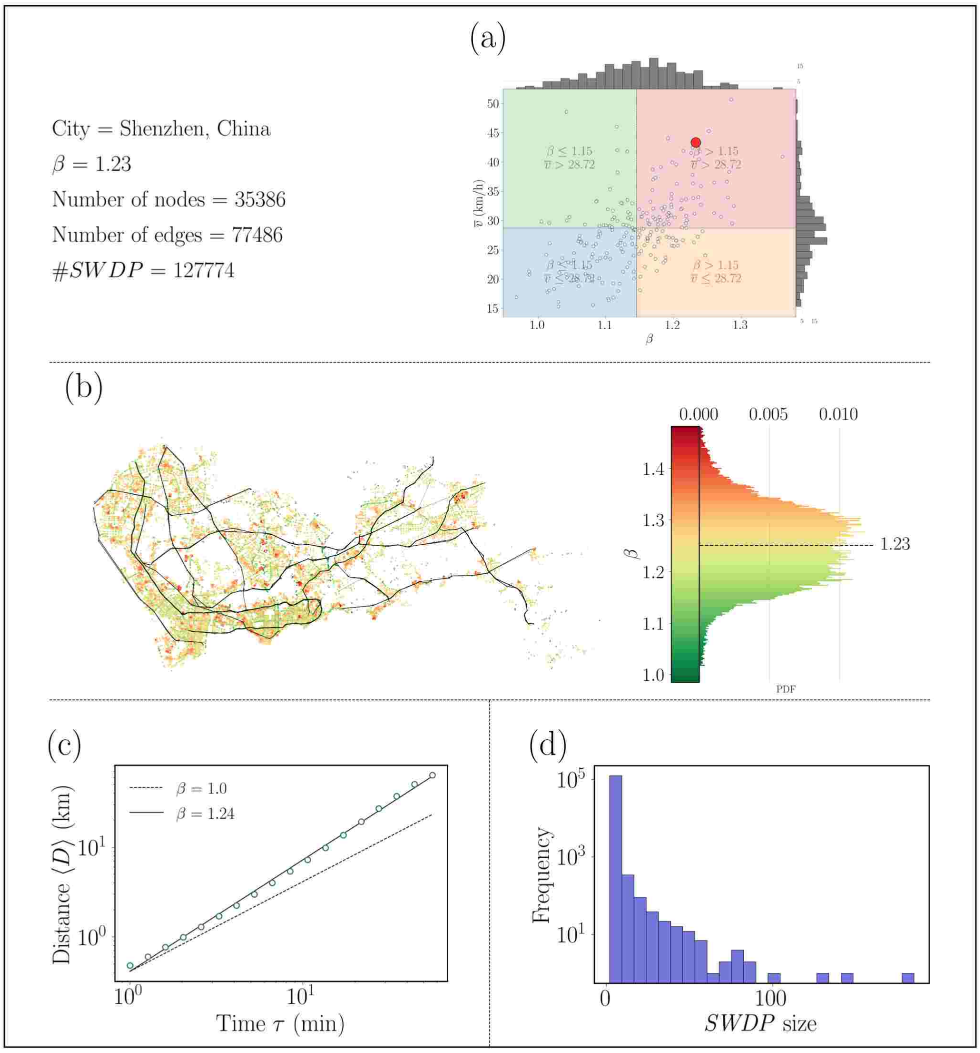

Fig. S171. Methodological sheet - Shenzhen, China. In (a) Each point represents a city, with mean exponent (β), on the x-axis, and mean speed $\overline{v}$ obtained in all trips made to calculate the exponent on the axis y. The histograms of the values of β and $\overline{v}$ are shown on the axes in the upper and right corners, respectively. The graph was segmented into four quadrants, in which the division is performed by the mean values of β and $\overline{v}$. The quadrants were colored and annotated according to the division criteria. The red dot represents the location of Shenzhen, China. In (b) taking all the nodes of Shenzhen, China as origin, the dots are colored as a function of their exponent value and their color is quantified by the color bar in the center. The longest segments without a deceleration point (SWDP) are plotted in black. The probability density function of the $\beta's$ for each experiment is shown on the left of the color scale Figure (c) shows the mean correlation curve between time τ and the distance $\langle D \rangle$. The black traced line represents the exponent equal to 1.0. Figure (d) shows the distribution of SWDP sizes in number of nodes per frequency of occurrence.

Singapore

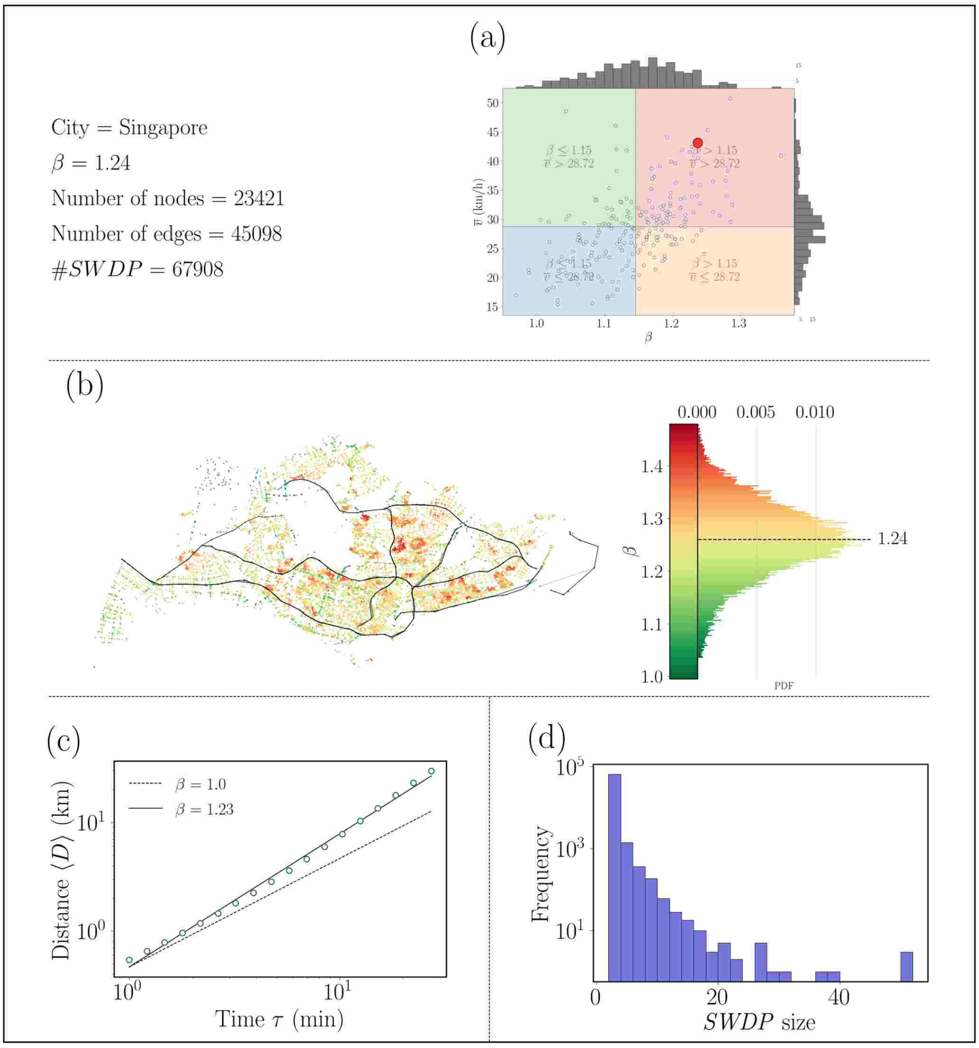

Fig. S172. Methodological sheet - Singapore. In (a) Each point represents a city, with mean exponent (β), on the x-axis, and mean speed $\overline{v}$ obtained in all trips made to calculate the exponent on the axis y. The histograms of the values of β and $\overline{v}$ are shown on the axes in the upper and right corners, respectively. The graph was segmented into four quadrants, in which the division is performed by the mean values of β and $\overline{v}$. The quadrants were colored and annotated according to the division criteria. The red dot represents the location of Singapore. In (b) taking all the nodes of Singapore as origin, the dots are colored as a function of their exponent value and their color is quantified by the color bar in the center. The longest segments without a deceleration point (SWDP) are plotted in black. The probability density function of the $\beta's$ for each experiment is shown on the left of the color scale Figure (c) shows the mean correlation curve between time τ and the distance $\langle D \rangle$. The black traced line represents the exponent equal to 1.0. Figure (d) shows the distribution of SWDP sizes in number of nodes per frequency of occurrence.

Sofia, Bulgaria

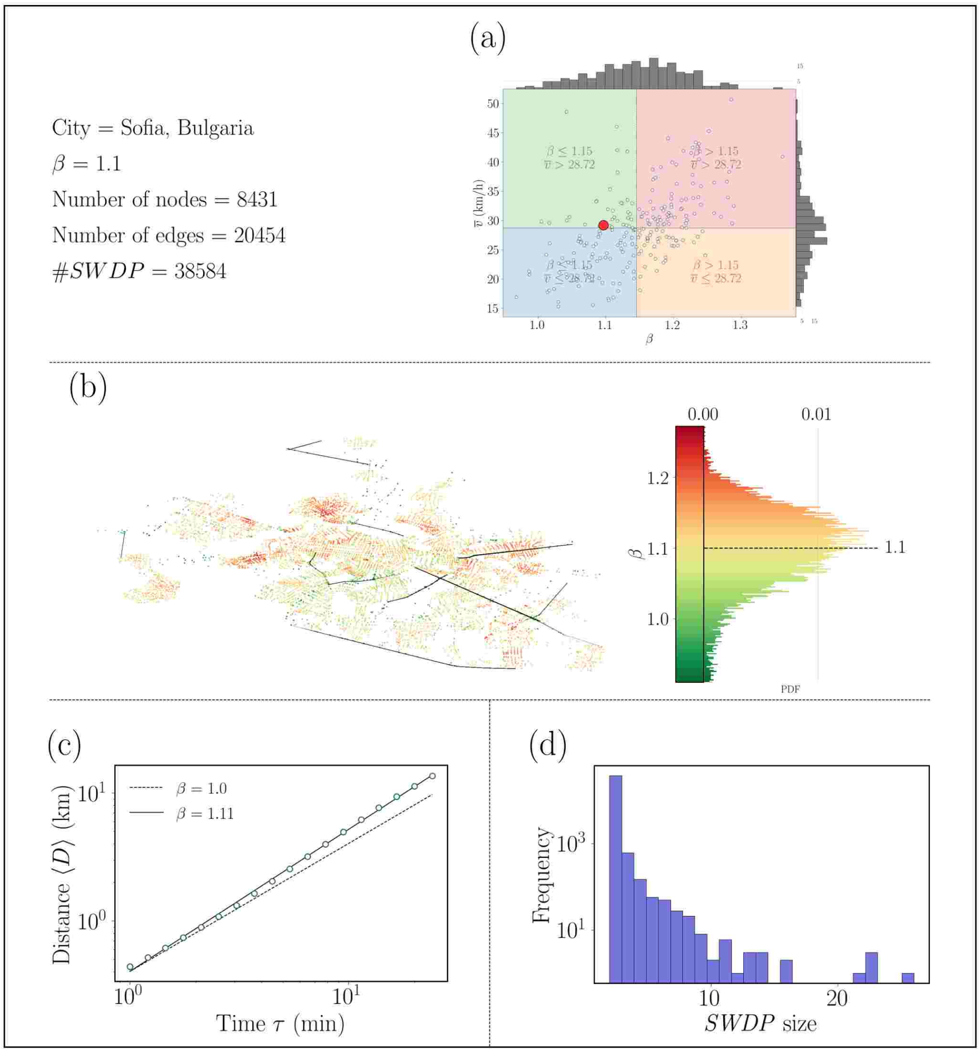

Fig. S173. Methodological sheet - Sofia, Bulgaria. In (a) Each point represents a city, with mean exponent (β), on the x-axis, and mean speed $\overline{v}$ obtained in all trips made to calculate the exponent on the axis y. The histograms of the values of β and $\overline{v}$ are shown on the axes in the upper and right corners, respectively. The graph was segmented into four quadrants, in which the division is performed by the mean values of β and $\overline{v}$. The quadrants were colored and annotated according to the division criteria. The red dot represents the location of Sofia, Bulgaria. In (b) taking all the nodes of Sofia, Bulgaria as origin, the dots are colored as a function of their exponent value and their color is quantified by the color bar in the center. The longest segments without a deceleration point (SWDP) are plotted in black. The probability density function of the $\beta' s$ for each experiment is shown on the left of the color scale Figure (c) shows the mean correlation curve between time τ and the distance $\langle D \rangle$. The black traced line represents the exponent equal to 1.0. Figure (d) shows the distribution of SWDP sizes in number of nodes per frequency of occurrence.

Sundsvall, Sweden

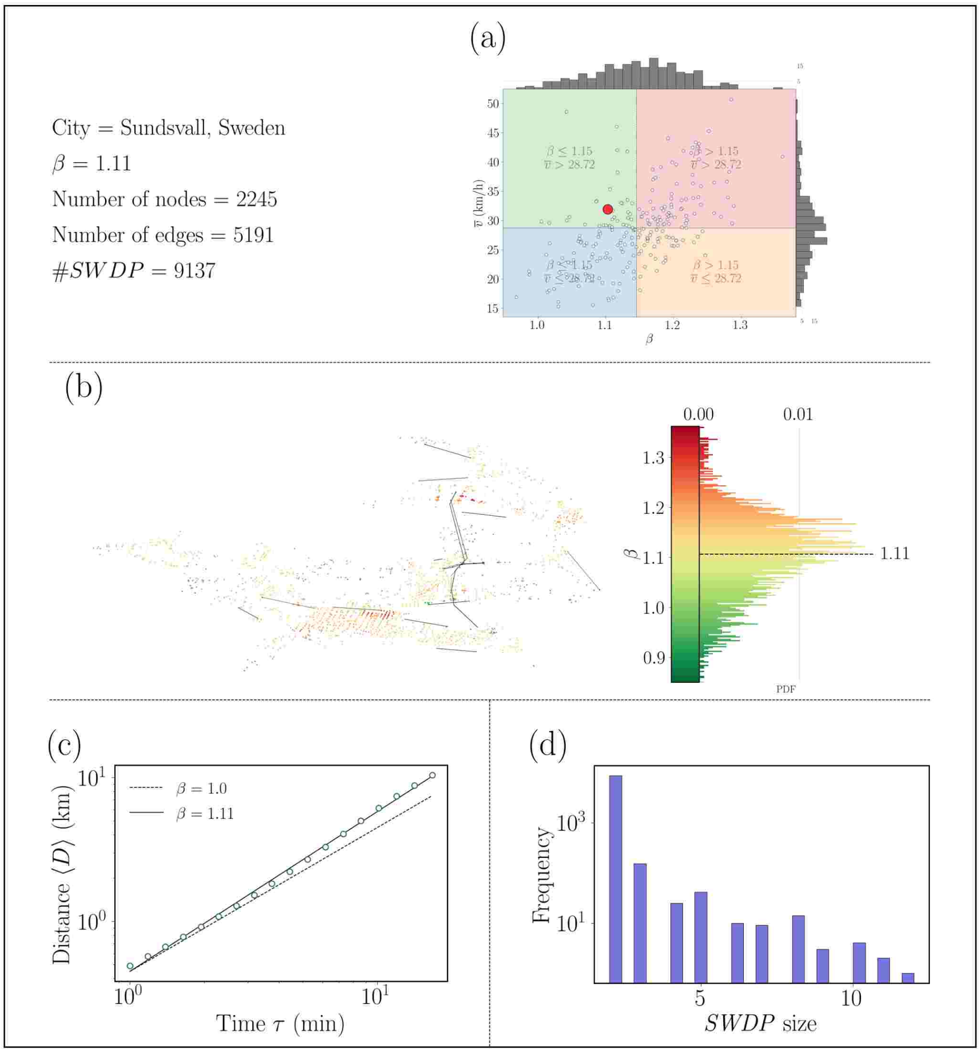

Fig. S174. Methodological sheet - Sundsvall, Sweden. In (a) Each point represents a city, with mean exponent (β), on the x-axis, and mean speed $\overline{v}$ obtained in all trips made to calculate the exponent on the axis y. The histograms of the values of β and $\overline{v}$ are shown on the axes in the upper and right corners, respectively. The graph was segmented into four quadrants, in which the division is performed by the mean values of β and $\overline{v}$. The quadrants were colored and annotated according to the division criteria. The red dot represents the location of Sundsvall, Sweden. In (b) taking all the nodes of Sundsvall, Sweden as origin, the dots are colored as a function of their exponent value and their color is quantified by the color bar in the center. The longest segments without a deceleration point (SWDP) are plotted in black. The probability density function of the $\beta's$ for each experiment is shown on the left of the color scale Figure (c) shows the mean correlation curve between time τ and the distance $\langle D \rangle$. The black traced line represents the exponent equal to 1.0. Figure (d) shows the distribution of SWDP sizes in number of nodes per frequency of occurrence.

Sydney, Australia

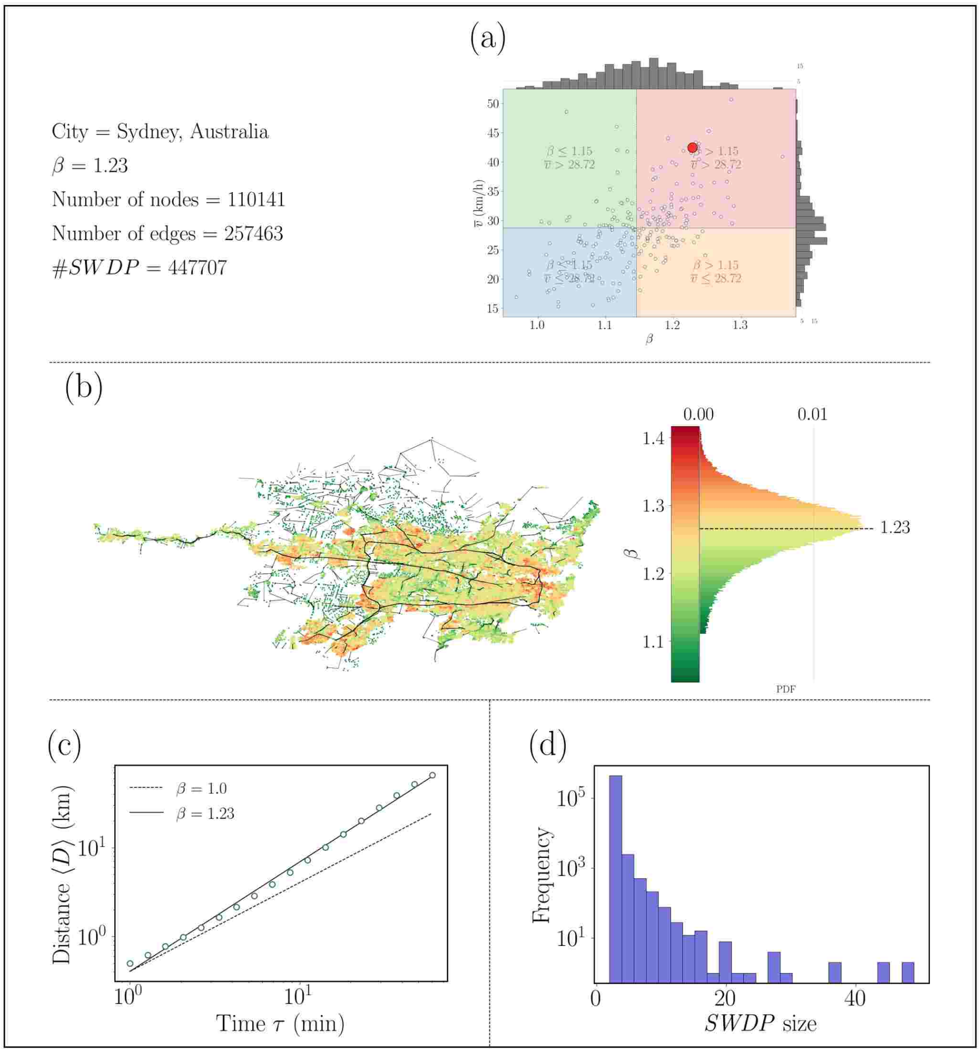

Fig. S175. Methodological sheet - Sydney, Australia. In (a) Each point represents a city, with mean exponent (β), on the x-axis, and mean speed $\overline{v}$ obtained in all trips made to calculate the exponent on the axis y. The histograms of the values of β and $\overline{v}$ are shown on the axes in the upper and right corners, respectively. The graph was segmented into four quadrants, in which the division is performed by the mean values of β and $\overline{v}$. The quadrants were colored and annotated according to the division criteria. The red dot represents the location of Sydney, Australia. In (b) taking all the nodes of Sydney, Australia as origin, the dots are colored as a function of their exponent value and their color is quantified by the color bar in the center. The longest segments without a deceleration point (SWDP) are plotted in black. The probability density function of the $\beta's$ for each experiment is shown on the left of the color scale Figure (c) shows the mean correlation curve between time τ and the distance $\langle D \rangle$. The black traced line represents the exponent equal to 1.0. Figure (d) shows the distribution of SWDP sizes in number of nodes per frequency of occurrence.

São Bernardo do Campo, Brasil

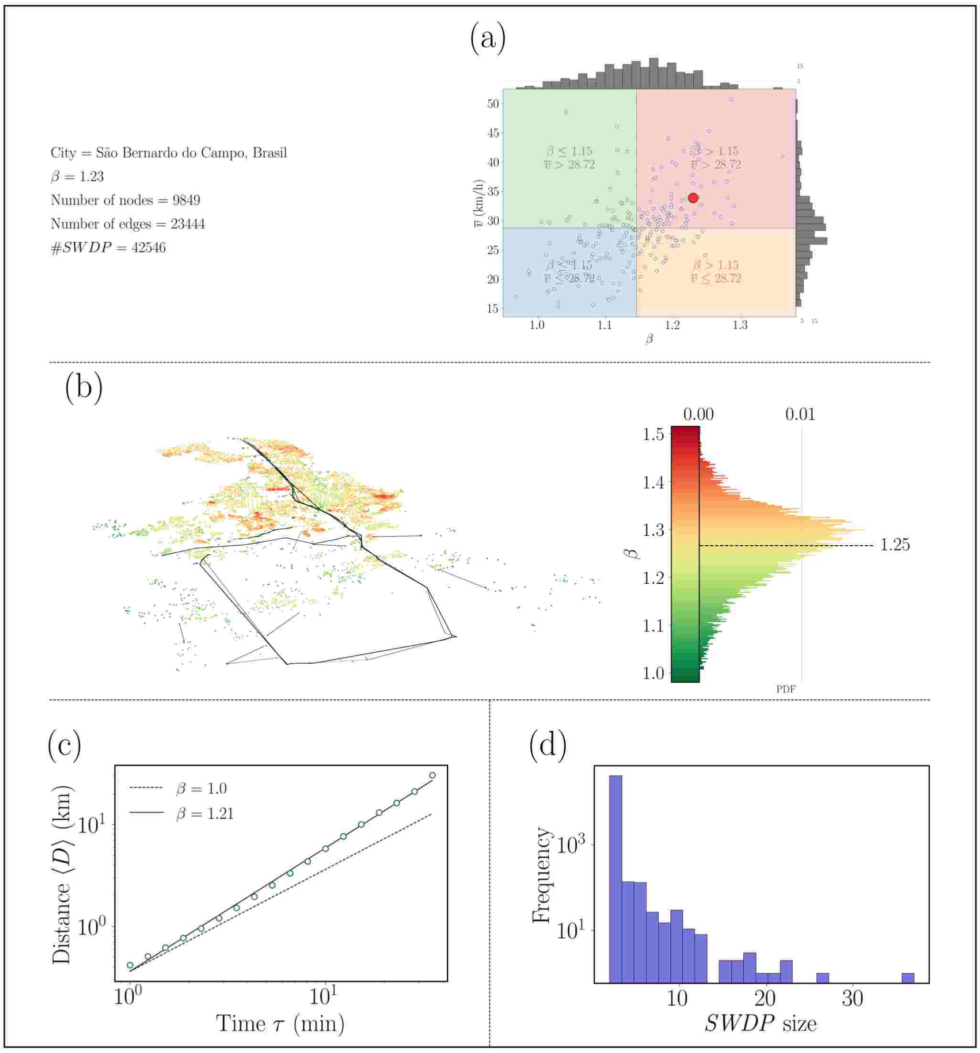

Fig. S176. Methodological sheet - São Bernardo do Campo, Brasil. In (a) Each point represents a city, with mean exponent (β), on the x-axis, and mean speed $\overline{v}$ obtained in all trips made to calculate the exponent on the axis y. The histograms of the values of β and $\overline{v}$ are shown on the axes in the upper and right corners, respectively. The graph was segmented into four quadrants, in which the division is performed by the mean values of β and $\overline{v}$. The quadrants were colored and annotated according to the division criteria. The red dot represents the location of São Bernardo do Campo, Brasil. In (b) taking all the nodes of São Bernardo do Campo, Brasil as origin, the dots are colored as a function of their exponent value and their color is quantified by the color bar in the center. The longest segments without a deceleration point (SWDP) are plotted in black. The probability density function of the $\beta's$ for each experiment is shown on the left of the color scale Figure (c) shows the mean correlation curve between time τ and the distance $\langle D \rangle$. The black traced line represents the exponent equal to 1.0. Figure (d) shows the distribution of SWDP sizes in number of nodes per frequency of occurrence.

São Luís, Brasil

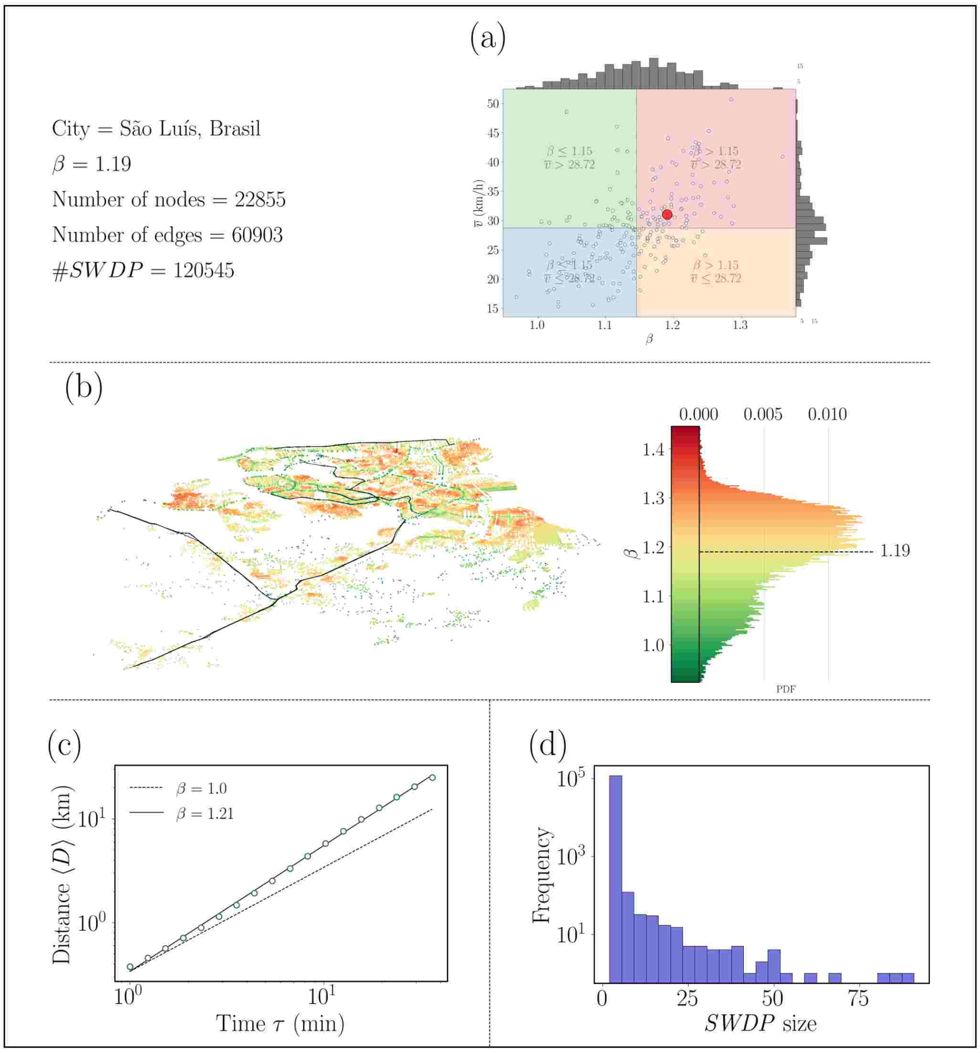

Fig. S177. Methodological sheet - São Luís, Brasil. In (a) Each point represents a city, with mean exponent (β), on the x-axis, and mean speed $\overline{v}$ obtained in all trips made to calculate the exponent on the axis y. The histograms of the values of β and $\overline{v}$ are shown on the axes in the upper and right corners, respectively. The graph was segmented into four quadrants, in which the division is performed by the mean values of β and $\overline{v}$. The quadrants were colored and annotated according to the division criteria. The red dot represents the location of São Luís, Brasil. In (b) taking all the nodes of São Luís, Brasil as origin, the dots are colored as a function of their exponent value and their color is quantified by the color bar in the center. The longest segments without a deceleration point (SWDP) are plotted in black. The probability density function of the $\beta's$ for each experiment is shown on the left of the color scale Figure (c) shows the mean correlation curve between time τ and the distance $\langle D \rangle$. The black traced line represents the exponent equal to 1.0. Figure (d) shows the distribution of SWDP sizes in number of nodes per frequency of occurrence.

São Paulo, Brasil

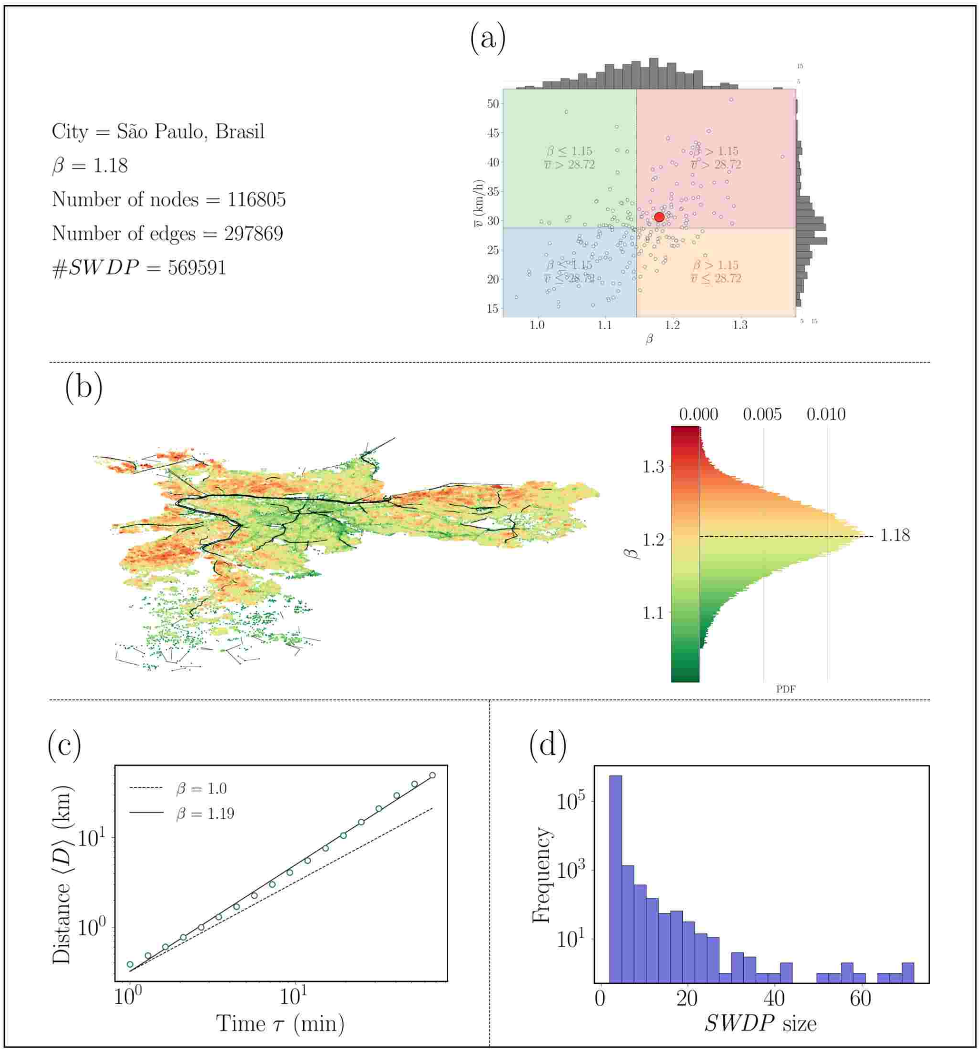

Fig. S178. Methodological sheet - São Paulo, Brasil. In (a) Each point represents a city, with mean exponent (β), on the x-axis, and mean speed $\overline{v}$ obtained in all trips made to calculate the exponent on the axis y. The histograms of the values of β and $\overline{v}$ are shown on the axes in the upper and right corners, respectively. The graph was segmented into four quadrants, in which the division is performed by the mean values of β and $\overline{v}$. The quadrants were colored and annotated according to the division criteria. The red dot represents the location of São Paulo, Brasil. In (b) taking all the nodes of São Paulo, Brasil as origin, the dots are colored as a function of their exponent value and their color is quantified by the color bar in the center. The longest segments without a deceleration point (SWDP) are plotted in black. The probability density function of the $\beta's$ for each experiment is shown on the left of the color scale Figure (c) shows the mean correlation curve between time τ and the distance $\langle D \rangle$. The black traced line represents the exponent equal to 1.0. Figure (d) shows the distribution of SWDP sizes in number of nodes per frequency of occurrence.

Taipei, Taiwan

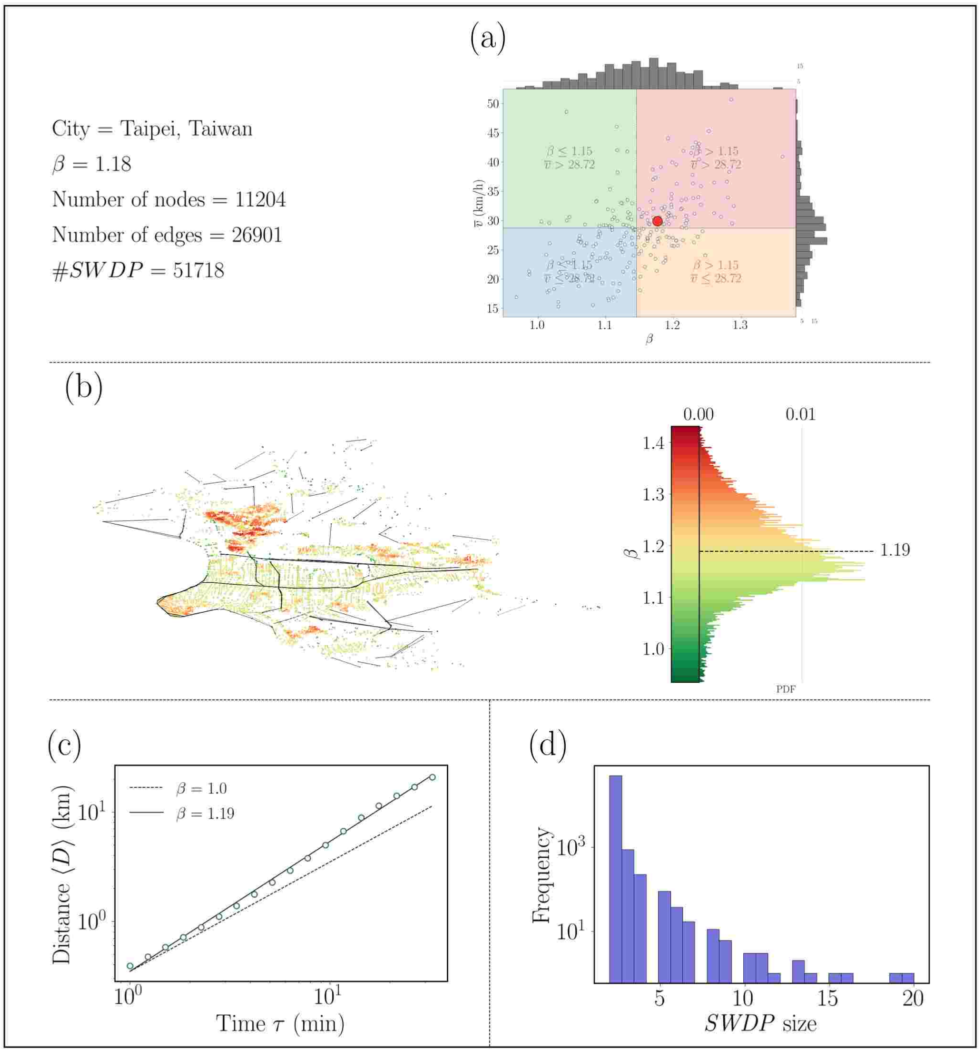

Fig. S179. Methodological sheet - Taipei, Taiwan. In (a) Each point represents a city, with mean exponent (β), on the x-axis, and mean speed $\overline{v}$ obtained in all trips made to calculate the exponent on the axis y. The histograms of the values of β and $\overline{v}$ are shown on the axes in the upper and right corners, respectively. The graph was segmented into four quadrants, in which the division is performed by the mean values of β and $\overline{v}$. The quadrants were colored and annotated according to the division criteria. The red dot represents the location of Taipei, Taiwan. In (b) taking all the nodes of Taipei, Taiwan as origin, the dots are colored as a function of their exponent value and their color is quantified by the color bar in the center. The longest segments without a deceleration point (SWDP) are plotted in black. The probability density function of the $\beta's$ for each experiment is shown on the left of the color scale Figure (c) shows the mean correlation curve between time τ and the distance $\langle D \rangle$. The black traced line represents the exponent equal to 1.0. Figure (d) shows the distribution of SWDP sizes in number of nodes per frequency of occurrence.

Teresina, Brasil

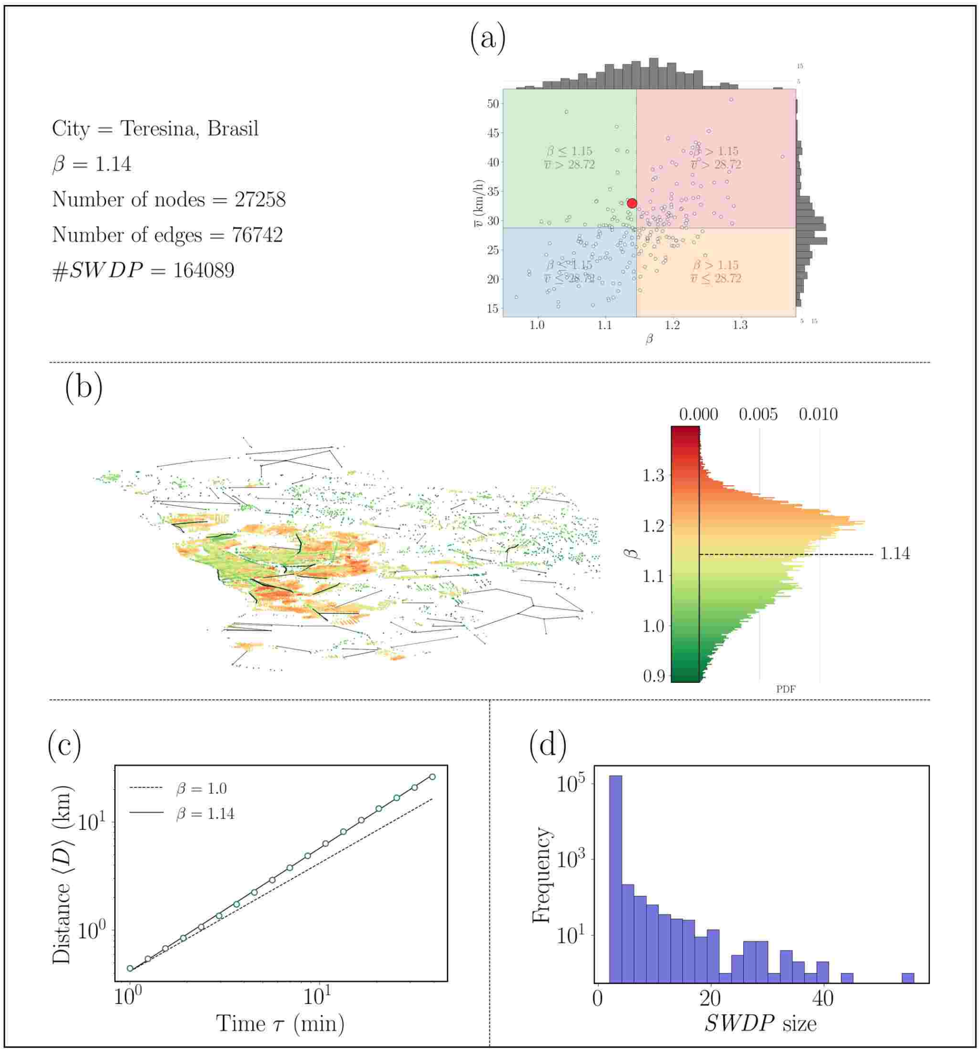

Fig. S180. Methodological sheet - Teresina, Brasil. In (a) Each point represents a city, with mean exponent (β), on the x-axis, and mean speed $\overline{v}$ obtained in all trips made to calculate the exponent on the axis y. The histograms of the values of β and $\overline{v}$ are shown on the axes in the upper and right corners, respectively. The graph was segmented into four quadrants, in which the division is performed by the mean values of β and $\overline{v}$. The quadrants were colored and annotated according to the division criteria. The red dot represents the location of Teresina, Brasil. In (b) taking all the nodes of Teresina, Brasil as origin, the dots are colored as a function of their exponent value and their color is quantified by the color bar in the center. The longest segments without a deceleration point (SWDP) are plotted in black. The probability density function of the $\beta's$ for each experiment is shown on the left of the color scale Figure (c) shows the mean correlation curve between time τ and the distance $\langle D \rangle$. The black traced line represents the exponent equal to 1.0. Figure (d) shows the distribution of SWDP sizes in number of nodes per frequency of occurrence.

The Hague, Netherlands

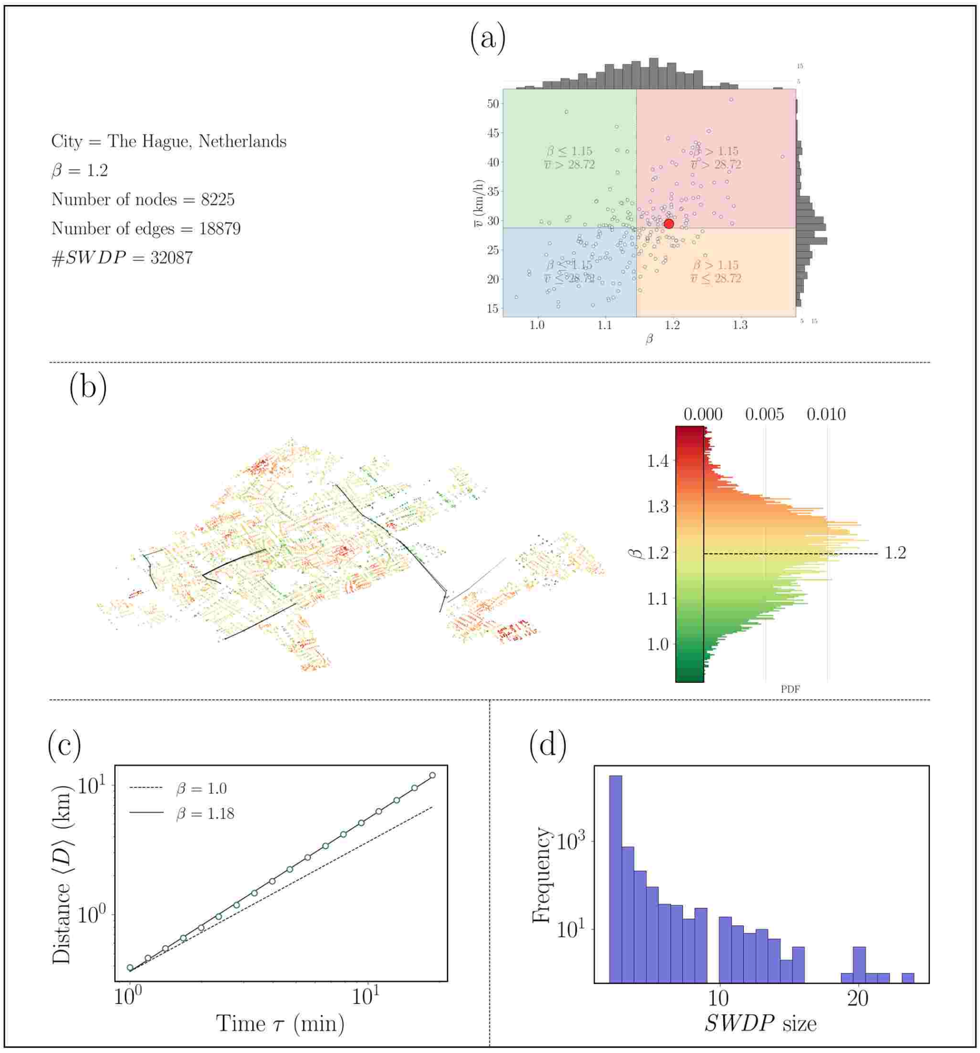

Fig. S181. Methodological sheet - The Hague, Netherlands. In (a) Each point represents a city, with mean exponent (β), on the x-axis, and mean speed $\overline{v}$ obtained in all trips made to calculate the exponent on the axis y. The histograms of the values of β and $\overline{v}$ are shown on the axes in the upper and right corners, respectively. The graph was segmented into four quadrants, in which the division is performed by the mean values of β and $\overline{v}$. The quadrants were colored and annotated according to the division criteria. The red dot represents the location of The Hague, Netherlands. In (b) taking all the nodes of The Hague, Netherlands as origin, the dots are colored as a function of their exponent value and their color is quantified by the color bar in the center. The longest segments without a deceleration point (SWDP) are plotted in black. The probability density function of the $\beta's$ for each experiment is shown on the left of the color scale Figure (c) shows the mean correlation curve between time τ and the distance $\langle D \rangle$. The black traced line represents the exponent equal to 1.0. Figure (d) shows the distribution of SWDP sizes in number of nodes per frequency of occurrence.

Tirana, Albania

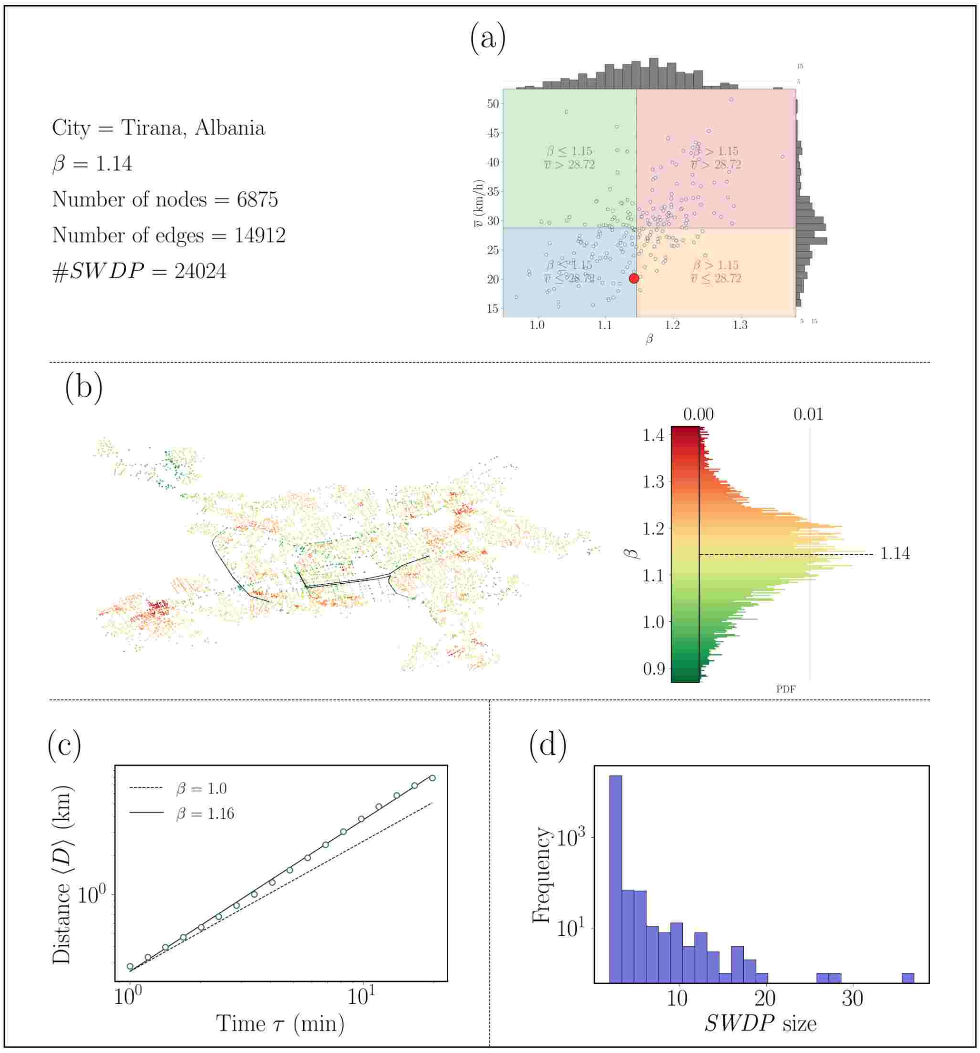

Fig. S182. Methodological sheet - Tirana, Albania. In (a) Each point represents a city, with mean exponent (β), on the x-axis, and mean speed $\overline{v}$ obtained in all trips made to calculate the exponent on the axis y. The histograms of the values of β and $\overline{v}$ are shown on the axes in the upper and right corners, respectively. The graph was segmented into four quadrants, in which the division is performed by the mean values of β and $\overline{v}$. The quadrants were colored and annotated according to the division criteria. The red dot represents the location of Tirana, Albania. In (b) taking all the nodes of Tirana, Albania as origin, the dots are colored as a function of their exponent value and their color is quantified by the color bar in the center. The longest segments without a deceleration point (SWDP) are plotted in black. The probability density function of the $\beta's$ for each experiment is shown on the left of the color scale Figure (c) shows the mean correlation curve between time τ and the distance $\langle D \rangle$. The black traced line represents the exponent equal to 1.0. Figure (d) shows the distribution of SWDP sizes in number of nodes per frequency of occurrence.

Toronto, Canadá

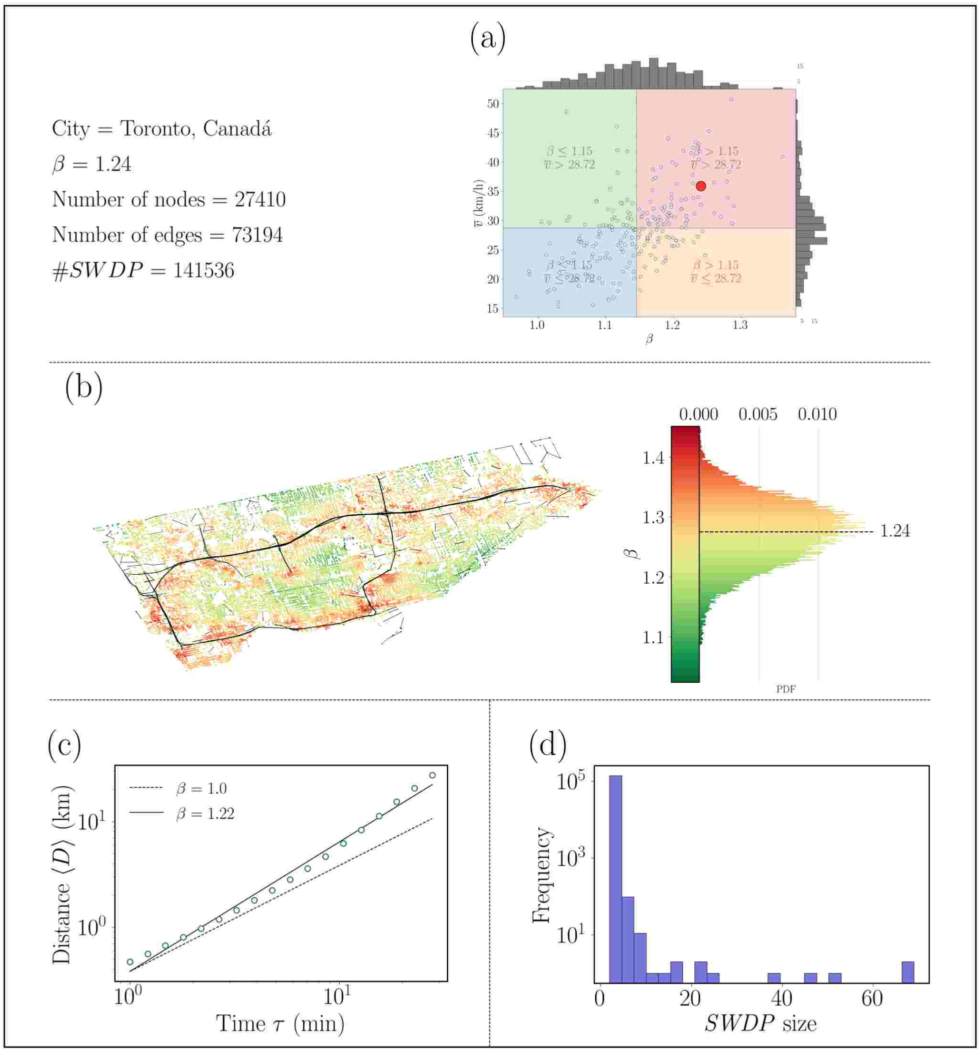

Fig. S183. Methodological sheet - Toronto, Canadá. In (a) Each point represents a city, with mean exponent (β), on the x-axis, and mean speed $\overline{v}$ obtained in all trips made to calculate the exponent on the axis y. The histograms of the values of β and $\overline{v}$ are shown on the axes in the upper and right corners, respectively. The graph was segmented into four quadrants, in which the division is performed by the mean values of β and $\overline{v}$. The quadrants were colored and annotated according to the division criteria. The red dot represents the location of Toronto, Canadá. In (b) taking all the nodes of Toronto, Canadá as origin, the dots are colored as a function of their exponent value and their color is quantified by the color bar in the center. The longest segments without a deceleration point (SWDP) are plotted in black. The probability density function of the $\beta's$ for each experiment is shown on the left of the color scale Figure (c) shows the mean correlation curve between time τ and the distance $\langle D \rangle$. The black traced line represents the exponent equal to 1.0. Figure (d) shows the distribution of SWDP sizes in number of nodes per frequency of occurrence.

Tripoli, Libya

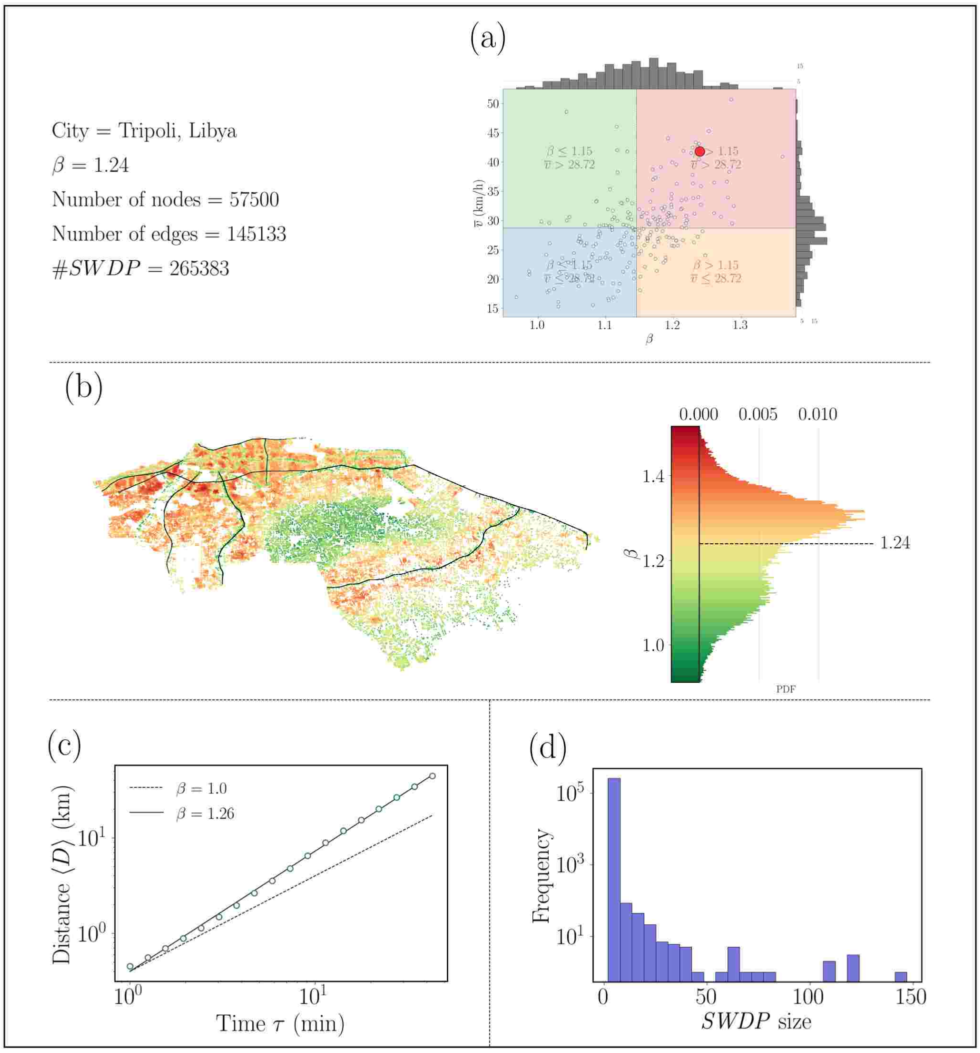

Fig. S184. Methodological sheet - Tripoli, Libya. In (a) Each point represents a city, with mean exponent (β), on the x-axis, and mean speed $\overline{v}$ obtained in all trips made to calculate the exponent on the axis y. The histograms of the values of β and $\overline{v}$ are shown on the axes in the upper and right corners, respectively. The graph was segmented into four quadrants, in which the division is performed by the mean values of β and $\overline{v}$. The quadrants were colored and annotated according to the division criteria. The red dot represents the location of Tripoli, Libya. In (b) taking all the nodes of Tripoli, Libya as origin, the dots are colored as a function of their exponent value and their color is quantified by the color bar in the center. The longest segments without a deceleration point (SWDP) are plotted in black. The probability density function of the $\beta's$ for each experiment is shown on the left of the color scale Figure (c) shows the mean correlation curve between time τ and the distance $\langle D \rangle$. The black traced line represents the exponent equal to 1.0. Figure (d) shows the distribution of SWDP sizes in number of nodes per frequency of occurrence.

Trondheim, Trøndelag, Norway

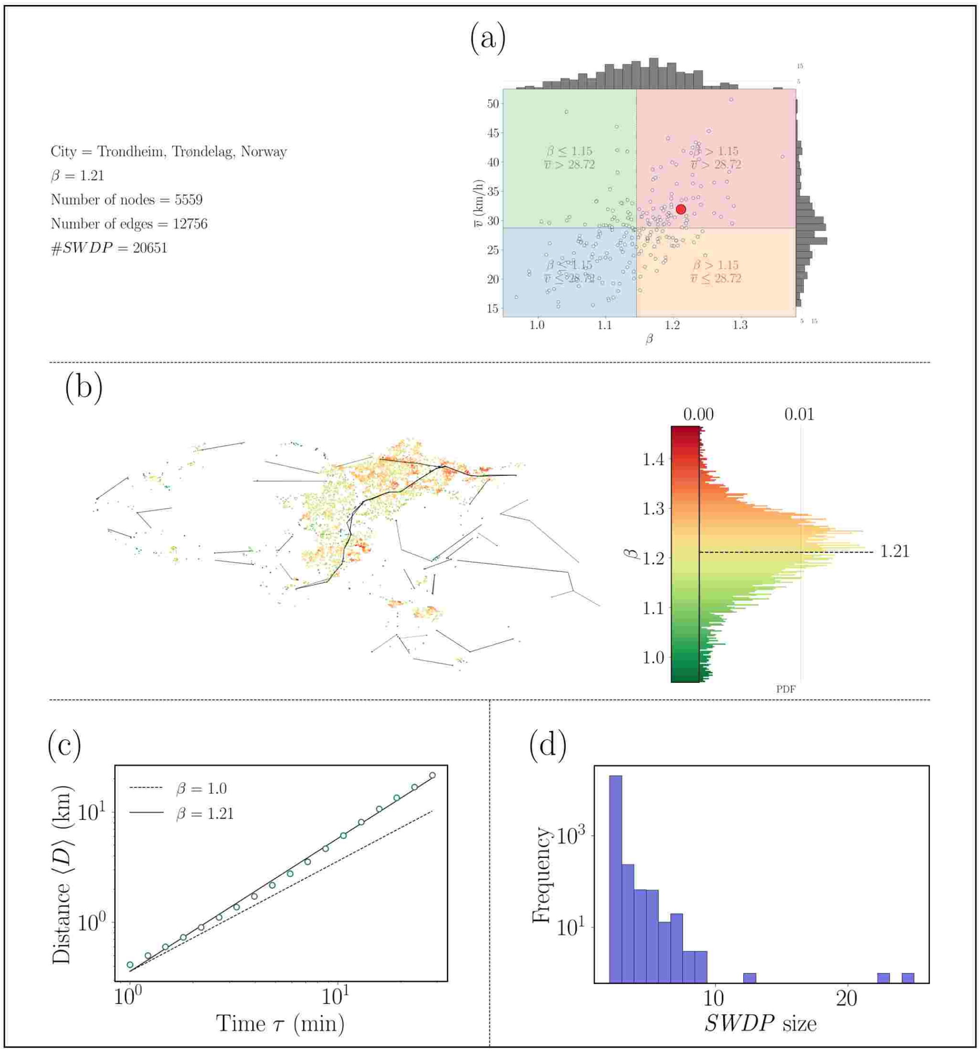

Fig. S185. Methodological sheet - Trondheim, Trøndelag, Norway. In (a) Each point represents a city, with mean exponent (β), on the x-axis, and mean speed $\overline{v}$ obtained in all trips made to calculate the exponent on the axis y. The histograms of the values of β and $\overline{v}$ are shown on the axes in the upper and right corners, respectively. The graph was segmented into four quadrants, in which the division is performed by the mean values of β and $\overline{v}$. The quadrants were colored and annotated according to the division criteria. The red dot represents the location of Trondheim, Trøndelag, Norway. In (b) taking all the nodes of Trondheim, Trøndelag, Norway as origin, the dots are colored as a function of their exponent value and their color is quantified by the color bar in the center. The longest segments without a deceleration point (SWDP) are plotted in black. The probability density function of the $\beta's$ for each experiment is shown on the left of the color scale Figure (c) shows the mean correlation curve between time τ and the distance $\langle D \rangle$. The black traced line represents the exponent equal to 1.0. Figure (d) shows the distribution of SWDP sizes in number of nodes per frequency of occurrence.

Tulsa, USA

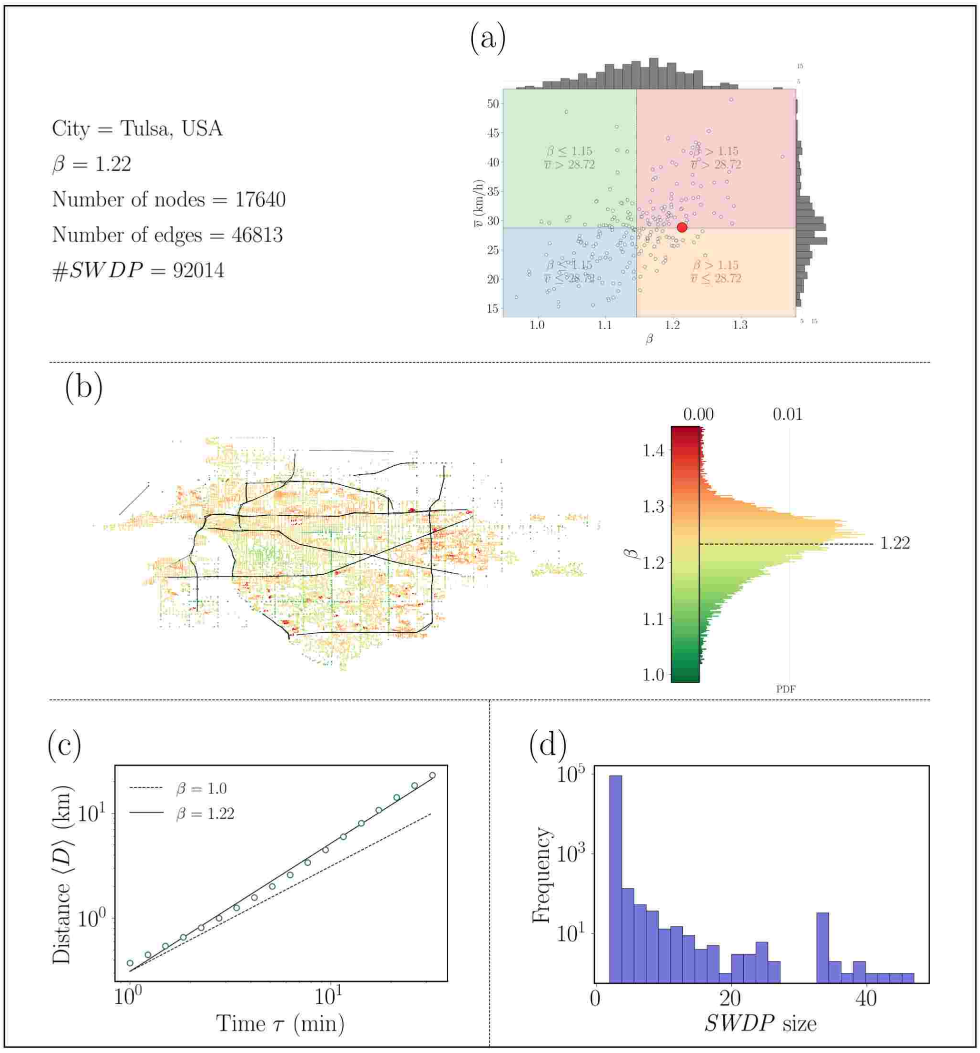

Fig. S186. Methodological sheet - Tulsa, USA. In (a) Each point represents a city, with mean exponent (β), on the x-axis, and mean speed $\overline{v}$ obtained in all trips made to calculate the exponent on the axis y. The histograms of the values of β and $\overline{v}$ are shown on the axes in the upper and right corners, respectively. The graph was segmented into four quadrants, in which the division is performed by the mean values of β and $\overline{v}$. The quadrants were colored and annotated according to the division criteria. The red dot represents the location of Tulsa, USA. In (b) taking all the nodes of Tulsa, USA as origin, the dots are colored as a function of their exponent value and their color is quantified by the color bar in the center. The longest segments without a deceleration point (SWDP) are plotted in black. The probability density function of the $\beta's$ for each experiment is shown on the left of the color scale Figure (c) shows the mean correlation curve between time τ and the distance $\langle D \rangle$. The black traced line represents the exponent equal to 1.0. Figure (d) shows the distribution of SWDP sizes in number of nodes per frequency of occurrence.

Turin, Italy

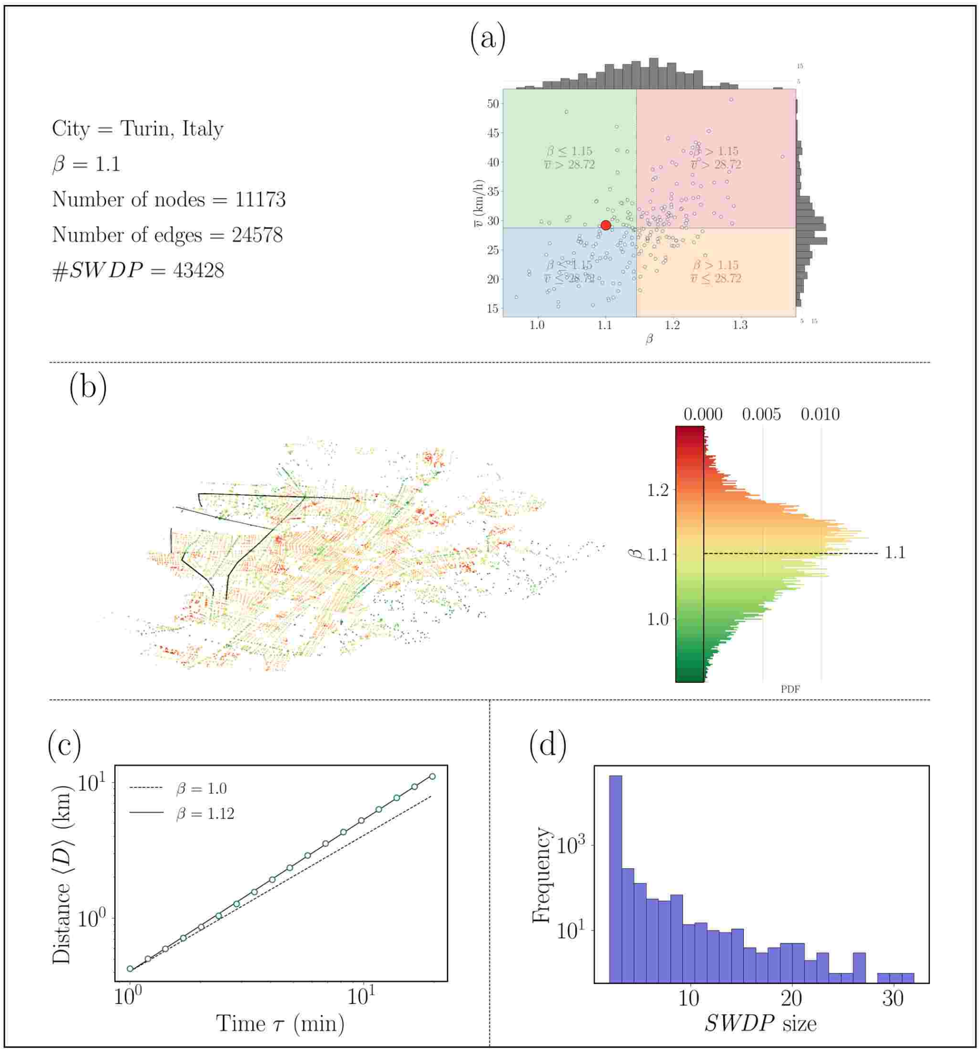

Fig. S187. Methodological sheet - Turin, Italy. In (a) Each point represents a city, with mean exponent (β), on the x-axis, and mean speed $\overline{v}$ obtained in all trips made to calculate the exponent on the axis y. The histograms of the values of β and $\overline{v}$ are shown on the axes in the upper and right corners, respectively. The graph was segmented into four quadrants, in which the division is performed by the mean values of β and $\overline{v}$. The quadrants were colored and annotated according to the division criteria. The red dot represents the location of Turin, Italy. In (b) taking all the nodes of Turin, Italy as origin, the dots are colored as a function of their exponent value and their color is quantified by the color bar in the center. The longest segments without a deceleration point (SWDP) are plotted in black. The probability density function of the $\beta's$ for each experiment is shown on the left of the color scale Figure (c) shows the mean correlation curve between time τ and the distance $\langle D \rangle$. The black traced line represents the exponent equal to 1.0. Figure (d) shows the distribution of SWDP sizes in number of nodes per frequency of occurrence.

Turku, Finland

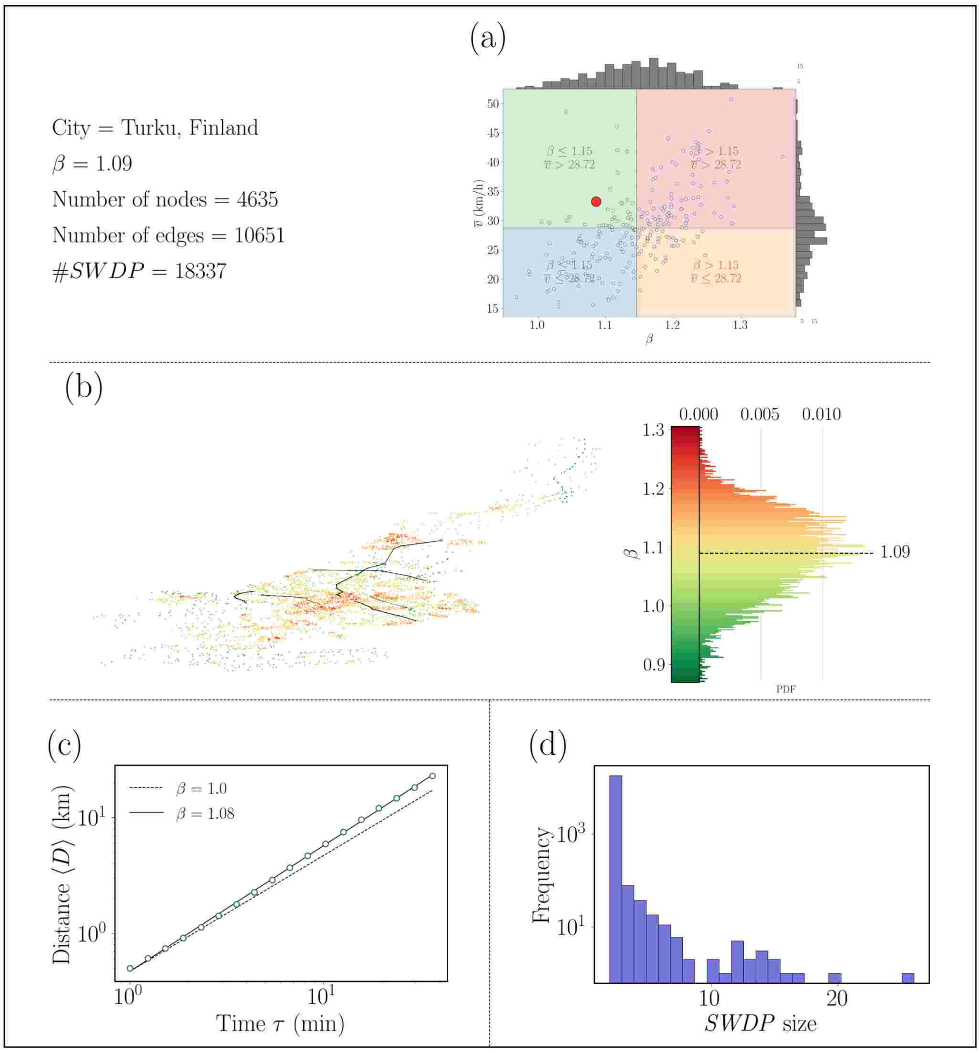

Fig. S188. Methodological sheet - Turku, Finland. In (a) Each point represents a city, with mean exponent (β), on the x-axis, and mean speed $\overline{v}$ obtained in all trips made to calculate the exponent on the axis y. The histograms of the values of β and $\overline{v}$ are shown on the axes in the upper and right corners, respectively. The graph was segmented into four quadrants, in which the division is performed by the mean values of β and $\overline{v}$. The quadrants were colored and annotated according to the division criteria. The red dot represents the location of Turku, Finland. In (b) taking all the nodes of Turku, Finland as origin, the dots are colored as a function of their exponent value and their color is quantified by the color bar in the center. The longest segments without a deceleration point (SWDP) are plotted in black. The probability density function of the $\beta's$ for each experiment is shown on the left of the color scale Figure (c) shows the mean correlation curve between time τ and the distance $\langle D \rangle$. The black traced line represents the exponent equal to 1.0. Figure (d) shows the distribution of SWDP sizes in number of nodes per frequency of occurrence.

Ulaanbaatar, Mongolia

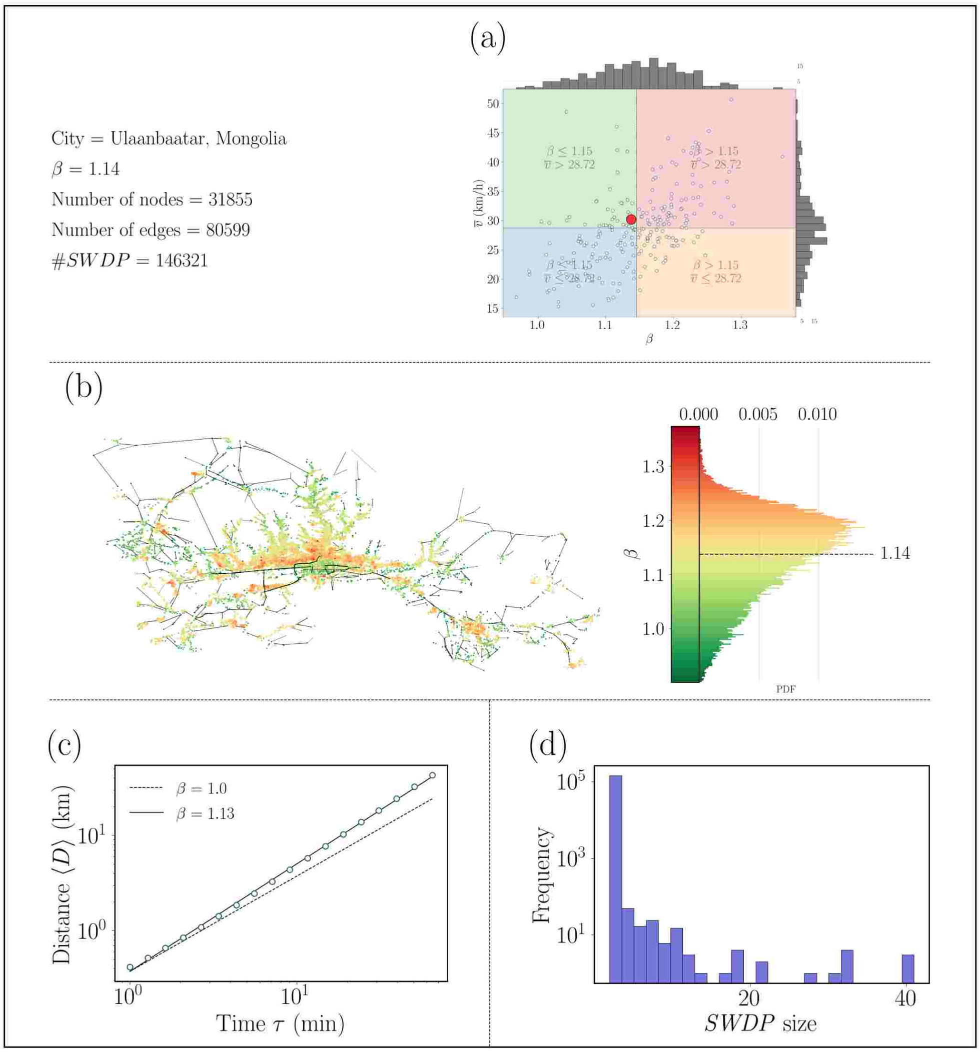

Fig. S189. Methodological sheet - Ulaanbaatar, Mongolia. In (a) Each point represents a city, with mean exponent (β), on the x-axis, and mean speed $\overline{v}$ obtained in all trips made to calculate the exponent on the axis y. The histograms of the values of β and $\overline{v}$ are shown on the axes in the upper and right corners, respectively. The graph was segmented into four quadrants, in which the division is performed by the mean values of β and $\overline{v}$. The quadrants were colored and annotated according to the division criteria. The red dot represents the location of Ulaanbaatar, Mongolia. In (b) taking all the nodes of Ulaanbaatar, Mongolia as origin, the dots are colored as a function of their exponent value and their color is quantified by the color bar in the center. The longest segments without a deceleration point (SWDP) are plotted in black. The probability density function of the $\beta's$ for each experiment is shown on the left of the color scale Figure (c) shows the mean correlation curve between time τ and the distance $\langle D \rangle$. The black traced line represents the exponent equal to 1.0. Figure (d) shows the distribution of SWDP sizes in number of nodes per frequency of occurrence.

Umea, Sweden

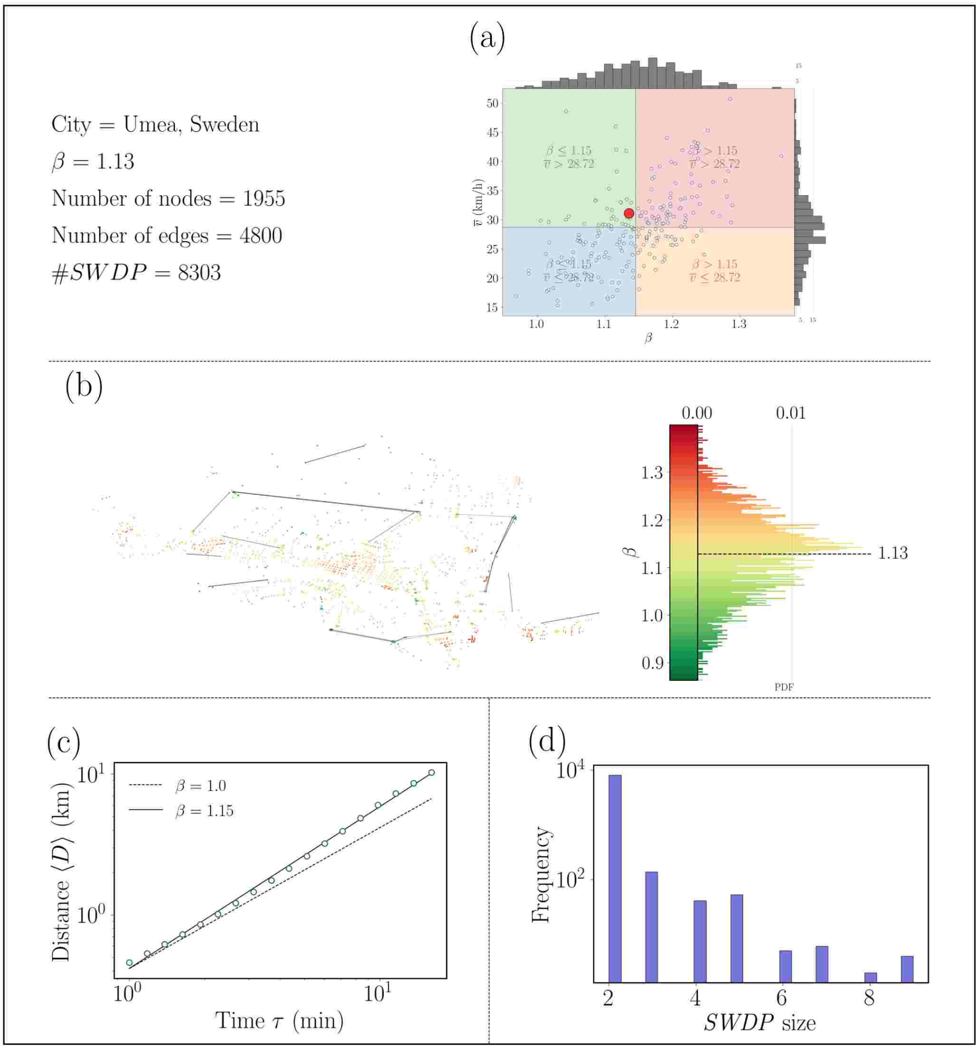

Fig. S190. Methodological sheet - Umea, Sweden. In (a) Each point represents a city, with mean exponent (β), on the x-axis, and mean speed $\overline{v}$ obtained in all trips made to calculate the exponent on the axis y. The histograms of the values of β and $\overline{v}$ are shown on the axes in the upper and right corners, respectively. The graph was segmented into four quadrants, in which the division is performed by the mean values of β and $\overline{v}$. The quadrants were colored and annotated according to the division criteria. The red dot represents the location of Umea, Sweden. In (b) taking all the nodes of Umea, Sweden as origin, the dots are colored as a function of their exponent value and their color is quantified by the color bar in the center. The longest segments without a deceleration point (SWDP) are plotted in black. The probability density function of the $\beta' s$ for each experiment is shown on the left of the color scale Figure (c) shows the mean correlation curve between time τ and the distance $\langle D \rangle$. The black traced line represents the exponent equal to 1.0. Figure (d) shows the distribution of SWDP sizes in number of nodes per frequency of occurrence.

Utrecht, Netherlands

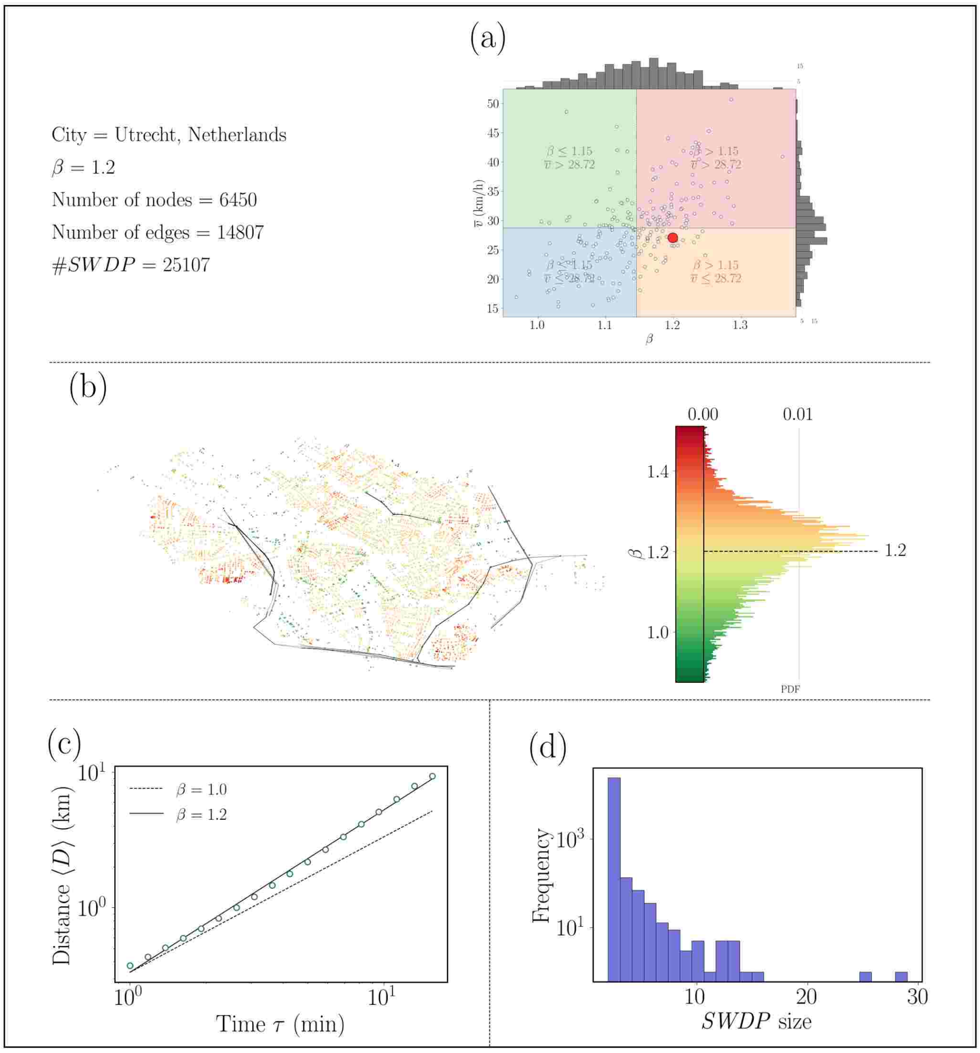

Fig. S191. Methodological sheet - Utrecht, Netherlands. In (a) Each point represents a city, with mean exponent (β), on the x-axis, and mean speed $\overline{v}$ obtained in all trips made to calculate the exponent on the axis y. The histograms of the values of β and $\overline{v}$ are shown on the axes in the upper and right corners, respectively. The graph was segmented into four quadrants, in which the division is performed by the mean values of β and $\overline{v}$. The quadrants were colored and annotated according to the division criteria. The red dot represents the location of Utrecht, Netherlands. In (b) taking all the nodes of Utrecht, Netherlands as origin, the dots are colored as a function of their exponent value and their color is quantified by the color bar in the center. The longest segments without a deceleration point (SWDP) are plotted in black. The probability density function of the $\beta's$ for each experiment is shown on the left of the color scale Figure (c) shows the mean correlation curve between time τ and the distance $\langle D \rangle$. The black traced line represents the exponent equal to 1.0. Figure (d) shows the distribution of SWDP sizes in number of nodes per frequency of occurrence.

Valencia, Spain

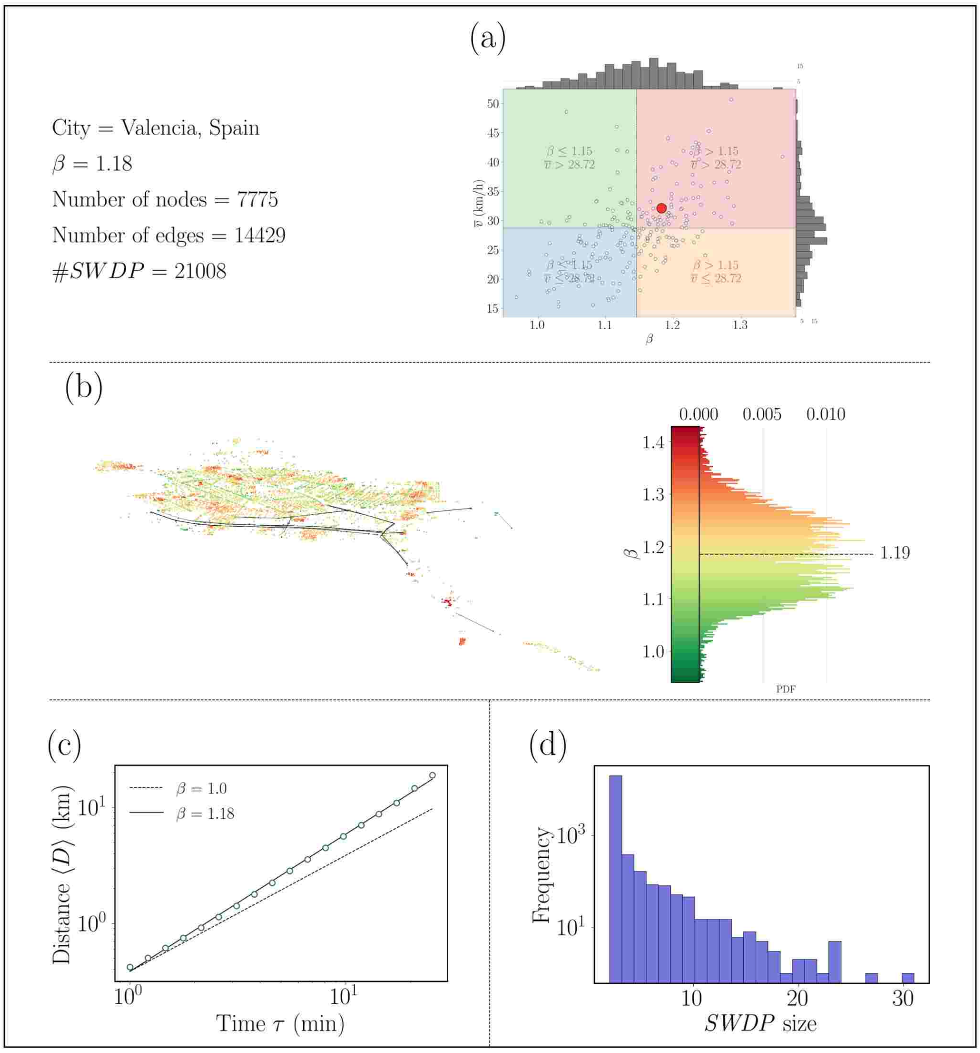

Fig. S192. Methodological sheet - Valencia, Spain. In (a) Each point represents a city, with mean exponent (β), on the x-axis, and mean speed $\overline{v}$ obtained in all trips made to calculate the exponent on the axis y. The histograms of the values of β and $\overline{v}$ are shown on the axes in the upper and right corners, respectively. The graph was segmented into four quadrants, in which the division is performed by the mean values of β and $\overline{v}$. The quadrants were colored and annotated according to the division criteria. The red dot represents the location of Valencia, Spain. In (b) taking all the nodes of Valencia, Spain as origin, the dots are colored as a function of their exponent value and their color is quantified by the color bar in the center. The longest segments without a deceleration point (SWDP) are plotted in black. The probability density function of the $\beta's$ for each experiment is shown on the left of the color scale Figure (c) shows the mean correlation curve between time τ and the distance $\langle D \rangle$. The black traced line represents the exponent equal to 1.0. Figure (d) shows the distribution of SWDP sizes in number of nodes per frequency of occurrence.

Valparaíso, Chile

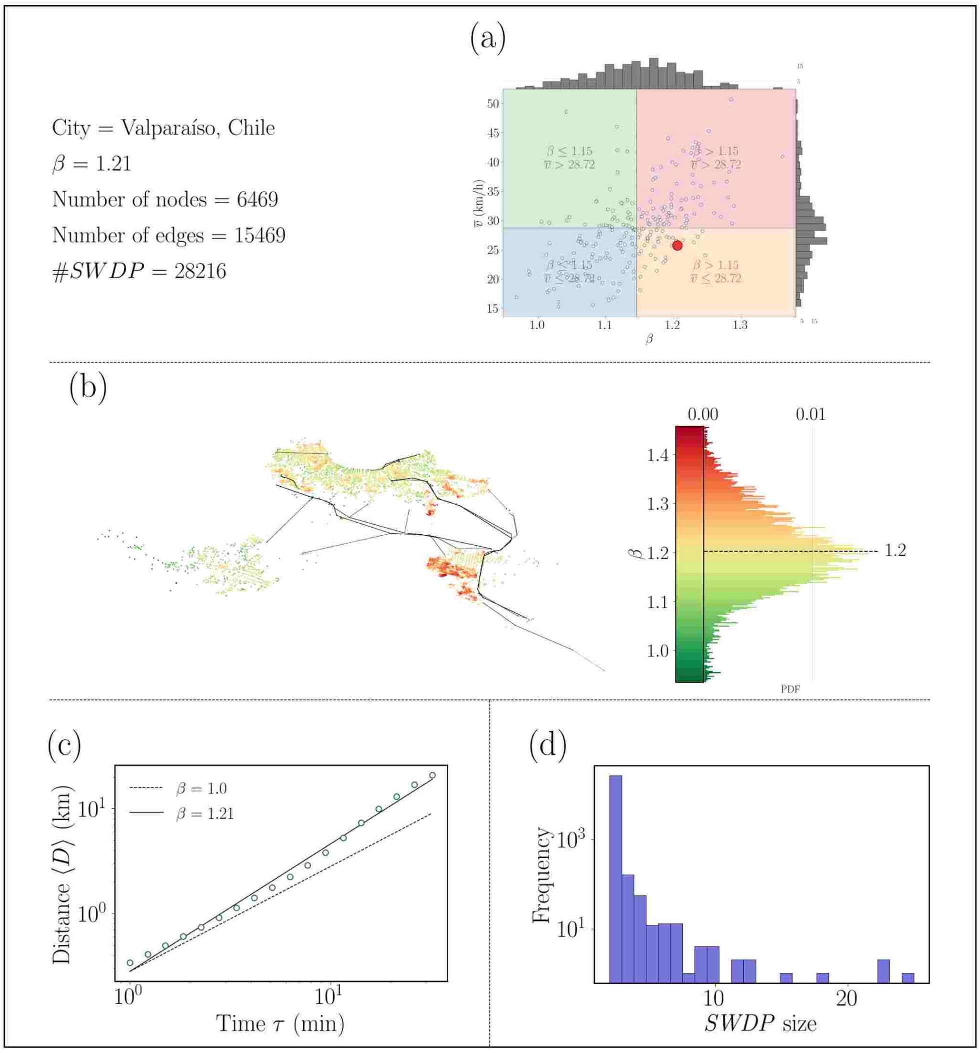

Fig. S193. Methodological sheet - Valparaíso, Chile. In (a) Each point represents a city, with mean exponent (β), on the x-axis, and mean speed $\overline{v}$ obtained in all trips made to calculate the exponent on the axis y. The histograms of the values of β and $\overline{v}$ are shown on the axes in the upper and right corners, respectively. The graph was segmented into four quadrants, in which the division is performed by the mean values of β and $\overline{v}$. The quadrants were colored and annotated according to the division criteria. The red dot represents the location of Valparaíso, Chile. In (b) taking all the nodes of Valparaíso, Chile as origin, the dots are colored as a function of their exponent value and their color is quantified by the color bar in the center. The longest segments without a deceleration point (SWDP) are plotted in black. The probability density function of the $\beta' s$ for each experiment is shown on the left of the color scale Figure (c) shows the mean correlation curve between time τ and the distance $\langle D \rangle$. The black traced line represents the exponent equal to 1.0. Figure (d) shows the distribution of SWDP sizes in number of nodes per frequency of occurrence.

Vancouver, Canadá

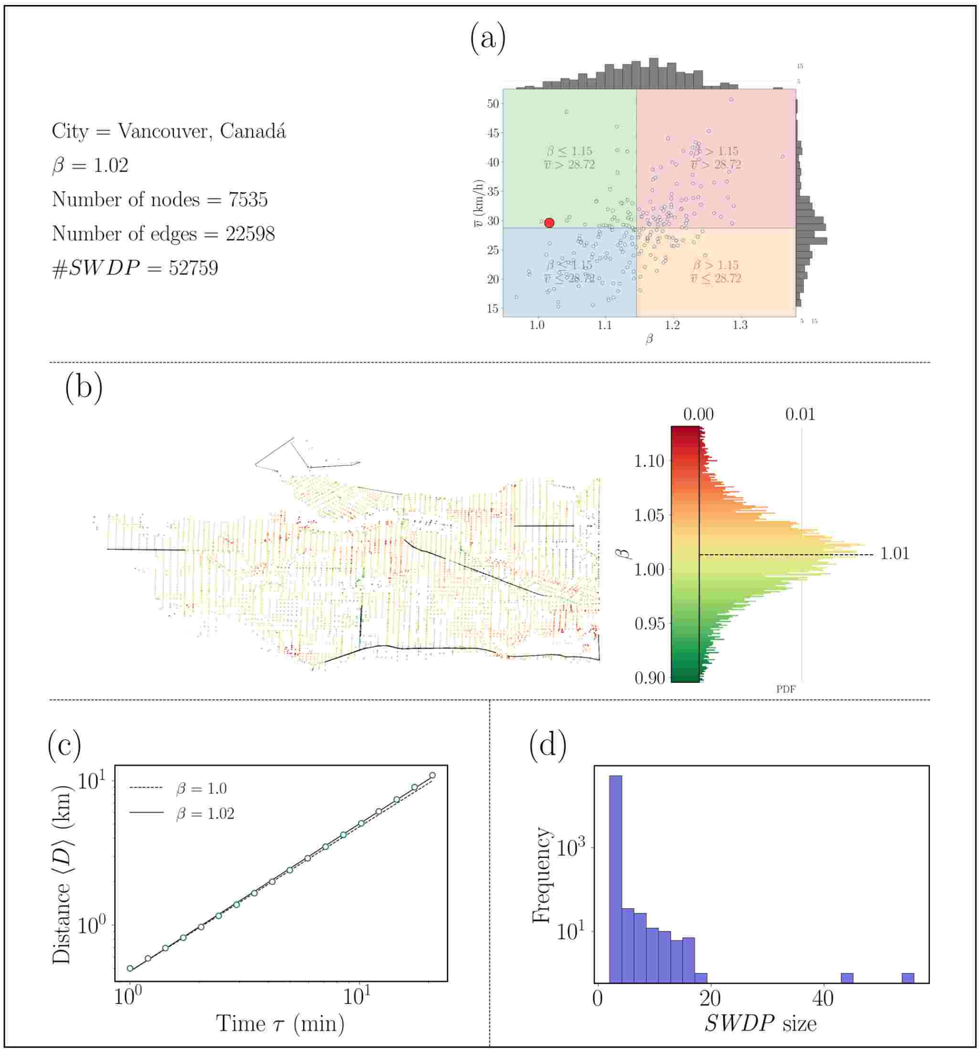

Fig. S194. Methodological sheet - Vancouver, Canadá. In (a) Each point represents a city, with mean exponent (β), on the x-axis, and mean speed $\overline{v}$ obtained in all trips made to calculate the exponent on the axis y. The histograms of the values of β and $\overline{v}$ are shown on the axes in the upper and right corners, respectively. The graph was segmented into four quadrants, in which the division is performed by the mean values of β and $\overline{v}$. The quadrants were colored and annotated according to the division criteria. The red dot represents the location of Vancouver, Canadá. In (b) taking all the nodes of Vancouver, Canadá as origin, the dots are colored as a function of their exponent value and their color is quantified by the color bar in the center. The longest segments without a deceleration point (SWDP) are plotted in black. The probability density function of the $\beta's$ for each experiment is shown on the left of the color scale Figure (c) shows the mean correlation curve between time τ and the distance $\langle D \rangle$. The black traced line represents the exponent equal to 1.0. Figure (d) shows the distribution of SWDP sizes in number of nodes per frequency of occurrence.

Venice, Italy

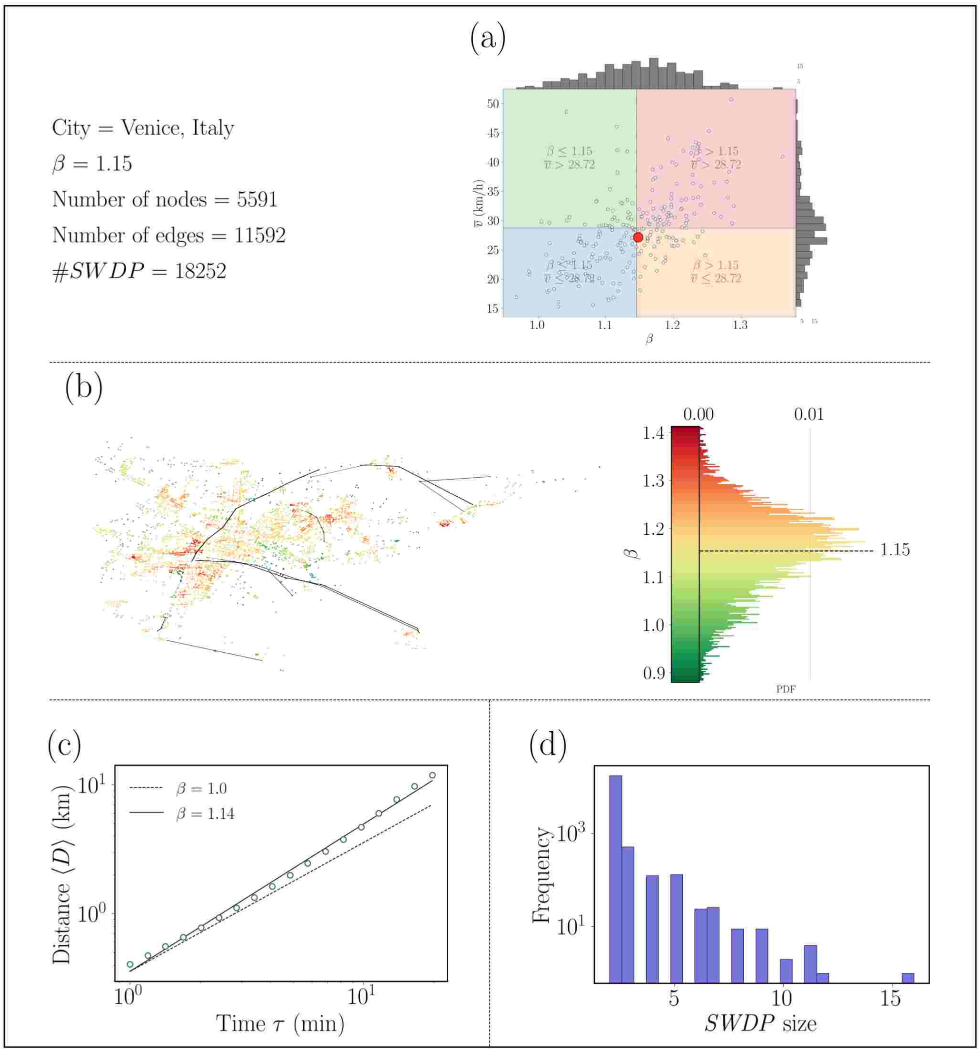

Fig. S195. Methodological sheet - Venice, Italy. In (a) Each point represents a city, with mean exponent (β), on the x-axis, and mean speed $\overline{v}$ obtained in all trips made to calculate the exponent on the axis y. The histograms of the values of β and $\overline{v}$ are shown on the axes in the upper and right corners, respectively. The graph was segmented into four quadrants, in which the division is performed by the mean values of β and $\overline{v}$. The quadrants were colored and annotated according to the division criteria. The red dot represents the location of Venice, Italy. In (b) taking all the nodes of Venice, Italy as origin, the dots are colored as a function of their exponent value and their color is quantified by the color bar in the center. The longest segments without a deceleration point (SWDP) are plotted in black. The probability density function of the $\beta's$ for each experiment is shown on the left of the color scale Figure (c) shows the mean correlation curve between time τ and the distance $\langle D \rangle$. The black traced line represents the exponent equal to 1.0. Figure (d) shows the distribution of SWDP sizes in number of nodes per frequency of occurrence.

Vienna, Austria

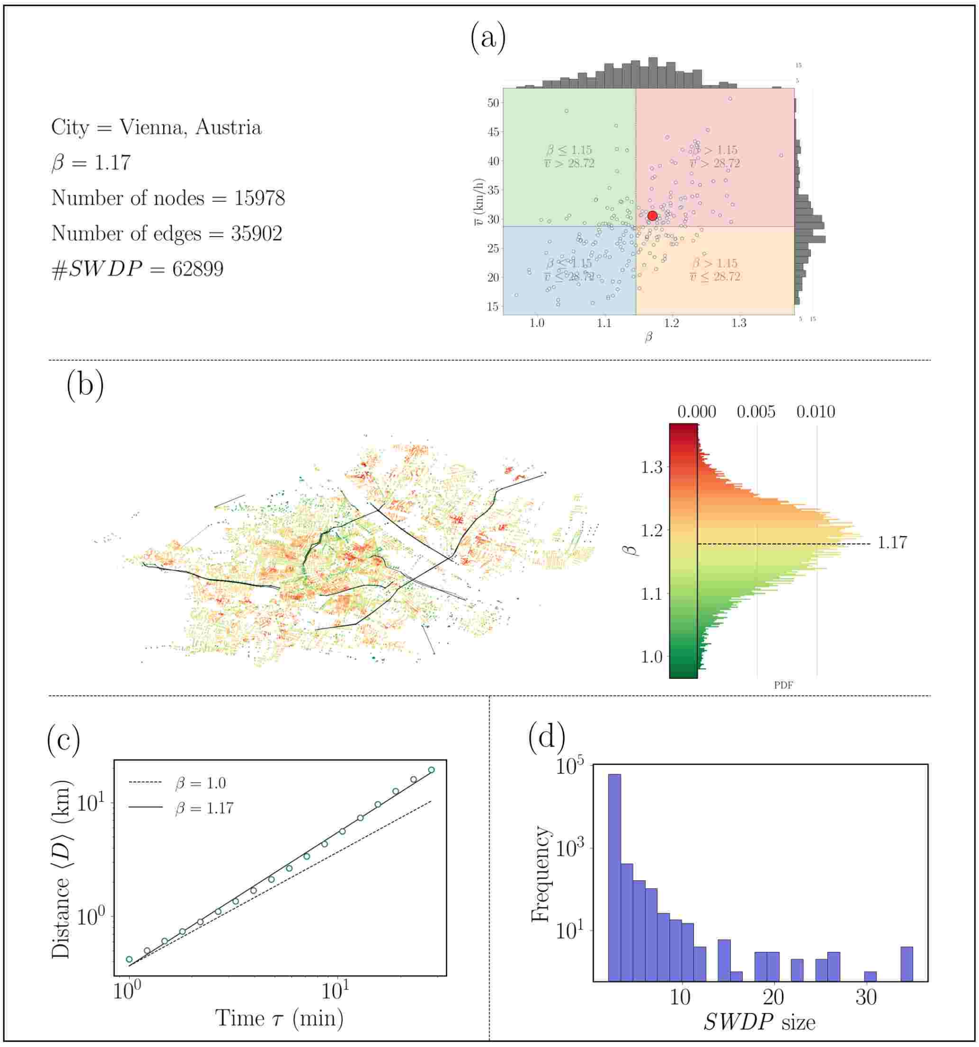

Fig. S196. Methodological sheet - Vienna, Austria. In (a) Each point represents a city, with mean exponent (β), on the x-axis, and mean speed $\overline{v}$ obtained in all trips made to calculate the exponent on the axis y. The histograms of the values of β and $\overline{v}$ are shown on the axes in the upper and right corners, respectively. The graph was segmented into four quadrants, in which the division is performed by the mean values of β and $\overline{v}$. The quadrants were colored and annotated according to the division criteria. The red dot represents the location of Vienna, Austria. In (b) taking all the nodes of Vienna, Austria as origin, the dots are colored as a function of their exponent value and their color is quantified by the color bar in the center. The longest segments without a deceleration point (SWDP) are plotted in black. The probability density function of the $\beta's$ for each experiment is shown on the left of the color scale Figure (c) shows the mean correlation curve between time τ and the distance $\langle D \rangle$. The black traced line represents the exponent equal to 1.0. Figure (d) shows the distribution of SWDP sizes in number of nodes per frequency of occurrence.

Viña del Mar, Chile

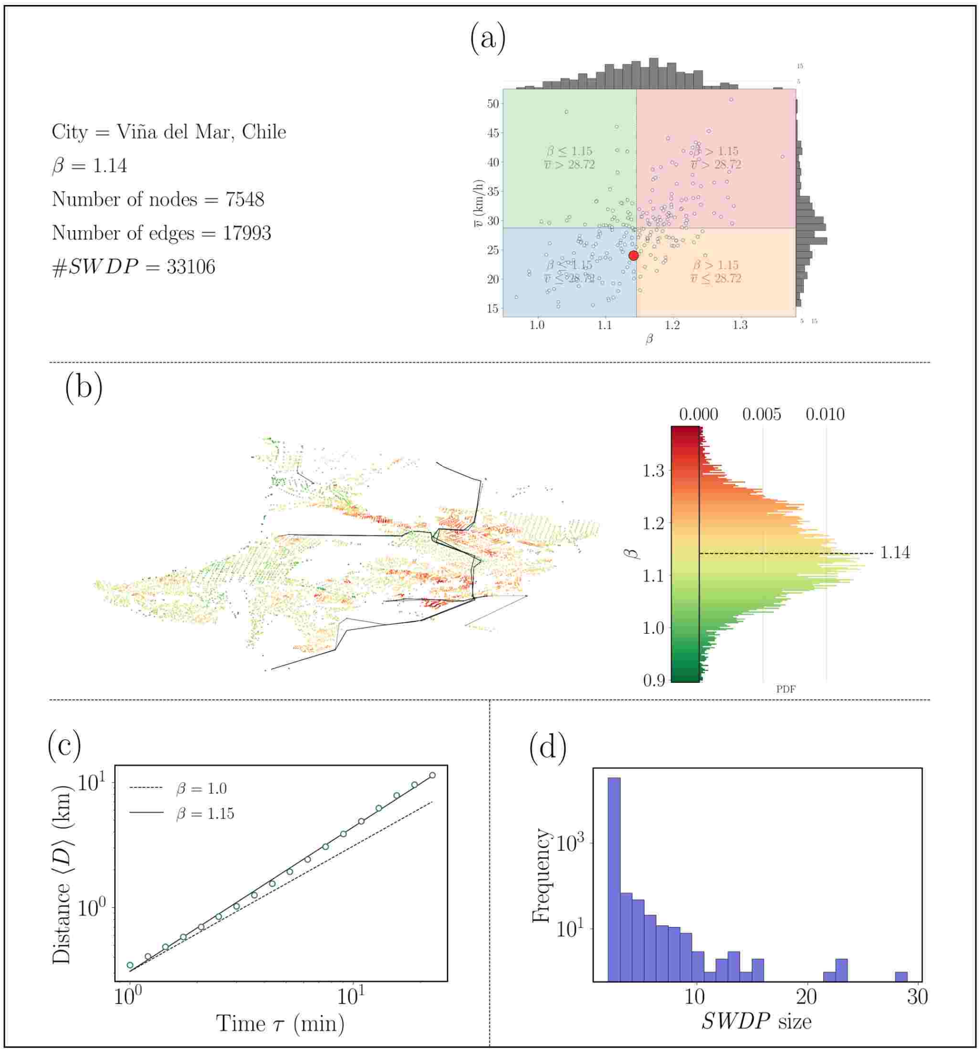

Fig. S197. Methodological sheet - Viña del Mar, Chile. In (a) Each point represents a city, with mean exponent (β), on the x-axis, and mean speed $\overline{v}$ obtained in all trips made to calculate the exponent on the axis y. The histograms of the values of β and $\overline{v}$ are shown on the axes in the upper and right corners, respectively. The graph was segmented into four quadrants, in which the division is performed by the mean values of β and $\overline{v}$. The quadrants were colored and annotated according to the division criteria. The red dot represents the location of Viña del Mar, Chile. In (b) taking all the nodes of Viña del Mar, Chile as origin, the dots are colored as a function of their exponent value and their color is quantified by the color bar in the center. The longest segments without a deceleration point (SWDP) are plotted in black. The probability density function of the $\beta's$ for each experiment is shown on the left of the color scale Figure (c) shows the mean correlation curve between time τ and the distance $\langle D \rangle$. The black traced line represents the exponent equal to 1.0. Figure (d) shows the distribution of SWDP sizes in number of nodes per frequency of occurrence.

Washington, USA

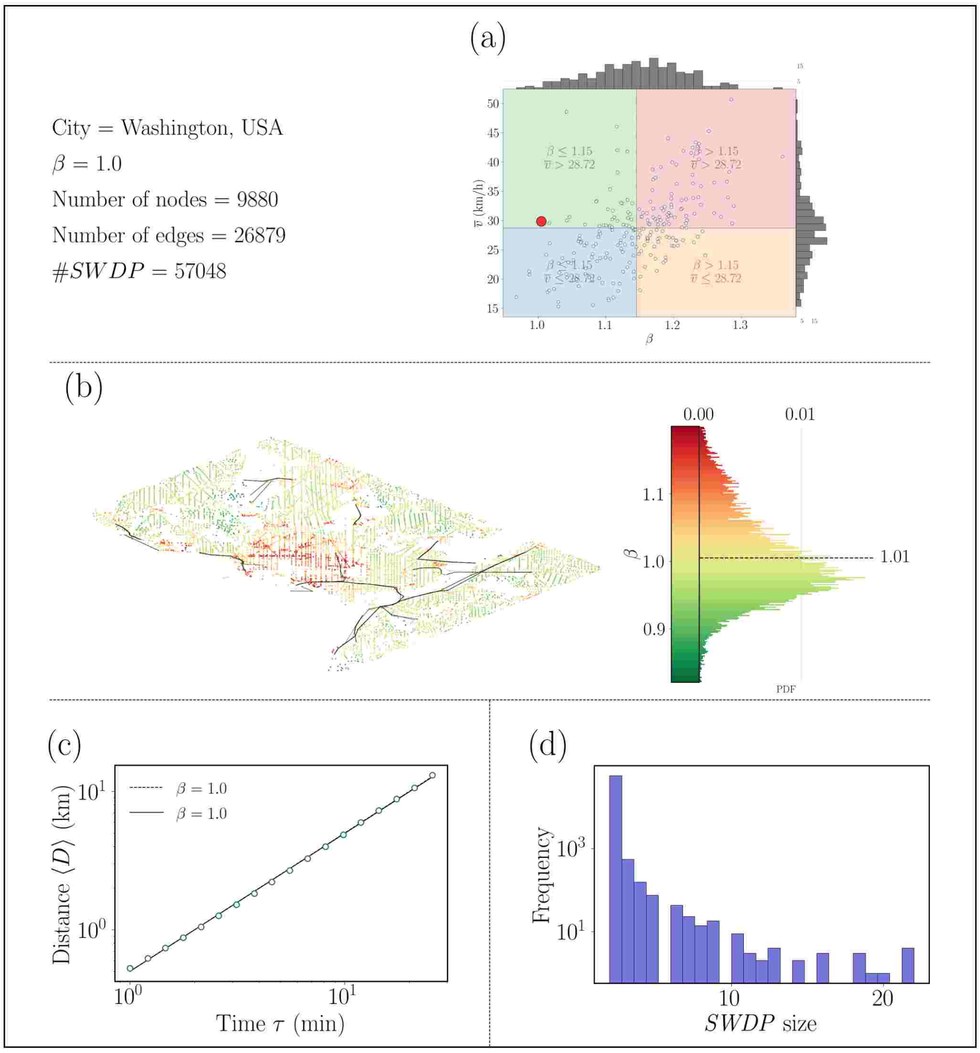

Fig. S198. Methodological sheet - Washington, USA. In (a) Each point represents a city, with mean exponent (β), on the x-axis, and mean speed $\overline{v}$ obtained in all trips made to calculate the exponent on the axis y. The histograms of the values of β and $\overline{v}$ are shown on the axes in the upper and right corners, respectively. The graph was segmented into four quadrants, in which the division is performed by the mean values of β and $\overline{v}$. The quadrants were colored and annotated according to the division criteria. The red dot represents the location of Washington, USA. In (b) taking all the nodes of Washington, USA as origin, the dots are colored as a function of their exponent value and their color is quantified by the color bar in the center. The longest segments without a deceleration point (SWDP) are plotted in black. The probability density function of the $\beta's$ for each experiment is shown on the left of the color scale Figure (c) shows the mean correlation curve between time τ and the distance $\langle D \rangle$. The black traced line represents the exponent equal to 1.0. Figure (d) shows the distribution of SWDP sizes in number of nodes per frequency of occurrence.

Wilmington, USA

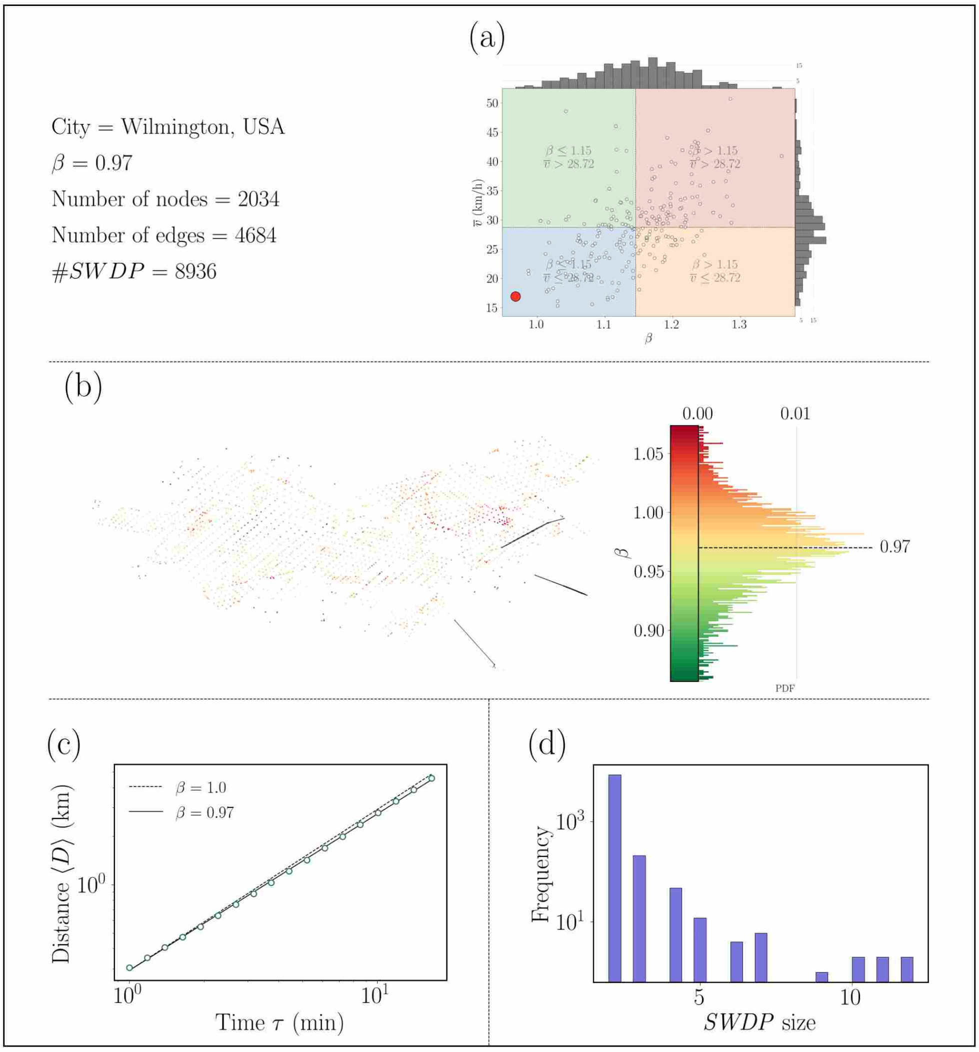

Fig. S199. Methodological sheet - Wilmington, USA. In (a) Each point represents a city, with mean exponent (β), on the x-axis, and mean speed $\overline{v}$ obtained in all trips made to calculate the exponent on the axis y. The histograms of the values of β and $\overline{v}$ are shown on the axes in the upper and right corners, respectively. The graph was segmented into four quadrants, in which the division is performed by the mean values of β and $\overline{v}$. The quadrants were colored and annotated according to the division criteria. The red dot represents the location of Wilmington, USA. In (b) taking all the nodes of Wilmington, USA as origin, the dots are colored as a function of their exponent value and their color is quantified by the color bar in the center. The longest segments without a deceleration point (SWDP) are plotted in black. The probability density function of the $\beta's$ for each experiment is shown on the left of the color scale Figure (c) shows the mean correlation curve between time τ and the distance $\langle D \rangle$. The black traced line represents the exponent equal to 1.0. Figure (d) shows the distribution of SWDP sizes in number of nodes per frequency of occurrence.

Winchester, UK

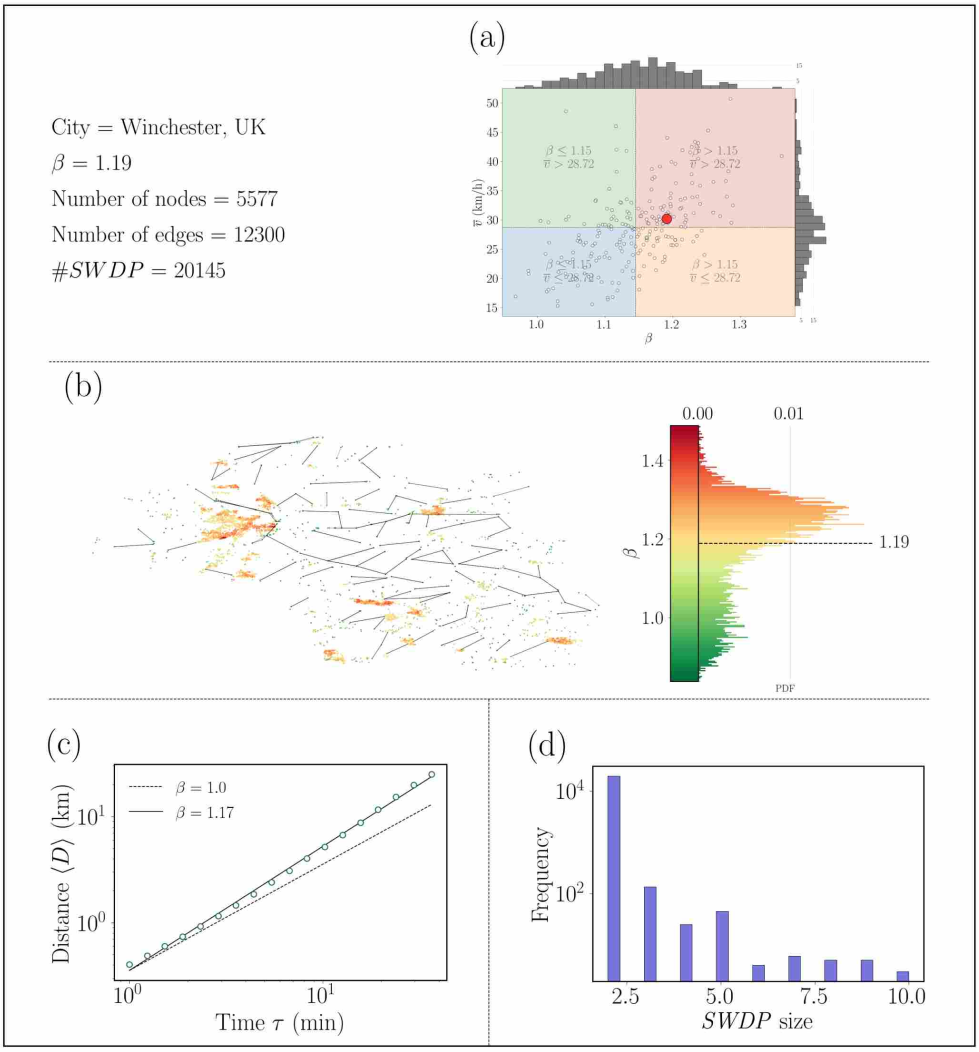

Fig. S200. Methodological sheet - Winchester, UK. In (a) Each point represents a city, with mean exponent (β), on the x-axis, and mean speed $\overline{v}$ obtained in all trips made to calculate the exponent on the axis y. The histograms of the values of β and $\overline{v}$ are shown on the axes in the upper and right corners, respectively. The graph was segmented into four quadrants, in which the division is performed by the mean values of β and $\overline{v}$. The quadrants were colored and annotated according to the division criteria. The red dot represents the location of Winchester, UK. In (b) taking all the nodes of Winchester, UK as origin, the dots are colored as a function of their exponent value and their color is quantified by the color bar in the center. The longest segments without a deceleration point (SWDP) are plotted in black. The probability density function of the $\beta's$ for each experiment is shown on the left of the color scale Figure (c) shows the mean correlation curve between time τ and the distance $\langle D \rangle$. The black traced line represents the exponent equal to 1.0. Figure (d) shows the distribution of SWDP sizes in number of nodes per frequency of occurrence.

Wollongong, Australia

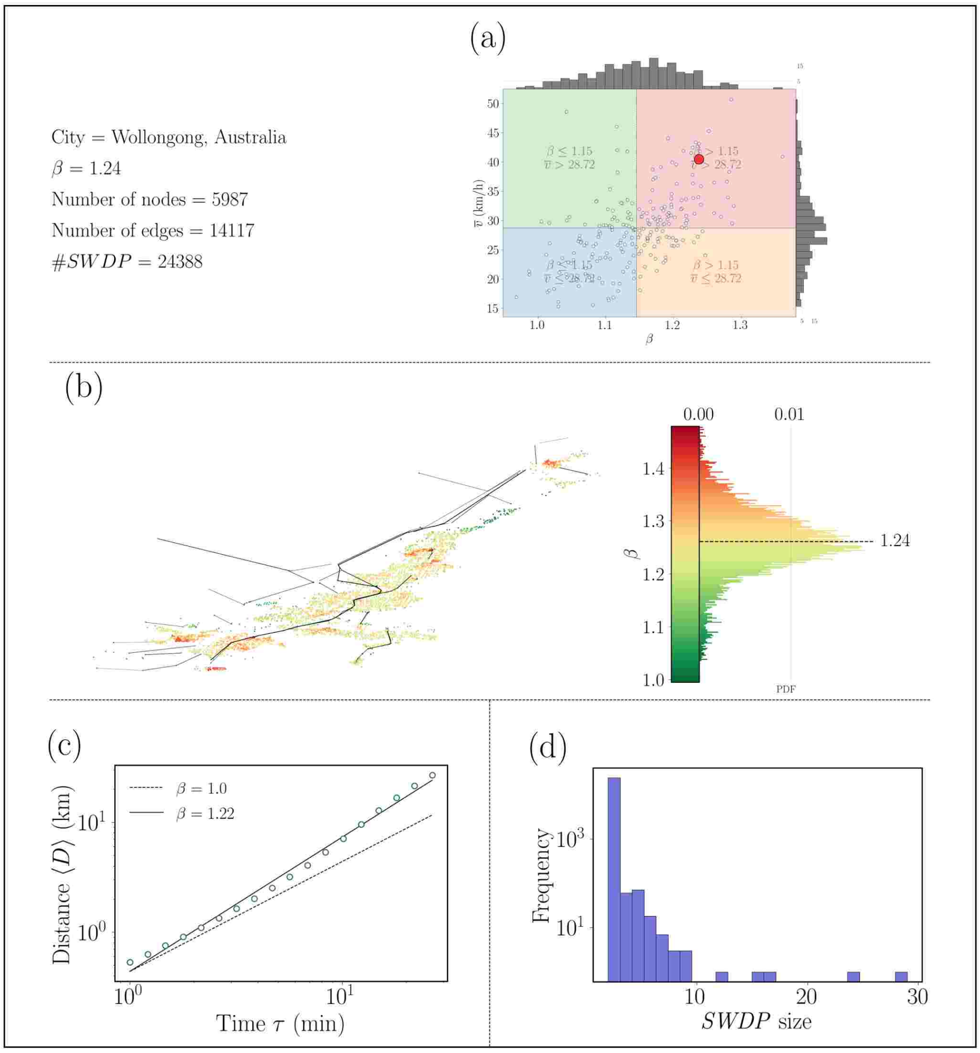

Fig. S201. Methodological sheet - Wollongong, Australia. In (a) Each point represents a city, with mean exponent (β), on the x-axis, and mean speed $\overline{v}$ obtained in all trips made to calculate the exponent on the axis y. The histograms of the values of β and $\overline{v}$ are shown on the axes in the upper and right corners, respectively. The graph was segmented into four quadrants, in which the division is performed by the mean values of β and $\overline{v}$. The quadrants were colored and annotated according to the division criteria. The red dot represents the location of Wollongong, Australia. In (b) taking all the nodes of Wollongong, Australia as origin, the dots are colored as a function of their exponent value and their color is quantified by the color bar in the center. The longest segments without a deceleration point (SWDP) are plotted in black. The probability density function of the $\beta's$ for each experiment is shown on the left of the color scale Figure (c) shows the mean correlation curve between time τ and the distance $\langle D \rangle$. The black traced line represents the exponent equal to 1.0. Figure (d) shows the distribution of SWDP sizes in number of nodes per frequency of occurrence.

Wuhan, China

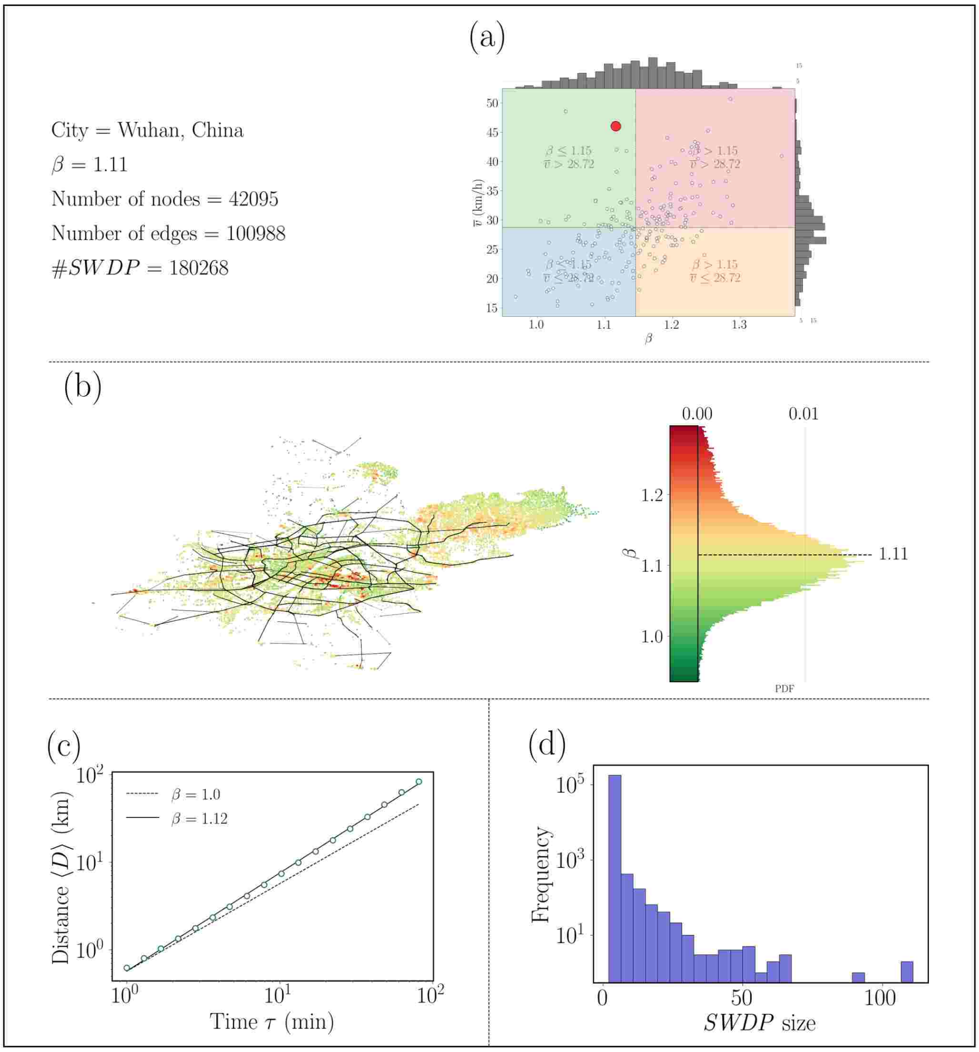

Fig. S202. Methodological sheet - Wuhan, China. In (a) Each point represents a city, with mean exponent (β), on the x-axis, and mean speed $\overline{v}$ obtained in all trips made to calculate the exponent on the axis y. The histograms of the values of β and $\overline{v}$ are shown on the axes in the upper and right corners, respectively. The graph was segmented into four quadrants, in which the division is performed by the mean values of β and $\overline{v}$. The quadrants were colored and annotated according to the division criteria. The red dot represents the location of Wuhan, China. In (b) taking all the nodes of Wuhan, China as origin, the dots are colored as a function of their exponent value and their color is quantified by the color bar in the center. The longest segments without a deceleration point (SWDP) are plotted in black. The probability density function of the $\beta's$ for each experiment is shown on the left of the color scale Figure (c) shows the mean correlation curve between time τ and the distance $\langle D \rangle$. The black traced line represents the exponent equal to 1.0. Figure (d) shows the distribution of SWDP sizes in number of nodes per frequency of occurrence.

Yantai, China

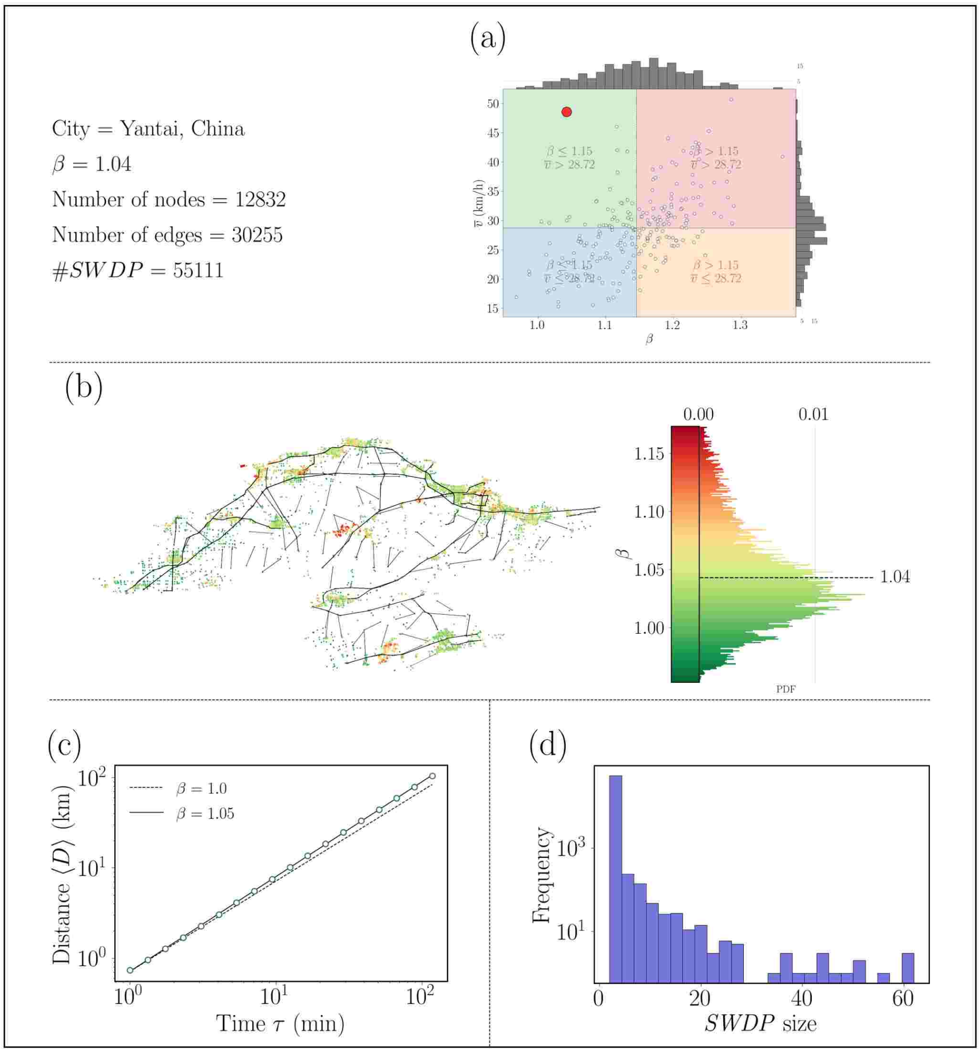

Fig. S203. Methodological sheet - Yantai, China. In (a) Each point represents a city, with mean exponent (β), on the x-axis, and mean speed $\overline{v}$ obtained in all trips made to calculate the exponent on the axis y. The histograms of the values of β and $\overline{v}$ are shown on the axes in the upper and right corners, respectively. The graph was segmented into four quadrants, in which the division is performed by the mean values of β and $\overline{v}$. The quadrants were colored and annotated according to the division criteria. The red dot represents the location of Yantai, China. In (b) taking all the nodes of Yantai, China as origin, the dots are colored as a function of their exponent value and their color is quantified by the color bar in the center. The longest segments without a deceleration point (SWDP) are plotted in black. The probability density function of the $\beta's$ for each experiment is shown on the left of the color scale Figure (c) shows the mean correlation curve between time τ and the distance $\langle D \rangle$. The black traced line represents the exponent equal to 1.0. Figure (d) shows the distribution of SWDP sizes in number of nodes per frequency of occurrence.

Yaoundé, Cameroon

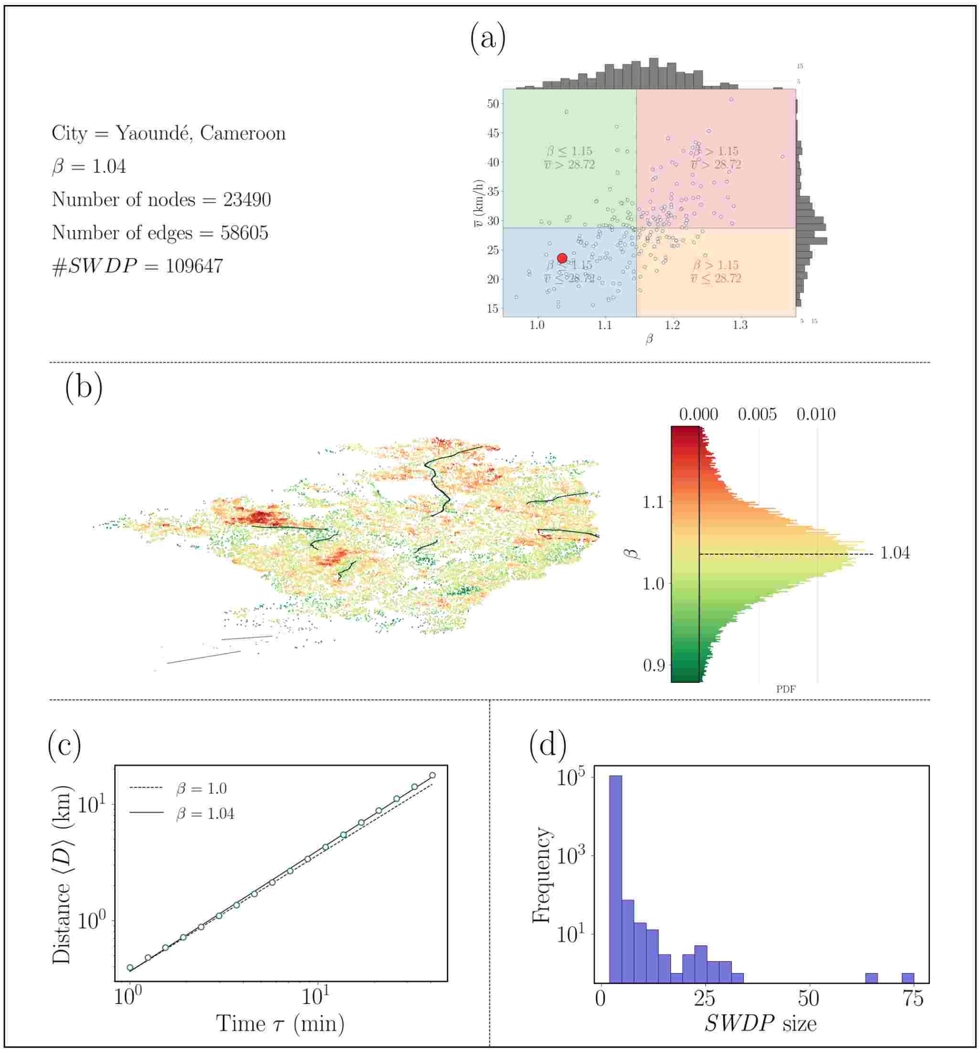

Fig. S204. Methodological sheet - Yaoundé, Cameroon. In (a) Each point represents a city, with mean exponent (β), on the x-axis, and mean speed $\overline{v}$ obtained in all trips made to calculate the exponent on the axis y. The histograms of the values of β and $\overline{v}$ are shown on the axes in the upper and right corners, respectively. The graph was segmented into four quadrants, in which the division is performed by the mean values of β and $\overline{v}$. The quadrants were colored and annotated according to the division criteria. The red dot represents the location of Yaoundé, Cameroon. In (b) taking all the nodes of Yaoundé, Cameroon as origin, the dots are colored as a function of their exponent value and their color is quantified by the color bar in the center. The longest segments without a deceleration point (SWDP) are plotted in black. The probability density function of the $\beta's$ for each experiment is shown on the left of the color scale Figure (c) shows the mean correlation curve between time τ and the distance $\langle D \rangle$. The black traced line represents the exponent equal to 1.0. Figure (d) shows the distribution of SWDP sizes in number of nodes per frequency of occurrence.

Zagreb, Croatia

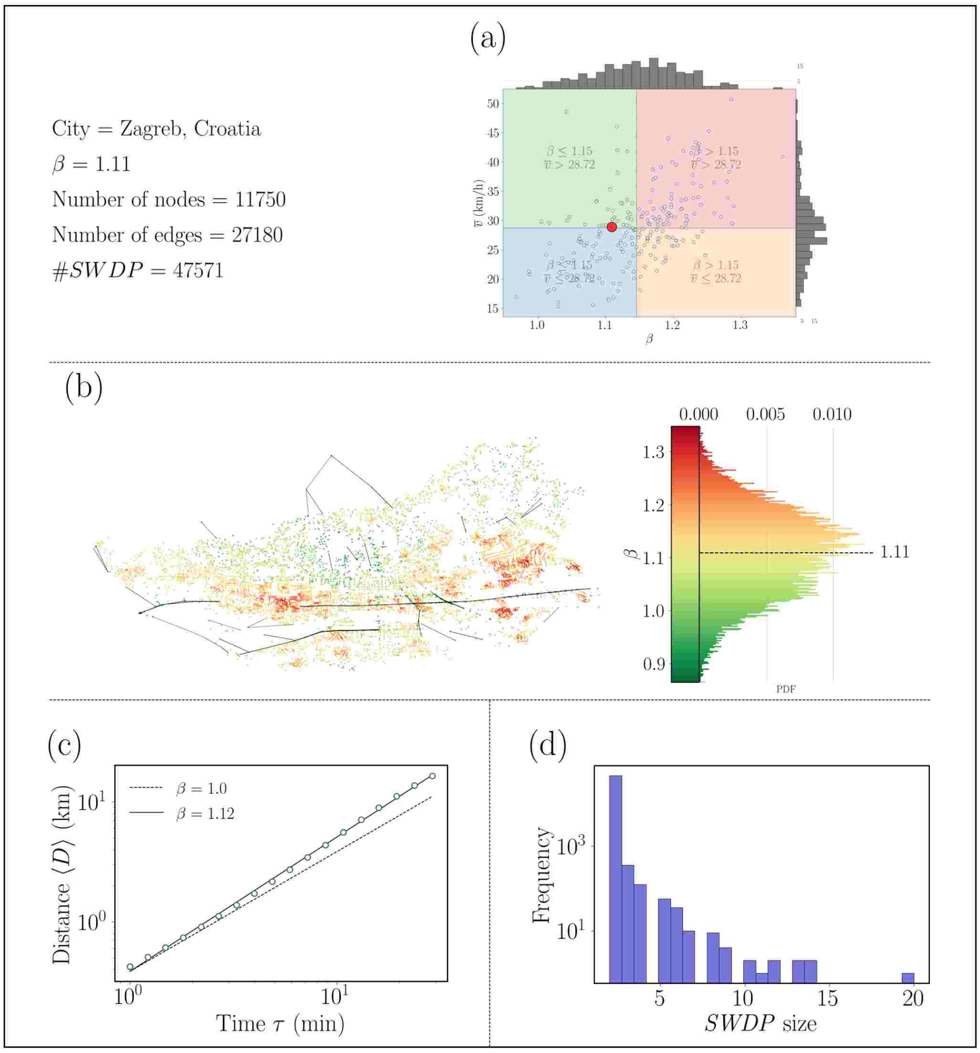

Fig. S205. Methodological sheet - Zagreb, Croatia. In (a) Each point represents a city, with mean exponent (β), on the x-axis, and mean speed $\overline{v}$ obtained in all trips made to calculate the exponent on the axis y. The histograms of the values of β and $\overline{v}$ are shown on the axes in the upper and right corners, respectively. The graph was segmented into four quadrants, in which the division is performed by the mean values of β and $\overline{v}$. The quadrants were colored and annotated according to the division criteria. The red dot represents the location of Zagreb, Croatia. In (b) taking all the nodes of Zagreb, Croatia as origin, the dots are colored as a function of their exponent value and their color is quantified by the color bar in the center. The longest segments without a deceleration point (SWDP) are plotted in black. The probability density function of the $\beta's$ for each experiment is shown on the left of the color scale Figure (c) shows the mean correlation curve between time τ and the distance $\langle D \rangle$. The black traced line represents the exponent equal to 1.0. Figure (d) shows the distribution of SWDP sizes in number of nodes per frequency of occurrence.

Zaragoza, Spain

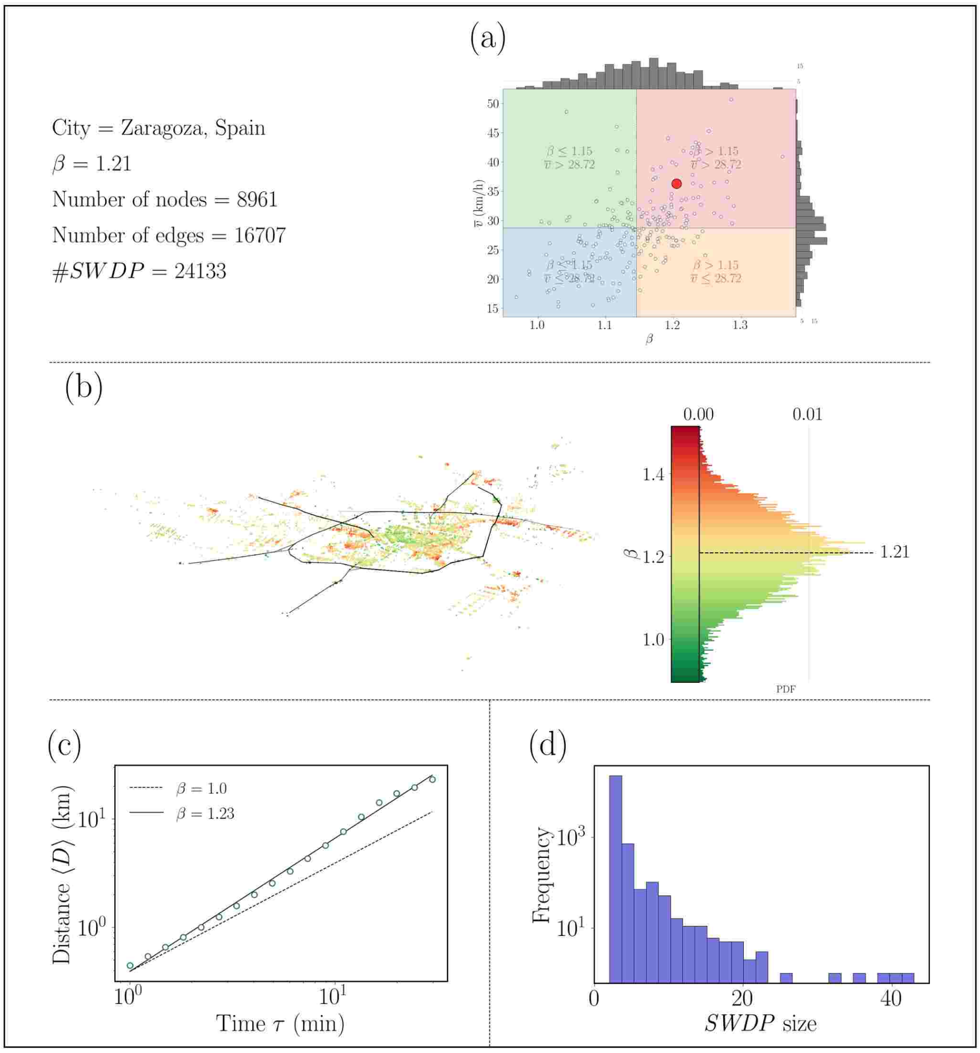

Fig. S206. Methodological sheet - Zaragoza, Spain. In (a) Each point represents a city, with mean exponent (β), on the x-axis, and mean speed $\overline{v}$ obtained in all trips made to calculate the exponent on the axis y. The histograms of the values of β and $\overline{v}$ are shown on the axes in the upper and right corners, respectively. The graph was segmented into four quadrants, in which the division is performed by the mean values of β and $\overline{v}$. The quadrants were colored and annotated according to the division criteria. The red dot represents the location of Zaragoza, Spain. In (b) taking all the nodes of Zaragoza, Spain as origin, the dots are colored as a function of their exponent value and their color is quantified by the color bar in the center. The longest segments without a deceleration point (SWDP) are plotted in black. The probability density function of the $\beta's$ for each experiment is shown on the left of the color scale Figure (c) shows the mean correlation curve between time τ and the distance $\langle D \rangle$. The black traced line represents the exponent equal to 1.0. Figure (d) shows the distribution of SWDP sizes in number of nodes per frequency of occurrence.

Zürich, Switzerland

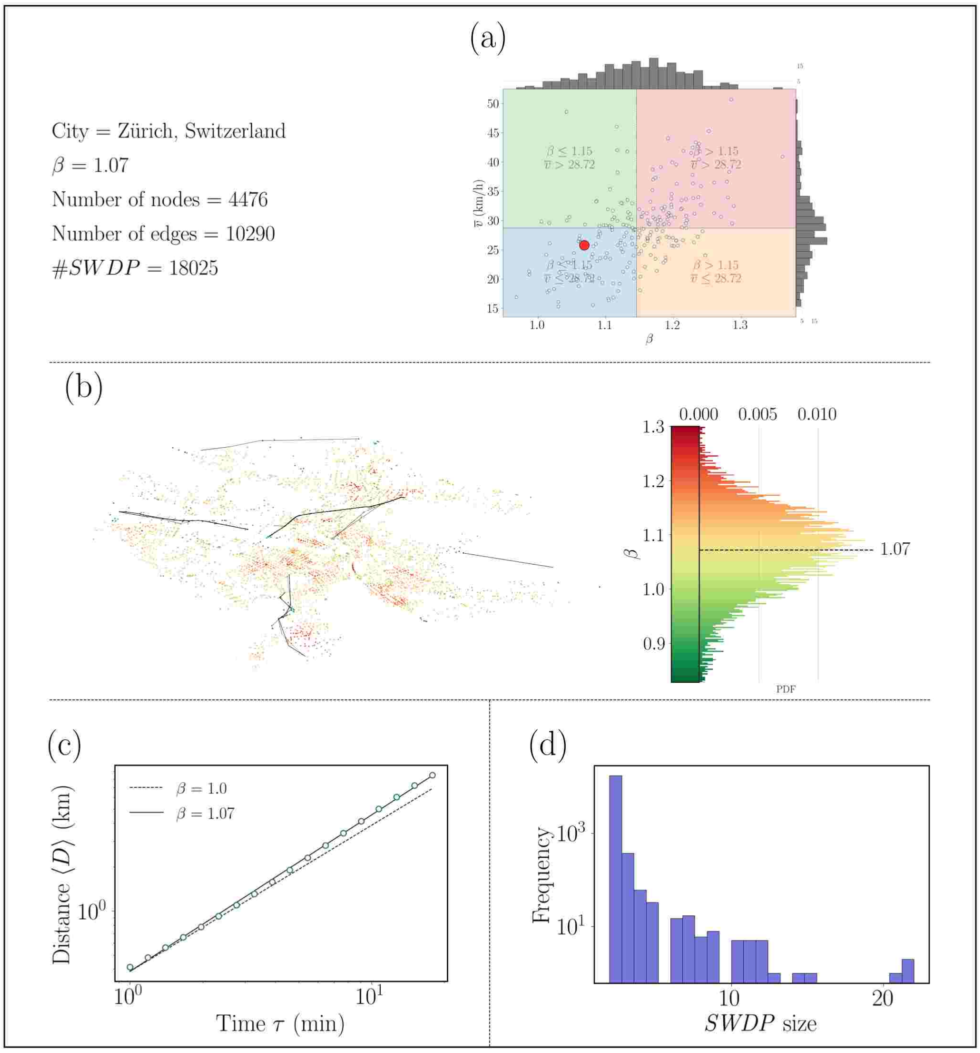

Fig. S207. Methodological sheet - Zürich, Switzerland. In (a) Each point represents a city, with mean exponent (β), on the x-axis, and mean speed $\overline{v}$ obtained in all trips made to calculate the exponent on the axis y. The histograms of the values of β and $\overline{v}$ are shown on the axes in the upper and right corners, respectively. The graph was segmented into four quadrants, in which the division is performed by the mean values of β and $\overline{v}$. The quadrants were colored and annotated according to the division criteria. The red dot represents the location of Zürich, Switzerland. In (b) taking all the nodes of Zürich, Switzerland as origin, the dots are colored as a function of their exponent value and their color is quantified by the color bar in the center. The longest segments without a deceleration point (SWDP) are plotted in black. The probability density function of the $\beta's$ for each experiment is shown on the left of the color scale Figure (c) shows the mean correlation curve between time τ and the distance $\langle D\rangle$. The black traced line represents the exponent equal to 1.0. Figure (d) shows the distribution of SWDP sizes in number of nodes per frequency of occurrence.
